# Polysulfurating reagent design for unsymmetrical polysulfide construction

**DOI:** 10.1038/s41467-018-04306-5

**Published:** 2018-06-06

**Authors:** Xiao Xiao, Jiahui Xue, Xuefeng Jiang

**Affiliations:** 1 Shanghai Key Laboratory of Green Chemistry and Chemical Process, Department of Chemistry, East China Normal University, 3663 North Zhongshan Road, Shanghai, 200062 China; 2 State Key Laboratory of Elemento-Organic Chemistry, Nankai University, Tianjin, 300071 China; 3 State Key Laboratory of Organometallic Chemistry, Shanghai Institute of Organic Chemistry, Chinese Academy of Sciences, 345 Lingling Road, Shanghai, 200032 China

## Abstract

From life science to material science, to pharmaceutical industry, and to food chemistry, polysulfides are vital structural scaffolds. However, there are limited synthetic methods for unsymmetrical polysulfides. Conventional strategies entail two pre-sulfurated cross-coupling substrates, R–S, with higher chances of side reactions due to the characteristic of sulfur. Herein, a library of broad-spectrum polysulfurating reagents, R–S–S–OMe, are designed and scalably synthesized, to which the R–S–S source can be directly introduced for late-stage modifications of biomolecules, natural products, and pharmaceuticals. Based on the hard and soft acids and bases principle, selective activation of sulfur-oxygen bond has been accomplished via utilizing proton and boride for efficient unsymmetrical polysulfuration. These polysulfurating reagents are highlighted with their outstanding multifunctional gram-scale transformations with various nucleophiles under mild conditions. A diversity of polysulfurated biomolecules, such as SS−(+)-δ-tocopherol, SS-sulfanilamide, SS-saccharides, SS-amino acids, and SSS-oligopeptides have been established for drug discovery and development.

## Abstract (web summary)

Synthetic methods to access unsymmetrical polysulfides are limited despite their high biological relevance. Here, the authors disclose a polysulfurating reagent affording unsymmetrical di- and tri-sulfides under mild conditions, and suitable for late-stage modification of natural products.

## Introduction

**Fig. 1:**
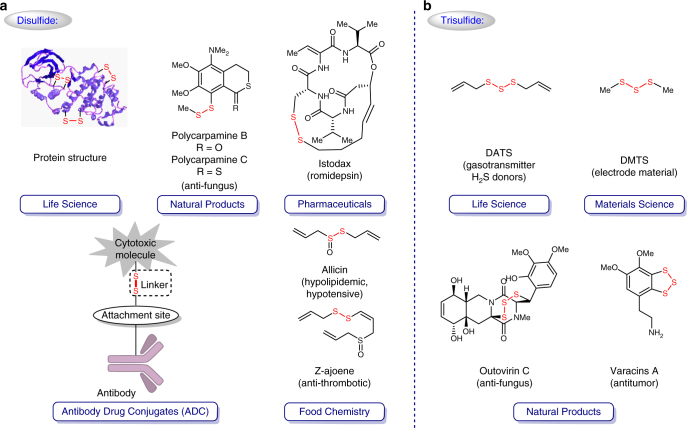
Significant polysulfides. **a** The importance of disulfide scaffolds in life science, natural products, pharmaceuticals, antibody drug conjugates, and food chemistry. **b** Functional trisulfide molecules

Disulfide scaffolds, containing two covalently linked sulfur atoms, are important molecular motifs in life science^1–6^, pharmaceutical science^7–15^, and food chemistry^16–18^ by virtue of their unique pharmacological and physiochemical properties (Fig. 1a). Disulfide bonds, for instance, in biomolecules take multifaceted roles in various biochemical redox processes to generate and regulate hormones, enzymes, growth factors, toxins, and immunoglobulins for very homeostasis and bio-signaling (e.g., metal trafficking); secondary and tertiary structures of proteins are also well formed and stabilized via the disulfide bridge^2–5^. In recent decades, potent bioactive natural products and pharmaceuticals possessing sulfur–sulfur bonds have been discovered, such as the antifungal polycarpamine family^7^, the anti-poliovirus epidithiodiketopiperazine (ETPs) family^8, 9^, romidepsin^10^, gliotoxin^11^, and some new histone deacetylase/methyltransferase inhibitors^12^, which, mechanism-wise, either sequester enzyme-cofactor zinc or generate highly reactive electrophiles to induce DNA strand scission. When it comes to antibody-drug conjugates (ADC), the disulfide bond has also been extensively utilized as a linker to deliver the active drug into the targeted cell after cleavage upon internalization of ADC^19–22^. Due to the higher intracellular concentration of free thiols (glutathione) than in the bloodstream, the sulfur–sulfur bonds can be selectively cleaved in the cytoplasm of cancer cell, thereby achieving the specified release of cytotoxic molecules. Notably, disulfide compounds in allium species plants can not only demonstrate vasorelaxation activity, but also inhibit ADP-induced platelet aggregation^16–18^.

Tri-sulfides have recently received considerable attention. To cite the allium-derived diallyl trisulfide (DATS) as an example, it serves as a gasotransmitter precursor and an excellent hydrogen sulfide donor, mediating and regulating the release of hydrogen sulfide upon physiological activation (Fig. 1b)^23, 24^. From the materials perspective, organotrisulfides, such as dimethyl trisulfide (DMTS) with a theoretical capacity of 849 mAhg^−1^, hold promise as high-capacity cathode materials for high-energy rechargeable lithium batteries^25^. It should also be pointed out that trisulfides do exist in bioactive natural products from marine invertebrates^7, 26–28^, such as the antitumor varacins A^26^ and the anti-fungus outovirin C^27^.

**Fig. 2:**
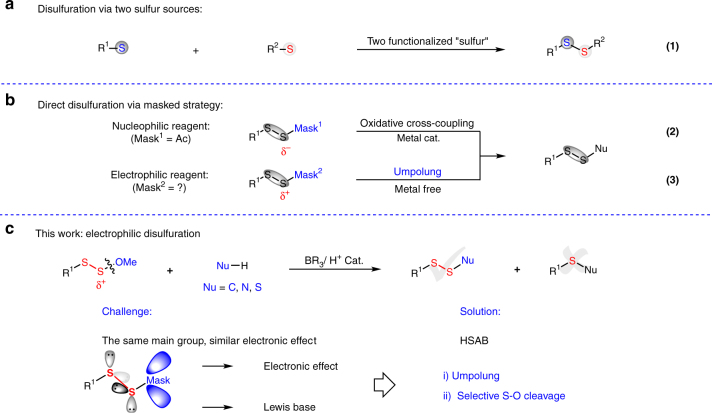
Strategies for polysulfide construction. **a** Traditional methodologies for unsymmetrical disulfide syntheses. **b** Masked strategy for disulfuration. **c** Electropilic disulfurating reagent for polysulfuration

Given the importance and predominance in pharmaceuticals and other bioactive compounds of polysulfurated structures, it is always sought-after to develop general polysulfuration protocols for synthetic purposes. Although typical methods for symmetrical disulfide preparation have been well developed^29^, the construction of unsymmetrical disulfides is still a challenging transformation due to the high reactivity of S–S bond^30–40^. In general, the synthesis of unsymmetrical disulfides can be achieved via an S_N_2 process between a thiol and a prefunctionalized thiol with leaving group^32–38^. Alternatively, one can employ either two different kinds of thiols with unavoidable formation of homocoupling byproducts^39^ or two distinct symmetrical disulfides with the use of rhodium(I) by Yamaguchi group^40^. Based on our continuous research in organic sulfur chemistry^41–48^, comproportionation between two distinct inorganic sulfur sources was utilized for unsymmetrical disulfides syntheses^49^. However, the strategy of aforementioned methods introduces disulfide bonds from two different kinds of sulfur-containing substrates, requiring more synthetic steps and leading to side-reactions due to both reactive thio-derivatives (Fig. 2a)^30–40, 49^. We intend to develop methodology which can introduce the RSS source with one disulfurating reagent at a later stage so as to provide great compatibility and several possibilities of polysulfuration. Hydropersulfide (RSSH) seems to be a prime disulfurating reagent, though it is unstable owing to its high reactivity^50, 51^. Two sulfur atoms were successfully introduced in one step via oxidative cross-couplings of acetyl masked disulfurating nucleophiles and organometallic reagents (Fig. 2b)^52^.

Nevertheless, there is a large demand for a universal disulfurating reagent, which is compatible with diverse coupling partners without transition-metal catalysis. The umpolung strategy, replacement of acetyl (RSS^−^) with methoxyl (RSS^+^) group, will afford the precursor of persulfide cation (Fig. 2c). Originating from the same main group, sulfur and oxygen possess similar electronic effect, which imposes a great challenge for selective cleavage of S–O bond with S–S bond untouched. Based on the hard and soft acids and bases (HSAB) principle^53^, we hypothesize that boride/proton can help to make the difference between S–S and S–O, in which the hard acid boride/proton prefers oxygen coordination. Herein, we disclose a polysulfurating reagent which can construct unsymmetrical disulfide and trisulfide products by utilizing a RSS source only on one substrate, which renders the late-stage functionalization feasible. Different nucleophilic regents, such as 1,3-dicarbonyl derivatives, electron-rich arenes, heteroarenes, amines, and thiols, had been smoothly coupled with disulfurating reagents under mild, transition-metal-free, and base-free conditions, especially suitable for the late-stage modification of natural products and pharmaceuticals.

## Results

### Optimization and synthesis of polysulfurating reagents

Initial studies commenced with the construction of designed electrophilic polysulfurating reagents. It was hypothesized that the electrophilic reagent could be obtained through hydropersulfide anion and methanol via oxidative cross-coupling. The polysulfurating reagent **2d** was obtained in 31% yield under the conditions of copper(II) as catalyst, 2,2′-bipyridine as ligand, and PhI(OAc)_2_ as oxidant (Table 1, entry 1). The bulky iodonium salt PhI(OPiv)_2_ was the oxidant of choice in this conversion (Table 1, entries 1–3). Systematic investigations of ligands showed that 4,7-diphenyl-1,10-phenanthroline helped to increase the yield of **2d** to 77% (Table 1, entries 3–5). Further study demonstrated that slightly lower temperature was important for keeping product **2d** stable in this system (Table 1, entry 6). Catalyst loading was lowered with the same efficiency of the transformation (Table 1, entries 7–9). The optimal conditions were found to involve treatment of **1d** with 5 mol% of catalyst, 10 mol% of ligand **L1**, 2.2 equivalents of bis(*tert*-butylcarbonyloxy)iodobenzene, and 1.0 equivalent of lithium carbonate in 0.1 M methanol at 20 °C, which afforded electrophilic polysulfurating reagent **2d** in the yield of 88% (Table 1, entry 10). When the oxidant bis(*tert*-butylcarbonyloxy)iodobenzene was reduced to 1.9 equivalents, the yield of **2d** was dropped sharply to 65% (Table 1, entry 11).

**Table 1:** Optimization of polysulfide reagents^a,b^


Entry	CuSO_4_ (mol%)	Ligand (mol%)	PhI(OPiv)_2_ (equiv)	Temp (°C)	Time (h)	Yields (%)
1^c^	10	bpy (10)	2.5	25	11	31
2^d^	10	bpy (10)	2.5	25	11	ND
3	10	bpy/ phen (10)	2.5	25	11	50/53
4	10	L1 (10)	2.5	25	11	77
5	10	L2/L3/L4 (10)	2.5	25	11	70/63/68
6	10	L1 (10)	2.5	20	13	86
7	5	L1 (10)	2.5	20	13	86
8	2.5	L1 (10)	2.5	20	13	79
9	5	L1 (5)	2.5	20	13	76
10	5	L1 (10)	2.2	20	13	88
11	5	L1 (10)	1.9	20	13	65


^a^Conditions: 1d (0.2 mmol, 1 equiv), CuSO_4_·5H_2_O, Ligand, Li_2_CO_3_ and PhI(OPiv)_2_ were added to MeOH (2 mL) at 20 °C for 13 h

^b^Isolated yields

^c^PhI(OAc)_2_ was instead of PhI(OPiv)_2_

^d^PhI(OTFA)_2_ was instead of PhI(OPiv)_2_

With the optimized conditions in hand, the syntheses of electrophilic polysulfurating reagents were comprehensively investigated. A scale of 5 mmol operation was practicably performed, decreasing catalyst loading to 0.25 mol% (for details see the Supplementary Table 2). Various acetyl substituted disulfides were readily transformed to methoxyl substituted disulfides (Table. 2). Initially, the reagents bearing both electron-donating and electron-withdrawing groups on aromatic rings were successfully obtained (Table 2, **2a**–**2f**). Notably, 1.84 g of **2d** was achieved in a yield of 87% with 10 mmol scale operation (Table 2, **2d**). The arene substituted with chloromethylene group was compatible under the standard conditions (Table 2, **2e**–**2f**). Reactions involving secondary benzyl and propargyl derivatives were carried out smoothly (Table 2, **2g**–**2h**). When aliphatic substrates were evaluated, the corresponding products were formed efficiently (Table 2, **2i**–**2m**). The scope was further demonstrated through the successful syntheses of bis-disulfurating reagents (Table 2, **2n**–**2o**). Notably, the modification of saccharides and amino acids were also converted into corresponding disulfurating reagents (Table 2, **2p**–**2t**). These reagents are fairly stable without deterioration when stored in a refrigerator (−18 °C) for half a year. Around 20% of these reagents will decompose at room temperature (+25 °C) after 1 week.

**Table 2:** The scope of polysulfurating reagents^a,b^

	

^a^1 (5 mmol, 1 equiv), CuSO_4_·5H_2_O (0.0125 mol, 0.125 mol%), L1 (0.025 mol, 0.25 mol%), Li_2_CO_3_ (5 mmol, 1 equiv) and PhI(OPiv)_2_ (11 mmol, 2.2 equiv) were added to MeOH (10 mL) at 20 °C for 15 h

^b^Isolated yields

^c^1 (10 mmol, 1 equiv) and MeOH (10 mL) were used

### Polysulfuration with designed reagents

With the class of disulfurating reagents in hand, the construction of unsymmetrical disulfides and trisulfides was consequently explored. We initiated our efforts with 1,3-dicarbonyl compounds due to their excellent nucleophilic property. Based on the HSAB principle, the coupling between acetylacetone and reagent **2d** has been explored under the assistance of the hard acid Tris(perfluorophenyl)borane as a catalyst (for details see the Supplementary Table 3). Various 1,3-dicarbonyl structures effectively afford disulfuration catalyzed with the combination of tris(perfluorophenyl)borane and 4-methoxypyridine (Table 3). Acyclic and cyclic 1,3-dicarbonyl substrates were smoothly converted to the desired disulfides (Table 3, **3a**–**3d**). The configuration of **3a** was further confirmed through X-ray crystallographic analysis. Aliphatic and propargyl derivatives were compatible in this process (Table 3, **3e**–**3h**). Significantly, disulfurating reagents bearing both saccharide and amino acid groups accomplished this transformation efficiently with two parts connected via the disulfur linkage (Table 3, **3i**–**3l**).

**Table 3:** Disulfuration with carbon nucleophiles ^a,b^

	

^a^Standard conditions A: NuH (0.22 mmol, 1.1 equiv), 2 (0.2 mmol, 1 equiv), B(C_6_F_5_)_3_ (0.01 mmol, 5 mol%) and 4-MeOPy (0.01 mmol, 5 mol%) were added to DCE (0.25 mL) at r.t. for 22 h. Standard conditions B: NuH (0.3 mmol, 1.5 equiv), 2 (0.2 mmol, 1 equiv) and B(C_6_F_5_)_3_ (0.01 mmol, 5 mol%) were added to PhMe (0.5 mL) at 0 °C for 24 h. Standard conditions C: NuH (0.3 mmol, 1.5 equiv), 2 (0.2 mmol, 1 equiv) and MeSO_3_H (0.02 mmol, 10 mol%) were added to ^*t*^AmylOH (0.5 mL) at 0 °C for 5–24 h

^b^Isolated yields

^c^r.t. was instead of 0 °C

^d^B(C_6_F_5_)_3_ (0.002 mmol, 1 mol%) was used

^e^B(C_6_F_5_)_3_ (0.01 mmol, 0.2 mol%) was used

^f^B(C_6_F_5_)_3_ (0.004 mmol, 2 mol%) were added to PhMe (0.25 mL) at r.t. for 24 h

^g^NuH (0.22 mmol, 1.1 equiv), 2 (0.2 mmol, 1 equiv) and B(C_6_F_5_)_3_ (0.004 mmol, 2 mol%) were added to PhMe (0.25 mL) at 0 °C for 24 h. Ar = 4-CNC_6_H_4_

Following the activation mode, electron-rich aromatics were readily accommodated under standard conditions (Table 3, **4a**–**4d**). (+)-δ-Tocopherol, a significant bioactive molecule, could be disulfurated directly despite the presence of free hydroxyl group (Table 3, **4c**–**4d**). Indole and pyrrole, ubiquitous in natural products and pharmaceuticals, are excellent coupling partners as well. Indoles bearing both electron-rich and -deficient functional groups proceeded smoothly with disulfurating reagents to afford the corresponding indolyl-disulfides on 3-position (Table 3, **5a-5p**). A bis-disulfurating electrophile also afforded the corresponding twofold disulfur-containing molecule efficiently (Table 3, **5q**). Saccharide and amino acid structures were directly installed with indoles via the disulfide linker (Table 3, **5r-5u**). A gram-scale operation was performed with 5 mmol of **2d** under the catalysis of 1 mol% of B(C_6_F_5_)_3_ affording **5o** in 93% yield (1.38 g), which structure was further confirmed through X-ray analysis. In particular, iodo- and formyl-substituted indoles were also compatible in this transformation (Table 3, **5m-5n**). Pyrroles substituted on different positions were treated to the disulfuration conditions, successfully providing desired products as well (Table 3, **5v-5y**).

Subsequently, amine partners were systematically varied providing access to a wide range of functional aza-disulfide in the presence of 2.5 mol% of tris(perfluorophenyl)borane. The anilines substituted with electron-withdrawing and electron-donating functional groups afforded the desired aza-disulfides in moderate to excellent yields (Table 4, **6a-6f**). The secondary amines proceeded in this transformation, affording corresponding products in favorable yields (Table 4, **6g-6h**). Notably, allyl, propargyl and heteroaromatic amines were all efficiently transformed to the corresponding products (Table 4, **6i-6k**). Sulfanilamides, as a significant type of antibiotic, could be modified with the designed persulfurating reagent in good to excellent yields (Table 4, **6m-6s**). Lenalidomide, a myeloma drug, was installed with the disulfide under mild reaction conditions (Table 4, **6t**). Furthermore, functional disulfurating electrophiles, modified with saccharide and amino acid groups, were furnished with the substituted disulfur amine linker (Table 4, **6u-6y**). The structure of **6a** was further confirmed by X-ray analysis. In order to validate the efficiency and practicability of this aza-disulfuration, 0.25 mol% catalyst loading was launched on a gram-scale reaction to afford **6a** in 81% yield (1.1 g).

**Table 4:** Disulfuration with heteroatomic nucleophiles ^a,b^

	

^a^Standard conditions D: NuH (0.22 mmol, 1.1 equiv), 2 (0.2 mmol, 1 equiv) and B(C_6_F_5_)_3_ (0.005 mmol, 2.5 mol%) were added to PhMe (0.5 mL) at r.t. for 24 h. Standard conditions E: NuH (0.22 mmol, 1.1 equiv) and 2 (0.2 mmol, 1 equiv) were added to DCM (2.0 mL) at r.t. for 8 h

^b^Isolated yields

^c^B(C_6_F_5_)_3_ (0.0125 mmol, 0.25 mol%) was used

^d^CH_3_CN was used as solvent

^e^NuH (0.2 mmol, 1 equiv), 2 (0.3 mmol, 1.5 equiv) and B(C_6_F_5_)_3_ (0.005 mmol, 2.5 mol%) were added to DMF at r.t. for 24 h

^f^B(C_6_F_5_)_3_ (2.5 mol%) was added at r.t. for 5 h

^g^B(C_6_F_5_)_3_ (2.5 mol%) and DCM (0.5 mL) was added

^h^B(C_6_F_5_)_3_ (2.5 mol%) and DMF (0.5 mL) was added

^i^24 h. Ar = 4-CNC_6_H_4_, R = (CH_2_)_9_Me

Trisulfuration was readily achieved with thiols as a nucleophile (Table 4, **7a-7q**). Even sterically bulky aliphatic thiols, *tert*-butylthiol and 1-adamantanethiol, displayed excellent trisulfurations (Table 4, **7d**, and **7l**). The structure of **7d** was further confirmed via X-ray analysis. A gram-scale production for **7g** could be performed in 92% yield practically. Thiols substituted with vinyl, polyfluoroalkyl, silyl, and hydroxyl groups, and heterocycles were all tolerated in this transformation, being converted to the unsymmetrical trisulfides, respectively (Table 4, **7h-7k**, and **7o**). Even dithiols efficiently formed the corresponding twofold trisulfur-containing products in good yields (Table 4, **7s-7t**). Aliphatic trisulfurations could be achieved in high yields (Table 4, **7u-7v**). It should be noted that trisulfides containing saccharide and cysteine fragments were readily formed through these reagents (Table 4, **7r**, **7w-7ac**). Cysteine was successfully utilized for constructing trisulfur-containing amino acids and oligopeptides, which might provide another access for peptide drug discovery (Table 4, **7aa-7ac**).

## Discussion

In summary, a class of stable and broad-spectrum polysulfurating reagents with masked strategy has been designed and a general polysulfurating methodology has been established under mild conditions, which can directly introduce two sulfur atoms into functional molecules. The designed reagents were compatible with a considerable range of significant biomolecules, such as saccharides, amino acids, peptides and variety of heterocycles. This protocol showcases the wide utility of both carbon and nitrogen nucleophiles resulting in the functional disulfides. Furthermore, the trisulfuration provides a convenient and efficient method for sulfur-containing drug discovery. Further studies on modification of biomolecules and pharmaceuticals with these disulfurating reagents are still ongoing.

## Methods

### General methods

See Supplementary Methods for further details.

### General procedure for syntheses of disulfurating reagents 2

To a Schlenk tube were added RSSAc **1 **(5 mmol, 1 equivalent), CuSO_4_·5H_2_O (0.0125 mmol, 0.25 mol%, 3.2 mg), **L1** (0.025 mmol, 0.5 mol%, 8.1 mg), Li_2_CO_3_ (5 mmol, 1 equivalent, 370 mg), PhI(OPiv)_2_ (11 mmol, 2.2 equivalents, 4.47 g) and undried MeOH (10 mL), the mixture was stirred at 20 °C under normal conditions for 15 h. Then the mixture was quenched by saturated NaHCO_3_ and extracted by DCM before the organic phase was concentrated under vacuum without adding silica gel. Purification by column chromatography afforded the desired product.

### General procedure for syntheses of disulfides 3

To a Schlenk tube were added 1,3-dicarbonyl compound (0.22 mmol, 1.1 equivalents), B(C_6_F_5_)_3_ (0.01 mmol, 5 mol%, 5.2 mg), 4-MeO-pyridine (0.01 mmol, 5 mol%, 1.1 mg), RSSOMe **2** (0.2 mmol, 1 equivalent), and 1,2-dichloroethane (0.25 mL), the mixture was stirred at r.t. for 22 h before it was concentrated under vacuum. Purification by column chromatography afforded the desired product.

### General procedure for syntheses of disulfides 4

To a Schlenk tube were added arene (0.3 mmol, 1.5 equivalents), B(C_6_F_5_)_3_ (0.01 mmol, 5 mol%, 5.2 mg), RSSOMe **2** (0.2 mmol, 1 equivalent), and toluene (0.5 mL), the mixture was stirred at 0 °C or r.t. for 24–60 h before it was concentrated under vacuum. Purification by column chromatography afforded the desired product.

### General procedure for syntheses of disulfides 5

Method A: To a Schlenk tube were added indole (0.3 mmol, 1.5 equivalents), MeSO_3_H (0.02 mmol, 10 mol%, 2 mg), RSSOMe **2** (0.2 mmol, 1 equivalent), and *t*-AmylOH (0.5 mL), the mixture was stirred at r.t. for 24 h before it was concentrated under vacuum. Purification by column chromatography afforded the desired product. Method B: To a Schlenk tube were added indole (0.22 mmol, 1.1 equivalents), B(C_6_F_5_)_3_ (0.004 mmol, 2 mol%, 2.1 mg), RSSOMe (0.2 mmol, 1 equivalent), and toluene (0.25 mL), the mixture was stirred at 0 °C or r.t. for 24 h before it was concentrated under vacuum. Purification by column chromatography afforded the desired product.

### General procedure for syntheses of aza-disulfides 6

To a Schlenk tube were added amine (0.22 mmol, 1.1 equivalents), B(C_6_F_5_)_3_ (0.01 mmol, 2.5 mol%, 2.6 mg), RSSOMe **2** (0.2 mmol, 1 equivalent), and toluene (0.5 mL), the mixture was stirred at 0 °C or r.t. for 24 h before it was concentrated under vacuum. Purification by column chromatography afforded the desired product.

### General procedure for syntheses of trisulfides 7

To a Schlenk tube were added thiol (0.22 mmol, 1.1 equivalents), B(C_6_F_5_)_3_, RSSOMe **2** (0.2 mmol, 1 equivalent), and DCM (0.5 mL), the mixture was stirred at r.t. under N_2_ atmosphere for 5–8 h before it was concentrated under vacuum. Purification by column chromatography afforded the desired product.

### Data availability

The X-ray crystallographic coordinates for structures reported in this study have been deposited at the Cambridge Crystallographic Data Centre (CCDC), under deposition number CCDC 1565934 (**3a**), 1565935(**5o**), 1565936 (**6a**) and 1565937 (**7d**). These data can be obtained free of charge from The Cambridge Crystallographic Data Centre via www.ccdc.cam.ac.uk/data_request/cif. The authors declare that all other data supporting the findings of this study are available within the article and Supplementary Information files, and also are available from the corresponding author on reasonable request.

## Electronic supplementary material

Supplementary Information

Peer Review File

## References

[1] Narayan M, Welker E, Wedemeyer WJ, Scheraga HD (2000). Oxidative folding of proteins. Acc. Chem. Res..

[2] A.-Cebollada J, Kosuri P, R.-Pardo JA, Fernández JM (2011). Direct observation of disulfide isomerization in a single protein. Nat. Chem..

[3] Wommack AJ (2014). Discovery and characterization of a disulfide-locked *C*_2_-symmetric defensin peptide. J. Am. Chem. Soc..

[4] Góngora-Benítez M, Tulla-Puche J, Albericio F (2014). Multifaceted roles of disulfide bonds. Peptides as therapeutics. Chem. Rev..

[5] Lu S (2015). Mapping native disulfide bonds at a proteome scale. Nat. Methods.

[6] Landeta C (2015). Compounds targeting disulfide bond forming enzyme DsbB of gram-negative bacteria. Nat. Chem. Biol..

[7] Jiang CS, Müller WEG, Schröder HC, Guo YW (2012). Disulfide- and multisulfide-containing metabolites from marine organisms. Chem. Rev..

[8] Nicolaou KC (2012). Synthesis and biological evaluation of epidithio-, epitetrathio-, and bis-(methylthio)diketopiperazines: synthetic methodology, enantioselective total synthesis of epicoccin G, 8,8^’^-*epi*-ent-rostratin B, gliotoxin, gliotoxin G, emethallicin E, and haematocin and discovery of new antiviral and antimalarial agents. J. Am. Chem. Soc..

[9] Chankhamjon P (2014). Biosynthesis of the halogenated mycotoxin aspirochlorine in koji mold involves a cryptic amino acid conversion. Angew. Chem. Int. Ed..

[10] Nielsen DS (2017). Orally absorbed cyclic peptides. Chem. Rev..

[11] Scharf DH (2011). A dedicated glutathione *S*-transferase mediates carbon-sulfur bond formation in gliotoxin biosynthesis. J. Am. Chem. Soc..

[12] Liu Y (2017). Development of the first generation of disulfide-based subtype-selective and potent covalent pyruvate dehydrogenase kinase 1 (PDK1) inhibitors. J. Med. Chem..

[13] Zorzi A, Deyle K, Heinis C (2017). Cyclic peptide therapeutics: past, present and future. Curr. Opin. Chem. Biol..

[14] Brocchini S (2006). PEGylation of native disulfide bonds in proteins. Nat. Protoc..

[15] Stephanopoulos N, Francis MB (2011). Choosing an effective protein bioconjugation strategy. Nat. Chem. Biol..

[16] Block E, Ahmad S, Catalfamo JL, Jain MK, Apitz-Castro RT (1986). Antithrombotic organosulfur compounds from garlic: structural, mechanistic, and synthetic studies. J. Am. Chem. Soc..

[17] Block E, Bayer T, Naganathan S, Zhao SH (1996). Allium chemistry: synthesis and sigmatropic rearrangements of alk(en)yl 1-propenyl disulfide *S*-oxides from cut onion and garlic. J. Am. Chem. Soc..

[18] Hanschen FS, Lamy E, Schreiner M, Rohn S (2014). Reactivity and stability of glucosinolates and their breakdown products in foods. Angew. Chem. Int. Ed..

[19] Senter PD (2009). Potent antibody drug conjugates for cancer therapy. Curr. Opin. Chem. Biol..

[20] Chari RVJ, Miller ML, Widdison WC (2014). Antibody–drug conjugates: an emerging concept in cancer therapy. Angew. Chem. Int. Ed..

[21] Staben LR (2016). Targeted drug delivery through the traceless release of tertiary and heteroaryl amines from antibody-drug conjugates. Nat. Chem..

[22] Beck A, Goetsch L, Dumontet C, Corvaïa N (2017). Strategies and challenges for the next generation of antibody-drug conjugates. Nat. Rev. Drug Discov..

[23] Cerda MM, Hammers MD, Earp MS, Zakharov LN, Pluth MD (2017). Applications of synthetic organic tetrasulfides as H_2_S donors. Org. Lett..

[24] Benavides GA (2007). Hydrogen sulfide mediates the vasoactivity of garlic. Proc. Natl Acad. Sci. USA.

[25] Wu M (2016). Organotrisulfide: a high capacity cathode material for rechargeable lithium batteries. Angew. Chem. Int. Ed..

[26] Makarieva TN (1995). Varacin and three new marine antimicrobial polysulfides from the far-eastern ascidian polycitorsp. J. Nat. Prod..

[27] Oku N, Matsunaga S, Fusetani N (2003). Shishijimicins A-C, novel enediyne antitumor antibiotics from the ascidian didemnum proliferum. J. Am. Chem. Soc..

[28] Kajula M (2016). Bridged epipolythiodiketopiperazines from penicillium raciborskii, an endophytic fungus of rhododendron tomentosum harmaja. J. Nat. Prod..

[29] Witt D (2008). Recent developments in disulfide bond formation. Synthesis.

[30] Musiejuka M, Witt D (2015). Recent developments in the synthesis of unsymmetrical disulfanes (disulfides). Org. Prep. Proced. Int..

[31] Feng M, Tang B, Liang S, Jiang X (2016). Sulfur containing scaffolds in drugs: synthesis and application in medicinal chemistry. Curr. Top. Med. Chem..

[32] Swan JM (1957). Thiols, disulphides and thiosulphates: some new reactions and possibilities in peptide and protein chemistry. Nature.

[33] Bao M, Shimizu M (2003). N-Trifluoroacetyl arenesulfenamides, effective precursors for synthesis of unsymmetrical disulfides and sulfenamides. Tetrahedron.

[34] Sivaramakrishnan S, Keerthi K, Gates KS (2005). A chemical model for redox regulation of protein tyrosine phosphatase 1B (PTP1B) activity. J. Am. Chem. Soc..

[35] Hunter R, Caira M, Stellenboom N (2006). Inexpensive, one-pot synthesis of unsymmetrical disulfides using 1-chlorobenzotriazole. J. Org. Chem..

[36] Antoniow S, Witt D (2007). A novel and eficient synthesis of unsymmetrical disulfides. Synthesis.

[37] Szymelfejnik M, Demkowicz S, Rachon J, Witt D (2007). Functionalization of cysteine derivatives by unsymmetrical disulfide bond formation. Synthesis.

[38] Taniguchi N (2017). Unsymmetrical disulfide and sulfenamide synthesis via reactions of thiosulfonates with thiols or amines. Tetrahedron.

[39] Vandavasi JK, Hu WP, Chen CY, Wang JJ (2011). Efficient synthesis of unsymmetrical disulfides. Tetrahedron.

[40] Arisawa M, Yamaguchi M (2003). Rhodium-catalyzed disulfide exchange reaction. J. Am. Chem. Soc..

[41] Liu H, Jiang X (2013). Transfer of sulfur: from simple to diverse. Chem. Asian J..

[42] Qiao Z (2013). Efficient access to 1, 4-benzothiazine: palladium-catalyzed double C-S bond formation using Na_2_S_2_O_3_ as sulfurating reagent. Org. Lett..

[43] Wei J, Li Y, Jiang X (2016). Aqueous compatible protocol to both alkyl and aryl thioamide synthesis. Org. Lett..

[44] Qiao Z, Jiang X (2017). Recent development in sulfur-carbon bond Formation reaction involving thiosulfate. Org. Biomol. Chem..

[45] Wang M, Wei J, Fan Q, Jiang X (2017). Cu(II)-catalyzed sulfide construction: both aryl groups utilization of intermolecular and intramolecular diaryliodonium salt. Chem. Commun..

[46] Tan W, Wei J, Jiang X (2017). Thiocarbonyl surrogate via combination of sulfur and chloroform for thiocarbamide and oxazolidinethione constructions. Org. Lett..

[47] Wang M, Chen S, Jiang X (2017). Construction of functionalized annulated sulfone via SO_2_/I exchange of cyclic diaryliodonium salts. Org. Lett..

[48] Li Y, Wang M, Jiang X (2017). Controllable sulfoxidationand sulfenylation with organic thiosulfate salts via dual electron- and energy-transfer photocatalysis. ACS Catalysis.

[49] Xiao X, Feng M, Jiang X (2015). Transition-metal-free persulfuration to construct unsymmetrical disulfides and mechanistic study of sulfur redox process. Chem. Commun..

[50] Bailey TS, Zakharov LN, Pluth MD (2014). Understanding hydrogen sulfide storage: probing conditions for sulfide release from hydrodisulfides. J. Am. Chem. Soc..

[51] Chauvin JPR, Griesser M, Pratt DA (2017). Hydropersulfides: H-atom transfer agents par excellence. J. Am. Chem. Soc..

[52] Xiao X, Feng M, Jiang X (2016). New design of disulfurating reagent: facile and straightforward pathway to unsymmetrical disulfanes via Cu-catalyzed oxidative cross coupling. Angew. Chem. Int. Ed..

[53] Pearson RG (1963). Hard and soft acids and bases. J. Am. Chem. Soc..

