# Supplementary material for: Polysulfurating reagent design for unsymmetrical polysulfide construction
Source: Nat Commun. 2018 Jun 6;9:2191. doi: 10.1038/s41467-018-04306-5 (PMC5989225; doi:10.1038/s41467-018-04306-5)
Supplement: Supplementary file 1 — Supplementary Information [file 41467_2018_4306_MOESM1_ESM.pdf]

Supporting Information

Polysulfurating Reagents Design for Unsymmetrical Polysulfides Construction

Xiao et al.

Supplementary Methods

General information

All experiments were conducted under air atmosphere unless otherwise noted. Anhydrous CH_2ClCH_2Cl was prepared by first distillation over P_2O_5 and then from CaH_2. Toluene and THF were prepared by distillation over sodium-benzophenone ketyl prior to use. Other solvents were undried solvents. ^{1}H and ^{13}C NMR spectra were collected on 400 MHz or 500 MHz NMR spectrometers (Bruker AVANCE) using $CDCl_3$ or DMSO-d_6. Chemical shifts are reported in parts per million (ppm). Chemical shifts for protons are reported in parts per million downfield and are referenced to residual protium in the NMR solvent ($CHCl_3$ = δ 7.26, DMSO = δ 2.50). Chemical shifts for carbon are reported in parts per million downfield and are referenced to the carbon resonances of the solvent ($CDCl_3$ = δ 77.0, DMSO = δ 39.5). Data are represented as follows: chemical shift, multiplicity (br = broad, s = singlet, d = doublet, t = triplet, q = quartet, m = multiplet), coupling constants in Hertz (Hz), integration. Unless otherwise noted, commercially available reagents purchased from Adamas-beta, TCI, or Energy Chemical and were used as received.

Mass spectroscopy:

Mass spectra were in general recorded on a Shimadzu GCMS-QP2010 Ultra and a HP 5989A mass selective detector.

Chromatography:

Column chromatography was performed with silica gel (200-300 mesh ASTM).

IR:

TENSOR (27) Series FT-IR Spectrometers.

Optimization of disulfurating reagents and gram scale reaction for disulfurating reagents synthese

Supplementary Table 1. Optimization of disulfurating reagents.

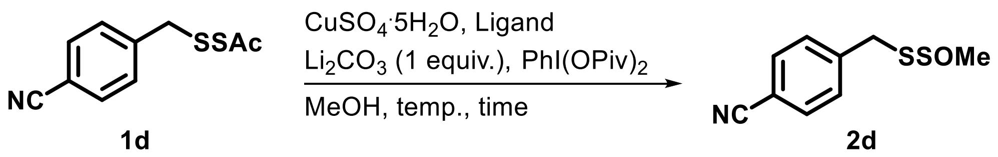

Entry	$CuSO_4$ (mol%)	Ligand (mol%)	$PhI(OPiv)_2$ (equiv.)	Temp. (°C)	Time (h)	Yield (%[b])
1[c]	10	bpy (10)	2.5	25	11	31
2[d]	10	bpy (10)	2.5	25	11	ND
3	10	bpy/ phen (10)	2.5	25	11	50/ 53
4	10	L1 (10)	2.5	25	11	77
5	10	L2/ L3/ L4 (10)	2.5	25	11	70/ 63/ 68
6	10	L1 (10)	2.5	20	13	86
7	5	L1 (10)	2.5	20	13	86
8	2.5	L1 (10)	2.5	20	13	79
9	5	L1 (5)	2.5	20	13	76
10	***5***	***L1 (10)***	***2.2***	***20***	***13***	***88***
11	5	L1 (10)	1.9	20	13	65

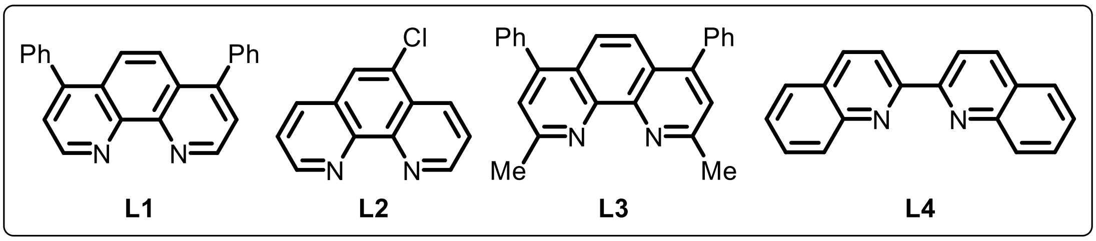

[a]Conditions: **1d** (0.2 mmol, 1 equiv.), $CuSO_4·5H_2O$, Ligand, Li_2CO_3 and $PhI(OPiv)_2$ were added to MeOH (2 mL) at 20 °C for 13 h. [b]Isolated yeild. [c]$PhI(OAc)_2$ was instead of $PhI(OPiv)_2$. [d]$PhI(OTFA)_2$ was instead of $PhI(OPiv)_2$.

To a Schlenk tube were added $4\text{-}CNC_6H_4CH_2SSAc$ **(1d)** (0.2 mmol, 1 equivalent, 44.7 mg), $CuSO_4·5H_2O$, Ligand, Li_2CO_3 (0.2 mmol, 1 equivalent, 14.8 mg), oxidant, and undistilled MeOH (2 mL), the mixture was stirred at 20 °C before it was concentrated under vacuum. Purification by column chromatography afforded the desired product.

Supplementary Table 2. Optimization of gram scale reaction for disulfurating reagents synthesis.

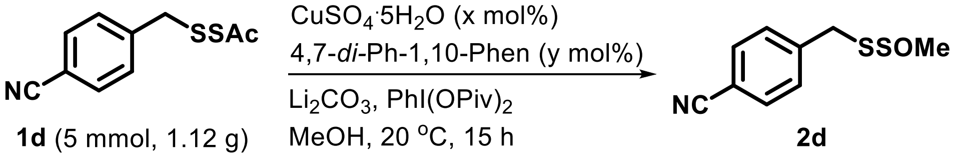

Entry	$CuSO_4$ /mol%	Ligand / mol%	$PhI(OPiv)_2$ / equiv.	MeOH / mL	time / h	yeild / %[b]
1	5	10	2.2	50	13	86
2	5	6	2.2	50	13	84
3	5	6	1.7	50	13	56
4	2.5	6	2.2	50	13	77
5	2.5	6	2.2	35	13	81
6	1	2	2.2	35	13	76
7	1	2	2.2	20	14	88
8	0.5	1	2.2	20	14	83
9	0.5	1	2.2	10	15	86
10	***0.25***	***0.5***	***2.2***	***10***	***15***	***87***
11[c]	0.25	0.5	2.2	10	15	87
12[c]	0.125	0.25	2.2	10	15	80

[a]Conditions: **1d** (5 mmol, 1 equiv.), $CuSO_4·5H_2O$, **L1**, Li_2CO_3 (5 mmol, 1 equiv.) and $PhI(OPiv)_2$ (11 mmol, 2.2 equiv.) were added to MeOH at 20 °C. [b]Isolated yeild. [c]**1d** (10 mmol, 1 equiv.).

To a Schlenk tube were added $4\text{-}CNC_6H_4CH_2SSAc$ (**1e)** (5 mmol, 1 equivalent, 1.12 g), $CuSO_4·5H_2O$, **L1**, Li_2CO_3 (5 mmol, 1 equivalent, 370 mg), $PhI(OPiv)_2$, and undistilled MeOH (2 mL), the mixture was stirred at 20 °C for 13-15 hours. The mixture was quenched by saturated $NaHCO_3$ and extracted by DCM before the organic phase was concentrated under vacuum without adding silica gel. Purification by column chromatography afforded the desired product.

General procedure and spectra data of disulfurating reagents

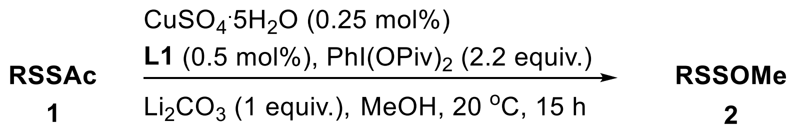

General procedure: To a Schlenk tube were added RSSAc **1**[1](5 mmol, 1 equivalent), $CuSO_4{\cdot}5H_2O$ (0.0125 mmol, 0.25 mol%, 3.2 mg), **L1** (0.025 mmol, 0.5 mol%, 8.1 mg), Li_2CO_3 (5 mmol, 1 equivalent, 370 mg), $PhI(OPiv)_2$[2-3] (11 mmol, 2.2 equivalents, 4.47 g), and undistilled MeOH (10 mL), the mixture was stirred at 20 ^{o}C for 15 hours. The mixture was quenched by saturated $NaHCO_3$ and extracted by DCM before the organic phase was concentrated under vacuum without adding silica gel. Purification by column chromatography afforded the desired product.

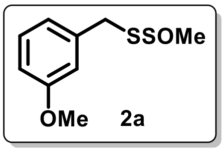

The reaction of 3-$MeOC_6H_4CH_2SSAc$ (5 mmol, 1 equivalent, 1.14 g), $CuSO_4.5H_2O$ (0.0125 mmol, 0.25 mol%, 3.2 mg), **L1** (0.025 mmol, 0.5 mol%, 8.1 mg), Li_2CO_3 (5 mmol, 1 equivalent, 370 mg), and $PhI(OPiv)_2$ (11 mmol, 2.2 equivalents, 4.47 g) in MeOH (10 mL) at 20 ^{o}C for 15 hours afforded compound **2a** in 54% yield (590 mg) as a colorless oil according to the general procedure. **^{1}H NMR** (400 MHz, $CDCl_3$) δ 7.25-7.18 (m, 1H), 6.94-6.84 (m, 2H), 6.82 (dd, J = 8.2, 2.0 Hz, 1H), 4.14 (s, 2H), 3.80 (s, 3H), 3.58 (s, 3H). **^{13}C NMR** (100 MHz, $CDCl_3$) δ 159.8, 138.2, 129.6, 121.6, 114.7, 113.2, 62.7, 62.6, 55.2, 55.2, 46.7. **IR** (film) 2973, 2913, 1599, 1489, 1435, 1263, 1152, 1051, 982, 872, 781, 672. **HRMS** (EI) Calcd for $C_9H_{12}O_2S_2$ 216.0279, Found 216.0283.

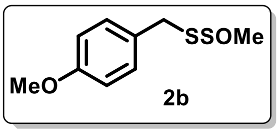

The reaction of 4-$MeOC_6H_4CH_2SSAc$ (5 mmol, 1 equivalent, 1.14 g), $CuSO_4.5H_2O$ (0.0125 mmol, 0.25 mol%, 3.2 mg), **L1** (0.025 mmol, 0.5 mol%, 8.1 mg), Li_2CO_3 (5 mmol, 1 equivalent, 370 mg), and $PhI(OPiv)_2$ (11 mmol, 2.2 equivalentss, 4.47 g) in MeOH (10 mL) at 20 ^{o}C for 15 hours afforded compound **2b** in 58% yield (630 mg) as a colorless oil according to the general procedure. **^{1}H NMR** (400 MHz, $CDCl_3$) δ 7.27 (d, J = 8.7 Hz, 2H), 6.90 (d, J = 8.7 Hz, 2H), 4.16 (s, 2H), 3.83 (s, 3H), 3.61 (s, 3H). **^{13}C NMR** (100 MHz, $CDCl_3$) δ 159.1, 130.4, 128.7, 114.1, 62.6, 55.3, 45.9. **IR** (film)

2964, 2922, 2831, 1610, 1510, 1452, 1245, 1176, 1034, 982, 827, 740, 672. **HRMS** (EI) Calcd for $C_9H_{12}O_2S_2$ 216.0279, Found 216.0277.

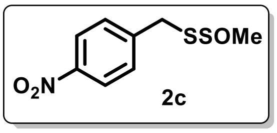

The reaction of 4-$NO_2C_6H_4CH_2SSAc$ (5 mmol, 1 equivalent, 1.22 g), $CuSO_4.5H_2O$ (0.0125 mmol, 0.25 mol%, 3.2 mg), **L1** (0.025 mmol, 0.5 mol%, 8.1 mg), Li_2CO_3 (5 mmol, 1 equivalent, 370 mg), and $PhI(OPiv)_2$ (11 mmol, 2.2 equivalents, 4.47 g) in MeOH (10 mL) at 20 ^oC for 15 hours afforded compound **2c** in 74% yield (856 mg) as a white solid according to the general procedure. **^{1}H NMR** (400 MHz, $CDCl_3$) δ8.20 (d, J = 8.7 Hz, 2H), 7.48 (d, J = 8.7 Hz, 2H), 4.19 (s, 2H), 3.57 (s, 3 H). **^{13}C NMR** (100 MHz, $CDCl_3$) δ 147.2, 144.4, 130.1, 123.8, 62.7, 44.9. **IR** (film) 2972, 1601, 1517, 1343, 1073, 980, 856, 800,754, 675. **HRMS** (EI) Calcd for $C_8H_9NO_3S_2$ 231.0024, Found 231.0022.

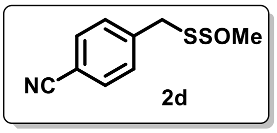

The reaction of 4-$CNC_6H_4CH_2SSAc$ (10 mmol, 1 equivalent, 2.24 g), $CuSO_4.5H_2O$ (0.025 mmol, 0.25 mol%, 6.4 mg), **L1** (0.05 mmol, 0.5 mol%, 16.2 mg), Li_2CO_3 (10 mmol, 1 equivalent, 740 mg), and $PhI(OPiv)_2$ (22 mmol, 2.2 equivalents, 8.94 g) in MeOH (10 mL) at 20 ^oC for 15 hours afforded compound **2d** in 87% yield (1.87 mg) as a colorless oil according to the general procedure. **^{1}H NMR** (400 MHz, $CDCl_3$) δ 7.62 (d, J = 8.3 Hz, 2H), 7.42 (d, J = 8.4 Hz, 2H), 4.15 (s, 2H), 3.56 (s, 3H). **^{13}C NMR** (100 MHz, $CDCl_3$) δ 142.3, 132.4, 130.0, 118.6, 111.4, 62.6, 45.4. **IR** (film) 2927, 2816, 2228, 1728, 1606, 1504, 1417, 1284, 1105, 981, 843, 740, 675. **HRMS** (EI) Calcd for $C_9H_9NOS_2$ 211.0126, Found 211.0132.

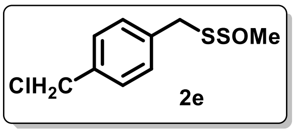

The reaction of 4-$ClCH_2C_6H_4CH_2SSAc$ (5 mmol, 1 equivalent, 1.24 g), $CuSO_4.5H_2O$ (0.0125 mmol, 0.25 mol%, 3.2 mg), **L1** (0.025 mmol, 0.5 mol%, 8.1 mg), Li_2CO_3 (5 mmol, 1 equivalent, 370 mg), and $PhI(OPiv)_2$ (11 mmol, 2.2 equivalents, 4.47 g) in MeOH (10 mL) at 20 ^oC for 15 hours afforded compound **2e** in 68% yield (800 mg) as a colorless oil according to the general procedure. **^{1}H NMR** (400 MHz, $CDCl_3$) δ 7.37 (d, J = 8.1 Hz, 2H), 7.32 (d, J = 8.1 Hz, 2H), 4.58 (s, 2H), 4.16 (s, 2H), 3.58 (s, 3H). **^{13}C NMR** (100 MHz, $CDCl_3$) δ137.0, 136.7, 129.6, 128.9, 62.6, 45.9, 45.8. **IR**

(film) 2970, 1512, 1418, 1264, 1072, 982, 829, 765, 672. **HRMS** (EI) Calcd for $C_9H_{11}ClOS_2$ 233.9940, Found 233.9943.

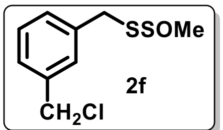

2f

The reaction of 3-$ClCH_2C_6H_4CH_2SSAc$ (5 mmol, 1 equivalent, 1.24 g), $CuSO_4.5H_2O$ (0.0125 mmol, 0.25 mol%, 3.2 mg), **L1** (0.025 mmol, 0.5 mol%, 8.1 mg), Li_2CO_3 (5 mmol, 1 equivalent, 370 mg), and $PhI(OPiv)_2$ (11 mmol, 2.2 equivalents, 4.47 g) in MeOH (10 mL) at 20 ^oC for 15 hours afforded compound **2f** in 68% yield (800 mg) as a colorless oil according to the general procedure. **^{1}H NMR** (400 MHz, $CDCl_3$) δ 7.38-7.28 (m, 4H), 4.60 (s, 2H), 4.18 (s, 2H), 3.59 (s, 3H). **^{13}C NMR** (100 MHz, $CDCl_3$) δ 137.9, 137.4, 129.4, 129.3, 129.0, 127.7, 62.7, 46.1, 45.9. **IR** (film) 2972, 21814,1607, 1443, 1271, 1075, 982, 900, 797, 706, 673. **HRMS** (EI) Calcd for $C_9H_{11}ClOS_2$ 233.9940, Found 233.9935.

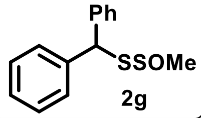

2g

The reaction of (Ph)2CHSSAc (5 mmol, 1 equivalent, 1.38 g), $CuSO_4.5H_2O$ (0.0125 mmol, 0.25 mol%, 3.2 mg), **L1** (0.025 mmol, 0.5 mol%, 8.1 mg), Li_2CO_3 (5 mmol, 1 equivalent, 370 mg), and $PhI(OPiv)_2$ (11 mmol, 2.2 equivalents, 4.47 g) in MeOH (10 mL) at 20 ^oC for 15 hours afforded compound **2g** in 76% yield (997 mg) as a colorless oil according to the general procedure. **^{1}H NMR** (400 MHz, $CDCl_3$) δ 7.46 - 7.44 (m, 4H), 7.40-7.36 (m, 4H), 7.33 - 7.28 (m, 2H), 5.55 (s, 1H), 3.53 (s, 3H).. **^{13}C NMR** (100 MHz, $CDCl_3$) δ 139.9, 128.9, 128.6, 127.6, 63.1, 62.9. **IR** (film), 3060, 2926, 2814, 1579, 1493, 1448, 1335, 1160, 983, 745, 697, 674, 625. **HRMS** (EI) Calcd for $C_{14}H_{14}OS_2$ 262.0486, Found 262.0488.

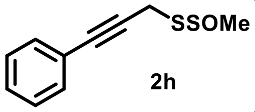

2h

The reaction of $C_6H_5CCCH_2SSAc$ (5 mmol, 1 equivalent, 1.11 g), $CuSO_4.5H_2O$ (0.0125 mmol, 0.25 mol%, 3.2 mg), **L1** (0.025 mmol, 0.5 mol%, 8.1 mg), Li_2CO_3 (5 mmol, 1 equivalent, 370 mg), and $PhI(OPiv)_2$ (11 mmol, 2.2 equivalents, 4.47 g) in MeOH (10 mL) at 20 ^oC for 15 hours afforded compound **2h** in 50% yield (525 mg) as a colorless oil according to the general procedure. **^{1}H NMR** (400 MHz, $CDCl_3$) δ 7.46-7.44 (m, 2H), 7.34-7.29 (m, 3H), 3.94 (s, 2H), 3.69 (s, 3H). **^{13}C NMR** (100 MHz, $CDCl_3$) δ 131.7, 128.4, 128.3, 122.7, 85.5, 84.0, 63.1, 31.2. **IR** (film) 2975, 2902,

2815, 2217, 1597, 1490, 1411, 1212, 1067, 982, 755, 676. **HRMS** (EI) Calcd for $C_{10}H_{10}OS_2$ 210.0173, Found 210.0170.

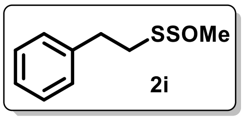

The reaction of $C_6H_5CH_2CH_2SSAc$ (5 mmol, 1 equivalent, 1.06 g), $CuSO_4.5H_2O$ (0.0125 mmol, 0.25 mol%, 3.2 mg), **L1** (0.025 mmol, 0.5 mol%, 8.1 mg), Li_2CO_3 (5 mmol, 1 equivalent, 370 mg), and $PhI(OPiv)_2$ (11 mmol, 2.2 equivalents, 4.47 g) in MeOH (10 mL) at 20 ^{o}C for 15 hours afforded compound **2i** in 72% yield (726 mg) as a colorless oil according to the general procedure. **^{1}H NMR** (400 MHz, $CDCl_3$) δ 7.26-7.22 (m, 2H), 7.18-7.14 (m, 3H), 3.55 (s, 3H), 3.15-3.06 (m, 2H), 3.01-2.98 (m, 2H). **^{13}C NMR** (100 MHz, $CDCl_3$) δ 139.7, 128.6, 128.5, 126.4, 62.5, 42.6, 35.8. **IR** (film) 2968, 2925, 2813, 1603, 1496, 1453, 1261, 1050, 984, 748, 699, 672. **HRMS** (EI) Calcd for $C_9H_{12}OS_2$ 200.0330, Found 200.0329.

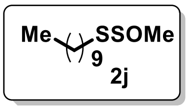

The reaction of $Me(CH_2)_9SSAc$ (5 mmol, 1 equivalent, 1.25 g), $CuSO_4.5H_2O$ (0.0125 mmol, 0.25 mol%, 3.2 mg), **L1** (0.025 mmol, 0.5 mol%, 8.1 mg), Li_2CO_3 (5 mmol, 1 equivalent, 370 mg), and $PhI(OPiv)_2$ (11 mmol, 2.2 equivalents, 4.47 g) in MeOH (10 mL) at 20 ^{o}C for 15 hours afforded compound **2j** in 63% yield (745 mg) as a colorless oil according to the general procedure. **^{1}H NMR** (400 MHz, $CDCl_3$) δ 3.61 (s, 3H), 3.28-2.63 (m, 2H), 1.89-1.61 (m, 2 H), 1.40-1.35 (m, 2H), 1.31-1.28 (m, 12H), 0.88 (t, J = 6.8 Hz, 3H). **^{13}C NMR** (100 MHz, $CDCl_3$) δ 62.4, 41.8, 31.9, 29.5, 29.5, 29.4, 29.3, 29.2, 28.4, 22.7, 14.1. **IR** (film), 2924, 1457, 1409, 1251, 1052, 989, 894, 673. **HRMS** (EI) Calcd for $C_{11}H_{24}OS_2$ 236.1269, Found 236.1274.

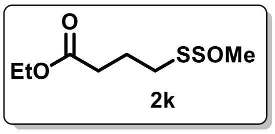

The reaction of $EtO_2C(CH_2)_3SSAc$ (5 mmol, 1 equivalent, 1.11 g), $CuSO_4.5H_2O$ (0.0125 mmol, 0.25 mol%, 3.2 mg), **L1** (0.025 mmol, 0.5 mol%, 8.1 mg), Li_2CO_3 (5 mmol, 1 equivalent, 370 mg), and $PhI(OPiv)_2$ (11 mmol, 2.2 equivalents, 4.47 g) in MeOH (10 mL) at 20 ^{o}C for 15 hours afforded compound **2k** in 75% yield (789 mg) as a colorless oil according to the general procedure. **^{1}H NMR** (400 MHz, $CDCl_3$) δ 4.13 (q, J = 7.1 Hz, 2H), 3.60 (s, 3H), 2.96 (t, J = 7.2 Hz, 2H), 2.43 (t, J = 7.2 Hz, 2H), 2.08 (p, J = 7.2 Hz, 2H), 1.25 (t, J = 7.1 Hz, 3H). **^{13}C NMR** (100 MHz, $CDCl_3$) δ

172.8, 62.5, 60.4, 40.6, 32.5, 24.5, 14.2. **IR** (film) 2981, 1731, 1374, 1200, 1133, 1041, 984, 801, 673. **HRMS** (EI) Calcd for $C_7H_{14}O_3S_2$ 210.0834, Found 210.0388.

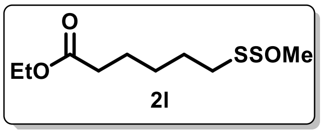

The reaction of $EtO_2C(CH_2)_5SSAc$ (5 mmol, 1 equivalent, 1.25 g), $CuSO_4.5H_2O$ (0.0125 mmol, 0.25 mol%, 3.2 mg), **L1** (0.025 mmol, 0.5 mol%, 8.1 mg), Li_2CO_3 (5 mmol, 1 equivalent, 370 mg), and $PhI(OPiv)_2$ (11 mmol, 2.2 equivalents, 4.47 g) in MeOH (10 mL) at 20 ^oC for 15 hours afforded compound **2l** in 36% yield (450 mg) as a colorless oil according to the general procedure. **^{1}H NMR** (400 MHz, $CDCl_3$) δ 7.26-7.22 (m, 2H), 7.18-7.14 (m, 3H), 3.55 (s, 3H), 3.15-3.06 (m, 2H), 3.01-2.98 (m, 2H). **^{13}C NMR** (100 MHz, $CDCl_3$) δ 173.56, 62.4, 60.3, 41.4, 34.1, 29.0, 27.8, 24.5, 14.2. **IR** (film) 2967, 2900, 1733, 1376, 1253, 1186, 1075, 1056, 986, 750, 673. **HRMS** (EI) Calcd for $C_9H_{18}O_3S_2$ 238.0697, Found 238.0699.

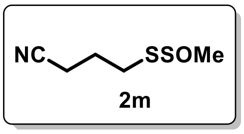

The reaction of $NC(CH_2)_3SSAc$ (5 mmol, 1 equivalent, 853 mg), $CuSO_4{\cdot}5H_2O$ (0.0125 mmol, 0.25 mol%, 3.2 mg), **L1** (0.025 mmol, 0.5 mol%, 8.1 mg), Li_2CO_3 (5 mmol, 1 equivalent, 370 mg), and $PhI(OPiv)_2$ (11 mmol, 2.2 equivalents, 4.47 g) in MeOH (10 mL) at 20 ^oC for 15 hours afforded compound **2m** in 75% yield (612 mg) as a colorless oil according to the general procedure. **^{1}H NMR** (400 MHz, $CDCl_3$) δ 3.62 (s, 1H), 3.02 (t, J = 6.9 Hz, 2 H), 2.51 (t, J = 7.0 Hz, 2H), 2.15 (tt, J = 7.0 Hz, J = 6.9 Hz, 2H). **^{13}C NMR** (100 MHz, $CDCl_3$) δ 118.9, 62.6, 39.1, 24.9, 15.7. **IR** (film) 2963, 2928, 2816, 2247, 1731, 1445, 1424, 981, 674. **HRMS** (EI) Calcd for $C_5H_9NOS_2$ 163.0126, Found 163.0125.

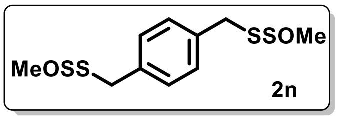

The reaction of $AcSSCH_2C_6H_4CH_2SSAc$ (2.5 mmol, 1 equivalent, 796 mg), $CuSO_4{\cdot}5H_2O$ (0.0125 mmol, 0.5 mol%, 3.2 mg), **L1** (0.05 mmol, 1 mol%, 8.1 mg), Li_2CO_3 (5 mmol, 2 equivalents, 370 mg), and $PhI(OPiv)_2$ (11 mmol, 4.4 equivalents, 4.47 g) in MeOH: acetone (9: 1) (10 mL) at 20 ^oC for 15 hours afforded compound **2n** in 38% yield (282 mg) as a white solid according to the general procedure. **^{1}H NMR** (400 MHz, $CDCl_3$) δ 7.30 (s, 4H), 4.15 (s, 4H), 3.57 (s, 6H). **^{13}C NMR** (100 MHz,

$CDCl_3$) δ 136.1, 129.6, 62.7, 46.0. **IR** (film) 2982, 1411, 1231, 1053, 979, 831, 669. **HRMS** (EI) Calcd for $C_{10}H_{14}O_2S_4$ 293.9872, Found 293.9877.

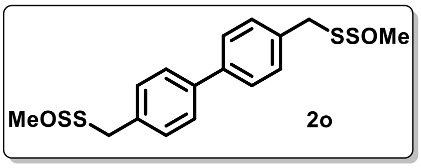

The reaction of $AcSSCH_2C_6H_4C_6H_4CH_2SSAc$ (2.5 mmol, 1 equivalent, 987 mg), $CuSO_4.5H_2O$ (0.0125 mmol, 0.5 mol%, 3.2 mg), **L1** (0.05 mmol, 1 mol%, 8.1 mg), Li_2CO_3 (5 mmol, 2 equivalents, 370 mg), and $PhI(OPiv)_2$ (11 mmol, 4.4 equivalents, 4.47 g) in MeOH : acetone (9: 1) (10 mL) at 20 ^oC for 15 hours afforded compound **2o** in 42% yield (388 mg) as a white solid according to the general procedure. **^{1}H NMR** (400 MHz, $CDCl_3$) δ 7.57 (d, J = 8.2 Hz, 4H), 7.40 (d, J = 8.1 Hz, 4H), 4.21 (s, 4H), 3.60 (s, 6H). **^{13}C NMR** (100 MHz, $CDCl_3$) δ 139.9, 135.9, 129.7, 127.3, 62.7, 46.1. **IR** (film) 2987, 2971, 2810, 1494, 1405, 1232, 1054, 981, 823, 725, 668. **HRMS** (EI) Calcd for $C_{16}H_{18}O_2S_4$ 370.0190, Found 370.0188.

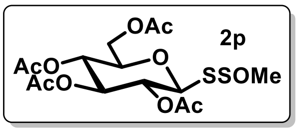

The reaction of RSSAc (5 mmol, 1 equivalent, 2.2 g), $CuSO_4.5H_2O$ (0.0125 mmol, 0.25 mol%, 3.2 mg), **L1** (0.025 mmol, 0.5 mol%, 8.1 mg), Li_2CO_3 (5 mmol, 1 equivalent, 370 mg), and $PhI(OPiv)_2$ (11 mmol, 2.2 equivalents, 4.47 g) in MeOH (10 mL) at 20 ^oC for 15 hours afforded compound **2p** in 88% yield (1.88 g) as a white solid according to the general procedure. **^{1}H NMR** (400 MHz, $CDCl_3$) δ 5.33 - 5.19 (m, 2H), 5.15-5.11 (m, 1H), 4.67-4.65 (m, 1H), 4.26 (d of ABq, J = 12.5, 4.7 Hz, 1H), 4.16 (d of ABq, J = 12.5, 2.3 Hz, 1H), 3.80 (ddd, J = 10.0, 4.7, 2.3 Hz, 1H), 3.64 (s, 3H), 2.05 (s, 3H), 2.03 (s, 3H), 2.02 (s, 3H), 2.01 (s, 3H). **^{13}C NMR** (100 MHz, $CDCl_3$) δ 170.5, 170.2, 169.3, 169.0, 76.5, 73.8, 69.9, 67.9, 63.7, 61.8, 20.6. **IR** (film) 2946, 1746, 1366, 1226, 1208, 1031, 973, 756. 695. **HRMS** (ESI) Calcd for $C_{15}H_{22}O_{10}S_2$ (M+Na$^+$) 449.0547, Found 449.0559.

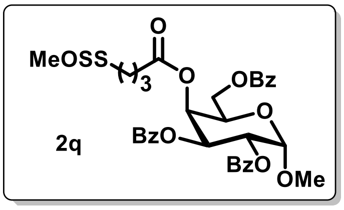

The reaction of RSSAc (5 mmol, 1 equivalent, 3.42 g), $CuSO_4.5H_2O$ (0.0125 mmol, 0.25 mol%, 3.2 mg), **L1** (0.025 mmol, 0.5 mol%, 8.1 mg), Li_2CO_3 (5 mmol, 1 equivalent, 370 mg), and $PhI(OPiv)_2$ (11 mmol, 2.2 equivalents, 4.47 g) in MeOH (10 mL) at 20 ^oC for 15 hours afforded compound **2q** in

64% yield (2.13 g) as a white solid according to the general procedure. **^{1}H NMR** (400 MHz, $CDCl_3$) δ 8.03 (d, J = 7.3 Hz, 2H), 8.00 (d, J = 7.3 Hz, 2H), 7.89 (d, J = 7.3 Hz, 2H), 7.59-7.55 (m, 1H), 7.54-7.48 (m, 2H), 7.47-7.43 (m, 2H), 7.40-7.35 (m, 4H), 5.92 - 5.78 (m, 2H), 5.60 (d of ABq, J = 10.6, 3.6 Hz, 1H), 5.24 (d, J = 3.6 Hz, 1H), 4.55-4.5o (m, 2H), 4.39-4.33 (m, 1H), 3.57 (s, 3H), 3.47 (s, 3H), 2.85 (t, J = 7.1 Hz, 2H), 2.66-2.52 (m, 2H), 2.05 (p, J = 7.1 Hz, 2H). **^{13}C NMR** (100 MHz, $CDCl_3$) δ 171.8, 165.9, 165.4, 133.3(3), 133.2($_6$), 133.2(5), 129.8, 129.6, 129.5, 129.4, 129.2, 128.4, 128.4, 128.4, 97.5, 68.9, 68.5, 68.3, 66.4, 62.4, 62.2, 55.6, 40.1, 32.0, 24.4. **IR** (film) 2974, 2902, 2817, 1721, 1602, 1451, 1315, 1064, 984, 708, 674. **HRMS** (ESI) Calcd for $C_{33}H_{34}O_{11}S_2$ (M+Na$^+$) 693.1435, Found 693.1449.

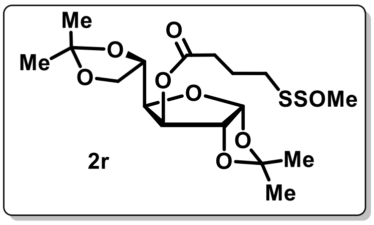

The reaction of RSSAc (5 mmol, 1 equivalent, 2.14 g), $CuSO_4.5H_2O$ (0.0125 mmol, 0.25 mol%, 3.2 mg), **L1** (0.025 mmol, 0.5 mol%, 8.1 mg), Li_2CO_3 (5 mmol, 1 equivalent, 370 mg), and $PhI(OPiv)_2$ (11 mmol, 2.2 equivalents, 4.47 g) in MeOH (10 mL) at 20 ^oC for 15 hours afforded compound **2r** in 67% yield (1.42 g) as a colorless oil according to the general procedure. **^{1}H NMR** (400 MHz, $CDCl_3$) δ 5.86 (d, J = 3.6 Hz, 1H), 5.27 (s, 1H), 4.47 (d, J = 3.6 Hz, 1H), 4.22 - 4.16 (m, 2H), 4.09 - 4.06 m, 1H), 4.03 - 3.95 (m, 1H), 3.59 (s, 3H), 2.95 (t, J = 7.1 Hz, 2H), 2.56 - 2.40 (m, 2H), 2.10 (p, J = 7.1 Hz, 2H), 1.50 (s, 3H), 1.39 (s, 3H), 1.30 (s, 3H) , 1.29 (s, 3H). **^{13}C NMR** (100 MHz, $CDCl_3$) δ 171.4, 112.2, 109.3, 105.0, 83.3, 79.8, 76.0, 72.4, 67.3, 62.4, 40.0, 32.3, 26.8, 26.7, 26.1, 25.2, 24.4. **IR** (film) 2986, 2903, 1744, 1376, 1215, 1161, 1072, 1023, 985, 845, 733, 675. **HRMS** (EI) Calcd for $C_{17}H_{28}O_8S_2$ 424.1226, Found 424.1229.

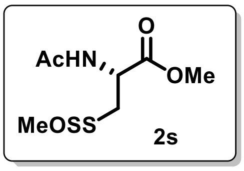

The reaction of RSSAc (5 mmol, 1 equivalent, 1.27 g), $CuSO_4.5H_2O$ (0.0125 mmol, 0.25 mol%, 3.2 mg), **L1** (0.025 mmol, 0.5 mol%, 8.1 mg), Li_2CO_3 (5 mmol, 1 equivalent, 370 mg), and $PhI(OPiv)_2$ (11 mmol, 2.2 equivalents, 4.47 g) in MeOH (10 mL) at 20 ^oC for 15 hours afforded compound **2s** in 67% yield (806 mg) as a white solid according to the general procedure. **^{1}H NMR** (400 MHz, $CDCl_3$) δ 6.73 (brs, 1H), 5.07-4.95 (m, 1H), 3.76 (s, 3H), 3.63 (s, 3H), 3.47 (d of ABq, J = 14.2, 4.4

Hz, 1H), 3.35 (d of ABq, J = 14.2, 3.1 Hz, 1H), 2.00 (s, 3H) .^{13}**C NMR** (100 MHz, $CDCl_3$) δ 170.4, 169.9, 62.6, 52.7, 51.4, 44.6, 23.0. **IR** (film) 3287, 2998, 2971, 2882, 1722, 1639, 1543, 1377, 1243, 1066, 980, 797, 757, 673. **HRMS** (ESI) Calcd for $C_7H_{13}NO_4S_2$ (M+Na$^+$) 262.0178, Found 262.0178.

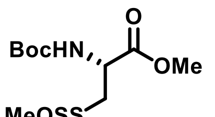

2t

The reaction of RSSAc (5 mmol, 1 equivalent, 1.56 g), $CuSO_4.5H_2O$ (0.0125 mmol, 0.25 mol%, 3.2 mg), **L1** (0.025 mmol, 0.5 mol%, 8.1 mg), Li_2CO_3 (5 mmol, 1 equivalent, 370 mg), and $PhI(OPiv)_2$ (11 mmol, 2.2 equivalents, 4.47 g) in MeOH (10 mL) at 20 ^{o}C for 15 hours afforded compound **2t** in 67% yield (1.22 g) as a colorless oil according to the general procedure. ^{1}**H NMR** (400 MHz, $CDCl_3$) δ 5.53 (s, 1H), 4.80 - 4.49 (m, 1H), 3.76 (s, 3H), 3.61 (s, 3H), 3.41 (d, J = 4.0 Hz, 2H), 1.43 (s, 9H). ^{13}**C NMR** (100 MHz, $CDCl_3$) δ 170.8, 155.1, 80.1, 62.5, 52.9, 52.6, 44.8, 28.2. **IR** (film) 3374, 2974, 2904, 1747, 1715, 1506, 1367, 1255, 1162, 1052, 983, 866, 760, 679. **HRMS** (EI) Calcd for $C_{10}H_{19}NO_5S_2$ 297.0705, Found 297.0707.

Optimization of polysulfuration

Supplementary Table 3. Optimization of disulfuration with acetylacetone.

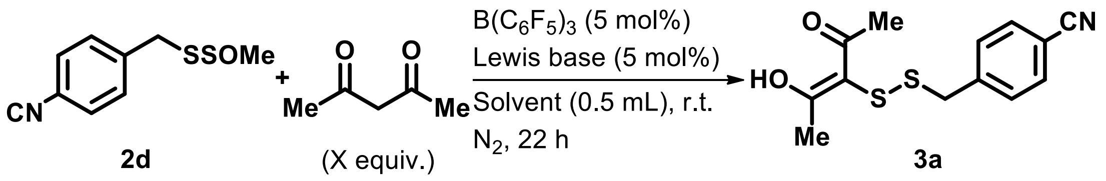

Entry	X (equiv.)	Lewis base (mol%)	Solvent	Yield (%)
1	2.4	-	PhMe	23
2	2.4	Et_3N/ TMP/ PMP[e]	PhMe	20/ 16/ 18
3	2.4	DMAP	PhMe	Complicated
4	2.4	4-MeOPy	PhMe	59
5	2.4	4-HOPy	PhMe	56
6	2.4	4-MePy	PhMe	42
7	2.4	2,4,6-Collidine	PhMe	20
8	2.4	2,4-diMeOPy	PhMe	22
9	2.4	4-MeOPy	DCE	76
10	2.4	4-MeOPy	THF	71
11	2.4	4-MeOPy	1,4-dioxane	41
12[c]	2.4	4-MeOPy	DCE	81
13[c]	1.1	4-MeOPy	DCE	82
14[c,d]	***1.1***	***4-MeOPy***	***DCE***	***85***

[a]**2d** (0.2 mmol, 1 equivalent), acetylacetone, $B(C_6F_5)_3$ (0.01 mmol, 5 mol%) and Lewis base (0.01 mmol, 5 mol%) were added to DCE at r.t. for 22 h. [b]Isolated yields. [c]DCE (0.25 mL). [d]Air atmosphere. [e]TMP = 2,2,6,6-tetramethylpiperidine, PMP = 1,2,2,6,6-pentamethylpiperidine.

To a Schlenk tube were added **2d** (0.2 mmol, 1 equivalent, 42.3 mg), acetylacetone, $B(C_6F_5)_3$ (0.01 mmol, 5 mol%, 5.2 mg), Lewis base (0.01 mmol, 5 mol%), and solvent, the mixture was stirred at r.t. before it was concentrated under vacuum. Purification by column chromatography afforded the desired product.

Supplementary Table 4. Optimization of disulfuration with indole.

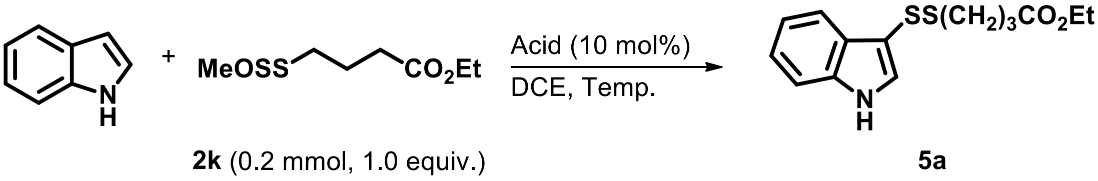

Entry	Acid	DCE /M	Temp. /^{o}C	Yield /%
1	TsOH	[0.2]	25	48
2[c]	TsOH	[0.2]	25	47
3[d]	TsOH	[0.2]	25	49
4	TsOH	[0.4]	25	53
5	TsOH	[0.8]	25	50
6	TsOH	[0.4]	0	60
7	TsOH	[0.4]	-10	55
8	CSA	[0.4]	0	50
9	4-$NO_2C_6H_4COOH$	[0.4]	0	NR
10	NsOH	[0.4]	0	52
11	4-$ClC_6H_4SO_3H$	[0.4]	0	30
12	4-$NH_2C_6H_4SO_3H$	[0.4]	0	trace
13	$C_6H_4SO_3H$	[0.4]	0	47

[a]**2k** (0.2 mmol, 1 equivalent), indole (0.4 mmol, 2 equivalents), and catalyst (0.02 mmol, 10 mol%) were added to DCE at r.t. for 5 h. [b]Isolated yields. [c]TsOH (5 mol%). [d] TsOH (10 mol%).

To a Schlenk tube were added **2k** (0.2 mmol, 1 equivalent, 42.1 mg), indole (0.4 mmol, 2 equivalents), catalyst (0.02 mmol, 10 mol%), and DCE, the mixture was stirred for 5 hours before it was concentrated under vacuum. Purification by column chromatography afforded the desired product.

Supplementary Table 5. Optimization of disulfuration with indole using Brønsted acids.

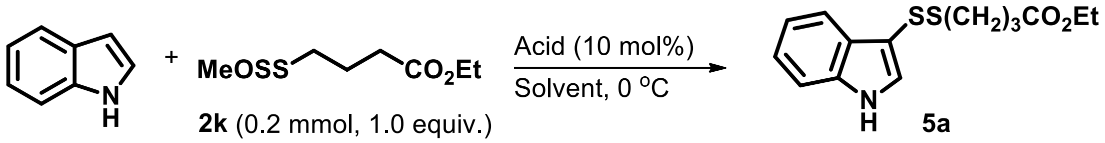

Entry	Indole /equiv.	Acid	Solvent /M	Yield /%
1	2.0	TsOH	THF	59
2	2.0	TsOH	PhCl	49
3	2.0	TsOH	MeCN	40
4	2.0	TsOH	$HCCl_3$	62
5	2.0	TsOH	MeOH	60
6	2.0	TsOH	Acetone	36
7	2.0	TsOH	EtOH	60
8	2.0	TsOH	Dioxane	60
9	2.0	TsOH	DMF	42
10	2.0	TsOH	PhMe	61
11	2.0	TsOH	EA	60
12	2.0	TsOH	^{i}PrOH	66
13	2.0	TsOH	^{n}PrOH	70
14	2.0	TsOH	^{n}BuOH	70
15	2.0	TsOH	^{i}BuOH	74
16	2.0	TsOH	^{t}AmylOH	74
17	2.0	TsOH	HFIP	52
18	1.5	TsOH	^{t}AmylOH	75
19	***1.5***	***$MeSO_3H$***	***^{t}AmylOH***	***78***
20	1.5	CSA	^{t}AmylOH	77

[a]**2k** (0.2 mmol, 1 equivalent), indole, and catalyst (0.02 mmol, 10 mol%) were added to solvent at 0 °C for 5 h. [b]Isolated yields.

To a Schlenk tube were added **2k** (0.2 mmol, 1 equivalent, 42.1 mg), indole (0.4 mmol, 2 equivalents), catalyst (0.02 mmol, 10 mol%), and solvent, the mixture was stirred at 0 °C for 5 hours before it was concentrated under vacuum. Purification by column chromatography afforded the desired product.

Supplementary Table 6. Optimization of disulfuration with indole using $B(C_6F_5)_3$.

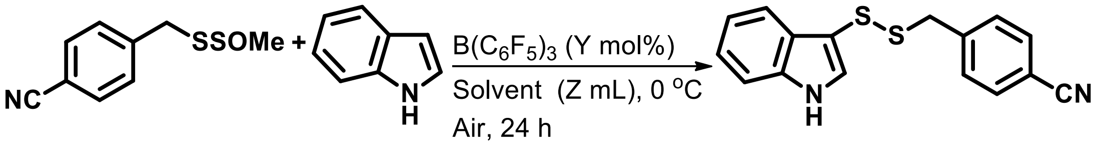

2d (X equiv.) 5u

Entry	X /equiv.	Y /mol%	Solvent /mL	Yield /%
1[c]	1.5	10	^{t}AmylOH (0.5)	79
2	1.5	2	PhMe (0.5)	83
3	1.2	2	PhMe (0.5)	81
4	1.2	2	PhMe (0.25)	87
5	1.1	2	PhMe (0.25)	86
6	***1.1***	***1***	***PhMe (0.25)***	***86***

[a]**2d** (0.2 mmol, 1 equivalent), indole, and catalyst were added to solvent at 0 °C for 24h. [b]Isolated yields. [c]$MeSO_3H$ (10 mol%).

To a Schlenk tube were added **2d** (0.2 mmol, 1 equivalent, 42.3 mg), indole, catalyst, and PhMe, the mixture was stirred at 0 °C for 24 hours before it was concentrated under vacuum. Purification by column chromatography afforded the desired product.

Supplementary Table 7. Optimization of disulfuration with aniline.

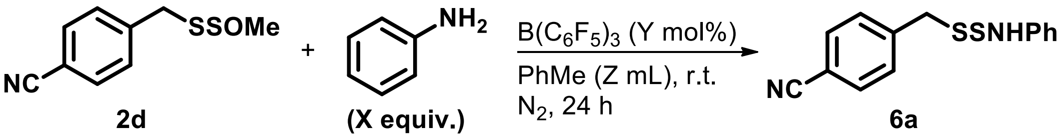

Entry	X/ equiv.	Y/ mol%	PhMe/ mL	Yield/ %
1	1.2	5	1.0	82
2	1.2	2	1.0	67
3	1.5	5	1.0	85
4	1.2	5	0.5	88
5	1.2	2.5	0.5	87
6	1.2	0	0.5	9
7[c]	***1.1***	***2.5***	***0.5***	***86***

[a]**2d** (0.2 mmol, 1 equivalent), $PhNH_2$, and $B(C_6F_5)_3$ were added to PhMe at r.t. for 24h. [b]Isolated yields. [c]Air atmosphere.

To a Schlenk tube were added **2d** (0.2 mmol, 1 equivalent, 42.3 mg), $PhNH_2$, $B(C_6F_5)_3$, and PhMe, the mixture was stirred at r.t. for 24 hours before it was concentrated under vacuum. Purification by column chromatography afforded the desired product.

General Procedure and Spectra Data of Polysulfuration

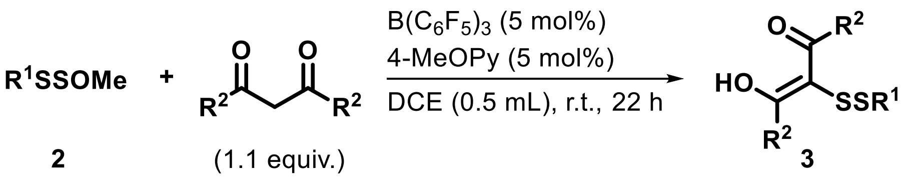

General procedure A: To a Schlenk tube were added 1,3-dicarbonyl compound (0.22 mmol, 1.1 equivalents), $B(C_6F_5)_3$ (0.01 mmol, 5 mol%, 5.2 mg), 4-MeO-pyridine (0.01 mmol, 5 mol%, 1.1 mg), RSSOMe (0.2 mmol, 1 equivalent), and 1,2-dichloroethane (0.25 mL), the mixture was stirred at r.t. for 22 hours before it was concentrated under vacuum. Purification by column chromatography afforded the desired product.

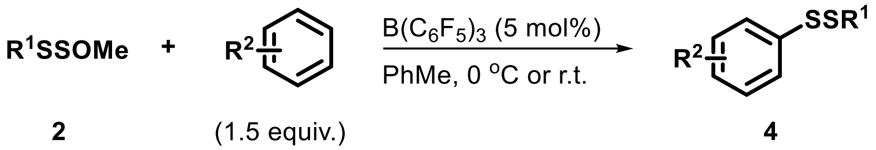

General procedure B: To a Schlenk tube were added arene (0.3mmol, 1.5 equivalents), $B(C_6F_5)_3$ (0.01 mmol, 5 mol%, 5.2 mg), RSSOMe (0.2 mmol, 1 equivalent), and toluene (0.5 mL), the mixture was stirred at 0 °C or r.t. for 24-60 hours before it was concentrated under vacuum. Purification by column chromatography afforded the desired product.

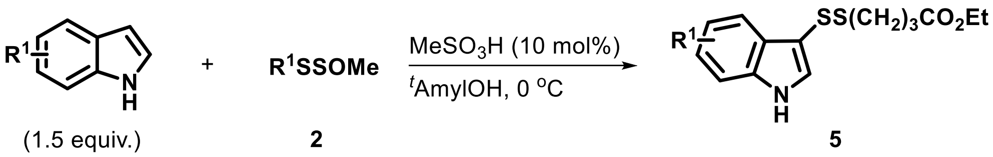

General procedure C_1: To a Schlenk tube were added indole (0.3 mmol, 1.5 equivalents), $MeSO_3H$ (0.02 mmol, 10 mol%, 2 mg), RSSOMe (0.2 mmol, 1 equivalent), and *t*-AmylOH (0.5 mL), the mixture was stirred at r.t. for 24 hours

before it was concentrated under vacuum. Purification by column chromatography afforded the desired product.

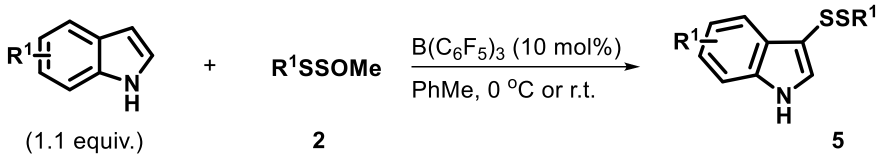

General procedure C$_2$: To a Schlenk tube were added indole (0.22 mmol, 1.1 equivalents), $B(C_6F_5)_3$ (0.004 mmol, 2 mol%, 2.1 mg), RSSOMe (0.2 mmol, 1 equivalent), and toluene (0.25 mL), the mixture was stirred at 0 °C or r.t. for 24 hours before it was concentrated under vacuum. Purification by column chromatography afforded the desired product.

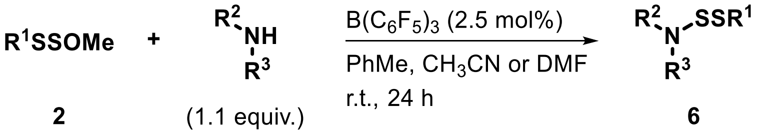

General procedure D: To a Schlenk tube were added amine (0.22 mmol, 1.1 equivalents), $B(C_6F_5)_3$ (0.01 mmol, 2.5 mol%, 2.6 mg), RSSOMe (0.2 mmol, 1 equivalent), and toluene/CH_3CN/DMF (0.5 mL), the mixture was stirred at 0 °C or r.t. for 24 hours before it was concentrated under vacuum. Purification by column chromatography afforded the desired product.

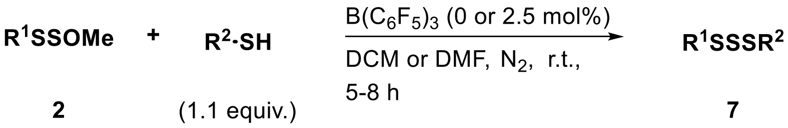

General procedure E: To a Schlenk tube were added thiol (0.22 mmol, 1.1 equivalents), $B(C_6F_5)_3$, RSSOMe (0.2 mmol, 1 equivalent), and DCM/DMF (0.5 mL), the mixture was stirred at r.t. under N_2 atmosphere for 5-8 hours before it was

concentrated under vacuum. Purification by column chromatography afforded the desired product.

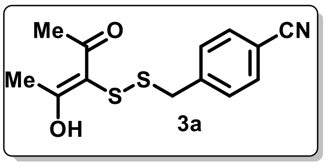

The reaction of acetylacetone (0.22 mmol, 1.1 equivalents, 22 mg), $B(C_6F_5)_3$ (0.01 mmol, 5 mol%, 5.2 mg), 4-MeO-pyridine (0.01 mmol, 5 mol%, 1.1 mg), 4-$NCC_6H_4CH_2SSOMe$ (0.2 mmol, 1 equivalent, 42.3 mg) in DCE (0.25 mL) at r.t. for 22 hours afforded compound **3a** in 85% yield (47.4 mg) as a white solid according to the general procedure A. **^{1}H NMR** (400 MHz, $CDCl_3$) δ 16.98 (s, 1H), 7.56 (d, J = 8.3 Hz, 2H), 7.38 (d, J = 8.2 Hz, 2H), 4.01 (s, 2H), 2.36 (s, 6H). **^{13}C NMR** (100 MHz, $CDCl_3$) δ 197.3, 142.1, 132.5, 129.7, 118.5, 111.5, 107.3, 42.1, 24.8. **IR** (film) 2974, 2905, 2229, 1737, 1405, 1253, 1227, 1052, 908, 845. **HRMS** (EI) Calcd for $C_{13}H_{13}NO_2S_2$ 279.0388, Found 279.0387.

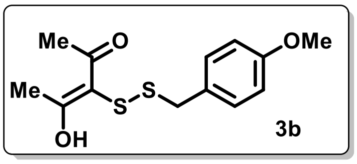

The reaction of acetylacetone (0.22 mmol, 1.1 equivalents, 22 mg), $B(C_6F_5)_3$ (0.01 mmol, 5 mol%, 5.2 mg), 4-MeO-pyridine (0.01 mmol, 5 mol%, 1.1 mg), 4-$MeOC_6H_4CH_2SSOMe$ (0.2 mmol, 1 equivalent, 43.3 mg) in DCE (0.25 mL) at r.t. for 22 hours afforded compound **3b** in 98% yield (55.7 mg) as a white solid according to the general procedure A. **^{1}H NMR** (400 MHz, $CDCl_3$) δ 17.04 (s, 1H), 7.23 (d, J = 8.6 Hz, 2H), 6.85 (d, J = 8.6 Hz, 2H), 4.04 (s, 2H), 3.80 (s, 3H), 2.45 (s, 6H). **^{13}C NMR** (100 MHz, $CDCl_3$) δ 197.3, 159.1, 130.3, 128.1, 114.1, 107.9, 55.3, 42.2, 24.8. **IR** (film) 2968, 2905, 1606, 1509, 1405, 1248, 1175, 1036, 827, 741, 684. **HRMS** (EI) Calcd for $C_{13}H_{16}O_3S_2$ 284.0541, Found 284.0540.

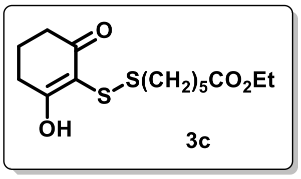

The reaction of 1,3-Cyclohexanedione (0.22 mmol, 1.1 equivalents, 24.7 mg), $B(C_6F_5)_3$ (0.01 mmol, 5 mol%, 5.2 mg), 4-MeO-pyridine (0.01 mmol, 5 mol%, 1.1 mg), $EtO_2C(CH_2)_5SSOMe$ (0.2 mmol, 1 equivalent, 47.7 mg) in DCE (0.25 mL) at r.t. for 22 hours afforded compound **3c** in 50% yield (31.9 mg) as a colorless oil according to the general procedure A. **^{1}H NMR** (400 MHz, $CDCl_3$) δ

7.82 (brs, 1H), 4.12 (q, J = 7.1 Hz, 2H), 2.94 (t, J = 7.3 Hz, 2H), 2.65 (s, 2H), 2.50 (s, 2H), 2.30 (t, J = 7.5 Hz, 2H), 2.06-1.95 (m, 2H), 1.78-1.71 (m, 2H), 1.69-1.61 (m, 2H), 1.47-1.39 (m, 2H), 1.25 (t, J = 7.1 Hz, 3H). **^{13}C NMR** (100 MHz, $CDCl_3$) δ 201.6, 173.6, 113.0, 60.2, 39.1, 34.2, 28.8, 27.9, 24.5, 19.7, 14.2. **IR** (film) 2937, 2865, 1730, 1658, 1562, 1373, 1174, 1135, 1029, 822, 732, 695. **HRMS** (EI) Calcd for $C_{14}H_{22}O_4S_2$ 318.0960, Found 318.0956.

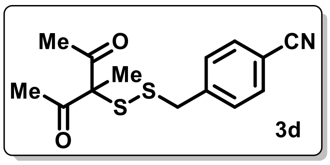

3d

The reaction of 3-methyl-2,4-pentanedione (0.22 mmol, 1.1 equivalents, 25.1 mg), $B(C_6F_5)_3$ (0.01 mmol, 5 mol%, 5.2 mg), 4-MeO-pyridine (0.01 mmol, 5 mol%, 1.1 mg), 4-$NCC_6H_4CH_2SSOMe$ (0.2 mmol, 1 equivalent, 42.3 mg) in DCE (0.25 mL) at r.t. for 22 hours afforded compound **3d** in 47% yield (27.6 mg) as a white solid according to the general procedure A. **^{1}H NMR** (400 MHz, $CDCl_3$) δ 7.61 (d, J = 8.1 Hz, 2H), 7.39 (d, J = 8.1 Hz, 2H), 3.89 (s, 2H), 2.26 (s, 6H), 1.67 (s, 3H). **^{13}C NMR** (100 MHz, $CDCl_3$) δ 201.9, 141.9, 132.4, 130.1, 118.6, 111.5, 73.7, 42.8, 26.8, 19.2. **IR** (film) 2980, 2905, 2228, 1690, 1447, 1253, 1073, 893, 757, 640. **HRMS** (EI) Calcd for $C_{14}H_{15}NO_2S_2$ 293.0544, Found 293.0549.

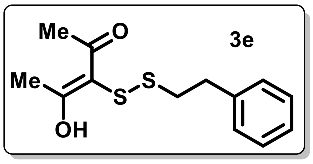

3e

The reaction of acetylacetone (0.22 mmol, 1.1 equivalents, 22 mg), $B(C_6F_5)_3$ (0.01 mmol, 5 mol%, 5.2 mg), 4-MeO-pyridine (0.01 mmol, 5 mol%, 1.1 mg), $C_6H_4CH_2CH_2SSOMe$ (0.2 mmol, 1 equivalent, 42.3 mg) in DCE (0.25 mL) at r.t. for 22 hours afforded compound **3e** in 67% yield (40.1 mg) as a colorless oil according to the general procedure A. **^{1}H NMR** (400 MHz, $CDCl_3$) δ 17.05 (s, 1H), 7.32 (t, J = 7.3 Hz, 2H), 7.26-7.17 (m, 3H), 3.03 (s, 4H), 2.49 (s, 6H). **^{13}C NMR** (100 MHz, $CDCl_3$) δ 197.3, 139.6, 128.6, 128.5, 126.6, 107.7, 38.8, 35.7, 24.8. **IR** (film) 2977, 2908, 1560, 1400, 1254, 1049, 907, 750, 701, 655. **HRMS** (EI) Calcd for $C_{13}H_{16}O_2S_2$ 268.0592, Found 268.0591.

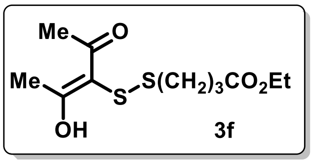

3f

The reaction of acetylacetone (0.22 mmol, 1.1 equivalents, 22 mg), $B(C_6F_5)_3$ (0.01 mmol, 5 mol%, 5.2 mg), 4-MeO-pyridine (0.01 mmol, 5 mol%, 1.1 mg),

$EtO_2C(CH_2)_3SSOMe$ (0.2 mmol, 1 equivalent, 42.1 mg) in DCE (0.25 mL) at r.t. for 22 hours afforded compound **3f** in 80% yield (44.5 mg) as a colorless oil according to the general procedure A. **^{1}H NMR** (400 MHz, $CDCl_3$) δ 17.01 (s, 1H), 4.11 (q, J = 7.1 Hz, 2H), 2.78 (t, J = 7.1 Hz, 2H), 2.45 (s, 6H), 2.41 (t, J = 7.3 Hz, 2H), 2.03 (p, J = 7.1 Hz, 2H), 1.24 (t, J = 7.1 Hz, 3H). **^{13}C NMR** (100 MHz, $CDCl_3$) δ 197.4, 172.7, 107.5, 60.4, 36.4, 32.6, 24.7, 24.3, 14.2. **IR** (film) 2980, 2914, 1732, 1564, 1406, 1120, 1041, 908, 790, 653. **HRMS** (EI) Calcd for $C_{11}H_{18}O_4S_2$ 278.0647, Found 278.0652.

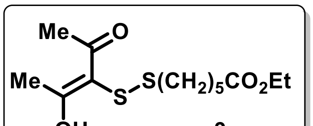

3g

The reaction of acetylacetone (0.22 mmol, 1.1 equivalents, 22 mg), $B(C_6F_5)_3$ (0.01 mmol, 5 mol%, 5.2 mg), 4-MeO-pyridine (0.01 mmol, 5 mol%, 1.1 mg), $EtO_2C(CH_2)_5SSOMe$ (0.2 mmol, 1 equivalent, 47.7 mg) in DCE (0.25 mL) at r.t. for 22 hours afforded compound **3g** in 84% yield (46.8 mg) as a colorless oil according to the general procedure A. **^{1}H NMR** (400 MHz, $CDCl_3$) δ 17.00 (s, 1H), 4.10 (q, J = 7.1 Hz, 2H), 2.74 (t, J = 7.2 Hz, 2H), 2.44 (s, 6H), 2.28 (t, J = 7.4 Hz, 2H), 1.74-1.67 (m, 2H), 1.67-1.58 (m, 2H), 1.44-1.37 (m, 2H), 1.23 (t, J = 7.2 Hz, 3H). **^{13}C NMR** (100 MHz, $CDCl_3$) δ 197.3, 173.4, 107.7, 60.2, 37.2, 34.0, 28.8, 28.0, 24.7, 24.4, 14.2. **IR** (film) 2936, 1733, 1568, 1425, 1182, 987, 915, 732, 653. **HRMS** (EI) Calcd for $C_{13}H_{22}O_4S_2$ 306.0960, Found 306.0957.

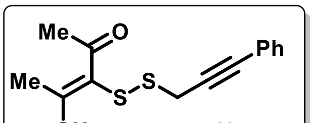

3h

The reaction of acetylacetone (0.22 mmol, 1.1 equivalents, 22 mg), $B(C_6F_5)_3$ (0.01 mmol, 5 mol%, 5.2 mg), 4-MeO-pyridine (0.01 mmol, 5 mol%, 1.1 mg), $C_6H_5CCCH_2SSOMe$ (0.2 mmol, 1 equivalent, 42.1 mg) in DCE (0.25 mL) at r.t. for 22 hours afforded compound **3h** in 44% yield (24.5 mg) as a colorless oil according to the general procedure A. **^{1}H NMR** (400 MHz, $CDCl_3$) δ 17.10 (s, 1H), 7.43-7.40 (m, 2H), 7.34-7.29 (m, 3H), 3.76 (s, 2H), 2.53 (s, 6H). **^{13}C NMR** (100 MHz, $CDCl_3$) δ 197.6, 131.7, 128.4, 128.3, 122.6, 107.1, 84.7, 84.5, 26.8, 24.8. **IR** (film) 2977, 2904, 2189, 1696, 1573, 1404, 1226, 1067, 891, 756, 692. **HRMS** (EI) Calcd for $C_{14}H_{14}O_2S_2$ 278.0435, Found 278.0428.

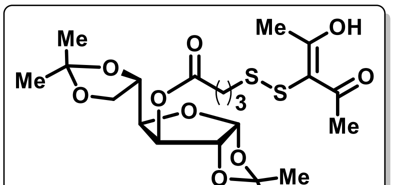

3i

The reaction of acetylacetone (0.22 mmol, 1.1 equivalents, 22 mg), $B(C_6F_5)_3$ (0.01 mmol, 5 mol%, 5.2 mg), 4-MeO-pyridine (0.01 mmol, 5 mol%, 1.1 mg), RSSOMe (0.2 mmol, 1 equivalent, 84.9 mg) in DCE (0.25 mL) at r.t. for 22 hours afforded compound **3i** in 75% yield (73.8 mg) as a colorless oil according to the general procedure A. **^{1}H NMR** (400 MHz, $CDCl_3$) δ 17.01 (s, 1H), 5.85 (d, J = 3.6 Hz, 1H), 5.25 (s, 1H), 4.46 (d, J = 3.6 Hz, 1H), 4.16 (s, 2H), 4.07-4.05 (m, 1H), 3.99 (d, J = 6.4 Hz, 1H), 2.78 (t, J = 6.8 Hz, 2H), 2.50-2.36 (m, 8H), 2.13-2.00 (m, 2H), 1.49 (s, 3H), 1.37 (s, 3H), 1.28 (s, 6H). **^{13}C NMR** (100 MHz, $CDCl_3$) δ 197.3, 171.3, 112.2, 109.3, 107.5, 105.0, 83.3, 79.8, 76.1, 72.4, 67.3, 36.2, 32.4, 26.8, 26.6, 26.1, 25.2, 24.7, 24.1. **IR** (film) 2980,2901, 1744, 1566, 1405, 1379, 1253, 1070, 887, 847, 738, 639. **HRMS** (EI) Calcd for $C_{21}H_{32}O_9S_2$ 492.1488, Found 492.1483.

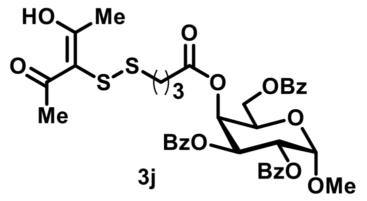

3j

The reaction of acetylacetone (0.22 mmol, 1.1 equivalents, 22 mg), $B(C_6F_5)_3$ (0.01 mmol, 5 mol%, 5.2 mg), 4-MeO-pyridine (0.01 mmol, 5 mol%, 1.1 mg), RSSOMe (0.2 mmol, 1 equivalent, 134.2 mg) in DCE (0.25 mL) at r.t. for 22 hours afforded compound **3j** in 81% yield (119.6 mg) as a white solid according to the general procedure A. **^{1}H NMR** (400 MHz, $CDCl_3$) δ 16.93 (s, 1H), 7.94 (d, J = 7.5 Hz, 2H), 7.90 (d, J = 7.5 Hz, 2H), 7.79 (d, J = 7.5 Hz, 2H), 7.49-7.31 (m, 5H), 7.29-7.17 (m, 4H), 5.79-5.75 (m, 2H), 5.52 (d of ABq, J = 10.5, 3.4 Hz, 1H), 5.14 (d, J = 3.3 Hz, 1H), 4.45-4.40 (m, 2H), 4.31-4,26 (m, 1H), 3.37 (s, 3H), 2.53 (dt, J = 17.8, 7.1 Hz, 4H), 2.31 (s, 6H), 2.00-1.86 (m, 2H). **^{13}C NMR** (100 MHz, $CDCl_3$) δ 197.3, 171.7, 165.9, 165.8, 165.3, 133.3, 133.2, 129.7, 129.6, 129.4, 129.4, 129.1, 129.1, 128.4, 128.3, 107.4, 97.5, 68.8, 68.5, 68.3, 66.4, 62.1, 55.6, 35.9, 32.1, 24.6, 24.6, 24.1. **IR** (film) 2977, 2907, 1723, 1597, 1449, 1404, 1261, 1067, 902, 709. **HRMS** (EI) Calcd for $C_{37}H_{38}O_{12}S_2$ 738.1805, Found 738.1799.

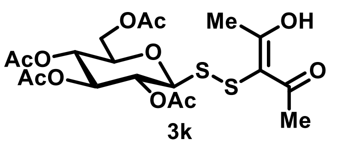

3k

The reaction of acetylacetone (0.22 mmol, 1.1 equivalents, 22 mg), $B(C_6F_5)_3$ (0.01 mmol, 5 mol%, 5.2 mg), 4-MeO-pyridine (0.01 mmol, 5 mol%, 1.1

mg), RSSOMe (0.2 mmol, 1 equivalent, 85.3 mg) in DCE (0.25 mL) at r.t. for 22 hours afforded compound **3k** in 60% yield (59.3 mg) as a white solid according to the general procedure A. **^{1}H NMR** (400 MHz, $CDCl_3$) δ 17.09 (s, 1H), 5.24-5.18 (m, 2H), 5.09 (t, J = 9.4 Hz, 1H), 4.65 (d, J = 9.3 Hz, 1H), 4.28 (d of ABq, J = 12.6, 4.6 Hz, 1H), 4.06 (d, J = 11.3 Hz, 1H), 3.73 (dd, J = 10.0, 2.6 Hz, 1H), 2.49 (s, 6H), 2.09 (s, 3H), 2.04 (s, 3H), 2.02 (s, 3H), 2.01 (s, 3H). **^{13}C NMR** (100 MHz, $CDCl_3$) δ 197.6, 170.5, 170.1, 169.3, 169.1, 107.5, 87.5, 76.6, 73.8, 69.6, 67.8, 61.9, 24.9, 20.7, 20.6, 20.5(3), 20.5(0). **IR** (film) 2996, 2954, 2895, 1745, 1567, 1370, 1213, 1083, 1052, 1032, 907, 733, 674. **HRMS** (ESI) Calcd for $C_{19}H_{26}O_{11}S_2$ (M+Na$^+$) 517.0809, Found 517.0809.

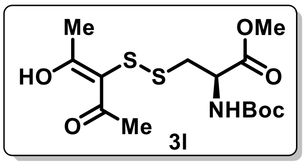

The reaction of acetylacetone (0.22 mmol, 1.1 equivalents, 22 mg), $B(C_6F_5)_3$ (0.01 mmol, 5 mol%, 5.2 mg), 4-MeO-pyridine (0.01 mmol, 5 mol%, 1.1 mg), RSSOMe (0.2 mmol, 1 equivalent, 59.5 mg) in DCE (0.25 mL) at r.t. for 22 hours afforded compound **3l** in 60% yield (58.4 mg) as a colorless oil according to the general procedure A. **^{1}H NMR** (400 MHz, $CDCl_3$) δ 17.07 (s, 1H), 5.30 (d, J = 6.9 Hz, 1H), 4.66 (d, J = 5.5 Hz, 1H), 3.76 (s, 3H), 3.25 (dd, J = 13.8, 4.4 Hz, 1H), 3.13 (dd, J = 13.8, 5.5 Hz, 1H), 2.45 (s, 6H), 1.43 (s, 9H). **^{13}C NMR** (100 MHz, $CDCl_3$) δ 197.6, 171.0, 155.0, 107.5, 80.3, 53.1, 52.7, 40.4, 28.2, 24.8. **IR** (film)3373, 2939, 1756, 1682, 1516, 1363, 1289, 1216, 1163, 1022, 976, 868, 781, 709. **HRMS** (EI) Calcd for $C_{14}H_{23}NO_6S_2$ 365.0967, Found 365.0965.

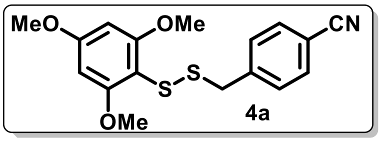

The reaction of 1,3,5-trimethoxybenzene (0.3mmol, 1.5 equivalents, 50.5 mg), $B(C_6F_5)_3$ (0.01 mmol, 5 mol%, 5.2 mg), 4-$NCC_6H_4CH_2$SSOMe (0.2 mmol, 1 equivalent, 42.3 mg) in toluene (0.5 mL) at 0 ^{o}C for 24 hours afforded compound **4a** in 70% yield (48.6 mg) as a white solid according to the general procedure B. **^{1}H NMR** (400 MHz, $CDCl_3$) δ 7.57 (d, J = 8.3 Hz, 2H), 7.46 (d, J = 8.3 Hz, 2H), 6.13 (s, 2H), 4.03 (s, 2H), 3.87 (s, 6H), 3.84 (s, 3H). **^{13}C NMR** (100 MHz, $CDCl_3$) δ 163.1, 162.2, 144.1, 132.1, 129.8, 119.0, 110.7, 104.4, 91.1, 56.2, 56.2, 55.5, 43.0. **IR** (film) 2967, 2925, 2225, 1580, 1451, 1407, 1227, 1120, 1081, 841, 808, 646. **HRMS** (EI) Calcd for $C_{17}H_{17}NO_3S_2$ 347.0650, Found 347.0645.

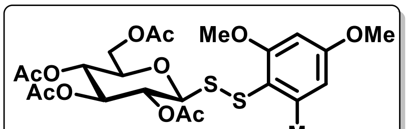

4b

The reaction of 1,3-dimethoxy-5-methylbenzene (0.3mmol, 1.5 equivalents, 45.7 mg), $B(C_6F_5)_3$ (0.01 mmol, 5 mol%, 5.2 mg), RSSOMe (0.2 mmol, 1 equivalent, 85.3 mg) in toluene (0.5 mL) at r.t. for 60 hours afforded compound **4b** in 48% yield (52.5 mg) as a white solid according to the general procedure B. **^{1}H NMR** (400 MHz, $CDCl_3$) δ 6.39 (d, J = 2.4 Hz, 1H), 6.32 (d, J = 2.4 Hz, 1H), 5.24-5.13 (m, 2H), 5.08 (t, J = 9.6 Hz, 1H), 4.84 (d, J = 9.8 Hz, 1H), 4.21 (d of ABq, J = 12.3, 4.8 Hz, 1H), 4.02 (d of ABq, J = 12.3, 2.4 Hz, 1H), 3.90 (s, 3H), 3.79 (s, 3H), 3.70 (ddd, J = 10.0, 4.7, 2.4 Hz, 1H), 2.52 (s, 3H), 2.05 (s, 3H), 2.02 (s, 3H), 2.00 (s, 3H), 1.87 (s, 3H). **^{13}C NMR** (100 MHz, $CDCl_3$) δ 170.6, 170.2, 169.4, 169.2, 161.9, 161.6, 145.3, 115.4, 107.2, 96.7, 89.8, 76.2, 74.0, 70.0, 68.3, 62.2, 56.1, 55.3, 21.9, 20.7, 20.5(9), 20.5(6), 20.4(6). **IR** (film) 2977, 2903, 1750, 1588, 1455, 1375, 1223, 1048, 907, 811, 733. **HRMS** (EI) Calcd for $C_{23}H_{30}O_{11}S_2$ 546.1230, Found 546.1238.

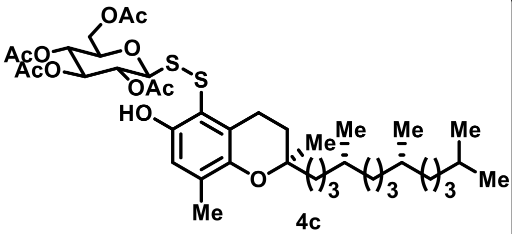

4c

The reaction of (+)-d-tocopherol (0.3mmol, 1.5 equivalents, 120.8 mg), $B(C_6F_5)_3$ (0.01 mmol, 5 mol%, 5.2 mg), RSSOMe (0.2 mmol, 1 equivalent, 85.3 mg) in toluene (0.5 mL) at r.t. for 60 hours afforded compound **4c** in 40% yield (63.7 mg) as a white solid according to the general procedure B. **^{1}H NMR** (400 MHz, $CDCl_3$) δ 6.66 (s, 1H), 6.45 (s, 1H), 5.26-5.10 (m, 3H), 4.56 (d, J = 9.6 Hz, 1H), 4.29-4.18 (m, 2H), 3.86-3.82 (m, 1H), 3.01-2.77 (m, 2H), 2.14 (s, 3H), 2.11 (s, 3H), 2.03 (s, 3H), 2.00 (s, 3H), 2.00 (s, 3H), 1.84-1.72 (m, 2H), 1.57-1.46 (m, 3H), 1.42-1.32 (m, 4H), 1.31-1.19 (m, 10H), 1.18-0.99 (m, 7H), 0.90-0.79 (m, 12H). **^{13}C NMR** (100 MHz, $CDCl_3$) δ 170.7, 170.1, 169.3, 169.2, 151.1, 146.3, 132.4, 124.3, 115.6, 115.0, 84.4, 76.6, 75.2, 73.7, 70.1, 68.2, 61.9, 39.9, 39.4, 37.5(1), 37.4(5), 37.4, 37.3, 32.8, 32.7, 31.3, 28.0, 24.8, 24.4, 23.7, 22.7, 22.6, 22.2, 21.0, 20.7(1) 20.7(0), 20.6, 19.7, 19.6, 16.5(1), 16.4(9). **IR** (film) 3449, 2958, 2929, 1752, 1458, 1372, 1217, 1048, 911, 734, 646. **HRMS** (EI) Calcd for $C_{41}H_{64}O_{11}S_2$ 796.3890, Found 796.3884.

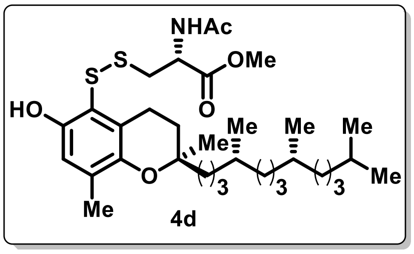

The reaction of (+)-d-tocopherol (0.3mmol, 1.5 equivalents, 120.8 mg), $B(C_6F_5)_3$ (0.01 mmol, 5 mol%, 5.2 mg), RSSOMe (0.2 mmol, 1 equivalent, 47.9 mg) in toluene (0.5 mL) at r.t. for 60 hours afforded compound **4d** in 53% yield (64.5 mg) as a white solid according to the general procedure B. **^{1}H NMR** (400 MHz, $CDCl_3$) δ 6.69 (s, 1H), 6.64-6.15 (m, 2H), 4.95 (dt, J = 10.3, 5.1 Hz, 1H), 3.79 (s, 3H), 3.34-3.23 (m, 2H), 2.90-2.85 (m, 2H), 2.14 (s, 3H), 2.03 (s, 3H), 1.84-1.73 (m, 2H), 1.56-1.02 (m, 24H), 0.90-0.76 (m, 12H). **^{13}C NMR** (100 MHz, $CDCl_3$) δ 171.0, 170.3, 150.6, 146.4, 131.9, 123.9, 116.1, 115.6, 75.2, 52.9, 52.4, 40.4, 40.0, 39.3, 37.4, 37.3(8), 37.3, 32.8, 32.7, 31.3, 27.9, 24.8, 24.4, 23.7, 23.1, 22.7, 22.6, 22.0, 20.9, 19.7, 19.6, 16.5, 16.4(5). **IR** (film) 3299, 2978, 2902, 1747, , 1462, 1555, 1446, 1406, 1253, 1957, 874, 754, 648. **HRMS** (EI) Calcd for $C_{33}H_{55}NO_5S_2$ 609.3522, Found 609.3527.

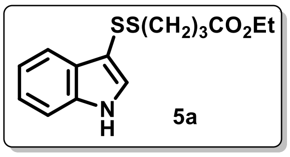

The reaction of indole (0.3 mmol, 1.5 equivalents), $MeSO_3H$ (0.02 mmol, 10 mol%, 2.0 mg), $EtO_2C(CH_2)_3SSOMe$ (0.2 mmol, 1 equivalent, 42.1 mg) in *t*-AmylOH (0.5 mL) at 0 ^oC for 5 hours afforded compound **5a** in 78% yield (46.0 mg) as a white solid according to the general procedure C_1. **^{1}H NMR** (400 MHz, $CDCl_3$) δ 8.47 (s, 1H), 7.83-7.81 (m, 1H), 7.38-7.34 (m, 2H), 7.27-7.21 (m, 2H), 4.12 (q, J = 7.1 Hz, 2H), 2.76 (t, J = 7.0 Hz, 2H), 2.41 (t, J = 7.3 Hz, 2H), 2.10 (p, J = 7.2 Hz, 2H), 1.24 (t, J = 7.1 Hz, 3H). **^{13}C NMR** (100 MHz, $CDCl_3$) δ 173.2, 136.3, 130.1, 128.6, 123.2, 121.1, 119.5, 111.6, 108.1, 60.4, 37.6, 32.8, 23.8, 14.3. **IR** (film) 3398, 2926, 1714, 1497, 1453, 1410, 1375, 1340, 1277,1211, 1131, 1095, 1035, 745. **HRMS** (EI) Calcd for $C_{15}H_{19}NO_2S_2$ 295.0701, Found 295.0705.

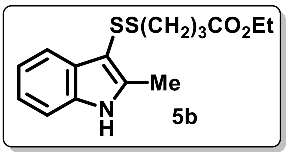

The reaction of 2-methylindole (0.3 mmol, 1.5 equivalents, 39.4 mg), $MeSO_3H$ (0.02 mmol, 10 mol%, 2 mg), $EtO_2C(CH_2)_3SSOMe$ (0.2 mmol, 1 equivalent, 42.1 mg) in *t*-AmylOH (0.5 mL) at 0 ^oC for 5 hours afforded compound **5b** in 80% yield (49.2 mg) as a white solid according to the general procedure C_1. **^{1}H NMR** (400 MHz, DMSO-d_6) δ 7.93 (d, J = 1.6 Hz, 1H), 7.73 (d, J = 2.7 Hz, 1H), 7.46 (dd, J = 8.5, 1.7 Hz, 1H), 7.31 (d, J = 8.4 Hz, 1H), 4.05 (q, J = 7.1 Hz, 2H), 3.32 (s,

3H), 2.74 (t, J = 7.3 Hz, 2H), 2.37 (t, J = 7.3 Hz, 2H), 1.96 (p, J = 7.3 Hz, 2H), 1.17 (t, J = 7.1 Hz, 3H). **^{13}C NMR** (100 MHz, DMSO-d$_6$) δ 172.3, 135.6, 133.1, 130.9, 130.2, 126.8, 114.7, 103.9, 84.3, 59.8, 36.6, 32.0, 23.6, 14.1. **IR** (film) 3305, 2927, 1707, 1539, 1449, 1399, 1309, 1226, 1183, 1020, 859, 744, 674. **HRMS** (EI) Calcd for $C_{15}H_{19}NO_2S_2$ 309.0857, Found 309.0851.

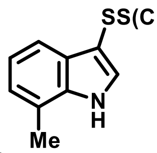

5c

The reaction of 7-methylindole (0.3 mmol, 1.5 equivalents, 39.4 mg), $MeSO_3H$ (0.02 mmol, 10 mol%, 2 mg), $EtO_2C(CH_2)_3SSOMe$ (0.2 mmol, 1 equivalent, 42.1 mg) in *t*-AmylOH (0.5 mL) at 0 ^{o}C for 5 hours afforded compound **5c** in 71% yield (43.9 mg) as a white solid according to the general procedure C_1. **^{1}H NMR** (400 MHz, $CDCl_3$) δ 8.40 (s, 1H), 7.68 (d, J = 7.9 Hz, 1H), 7.41 (d, J = 2.6 Hz, 1H), 7.17 (t, J = 7.5 Hz, 1H), 7.06 (d, J = 7.1 Hz, 1H), 4.14 (q, J = 7.1 Hz, 2H), 2.78 (t, J = 7.0 Hz, 2H), 2.48 (s, 3H), 2.43 (t, J = 7.3 Hz, 2H), 2.12 (p, J = 7.2 Hz, 2H), 1.26 (t, J = 7.1 Hz, 3H). **^{13}C NMR** (100 MHz, $CDCl_3$) δ 173.2, 135.8, 129.9, 128.2, 123.6, 121.1, 120.8, 117.1, 108.3, 60.4, 37.5, 32.8, 23.8, 16.4, 14.2. **IR** (film) 3303, 2929, 1718, 1612, 1497, 1417, 1375, 1346, 1213, 1178, 1031, 779, 747, 666. HRMS (EI) Calcd for $C_{15}H_{19}NO_2S_2$ 309.0857, Found 309.0862.

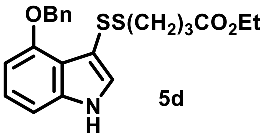

5d

The reaction of 4-benzyloxyindole (0.3 mmol, 1.5 equivalents, 67.0 mg), $MeSO_3H$ (0.02 mmol, 10 mol%, 2 mg), $EtO_2C(CH_2)_3SSOMe$ (0.2 mmol, 1 equivalent, 42.1 mg) in *t*-AmylOH (0.5 mL) at 0 ^{o}C for 5 hours afforded compound **5d** in 75% yield (60.1 mg) as a white solid according to the general procedure C_1. **^{1}H NMR** (400 MHz, $CDCl_3$) δ 8.39 (s, 1H), 7.59 (d, J = 7.4 Hz, 2H), 7.37 (t, J = 7.4 Hz, 2H), 7.29 (d, J = 7.3 Hz, 1H), 7.22 (d, J = 4.5 Hz, 1H), 7.08 (t, J = 7.9 Hz, 1H), 6.92 (d, J = 8.1 Hz, 1H), 6.62 (d, J = 7.8 Hz, 1H), 5.23 (s, 2H), 4.06 (q, J = 7.1 Hz, 2H), 2.71 (t, J = 7.0 Hz, 2H), 2.29 (t, J = 7.3 Hz, 2H), 2.03 - 1.68 (m, 2H), 1.20 (t, J = 7.1 Hz, 3H). **^{13}C NMR** (100 MHz, $CDCl_3$) δ 173.3, 153.4, 138.4, 137.3, 128.9, 128.4, 127.5, 127.1, 123.9, 117.7, 107.5, 105.1, 102.7, 70.1, 60.3, 36.5, 32.7, 24.1, 14.2. **IR** (film) 3376, 2982, 1726, 1583, 1508, 1314, 1247, 1087, 778, 736, 696. **HRMS** (EI) Calcd for $C_{21}H_{23}NO_3S_2$ 401.1119, Found 401.1124.

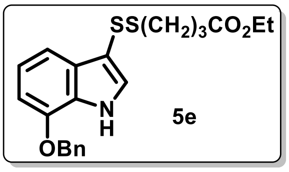

The reaction of 7-benzyloxyindole (0.3 mmol, 1.5 equivalents, 67.0 mg), $MeSO_3H$ (0.02 mmol, 10 mol%, 2 mg), $EtO_2C(CH_2)_3SSOMe$ (0.2 mmol, 1 equivalent, 42.1 mg) in *t*-AmylOH (0.5 mL) at 0 °C for 5 hours afforded compound **5e** in 85% yield (68.2 mg) as a white solid according to the general procedure C_1. **^{1}H NMR** (400 MHz, $CDCl_3$) δ 8.59 (s, 1H), 7.70 - 7.32 (m, 7H), 7.14 (t, *J* = 7.9 Hz, 1H), 6.78 (d, *J* = 7.7 Hz, 1H), 5.20 (s, 2H), 4.13 (q, *J* = 7.1 Hz, 2H), 2.77 (t, *J* = 7.0 Hz, 2H), 2.42 (t, *J* = 7.3 Hz, 2H), 2.11 (p, *J* = 7.2 Hz, 2H), 1.25 (t, *J* = 7.1 Hz, 3H). **^{13}C NMR** (100 MHz, $CDCl_3$) δ 173.1, 145.5, 136.7, 130.1, 129.6, 128.6, 128.2, 127.8, 127.0, 121.4, 112.3, 108.3, 104.1, 70.3, 60.4, 37.5, 32.7, 23.7, 14.2. **IR** (film) 3395, 2983, 1719, 1579, 1410, 1310, 1252, 1081, 1006, 855, 780, 735, 696. **HRMS** (EI) Calcd for $C_{21}H_{23}NO_3S_2$ 401.1119, Found 401.1127.

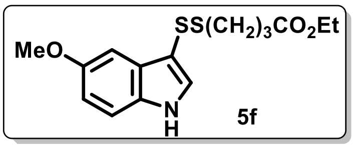

The reaction of 5-methoxyindole (0.3 mmol, 1.5 equivalents, 44.2 mg), $MeSO_3H$ (0.02 mmol, 10 mol%, 2 mg), $EtO_2C(CH_2)_3SSOMe$ (0.2 mmol, 1 equivalent, 42.1 mg) in *t*-AmylOH (0.5 mL) at 0 °C for 5 hours afforded compound **5f** in 77% yield (49.9 mg) as a white solid according to the general procedure C_1. **^{1}H NMR** (400 MHz, $CDCl_3$) δ 8.47 (s, 1H), 7.36 (d, *J* = 2.7 Hz, 1H), 7.25-7.23 (m, 2H), 6.90 (dd, *J* = 8.8, 2.5 Hz, 1H), 4.13 (q, *J* = 7.1 Hz, 2H), 3.89 (s, 3H), 2.77 (t, *J* = 7.0 Hz, 2H), 2.42 (t, *J* = 7.3 Hz, 2H), 2.11 (p, *J* = 7.2 Hz, 2H), 1.25 (t, *J* = 7.1 Hz, 3H). **^{13}C NMR** (100 MHz, $CDCl_3$) δ 173.2, 155.1, 131.2, 130.8, 129.2, 113.5, 112.5, 107.0, 100.7, 60.4, 55.8, 37.3, 32.7, 23.7, 14.2. **IR** (film) 3348, 2982, 1716, 1485, 1287, 1206, 1169, 1033, 747, 630. **HRMS** (EI) Calcd for $C_{15}H_{19}NO_3S_2$ 325.0806, Found 325.0811.

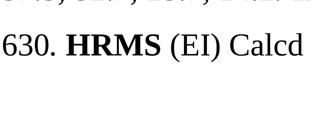

The reaction of 5-fluoroindole (0.3 mmol, 1.5 equivalents, 40.6 mg), $MeSO_3H$ (0.02 mmol, 10 mol%, 2 mg), $EtO_2C(CH_2)_3SSOMe$ (0.2 mmol, 1 equivalent, 42.1 mg) in *t*-AmylOH (0.5 mL) at 0 °C for 5 hours afforded compound **5g** in 68% yield (42.6 mg) as a white solid according to the general procedure C_1. **^{1}H NMR** (400 MHz, DMSO-d_6) δ 7.79 (d, *J* = 2.7 Hz, 1H), 7.45 (dd, *J* = 8.8, 4.4 Hz, 1H), 7.33 (dd, *J* = 9.5, 2.5 Hz, 1H), 7.04 (td, *J* = 9.2, 2.6 Hz, 1H), 4.05 (q, *J* = 7.1 Hz, 2H), 2.86 - 2.50 (m, 2H), 2.37 (t, *J* = 7.2 Hz, 2H), 1.96 (p, *J* = 7.3 Hz, 2H), 1.16 (t, *J* = 7.1

Hz, 3H). **^{19}F NMR** (376 MHz, DMSO-d_6) δ -123.02. **^{13}C NMR** (100 MHz, DMSO-d_6) δ 172.3, 157.8 (d, $^{1}J_{C\text{-}F}$ = 234.0 Hz), 134.1, 133.0, 129.0 (d, $^{3}J_{C\text{-}F}$ = 10.0 Hz), 113.5 (d, $^{3}J_{C\text{-}F}$ = 9.7 Hz), 110.5 (d, $^{2}J_{C\text{-}F}$ = 26.1 Hz), 104.8 (d, $^{4}J_{C\text{-}F}$ = 4.7 Hz, 3H), 103.1 (d, $^{2}J_{C\text{-}F}$ = 23.9 Hz), 59.8, 36.7, 32.0, 23.7, 14.0. **IR** (film) 3320, 2934, 1725, 1484, 1457, 1278, 1156, 1026, 929, 855, 800, 621. **HRMS** (EI) Calcd for $C_{14}H_{16}FNO_2S_2$ 313.0607, Found 313.0607.

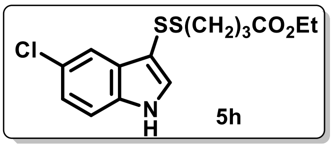

The reaction of 5-chloroindole (0.3 mmol, 1.5 equivalents, 44.2 mg), $MeSO_3H$ (0.02 mmol, 10 mol%, 2 mg), $EtO_2C(CH_2)_3SSOMe$ (0.2 mmol, 1 equivalent, 42.1 mg) in *t*-AmylOH (0.5 mL) at 0 ^{o}C for 5 hours afforded compound **5h** in 62% yield (40.8 mg) as a white solid according to the general procedure C_1. **^{1}H NMR** (400 MHz, DMSO-d_6) δ 11.81 (s, 1H), 7.80 (s, 1H), 7.60 (d, *J* = 2.0 Hz, 1H), 7.47 (d, *J* = 8.6 Hz, 1H), 7.20 (dd, *J* = 8.6, 2.1 Hz, 1H), 4.05 (q, *J* = 7.1 Hz, 2H), 2.87 - 2.66 (m, 2H), 2.37 (t, *J* = 7.3 Hz, 2H), 1.96 (p, *J* = 7.3 Hz, 2H), 1.16 (t, *J* = 7.1 Hz, 3H). **^{13}C NMR** (100 MHz, DMSO-d_6) δ 172.3, 134.9, 133.8, 129.5, 125.1, 122.3, 117.5, 114.0, 104.4, 59.8, 36.7, 32, 23.6, 14.0. **IR** (film) 3264, 2990, 1695, 1408, 1315, 1230, 1187, 1103, 1018, 866, 799, 703. **HRMS** (EI) Calcd for $C_{14}H_{16}ClNO_2S_2$ 329.0311, Found 329.0313.

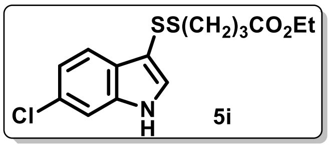

The reaction of 6-chloroindole (0.3 mmol, 1.5 equivalents, 44.2 mg), $MeSO_3H$ (0.02 mmol, 10 mol%, 2 mg), $EtO_2C(CH_2)_3SSOMe$ (0.2 mmol, 1 equivalent, 42.1 mg) in *t*-AmylOH (0.5 mL) at 0 ^{o}C for 5 hours afforded compound **5i** in 63% yield (41.5 mg) as a white solid according to the general procedure C_1. **^{1}H NMR** (400 MHz, $CDCl_3$) δ 8.56 (s, 1H), 7.72 (d, *J* = 8.4 Hz, 1H), 7.52 - 7.29 (m, 2H), 7.20 (d, *J* = 8.2 Hz, 1H), 4.13 (q, *J* = 7.1 Hz, 2H), 2.76 (t, *J* = 7.0 Hz, 2H), 2.41 (t, *J* = 7.2 Hz, 2H), 2.29 - 1.90 (m, 2H), 1.25 (t, *J* = 7.1 Hz, 3H). **^{13}C NMR** (100 MHz, $CDCl_3$) δ 173.2, 136.6, 130.6, 129.0, 127.1, 121.7, 120.4, 111.6, 108.2, 60.5, 37.5, 32.7, 23.7, 14.2. **IR** (film) 3264, 2925, 1705, 1308, 1220, 1176, 1176, 1012, 903, 852, 807, 780, 695. **HRMS** (EI) Calcd for $C_{14}H_{16}ClNO_2S_2$ 329.0311 Found 329.0310.

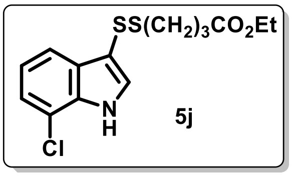

The reaction of 7-chloroindole (0.3 mmol, 1.5 equivalents, 44.2 mg), $MeSO_3H$ (0.02 mmol, 10 mol%, 2 mg), $EtO_2C(CH_2)_3SSOMe$ (0.2 mmol, 1 equivalent, 42.1 mg) in *t*-AmylOH (0.5 mL) at 0 ^oC for 5 hours afforded compound **5j** in 68% yield (44.8 mg) as a white solid according to the general procedure C_1. **^{1}H NMR** (400 MHz, $CDCl_3$) δ 8.61 (s, 1H), 7.73 (d, *J* = 7.9 Hz, 1H), 7.50 (d, *J* = 2.6 Hz, 1H), 7.28 - 7.22 (m, 1H), 7.17 (t, *J* = 7.8 Hz, 1H), 4.13 (q, *J* = 7.1 Hz, 2H), 2.77 (t, *J* = 7.1 Hz, 2H), 2.41 (t, *J* = 7.2 Hz, 2H), 2.10 (p, *J* = 7.2 Hz, 2H), 1.25 (t, *J* = 7.1 Hz, 3H). **^{13}C NMR** (100 MHz, $CDCl_3$) δ 173.1, 133.5, 130.5, 130.0, 122.5, 121.8, 118.2, 117.0, 109.4, 60.4, 37.5, 32.6, 23.7, 14.2. **IR** (film) 3317, 2923, 1705, 1411, 1220, 1194, 1135, 1024, 835, 776, 734, 685. **HRMS** (EI) Calcd for $C_{14}H_{16}ClNO_2S_2$ 329.0311, Found 329.0314.

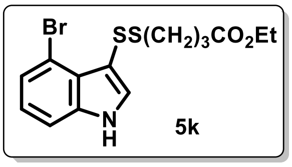

The reaction of 4-bromoindole (0.3 mmol, 1.5 equivalents, 58.9 mg), $MeSO_3H$ (0.02 mmol, 10 mol%, 2 mg), $EtO_2C(CH_2)_3SSOMe$ (0.2 mmol, 1 equivalent, 42.1 mg) in *t*-AmylOH (0.5 mL) at 0 ^oC for 5 hours afforded compound **5k** in 63% yield (47.1 mg) as a white solid according to the general procedure C_1. **^{1}H NMR** (400 MHz, DMSO-d_6) δ 11.92 (s, 1H), 7.80 (d, *J* = 2.8 Hz, 1H), 7.47 (dd, *J* = 8.1, 0.7 Hz, 1H), 7.30 (dd, *J* = 7.6, 0.7 Hz, 1H), 7.07 (t, *J* = 7.9 Hz, 1H), 4.05 (q, *J* = 7.1 Hz, 2H), 2.74 (t, *J* = 7.2 Hz, 2H), 2.37 (t, *J* = 7.3 Hz, 2H), 1.98 (p, *J* = 7.3 Hz, 2H), 1.16 (t, *J* = 7.1 Hz, 3H). **^{13}C NMR** (100 MHz, DMSO-d_6) δ 172.3, 137.9, 134.6, 124.7, 124.7, 123.3, 113.2, 112.1, 105.2, 59.8, 35.2, 32.1, 24.0, 14. **IR** (film) 3100, 2933, 1725, 1436, 1306, 1183, 1001, 911, 776, 739. **HRMS** (EI) Calcd for $C_{14}H_{16}BrNO_2S_2$ 372.9806, Found 372.9804.

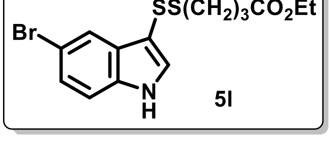

The reaction of 5-bromoindole (0.3 mmol, 1.5 equivalents, 58.9 mg), $MeSO_3H$ (0.02 mmol, 10 mol%, 2 mg), $EtO_2C(CH_2)_3SSOMe$ (0.2 mmol, 1 equivalent, 42.1 mg) in *t*-AmylOH (0.5 mL) at 0 ^oC for 5 hours afforded compound **5l** in 58% yield (43.4 mg) as a white solid according to the general procedure C_1. **^{1}H NMR** (400 MHz, DMSO-d_6) δ 11.82 (s, 1H), 7.79 (d, *J* = 2.7 Hz, 1H), 7.74 (d, *J* = 1.8 Hz, 1H), 7.44-7.42 (m, 1H), 7.33 - 7.26 (m, 1H), 4.05 (q, *J* = 7.1

Hz, 2H), 2.74 (t, *J* = 7.2 Hz, 2H), 2.37 (t, *J* = 7.3 Hz, 2H), 1.96 (p, *J* = 7.3 Hz, 2H), 1.17 (t, *J* = 7.1 Hz, 3H). **^{13}C NMR** (100 MHz, DMSO-d$_6$) δ 172.3, 135.2, 133.6, 130.1, 124.8, 120.6, 114.4, 113, 104.3, 59.8, 36.6, 32.0, 23.6, 14.0. **IR** (film) 3154, 2931, 1726, 1450, 1292, 1106, 1025, 881, 799, 756. **HRMS** (EI) Calcd for $C_{14}H_{16}BrNO_2S_2$ 372.9806, Found 372.9809.

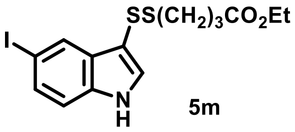

5m

The reaction of 5-iodoindole (0.3 mmol, 1.5 equivalents, 72.9 mg), $MeSO_3H$ (0.02 mmol, 10 mol%, 2 mg), $EtO_2C(CH_2)_3SSOMe$ (0.2 mmol, 1 equivalent, 42.1 mg) in *t*-AmylOH (0.5 mL) at 0 °C for 5 hours afforded compound **5m** in 53% yield (44.6 mg) as a white solid according to the general procedure C_1. **^{1}H NMR** (400 MHz, DMSO-d$_6$) δ 11.78 (s, 1H), 7.93 (d, *J* = 1.6 Hz, 1H), 7.73 (d, *J* = 2.7 Hz, 1H), 7.46 (dd, *J* = 8.5, 1.7 Hz, 1H), 7.31 (d, *J* = 8.4 Hz, 1H), 4.05 (q, *J* = 7.1 Hz, 2H), 2.74 (t, *J* = 7.2 Hz, 2H), 2.37 (t, *J* = 7.3 Hz, 2H), 1.96 (p, *J* = 7.3 Hz, 2H), 1.17 (t, *J* = 7.1 Hz, 3H). **^{13}C NMR** (100 MHz, DMSO-d$_6$) δ 172.3, 135.5, 133.1, 130.9, 130.2, 126.8, 114.7, 103.9, 84.3, 59.8, 36.6, 32.0, 23.6, 14.1. **IR** (film) 3328, 2935, 1724, 1446, 1292, 1208, 1136, 1027, 876, 797. **HRMS** (EI) Calcd for $C_{14}H_{16}INO_2S_2$ 420.9667, Found 420.9673.

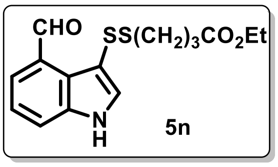

5n

The reaction of indole-4-carboxaldehyde (0.3 mmol, 1.5 equivalents, 43.6 mg), $MeSO_3H$ (0.02 mmol, 10 mol%, 2 mg), $EtO_2C(CH_2)_3SSOMe$ (0.2 mmol, 1 equivalent, 42.1 mg) in *t*-AmylOH (0.5 mL) at 0 °C for 24 hours afforded compound **5n** in 39% yield (25.2 mg) as a white solid according to the general procedure C_1. **^{1}H NMR** (400 MHz, $CDCl_3$) δ 11.44 (s, 1H), 9.20 (s, 1H), 7.91 (d, *J* = 7.5 Hz, 1H), 7.72 - 7.57 (m, 2H), 7.34 (t, *J* = 7.7 Hz, 1H), 4.11 (q, *J* = 7.1 Hz, 2H), 2.73 (t, *J* = 7.1 Hz, 2H), 2.40 (t, *J* = 7.2 Hz, 2H), 2.06 (p, *J* = 7.3 Hz, 2H), 1.24 (t, *J* = 7.1 Hz, 4H). **^{13}C NMR** (100 MHz, $CDCl_3$) δ 192.2, 173.2, 138.2, 133.8, 130.1, 127.4, 122.8, 121.2, 117.9, 106.3, 60.5, 36.2, 32.6, 23.8, 14.2. **IR** (film) 3314, 2932, 1728, 1673, 1609, 1414, 1386, 1345, 1259, 1210, 1126, 1035, 998, 792, 748. **HRMS** (EI) Calcd for $C_{15}H_{17}NO_3S_2$ 323.0650, Found 323.0655.

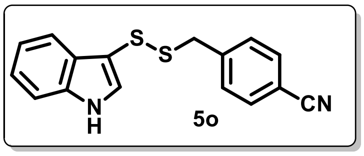

The reaction of indole (0.22 mmol, 1.1 equivalents, 25.8 mg), $B(C_6F_5)_3$ (0.002 mmol, 1 mol%, 1 mg), 4-NCC_6H_4SSOMe (0.2 mmol, 1 equivalent, 42.3 mg) in toluene (0.25 mL) at 0 °C for 24 hours afforded compound **5o** in 86% yield (51.0 mg) as a white solid according to the general procedure C_2. **^{1}H NMR** (400 MHz, DMSO-d_6) δ 11.65 (s, 1H), 7.79 (d, J = 6.8 Hz, 2H), 7.70 - 7.60 (m, 2H), 7.57 (d, J = 6.8 Hz, 2H), 7.45 (d, J = 8.0 Hz, 1H), 7.26 - 7.03 (m, 2H), 4.04 (s, 2H). **^{13}C NMR** (100 MHz, DMSO-d_6) δ 144.8, 137.5, 133.4, 133.2, 131.4, 129.2, 123.3, 121.2, 119.9, 119.5, 113.3, 110.8, 104.9, 42.1. **IR** (film) 3311, 2985, 2903, 2232, 1408, 1234, 1058, 868, 827, 772, 654. **HRMS** (EI) Calcd for $C_{16}H_{12}N_2S_2$ 296.0442, Found 296.0449.

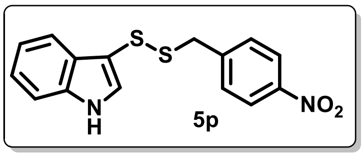

The reaction of indole (0.22 mmol, 1.1 equivalents, 25.8 mg), $B(C_6F_5)_3$ (0.002 mmol, 1 mol%, 1 mg), 4-$NO_2C_6H_4SSOMe$ (0.2 mmol, 1 equivalent, 46.3 mg) in toluene (0.25 mL) at 0 °C for 24 hours afforded compound **5p** in 99% yield (64.5 mg) as a white solid according to the general procedure C_2. **^{1}H NMR** (400 MHz, DMSO-d_6) δ 11.63 (s, 1H), 8.17 (d, J = 8.5 Hz, 2H), 7.71-7.53 (m, 4H), 7.44 (d, J = 8.0 Hz, 1H), 7.25-7.07 (m, 2H), 4.10 (s, 2H). **^{13}C NMR** (100 MHz, DMSO-d_6) δ146.47, 145.98, 136.41, 132.31, 130.51, 128.15, 123.35, 122.21, 120.20, 118.40, 112.22, 103.87, 40.8. **IR** (film) 3391, 2980, 2901, 1448, 1403, 1203, 1054, 891, 749. **HRMS** (EI) Calcd for $C_{15}H_{12}N_2O_2S_2$ 316.0340, Found 316.0339.

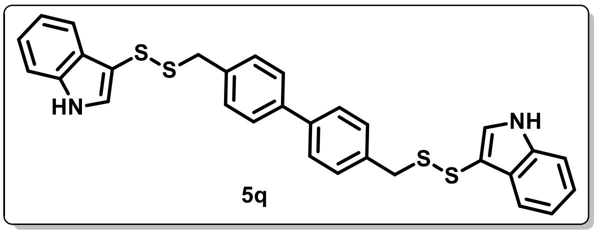

The reaction of indole (0.22 mmol, 2.2 equivalentss, 25.8 mg), $B(C_6F_5)_3$ (0.002 mmol, 2 mol%, 1 mg), 4-$MeOCH_2SSC_6H_4C_6H_4CH_2SSOMe$ (0.1 mmol, 1 equivalent, 37.1 mg) in toluene (0.25 mL) at 0 °C for 24 hours afforded compound **5q** in 35% yield (20 mg) as a white solid according to the general procedure C_2. **^{1}H NMR** (400 MHz, DMSO-d_6) δ 11.62 (s, 2H), 7.73-7.58 (m, 8H), 7.46-7.42 (m,, 6H), 7.22-7.13 (m, 4H), 4.06 (s, 4H). **^{13}C NMR** (100 MHz, DMSO-d_6) δ 138.7, 136.7, 136.5, 132.2, 129.9, 128.3,

126.6, 122.2(0), 120.2(2), 118.5, 112.3, 104.6, 41.9. **IR** (film) 3443, 3372, 2986, 2901, 1495, 1453, 1431, 1243, 1052, 874, 814, 750, 624. **HRMS** (ESI) Calcd for $C_{30}H_{24}N_2S_4$ (M+Na$^+$) 563.0715, Found 563.0712.

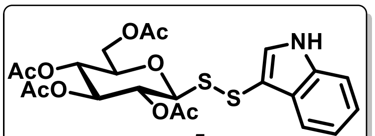

5r

The reaction of indole (0.22 mmol, 1.1 equivalents, 25.8 mg), $B(C_6F_5)_3$ (0.004 mmol, 2 mol%, 2.1 mg), RSSOMe (0.2 mmol, 1 equivalent, 85.3 mg) in toluene (0.25 mL) at r.t. for 24 hours afforded compound **5r** in 94% yield (96 mg) as a white solid according to the general procedure C_2. **^{1}H NMR** (400 MHz, $CDCl_3$) δ 8.78 (s, 1H), 7.92-7.66 (m, 1H), 7.44 (d, J = 1.4 Hz, 1H), 7.34-7.33 (m, 1H), 7.26-7.14 (m, 2H), 5.30-5.18 (m, 2H), 5.17-5.09 (m, 1H), 4.77-4.61 (m, 1H), 4.22 (d of ABq, J = 12.4, 4.4 Hz, 1H), 3.94 (d, J = 12.1 Hz, 1H), 3.83-3.60 (m, 1H), 2.01 (s, 6H), 1.98 (s, 3H), 1.75 (s, 3H). **^{13}C NMR** (100 MHz, $CDCl_3$) δ 170.6, 170.1, 169.4, 169.3, 136.0, 131.4, 128.5, 123.0, 120.8, 119.14, 111.7, 107.1, 88.8, 76.1, 73.8, 69.6, 68.0, 61.9, 20.6, 20.5, 20.5, 20.2. **IR** (film) 3392, 2988, 2948, 1744, 1371, 1215, 1036, 910, 744, 644. **HRMS** (EI) Calcd for $C_{22}H_{25}NO_9S_2$ 511.0971, Found 511.0977.

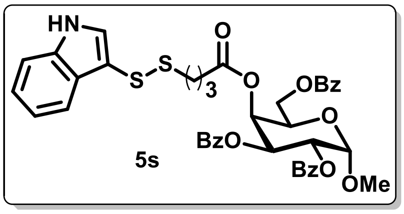

5s

The reaction of indole (0.22 mmol, 1.1 equivalents, 25.8 mg), $B(C_6F_5)_3$ (0.004 mmol, 2 mol%, 2.1 mg), RSSOMe (0.2 mmol, 1 equivalent, 134.2 mg) in toluene (0.25 mL) at 0 ^{o}C for 24 hours afforded compound **5s** in 67% yield (104.2 mg) as a white solid according to the general procedure C_2. **^{1}H NMR** (400 MHz, $CDCl_3$) δ 8.67 (s, 1H), 7.95 - 7.86 (m, 4H), 7.74 - 7.66 (m, 3H), 7.46-7.38 (m, 2H), 7.33 - 7.20 (m, 7H), 7.15 - 7.07 (m, 4H), 5.74 (d of ABq, J = 10.7, 3.4 Hz, 1H), 5.66 (d, J = 3.1 Hz, 1H), 5.47 (d of ABq, J = 10.7, 3.6 Hz, 1H), 5.11 (d, J = 3.5 Hz, 1H), 4.40-4.35 (m, 2H), 4.23-4.17 (m, 1H), 3.34 (s, 3H), 2.63-2.50 (m, 2H), 2.35 (t, J = 7.4 Hz, 2H), 1.99-1.85 (m, 2H). **^{13}C NMR** (100 MHz, $CDCl_3$) δ 172.2, 166.2, 166.1(5), 165.5, 136.4, 133.4, 133.3, 133.2(9), 130.2, 129.8, 129.7, 129.4(4), 129.4(0), 129.1, 129.0, 128.6, 128.5, 128.4(3), 128.4, 123.0, 120.9, 119.4, 111.7, 107.6, 97.5, 69.0, 68.5, 68.3, 66.6, 62.5, 55.7, 37.6, 32.4, 23.6. **IR** (film) 3408, 2980, 2904, 1720, 1450, 1404, 1258, 1069, 899, 744, 707. **HRMS** (ESI) Calcd for $C_{40}H_{37}NO_{10}S_2$ (M+Na$^+$) 778.1751, Found 778.1754.

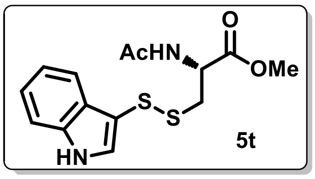

5t

The reaction of indole (0.22 mmol, 1.1 equivalents, 25.8 mg), $B(C_6F_5)_3$ (0.004 mmol, 2 mol%, 2.1 mg), RSSOMe (0.2 mmol, 1 equivalent, 47.9 mg) in toluene (0.25 mL) at r.t. for 24 hours afforded compound **5t** in 95% yield (61.5 mg) as a white solid according to the general procedure C_2. **^{1}H NMR** (400 MHz, $CDCl_3$) δ 9.11 (brs, 1H), 7.82-7.80 (m, 1H), 7.43 (m, 2H), 7.28 - 7.17 (m, 1H), 6.45 (s, 1H), 4.94 (dt, J = 7.3, 5.3 Hz, 1H), 3.73 (s, 3H), 3.32 - 3.15 (m, 2H), 1.89 (s, 3H). **^{13}C NMR** (100 MHz, $CDCl_3$) δ 171.1, 170.3, 136.6, 130.9, 128.2, 123.2, 121.1, 119.3, 111.9, 106.4, 52.7, 51.7, 39.7, 22.9. **IR** (film) 3379, 3266, 2982, 2906, 1738, 1657, 1519, 1408, 1372, 1217, 1037, 907, 831, 737. **HRMS** (EI) Calcd for $C_{14}H_{16}N_2O_3S_2$ 324.0602, Found 324.0606.

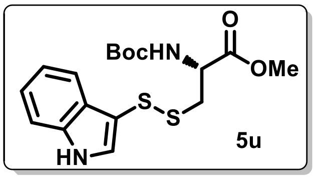

5u

The reaction of indole (0.22 mmol, 1.1 equivalents, 25.8 mg), $B(C_6F_5)_3$ (0.004 mmol, 2 mol%, 2.1 mg), RSSOMe (0.2 mmol, 1 equivalent, 59.5 mg) in toluene (0.25 mL) at 0 ^{o}C for 24 hours afforded compound **5u** in 89% yield (68 mg) as a white solid according to the general procedure C_2. **^{1}H NMR** (400 MHz, $CDCl_3$) δ 8.44 (brs, 1H), 7.71-7.69 (m, 1H), 7.33 (d, J = 1.9 Hz, 1H), 7.27-7.25 (m, 1H), 7.15 - 7.08 (m, 2H), 5.25 (d, J = 6.9 Hz, 1H), 4.58 (d, J = 6.5 Hz, 1H), 3.60 (s, 3H), 3.09-2.89 (m, 2H), 1.31 (s, 9H). **^{13}C NMR** (100 MHz, $CDCl_3$) δ 171.5, 155.2, 136.5, 130.6, 128.4, 123.3, 121.2, 119.5, 111.7, 107.0, 80.2, 52.9, 52.6, 40.2, 28.3. **IR** (film) 3380, 2974, 2900, 1736, 1650, 1407, 1253, 1067, 892, 750. **HRMS** (EI) Calcd for $C_{17}H_{22}N_2O_4S_2$ 382.1021, Found 382.1017.

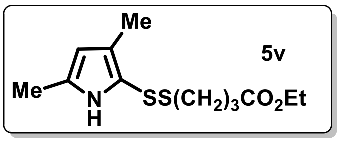

5v

The reaction of 2,4-dimethylpyrrole (0.3 mmol, 1.5 equivalents, 28.6 mg), $MeSO_3H$ (0.02 mmol, 10 mol%, 2 mg), $EtO_2C(CH_2)_3SSOMe$ (0.2 mmol, 1 equivalent, 42.1 mg) in *t*-AmylOH (0.5 mL) at 0 ^{o}C for 5 hours afforded compound **5v** in 62% yield (33.7 mg) as a colorless oil according to the general procedure C_1. **^{1}H NMR** (400 MHz, $CDCl_3$) δ 8.45 (s, 1H), 5.78 (s, 1H), 4.16 (q, J = 7.1 Hz, 2H), 2.76 (t, J = 7.2 Hz, 2H), 2.45 (t, J = 6.7 Hz, 2H), 2.33 - 1.91 (m, 8H), 1.27 (t, J = 7.0 Hz, 3H). **^{13}C NMR** (100 MHz, $CDCl_3$) δ 173.6, 131.7, 128.0, 115.3, 109.6, 60.6, 36.7, 32.5, 23.8,

14.2, 13.2, 11.8. **IR** (film) 3340, 2921, 1729, 1560, 1444, 1374, 1293, 1204, 1135, 1034, 858, 792. **HRMS** (EI) Calcd for $C_{12}H_{19}NO_2S_2$ 273.0857, Found 273.0863.

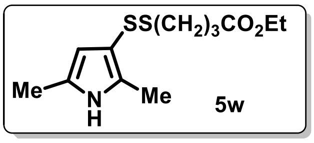

The reaction of 2,5-dimethylpyrrole (0.3 mmol, 1.5 equivalents, 28.6 mg), $MeSO_3H$ (0.02 mmol, 10 mol%, 2 mg), $EtO_2C(CH_2)_3SSOMe$ (0.2 mmol, 1 equivalent, 42.1 mg) in *t*-AmylOH (0.5 mL) at 0 ^oC for 5 hours afforded compound **5w** in 80% yield (43.7 mg) as a colorless oil according to the general procedure C_1. **^{1}H NMR** (400 MHz, $CDCl_3$) δ 7.78 (s, 1H), 5.92 - 5.83 (m, 1H), 4.13 (q, J = 7.1 Hz, 2H), 2.77 (t, J = 7.0 Hz, 2H), 2.42 (t, J = 7.3 Hz, 2H), 2.32 (s, 3H), 2.19 (s, 3H), 2.09 (p, J = 7.2 Hz, 2H), 1.26 (t, J = 7.1 Hz, 3H). **^{13}C NMR** (100 MHz, $CDCl_3$) δ 173.2, 131.7, 126.3, 111.0, 110.9, 60.3, 37.4, 32.9, 23.9, 14.2, 12.9, 11.4. **IR** (film) 3358, 2922, 1713, 1587, 1444, 1373, 1309, 1206, 1181, 1131, 1034, 859, 785, 647. **HRMS** (EI) Calcd for $C_{15}H_{23}NO_4S_2$ 273.0857, Found 273.0856.

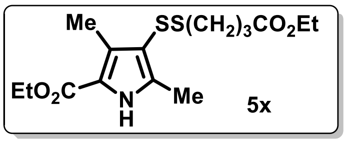

The reaction of ethyl 3,5-dimethyl-1H-pyrrole-2-carboxylate (0.3 mmol, 1.5 equivalents, 50.2 mg), $MeSO_3H$ (0.02 mmol, 10 mol%, 2 mg), $EtO_2C(CH_2)_3SSOMe$ (0.2 mmol, 1 equivalent, 42.1 mg) in *t*-AmylOH (0.5 mL) at 0 ^oC for 24 hours afforded compound **5x** in 41% yield (28.3 mg) as a white solid according to the general procedure C_1. **^{1}H NMR** (400 MHz, $CDCl_3$) δ 9.41 (s, 1H), 4.32 (q, J = 6.9 Hz, 2H), 4.12 (q, J = 7.0 Hz, 2H), 2.72 (t, J = 6.9 Hz, 2H), 2.51 - 2.29 (m, 8H), 2.19 - 1.94 (m, 2H), 1.36 (t, J = 7.0 Hz, 3H), 1.24 (t, J = 7.0 Hz, 3H). **^{13}C NMR** (100 MHz, $CDCl_3$) δ 173.0, 161.7, 137.9, 131.4, 117.9, 114.8, 60.4, 60.2, 37.1, 32.7, 24.0, 14.5, 14.2, 12.1, 11.5. **IR** (film) 3270, 2981, 1728, 1665, 1434, 1376, 1276, 1206, 1129, 1078, 1019, 877, 775, 624. **HRMS** (EI) Calcd for $C_{15}H_{23}NO_4S_2$ 345.1069, Found 345.1073.

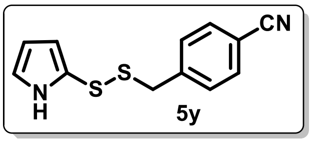

The reaction of pyrrole (0.22 mmol, 1.1 equivalents, 14.8 mg), $B(C_6F_5)_3$ (0.004 mmol, 2 mol%, 2.1 mg), 4-NCC_6H_4SSOMe (0.2 mmol, 1 equivalent, 42.3 mg) in toluene (0.25 mL) at 0 ^oC for 24 hours afforded compound **5y** in 67% yield (33.0 mg) as a white solid according to the general procedure C_2. **^{1}H NMR** (400 MHz, $CDCl_3$) δ

8.21 (brs, 1H), 7.61 (d, J = 8.2 Hz, 2H), 7.41 (d, J = 8.2 Hz, 2H), 6.85 (dd, J = 4.0, 2.7 Hz, 1H), 6.35 (t, J = 3.5 Hz, 1H), 6.17 (dd, J = 5.9, 2.9 Hz, 1H), 3.98 (s, 2H). **^{13}C NMR** (100 MHz, $CDCl_3$) δ 142.9, 132.2, 130.1, 122.6, 119.3, 118.7, 117.8, 111.1, 110.2, 42.2. **IR** (film) 3448, 2979, 2908, 2231, 1404, 1252, 1049, 873, 730, 695. **HRMS** (EI) Calcd for $C_{12}H_{10}N_2S_2$ 246.0285, Found 246.0283.

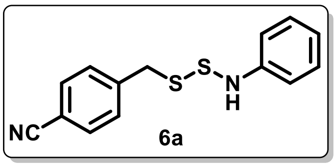

6a

The reaction of aniline (0.22 mmol, 1.1 equivalents, 20.5 mg), $B(C_6F_5)_3$ (0.005 mmol, 2.5 mol%, 2.6 mg), 4-$NCC_6H_4CH_2SSOMe$ (0.2 mmol, 1 equivalent, 42.3 mg) in toluene (0.5 mL) at r.t. for 24 hours afforded compound **6a** in 86% yield (46.9 mg) as a white solid according to the general procedure D. **^{1}H NMR** (400 MHz, $CDCl_3$) δ 7.60 (d, J = 8.1 Hz, 2H), 7.39 (d, J = 8.1 Hz, 2H), 7.25 (t, J = 7.8 Hz, 2H), 7.05 - 6.81 (m, 3H), 4.72 (s, 1H), 4.08 (s, 2H). **^{13}C NMR** (100 MHz, $CDCl_3$) δ 145.0, 143.7, 132.3, 129.8, 129.2, 121.9, 118.6, 116.9, 111.1, 42.9. **IR** (film) 3314, 2975, 2227, 1595, 1491, 1397,1285, 1225, 1073, 890, 841, 744, 686. **HRMS** (EI) Calcd for $C_{14}H_{12}N_2S_2$ 272.0442, Found 272.0447.

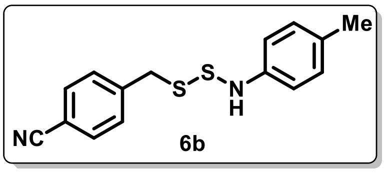

6b

The reaction of 4-Methylaniline (0.22 mmol, 1.1 equivalents, 23.6 mg), $B(C_6F_5)_3$ (0.005 mmol, 2.5 mol%, 2.6 mg), 4-$NCC_6H_4CH_2SSOMe$ (0.2 mmol, 1 equivalent, 42.3 mg) in toluene (0.5 mL) at r.t. for 24 hours afforded compound **6b** in 93% yield (53.3 mg) as a white solid according to the general procedure D. **^{1}H NMR** (400 MHz, DMSO-d_6) δ 7.90 (s, 1H), 7.79 (d, J = 8.1 Hz, 2H), 7.53 (d, J = 8.1 Hz, 3H), 7.05 (d, J = 8.2 Hz, 2H), 6.96 (d, J = 8.3 Hz, 2H), 4.17 (s, 2H), 2.36 (s, 3H). **^{13}C NMR** (100 MHz, DMSO-d_6) δ 144.1, 143.3, 132.3, 130.0, 129.5, 118.8, 116.5, 109.8, 40.6, 20.2. **IR** (film) 3311, 2922, 2228, 1603, 1504, 1458, 1283, 1224, 896, 841, 809, 740, 646. **HRMS** (EI) Calcd for $C_{15}H_{14}N_2S_2$ 286.0596, Found 286.0593.

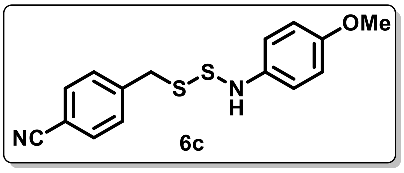

6c

The reaction of 4-Methoxyaniline (0.22 mmol, 1.1 equivalents, 27.1 mg), $B(C_6F_5)_3$ (0.005 mmol, 2.5

mol%, 2.6 mg), 4-$NCC_6H_4CH_2SSOMe$ (0.2 mmol, 1 equivalent, 42.3 mg) in toluene (0.5 mL) at r.t. for 24 hours afforded compound **6c** in 82% yield (49.9 mg) as a white solid according to the general procedure D. **^{1}H NMR** (400 MHz, DMSO-d_6) δ 7.79 (d, J = 8.2 Hz, 2H), 7.75 (s, 1H), 7.53 (d, J = 8.2 Hz, 2H), 7.00 (d, J = 8.9 Hz, 2H), 6.85 (d, J = 8.9 Hz, 2H), 4.15 (s, 2H), 3.70 (s, 3H). **^{13}C NMR** (100 MHz, DMSO-d_6) δ 154.0, 144.2, 139.1, 132.3, 130.0, 118.8, 117.9, 114.5, 109.7, 55.2, 40.6. **IR** (film) 3314, 2949, 2834, 2228, 1605, 1504, 1463, 1280, 1222, 1029, 900, 824, 753, 655. **HRMS** (EI) Calcd for $C_{15}H_{14}ON_2S_2$ 302.0548, Found 302.0554.

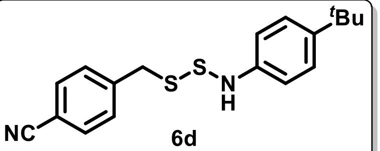
6d

The reaction of 4-*tert*-butylaniline (0.22 mmol, 1.1 equivalents, 32.9 mg), $B(C_6F_5)_3$ (0.005 mmol, 2.5 mol%, 2.6 mg), 4-$NCC_6H_4CH_2SSOMe$ (0.2 mmol, 1 equivalent, 42.3 mg) in toluene (0.5 mL) at r.t. for 24 hours afforded compound **6d** in 92% yield (60.9 mg) as a white solid according to the general procedure D. **^{1}H NMR** (400 MHz, DMSO-d_6) δ 7.94 (s, 1H), 7.78 (d, J = 8.2 Hz, 2H), 7.54 (d, J = 8.3 Hz, 2H), 7.26 (d, J = 8.7 Hz, 2H), 7.00 (d, J = 8.6 Hz, 2H), 4.17 (s, 2H), 1.23 (s, 9H). **^{13}C NMR** (100 MHz, DMSO-d_6) δ 144.1, 143.2, 143.0, 132.2, 130.0, 125.7, 118.8, 116.1, 109.8, 40.6, 33.7, 31.3. **IR** (film) 3332, 2956, 2852, 2227, 1607, 1510, 1464, 1362, 1282, 1233, 1285, 998, 826, 733, 653. **HRMS** (EI) Calcd for $C_{18}H_{20}N_2S_2$ 328.1070, Found 328.1068.

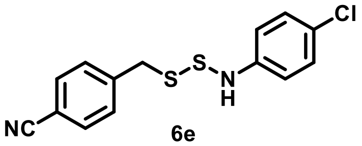
6e

The reaction of 4-chloroaniline (0.22 mmol, 1.1 equivalents, 28.1 mg), $B(C_6F_5)_3$ (0.005 mmol, 2.5 mol%, 2.6 mg), 4-$NCC_6H_4CH_2SSOMe$ (0.2 mmol, 1 equivalent, 42.3 mg) in toluene (0.5 mL) at r.t. for 24 hours afforded compound **6e** in 87% yield (54 mg) as a white solid according to the general procedure D. **^{1}H NMR** (400 MHz, DMSO-d_6) δ 8.20 (s, 1H), 7.76 (s, 2H), 7.53 (s, 2H), 7.25 (s, 2H), 7.05 (s, 2H), 4.18 (s, 2H). **^{13}C NMR** (100 MHz, DMSO-d_6) δ 144.9, 143.9, 132.3, 130.1, 128.9, 124.3, 118.8, 117.8, 109.9, 40.7. **IR** (film) 3305, 2962, 2852, 2230, 1597, 1486, 1262, 1225, 1093, 894, 814, 737. **HRMS** (EI) Calcd for $C_{14}H_{11}ClN_2S_2$ 306.0051, Found 306.0052.

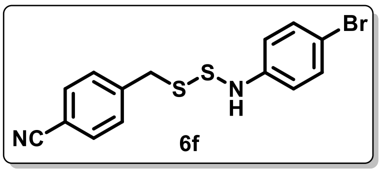

The reaction of 4-bromoaniline (0.22 mmol, 1.1 equivalents, 37.9 mg), $B(C_6F_5)_3$ (0.005 mmol, 2.5 mol%, 2.6 mg), $4\text{-}NCC_6H_4CH_2SSOMe$ (0.2 mmol, 1 equivalent, 42.3 mg) in toluene (0.5 mL) at r.t. for 24 hours afforded compound **6f** in 71% yield (50 mg) as a white solid according to the general procedure D. **^{1}H NMR** (400 MHz, DMSO-d_6) δ 8.20 (s, 1H), 7.79 (d, *J* = 8.1 Hz, 2H), 7.54 (d, *J* = 8.1 Hz, 2H), 7.40 (d, *J* = 8.7 Hz, 2H), 7.01 (d, *J* = 8.8 Hz, 2H), 4.20 (s, 2H). **^{13}C NMR** (100 MHz, DMSO) δ 145.3, 143.9, 132.3, 131.8, 130.1, 118.8, 118.3, 112.0, 109.9, 40.7. **IR** (film) 3300, 2960, 2850, 2231, 1587, 1482, 1226, 1072, 894, 814. **HRMS** (EI) Calcd for $C_{14}H_{11}BrN_2S_2$ 349.9547, Found 349.9556.

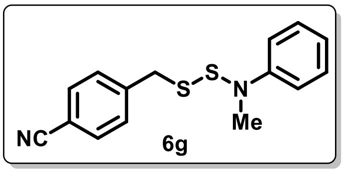

The reaction of *N*-methylaniline (0.22 mmol, 1.1 equivalents, 23.6 mg), $B(C_6F_5)_3$ (0.005 mmol, 2.5 mol%, 2.6 mg), $4\text{-}NCC_6H_4CH_2SSOMe$ (0.2 mmol, 1 equivalent, 42.3 mg) in toluene (0.5 mL) at r.t. for 24 hours afforded compound **6g** in 60% yield (35.3 mg) as a white solid according to the general procedure D. **^{1}H NMR** (400 MHz, DMSO-d_6) δ 7.76 (d, *J* = 8.3 Hz, 2H), 7.42 (d, *J* = 8.3 Hz, 2H), 7.36 - 7.25 (m, 2H), 7.26 - 7.17 (m, 2H), 7.09 - 6.83 (m, 1H), 4.11 (s, 2H), 3.15 (s, 3H). **^{13}C NMR** (100 MHz, DMSO-d_6) δ 149.2, 144.0, 132.4, 129.9, 128.9, 121.7, 118.7, 118.4, 109.9, 43.9, 42.3. **IR** (film) 2990, 2956, 2227, 1933, 1595, 1489, 1413, 1253, 1082, 1062, 849, 747, 683. **HRMS** (EI) Calcd for $C_{15}H_{14}N_2S_2$ 286.0596, Found 286.0598.

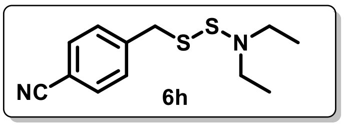

The reaction of diethylamine (0.22 mmol, 1.1 equivalents, 17.6 mg), $B(C_6F_5)_3$ (0.005 mmol, 2.5 mol%, 2.6 mg), $4\text{-}NCC_6H_4CH_2SSOMe$ (0.2 mmol, 1 equivalent, 42.3 mg) in toluene (0.5 mL) at r.t. for 24 hours afforded compound **6h** in 69% yield (34.7 mg) as a colorless oil according to the general procedure D. **^{1}H NMR** (400 MHz, $CDCl_3$) δ 7.61 (d, *J* = 8.3 Hz, 2H), 7.42 (d, *J* = 8.4 Hz, 2H), 4.09 (s, 2H), 2.82 (q, *J* = 7.1 Hz, 4H), 1.15 (t, *J* = 7.1 Hz, 6H). **^{13}C NMR** (100 MHz, $CDCl_3$) δ 144.3, 132.4, 129.5, 118.7, 111.0, 51.3, 44.4, 13.3. **IR** (film) 2977, 2935, 2847, 2229, 1606, 1504, 1465, 1379, 1178, 1060, 1024, 904, 844, 841, 614. **HRMS** (EI) Calcd for $C_{12}H_{16}N_2S_2$ 252.0755, Found 252.0760.

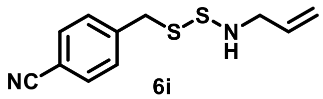

6i

The reaction of allylamine (0.22 mmol, 1.1 equivalents, 12.6 mg), $B(C_6F_5)_3$ (0.005 mmol, 2.5 mol%, 2.6 mg), 4-$NCC_6H_4CH_2SSOMe$ (0.2 mmol, 1 equivalent, 42.3 mg) in toluene (0.5 mL) at r.t. for 24 hours afforded compound **6i** in 67% yield (32.1 mg) as a white solid according to the general procedure D. **^{1}H NMR** (400 MHz, DMSO-d_6) δ 7.82 (d, J = 7.8 Hz, 2H), 7.54 (d, J = 7.7 Hz, 2H), 5.87-5.75 (m, 1H), 5.20 (d, J = 17.2 Hz, 1H), 5.13-5.11 (m, 2H), 4.15 (s, 2H), 3.43 (s, 2H). **^{13}C NMR** (100 MHz, DMSO-d_6) δ 144.5, 135.6, 132.3, 130.1, 118.8, 116.7, 109.7, 52.4, 40.6. **IR** (film) 3292, 2928, 2228, 1919, 1604, 1501, 1417, 1200, 1053, 991, 929, 839, 680. **HRMS** (EI) Calcd for $C_{11}H_{12}N_2S_2$ 236.0442, Found 236.0446.

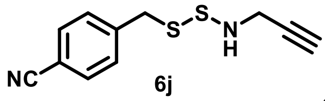

6j

The reaction of 2-propynylamine (0.22 mmol, 1.1 equivalents, 12.2 mg), $B(C_6F_5)_3$ (0.005 mmol, 2.5 mol%, 2.6 mg), 4-$NCC_6H_4CH_2SSOMe$ (0.2 mmol, 1 equivalent, 42.3 mg) in toluene (0.5 mL) at r.t. for 24 hours afforded compound **6j** in 56% yield (26.4 mg) as a white solid according to the general procedure D. **^{1}H NMR** (400 MHz, DMSO-d_6) δ 7.80 (d, J = 8.3 Hz, 2H), 7.55 (d, J = 8.3 Hz, 2H), 5.34 (t, J = 4.6 Hz, 1H), 4.17 (s, 2H), 3.60 (dd, J = 4.6, 2.5 Hz, 2H), 3.23 (t, J = 2.5 Hz, 1H). **^{13}C NMR** (100 MHz, DMSO-d_6) δ 144.3, 132.3, 130.2, 118.9, 109.7, 81.2, 74.9, 40.7, 38.6. **IR** (film) 3293, 2925, 2361, 2225, 1603, 1503, 1418, 1320, 1048, 838, 658. **HRMS** (EI) Calcd for $C_{11}H_{10}N_2S_2$ 234.0285, Found 234.0282.

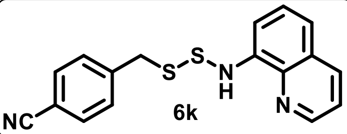

6k

The reaction of 8-aminoquinoline (0.22 mmol, 1.1 equivalents, 32.9 mg), $B(C_6F_5)_3$ (0.005 mmol, 2.5 mol%, 2.6 mg), 4-$NCC_6H_4CH_2SSOMe$ (0.2 mmol, 1 equivalent, 42.3 mg) in toluene (0.5 mL) at r.t. for 24 hours afforded compound **6k** in 70% yield (38.9 mg) as a white solid according to the general procedure D. **^{1}H NMR** (400 MHz, DMSO-d_6) δ 9.35 (dd, J = 4.2, 1.7 Hz, 1H), 8.90 (s, 1H), 8.85 (dd, J = 8.3, 1.6 Hz, 1H), 8.29 (d, J = 8.3 Hz, 2H), 8.16 (d, J = 8.3 Hz, 2H), 8.10 (dd, J = 8.3, 4.2 Hz, 1H), 8.08-8.04 (m, 1H), 7.99 (dd, J = 7.6, 1.3 Hz, 1H), 7.95 (dd, J = 8.0, 1.3 Hz, 1H), 4.79 (s, 2H). **^{13}C NMR** (100 MHz, DMSO-d_6) δ 148.2, 144.8, 141.7, 139.1, 136.3, 132.3, 130.2, 128.3, 127.1, 122.1, 119.0, 118.8, 111.3, 109.8, 40.5. **IR** (film)

3318, 3045, 2221, 1610, 1464, 1407, 1372, 1308, 1083, 908, 840, 820, 745, 628. **HRMS** (EI) Calcd for $C_{17}H_{13}N_3S_2$ 323.0551, Found 323.0560.

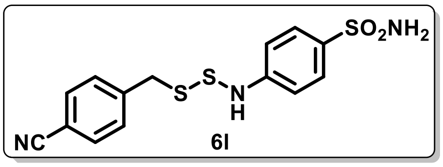

The reaction of sulfanilamide (0.22 mmol, 1.1 equivalents, 41.1 mg), $B(C_6F_5)_3$ (0.005 mmol, 2.5 mol%, 2.6 mg), 4-$NCC_6H_4CH_2SSOMe$ (0.2 mmol, 1 equivalent, 42.3 mg) in CH_3CN (0.5 mL) at r.t. for 24 hours afforded compound **6l** in 80% yield (56 mg) as a white solid according to the general procedure D. **^{1}H NMR** (400 MHz, DMSO-d$_6$) δ 8.55 (s, 1H), 7.82 (d, J = 8.1 Hz, 2H), 7.71 (d, J = 8.7 Hz, 2H), 7.57 (d, J = 8.2 Hz, 2H), 7.32 - 7.03 (m, 4H), 4.23 (s, 2H). **^{13}C NMR** (100 MHz, DMSO-d$_6$) δ 149.1, 143.8, 135.8, 132.4, 130.1, 127.3, 118.8, 115.7, 109.9, 40.8. **IR** (film) 3292, 2918, 2232, 1593, 1496, 1334, 1246, 1152, 1093, 890, 824, 771, 649. **HRMS** (EI) Calcd for $C_{14}H_{13}N_3O_2S_3$ 351.0170, Found 351.0168.

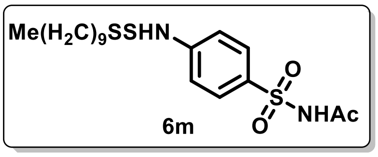

The reaction of acetosulfamine (0.2 mmol, 1 equivalent, 42.9 mg), $B(C_6F_5)_3$ (0.005 mmol, 2.5 mol%, 2.6 mg), $Me(CH_2)_9SSOMe$ (0.3 mmol, 1.5 equivalents, 70.9 mg) in DMF (0.5 mL) at r.t. for 24 hours afforded compound **6m** in 80% yield (67 mg) as a white solid according to the general procedure D. **^{1}H NMR** (400 MHz, DMSO-d$_6$) δ 11.84 (brs, 1H), 8.64 (s, 1H), 7.76 (d, J = 8.7 Hz, 2H), 7.17 (d, J = 8.7 Hz, 2H), 2.91 (t, J = 7.3 Hz, 2H), 1.89 (s, 3H), 1.72-1.58 (m, 2H), 1.33-1.19 (m, 14H), 0.85 (t, J = 6.5 Hz, 3H). **^{13}C NMR** (100 MHz, DMSO-d$_6$) δ 168.4, 151.2, 129.8, 129.3, 115.2, 38.1, 31.2, 29.1, 28.9, 28.6, 28.5, 27.8, 23.1, 22.0, 13.9. **IR** (film) 3329, 3240, 2966, 2921, 1698, 1590, 1490, 1449, 1254, 1152, 1080, 1048, 831, 681, 627. **HRMS** (ESI) Calcd for $C_{18}H_{30}N_2O_3S_3$ (M+Na$^+$) 441.1311, Found 441.1303.

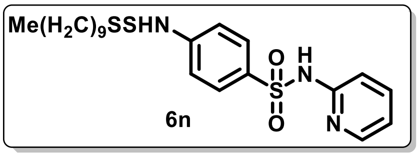

The reaction of sulfapyridine (0.2 mmol, 1 equivalent, 49.8 mg), $B(C_6F_5)_3$ (0.005 mmol, 2.5 mol%, 2.6 mg), $Me(CH_2)_9SSOMe$ (0.3 mmol, 1.5 equivalents, 70.9 mg) in DMF (0.5 mL) at r.t. for 24 hours afforded compound **6n** in 86% yield (78 mg) as a white solid according to the general procedure D. **^{1}H NMR**

(400 MHz, DMSO-d_6) δ 11.48 (brs, 1H), 8.48 (s, 1H), 8.3 (m, 1H), 7.75 (d, *J* = 8.8 Hz, 2H), 7.67 (m, 1H), 7.14-7.05 (m, 3H), 6.87 (m, 1H), 2.87 (t, *J* = 7.3 Hz, 2H), 1.67-1.56 (m, 2H), 1.31- 1.19 (m, 14H), 0.85 (t, *J* = 6.8 Hz, 3H). **^{13}C NMR** (100 MHz, DMSO-d_6) δ 152.1, 149.6, 144.3, 138.9, 131.7, 127.9, 115.8, 114.8, 112.4, 37.5, 30.7, 28.5, 28.3, 28.1, 28.0, 27.3, 21.5, 13.4. **IR** (film) 3315, 2978, 2905, 1591, 1453, 1386, 1251, 1073, 890, 767, 683. **HRMS** (ESI) Calcd for $C_{21}H_{31}N_3O_2S_3$ (M+H$^+$) 454.1651, Found 454.1642.

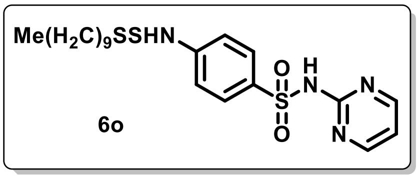

6o

The reaction of sulfadiazine (0.2 mmol, 1 equivalent, 50.1 mg), $B(C_6F_5)_3$ (0.005 mmol, 2.5 mol%, 2.6 mg), $Me(CH_2)_9SSOMe$ (0.3 mmol, 1.5 equivalents, 70.9 mg) in DMF (0.5 mL) at r.t. for 24 hours afforded compound **6o** in 60% yield (54.5 mg) as a white solid according to the general procedure D. **^{1}H NMR** (400 MHz, DMSO-d_6) δ 11.52 (brs, 1H), 8.65 - 8.38 (m, 3H), 7.84 (d, *J* = 8.7 Hz, 2H), 7.13 (d, *J* = 8.8 Hz, 2H), 7.03 (t, *J* = 4.8 Hz, 1H), 2.88 (t, *J* = 7.2 Hz, 2H), 1.72 - 1.56 (m, 2H), 1.3 -1.15 (m, 14H), 0.85 (t, *J* = 6.7 Hz, 3H). **^{13}C NMR** (100 MHz, DMSO-d_6) δ 158.3, 157.0, 150.6, 130.9, 129.4, 115.7, 115.1, 38.0, 31.3, 29.1, 28.9, 28.6, 28.5, 27.8, 22.1, 13.9. **IR** (film) 3303, 2979, 2904, 1583, 1406, 1356, 1249, 1153, 1052, 892, 801, 673. **HRMS** (ESI) Calcd for $C_{20}H_{30}N_4O_2S_3$ (M+Na$^+$) 477.1423, Found 477.1420.

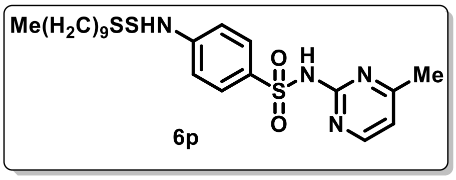

6p

The reaction of sulfamerazine (0.2 mmol, 1 equivalent, 52.9 mg), $B(C_6F_5)_3$ (0.005 mmol, 2.5 mol%, 2.6 mg), $Me(CH_2)_9SSOMe$ (0.3 mmol, 1.5 equivalents, 70.9 mg) in DMF (0.5 mL) at r.t. for 24 hours afforded compound **6p** in 90% yield (84.4 mg) as a white solid according to the general procedure D. **^{1}H NMR** (400 MHz, $CDCl_3$) δ 8.54 (d, *J* = 5.2 Hz, 1H), 8.00 (d, *J* = 8.7 Hz, 2H), 7.09 (d, *J* = 8.8 Hz, 2H), 6.78 (d, *J* = 5.2 Hz, 1H), 5.64 (s, 1H), 2.87 (t, *J* = 7.3 Hz, 2H), 2.42 (s, 3H), 1.75-1.59 (m, 2H), 1.38-1.21 (m, 14H), 0.87 (t, *J* = 6.8 Hz, 3H). **^{13}C NMR** (100 MHz, $CDCl_3$) δ 169.5, 157.3, 156.5, 150.2, 131.2, 130.6, 115.1, 115.1, 39.4, 31.8, 29.7, 29.5, 29.4, 29.2, 29.1, 28.4, 24.0, 22.6, 14.1. **IR** (film) 3314, 2981, 2903, 1593, 1404, 1250, 1070, 892, 745. **HRMS** (ESI) Calcd for $C_{21}H_{32}N_4O_2S_3$ (M+Na$^+$) 491.1580, Found 491.1577.

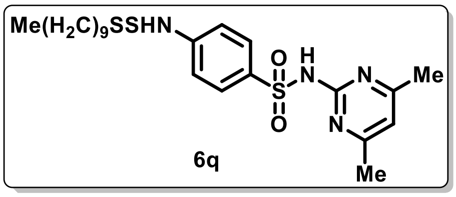

6q

The reaction of sulfamethazine (0.2 mmol, 1 equivalent, 56.7 mg), $B(C_6F_5)_3$ (0.005 mmol, 2.5 mol%, 2.6 mg), $Me(CH_2)_9SSOMe$ (0.3 mmol, 1.5 equivalents, 70.9 mg) in DMF (0.5 mL) at r.t. for 24 hours afforded compound **6q** in 81% yield (78.1 mg) as a white solid according to the general procedure D. **^{1}H NMR** (400 MHz, DMSO-d$_6$) δ 11.38 (brs, 1H), 8.49 (s, 1H), 7.86 (d, J = 8.8 Hz, 2H), 7.12 (d, J = 8.8 Hz, 2H), 6.74 (s, 1H), 2.88 (t, J = 7.3 Hz, 2H), 2.24 (s, 6H), 1.66-1.59 (m, 2H), 1.30-1.16 (m, 14H), 0.85 (t, J = 6.8 Hz, 3H). **^{13}C NMR** (100 MHz, DMSO-d$_6$) δ 167.3, 156.3, 150.3, 131.2, 129.8, 114.7, 113.7, 38.1, 31.2, 29.0, 28.8, 28.6, 28.4, 27.7, 22.9, 22.0, 13.9. **IR** (film) 3316, 2977, 2903, 1593, 1489, 1384, 1249, 1151, 1075, 872, 832, 676. **HRMS** (ESI) Calcd for $C_{22}H_{34}N_4O_2S_3$ (M+H$^+$)483.1917, Found 483.1913.

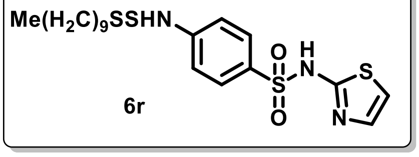

6r

The reaction of sulfathiazole (0.2 mmol, 1 equivalent, 51.1 mg), $B(C_6F_5)_3$ (0.005 mmol, 2.5 mol%, 2.6 mg), $Me(CH_2)_9SSOMe$ (0.3 mmol, 1.5 equivalents, 70.9 mg) in DMF (0.5 mL) at r.t. for 24 hours afforded compound **6r** in 65% yield (59.8 mg) as a white solid according to the general procedure D. **^{1}H NMR** (400 MHz, DMSO-d$_6$) δ 12.57 (brs, 1H), 8.43 (s, 1H), 7.66 (d, J = 8.7 Hz, 2H), 7.21 (d, J = 4.6 Hz, 1H), 7.14 (d, J = 8.7 Hz, 2H), 6.78 (d, J = 4.6 Hz, 1H), 2.88 (t, J = 7.3 Hz, 2H), 1.68-1.61 (m, 2H), 1.31-1.17 (m, 14H), 0.85 (t, J = 6.7 Hz, 3H). **^{13}C NMR** (100 MHz, DMSO-d$_6$) δ 168.4, 149.7, 133.5, 127.4, 124.2, 115.3, 38.0, 31.2, 29.1, 28.9, 28.6, 28.5, 27.8, 22.0, 13.9. **IR** (film) 3319, 2953, 2923, 2853, 1573, 1536, 1490, 1289, 1138, 1186, 930, 856, 750, 683, 637. **HRMS** (ESI) Calcd for $C_{19}H_{29}N_3O_2S_4$ (M+Na$^+$) 482.1035, Found 482.1034.

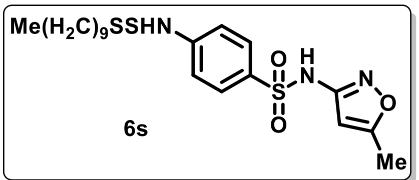

6s

The reaction of sulfamethoxazole (0.2 mmol, 1 equivalent, 50.7 mg), $B(C_6F_5)_3$ (0.005 mmol, 2.5 mol%, 2.6 mg), $Me(CH_2)_9SSOMe$ (0.3 mmol, 1.5 equivalents, 70.9 mg) in DMF (0.5 mL) at r.t. for 24 hours afforded compound **6s** in 86% yield (78.7 mg) as a white solid according to the general procedure D. **^{1}H NMR** (400 MHz, DMSO-d$_6$) δ 11.17 (s, 1H), 8.60 (s, 1H), 7.70 (d, J = 8.8 Hz, 2H), 7.15 (d, J = 8.8 Hz, 2H), 6.11 (s, 1H), 2.89 (t, J = 7.3

Hz, 2H), 2.29 (s, 3H), 1.73-1.58 (m, 2H), 1.32-1.15 (m, 14H), 0.85 (t, *J* = 6.7 Hz, 3H). **^{13}C NMR** (100 MHz, DMSO-d$_6$) δ 167.0, 157.6, 150.9, 130.1, 128.4, 115.5, 95.3, 38.0, 31.2, 29.0, 28.8, 28.6, 28.5, 27.8, 22.0, 13.9, 11.9(8), 11.9(5). **IR** (film) 3339, 3284, 2978, 2918, 1590, 1463, 1376, 1256, 1155, 1054, 887, 823, 682. **HRMS** (ESI) Calcd for $C_{19}H_{29}N_3O_2S_4$ (M+Na$^+$) 480.1420, Found 480.1416.

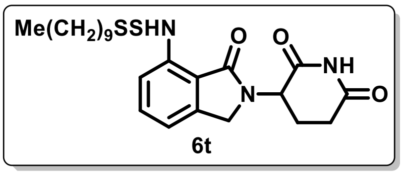

The reaction of lenalidomide (0.2 mmol, 1 equivalent, 51.9 mg), $B(C_6F_5)_3$ (0.005 mmol, 2.5 mol%, 2.6 mg), $Me(CH_2)_9SSOMe$ (0.3 mmol, 1.5 equivalents, 70.9 mg) in DMF (0.5 mL) at r.t. for 24 hours afforded compound **6t** in 62% yield (57.5 mg) as a white solid according to the general procedure D. **^{1}H NMR** (400 MHz, CDCl$_3$) δ 11.02 (s, 1H), 8.01 (s, 1H), 7.44-7.42 (m, 2H), 7.27 (d, *J* = 6.0 Hz, 1H), 5.13 (dd, *J* = 12.7, 4.1 Hz, 1H), 4.33 (q, *J* = 17.3 Hz, 2H), 2.96-2.88 (m, 3H), 2.64-2.59 (m, 1H), 2.38-2.29 (m, 1H), 2.05-2.04 (m, 1H), 1.62-1.60 (m, 2H), 1.35-1.20 (m, 14H), 0.84 (t, *J* = 6.4 Hz, 3H). **^{13}C NMR** (100 MHz, CDCl$_3$) δ 172.8, 171.0, 167.9, 141.5, 132.9, 129.8, 129.0, 118.6, 115.3, 51.6, 46.2, 37.8, 31.3, 31.2, 29.2, 28.9, 28.6, 28.5, 27.8, 22.7, 22.1, 13.9. **IR** (film) 3287, 2982, 2920, 1703, 1669, 1600, 1406, 1237, 1050, 865, 748, 658. **HRMS** (EI) Calcd for $C_{23}H_{33}N_3O_3S_2$ 463.1963, Found 463.1959.

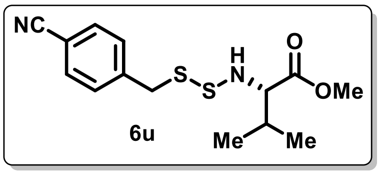

The reaction of L-valine (0.22 mmol, 1 equivalent, 25.8 mg), $B(C_6F_5)_3$ (0.005 mmol, 2.5 mol%, 2.6 mg), 4-$NCC_6H_4CH_2SSOMe$ (0.2 mmol, 1 equivalent, 42.3 mg) in toluene (0.5 mL) at r.t. for 24 hours afforded compound **6u** in 45% yield (27.9 mg) as a colorless oil according to the general procedure D. **^{1}H NMR** (400 MHz, CDCl$_3$) δ 7.61 (d, *J* = 8.1 Hz, 2H), 7.43 (d, *J* = 8.1 Hz, 2H), 4.01 (s, 2H), 3.73 (s, 3H), 3.42 (d, *J* = 8.2 Hz, 1H), 3.27 (dd, *J* = 8.2, 5.9 Hz, 1H), 1.89 (dq, *J* = 13.3, 6.7 Hz, 1H), 0.89 (d, *J* = 6.8 Hz, 3H), 0.86 (d, *J* = 6.8 Hz, 3H). **^{13}C NMR** (100 MHz, CDCl$_3$) δ 174.4, 143.2, 132.3, 129.9, 118.6, 111.1, 70.6, 52.1, 42.2, 31.9, 18.8, 18.1. **IR** (film)3317, 2940, 2229, 1732, 1606, 1505, 1437, 1301, 1201, 1140, 994, 845, 743, 648. **HRMS** (EI) Calcd for $C_{14}H_{18}N_2O_2S_2$ 310.0810, Found 310.0815.

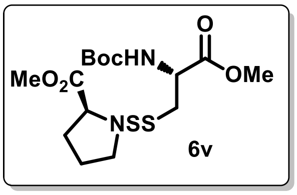

The reaction of methyl L-prolinate (0.22 mmol, 1.1 equivalents, 28.4 mg), $B(C_6F_5)_3$ (0.005 mmol, 2.5 mol%, 2.6 mg), RSSOMe (0.2 mmol, 1 equivalent, 59.5 mg) in toluene (0.5 mL) at r.t. for 24 hours afforded compound **6v** in 82% yield (64.6 mg) as a white solid according to the general procedure D. **^{1}H NMR** (400 MHz, $CDCl_3$) δ 5.35 (d, J = 7.5 Hz, 1H), 4.57 (s, 1H), 3.87-3.59 (m, 7H), 3.34 (s, 2H), 3.27 (d, J = 4.6 Hz, 1H), 3.06 (q, J = 7.9 Hz, 1H), 2.25-2.04 (m, 1H), 1.90-1.85 (m, 3H), 1.41 (s, 9H). **^{13}C NMR** (100 MHz, $CDCl_3$) δ 172.8, 170.9, 154.9, 80.1, 65.8, 55.7, 53.6, 52.5, 52.1, 43.1, 30.5, 28.2, 24.5. **IR** (film) 3380, 2980, 2905, 1743, 1714, 1403, 1252, 1164, 1074, 1048, 869, 754. **HRMS** (EI) Calcd for $C_{15}H_{26}N_2O_6S_2$ 394.1232, Found 394.1234.

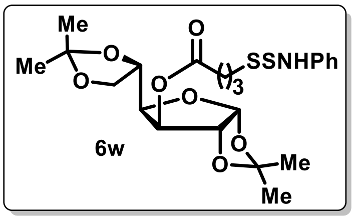

The reaction of aniline (0.22 mmol, 1.1 equivalents, 20.5 mg), $B(C_6F_5)_3$ (0.005 mmol, 2.5 mol%, 2.6 mg), RSSOMe (0.2 mmol, 1 equivalent, 84.9 mg) in toluene (0.5 mL) at r.t. for 24 hours afforded compound **6w** in 77% yield (74.7 mg) as a colorless oil according to the general procedure D. **^{1}H NMR** (400 MHz, $CDCl_3$) δ 7.25 (t, J = 7.9 Hz, 2H), 7.06 (d, J = 7.7 Hz, 2H), 6.93 (t, J = 7.3 Hz, 1H), 5.87 (d, J = 3.7 Hz, 1H), 5.47 (s, 1H), 5.29 (s, 1H), 4.47 (d, J = 3.7 Hz, 1H), 4.20 (t, J = 2.8 Hz, 2H), 4.13-4.05 (m, 1H), 4.05-3.97 (m, 1H), 2.88 (t, J = 7.0 Hz, 2H), 2.44 (t, J = 7.1 Hz, 2H), 2.03 (p, J = 7.2 Hz, 2H), 1.52 (s, 3H), 1.40 (s, 3H), 1.31 (s, 6H). **^{13}C NMR** (100 MHz, $CDCl_3$) δ 171.6, 145.4, 129.1, 121.5, 116.6, 112.2, 109.3, 105.0, 83.3, 79.8, 76.1, 72.4, 67.3, 37.9, 32.3, 26.8, 26.6, 26.1, 25.2, 24.7. **IR** (film)3334, 2981, 2903, 1742, 1598, 1490, 1378, 1222, 1070, 889, 752, 693. **HRMS** (EI) Calcd for $C_{22}H_{31}NO_7S_2$ 485.1542, Found 485.1540.

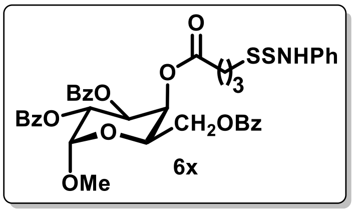

The reaction of aniline (0.22 mmol, 1.1 equivalents, 20.5 mg), $B(C_6F_5)_3$ (0.005 mmol, 2.5 mol%, 2.6 mg), RSSOMe (0.2 mmol, 1 equivalent, 134.2 mg) in toluene (0.5 mL) at r.t. for 24 hours afforded compound **6x** in 92% yield (134.4 mg) as a white solid according to the general procedure D. **^{1}H NMR** (400 MHz, $CDCl_3$) δ 8.07 (d, J = 7.8 Hz, 2H), 8.03 (d, J = 7.8 Hz, 2H), 7.91 (d, J = 7.9 Hz, 2H), 7.62-7.43 (m, 5H), 7.39 (t, J = 7.7

Hz, 2H), 7.34 (t, *J* = 7.7 Hz, 2H), 7.23 (t, *J* = 7.8 Hz, 2H), 7.05 (d, *J* = 8.3 Hz, 2H), 6.92 (t, *J* = 7.3 Hz, 1H), 5.98-5.82 (m, 2H), 5.71-5.67 (m, 2H), 5.26 (d, *J* = 3.5 Hz, 1H), 4.63-4.49 (m, 2H), 4.44-5.38 (m, 1H), 3.50 (s, 3H), 2.84 (t, *J* = 7.6 Hz, 2H), 2.55 (t, *J* = 7.0 Hz, 2H), 2.01 (p, *J* = 7.1 Hz, 2H). **^{13}C NMR** (100 MHz, $CDCl_3$) δ 172.0, 166.0, 165.9(7), 165.4, 145.4, 133.3, 133.2, 129.8, 129.6, 129.4, 129.3, 129.1, 128.4, 128.3(5), 121.3, 116.7, 97.6, 68.8, 68.5, 68.4, 66.4, 62.1, 55.6, 37.8, 32.3, 25.0. **IR** (film) 3340, 2970, 2904, 1721, 1597, 1403, 1261, 1072, 891, 753, 709. **HRMS** (ESI) Calcd for $C_{38}H_{37}NO_{10}S2$ ($M+Na^+$) 754.1751, Found 754.1732.

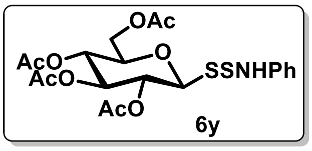

The reaction of aniline (0.22 mmol, 1.1 equivalents, 20.5 mg), $B(C_6F_5)_3$ (0.005 mmol, 2.5 mol%, 2.6 mg), RSSOMe (0.2 mmol, 1 equivalent, 85.3 mg) in toluene (0.5 mL) at r.t. for 24 hours afforded compound **6y** in 95% yield (92.5 mg) as a white solid according to the general procedure D. **^{1}H NMR** (400 MHz, $CDCl_3$) δ 7.23 (t, *J* = 7.9 Hz, 2H), 7.03 (d, *J* = 7.7 Hz, 2H), 6.92 (t, *J* = 7.3 Hz, 1H), 5.67 (s, 1H), 5.42 (t, *J* = 9.5 Hz, 1H), 5.24 (t, *J* = 9.4 Hz, 1H), 5.13 (t, *J* = 9.7 Hz, 1H), 4.68 (d, *J* = 9.7 Hz, 1H), 4.26-4.14 (m, 2H), 3.79-3.66 (m, 1H), 2.09 (s, 3H), 2.03 (s, 3H), 2.00 (s, 3H), 1.95 (s, 3H). **^{13}C NMR** (100 MHz, $CDCl_3$) δ 170.6, 170.0, 169.3, 169.2, 145.2, 129.0, 121.7, 116.8, 85.5, 76.2, 73.7, 69.5, 68.0, 61.7, 20.7, 20.6, 20.5, 20.4. **IR** (film) 3338, 2995, 1744, 1597, 1375, 1222, 1045, 912, 760, 690, 630. **HRMS** (EI) Calcd for $C_{20}H_{25}NO_9S_2$ 487.0971, Found 487.0978.

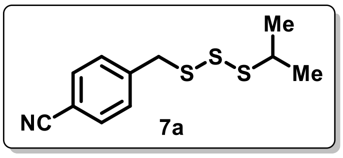

The reaction of 2-propanethiol (0.22 mmol, 1.1 equivalents, 20.5 uL), $B(C_6F_5)_3$ (0.005 mmol, 2.5 mol%, 2.6 mg), 4-$NCC_6H_4CH_2SSOMe$ (0.2 mmol, 1 equivalent, 42.3 mg) in DCM (2 mL) at r.t. for 5 hours afforded compound **7a** in 80% yield (40.9 mg) as a colorless oil according to the general procedure E. **^{1}H NMR** (400 MHz, $CDCl_3$) δ 7.62 (d, *J* = 8.3 Hz, 2H), 7.43 (d, *J* = 8.3 Hz, 2H), 4.07 (s, 2H), 3.15 (tt, *J* = 6.8 Hz, *J* = 6.8 Hz, 1H), 1.33 (d, *J* = 6.8 Hz, 6H). **^{13}C NMR** (100 MHz, $CDCl_3$) δ 142.3, 132.3, 130.1, 118.7, 111.2, 42.2, 41.8, 22.4. **IR** (film) 2962, 2922, 2862, 2228, 1919, 1606, 1503, 1446, 1235, 1153, 1047 , 875, 842, 741, 652. **HRMS** (EI) Calcd for $C_{11}H_{13}NS_3$ 255.0210, Found 255.0213.

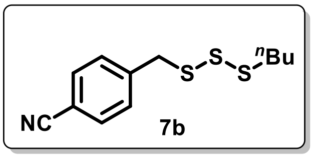

The reaction of butanethiol (0.22 mmol, 1.1 equivalents, 23.5 uL), $B(C_6F_5)_3$ (0.005 mmol, 2.5 mol%, 2.6 mg), 4-$NCC_6H_4CH_2SSOMe$ (0.2 mmol, 1 equivalent, 42.3 mg) in DCM (2 mL) at r.t. for 5 hours afforded compound **7b** in 88% yield (47.2 mg) as a colorless oil according to the general procedure E. **^{1}H NMR** (400 MHz, $CDCl_3$) δ 7.62 (d, J = 8.1 Hz, 2H), 7.43 (d, J = 8.1 Hz, 2H), 4.07 (s, 2H), 2.81 (t, J = 7.3 Hz, 2H), 1.84-1.55 (m, 2H), 1.57-1.30 (m, 2H), 0.92 (t, J = 7.4 Hz, 3H). **^{13}C NMR** (100 MHz, $CDCl_3$) δ 142.3, 132.3, 130.1, 118.7, 111.3, 42.1, 38.5, 30.7, 21.5, 13.6. **IR** (film) 2957, 2927, 2867, 2228, 1919, 1606, 1504, 1460, 1380, 1222, 1101, 1047 , 875, 842, 740, 653. **HRMS** (EI) Calcd for $C_{12}H_{15}NS_3$ 269.0367, Found 269.0371.

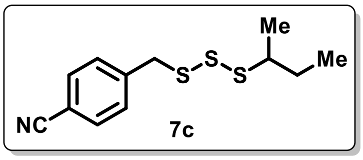

The reaction of butane-2-thiol (0.22 mmol, 1.1 equivalents, 24 uL), $B(C_6F_5)_3$ (0.005 mmol, 2.5 mol%, 2.6 mg), 4-$NCC_6H_4CH_2SSOMe$ (0.2 mmol, 1 equivalent, 42.3 mg) in DCM (2 mL) at r.t. for 5 hours afforded compound **7c** in 88% yield (47.5 mg) as a colorless oil according to the general procedure E. **^{1}H NMR** (400 MHz, $CDCl_3$) δ 7.62 (d, J = 8.3 Hz, 2H), 7.44 (d, J = 8.3 Hz, 2H), 4.07 (s, 2H), 2.94-2.89 (m, 1H), 1.82-1.66 (m, 1H), 1.63-1.50 (m, 1H), 1.33 (d, J = 6.8 Hz, 3H), 0.96 (t, J = 7.4 Hz, 3H). **^{13}C NMR** (100 MHz, $CDCl_3$) δ 142.4, 132.3, 130.2, 118.7, 111.3, 48.8, 42.2, 28.7, 20.0, 11.4. **IR** (film) 2963, 2924, 2871, 2229, 1606, 1499, 1454, 1415, 1288, 1180, 1080, 962, 842, 741, 651. **HRMS** (EI) Calcd for $C_{12}H_{15}NS_3$ 269.0365, Found 269.0365.

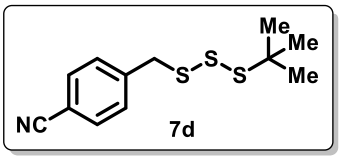

The reaction of 2-methyl-2-propanethiol (0.22 mmol, 1.1 equivalents, 19.9 mg), $B(C_6H_5)_3$ (0.005 mmol, 2.5 mol%, 2.6 mg), 4-$NCC_6H_4CH_2SSOMe$ (0.2 mmol, 1 equivalent, 42.3 mg) in DCM (2 mL) at r.t. for 5 hours afforded compound **7d** in 94% yield (50.8 mg) as a white solid according to the general procedure E. **^{1}H NMR** (400 MHz, $CDCl_3$) δ 7.61 (d, J = 8.1 Hz, 2H), 7.42 (d, J = 8.2 Hz, 2H), 4.07 (s, 2H), 1.36 (s, 9H). **^{13}C NMR** (100 MHz, $CDCl_3$) δ 142.4, 132.2,

130.1, 118.7, 111.2, 49.1, 42.3, 29.8. **IR** (film) 2965, 2918, 2227, 1607, 1507, 1455, 1419, 1391,1161, 871, 851, 651. **HRMS** (EI) Calcd for $C_{12}H_{15}NS_3$ 269.0367, Found 269.0368.

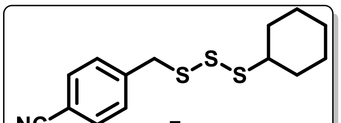

7e

The reaction of cyclohexanethiol (0.22 mmol, 1.1 equivalents, 27 uL), $B(C_6F_5)_3$ (0.005 mmol, 2.5 mol%, 2.6 mg), 4-$NCC_6H_4CH_2SSOMe$ (0.2 mmol, 1 equivalent, 42.3 mg) in DCM (2 mL) at r.t. for 5 hours afforded compound **7e** in 87% yield (49.2 mg) as a colorless oil according to the general procedure E. **^{1}H NMR** (400 MHz, $CDCl_3$) δ 7.62 (d, J = 8.2 Hz, 2H), 7.43 (d, J = 8.2 Hz, 2H), 4.07 (s, 2H), 2.90 (tt, J = 10.6, 3.7 Hz, 1H), 2.13-1.90 (m, 2H), 1.89-1.67 (m, 2H), 1.63-1.60 (m, 1H), 1.49-0.98 (m, 5H). **^{13}C NMR** (100 MHz, $CDCl_3$) δ 142.4, 132.3, 130.2, 118.7, 111.3, 50.1, 42.2, 32.5, 25.9, 25.5. **IR** (film) 2924, 2852, 2229, 1605, 1497, 1447, 1262, 1183, 1080, 965, 827, 739, 648. **HRMS** (EI) Calcd for $C_{14}H_{17}NS_3$ 295.0523, Found 295.0525.

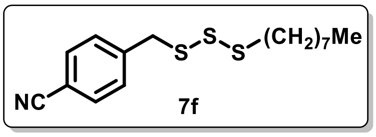

7f

The reaction of 1-octanethiol (0.22 mmol, 1.1 equivalents, 38 uL), $B(C_6F_5)_3$ (0.005 mmol, 2.5 mol%, 2.6 mg), 4-$NCC_6H_4CH_2SSOMe$ (0.2 mmol, 1 equivalent, 42.3 mg) in DCM (2 mL) at r.t. for 5 hours afforded compound **7f** in 85% yield (55.2 mg) as a colorless oil according to the general procedure E. **^{1}H NMR** (400 MHz, $CDCl_3$) δ 7.62 (d, J = 8.2 Hz, 2H), 7.43 (d, J = 8.2 Hz, 2H), 4.07 (s, 2H), 2.90 (tt, J = 10.6, 3.7 Hz, 1H), 2.13-1.90 (m, 2H), 1.89-1.67 (m, 2H), 1.63-1.60 (m, 1H), 1.49 - 0.98 (m, 5H). **^{13}C NMR** (100 MHz, $CDCl_3$) δ 142.3, 132.3, 130.1, 118.7, 111.3, 42.1, 38.8, 31.7, 29.1, 29.0, 28.7, 28.4, 22.6, 14.1. **IR** (film) 3063, 2925, 2853, 2229, 1726, 1606, 1503, 1461, 1289, 1196, 1020, 965, 842, 740, 652. **HRMS** (EI) Calcd for $C_{16}H_{23}NS_3$ 325.0993, Found 325.0989.

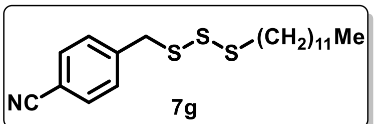

7g

The reaction of 1-dodecanethiol (0.22 mmol, 1.1 equivalents, 48 uL), 4-$NCC_6H_4CH_2SSOMe$ (0.2 mmol, 1 equivalent, 42.3 mg) in DCM (2 mL) at r.t. for 8 hours afforded compound **7g** in 89% yield (68 mg) as a colorless oil according to the general procedure E. **^{1}H NMR** (400 MHz, $CDCl_3$) δ 7.62 (d, J = 8.3 Hz, 2H),

7.43 (d, J = 8.3 Hz, 2H), 4.07 (s, 2H), 2.85-2.71 (m, 2H), 1.80-1.63 (m, 2H), 1.40-1.25 (m, 18H), 0.88 (t, J = 6.8 Hz, 3H). **^{13}C NMR** (100 MHz, $CDCl_3$) δ 142.3, 132.3, 130.1, 118.6, 111.4, 42.2, 38.9, 31.9, 29.6, 29.6, 29.5, 29.4, 29.3, 29.1, 28.7, 28.4, 22.7, 14.1. **IR** (film) 2922, 2852, 2229, 1606, 1504, 1461, 1414, 1234, 1079, 963, 842, 742, 652. **HRMS** (EI) Calcd for $C_{20}H_{31}NS_3$ 381.1619, Found 381.1626.

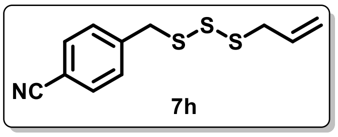

The reaction of allyl mercaptan (0.22 mmol, 1.1 equivalents, 18 uL), $B(C_6F_5)_3$ (0.005 mmol, 2.5 mol%, 2.6 mg), 4-$NCC_6H_4CH_2SSOMe$ (0.2 mmol, 1 equivalent, 42.3 mg) in DCM (2 mL) at r.t. for 5 hours afforded compound **7h** in 86% yield (41.3 mg) as a colorless oil according to the general procedure E. **^{1}H NMR** (400 MHz, $CDCl_3$) δ 7.62 (d, J = 8.2 Hz, 2H), 7.43 (d, J = 8.2 Hz, 2H), 5.89-5.79 (m, 1H), 5.28-5.14 (m, 2H), 4.07 (s, 2H), 3.45 (d, J = 7.3 Hz, 2H). **^{13}C NMR** (100 MHz, $CDCl_3$) δ 142.2, 132.4, 132.3, 130.1, 119.3, 118.6, 111.3, 42.3, 41.5. **IR** (film) 2979, 2913, 2229,1923, 1632, 1606, 1505, 1414, 1223, 1103, 1073, 916, 844, 724, 649. **HRMS** (EI) Calcd for $C_{11}H_{11}NS_3$ 253.0544, Found 253.0049.

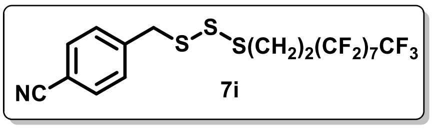

The reaction of thiol (0.22 mmol, 1.1 equivalents, 105.8 mg), 4-$NCC_6H_4CH_2SSOMe$ (0.2 mmol, 1 equivalent, 42.3 mg) in DCM (2 mL) at r.t. for 8 hours afforded compound **7i** in 78% yield (103.1 mg) as a white solid according to the general procedure E. **^{1}H NMR** (400 MHz, $CDCl_3$) δ7.64 (d, J = 8.0 Hz, 2H), 7.44 (d, J = 7.9 Hz, 2H), 4.09 (s, 2H), 3.29 - 2.86 (m, 2H), 2.62-2.49 (m, 2H). **^{19}F NMR** (377 MHz, $CDCl_3$) 19F NMR (376 MHz, CDCl3) δ -80.8 (3F, t, J = 9.9 Hz), -[113.8(6)-113.9(3)] (2F, m. CF_2), -[121.7-121.8] (2F, m. CF_2), -122.0 (4F, s, 2 × CF_2), -122.8 (2F, s, CF_2), -123.3 (2F, s, CF_2), -126.17 (2F, m. CF_2). **^{13}C NMR** (100 MHz, $CDCl_3$) δ 141.8, 132.4, 130.2, 118.6, 111.6, 42.1, 31.3 (t,$^2J_{\text{C-F}}$ = 22.2 Hz), 28.8. **IR** (film) 2952, 2920, 2230,1332, 1147, 1116, 953, 844, 703, 647. **HRMS** (EI) Calcd for $C_{18}H_{10}F_{17}NS_3$ 658.9704, Found 658.9711.

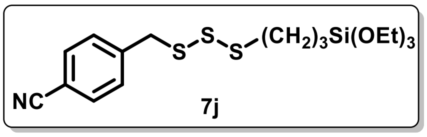

The reaction of thiol (0.22 mmol, 1.1 equivalents, 53 uL), $B(C_6F_5)_3$ (0.005 mmol, 2.5 mol%, 2.6 mg),

4-$NCC_6H_4CH_2SSOMe$ (0.2 mmol, 1 equivalent, 42.3 mg) in DCM (2 mL) at r.t. for 5 hours afforded compound **7j** in 52% yield (43.6 mg) as a colorless oil according to the general procedure E. **^{1}H NMR** (400 MHz, $CDCl_3$) δ 7.61 (d, *J* = 7.9 Hz, 2H), 7.43 (d, *J* = 8.1 Hz, 2H), 4.06 (s, 2H), 3.81 (q, *J* = 7.0 Hz, 6H), 2.84 (t, *J* = 7.2 Hz, 2H), 1.87-1.80 (m, 2H), 1.21 (t, *J* = 7.0 Hz, 9H), 0.90 - 0.41 (m, 2H). **^{13}C NMR** (100 MHz, $CDCl_3$) δ 142.3, 132.3, 130.1, 118.7, 111.2, 58.4, 42.0, 41.5, 22.3, 18.3, 9.5. **IR** (film) 2974, 2888, 2229, 1606, 1390, 1242, 1165, 1075, 957, 785. **HRMS** (EI) Calcd for $C_{17}H_{27}NO_3S_3$ 417.0922, Found 417.0914.

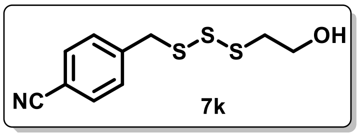

The reaction of 2-mercaptoethanol (0.22 mmol, 1.1 equivalents, 15.5 uL), $B(C_6F_5)_3$ (0.005 mmol, 2.5 mol%, 2.6 mg), 4-$NCC_6H_4CH_2SSOMe$ (0.2 mmol, 1 equivalent, 42.3 mg) in DCM (2 mL) at r.t. for 5 hours afforded compound **7k** in 89% yield (46 mg) as a white solid according to the general procedure E. **^{1}H NMR** (400 MHz, $CDCl_3$) δ 7.62 (d, *J* = 8.2 Hz, 2H), 7.43 (d, *J* = 8.0 Hz, 2H), 4.09 (s, 2H), 3.90 (t, *J* = 5.6 Hz, 2H), 2.98 (t, *J* = 5.8 Hz, 2H), 2.17 (s, 1H). **^{13}C NMR** (100 MHz, $CDCl_3$) δ 141.9, 132.3, 130.1, 118.6, 111.4, 59.6, 42.1, 41.4. **IR** (film) 3381, 2927, 2232, 1607, 1504, 1415, 1182, 1104, 1040, 1101, 842, 783, 650. **HRMS** (EI) Calcd for $C_{10}H_{11}NOS_3$ 257.0003, Found 257.0008.

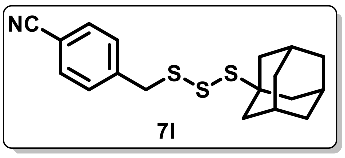

The reaction of 1-adamantanethiol (0.22 mmol, 1.1 equivalents, 37 mg), $B(C_6F_5)_3$ (0.005 mmol, 2.5 mol%, 2.6 mg), 4-$NCC_6H_4CH_2SSOMe$ (0.2 mmol, 1 equivalent, 42.3 mg) in DCM (2 mL) at r.t. for 5 hours afforded compound **7l** in 89% yield (62.2 mg) as a white solid according to the general procedure E. **^{1}H NMR** (400 MHz, $CDCl_3$) δ 7.61 (d, *J* = 8.2 Hz, 2H), 7.43 (d, *J* = 8.2 Hz, 2H), 4.06 (s, 2H), 2.08 (s, 3H), 1.88 (m, 6H), 1.74-1.63 (m, 6H); **^{13}C NMR** (100 MHz, $CDCl_3$) δ 142.4, 132.2, 130.1, 118.7, 111.1, 50.8, 42.4, 42.3, 35.9, 29.8; **IR** (film) 2905, 2851, 2229, 1607, 1504, 1448, 1414, 1295, 1038, 908, 840, 732, 651; **HRMS** (EI) Calcd for $C_{18}H_{21}NS_3$ 347.0836, Found 347.0829.

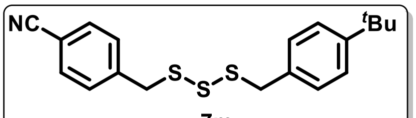

7m

The reaction of 4-(tert-butyl)benzyl mercaptan (0.22 mmol, 1.1 equivalents, 39.8 mg), 4-$NCC_6H_4CH_2SSOMe$ (0.2 mmol, 1 equivalent, 42.3 mg) in DCM (2 mL) at r.t. for 8 hours afforded compound **7m** in 92% yield (66.4 mg) as a colorless oil according to the general procedure E. **^{1}H NMR** (400 MHz, $CDCl_3$) δ 7.61 (d, J = 8.3 Hz, 2H), 7.39 (d, J = 8.3 Hz, 2H), 7.36 (d, J = 8.3 Hz, 2H), 7.24 (d, J = 8.3 Hz, 2H), 4.04 (s, 2H), 3.98 (s, 2H), 1.31 (s, 9H). **^{13}C NMR** (100 MHz, $CDCl_3$) δ 150.8, 142.2, 133.1, 132.3, 130.1, 129.1, 125.6, 118.6, 111.4, 42.9, 42.3, 34.5, 31.3. **IR** (film) 3055, 2956, 2228, 1606, 1508, 1362, 1293, 1200, 970, 836, 657. **HRMS** (EI) Calcd for $C_{19}H_{21}NS_3$ 359.0836, Found 359.0838.

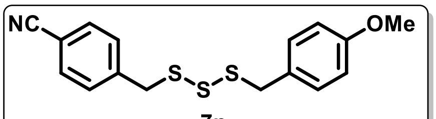

7n

The reaction of 4-(Methoxy)benzyl mercaptan (0.22 mmol, 1.1 equivalents, 34 mg), 4-$NCC_6H_4CH_2SSOMe$ (0.2 mmol, 1 equivalent, 42.3 mg) in DCM (2 mL) at r.t. for 8 hours afforded compound **7n** in 87% yield (58.2 mg) as a colorless oil according to the general procedure E. **^{1}H NMR** (400 MHz, $CDCl_3$) δ 7.61 (d, J = 8.2 Hz, 2H), 7.40 (d, J = 8.2 Hz, 2H), 7.21 (d, J = 8.6 Hz, 2H), 6.86 (d, J = 8.7 Hz, 2H), 4.01 (s, 4H), 3.80 (s, 3H). **^{13}C NMR** (100 MHz, $CDCl_3$) δ 159.2, 142.3, 132.3, 132.3, 130.5, 130.1, 128.1, 118.6, 114.1, 114.0, 111.3, 55.3, 42.6, 42.2. **IR** (film) 3065, 2929, 2838, 2229, 1607, 1509, 1300, 1250, 1174, 1032, 825, 741, 649. **HRMS** (EI) Calcd for $C_{16}H_{15}NOS_3$ 333.0316, Found 333.0323.

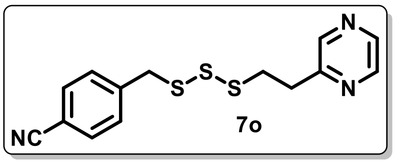

7o

The reaction of 2-pyrazinylethanethiol (0.22 mmol, 1.1 equivalents, 30.8 mg), 4-$NCC_6H_4CH_2SSOMe$ (0.2 mmol, 1 equivalent, 42.3 mg) in DCM (2 mL) at r.t. for 8 hours afforded compound **7o** in 84% yield (53.8 mg) as a colorless oil according to the general procedure E. **^{1}H NMR** (400 MHz, $CDCl_3$) δ 8.54 - 8.40 (m, 3H), 7.60 (d, J = 8.3 Hz, 2H), 7.41 (d, J = 8.2 Hz, 2H), 4.06 (s, 2H), 3.25 (s, 4H). **^{13}C NMR** (100 MHz, $CDCl_3$) δ 154.8, 145.0, 144.3, 142.7, 142.0, 132.3, 130.1, 118.6, 111.4, 42.2, 37.3, 34.2. **IR** (film) 2974, 2229, 1720, 1517, 1476, 1403, 1212, 1160, 1058, 1017, 768. **HRMS** (ESI) Calcd for $C_{14}H_{13}N_3S_3$ $(M+H^+)$ 320.0344, Found 320.0311.

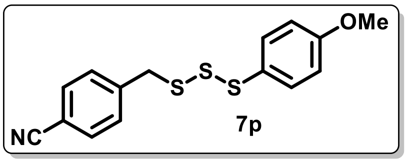

7p

The reaction of 4-methoxythiophenol (0.22 mmol, 1.1 equivalents, 30.9 mg), $B(C_6F_5)_3$ (0.005 mmol, 2.5 mol%, 2.6 mg), 4-$NCC_6H_4CH_2SSOMe$ (0.2 mmol, 1 equivalent, 42.3 mg) in DCM (2 mL) at r.t. for 5 hours afforded compound **7p** in 86% yield (55.4mg) as a colorless oil according to the general procedure E. ^{1}**H NMR** (400 MHz, $CDCl_3$) δ 7.58 (d, J = 8.1 Hz, 2H), 7.53 (d, J = 8.7 Hz, 2H), 7.30 (d, J = 8.1 Hz, 2H), 6.89 (d, J = 8.7 Hz, 2H), 3.99 (s, 2H), 3.82 (s, 3H). ^{13}**C NMR** (100 MHz, $CDCl_3$) δ 160.7, 142.1, 134.3, 132.2, 130.1, 127.2, 118.6, 114.8, 111.2, 55.4, 42.2. **IR** (film) 3056, 2974, 2226, 1587, 1490, 1459, 1291, 1245, 1174, 1024, 822, 634. **HRMS** (EI) Calcd for $C_{15}H_{13}NOS_3$ 319.0159, Found 319.0154.

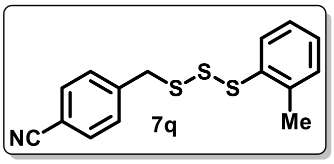

7q

The reaction of 2-methylthiophenol (0.22 mmol, 1.1 equivalents, 27.4 mg), $B(C_6F_5)_3$ (0.005 mmol, 2.5 mol%, 2.6 mg), 4-$NCC_6H_4CH_2SSOMe$ (0.2 mmol, 1 equivalent, 42.3 mg) in DCM (2 mL) at r.t. for 8 hours afforded compound **7q** in 95% yield (58.8 mg) as a colorless oil according to the general procedure E. ^{1}**H NMR** (400 MHz, $CDCl_3$) δ 7.72-7.65 (m, 1H), 7.59 (d, J = 8.3 Hz, 2H), 7.31-7.19 (m, 5H), 3.99 (s, 2H), 2.46 (s, 3H). ^{13}**C NMR** (100 MHz, $CDCl_3$) δ 142.1, 139.8, 135.2, 132.7, 132.3, 130.7, 130.1, 129.2, 126.7, 118.6, 111.4, 42.2, 20.7. **IR** (film) 2970, 2228, 1918, 1605, 1503, 1463, 1413, 1045, 842, 749, 707, 649. **HRMS** (EI) Calcd for $C_{15}H_{13}NS_3$ 303.0210, Found 303.0211.

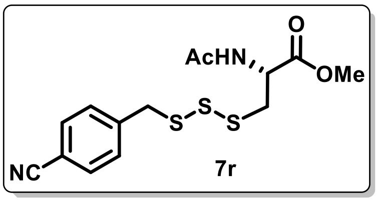

7r

The reaction of RSH (0.22 mmol, 1.1 equivalents, 39 mg), $B(C_6F_5)_3$ (0.005 mmol, 2.5 mol%, 2.6 mg), 4-$NCC_6H_4CH_2SSOMe$ (0.2 mmol, 1 equivalent, 42.3 mg) in DCM (0.5 mL) at r.t. for 24 hours afforded compound **7r** in 65% yield (46.6 mg) as a white solid according to the general procedure E. ^{1}**H NMR** (400 MHz, $CDCl_3$) δ 7.62 (d, J = 7.9 Hz, 2H), 7.42 (d, J = 8.0 Hz, 2H), 6.38 (s, 1H), 4.94 (s, 1H), 4.08 (s, 2H), 3.76 (s, 3H), 3.38-3.29 (m, 2H), 2.03 (s, 3H). ^{13}**C NMR** (100 MHz, $CDCl_3$) δ 170.6, 141.8, 132.4, 130.2, 118.6, 111.5, 77.3, 77.0, 76.7, 52.8, 51.6, 42.2, 40.8, 23.2. **IR** (film) 3330, 2920, 2851, 2230, 1711, 1609,

1533, 1416, 1373, 1209, 1123, 969, 826, 730, 648. **HRMS** (ESI) Calcd for $C_{14}H_{16}N_2O_3S_3$ (M+Na$^+$) 379.0215, Found 379.0208.

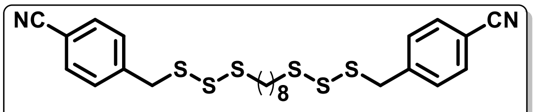

7s

The reaction of 1,8-octanedithiol (0.11 mmol, 0.55 equivalent, 19.6 mg), $B(C_6F_5)_3$ (0.005 mmol, 2.5 mol%, 2.6 mg), 4-$NCC_6H_4CH_2SSOMe$ (0.2 mmol, 1 equivalent, 42.3 mg) in DCM (2 mL) at r.t. for 5 hours afforded compound **7s** in 85% yield (46.1 mg) as a colorless oil according to the general procedure E. **^{1}H NMR** (400 MHz, $CDCl_3$) δ 7.62 (d, J = 8.2 Hz, 4H), 7.43 (d, J = 8.2 Hz, 4H), 4.07 (s, 4H), 2.80 (t, J = 7.3 Hz, 4H), 1.69 (dt, J = 14.7, 7.3 Hz, 4H), 1.37-1.30 (m, 8H). **^{13}C NMR** (100 MHz, $CDCl_3$) δ 142.3, 132.3, 130.1, 118.7, 111.3, 42.1, 38.7, 28.9, 28.6, 28.2. **IR** (film) 2924, 2853, 2227, 1606, 1503, 1413, 1291, 1233, 1051, 843, 721, 650. **HRMS** (ESI) Calcd for $C_{22}H_{24}N_2O_2S_6$ (M+Na$^+$) 559.0469, Found 559.0462.

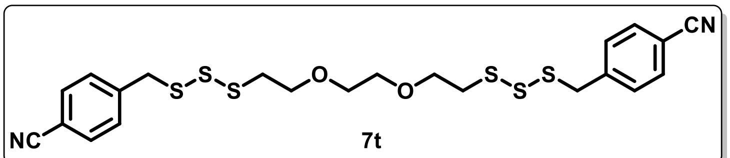

7t

The reaction of 3,6-dioxa-1,8-octanedithiol (0.11 mmol, 0.55 equivalent, 20.1 mg), $B(C_6F_5)_3$ (0.005 mmol, 2.5 mol%, 2.6 mg), 4-$NCC_6H_4CH_2SSOMe$ (0.2 mmol, 1 equivalent, 42.3 mg) in DCM (2 mL) at r.t. for 5 hours afforded compound **7t** in 79% yield (42.9 mg) as a white solid according to the general procedure E. **^{1}H NMR** (400 MHz, $CDCl_3$) δ 7.62 (d, J = 8.2 Hz, 4H), 7.43 (d, J = 8.2 Hz, 4H), 4.07 (s, 4H), 3.76 (t, J = 6.6 Hz, 4H), 3.63 (s, 4H), 3.01 (t, J = 6.6 Hz, 4H). **^{13}C NMR** (100 MHz, $CDCl_3$) δ 142.1, 132.3, 130.1, 118.6, 111.3, 70.4, 69.1, 42.0, 38.0. **IR** (film) 3063, 2854, 2227, 1605, 1503, 1417, 1325, 1290, 1196, 1103, 1068, 845, 737, 650. **HRMS** (ESI) Calcd for $C_{22}H_{24}N_2O_2S_6$ (M+NH_4^+) 558.0500, Found 558.0449.

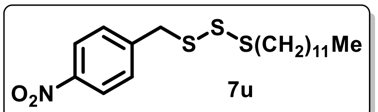

7u

The reaction of 1-dodecanethiol (0.22 mmol, 1.1 equivalents, 44.5 mg), $B(C_6F_5)_3$ (0.005 mmol, 2.5 mol%, 2.6 mg), 4-$NO_2C_6H_4CH_2SSOMe$ (0.2 mmol, 1 equivalent, 46.3 mg) in DCM (2 mL) at r.t. for 5 hours afforded compound **7u** in 99% yield (79.4 mg) as a white solid according to the general procedure E. **^{1}H NMR** (400

MHz, $CDCl_3$) δ 8.19 (d, J = 8.7 Hz, 2H), 7.49 (d, J = 8.7 Hz, 2H), 4.11 (s, 2H), 2.81 (t, J = 7.2 Hz, 2H), 1.76-1.63 (m, 2H), 1.40-1.23 (m, 18H), 0.87 (t, J = 6.8 Hz, 3H). **^{13}C NMR** (100 MHz, $CDCl_3$) δ 147.2, 144.4, 130.2, 123.7, 41.6, 38.8, 31.9, 29.6, 29.5(8), 29.5, 29.4, 29.3, 29.1, 28.7, 28.4, 22.6, 14.1. **IR** (film) 2925, 2849, 1601, 1521, 1344, 1178, 962, 800, 705. **HRMS** (EI) Calcd for $C_{19}H_{31}NO_2S_3$ 401.1517, Found 401.1519.

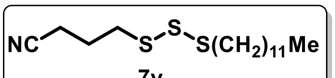

7v

The reaction of 1-dodecanethiol (0.22 mmol, 1.1 equivalents, 44.5 mg), $B(C_6F_5)_3$ (0.005 mmol, 2.5 mol%, 2.6 mg), 4-$NC(CH_2)_3SSOMe$ (0.2 mmol, 1 equivalent, 32.7 mg) in DCM (2 mL) at r.t. for 5 hours afforded compound **7v** in 99% yield (60.7 mg) as a colorless oil according to the general procedure E. **^{1}H NMR** (400 MHz, $CDCl_3$) δ 2.97 (t, J = 6.7 Hz, 2H), 2.86 (t, J = 7.2 Hz, 2H), 2.54 (t, J = 7.0 Hz, 2H), 2.19-2.12 (m, 2H), 1.76-1.69 (m, 2H), 1.41-1.21 (m, 18H), 0.87 (t, J = 6.8 Hz, 3H). **^{13}C NMR** (100 MHz, $CDCl_3$) δ 118.9, 38.8, 36.4, 31.8, 29.6, 29.6, 29.5, 29.4, 29.3, 29.1, 28.6, 28.4, 24.0, 22.6, 15.7, 14.1. **IR** (film) 2924, 2849, 2248, 1462, 1296, 971, 806, 723. **HRMS** (EI) Calcd for $C_{16}H_{31}NS_3$ 333.1619, Found 333.1619.

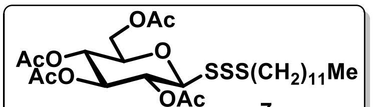

7w

The reaction of 1-dodecanethiol (0.22 mmol, 1.1 equivalents, 44.5 mg), $B(C_6F_5)_3$ (0.005 mmol, 2.5 mol%, 2.6 mg), RSSOMe (0.2 mmol, 1 equivalent, 85.3 mg) in DCM (0.5 mL) at r.t. for 5 hours afforded compound **7w** in 88% yield (60.7 mg) as a white solid according to the general procedure E. **^{1}H NMR** (400 MHz, $CDCl_3$) δ 5.24-5.21 (m, 1H), 5.17-5.02 (m, 2H), 4.65 (d, J = 9.8 Hz, 1H), 4.26 (d of ABq, J = 12.5, 4.6 Hz, 1H), 4.12 (d of ABq, J = 12.4, 1.8 Hz, 1H), 3.76 (ddd, J = 9.8, 4.3, 2.0 Hz, 1H), 2.85 (t, J = 6.9 Hz, 2H), 2.05 (s, 3H), 2.00 (s, 3H), 1.99 (s, 3H), 1.97 (s, 3H), 1.71-1.63 (m, 2H), 1.42-1.16 (m, 18H), 0.84 (t, J = 6.7 Hz, 3H). **^{13}C NMR** (100 MHz, $CDCl_3$) δ 170.4, 170.0, 169.2, 169.0, 87.6, 76.3, 73.7, 69.6, 67.9, 61.8, 39.2, 31.8, 29.5, 29.4(5), 29.3, 29.2, 29.1, 28.6, 28.3, 22.6, 20.6(4), 20.6(3), 20.5(7), 20.5($_6$),20.4(5), 14.0. **IR** (film) 2978, 2904, 1745, 1403, 1227, 1005, 892. **HRMS** (ESI) Calcd for $C_{26}H_{44}O_9S_3$ (M+Na^+) 619.2039, Found 619.2040.

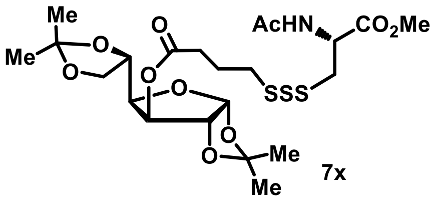

7x

The reaction of RSH (0.22 mmol, 1.1 equivalents, 39 mg), $B(C_6F_5)_3$ (0.005 mmol, 2.5 mol%, 2.6 mg), RSSOMe (0.2 mmol, 1 equivalent, 84.9 mg) in DCM (0.5 mL) at r.t. for 5 hours afforded compound **7x** in 60% yield (68.3 mg) as a colorless oil according to the general procedure E. **^{1}H NMR** (400 MHz, $CDCl_3$) δ 6.48 (d, J = 7.1 Hz, 1H), 5.85 (d, J = 3.6 Hz, 1H), 5.25 (s, 1H), 4.92 (dt, J = 7.7, 4.9 Hz, 1H), 4.46 (d, J = 3.6 Hz, 1H), 4.16 (s, 2H), 4.09-4.04 (m, 1H), 4.01-3.94 (m, 1H), 3.75 (s, 3H), 3.45-3.27 (m, 2H), 2.90 (t, J = 7.0 Hz, 2H), 2.48 (t, J = 7.1 Hz, 2H), 2.14-2.01 (m, 5H), 1.48 (s, 3H), 1.37 (s, 3H), 1.28 (s, 3H), 1.27 (s, 3H). **^{13}C NMR** (100 MHz, $CDCl_3$) δ 171.3, 170.6, 169.8, 112.2, 109.3, 105.0, 83.3, 79.8, 76.1, 72.4, 67.3, 52.7, 51.6, 40.6, 37.4, 32.4, 26.8, 26.6, 26.1, 25.2, 23.6, 23.0. **IR** (film) 3304, 2986, 2939, 1743, 1660, 1528, 1375, 1213, 1162, 1073, 1021, 846, 732, 642. **HRMS** (ESI) Calcd for $C_{22}H_{35}NO_{10}S_3$ (M+Na$^+$) 592.1315, Found 592.1344.

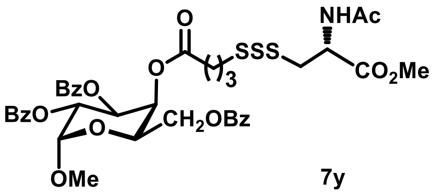

7y

The reaction of RSH (0.22 mmol, 1.1 equivalents, 39 mg), $B(C_6F_5)_3$ (0.005 mmol, 2.5 mol%, 2.6 mg), RSSOMe (0.2 mmol, 1 equivalent, 134.2 mg) in DCM (0.5 mL) at r.t. for 5 hours afforded compound **7y** in 40% yield (65.2 mg) as a white solid according to the general procedure E. **^{1}H NMR** (400 MHz, $CDCl_3$) δ 8.02 (d, J = 7.3 Hz, 2H), 7.99 (d, J = 7.4 Hz, 2H), 7.88 (d, J = 7.3 Hz, 2H), 7.59-7.48 (m, 3H), 7.44 (t, J = 7.7 Hz, 2H), 7.40-7.34 (m, 4H), 6.52 (d, J = 7.5 Hz, 1H), 5.91-5.77 (m, 2H), 5.60 (d of ABq, J = 10.6, 3.6 Hz, 1H), 5.22 (d, J = 3.5 Hz, 1H), 4.93 (dt, J = 7.6, 5.0 Hz, 1H), 4.53-4.48 (m, 2H), 4.39-4.33 (m, 1H), 3.75 (s, 3H), 3.47 (s, 3H), 3.37-3.36 (m, 2H), 2.89-2.74 (m, 2H), 2.62 (t, J = 7.2 Hz, 2H), 2.09-1.99 (m, 5H). **^{13}C NMR** (100 MHz, $CDCl_3$) δ 171.8, 170.7, 169.8, 166.1, 166.0, 165.4, 133.4, 133.3(1), 133.3(0),129.8, 129.7, 129.5, 129.4, 129.2, 129.1, 128.5, 128.4, 97.6, 68.9, 68.6, 68.4, 66.5, 62.3, 55.7, 52.7, 51.6, 40.7, 37.5, 32.1, 23.5, 23.1. **IR** (film) 3376, 2976, 2904, 1723, 1678, 1375, 1262, 1066, 710. **HRMS** (ESI) Calcd for $C_{38}H_{41}NO_{13}S_3$ (M+Na$^+$) 838.1632, Found 838.1664.

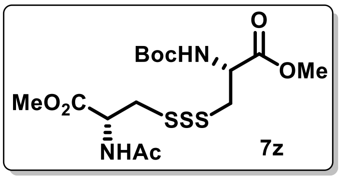

The reaction of RSH (0.22 mmol, 1.1 equivalents, 39 mg), $B(C_6F_5)_3$ (0.005 mmol, 2.5 mol%, 2.6 mg), 4-RSSOMe (0.2 mmol, 1 equivalent, 59.5 mg) in DCM (0.5 mL) at r.t. for 24 hours afforded compound **7z** in 98% yield (86.7 mg) as a white solid according to the general procedure E. **^{1}H NMR** (400 MHz, $CDCl_3$) δ 6.69 (brs, 1H), 5.49 (brs, 1H), 4.86 (s, 1H), 4.61 (s, 1H), 3.72 (s, 3H), 3.72 (s, 3H), 3.35-3.31 (m, 4H), 2.01 (s, 3H), 1.39 (s, 9H). **^{13}C NMR** (125 MHz, $CDCl_3$) δ 170.8, 170.6, 170.0, 154.9, 80.2, 52.7, 52.7, 52.6, 51.5, 41.3, 40.6, 28.1, 22.9. **IR** (film) 3337, 2973, 1743, 1712, 1664, 1517, 1369, 1216, 1163, 1050, 782. **HRMS** (EI) Calcd for $C_{15}H_{26}N_2O_7S_3$ 442.0902, Found 442.0907.

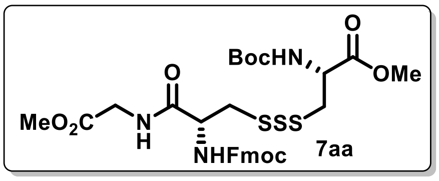

The reaction of RSH (0.22 mmol, 1.1 equivalents, 91.2 mg), $B(C_6F_5)_3$ (0.005 mmol, 2.5 mol%, 2.6 mg), RSSOMe (0.2 mmol, 1 equivalent, 59.5 mg) in DMF (0.5 mL) at r.t. for 8 hours afforded compound **7aa** in 52% yield (70.6 mg) as a colorless oil according to the general procedure E. **^{1}H NMR** (400 MHz, DMSO-d_6) δ 8.53 (t, J = 5.6 Hz, 1H), 7.89 (d, J = 7.5 Hz, 2H), 7.78 (d, J = 8.5 Hz, 1H), 7.73 (d, J = 7.4 Hz, 2H), 7.42 (t, J = 7.5 Hz, 3H), 7.33 (t, J = 7.4 Hz, 2H), 4.55-4.30 (m, 3H), 4.29-4.20 (m, 2H), 3.96-3.76 (m, 2H), 3.65 (s, 3H), 3.63 (s, 3H), 3.34-3.22 (m, 2H), 3.12 (td, J = 14.0, 10.1 Hz, 2H), 1.38 (s, 9H). **^{13}C NMR** (100 MHz, DMSO-d_6) δ 170.6, 169.9, 169.4, 155.4, 154.8, 143.3, 140.2, 127.1, 126.5, 124.8, 119.6, 78.1, 65.3, 53.1, 52.1, 51.7, 51.2, 46.1, 40.6, 39.7, 38.5, 27.6. **IR** (film) 3330, 2977,2903, 1691, 1519, 1403, 1223, 1163, 1049, 866, 739. **HRMS** (EI) Calcd for $C_{30}H_{37}N_3O_9S_3$ (M+Na$^+$) 702.1584, Found 702.1598.

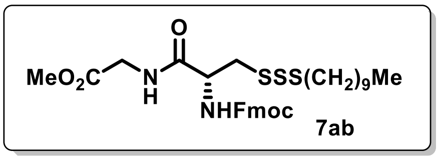

The reaction of RSH (0.22 mmol, 1.1 equivalents, 91.2 mg), $B(C_6F_5)_3$ (0.005 mmol, 2.5 mol%, 2.6 mg), $Me(CH_2)_9SSOMe$ (0.2 mmol, 1 equivalent, 47.3 mg) in DCM (2 mL) at r.t. for 8 hours afforded compound **7ab** in 60% yield (74.3 mg) as a colorless oil according to the general procedure E. **^{1}H NMR** (400 MHz, $CDCl_3$) δ 7.76 (d, J = 7.5 Hz, 2H), 7.60 (d, J = 7.3 Hz, 2H), 7.40 (t, J = 7.4 Hz, 2H), 7.33-7.29 (m, 2H), 6.90 (s, 1H), 5.80 (s, 1H), 4.65 (s, 1H), 4.47-4.45 (d, J = 6.7 Hz, 2H), 4.23 (t, J = 6.8 Hz, 1H), 4.12-3.99 (m, 2H), 3.75 (s, 3H), 3.44-3.35 (m, 1H),

3.27-3.22 (m, 1H), 2.87 (t, *J* = 7.3 Hz, 2H), 1.75-1.68 (m, 2H), 1.39-1.34 (m, 2H), 1.30-1.22 (m, 12H), 0.88 (t, *J* = 6.8 Hz, 3H). **^{13}C NMR** (100 MHz, $CDCl_3$) δ 170.1, 169.8, 156.1, 143.7, 141.3, 127.8, 127.1, 125.1, 120.0, 67.4, 53.8, 52.5, 47.2, 41.4, 41.2, 38.9, 31.9, 29.5, 29.4(9), 29.3, 29.2, 28.8, 28.5, 22.7, 14.1. **IR** (film) 3293, 2976, 2908, 1741, 1692, 1651, 1533, 1403, 1255, 1051, 893, 734, 663. **HRMS** (ESI) Calcd for $C_{31}H_{42}N_2O_5S_3$ (M+H$^+$) 619.2329, Found 619.2326.

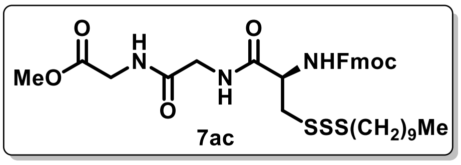

The reaction of RSH (0.22 mmol, 1.1 equivalents, 103.8 mg), $B(C_6F_5)_3$ (0.005 mmol, 2.5 mol%, 2.6 mg), $Me(CH_2)_9SSOMe$ (0.2 mmol, 1 equivalent, 47.3 mg) in DCM (2 mL) at r.t. for 8 hours afforded compound **7ab** in 50% yield (67.5mg) as a colorless oil according to the general procedure E. **^{1}H NMR** (400 MHz, $CDCl_3$) δ 7.75 (d, *J* = 7.5 Hz, 2H), 7.59 (d, *J* = 7.3 Hz, 2H), 7.39 (t, *J* = 7.4 Hz, 2H), 7.30 (t, *J* = 7.4 Hz, 2H), 7.21 (brs, 1H), 6.98 (brs, 1H), 5.94 (brs, 1H), 4.57 (s, 1H), 4.44 (m, 2H), 4.21 (t, *J* = 6.8 Hz, 1H), 4.16 - 3.84 (m, 4H), 3.69 (s, 3H), 3.31 (d, *J* = 4.7 Hz, 2H), 2.85 (t, *J* = 7.3 Hz, 2H), 1.70 (m, 2H), 1.40 - 1.21 (m, 14H), 0.88 (t, *J* = 6.8 Hz, 3H). **^{13}C NMR** (100 MHz, $CDCl_3$) δ 170.5, 170.1, 168.8, 156.3, 143.6, 141.3, 127.8, 127.1, 125.0, 120.0, 67.4, 54.1, 52.3, 47.1, 43.1, 41.1, 40.6, 38.8, 31.8, 29.5, 29.4(5), 29.2, 29.1, 28.7, 28.5, 22.6, 14.1. **IR** (film) 3292, 2970, 2921, 1742, 1689, 1644, 1529, 1404, 1257, 1068, 802, 736, 668. **HRMS** (ESI) Calcd for $C_{33}H_{45}N_3O_6S_3$ (M+Na$^+$) 698.2363, Found 698.2362.

Gram scale operation

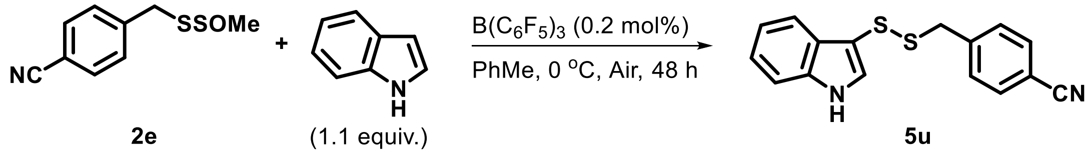

To a Schlenk tube were added indole (5.5 mmol, 1.1 equivalents, 644 mg), $B(C_6F_5)_3$ (0.01 mmol, 0.2 mol%, 5.1 mg), RSSOMe (5 mmol, 1 equivalent, 1.057 g), and toluene (1 mL), the mixture was stirred at 0 °C for 48 hours before it was concentrated under vacuum. Purification by column chromatography afforded the desired product in yield of 93% (1.38 g).

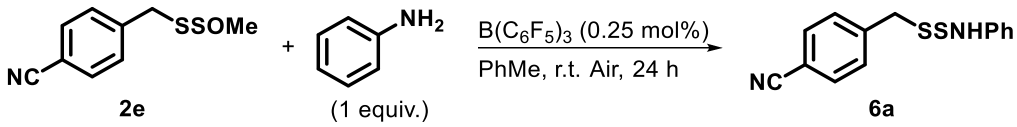

To a Schlenk tube were added amine (5 mmol, 1 equivalent, 465.7 mg), $B(C_6F_5)_3$ (0.0125 mmol, 0.25 mol%, 6.5 mg), RSSOMe (0.2 mmol, 1 equivalent, 1.057 g), and toluene (1 mL), the mixture was stirred at r.t. for 24 hours before it was concentrated under vacuum. Purification by column chromatography afforded the desired product in yield of 81% (1.1 g).

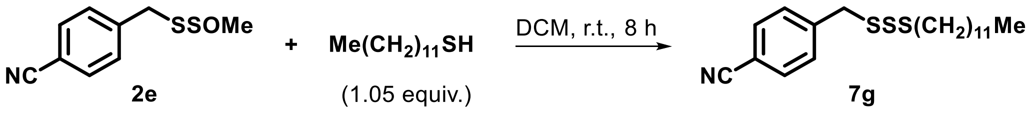

To a Schlenk tube were added thiol (5.25 mmol, 1.05 equivalents, 1.063 g), RSSOMe (0.2 mmol, 1 equivalent, 1.057 g), and DCM (10 mL), the mixture was stirred at r.t. for 6 hours before it was concentrated under vacuum. Purification by column chromatography afforded the desired product in yield of 92% (1.76 g).

X-ray Crystallography Analysis

Compound 3a (CCDC-1565934)

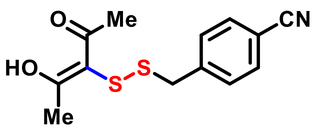

≡

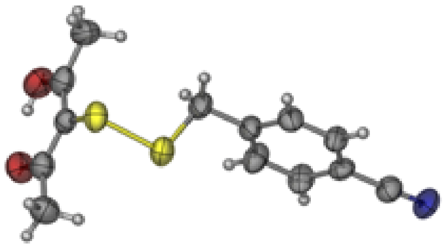

3a

Datablock: z

Bond precision: C-C = 0.0112 A Wavelength=0.71073

Cell:	a=7.509(3)	b=7.826(3)	c=12.235(5)
	alpha=76.567(13)	beta=86.274(15)	gamma=79.030(13)

Temperature: 296 K

	Calculated	Reported
Volume	686.4(5)	686.5(4)
Space group	P -1	P-1
Hall group	-P 1	?
Moiety formula	C13 H13 N O2 S2	?
Sum formula	C13 H13 N O2 S2	C13 H13 N O2 S2
Mr	279.36	279.36
Dx,g cm-3	1.352	1.352
Z	2	2
Mu (mm-1)	0.381	0.381
F000	292.0	292.0
F000'	292.60	
h,k,lmax	8,9,14	8,9,14
Nref	2419	2398
Tmin,Tmax	0.852,0.920	0.857,0.921
Tmin'	0.852	

Correction method= # Reported T Limits: Tmin=0.857 Tmax=0.921
AbsCorr = MULTI-SCAN

Data completeness= 0.991 Theta(max)= 25.010

R(reflections)= 0.0990(1287) wR2(reflections)= 0.3072(2398)

S = 1.036 Npar= 163

The following ALERTS were generated. Each ALERT has the format
test-name_ALERT_alert-type_alert-level.
Click on the hyperlinks for more details of the test.

Alert level B

PLAT340_ALERT_3_B Low Bond Precision on C-C Bonds .... 0.01125 Ang.

Alert level C

PLAT084_ALERT_3_C High wR2 Value (i.e. > 0.25) .... 0.31 Report

Alert level G

PLAT005_ALERT_5_G No Embedded Refinement Details found in the CIF Please Do !
PLAT007_ALERT_5_G Number of Unrefined Donor-H Atoms .... 1 Report
PLAT066_ALERT_1_G Predicted and Reported Tmin&Tmax Range Identical ? Check
PLAT072_ALERT_2_G SHELXL First Parameter in WGHT Unusually Large 0.18 Report
PLAT380_ALERT_4_G Incorrectly? Oriented X(sp2)-Methyl Moiety ..... C11 Check
PLAT380_ALERT_4_G Incorrectly? Oriented X(sp2)-Methyl Moiety ..... C13 Check
PLAT710_ALERT_4_G Delete 1-2-3 or 2-3-4 Linear Torsion Angle ... # 2 Do !
N1 -C1 -C2 -C3 72.00 13.00 1.555 1.555 1.555 1.555
PLAT710_ALERT_4_G Delete 1-2-3 or 2-3-4 Linear Torsion Angle ... # 3 Do !

Supplementary Figure 1. Single-Crystal X-ray Crystallography of **3a**.

Compound 5o (CCDC-1565935)

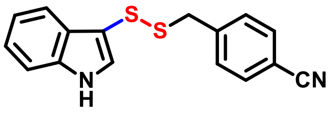

5o

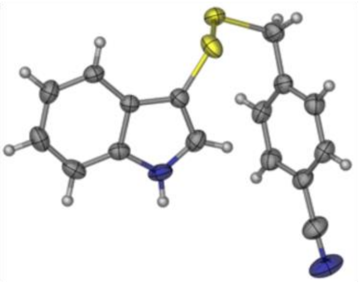

Datablock: z

Bond precision: C-C = 0.0021 A Wavelength=0.71073

Cell:	a=9.2933(2)	b=8.8912(2)	c=17.7846(4)
	alpha=90	beta=103.798(1)	gamma=90

Temperature: 296 K

	Calculated	Reported
Volume	1427.11(6)	1427.11(5)
Space group	P 21/c	P2(1)/c
Hall group	-P 2ybc	?
Moiety formula	C16 H12 N2 S2	?
Sum formula	C16 H12 N2 S2	C16 H12 N2 S2
Mr	296.40	296.40
Dx,g cm-3	1.380	1.380
Z	4	4
Mu (mm-1)	0.363	0.363
F000	616.0	616.0
F000'	617.16	
h,k,lmax	11,10,21	11,10,21
Nref	2517	2512
Tmin,Tmax	0.881,0.927	0.839,0.928
Tmin'	0.834	

Correction method= # Reported T Limits: Tmin=0.839 Tmax=0.928
AbsCorr = MULTI-SCAN

Data completeness= 0.998 Theta(max)= 25.010

R(reflections)= 0.0286(2301) wR2(reflections)= 0.0828(2512)

S = 0.988 Npar= 181

The following ALERTS were generated. Each ALERT has the format
test-name_ALERT_alert-type_alert-level.
Click on the hyperlinks for more details of the test.

Alert level G

PLAT005_ALERT_5_G No Embedded Refinement Details found in the CIF Please Do !
PLAT007_ALERT_5_G Number of Unrefined Donor-H Atoms .... 1 Report
PLAT066_ALERT_1_G Predicted and Reported Tmin&Tmax Range Identical ? Check
PLAT230_ALERT_2_G Hirshfeld Test Diff for C12 -- C16 .. 5.9 s.u.
PLAT710_ALERT_4_G Delete 1-2-3 or 2-3-4 Linear Torsion Angle ... # 37 Do !
C11 -C12 -C16 -N2 -85.00 5.00 1.555 1.555 1.555 1.555
PLAT710_ALERT_4_G Delete 1-2-3 or 2-3-4 Linear Torsion Angle ... # 38 Do !
C13 -C12 -C16 -N2 92.00 5.00 1.555 1.555 1.555 1.555
PLAT899_ALERT_4_G SHELXL97 is Deprecated and Succeeded by SHELXL 2014 Note

0 ALERT level A = Most likely a serious problem - resolve or explain
0 ALERT level B = A potentially serious problem, consider carefully
0 ALERT level C = Check. Ensure it is not caused by an omission or oversight
7 ALERT level G = General information/check it is not something unexpected

Supplementary Figure 2. Single-Crystal X-ray Crystallography of **5o**.

Compound 6a (CCDC-1565936)

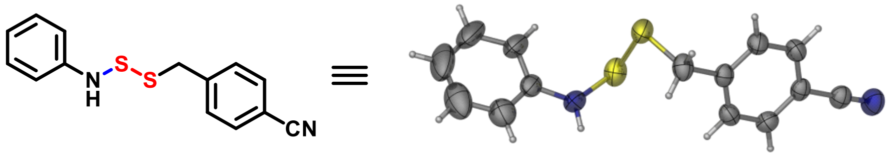

6a

Datablock: z

Bond precision:	C-C = 0.0042 A		Wavelength=0.71073
Cell:	a=15.913(3)	b=11.4032(17)	c=7.8620(12)
	alpha=90	beta=103.384(5)	gamma=90
Temperature: 296 K			

	Calculated	Reported
Volume	1387.9(4)	1387.9(4)
Space group	P 21/c	P2(1)/c
Hall group	-P 2ybc	?
Moiety formula	C14 H12 N2 S2	?
Sum formula	C14 H12 N2 S2	C14 H12 N2 S2
Mr	272.38	272.38
Dx,g cm-3	1.304	1.304
Z	4	4
Mu (mm-1)	0.367	0.367
F000	568.0	568.0
F000'	569.14	
h,k,lmax	18,13,9	18,13,9
Nref	2445	2444
Tmin,Tmax	0.851,0.971	0.855,0.971
Tmin'	0.851	

Correction method= # Reported T Limits: Tmin=0.855 Tmax=0.971
AbsCorr = MULTI-SCAN

Data completeness= 1.000 Theta(max)= 25.010

R(reflections)= 0.0405(1731) wR2(reflections)= 0.1038(2444)

S = 1.027 Npar= 163

The following ALERTS were generated. Each ALERT has the format
test-name_ALERT_alert-type_alert-level.
Click on the hyperlinks for more details of the test.

Alert level C

PLAT241_ALERT_2_C High 'MainMol' Ueq as Compared to Neighbors of C5 Check
PLAT242_ALERT_2_C Low 'MainMol' Ueq as Compared to Neighbors of C1 Check
PLAT340_ALERT_3_C Low Bond Precision on C-C Bonds .... 0.00421 Ang.

Alert level G

PLAT005_ALERT_5_G No Embedded Refinement Details found in the CIF Please Do !
PLAT007_ALERT_5_G Number of Unrefined Donor-H Atoms .... 1 Report
PLAT066_ALERT_1_G Predicted and Reported Tmin&Tmax Range Identical ? Check
PLAT710_ALERT_4_G Delete 1-2-3 or 2-3-4 Linear Torsion Angle ... # 26 Do !
C10 -C11 -C14 -N2 0.00 8.00 1.555 1.555 1.555 1.555
PLAT710_ALERT_4_G Delete 1-2-3 or 2-3-4 Linear Torsion Angle ... # 27 Do !
C12 -C11 -C14 -N2 18.00 0.00 1.555 1.555 1.555 1.555
PLAT899_ALERT_4_G SHELXL97 is Deprecated and Succeeded by SHELXL 2014 Note

0 **ALERT level A** = Most likely a serious problem - resolve or explain

Supplementary Figure 3. Single-Crystal X-ray Crystallography of **6a**.

Compound 7d (CCDC-1565937)

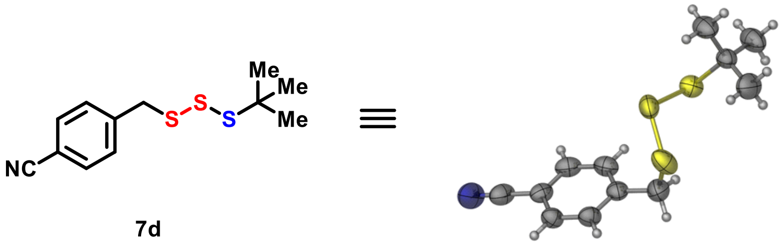

Datablock: z

Bond precision: C-C = 0.0030 A Wavelength=0.71073

Cell: a=15.2714(5) b=7.7707(2) c=12.0037(4)

alpha=90 beta=93.036(1) gamma=90

Temperature: 296 K

	Calculated	Reported
Volume	1422.47(8)	1422.47(8)
Space group	P 21/c	P2(1)/c
Hall group	-P 2ybc	?
Moiety formula	C12 H15 N S3	?
Sum formula	C12 H15 N S3	C12 H15 N S3
Mr	269.43	269.43
Dx,g cm-3	1.258	1.258
Z	4	4
Mu (mm-1)	0.496	0.496
F000	568.0	568.0
F000'	569.61	
h,k,lmax	18,9,14	18,9,14
Nref	2511	2505
Tmin,Tmax	0.841,0.915	0.838,0.916
Tmin'	0.832	

Correction method= # Reported T Limits: Tmin=0.838 Tmax=0.916

AbsCorr = MULTI-SCAN

Data completeness= 0.998 Theta(max)= 25.010

R(reflections)= 0.0328(2125) wR2(reflections)= 0.0899(2505)

S = 1.031 Npar= 145

The following ALERTS were generated. Each ALERT has the format

test-name_ALERT_alert-type_alert-level.

Click on the hyperlinks for more details of the test.

Alert level C

PLAT242_ALERT_2_C Low 'MainMol' Ueq as Compared to Neighbors of C9 Check

PLAT790_ALERT_4_C Centre of Gravity not Within Unit Cell: Resd. # 1 Note

C12 H15 N S3

Alert level G

PLAT005_ALERT_5_G No Embedded Refinement Details found in the CIF Please Do !

PLAT066_ALERT_1_G Predicted and Reported Tmin&Tmax Range Identical ? Check

PLAT710_ALERT_4_G Delete 1-2-3 or 2-3-4 Linear Torsion Angle ... # 13 Do !

C2 -C3 -C7 -N1 32.00 75.00 1.555 1.555 1.555 1.555

PLAT710_ALERT_4_G Delete 1-2-3 or 2-3-4 Linear Torsion Angle ... # 14 Do !

C4 -C3 -C7 -N1 15.00 0.00 1.555 1.555 1.555 1.555

PLAT899_ALERT_4_G SHELXL97 is Deprecated and Succeeded by SHELXL 2014 Note

0 **ALERT level A** = Most likely a serious problem - resolve or explain

0 **ALERT level B** = A potentially serious problem, consider carefully

Supplementary Figure 4. Single-Crystal X-ray Crystallography of **7d**.

All these data can be obtained free of charge from Cambridge Crystallographic Data Centre via www.ccdc.cam.ac.uk/ data_request/ci.

NMR Spectra

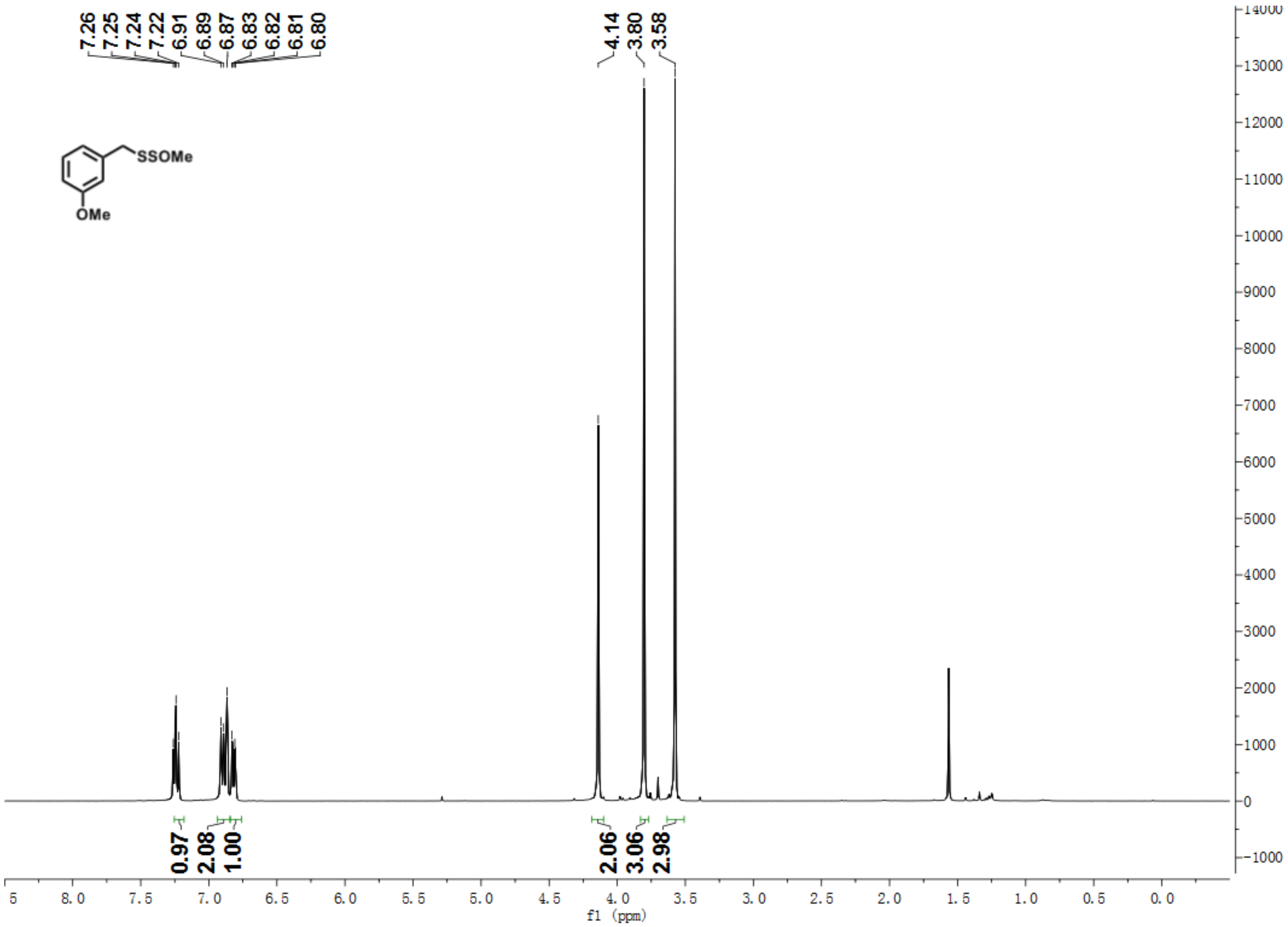

Supplementary Figure 5. ^{1}H NMR spectra for **2a**.

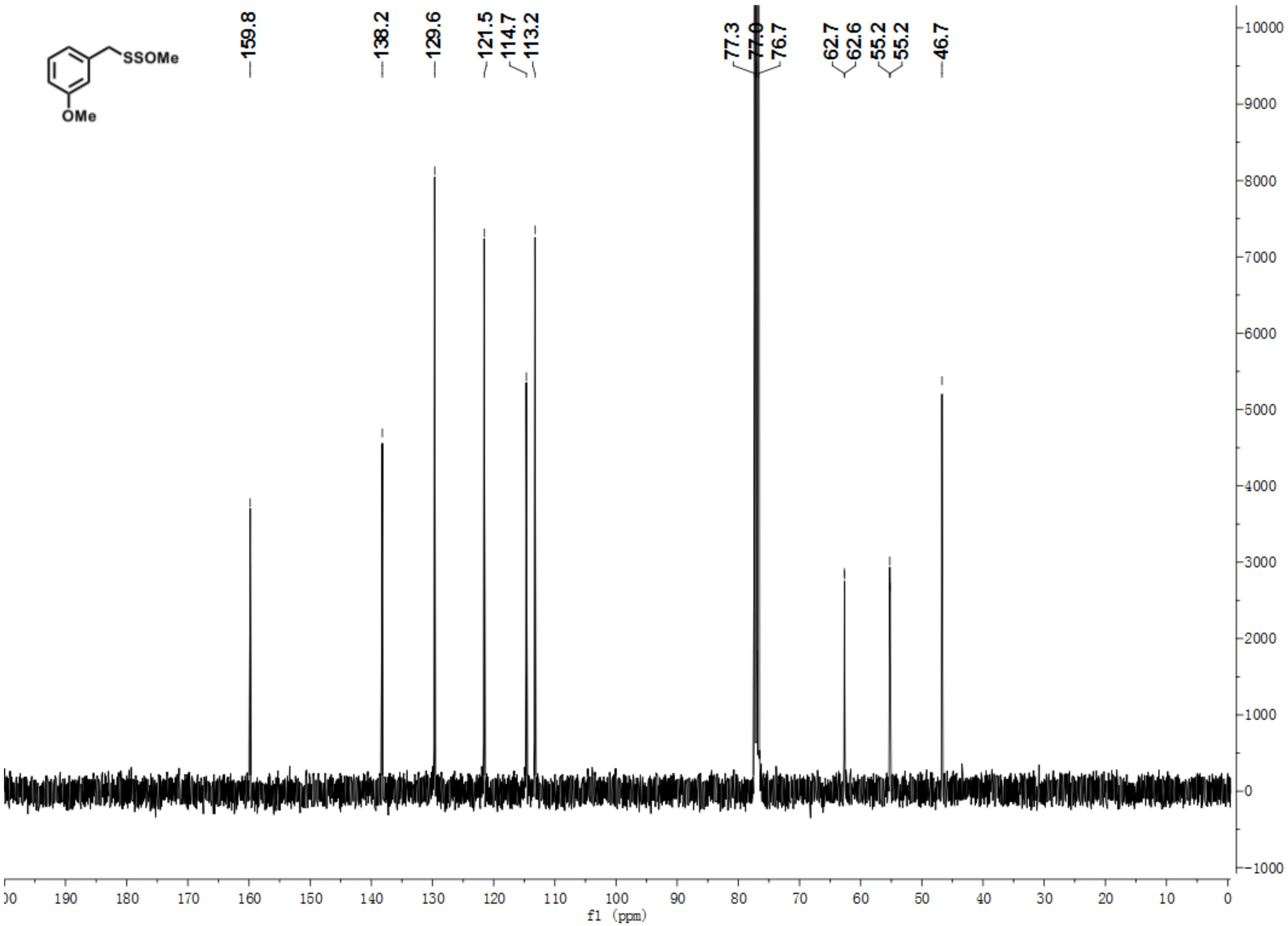

Supplementary Figure 6. ^{13}C NMR spectra for **2a**.

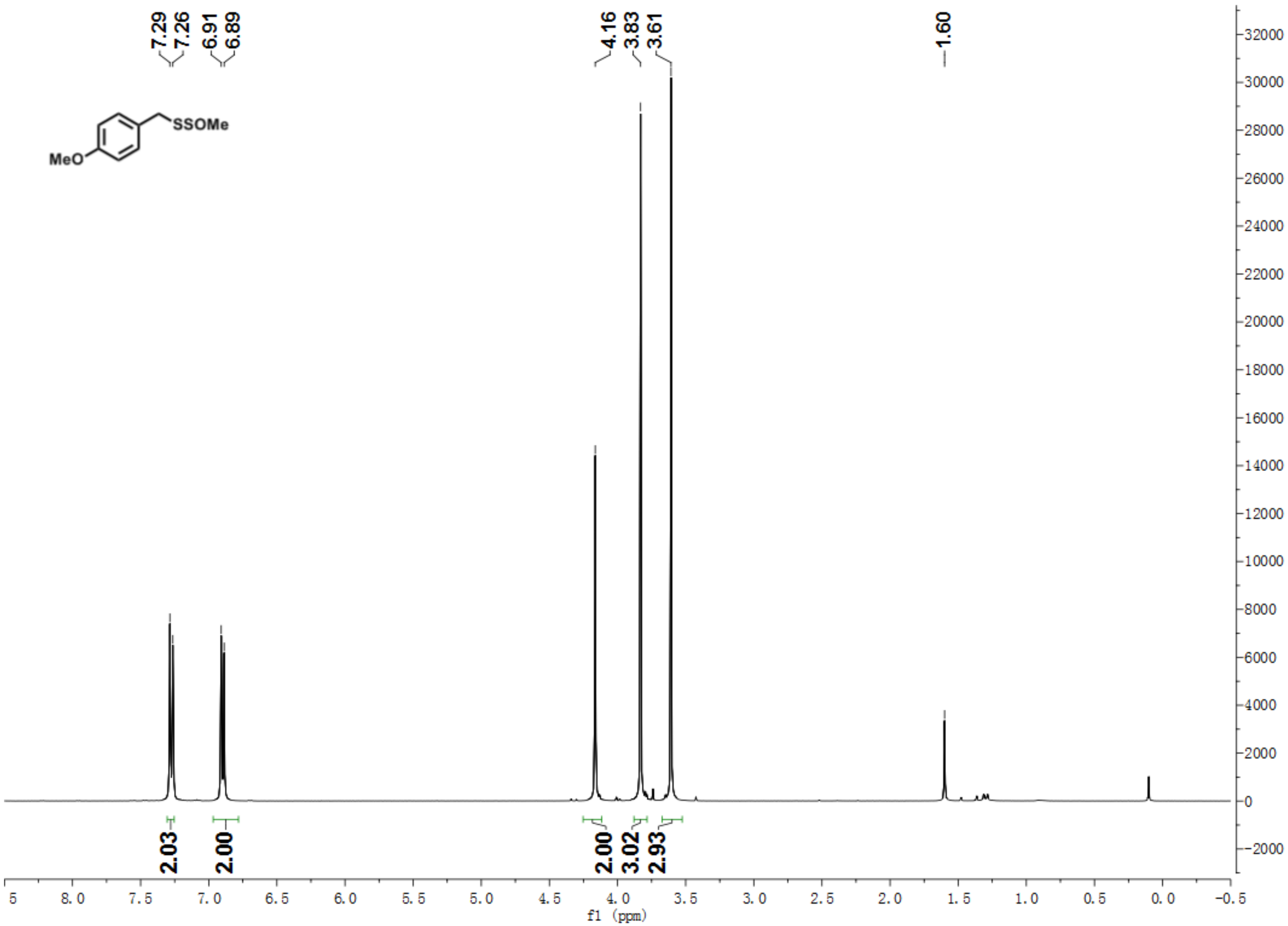

Supplementary Figure 7. ^{1}H NMR spectra for Compound **2b.**

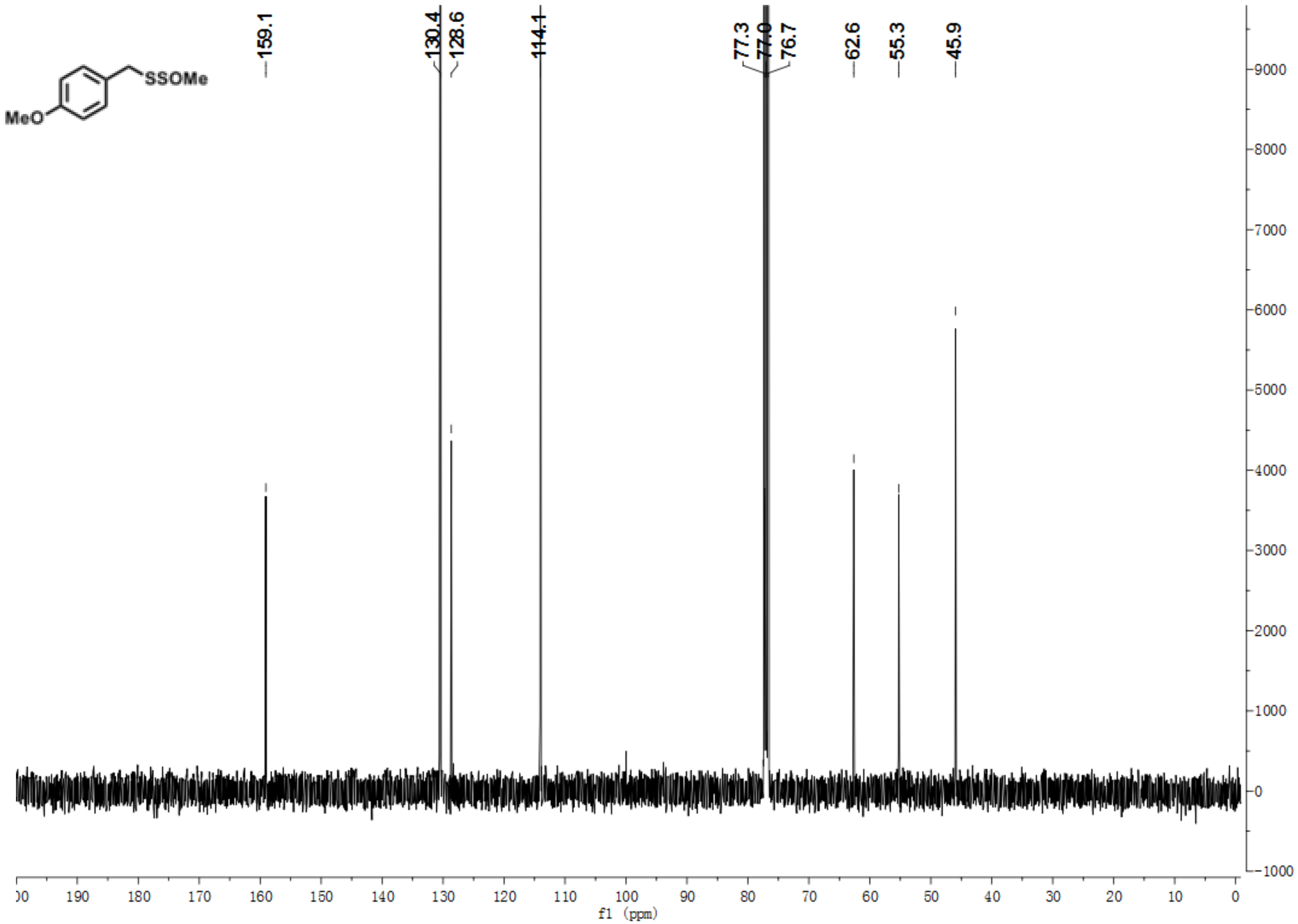

Supplementary Figure 8. ^{13}C NMR spectra for Compound **2b.**

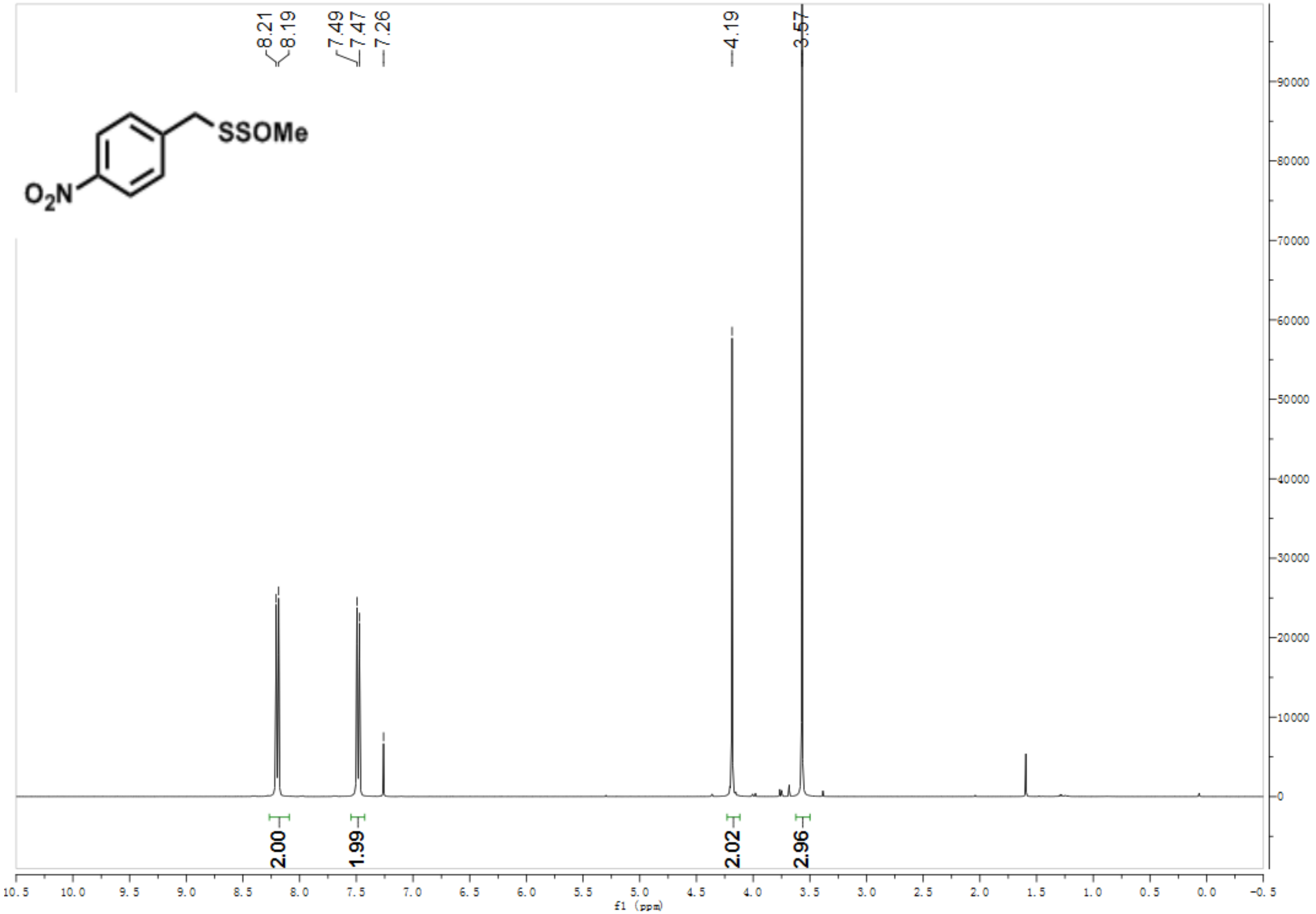

Supplementary Figure 9. ^{1}H NMR spectra for Compound **2c.**

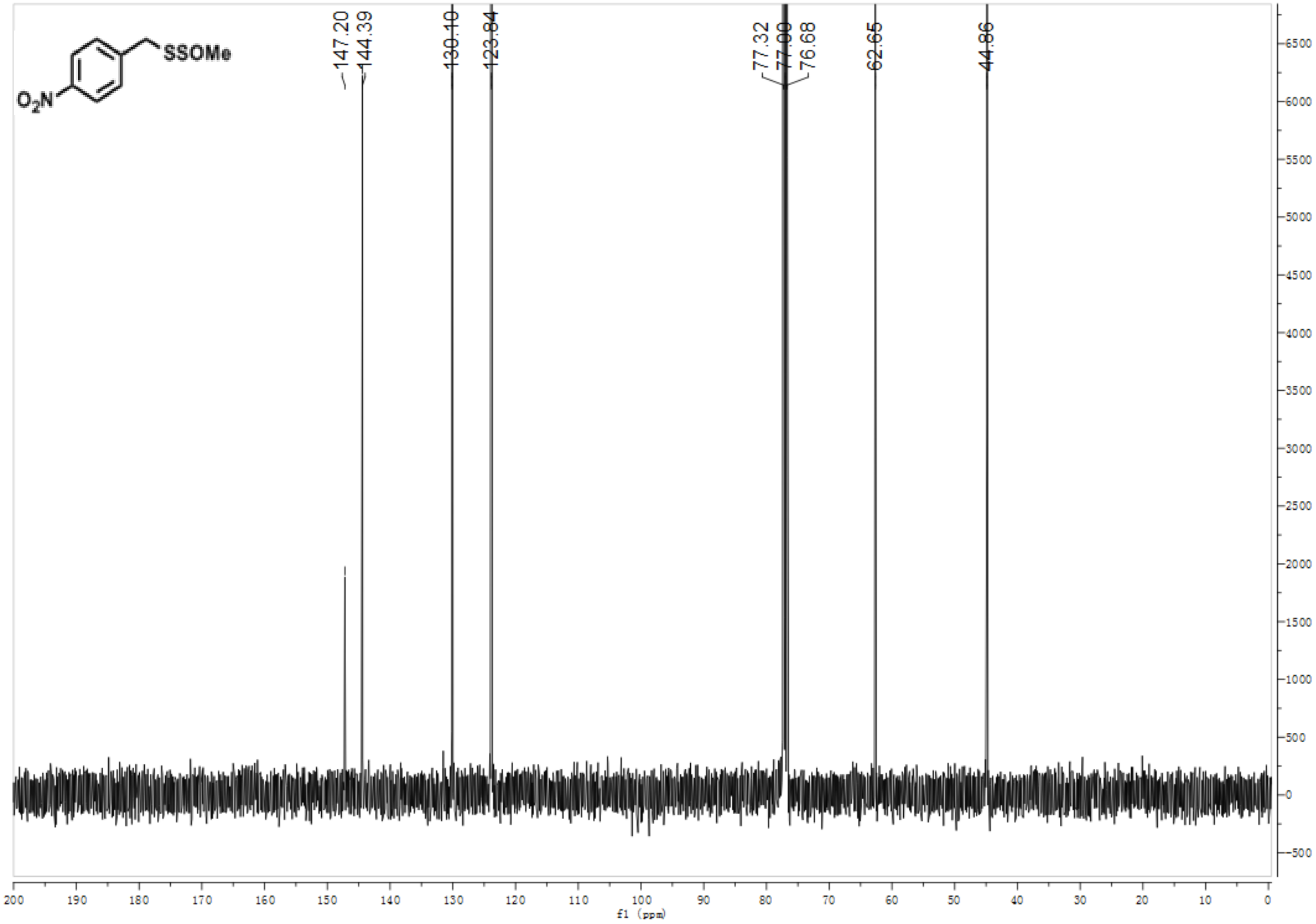

Supplementary Figure 10. ^{13}C NMR spectra for Compound **2c.**

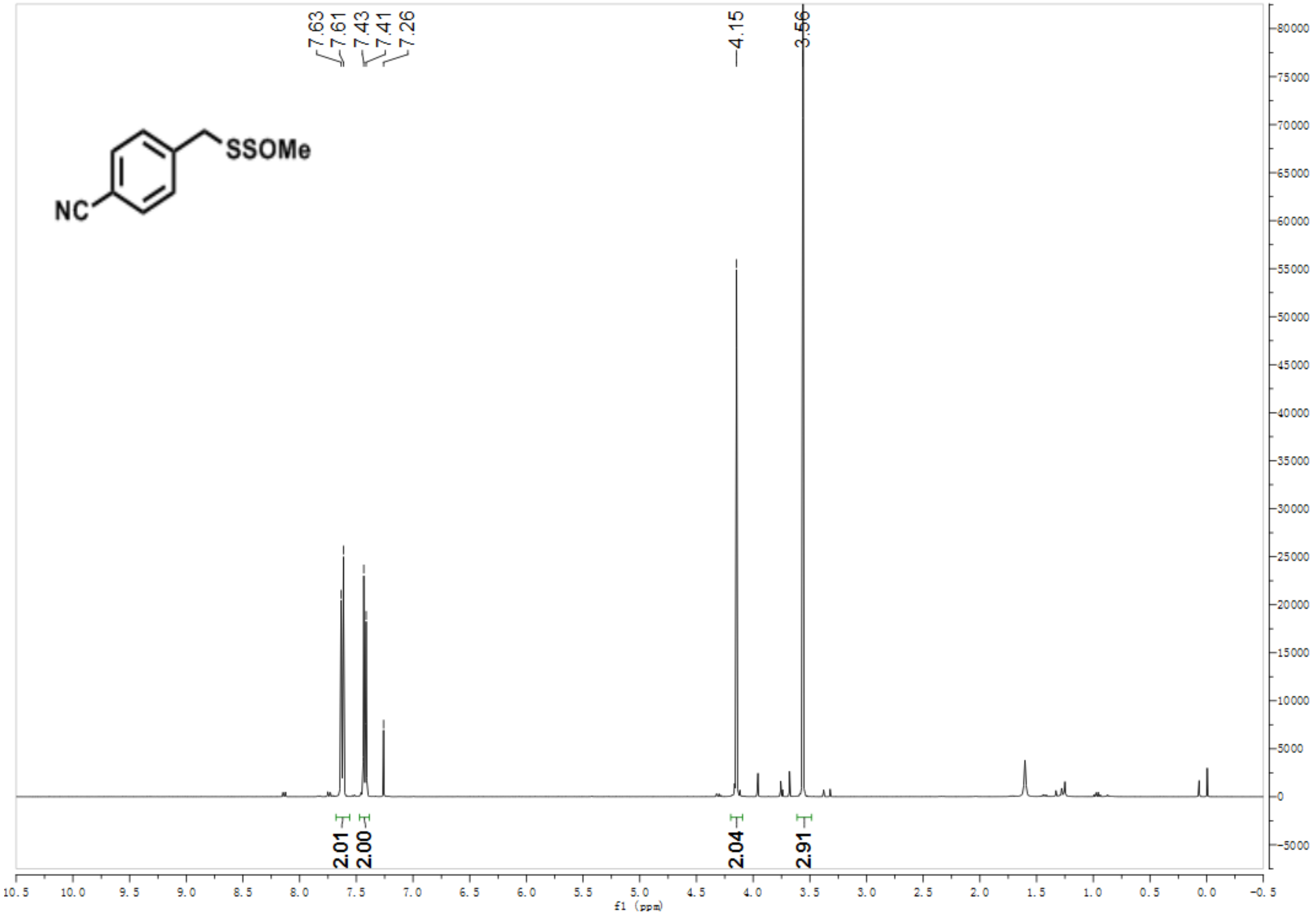

Supplementary Figure 11. ^{1}H NMR spectra for Compound **2d.**

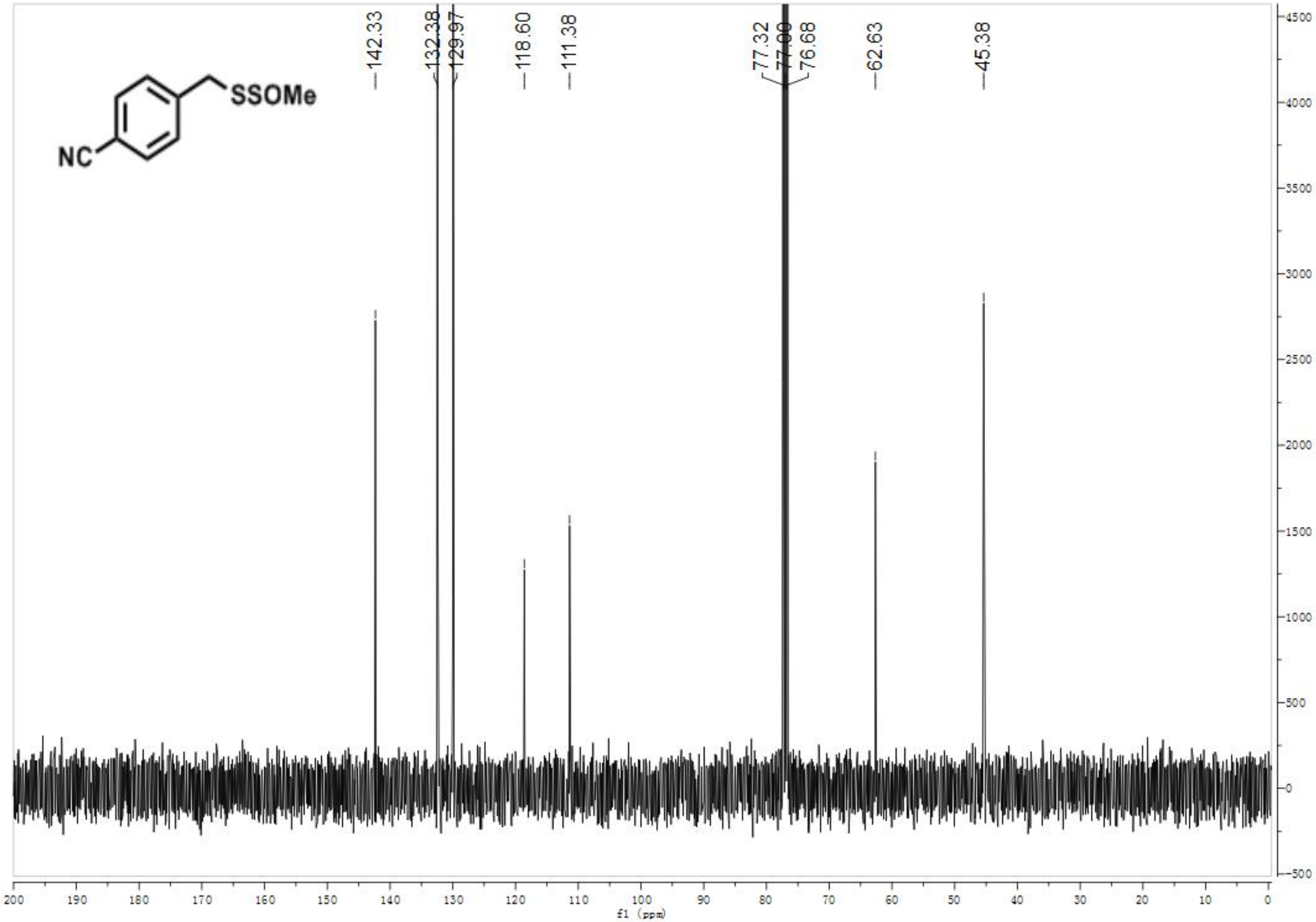

Supplementary Figure 12. ^{13}C NMR spectra for Compound **2d.**

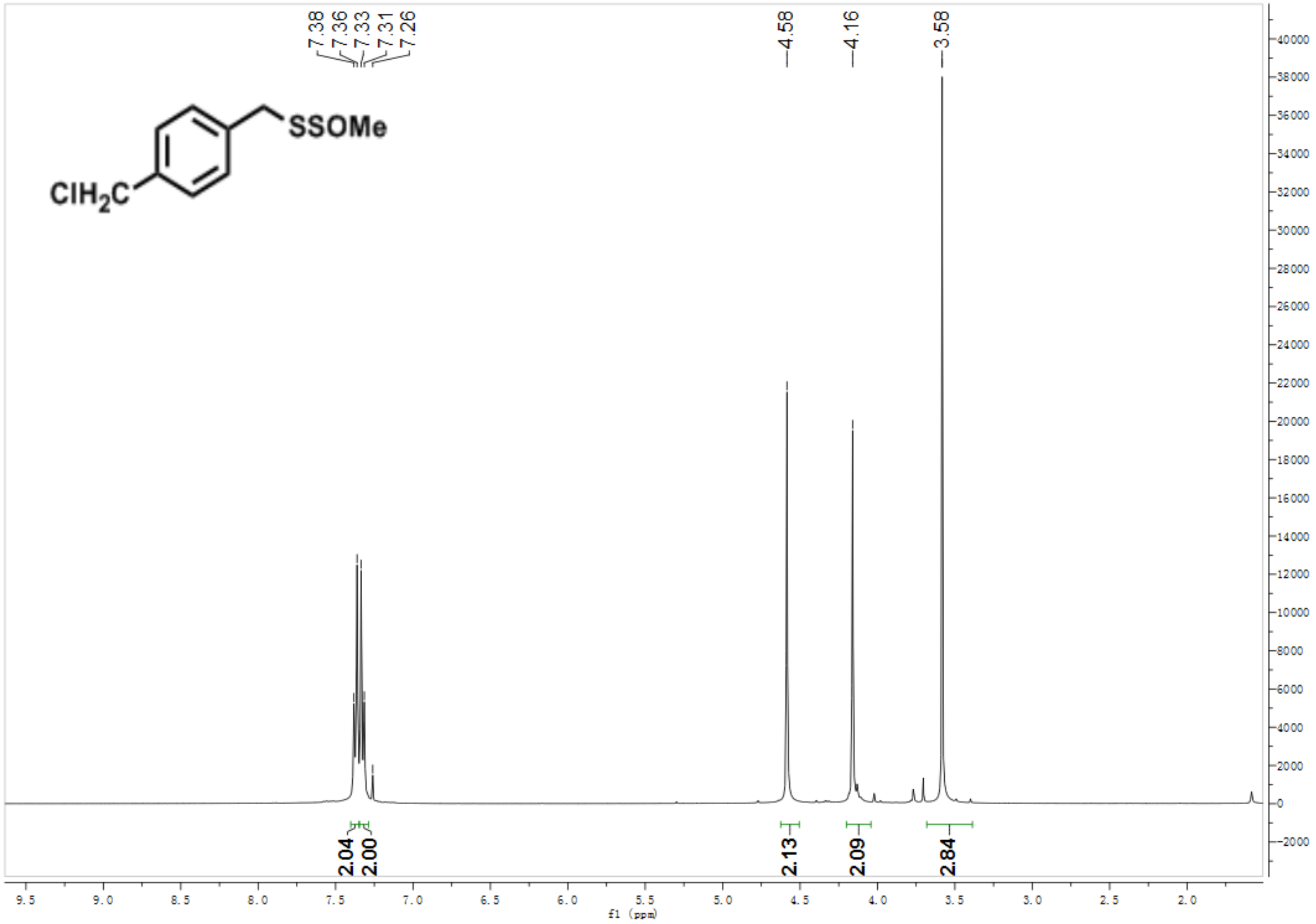

Supplementary Figure 13. ^{1}H NMR spectra for Compound **2e.**

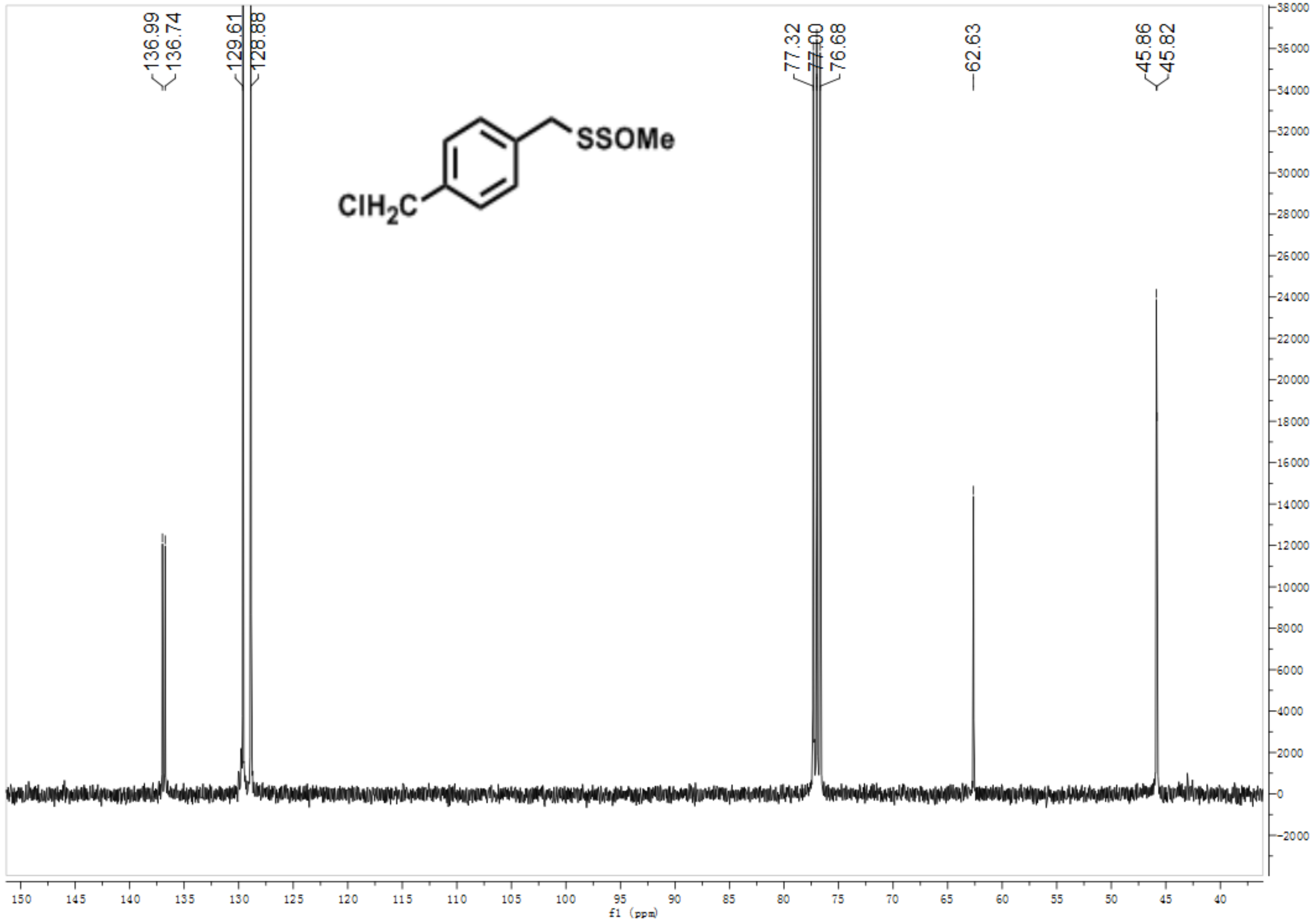

Supplementary Figure 14. ^{13}C NMR spectra for Compound **2e.**

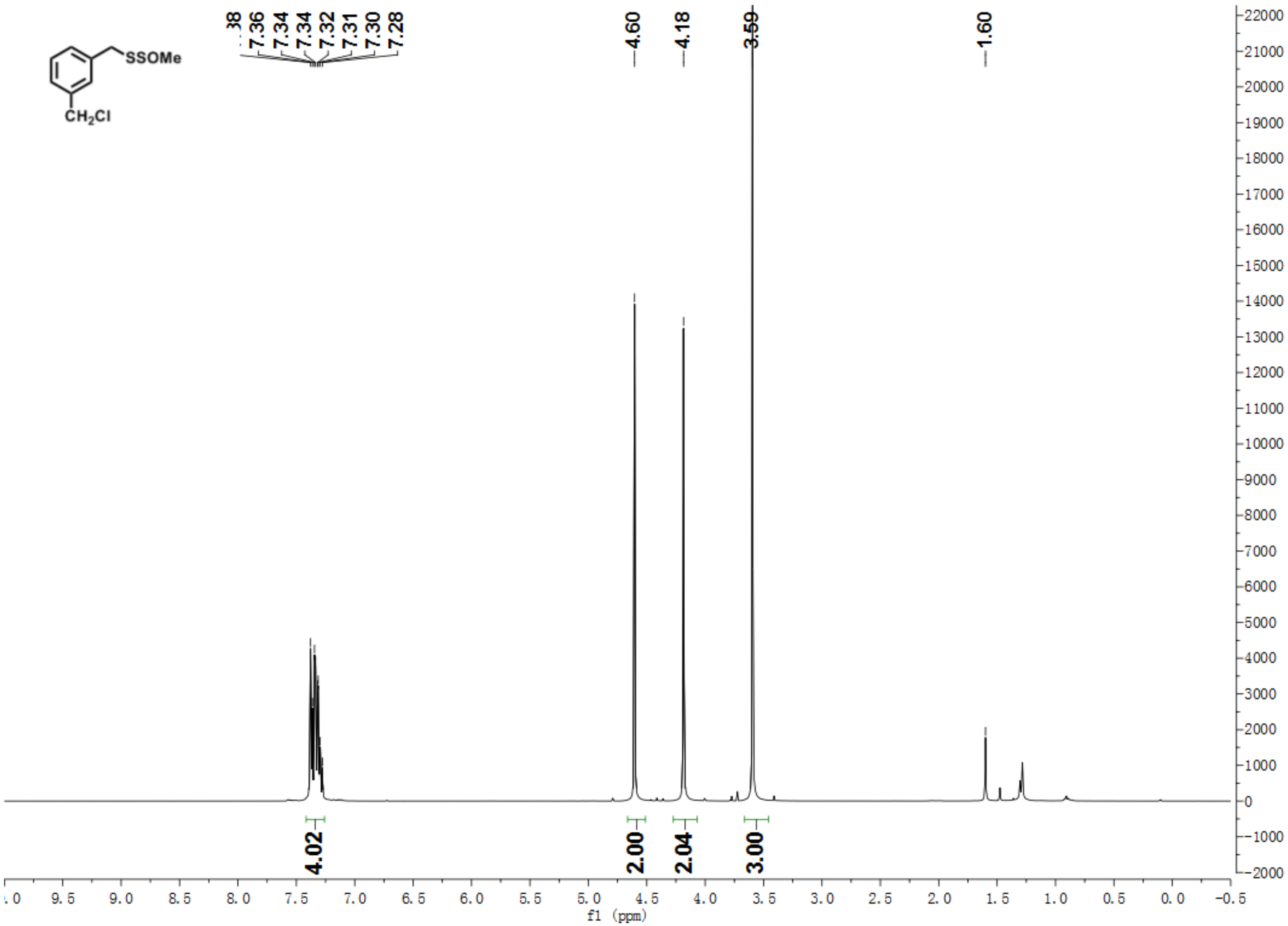

Supplementary Figure 15. ^{1}H NMR spectra for Compound **2f.**

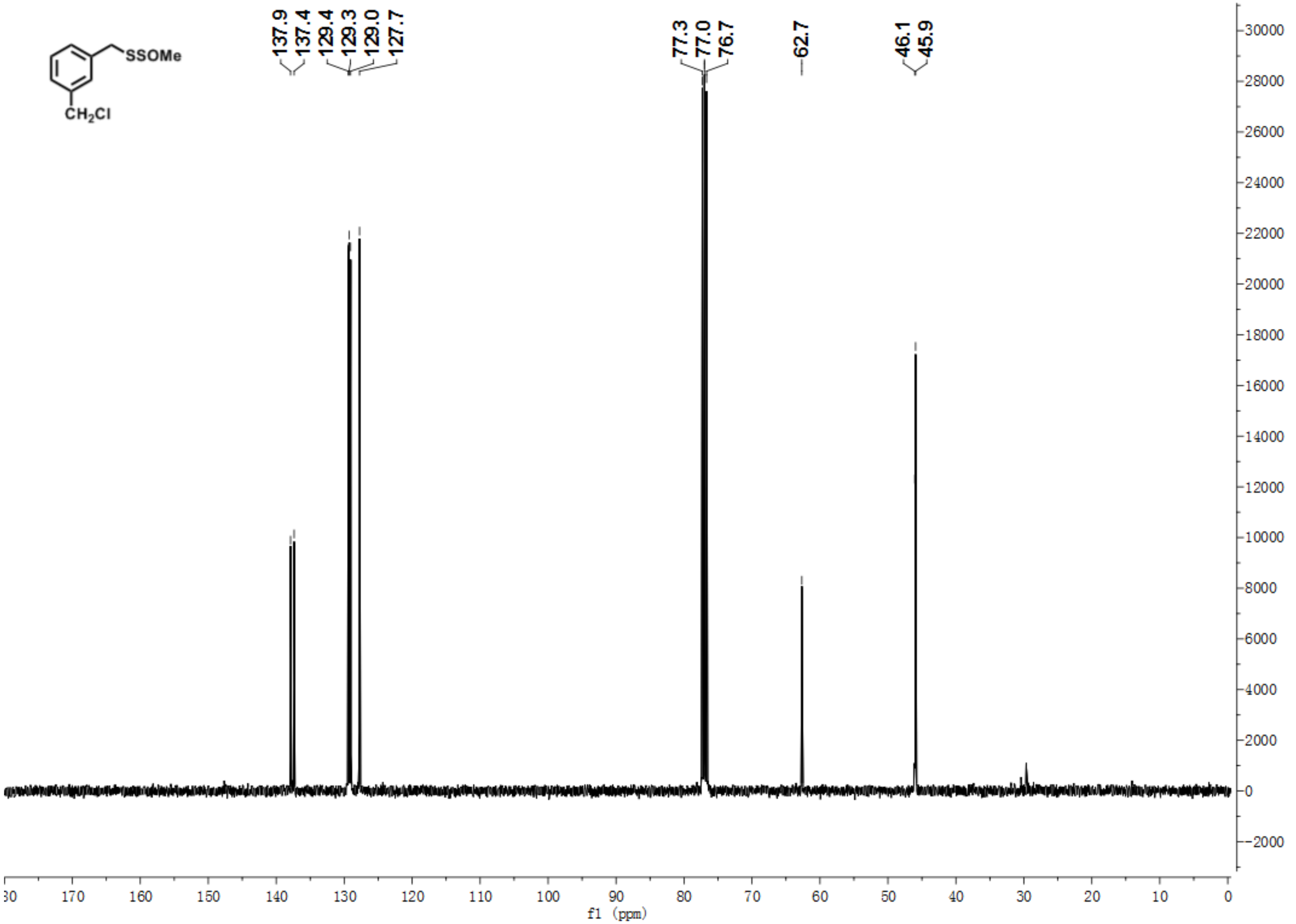

Supplementary Figure 16. ^{13}C NMR spectra for Compound **2f.**

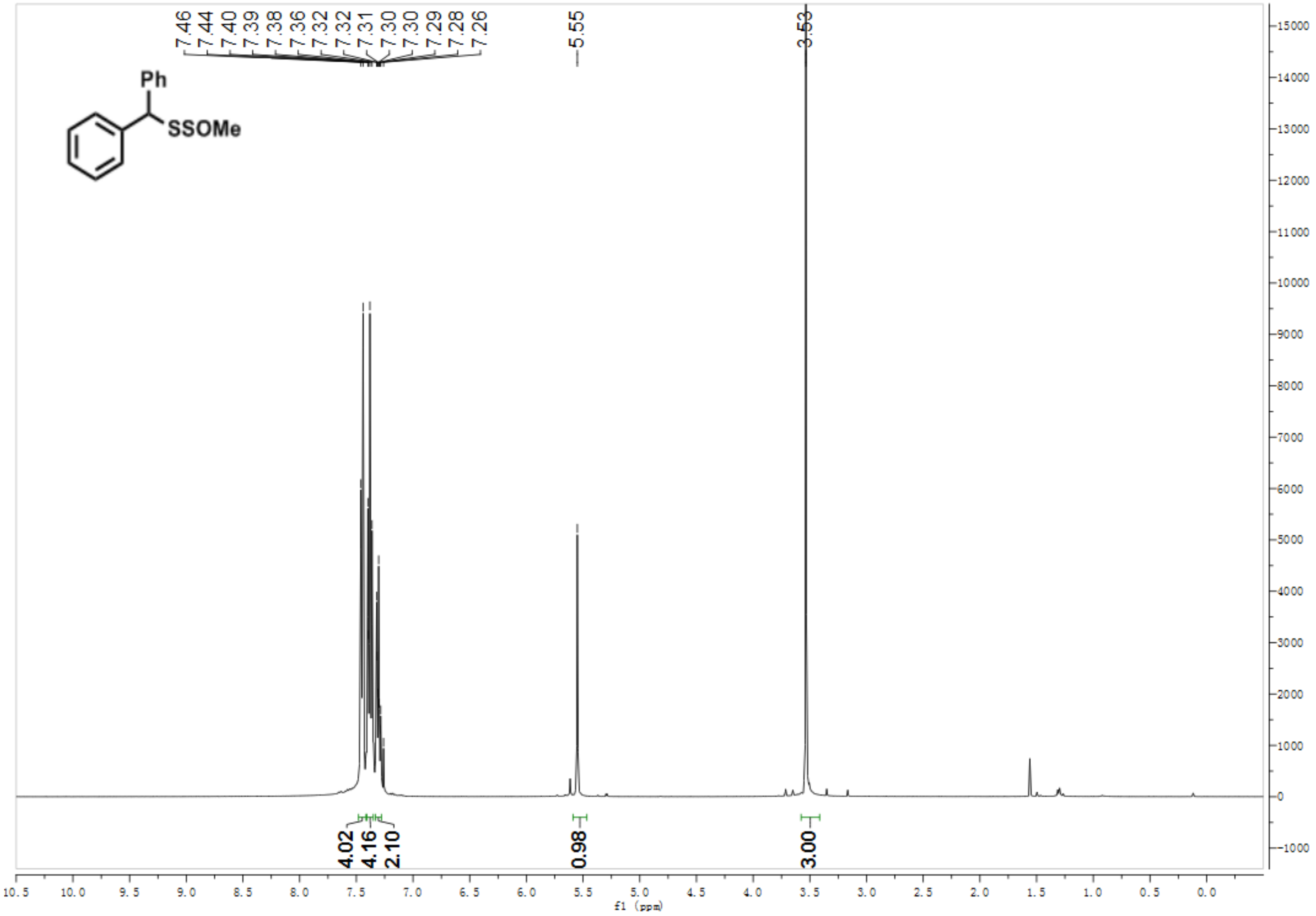

Supplementary Figure 17. ^{1}H NMR spectra for Compound **2g.**

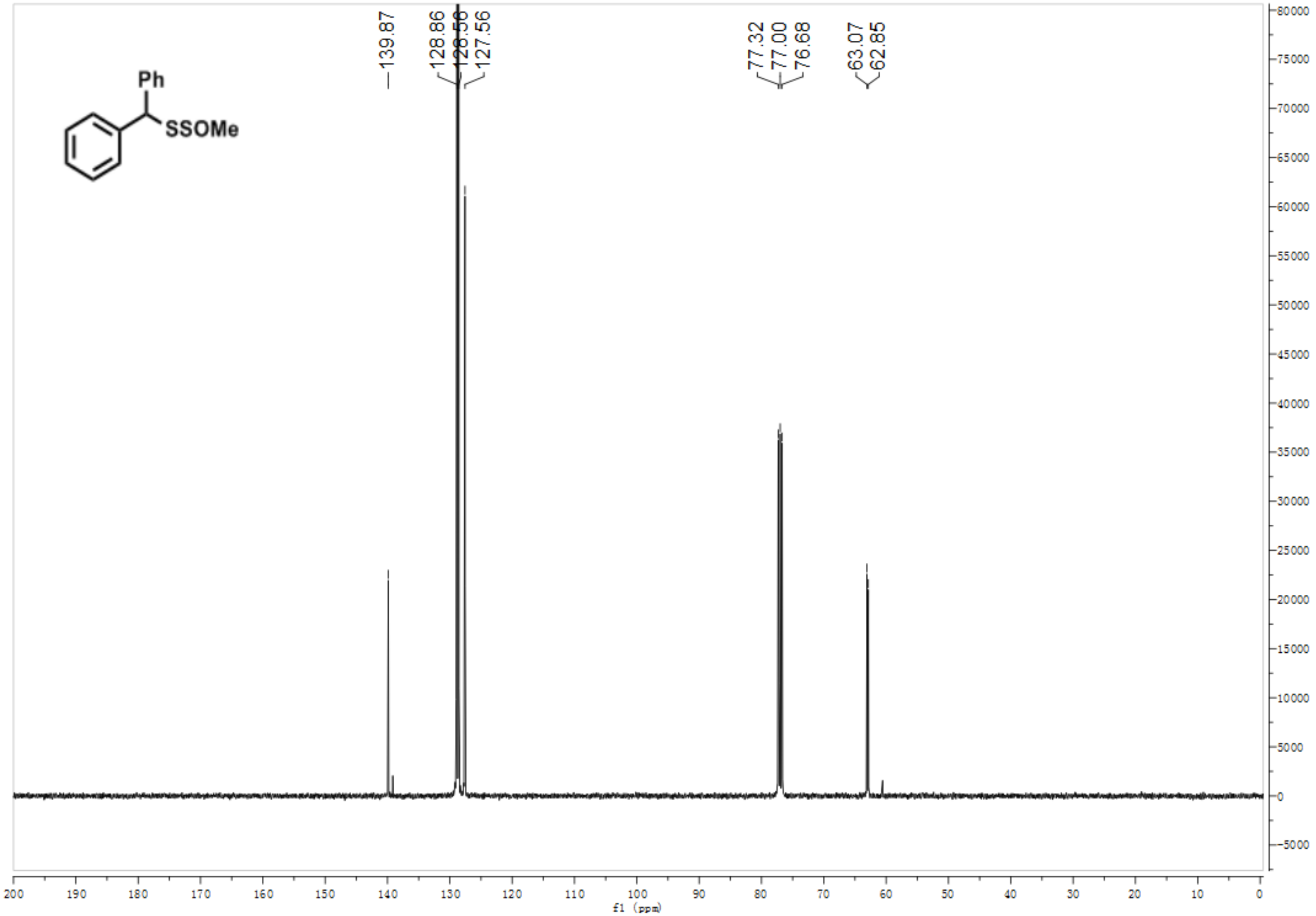

Supplementary Figure 18. ^{13}C NMR spectra for Compound **2g.**

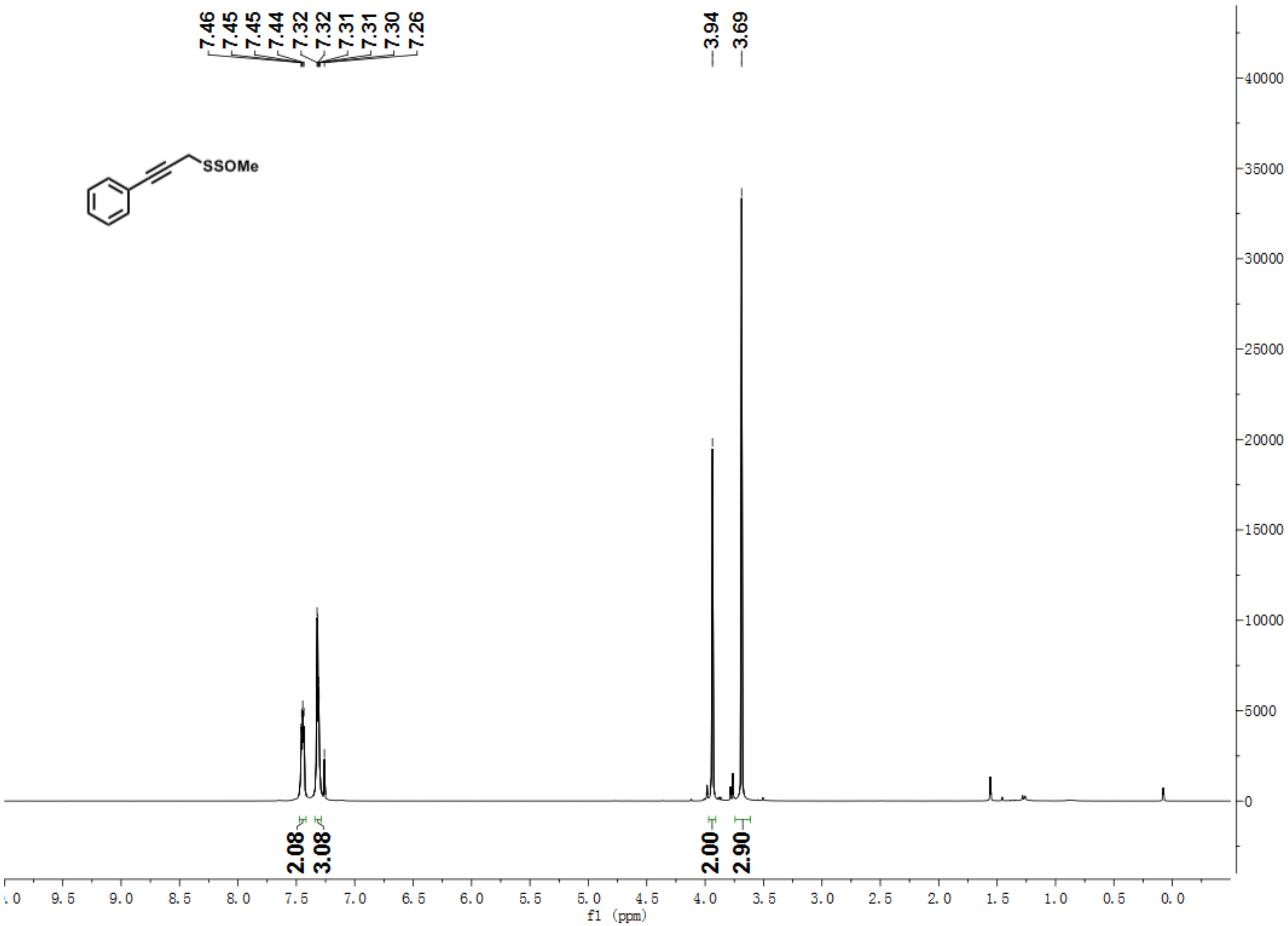

Supplementary Figure 19. ^{1}H NMR spectra for Compound **2h.**

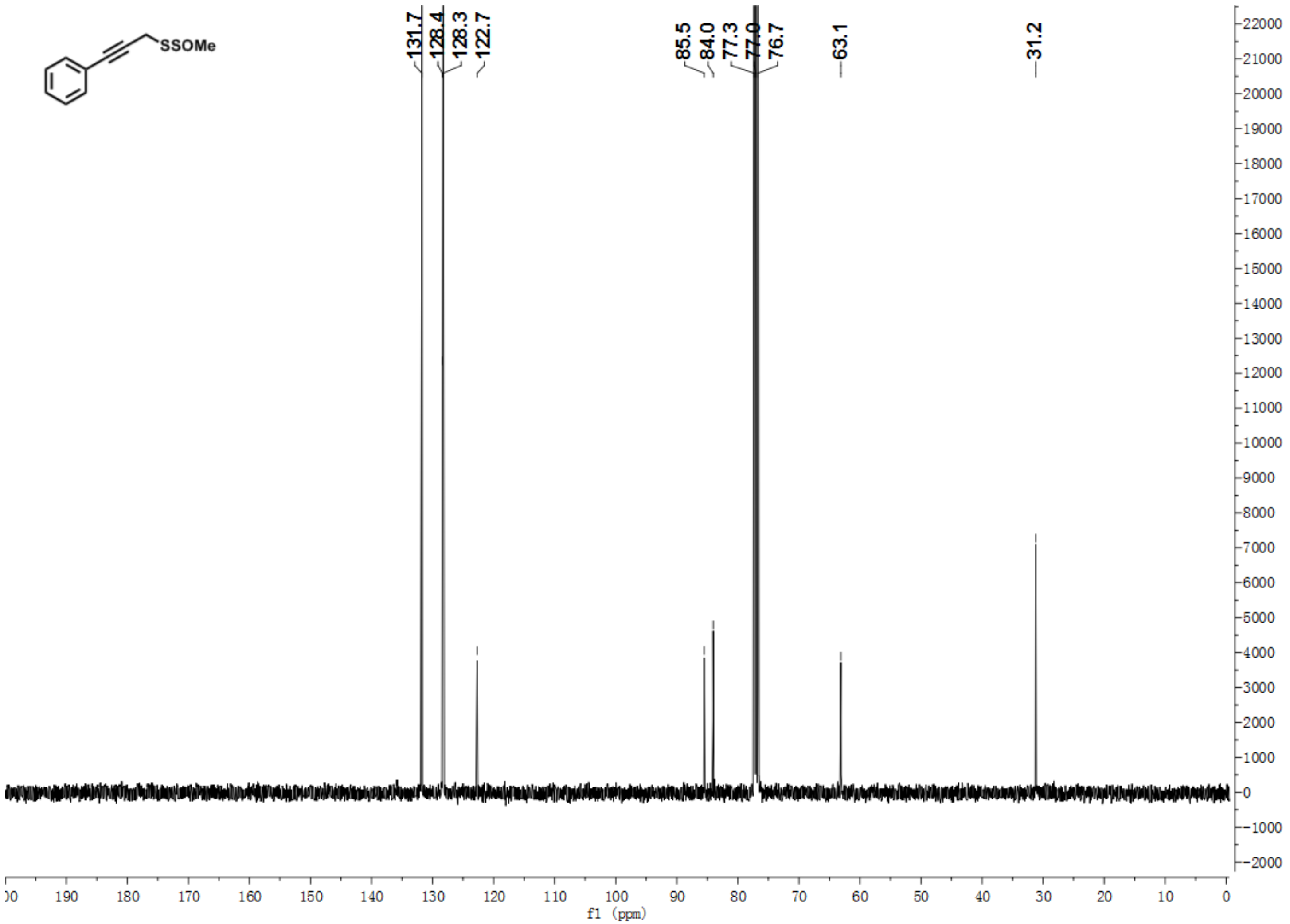

Supplementary Figure 20. ^{13}C NMR spectra for Compound **2h.**

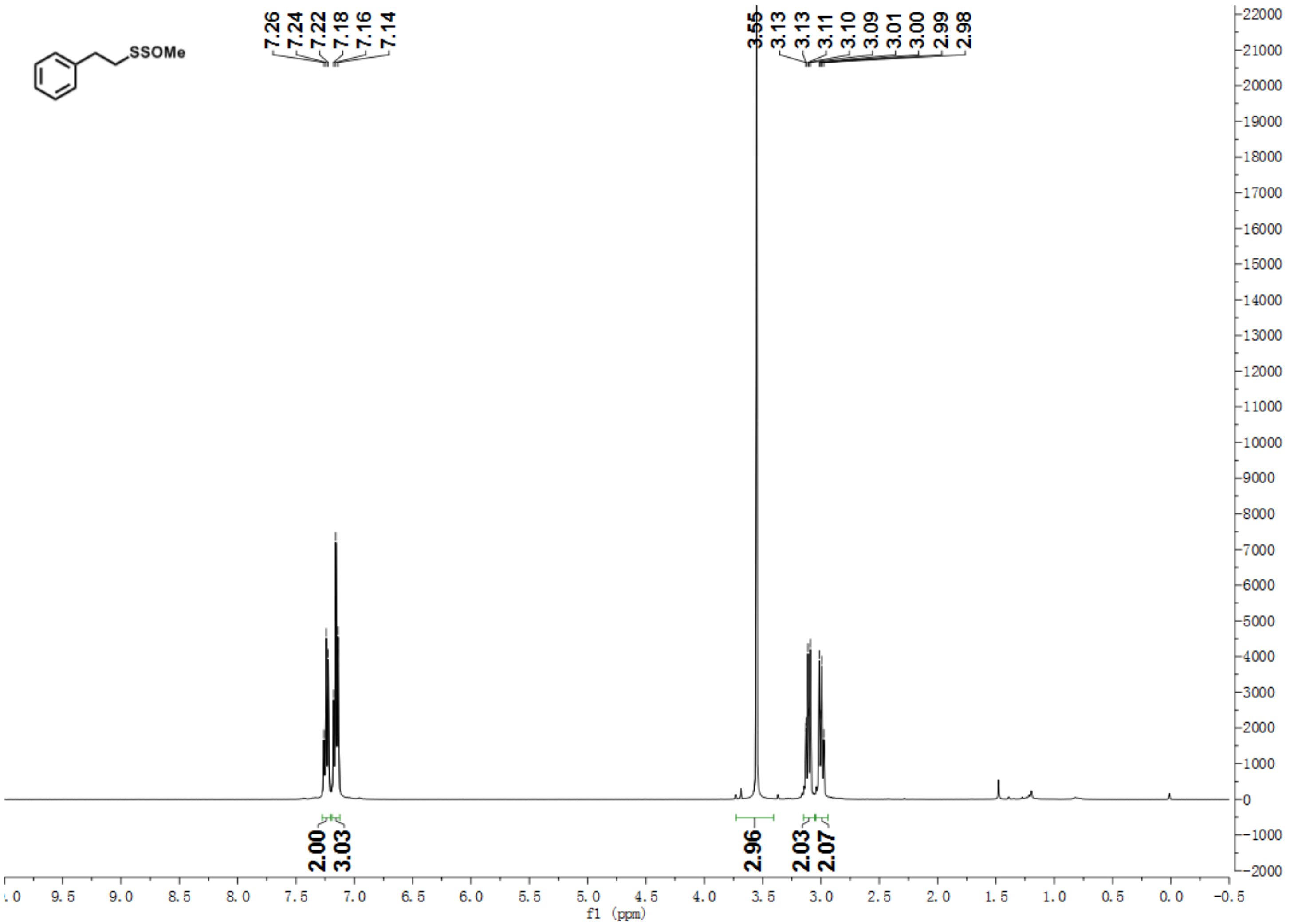

Supplementary Figure 21. ^{1}H NMR spectra for Compound **2i.**

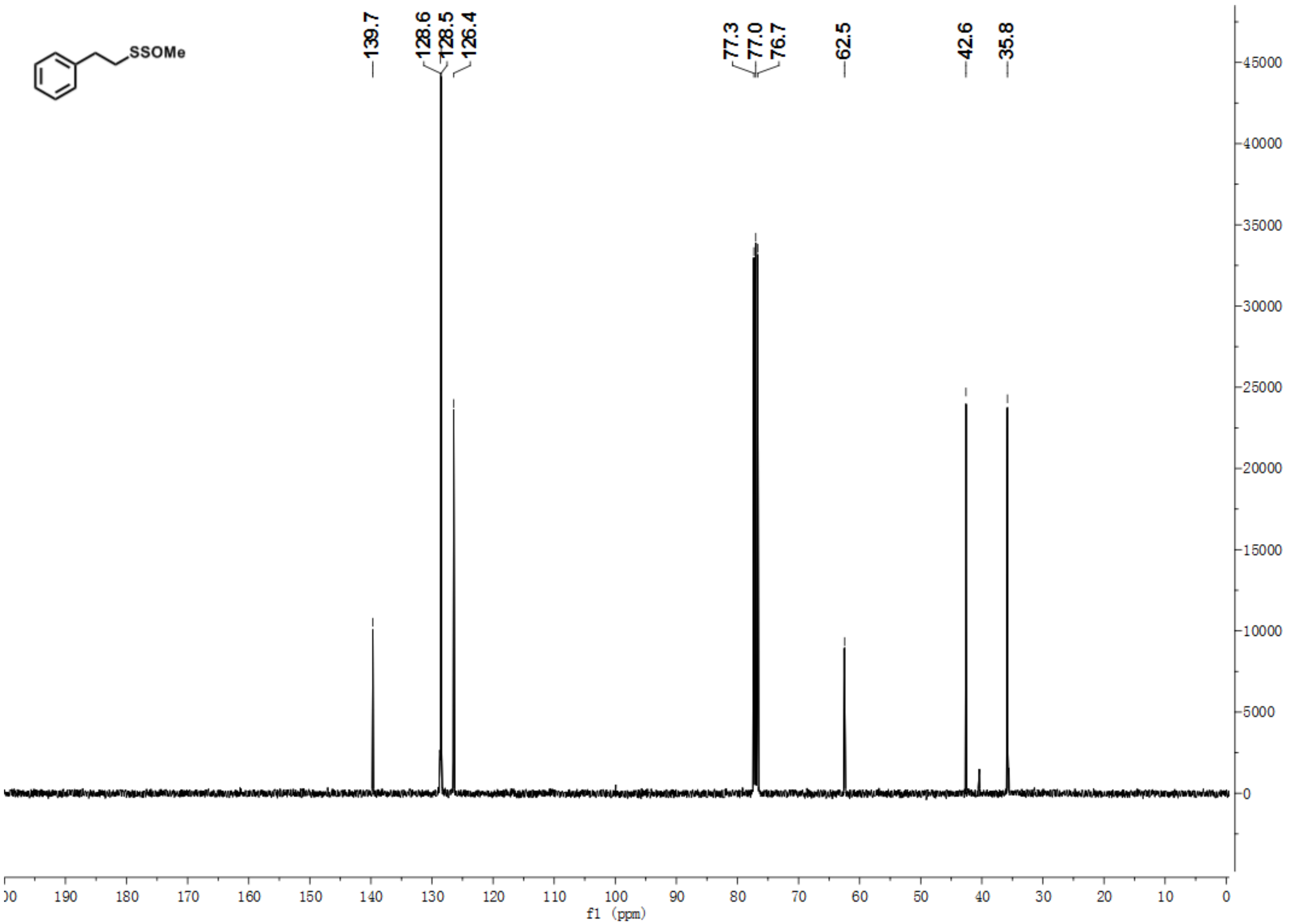

Supplementary Figure 22. ^{13}C NMR spectra for Compound **2i**.

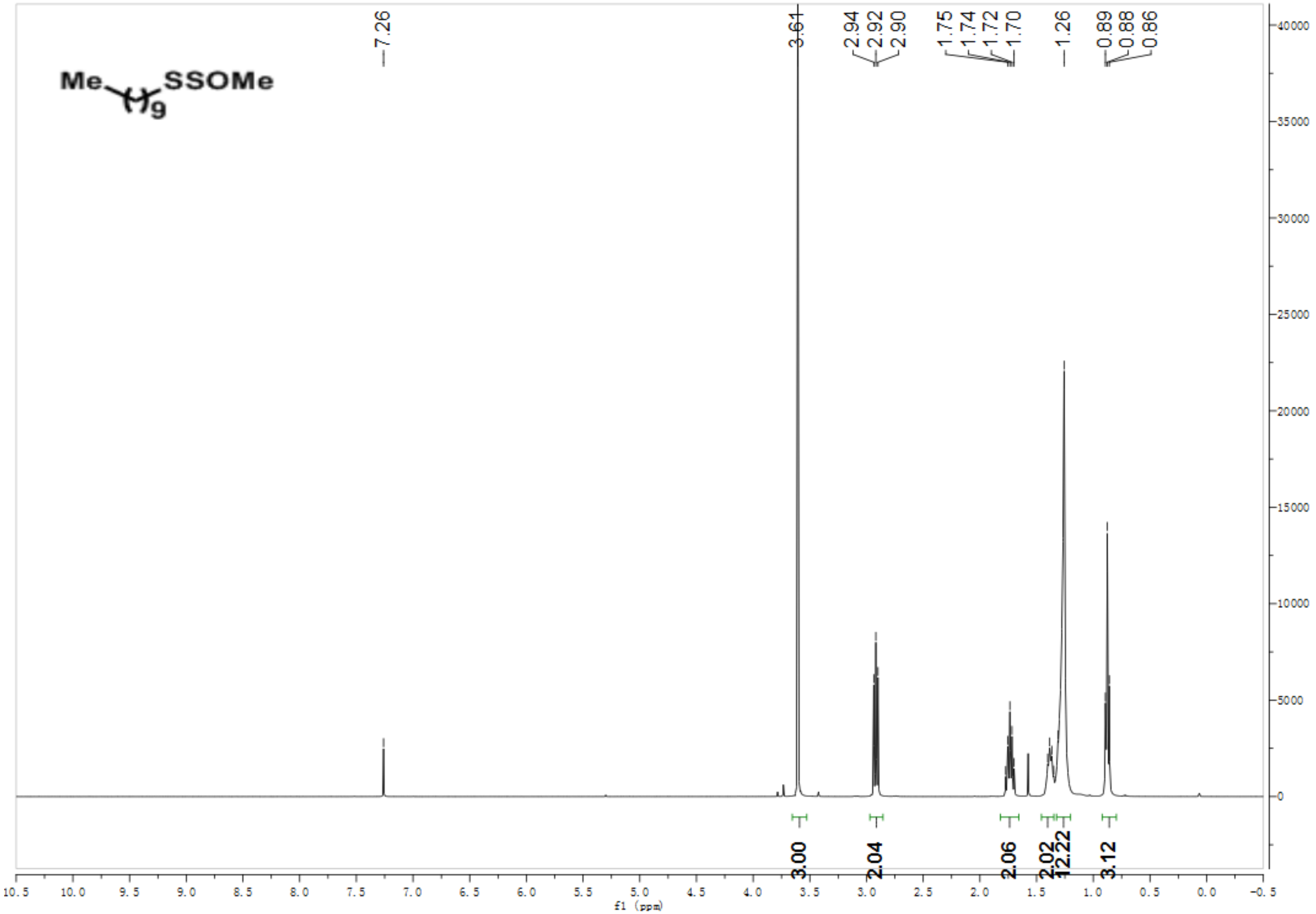

Supplementary Figure 23. ^{1}H NMR spectra for Compound **2j**.

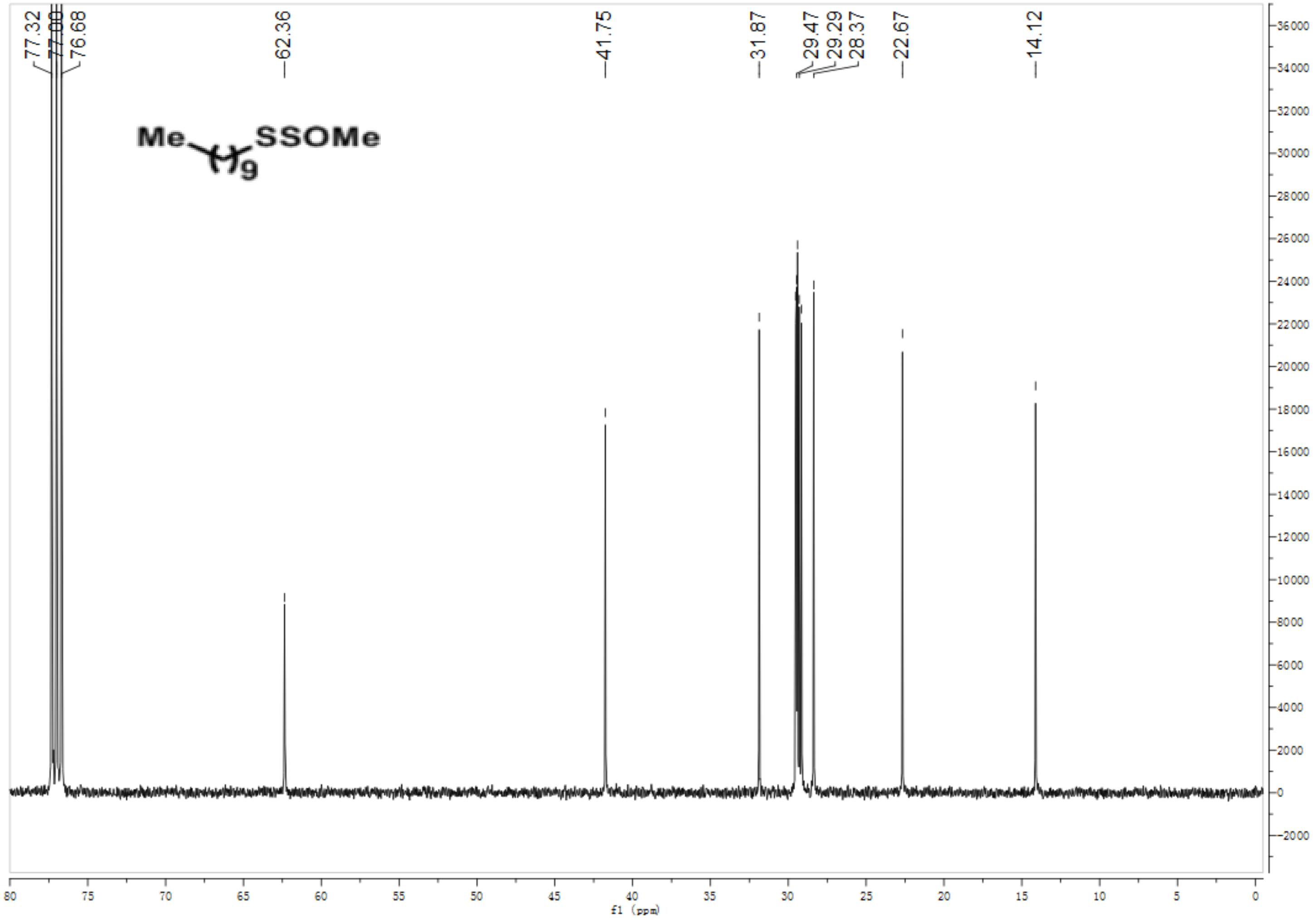

Supplementary Figure 24. ^{13}C NMR spectra for Compound **2j**.

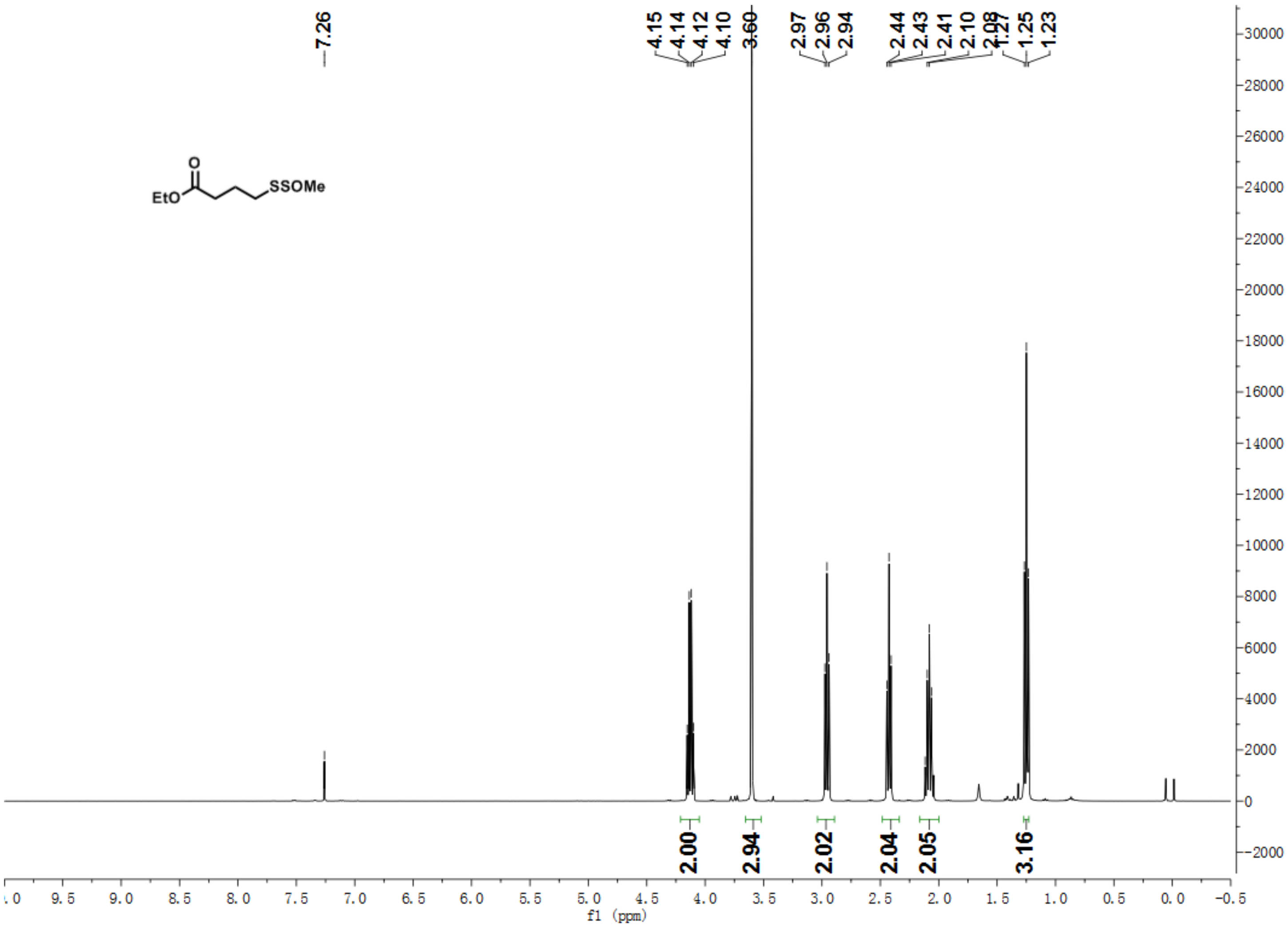

Supplementary Figure 25. ^{1}H NMR spectra for Compound **2k**.

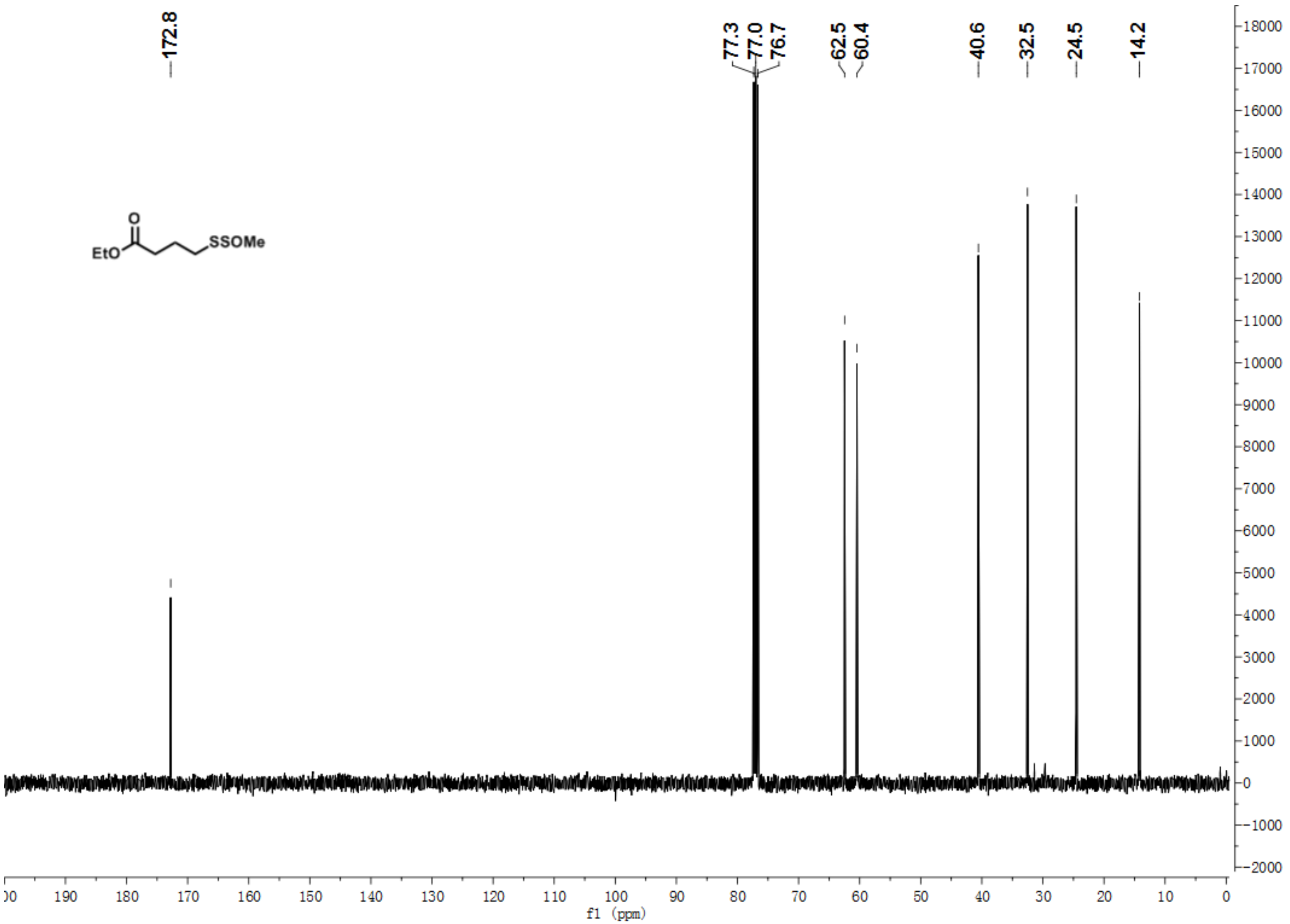

Supplementary Figure 26. ^{13}C NMR spectra for Compound **2k**.

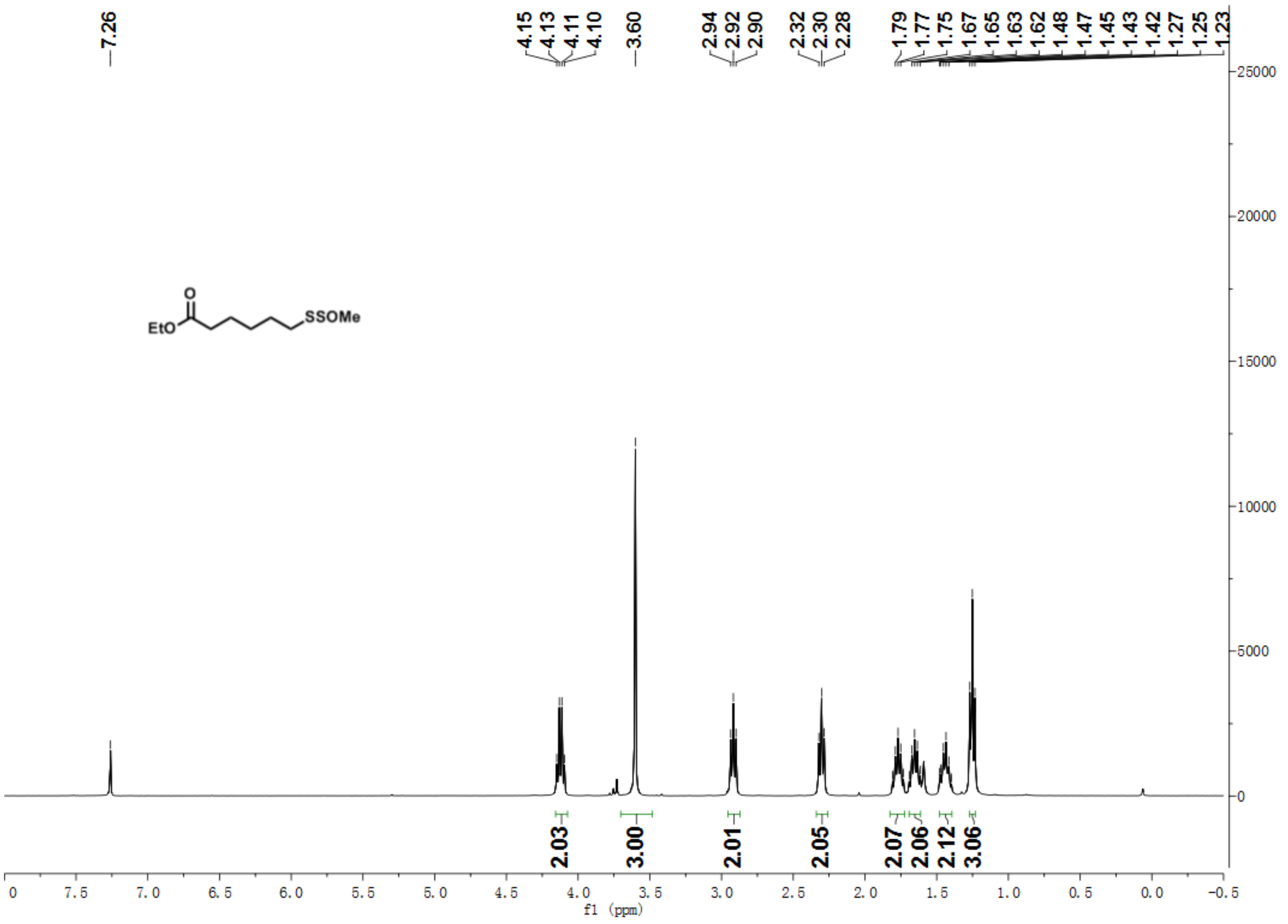

Supplementary Figure 27. ^{1}H NMR spectra for Compound **2l**.

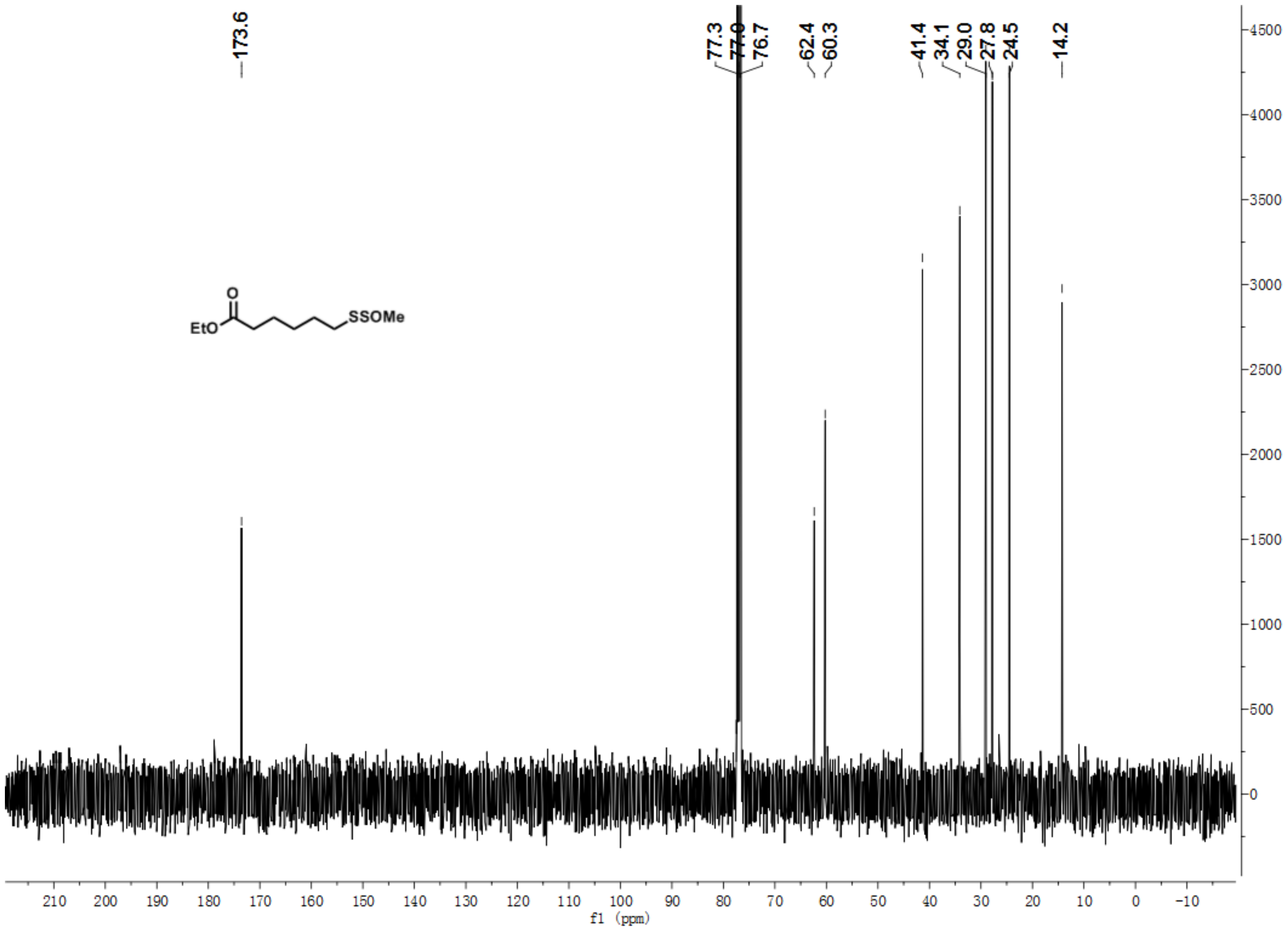

Supplementary Figure 28. ^{13}C NMR spectra for Compound **2l**.

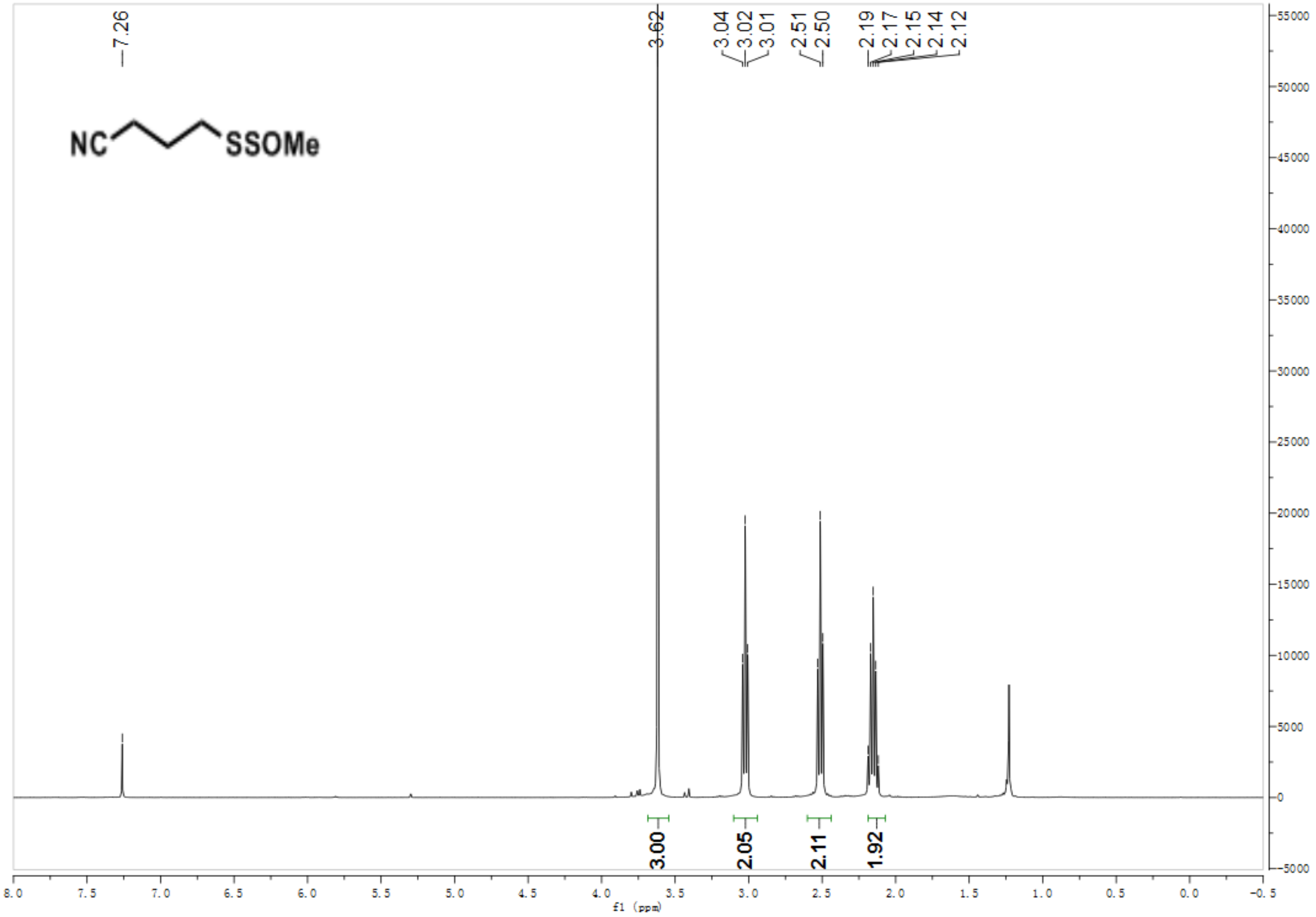

Supplementary Figure 29. ^{1}H NMR spectra for Compound **2m**.

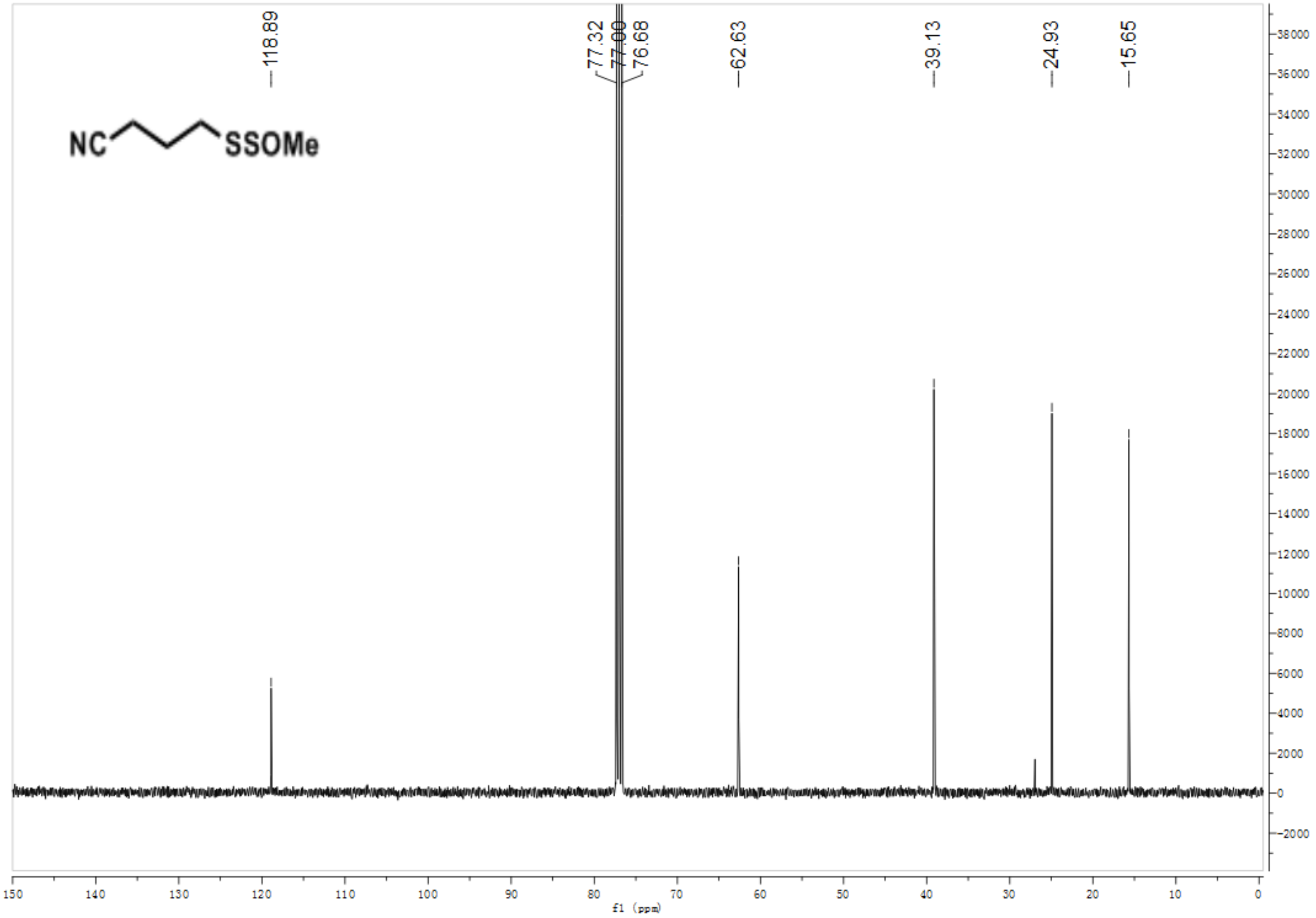

Supplementary Figure 30. ^{13}C NMR spectra for Compound **2m**.

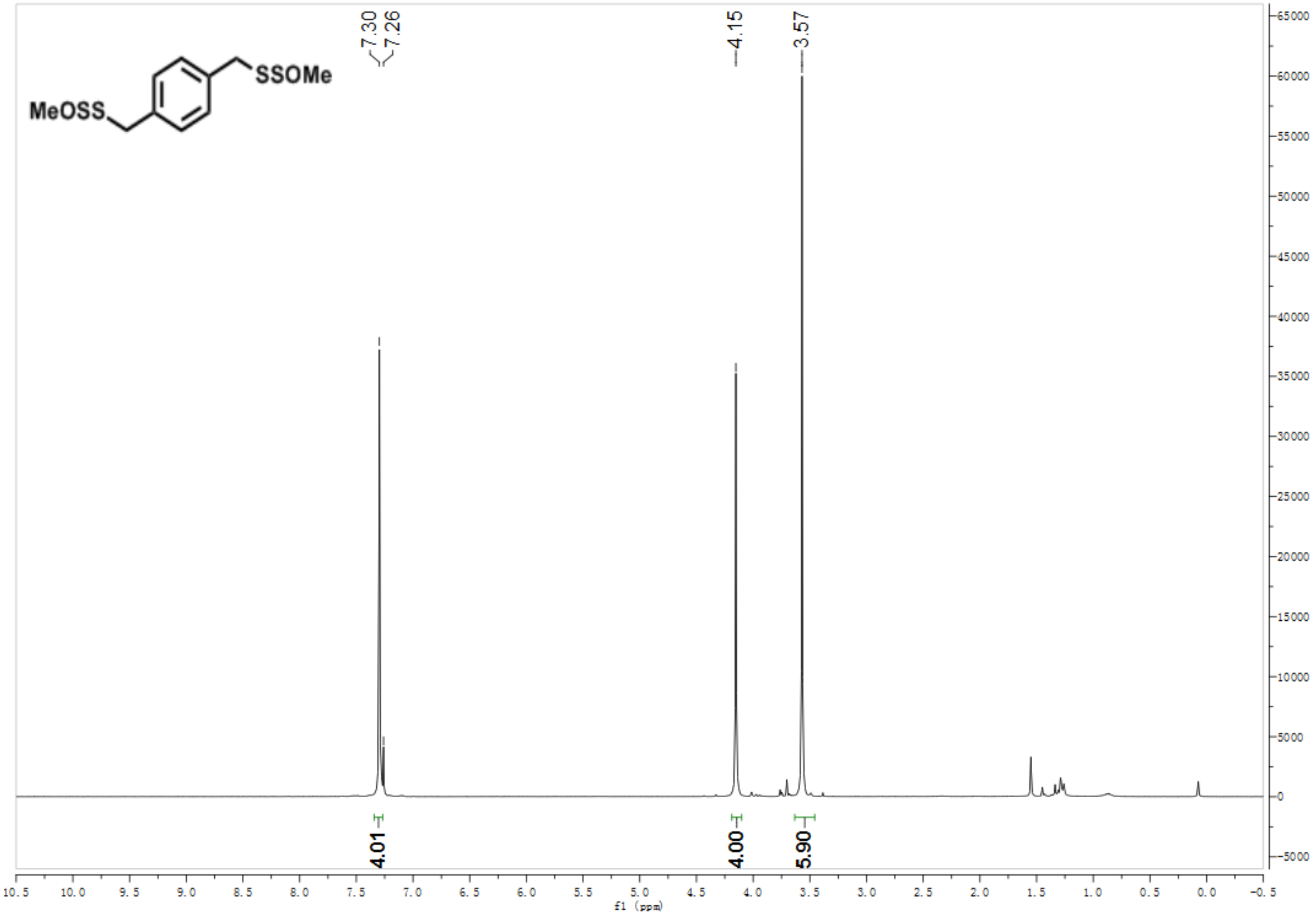

Supplementary Figure 31. ^{1}H NMR spectra for Compound **2n**.

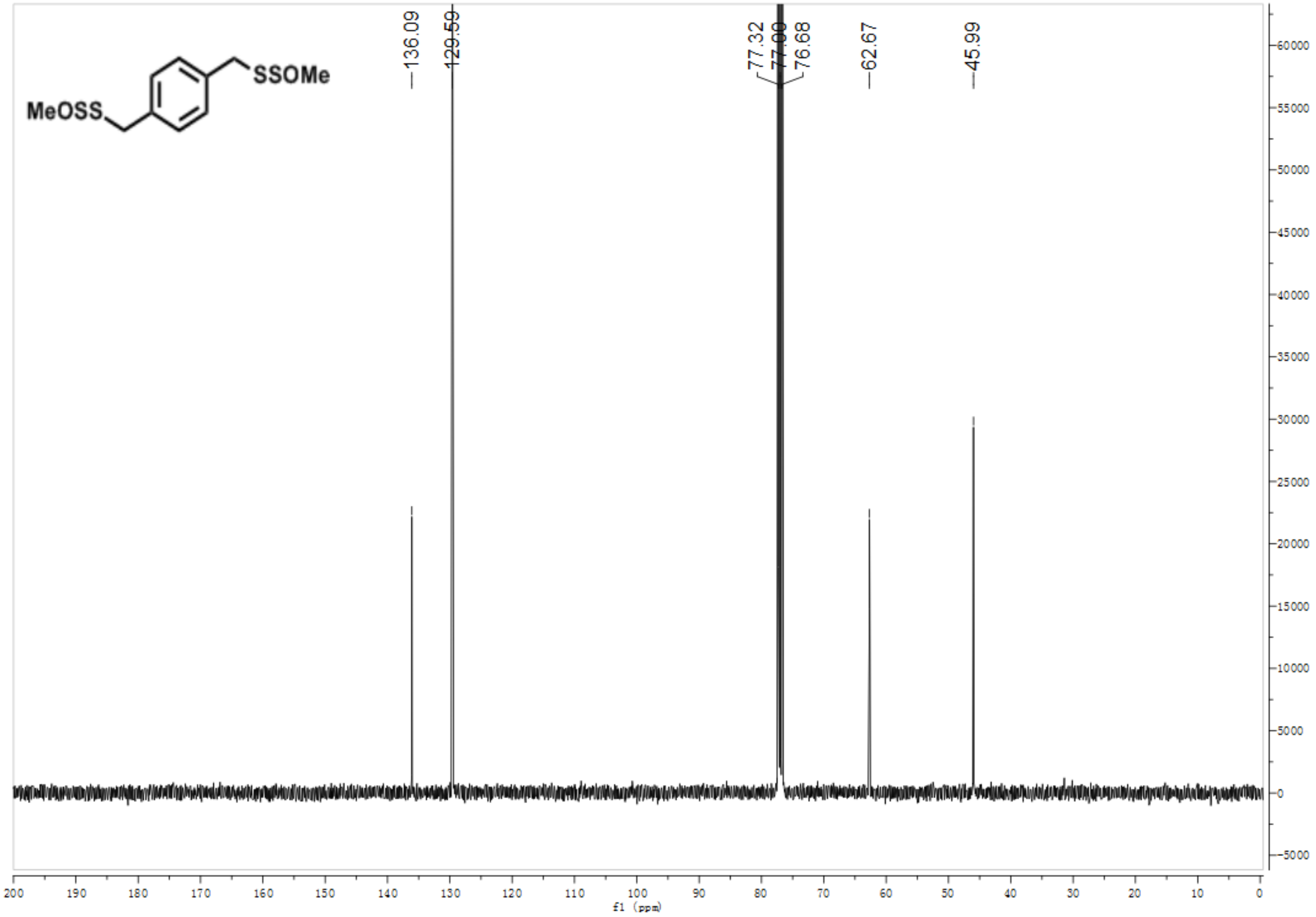

Supplementary Figure 32. ^{13}C NMR spectra for Compound **2n**.

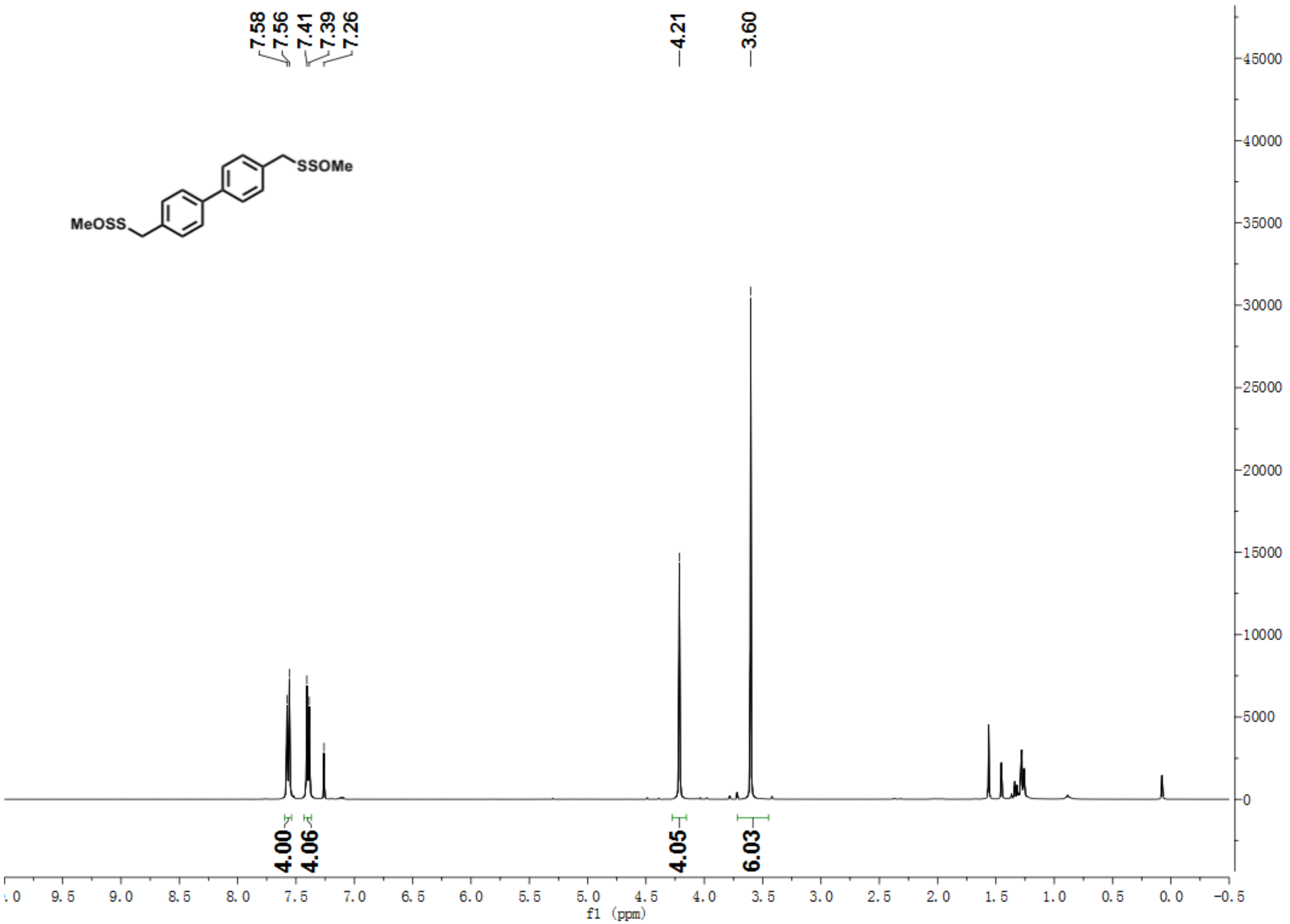

Supplementary Figure 33. ^{1}H NMR spectra for Compound **2o**.

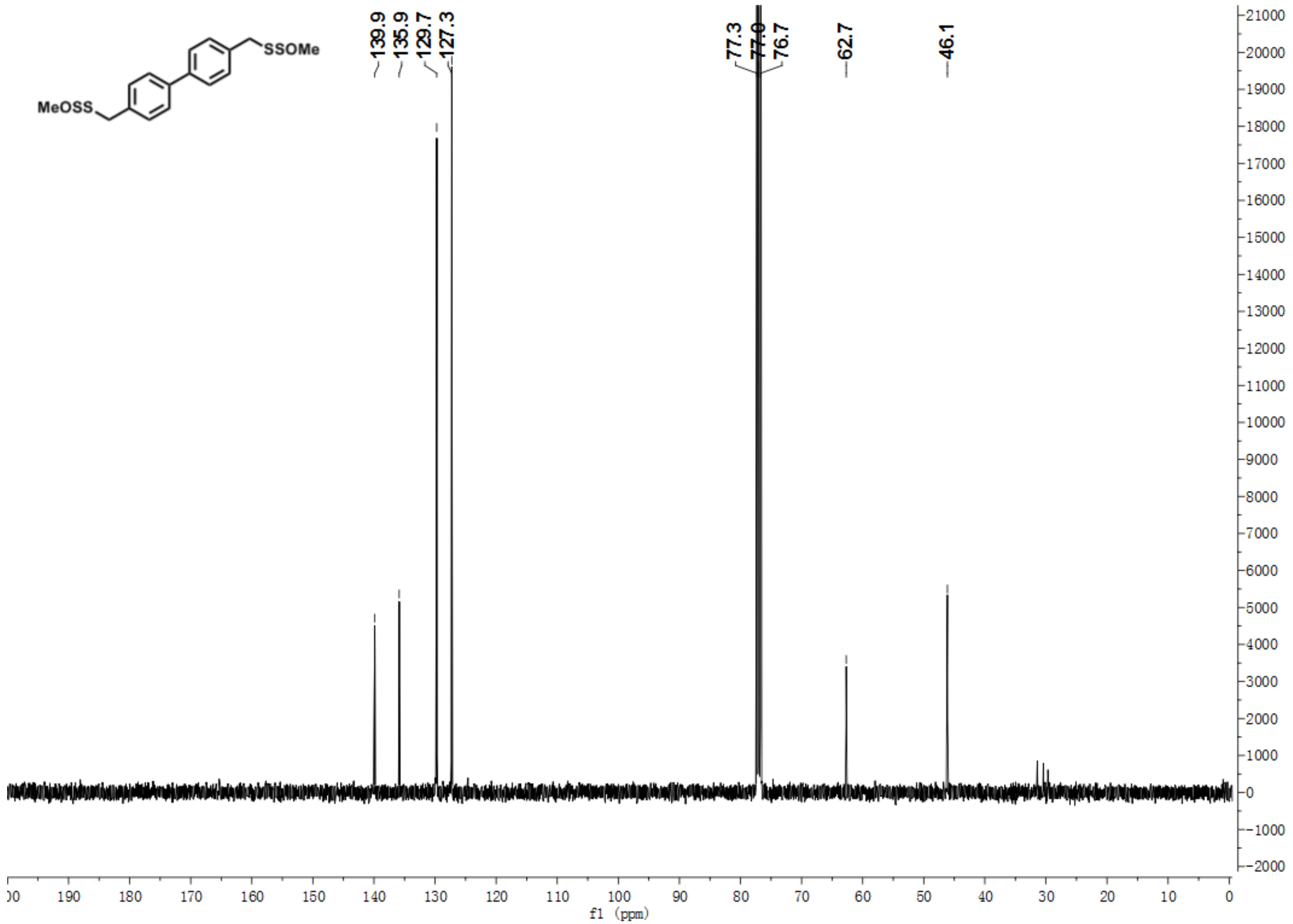

Supplementary Figure 34. ^{13}C NMR spectra for Compound **2o**.

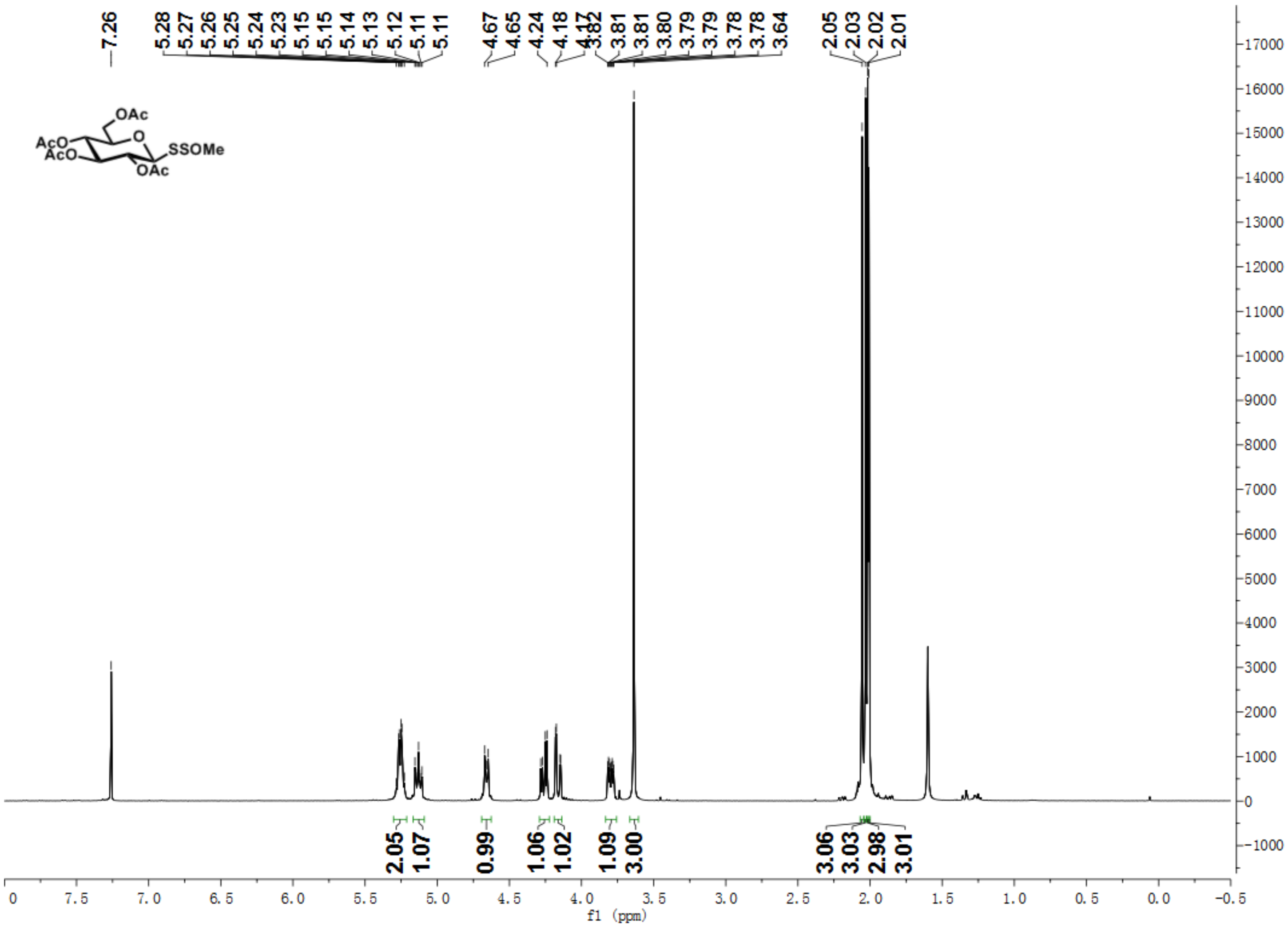

Supplementary Figure 35. ^{1}H NMR spectra for Compound **2p**.

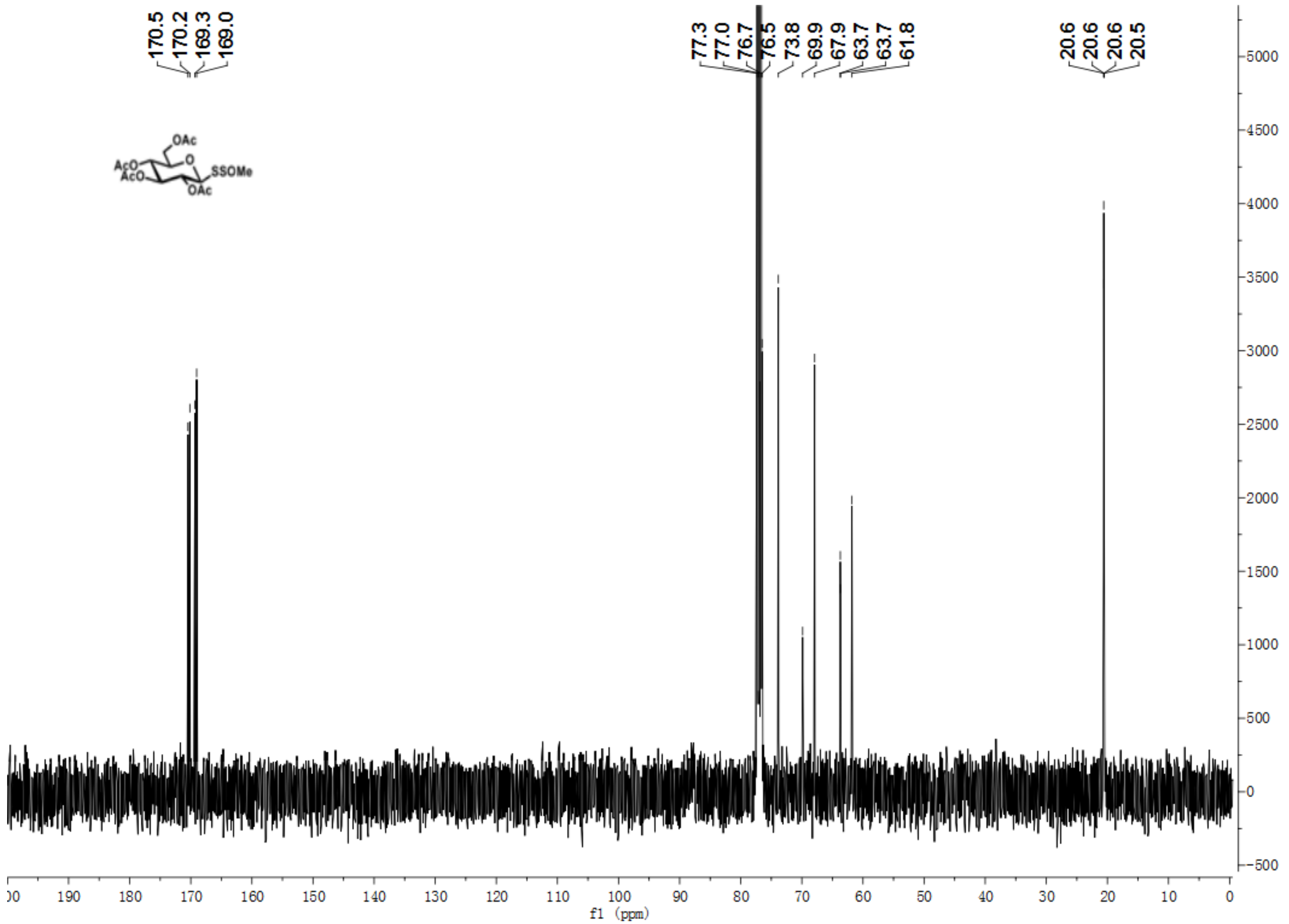

Supplementary Figure 36. ^{13}C NMR spectra for Compound **2p**.

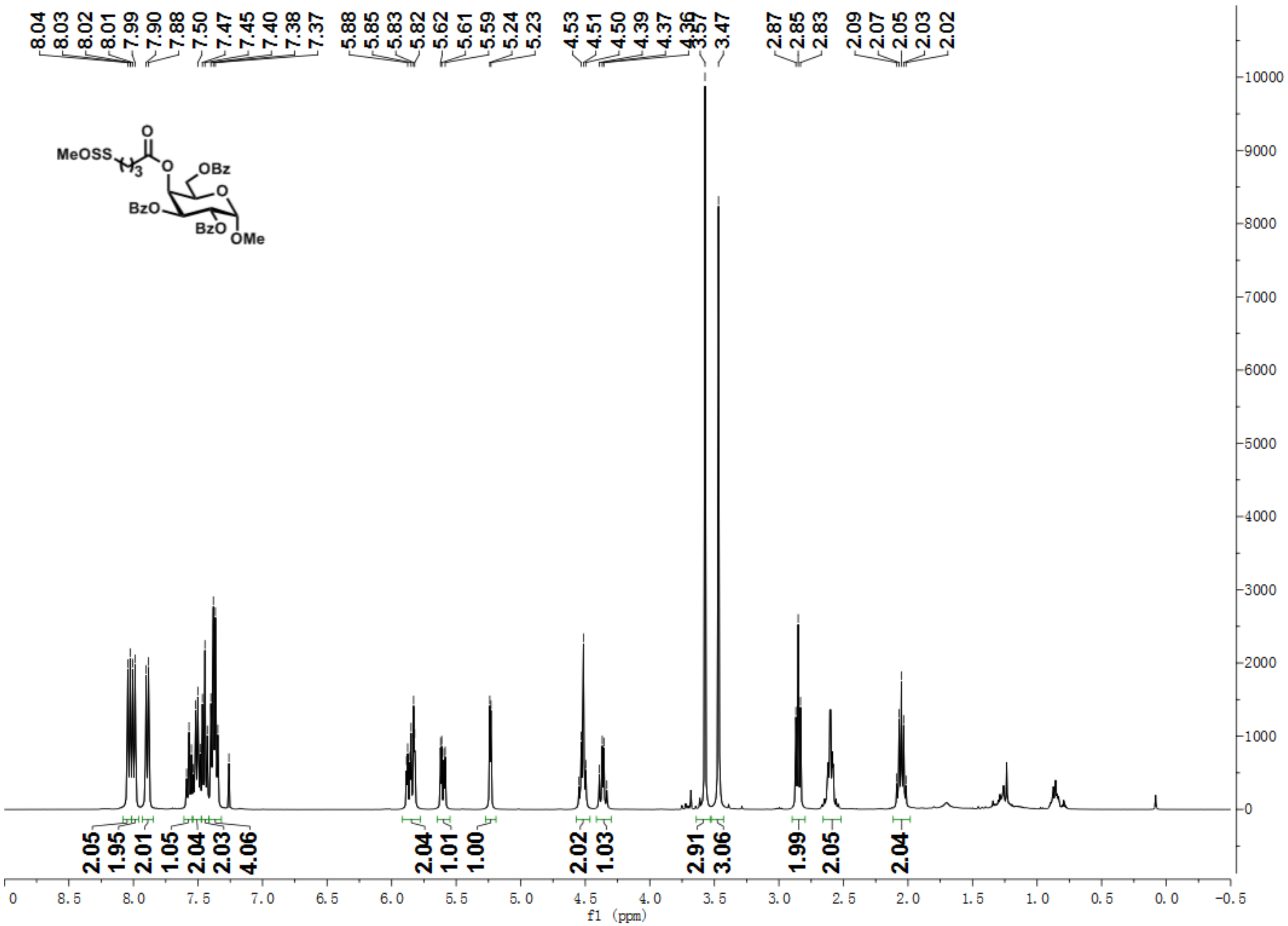

Supplementary Figure 37. ^{1}H NMR spectra for Compound **2q**.

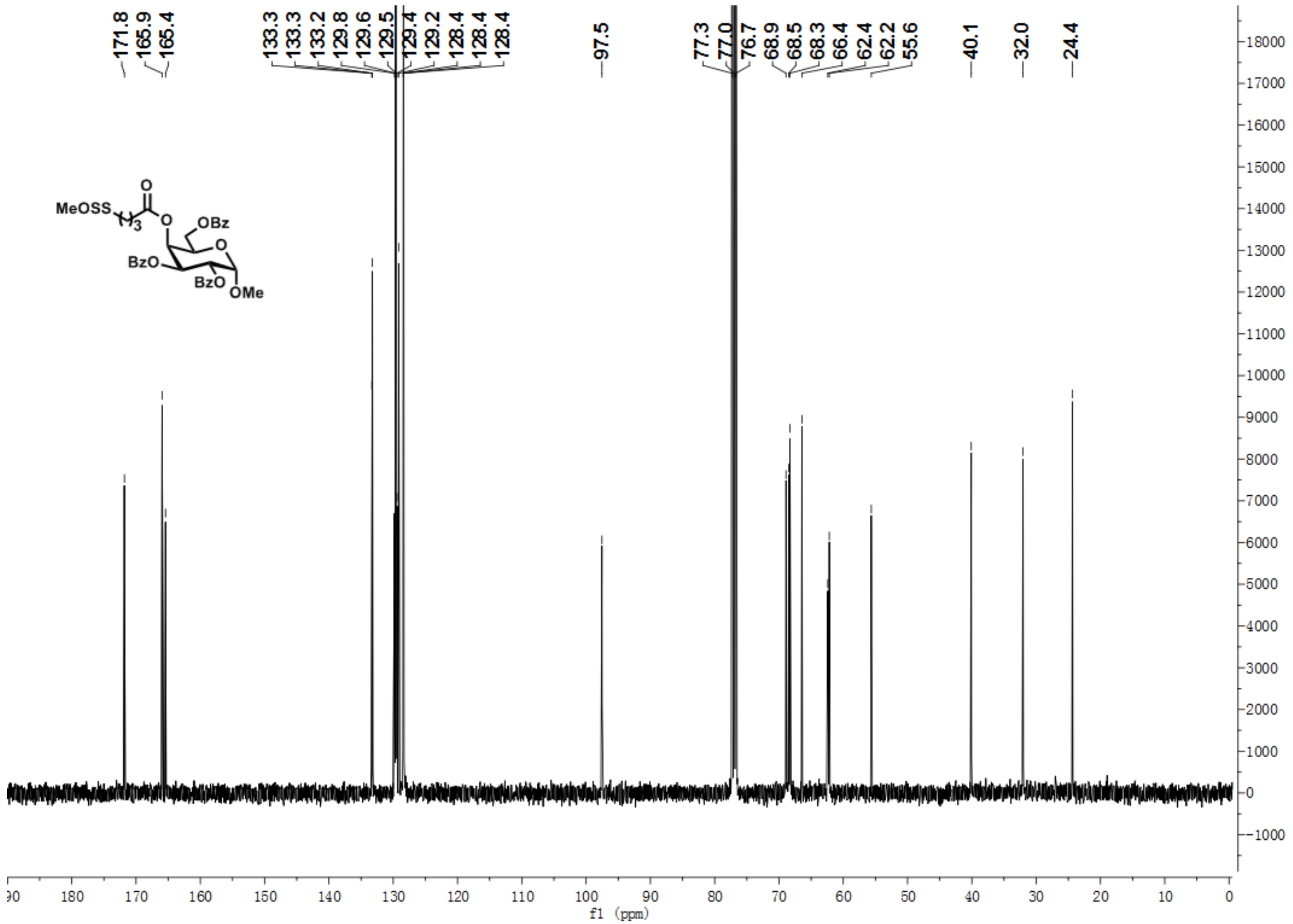

Supplementary Figure 38. ^{13}C NMR spectra for Compound **2q**.

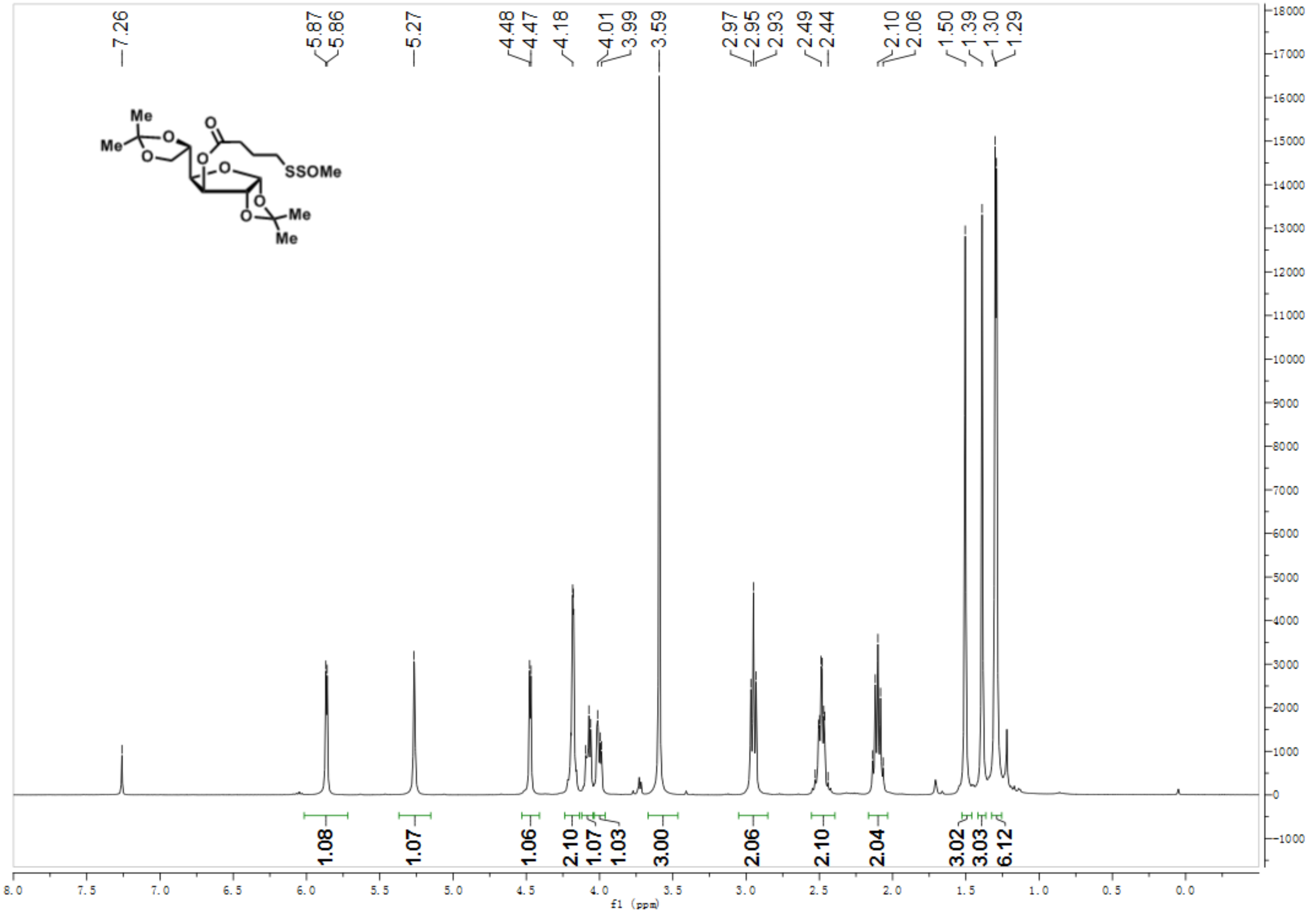

Supplementary Figure 39. ^{1}H NMR spectra for Compound **2r**.

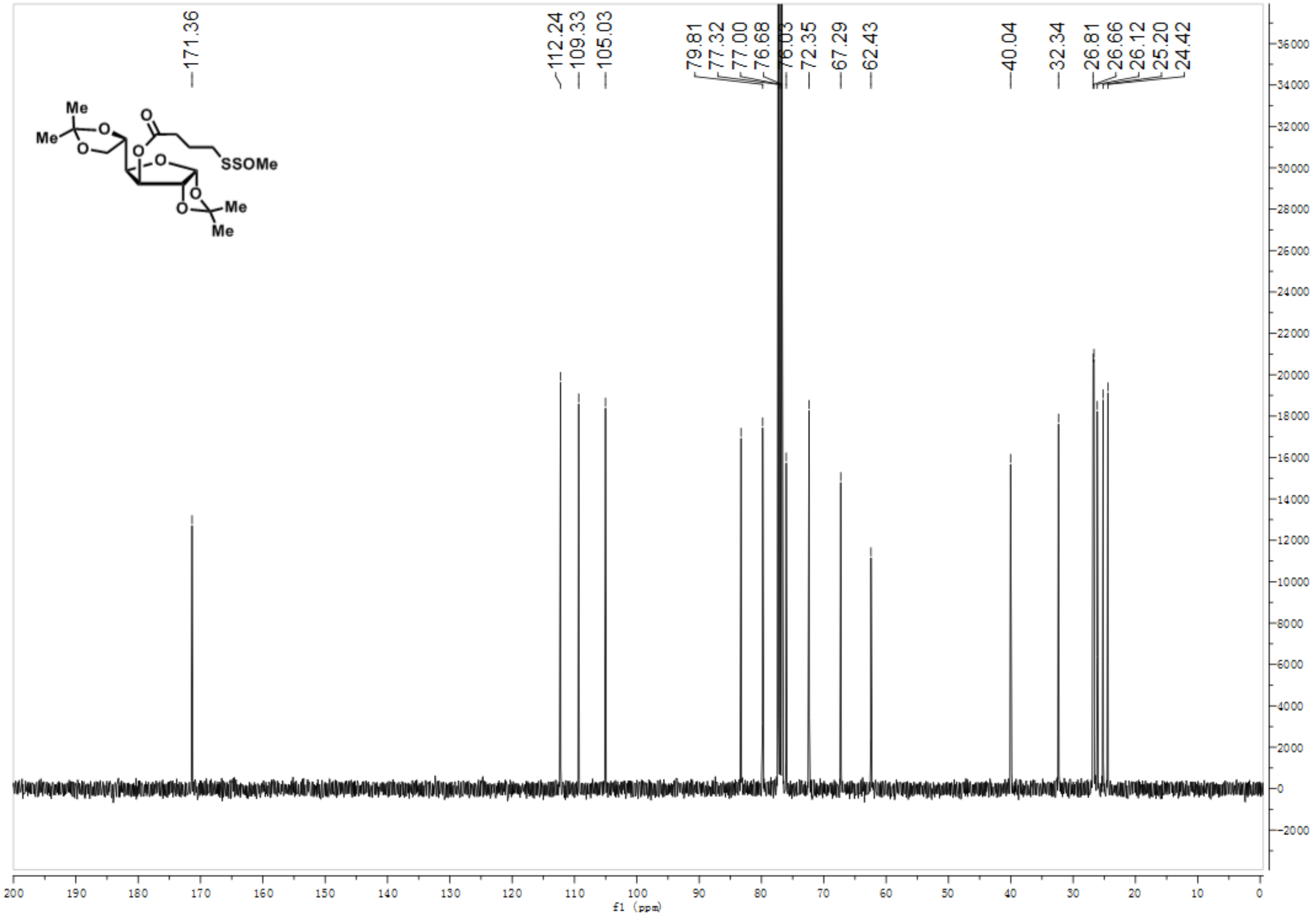

Supplementary Figure 40. ^{13}C NMR spectra for Compound **2r**.

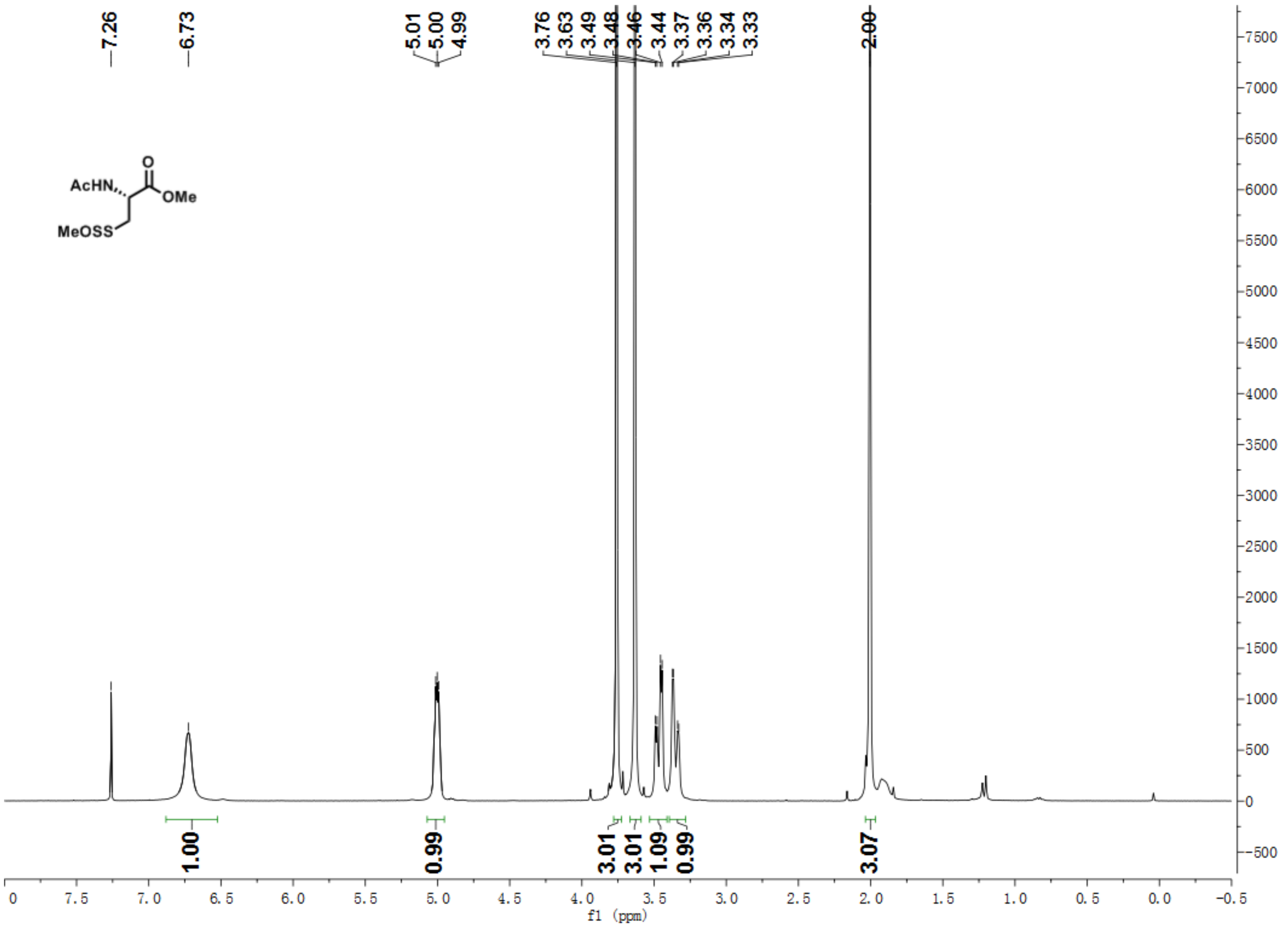

Supplementary Figure 41. ^{1}H NMR spectra for Compound **2s**.

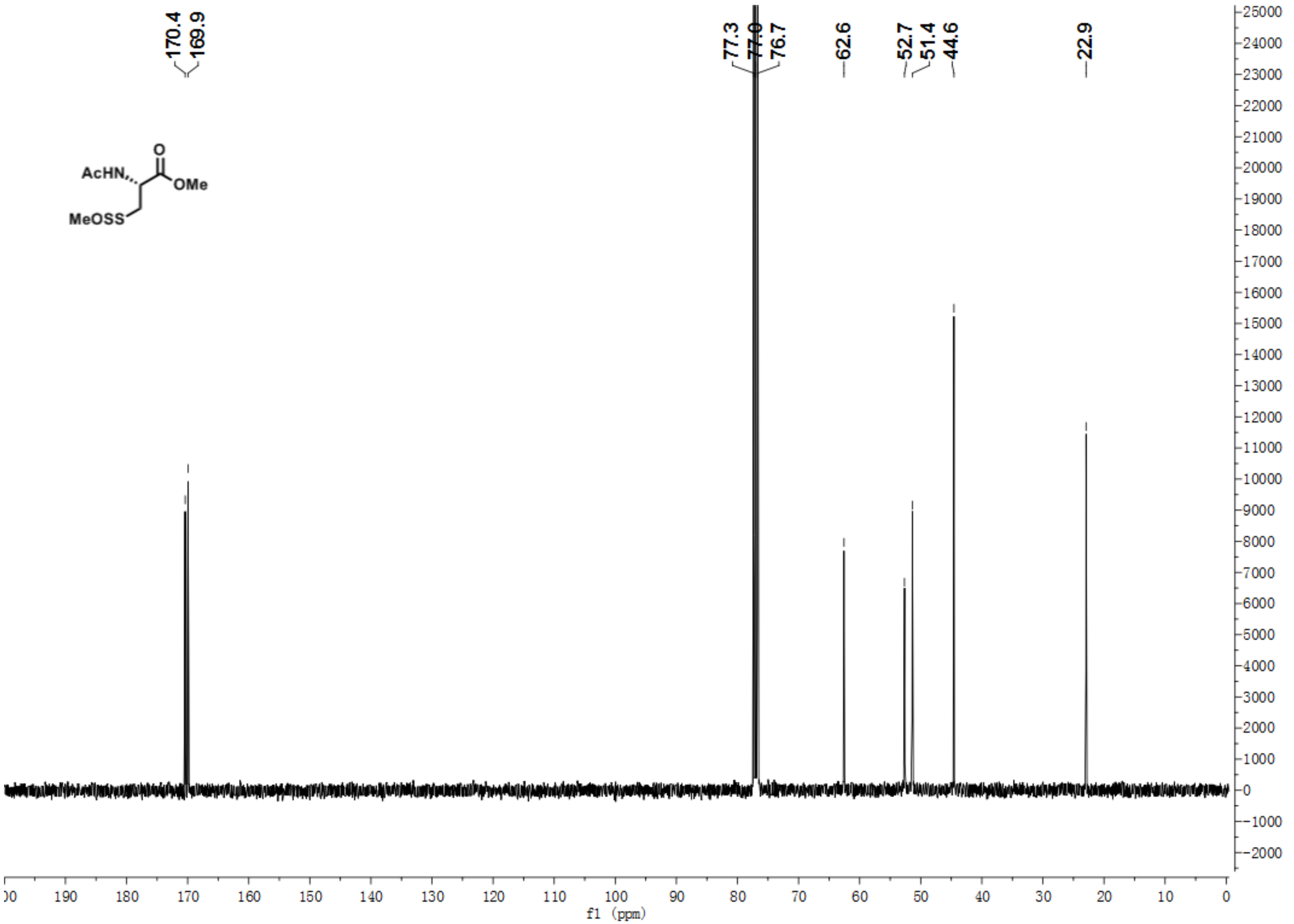

Supplementary Figure 42. ^{13}C NMR spectra for Compound **2s**.

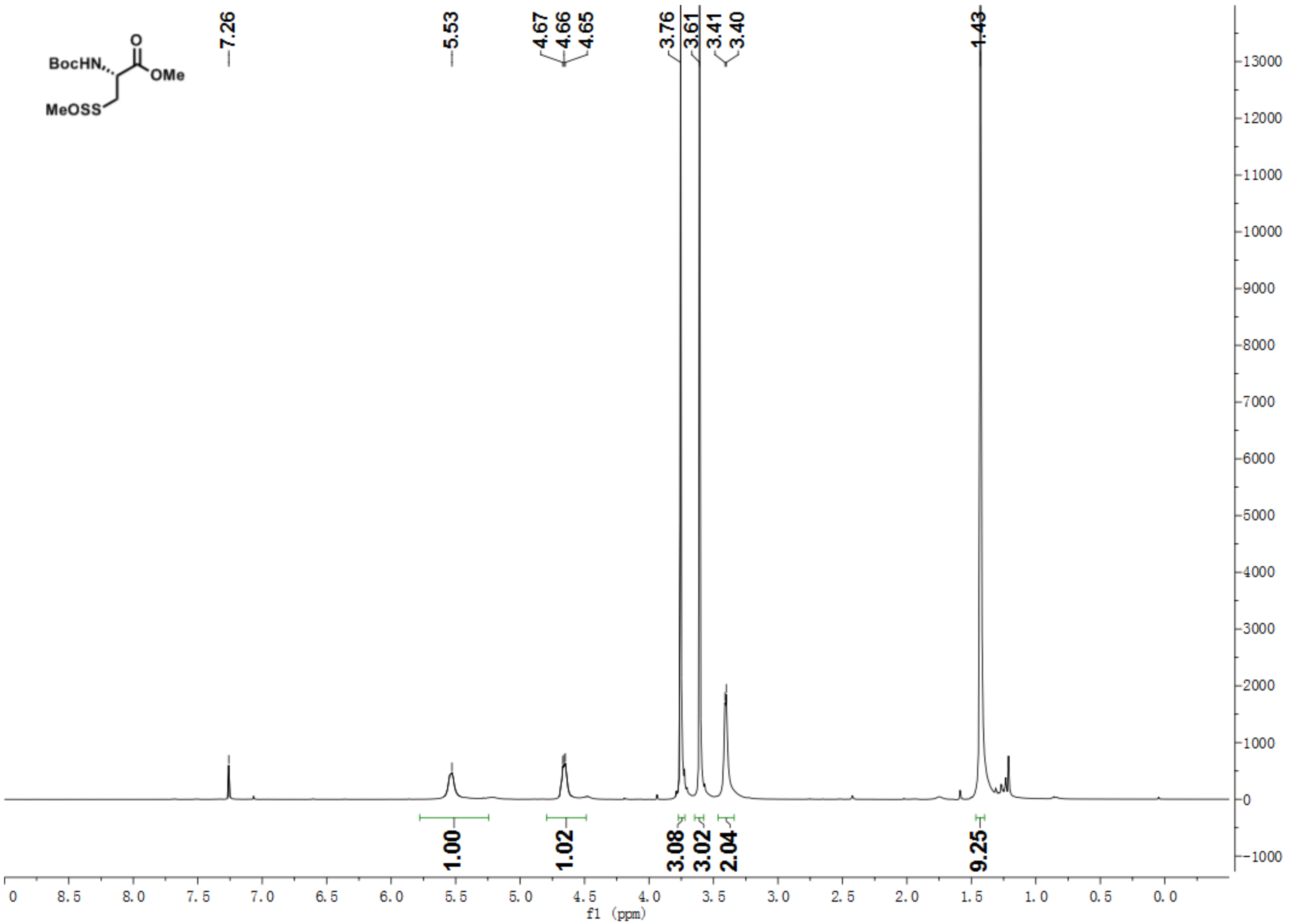

Supplementary Figure 43. ^{1}H NMR spectra for Compound **2t**.

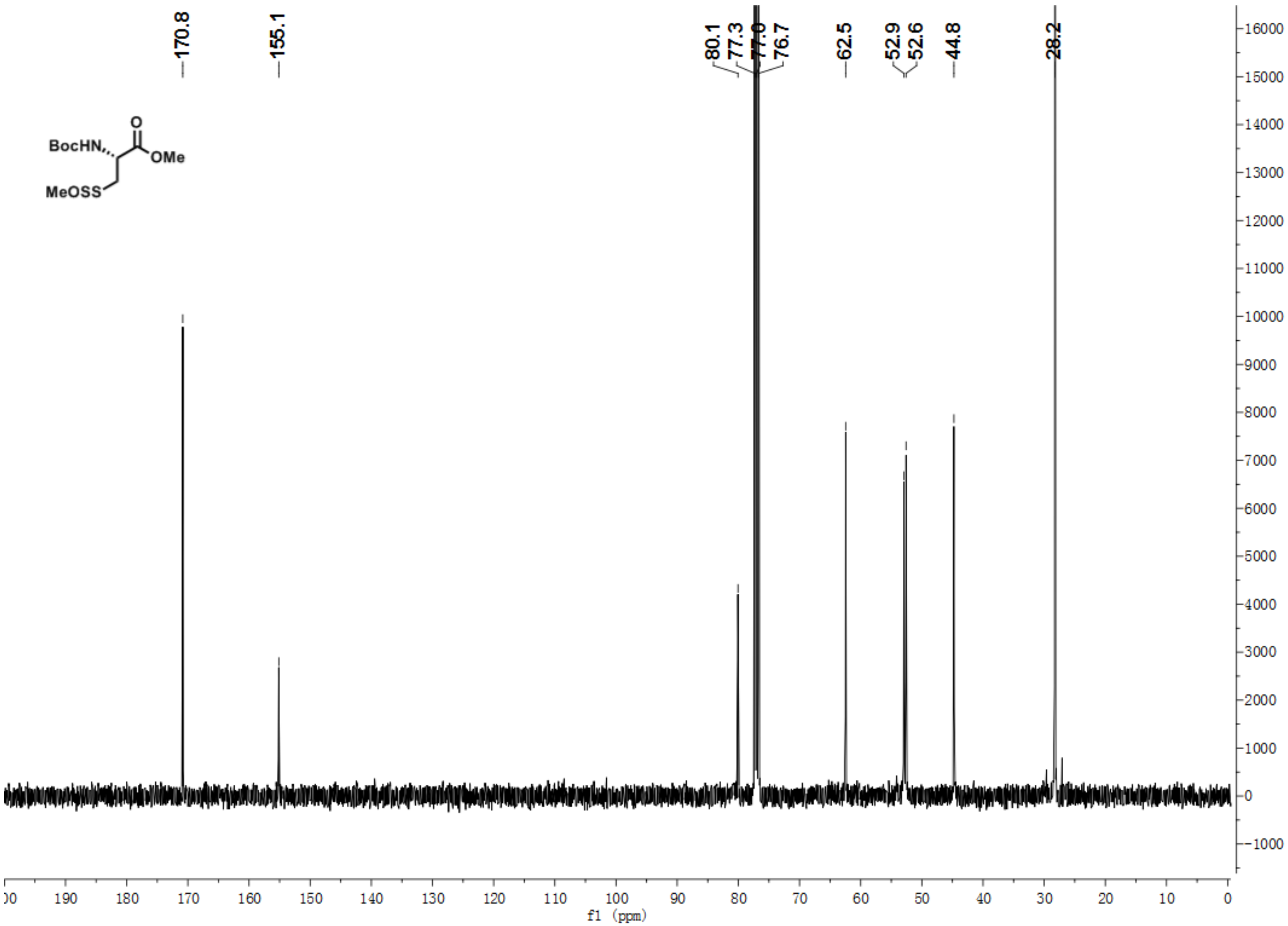

Supplementary Figure 44. ^{13}C NMR spectra for Compound **2t**.

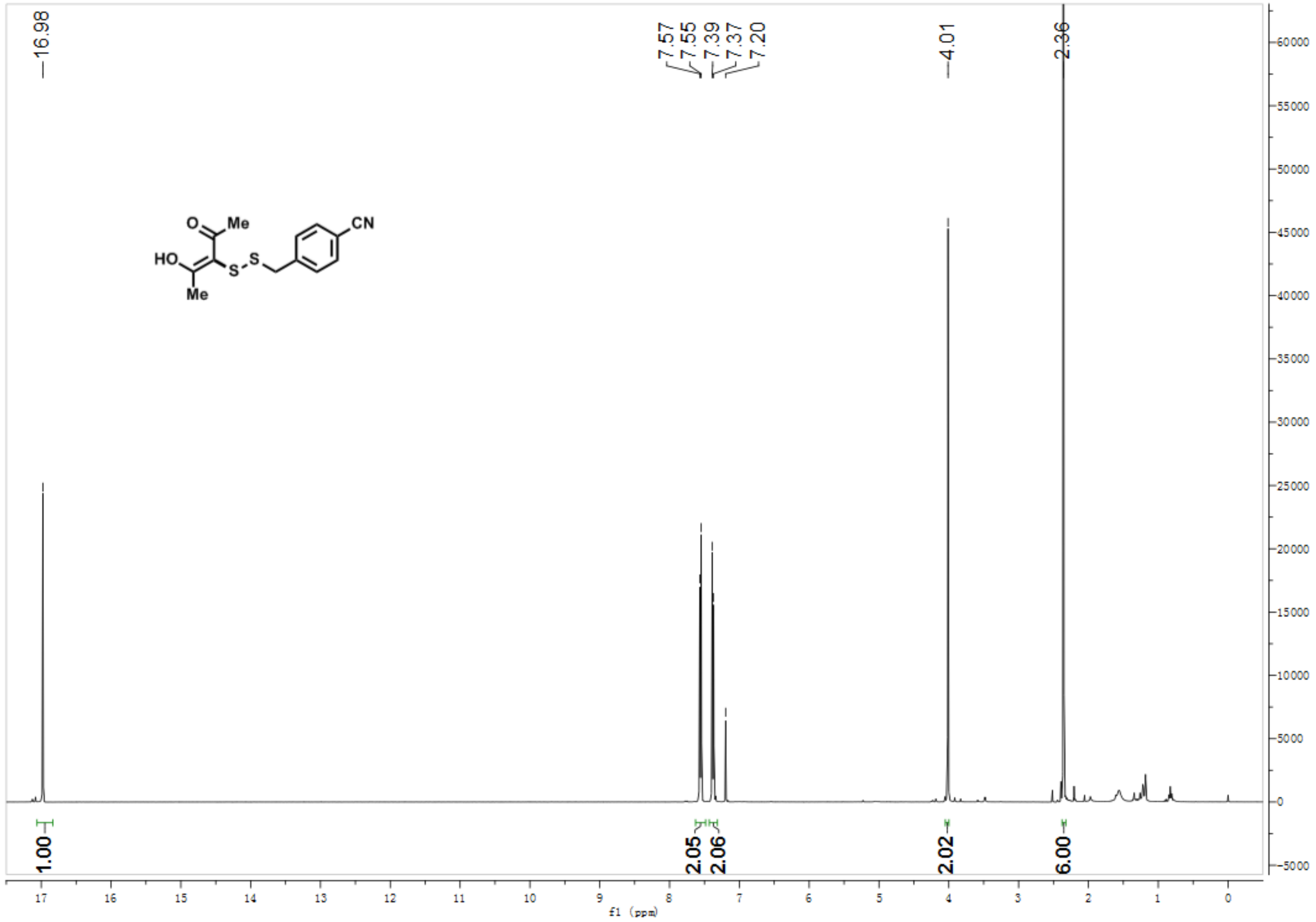

Supplementary Figure 45. ^{1}H NMR spectra for Compound **3a**.

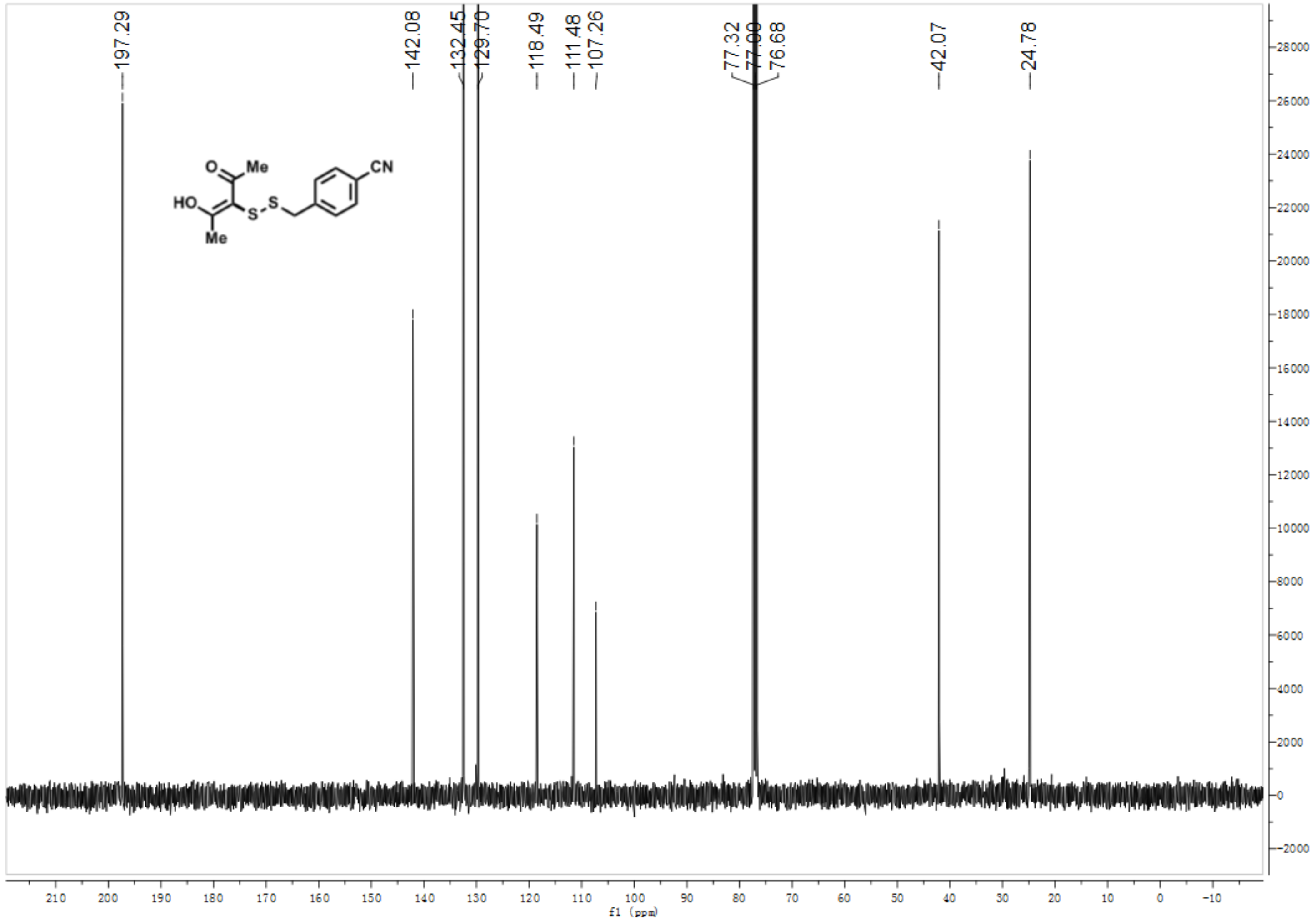

Supplementary Figure 46. ^{13}C NMR spectra for Compound **3a**.

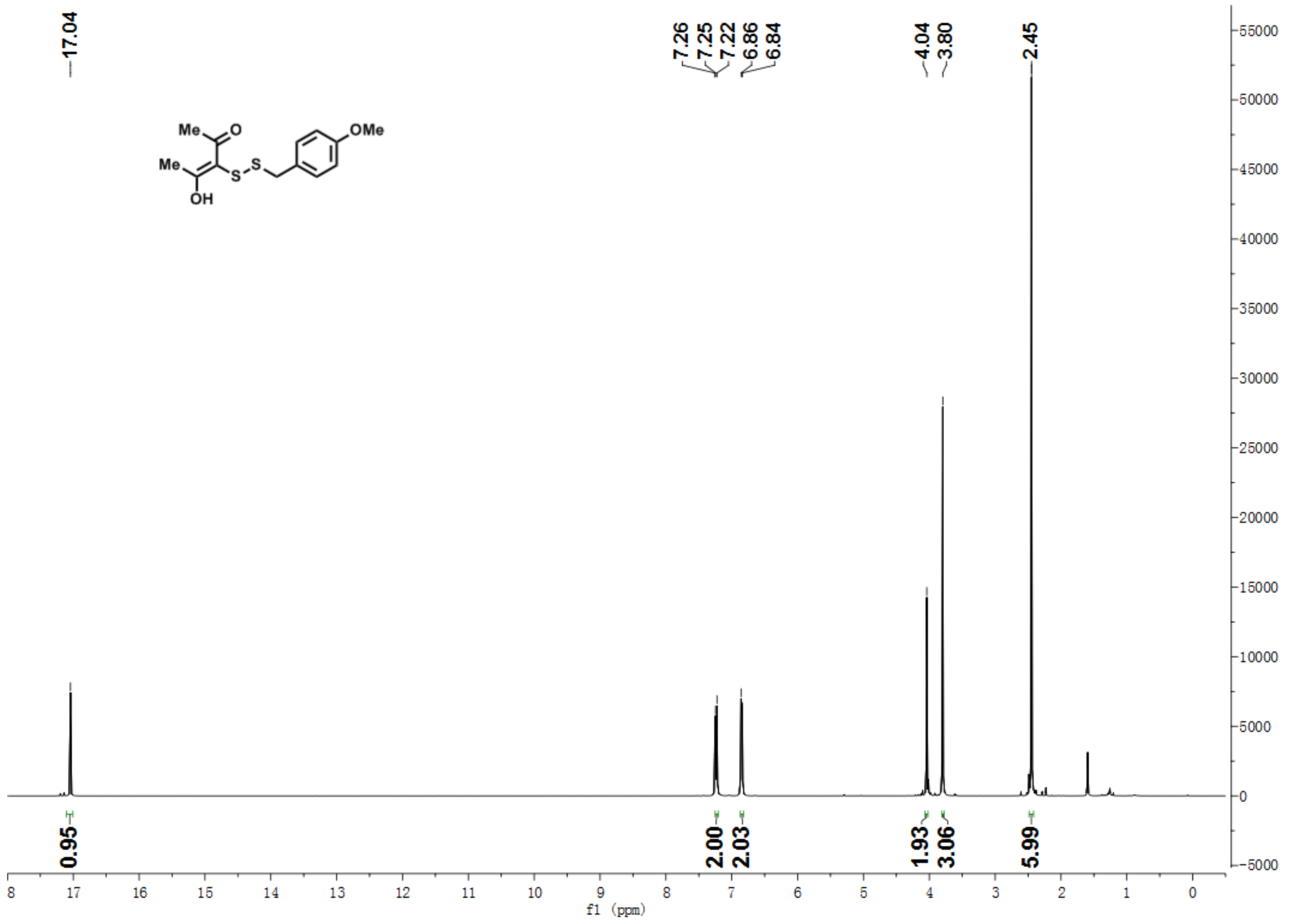

Supplementary Figure 47. ^{1}H NMR spectra for Compound **3b**.

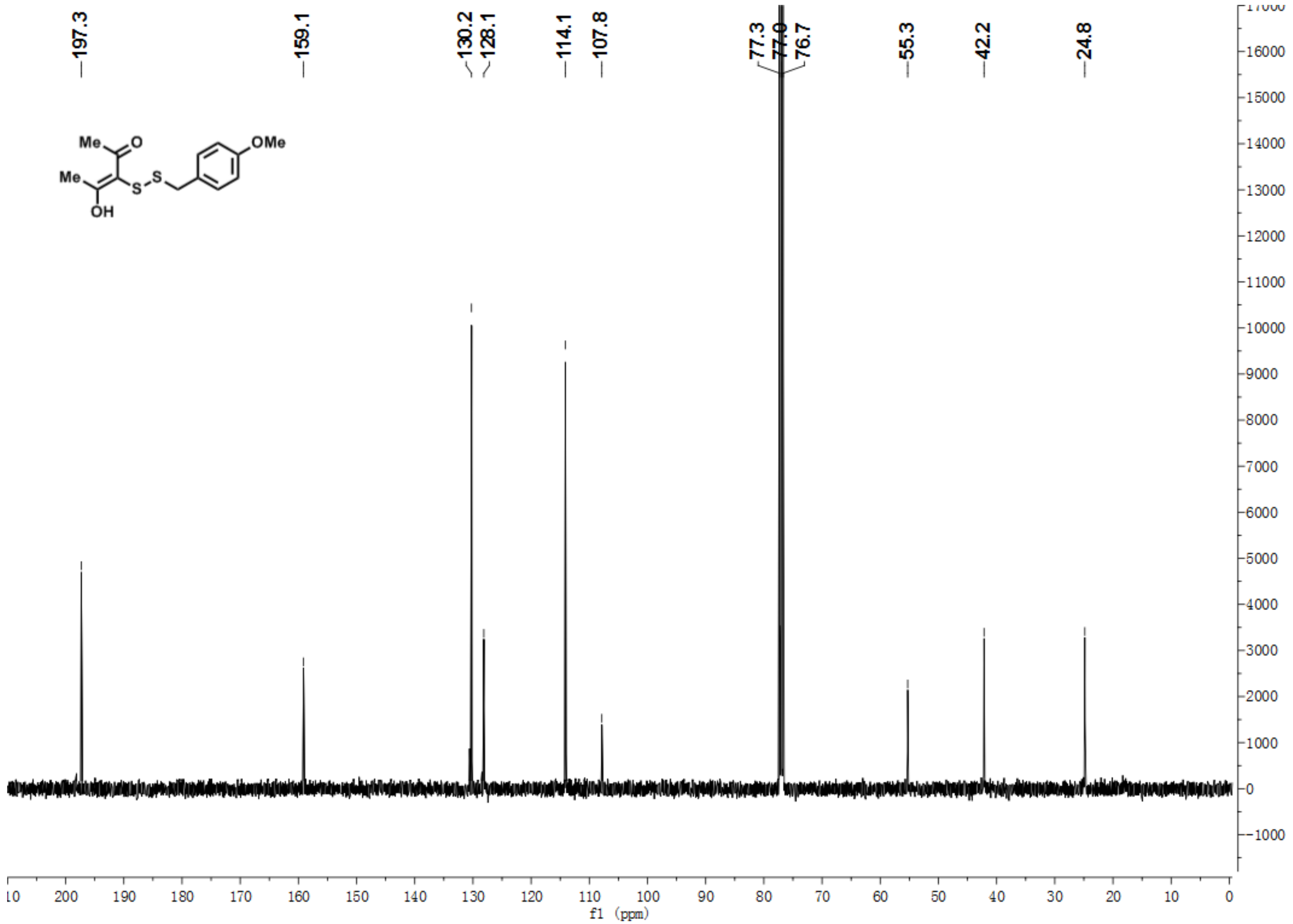

Supplementary Figure 48. ^{13}C NMR spectra for Compound **3b**.

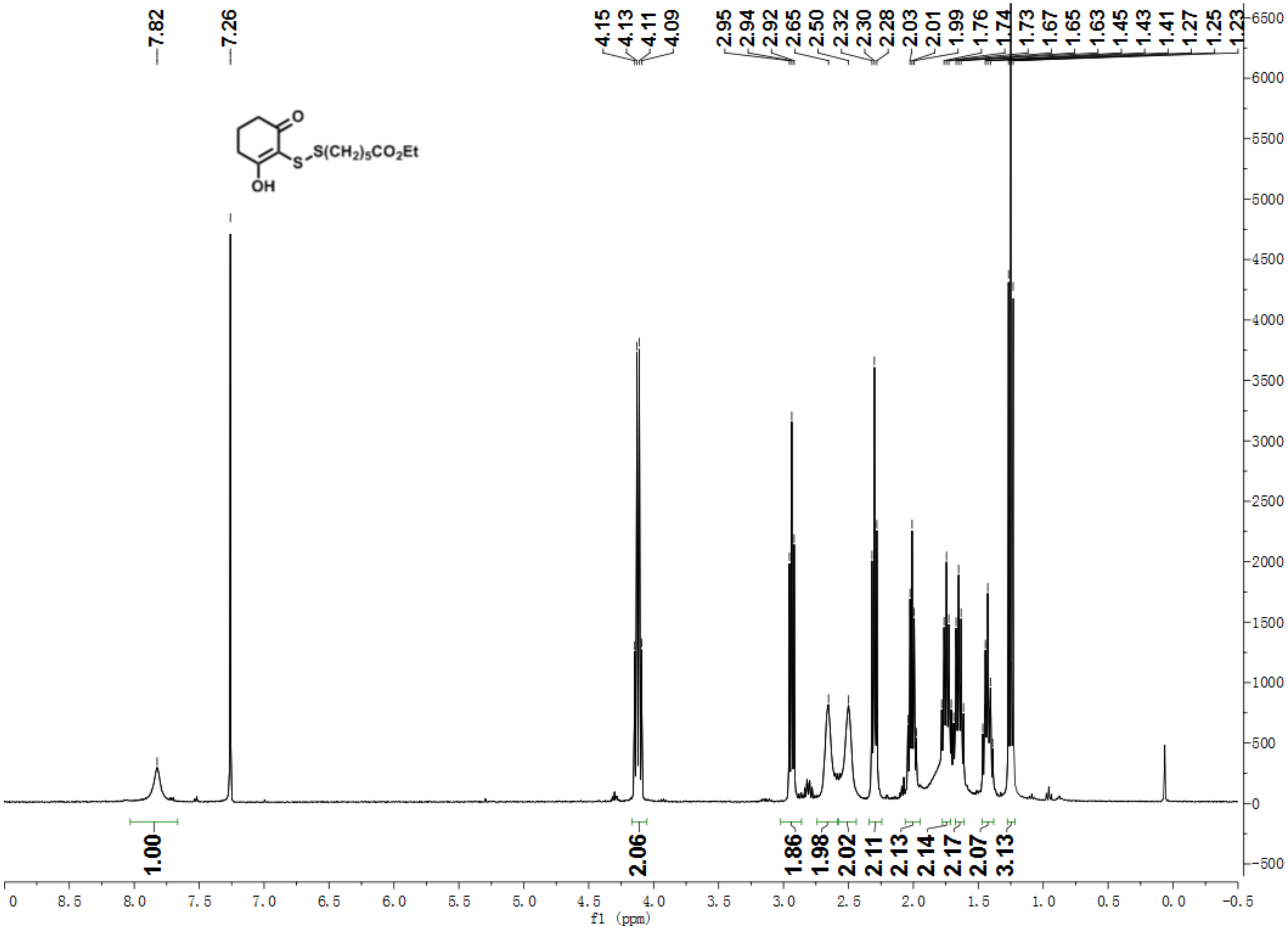

Supplementary Figure 49. ^{1}H NMR spectra for Compound **3c**.

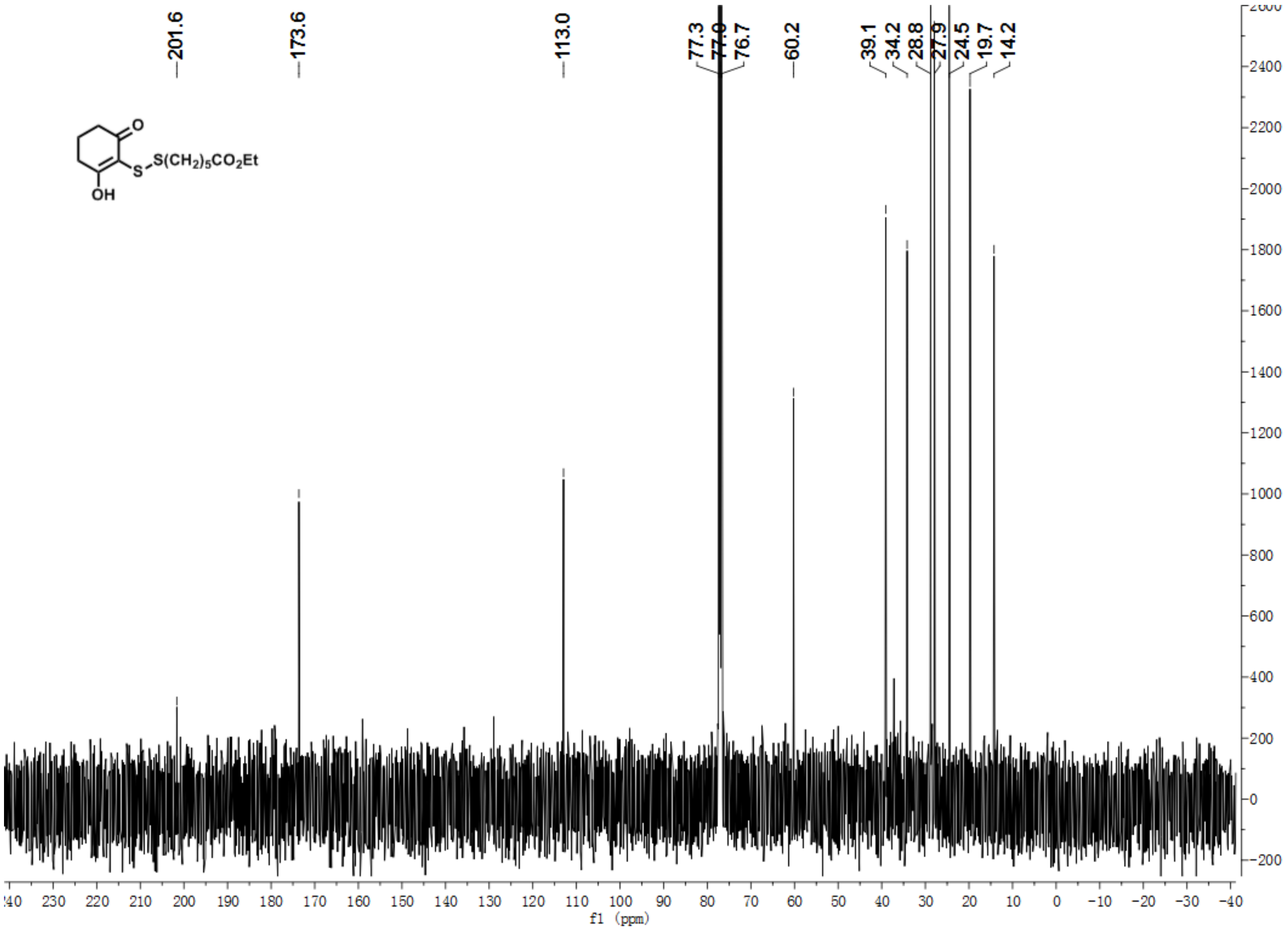

Supplementary Figure 50. ^{13}C NMR spectra for Compound **3c**.

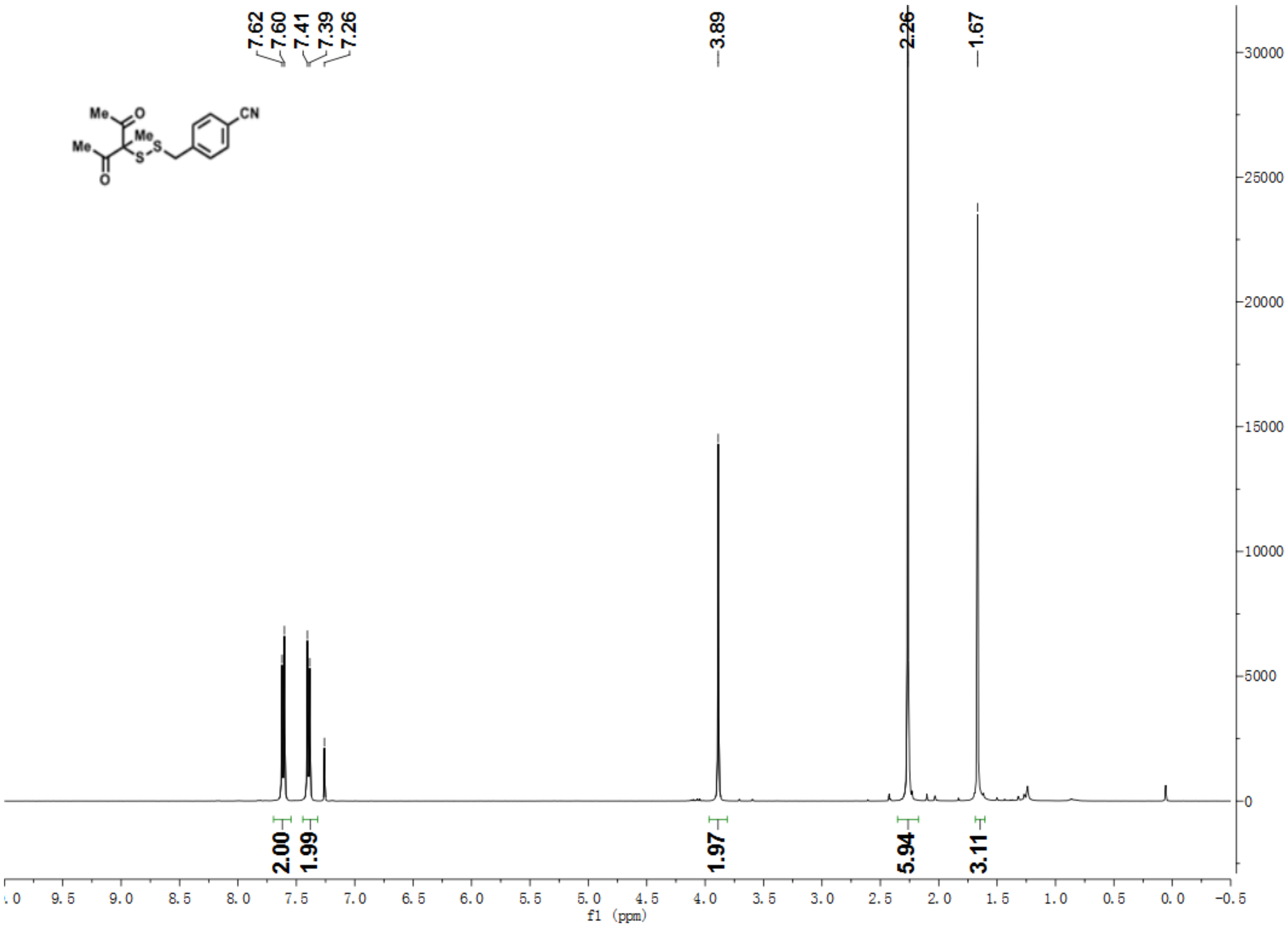

Supplementary Figure 51. ^{1}H NMR spectra for Compound **3d**.

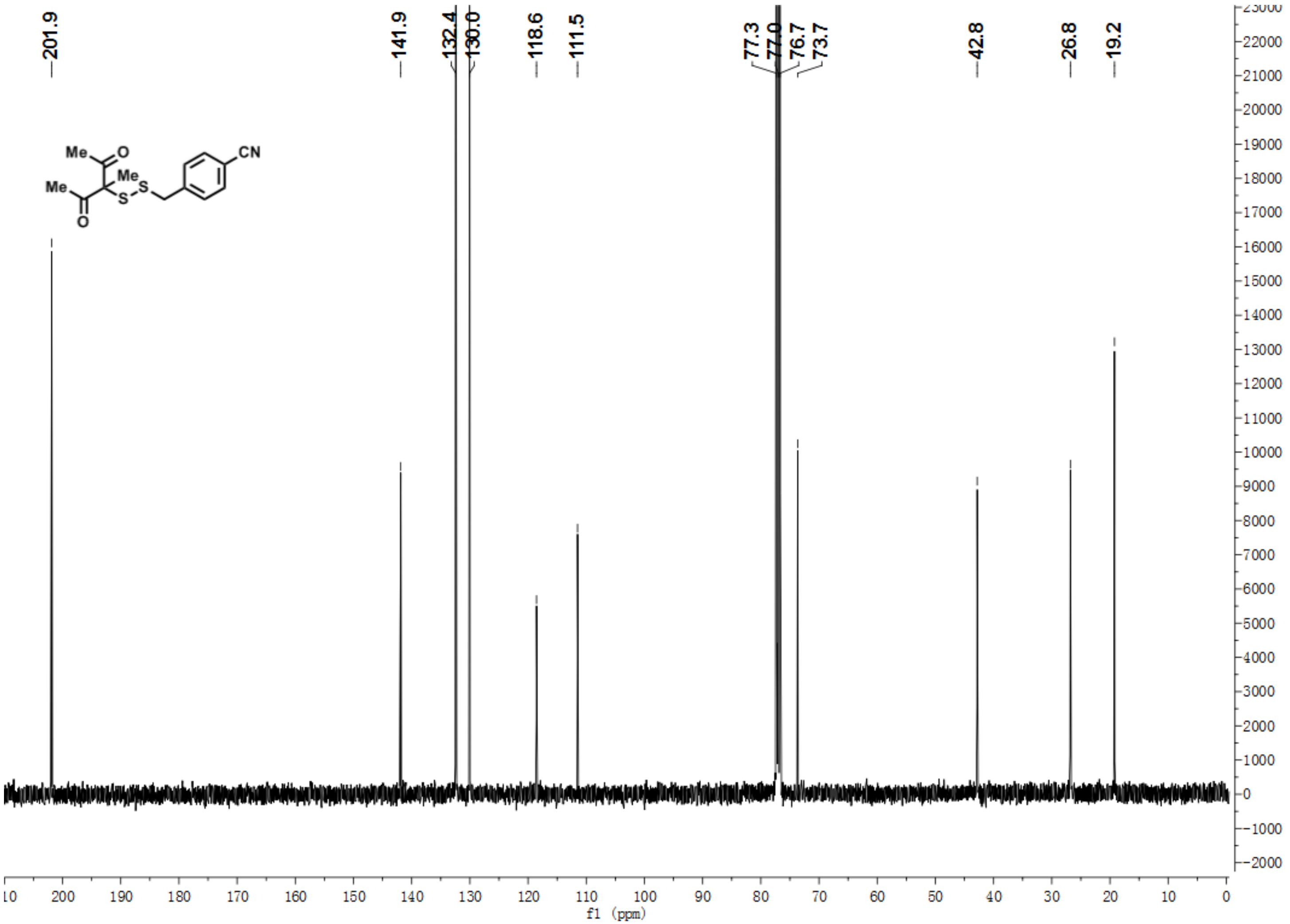

Supplementary Figure 52. ^{13}C NMR spectra for Compound **3d**.

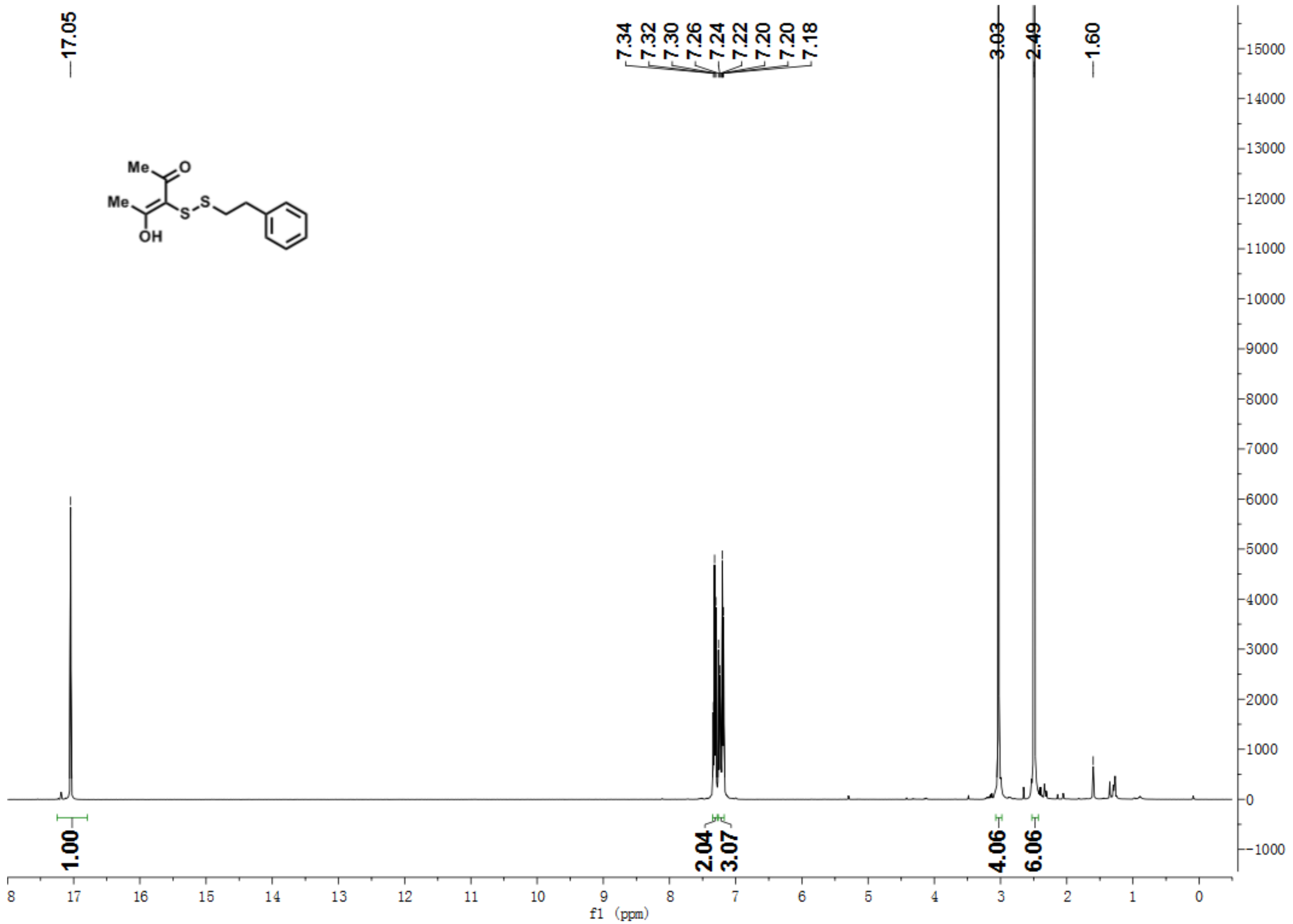

Supplementary Figure 53. ^{1}H NMR spectra for Compound **3e**.

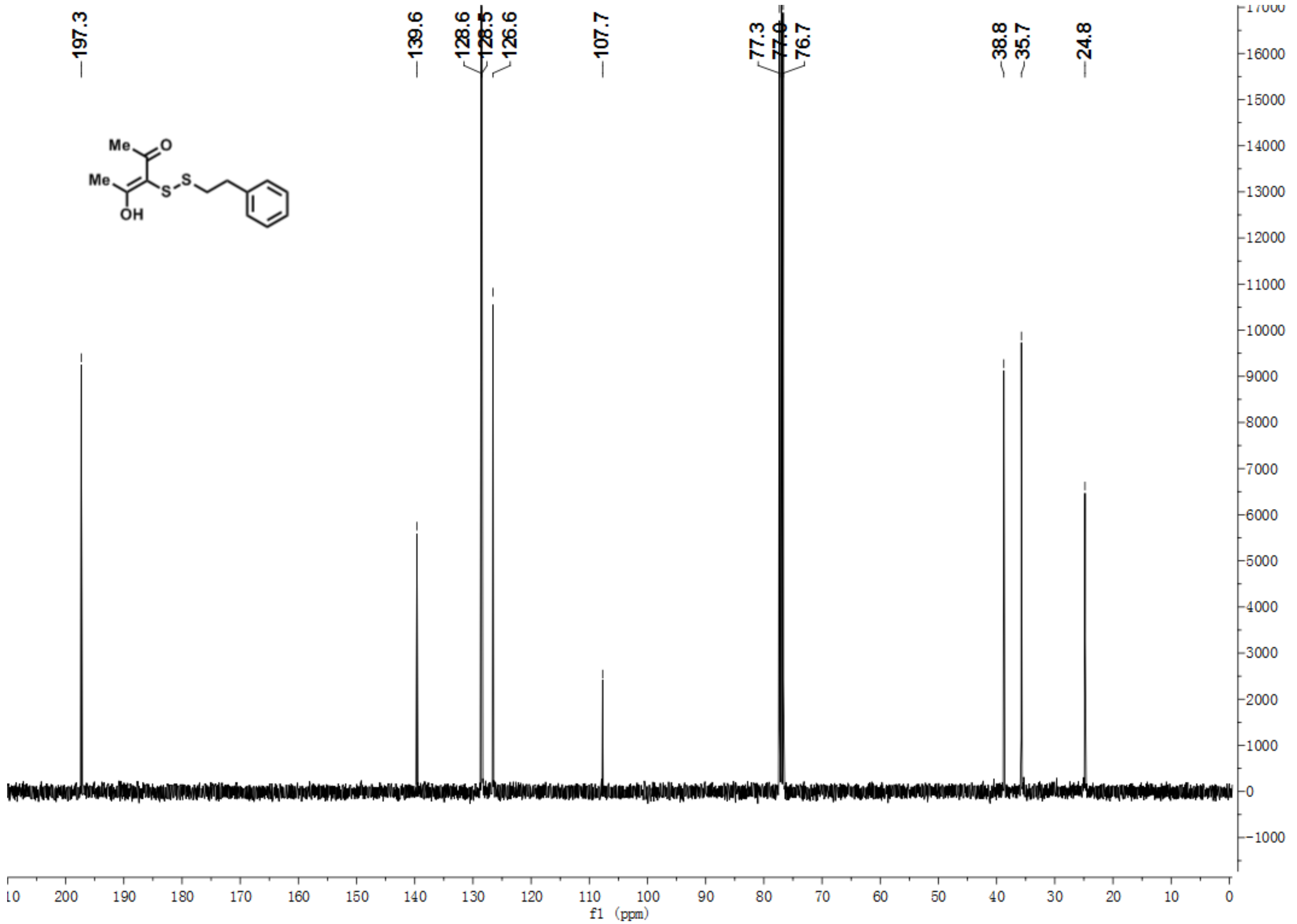

Supplementary Figure 54. ^{13}C NMR spectra for Compound **3e**.

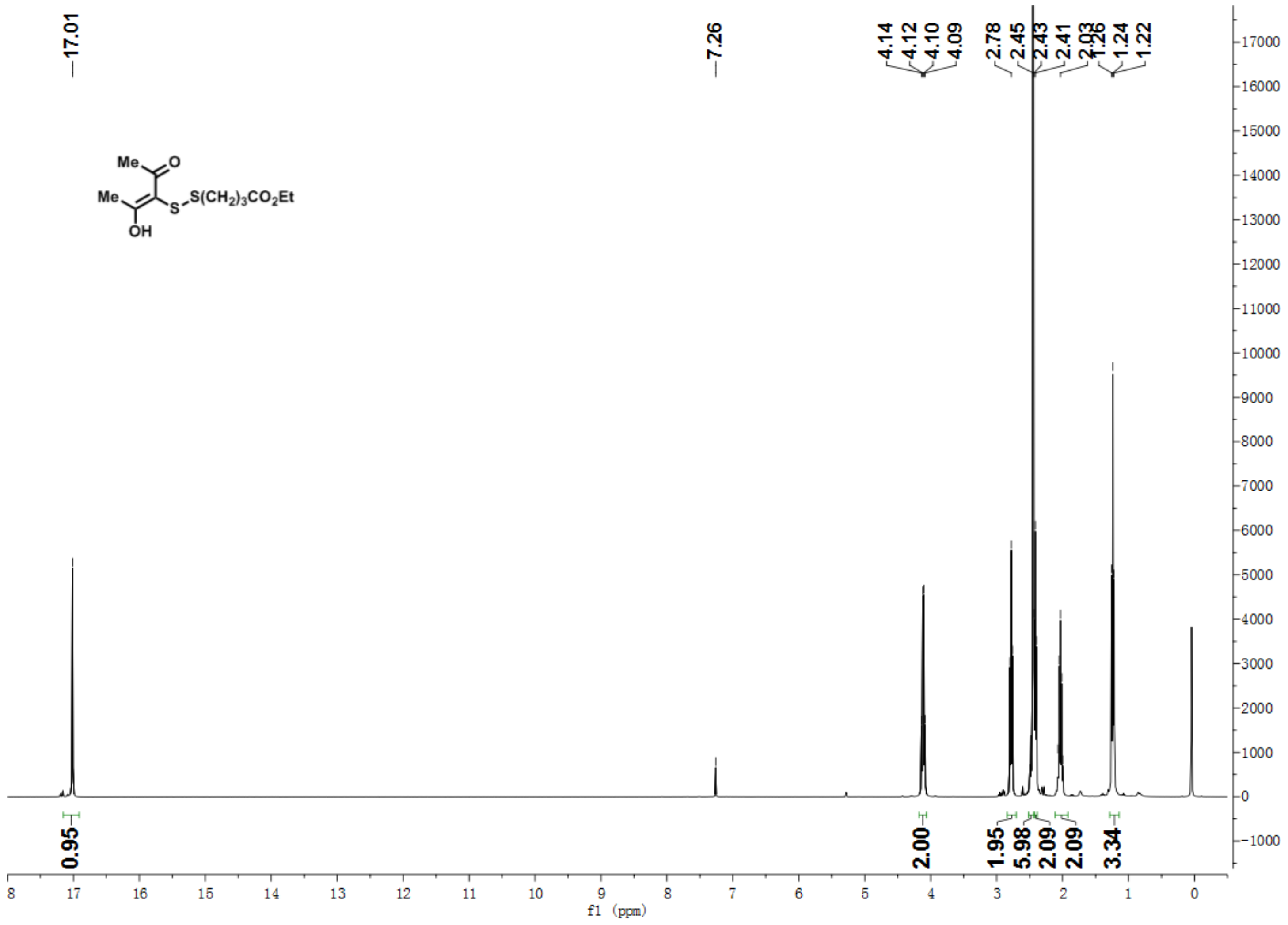

Supplementary Figure 55. ^{1}H NMR spectra for Compound **3f**.

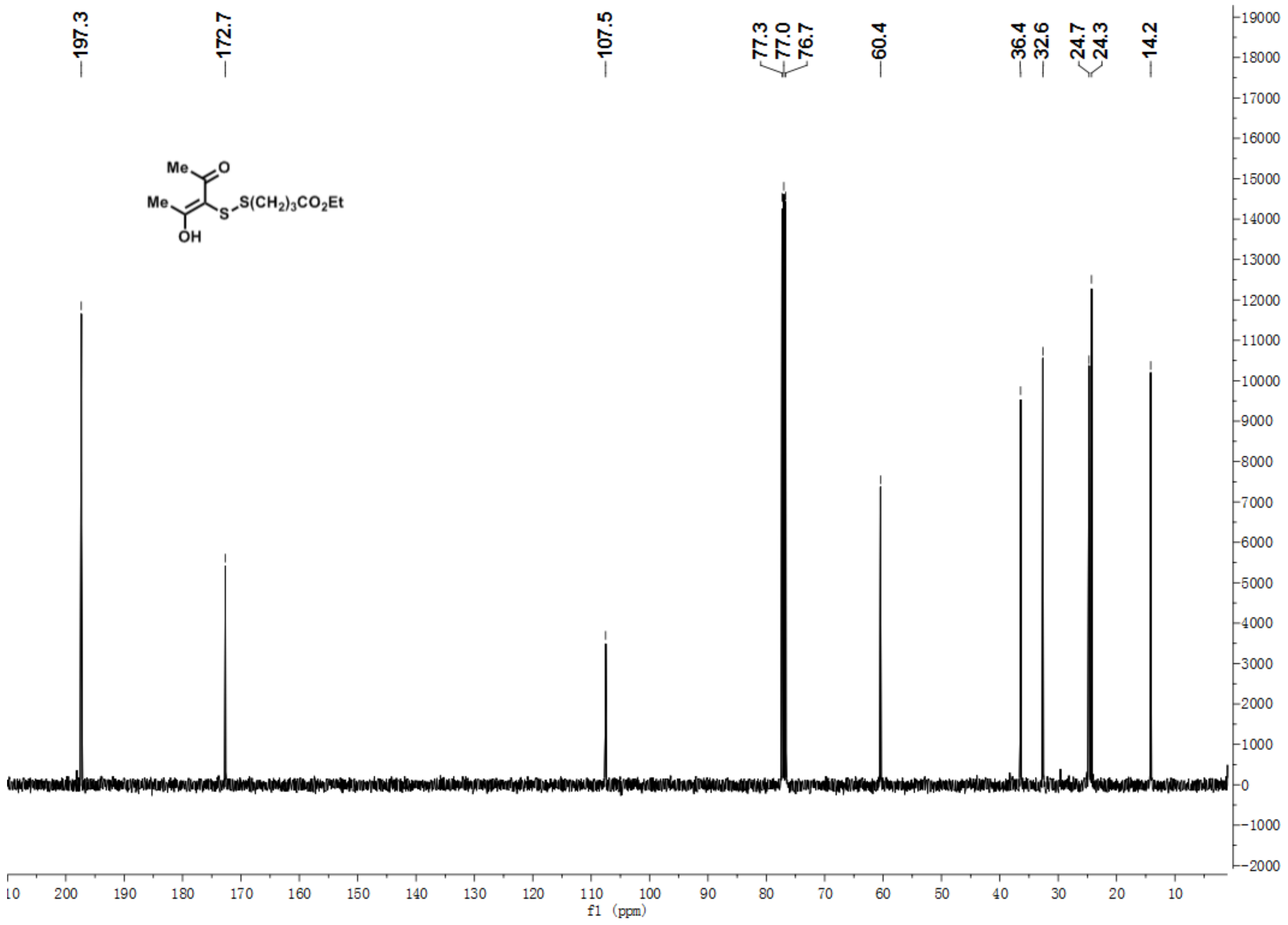

Supplementary Figure 56. ^{13}C NMR spectra for Compound **3f**.

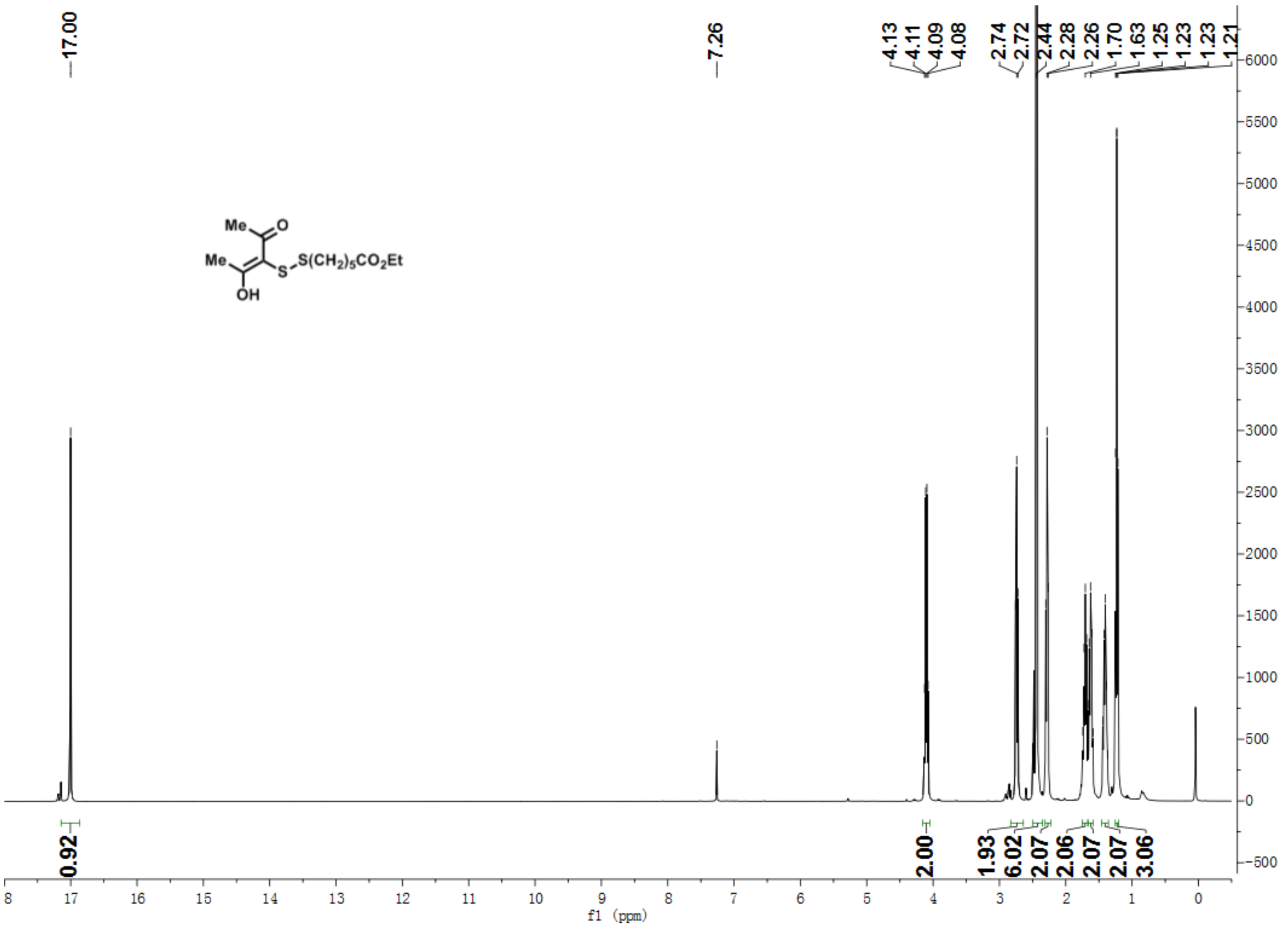

Supplementary Figure 57. ^{1}H NMR spectra for Compound **3g**.

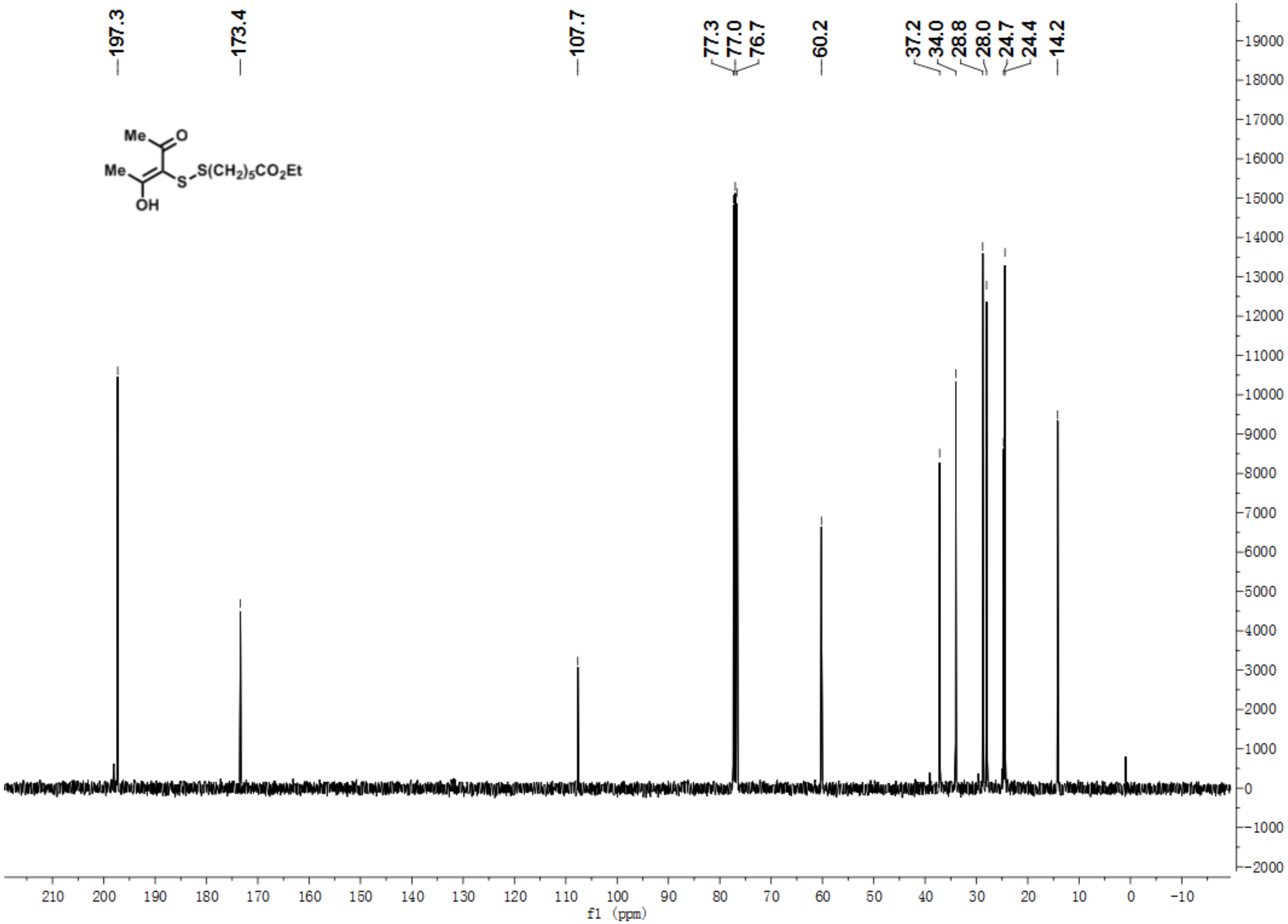

Supplementary Figure 58. ^{13}C NMR spectra for Compound **3g**.

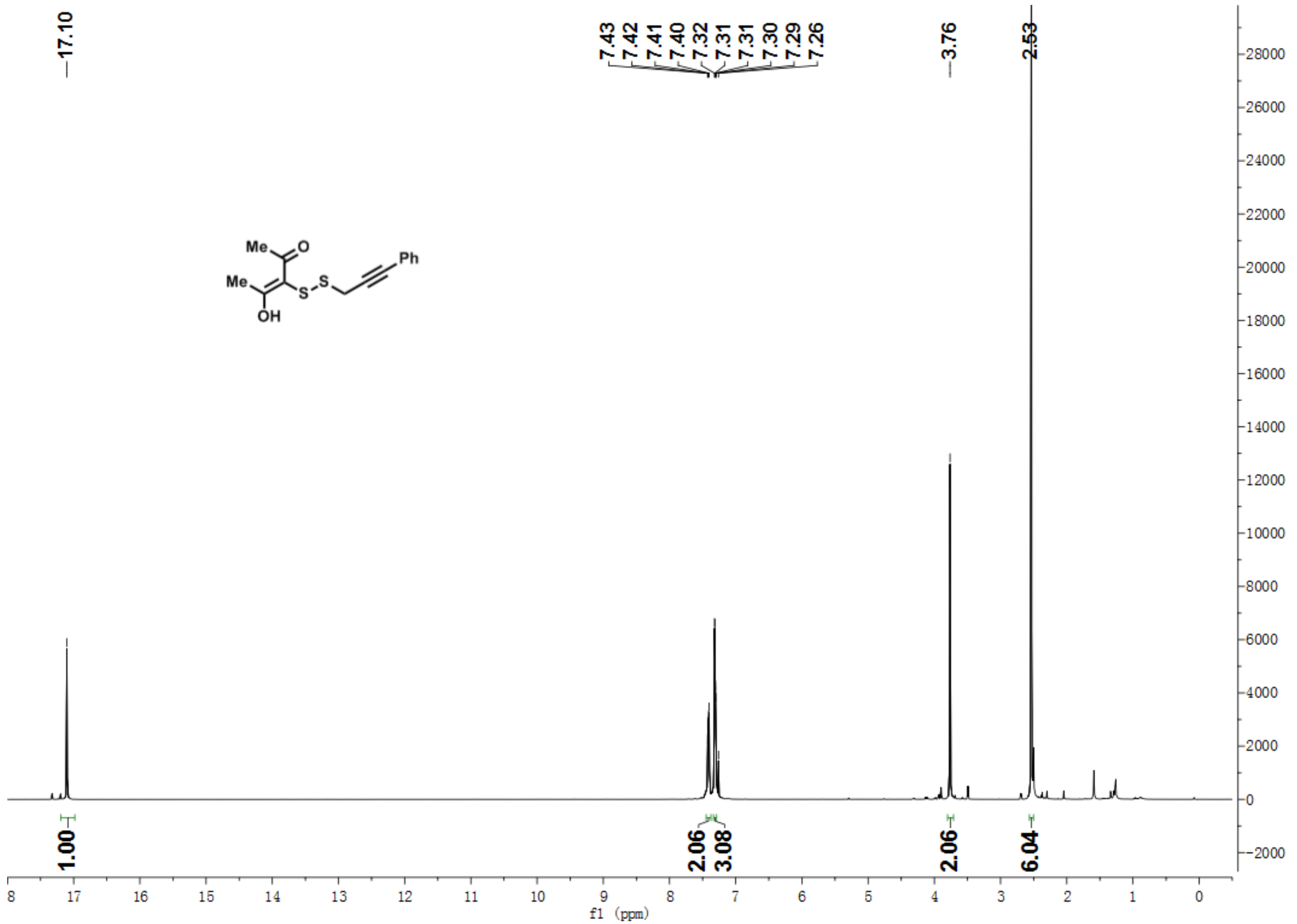

Supplementary Figure 59. ^{1}H NMR spectra for Compound **3h**.

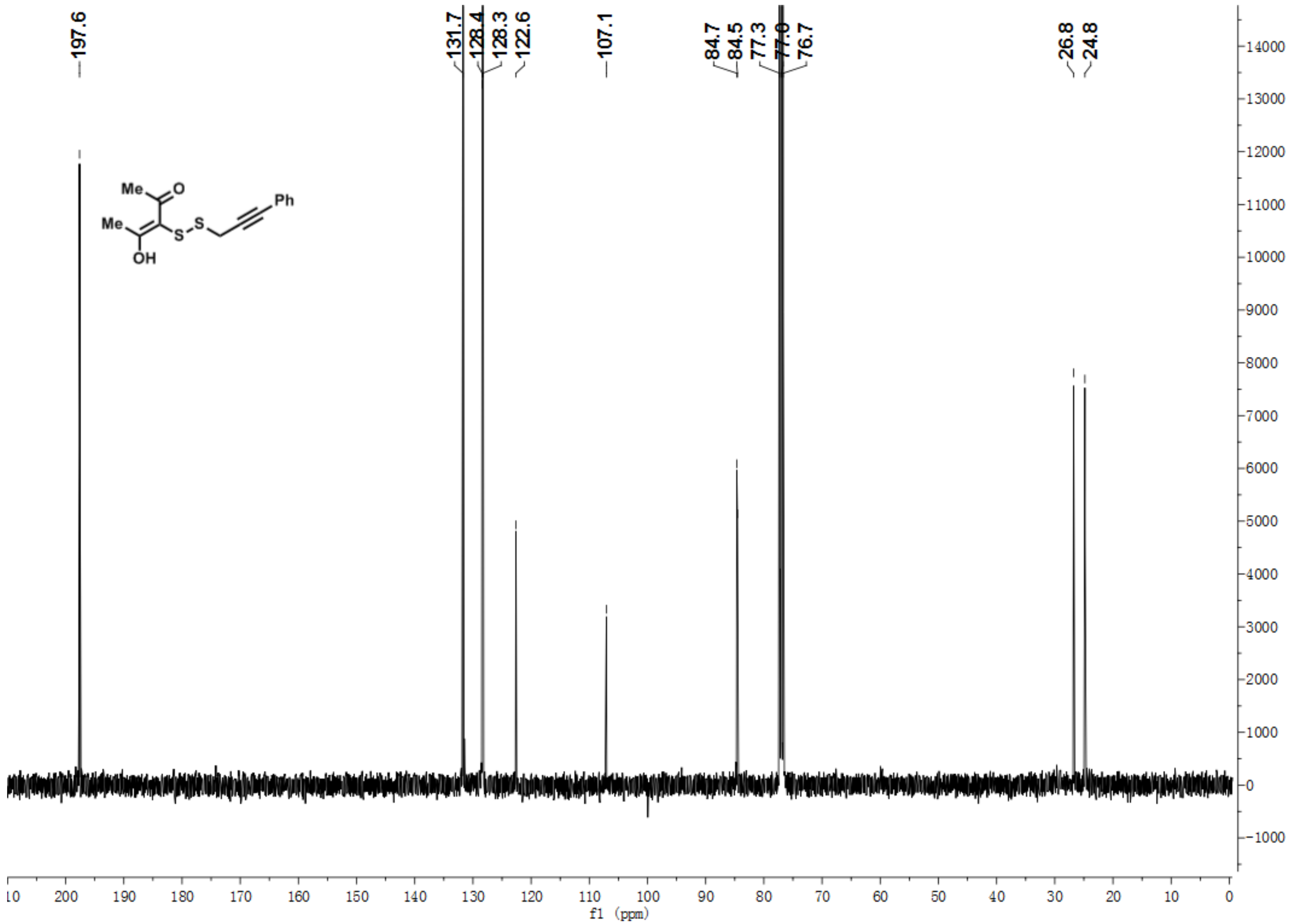

Supplementary Figure 60. ^{13}C NMR spectra for Compound **3h**.

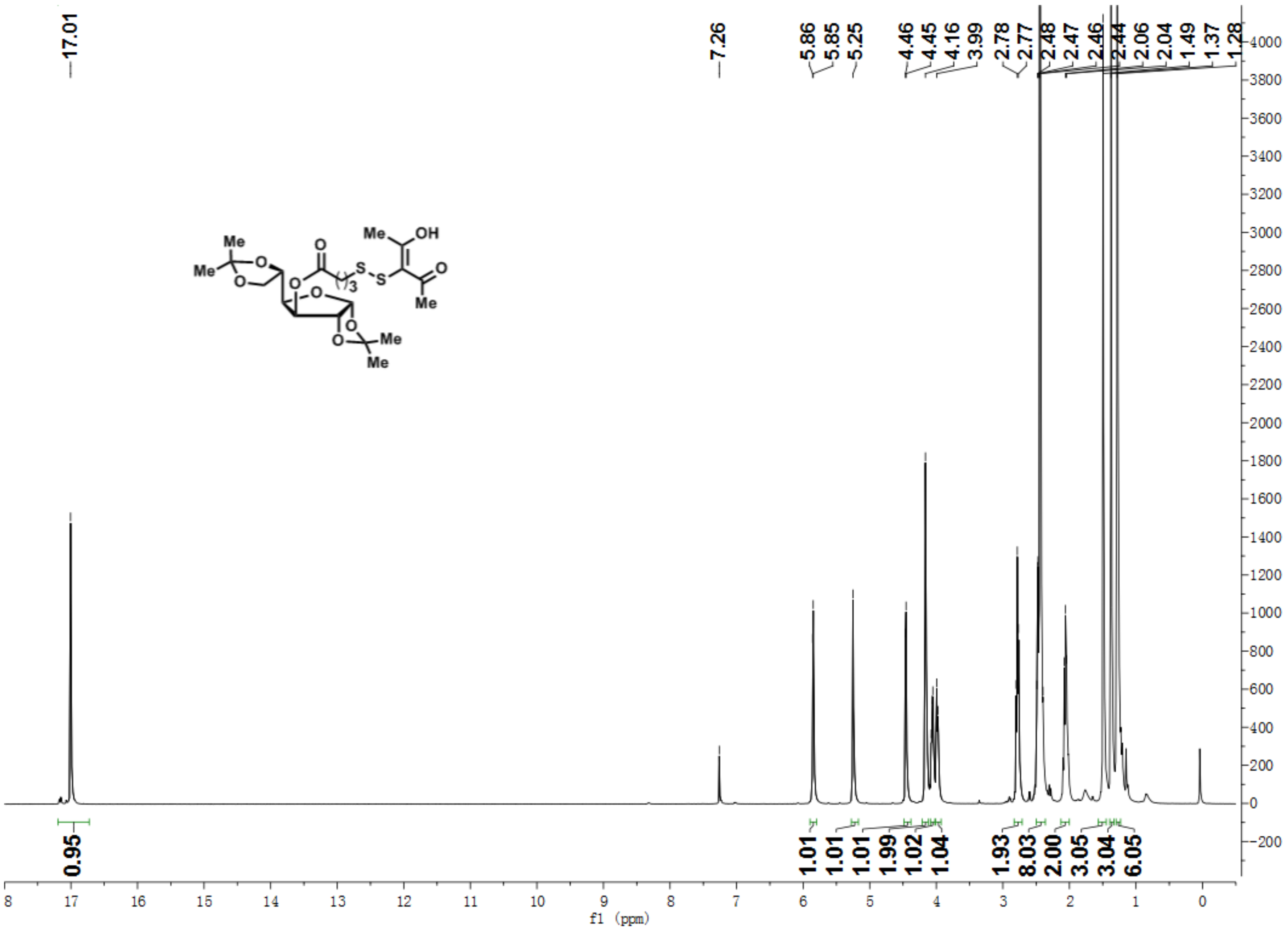

Supplementary Figure 61. ^{1}H NMR spectra for Compound **3i**.

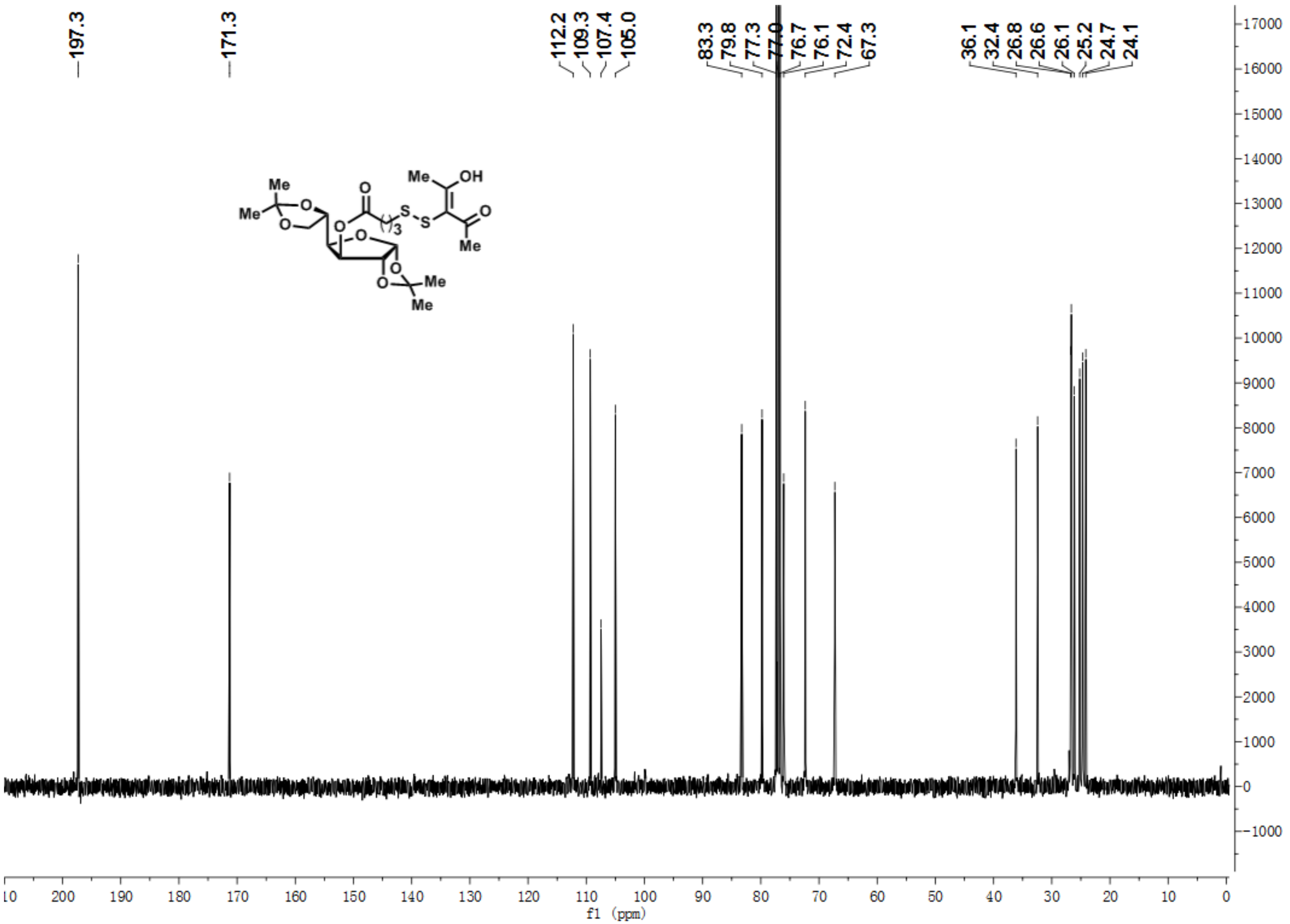

Supplementary Figure 62. ^{13}C NMR spectra for Compound **3i**.

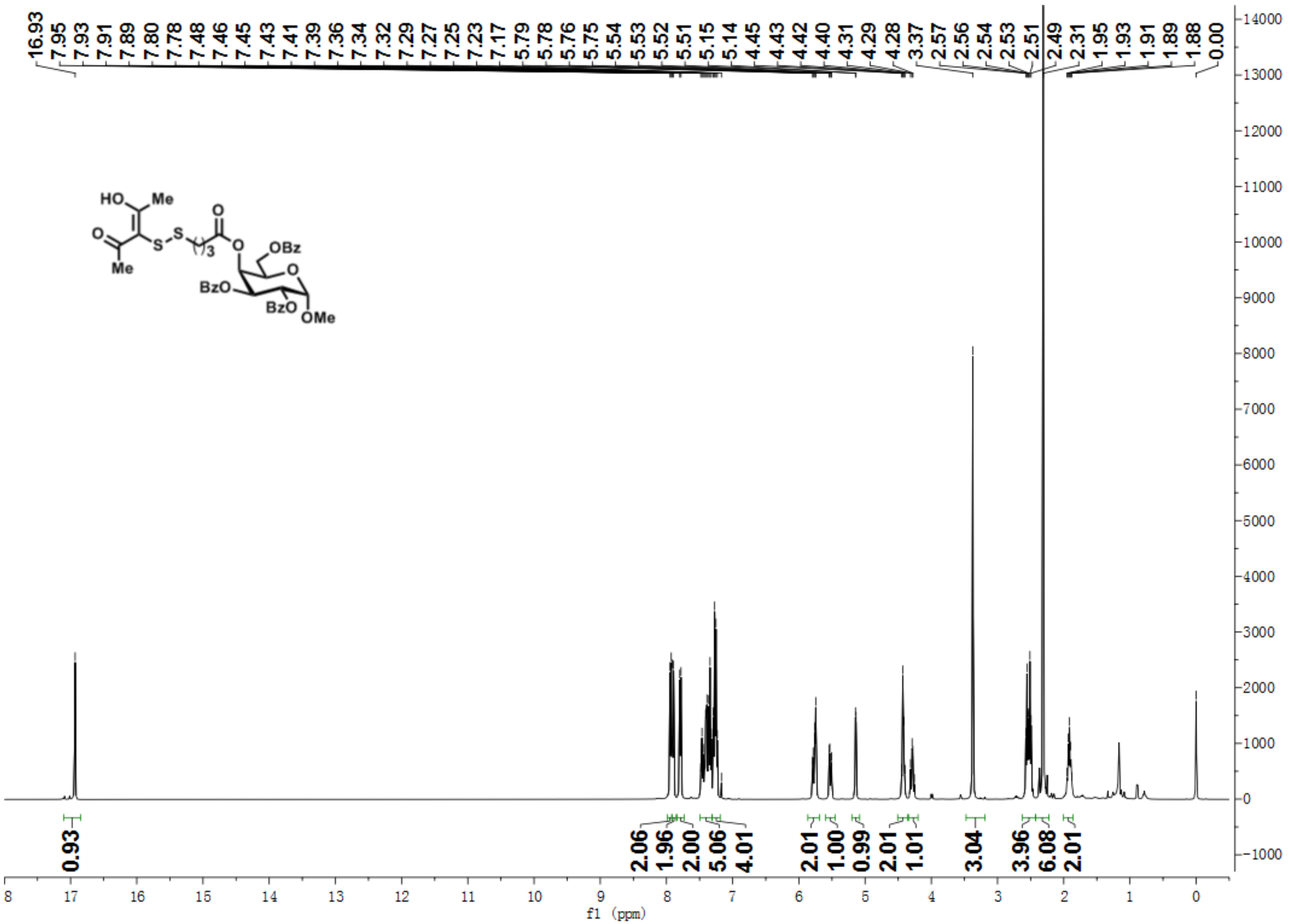

Supplementary Figure 63. ^{1}H NMR spectra for Compound **3j**.

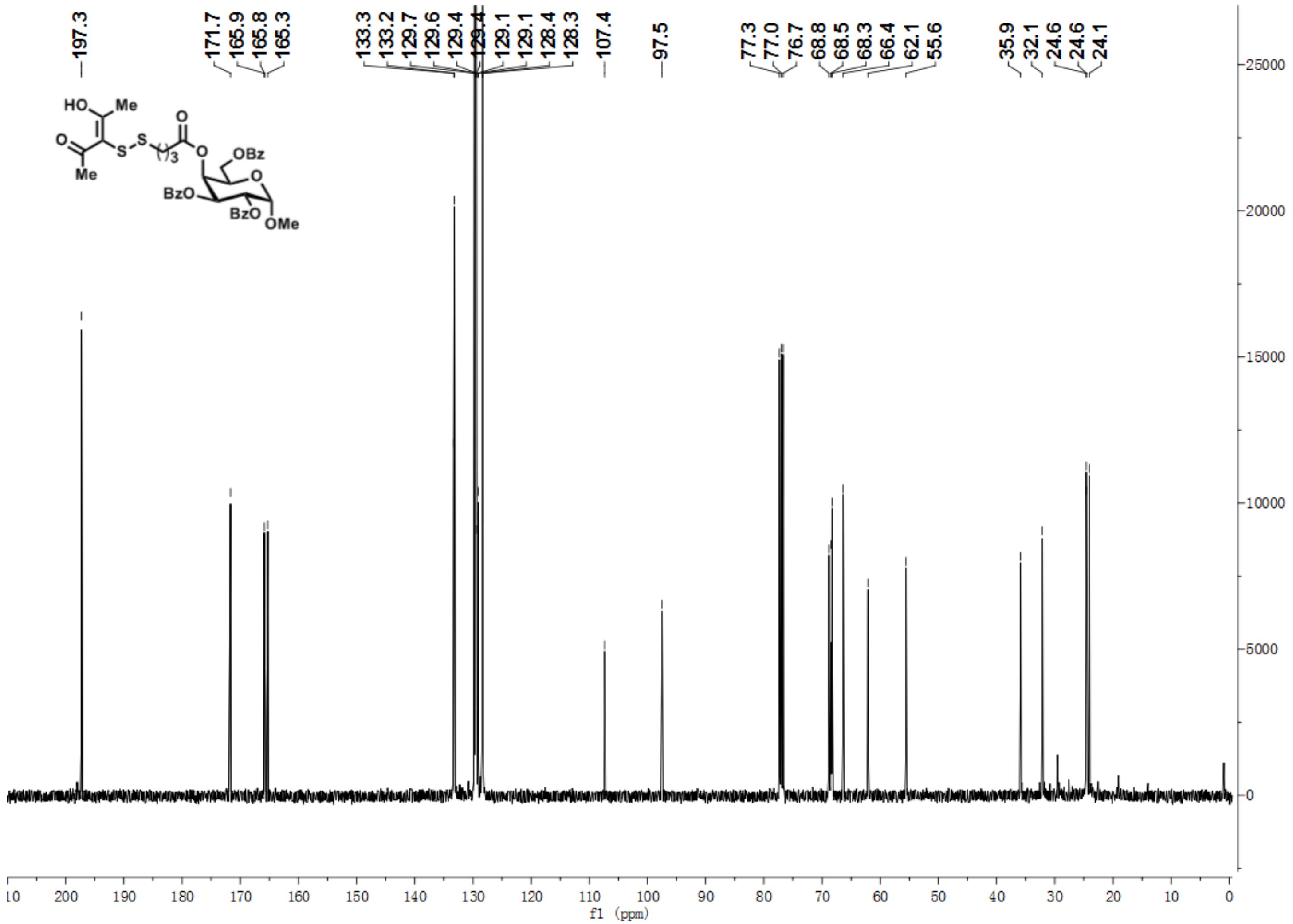

Supplementary Figure 64. ^{13}C NMR spectra for Compound **3j**.

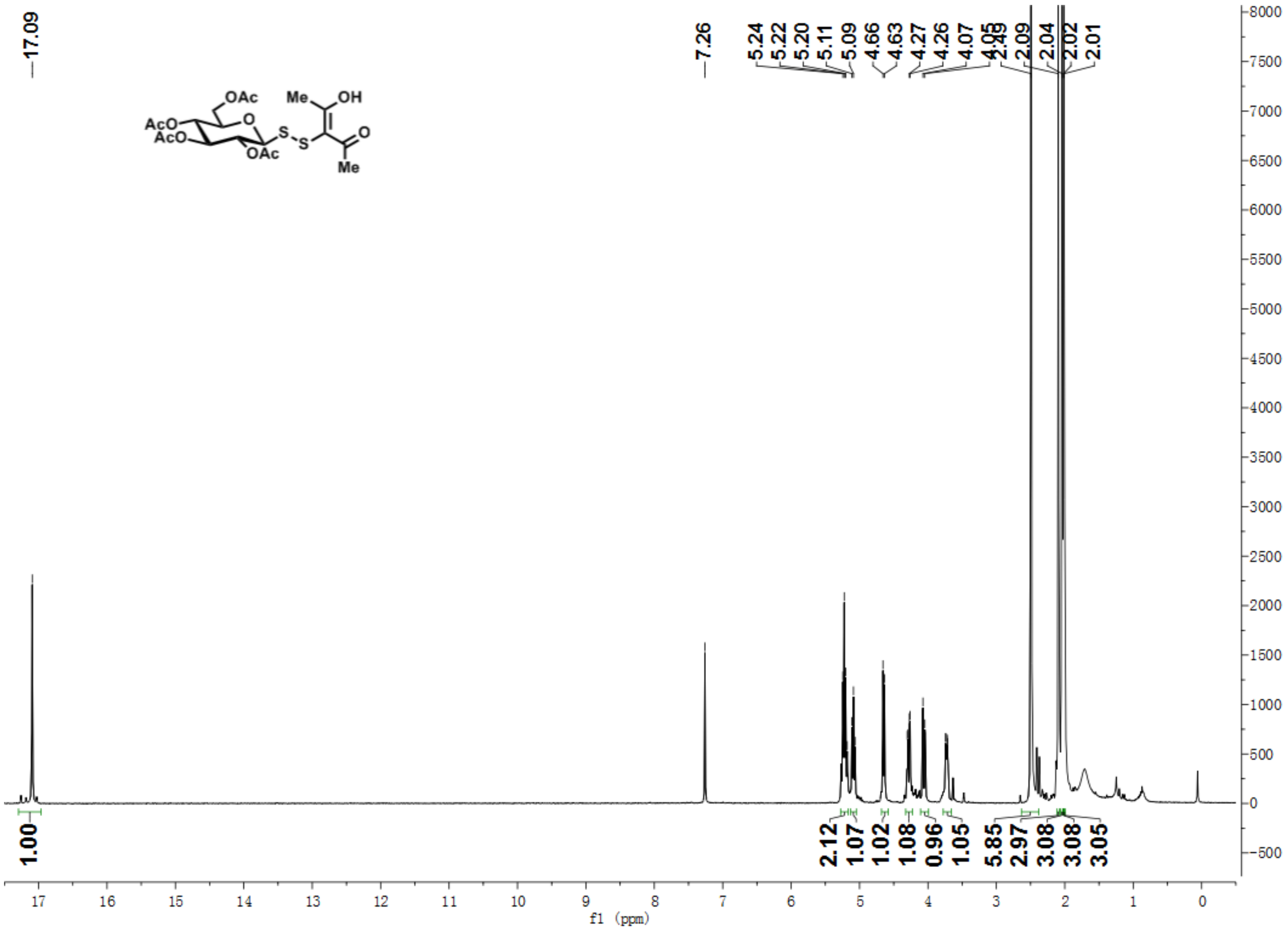

Supplementary Figure 65. ^{1}H NMR spectra for Compound **3k**.

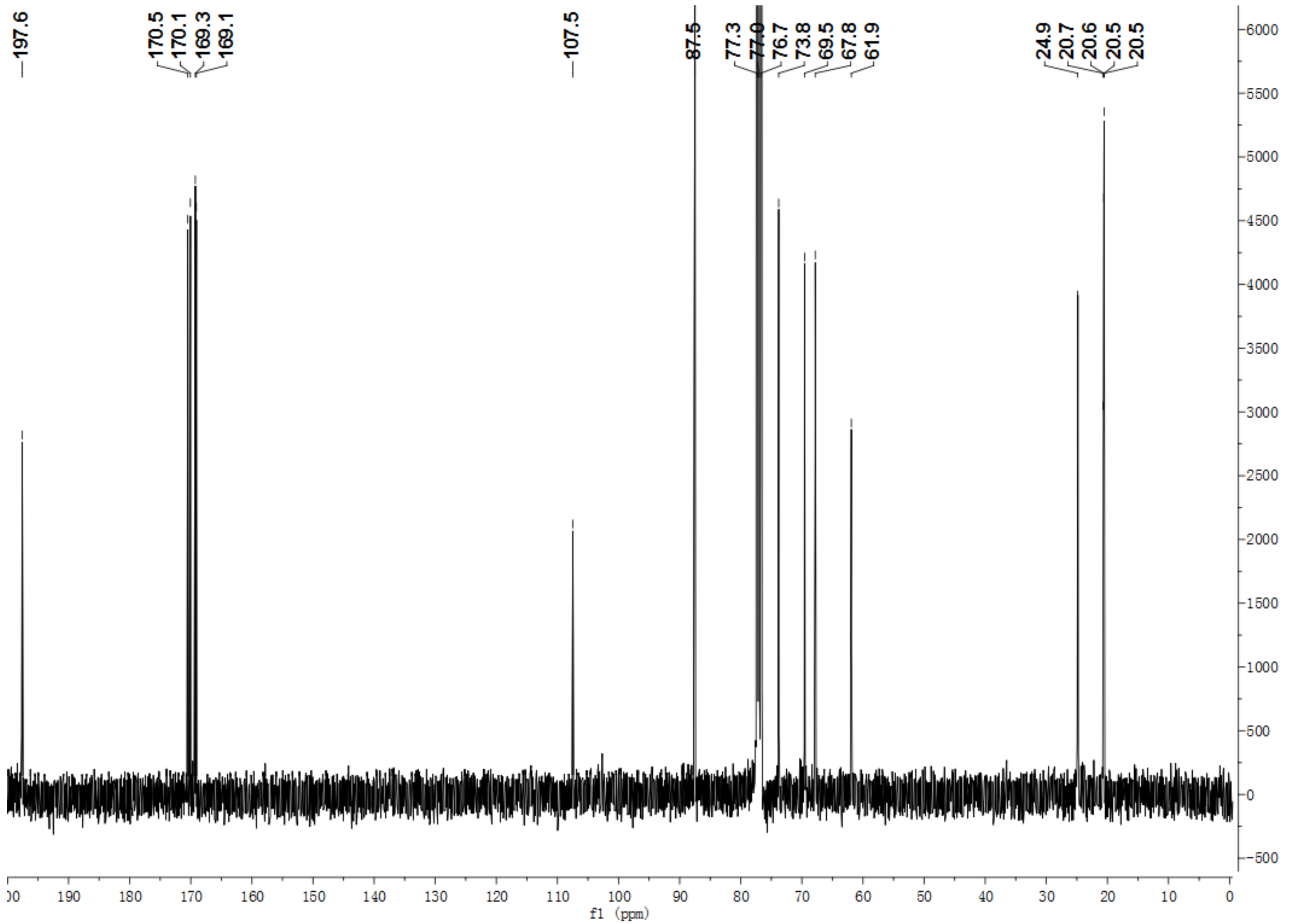

Supplementary Figure 66. ^{13}C NMR spectra for Compound **3k**.

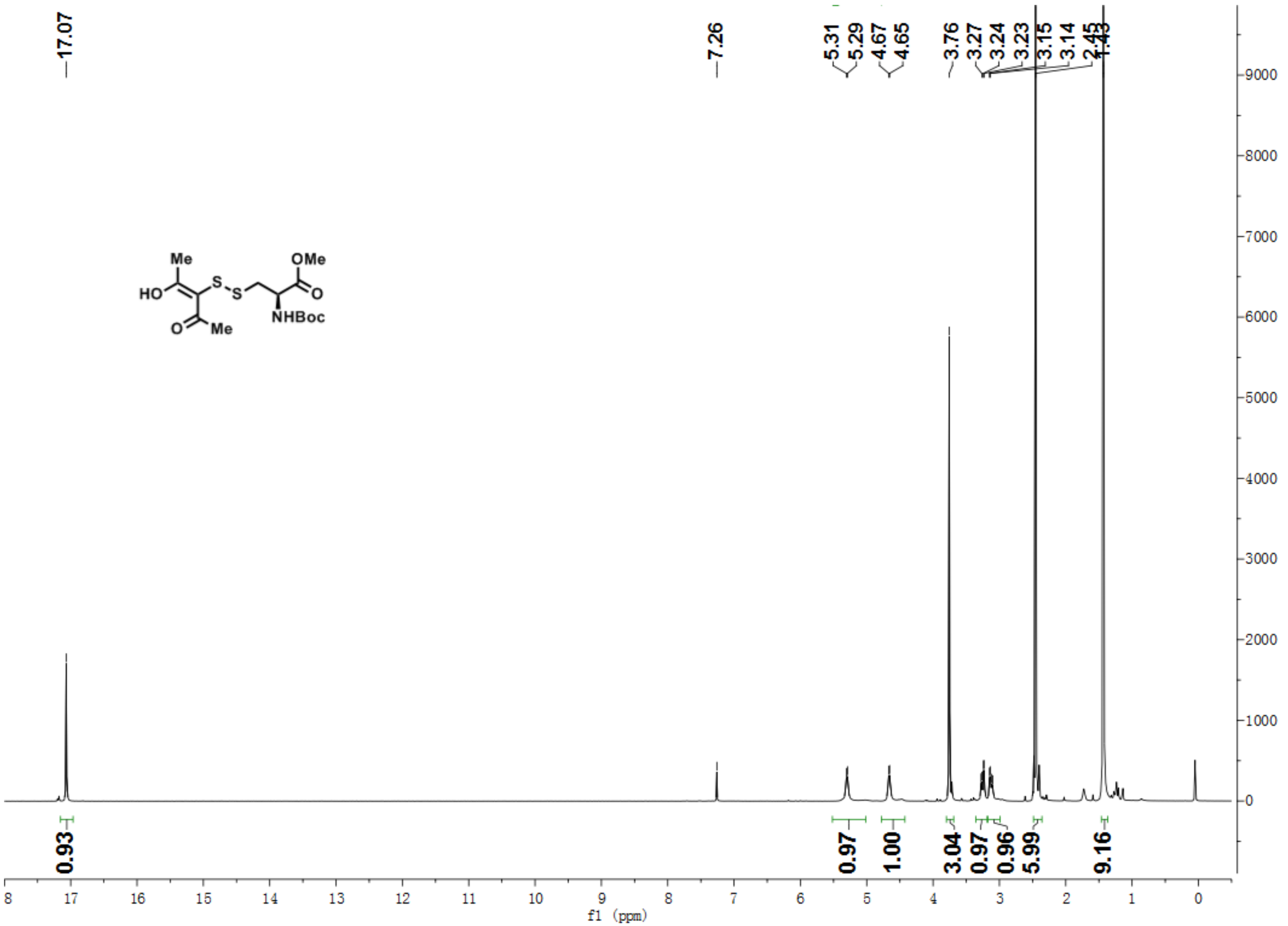

Supplementary Figure 67. ¹H NMR spectra for Compound **3l**.

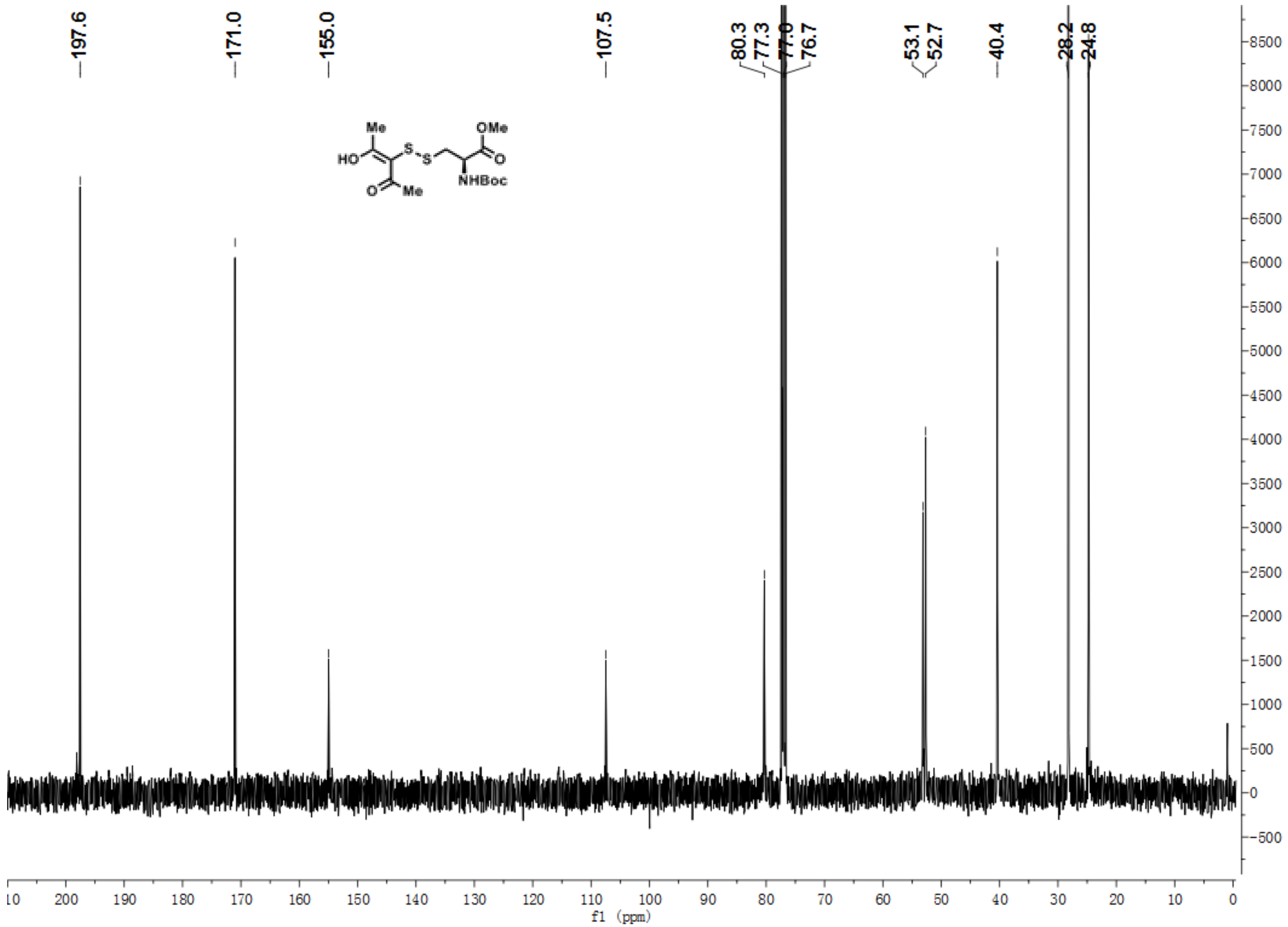

Supplementary Figure 68. ^{13}C NMR spectra for Compound **3l**.

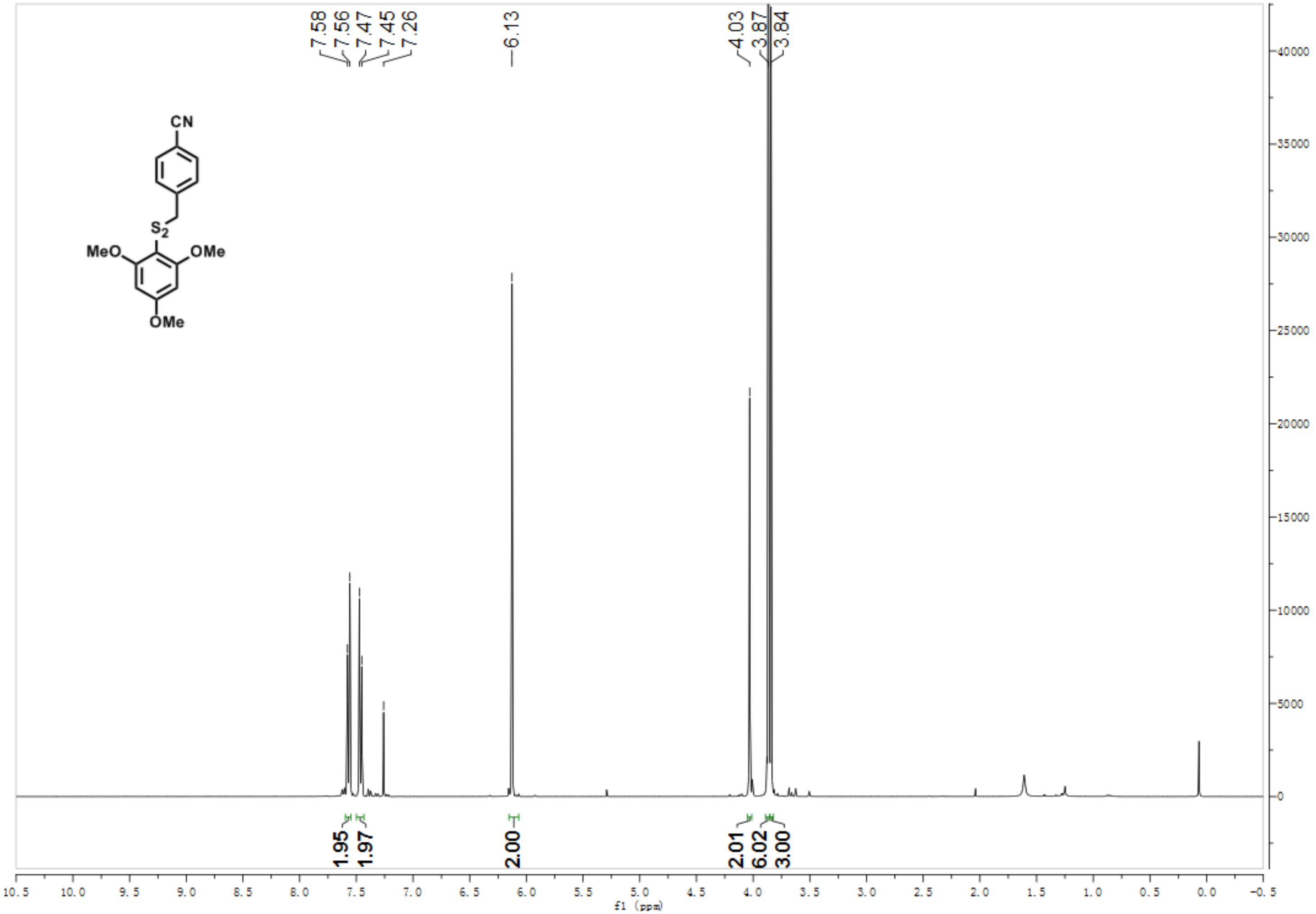

Supplementary Figure 69. ^{1}H NMR spectra for Compound **4a**.

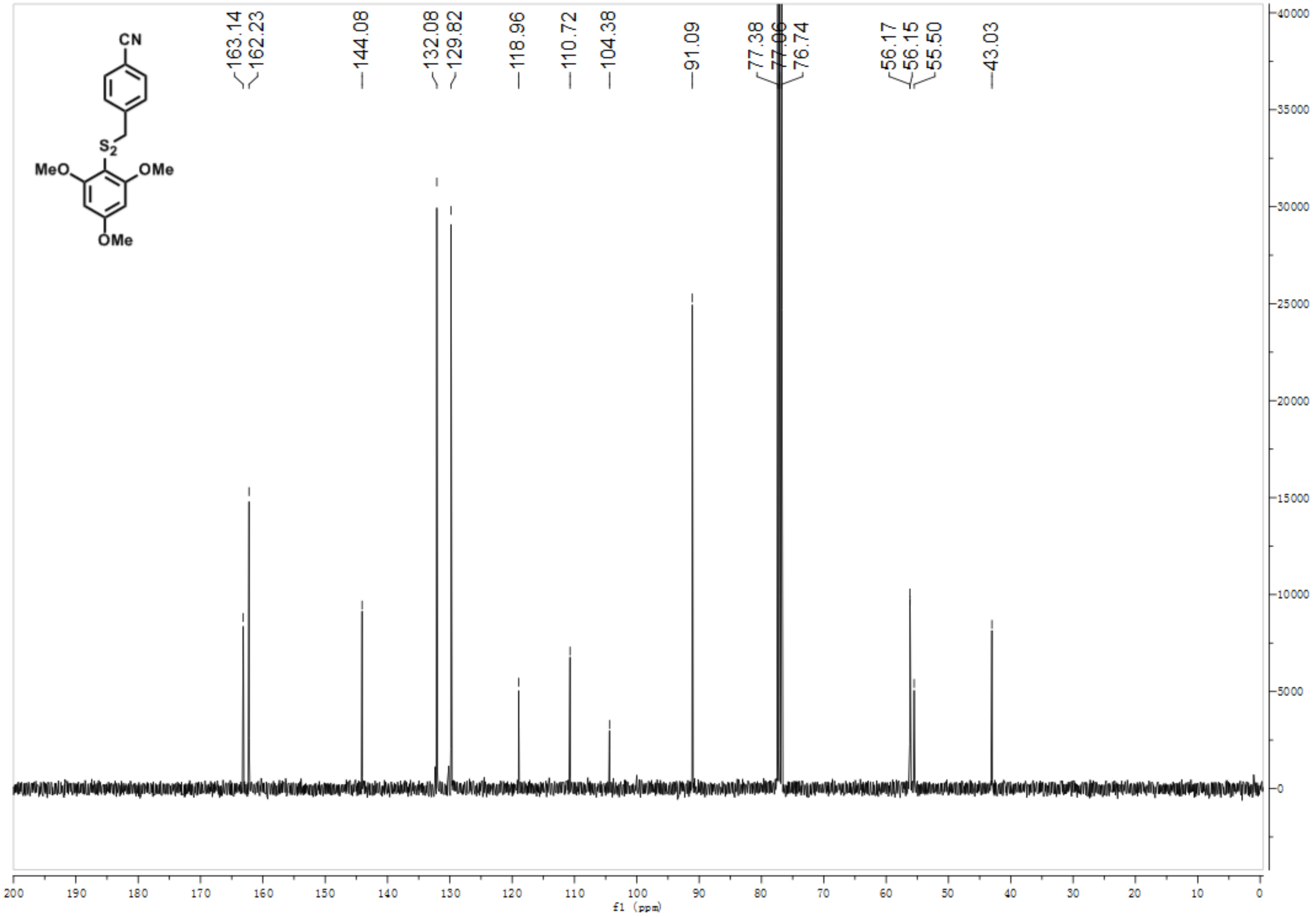

Supplementary Figure 70. ^{13}C NMR spectra for Compound **4a**.

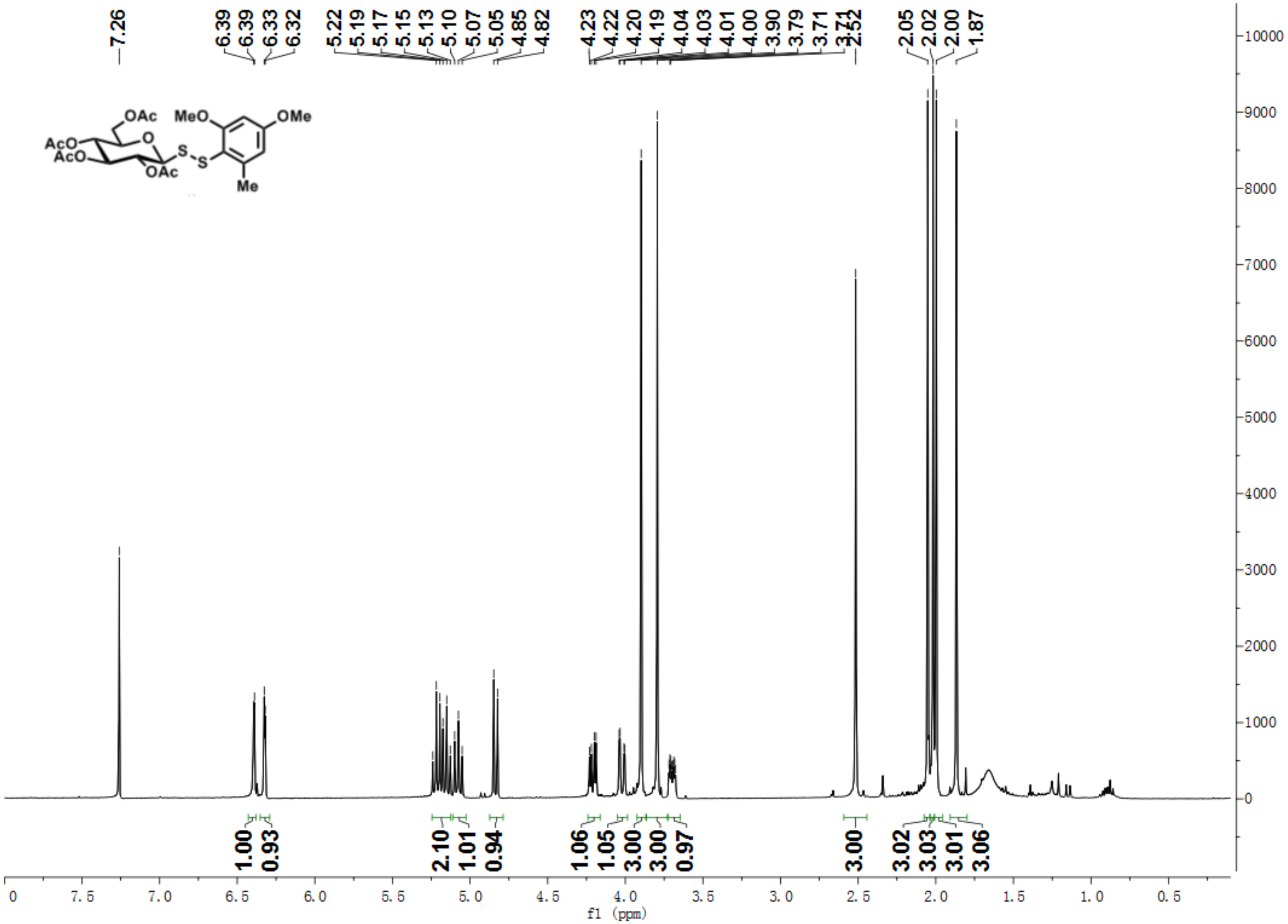

Supplementary Figure 71. ^{1}H NMR spectra for Compound **4b**.

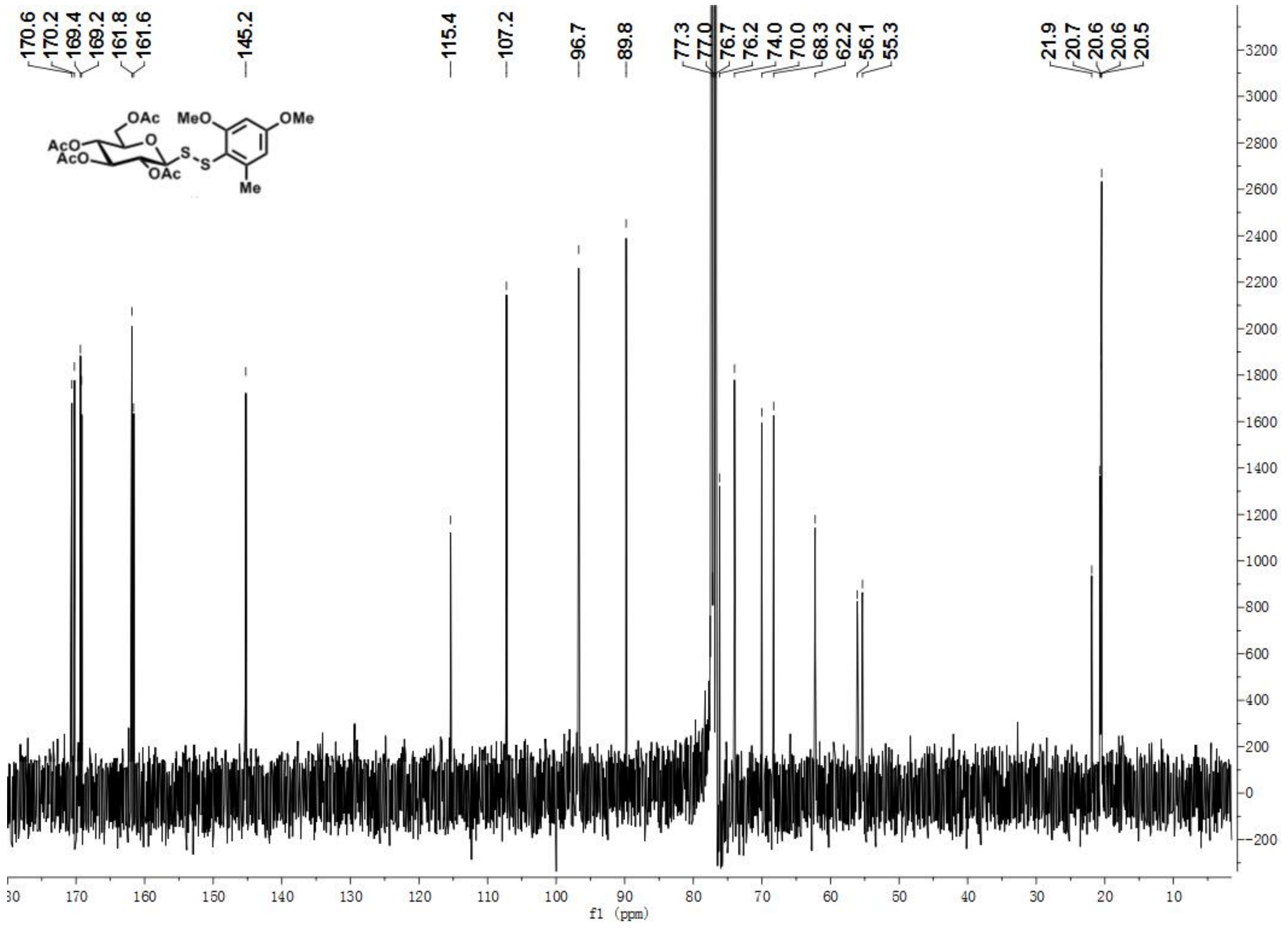

Supplementary Figure 72. ^{13}C NMR spectra for Compound **4b**.

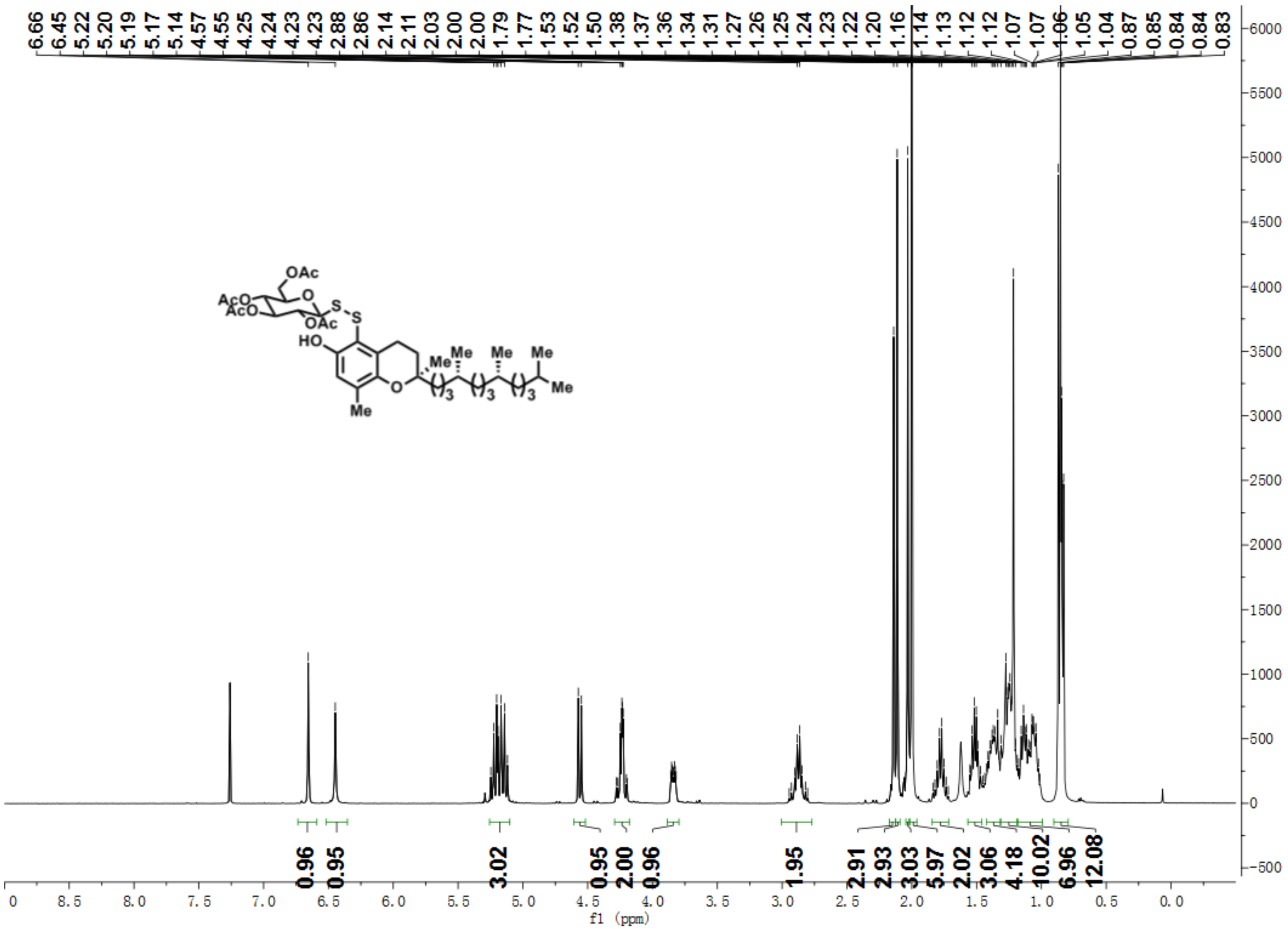

Supplementary Figure 73. ^{1}H NMR spectra for Compound **4c**.

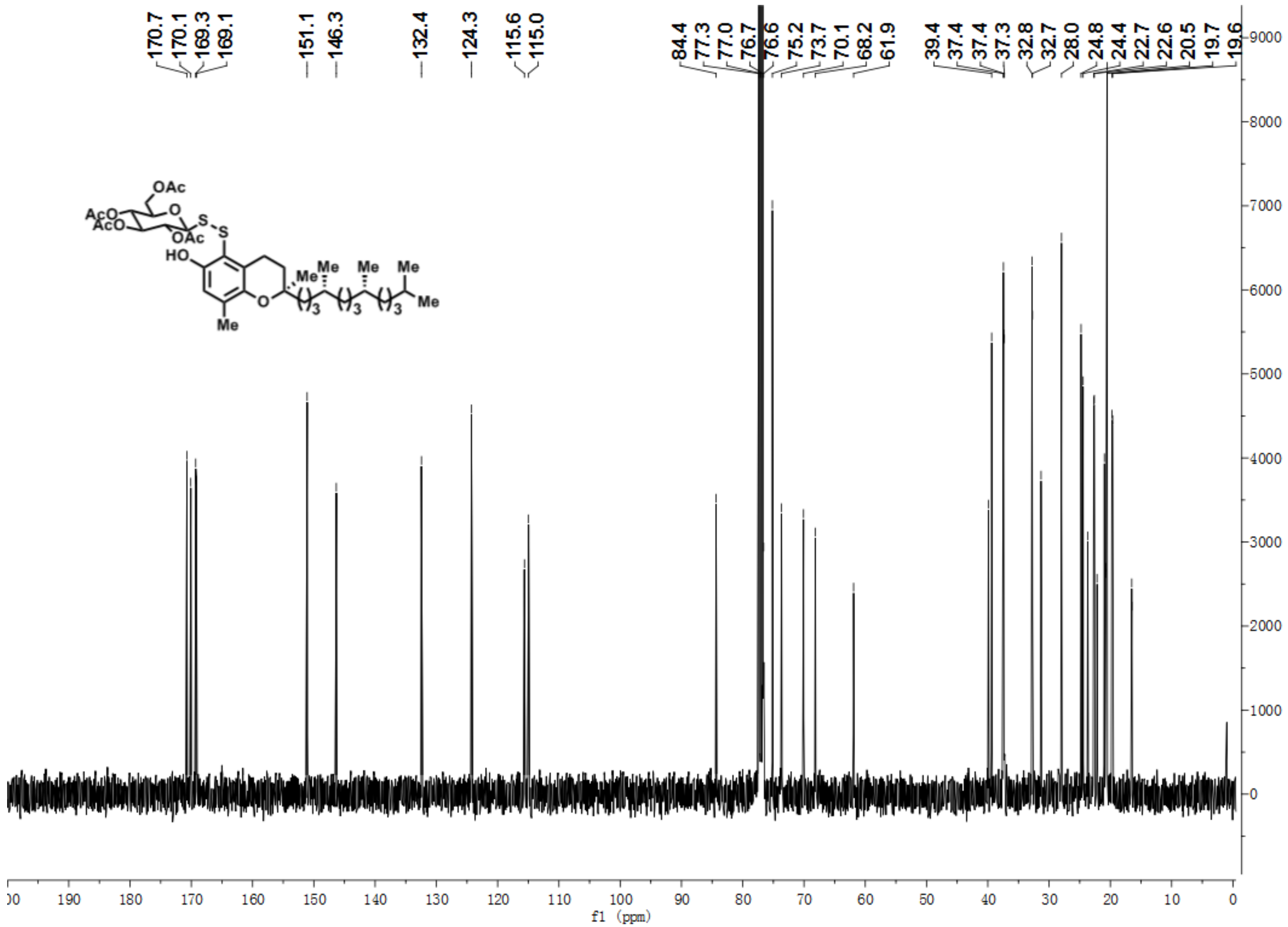

Supplementary Figure 74. ^{13}C NMR spectra for Compound **4c**.

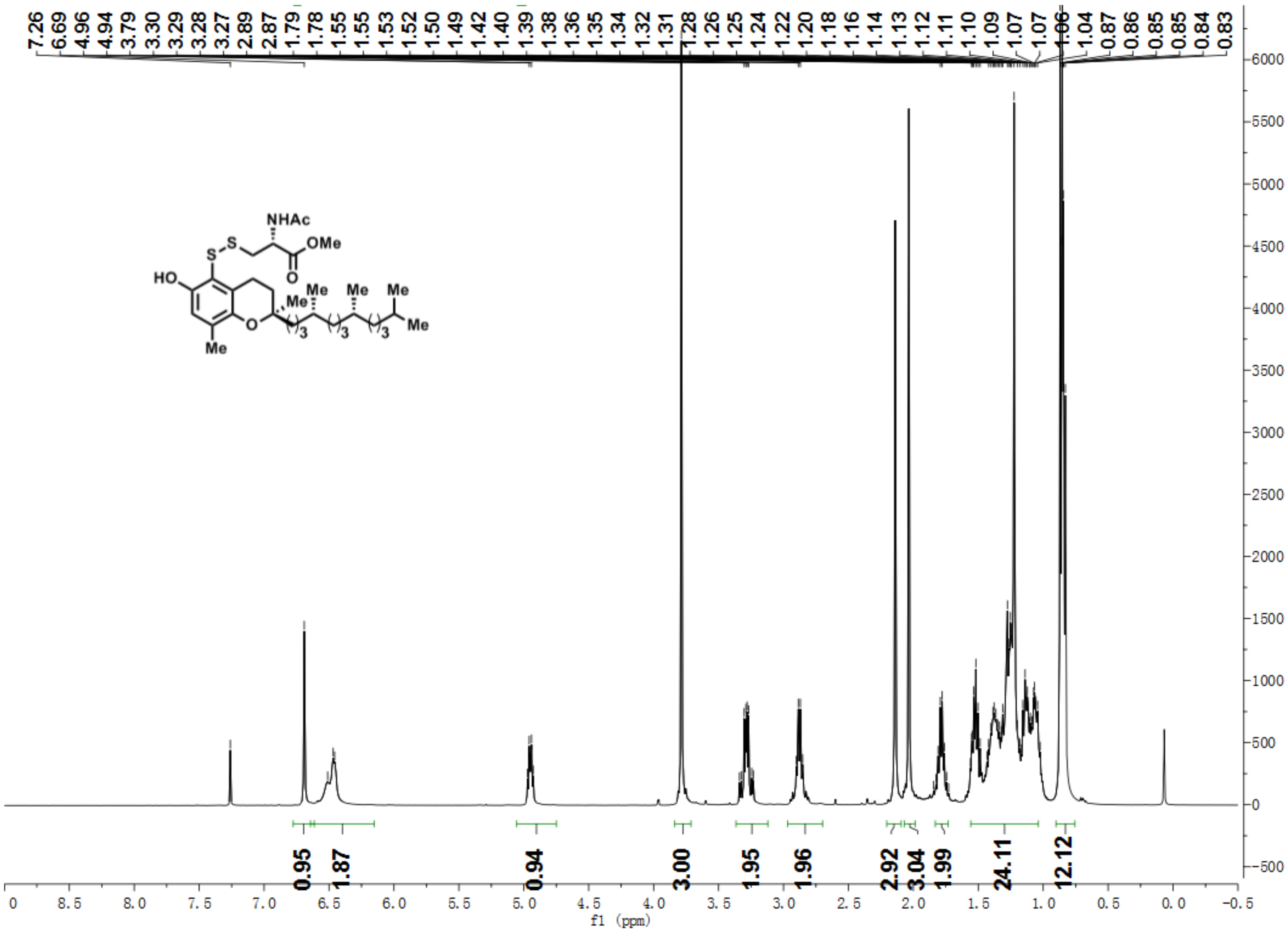

Supplementary Figure 75. ^{1}H NMR spectra for Compound **4d**.

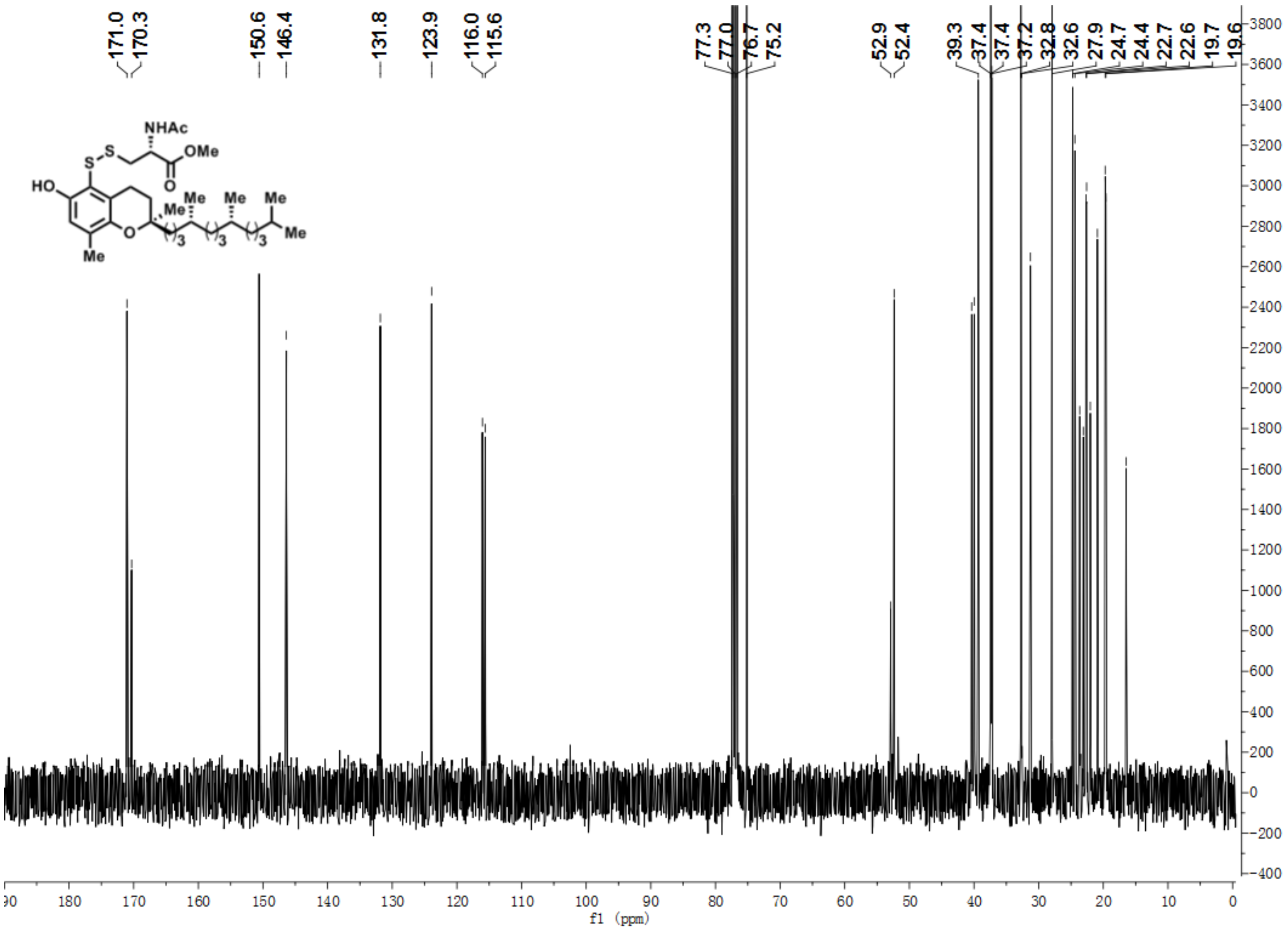

Supplementary Figure 76. ^{13}C NMR spectra for Compound **4d**.

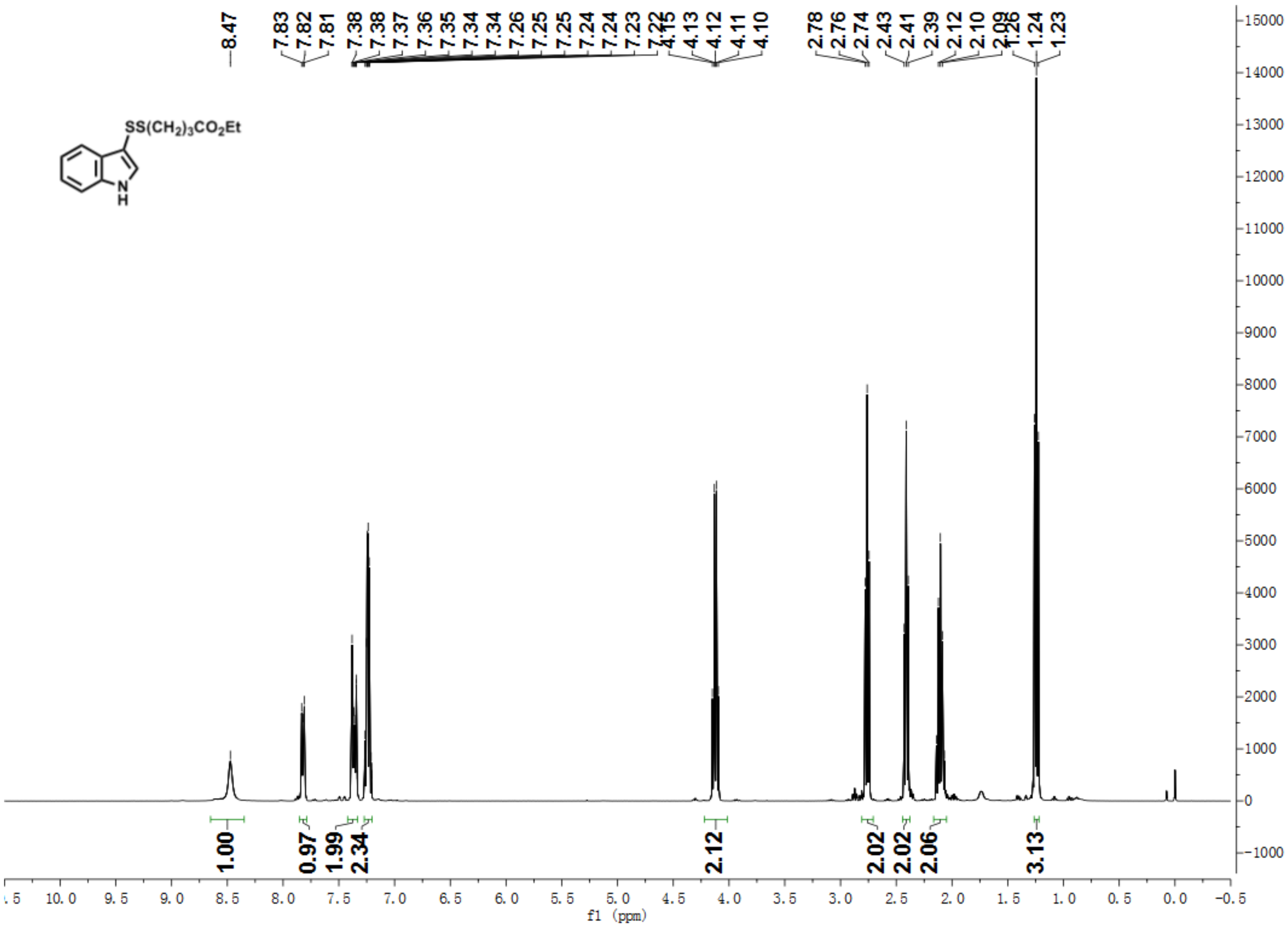

Supplementary Figure 77. ^{1}H NMR spectra for Compound **5a**.

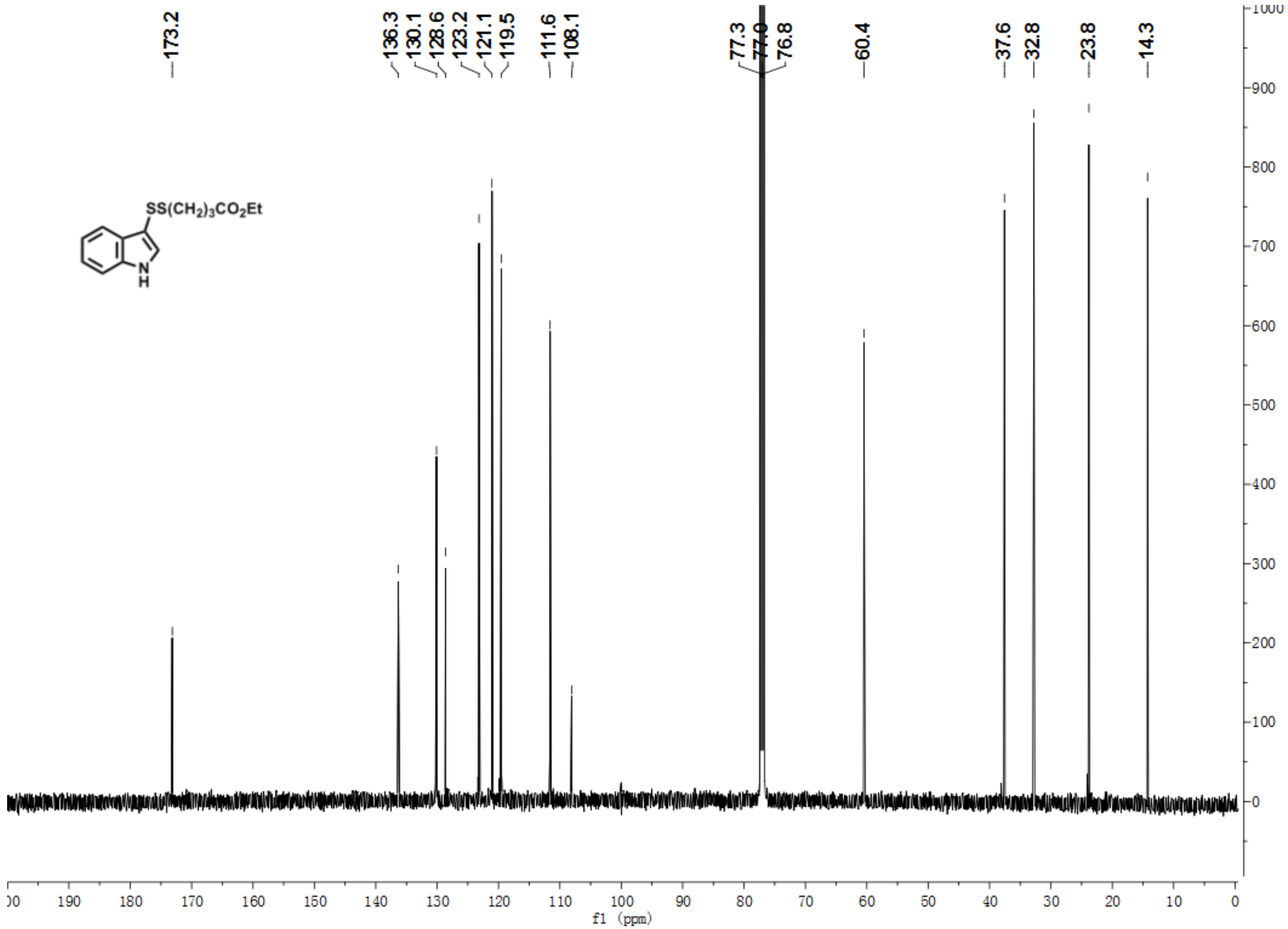

Supplementary Figure 78. ^{13}C NMR spectra for Compound **5a**.

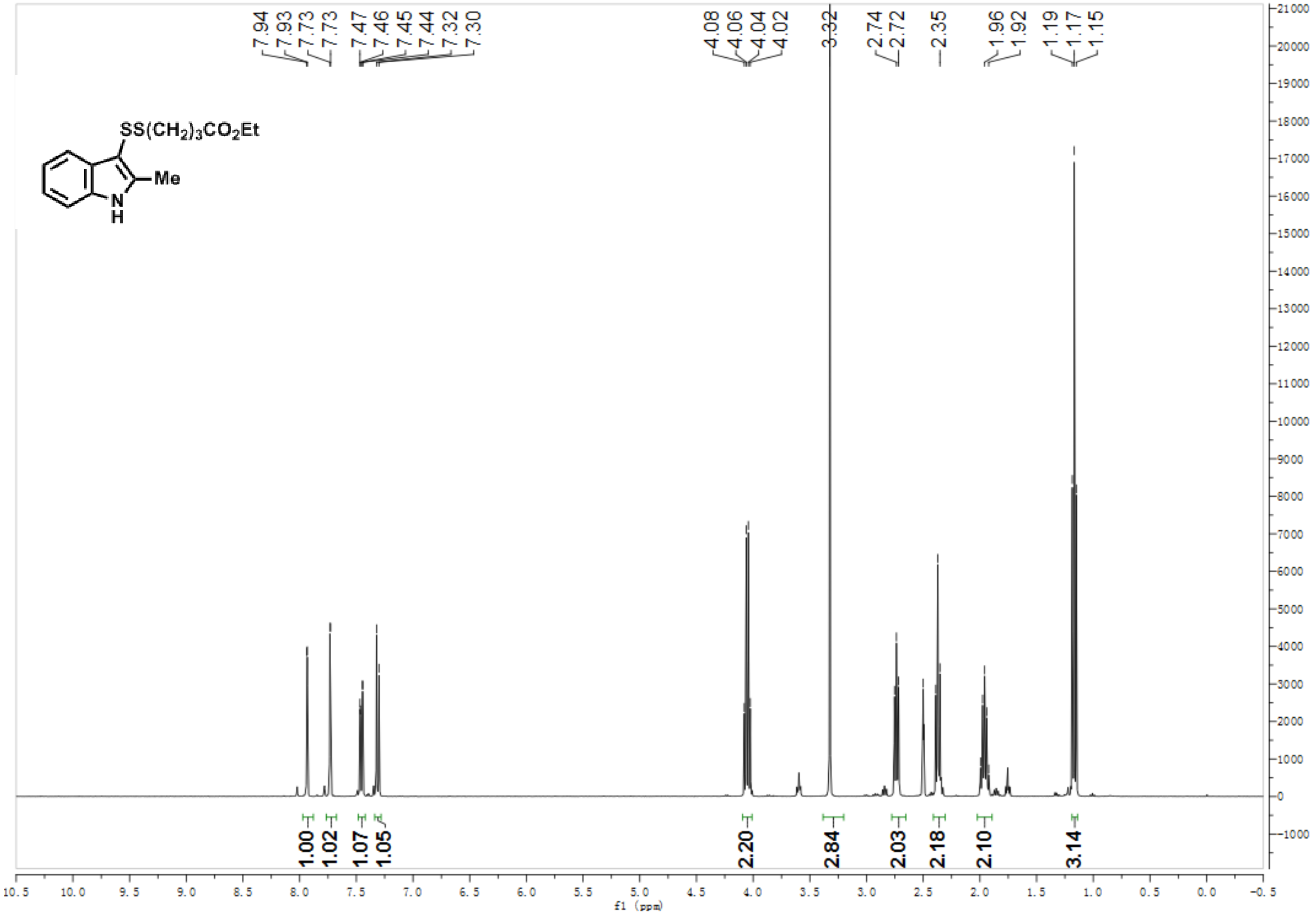

Supplementary Figure 79. ^{1}H NMR spectra for Compound **5b**.

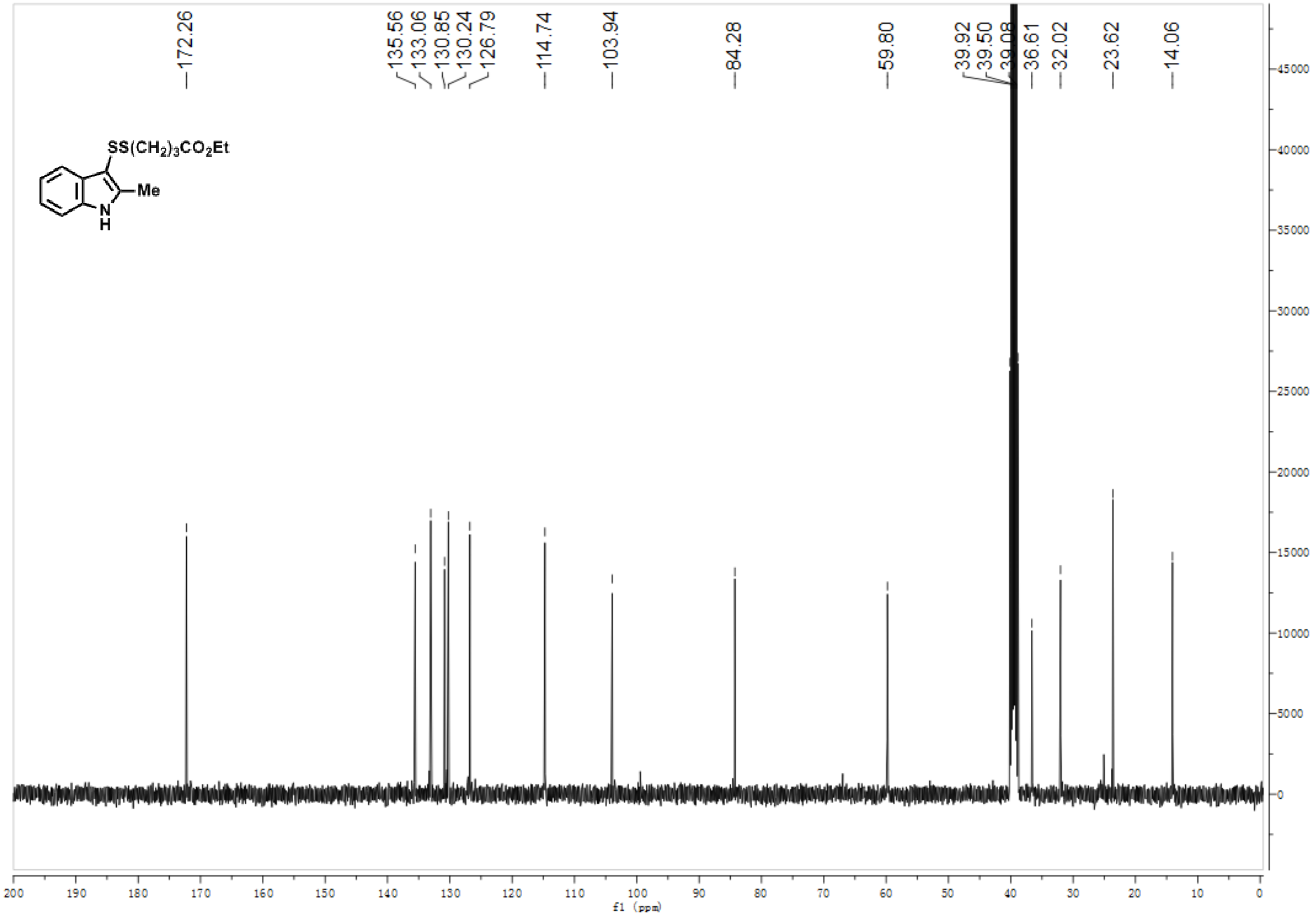

Supplementary Figure 80. ^{13}C NMR spectra for Compound **5b**.

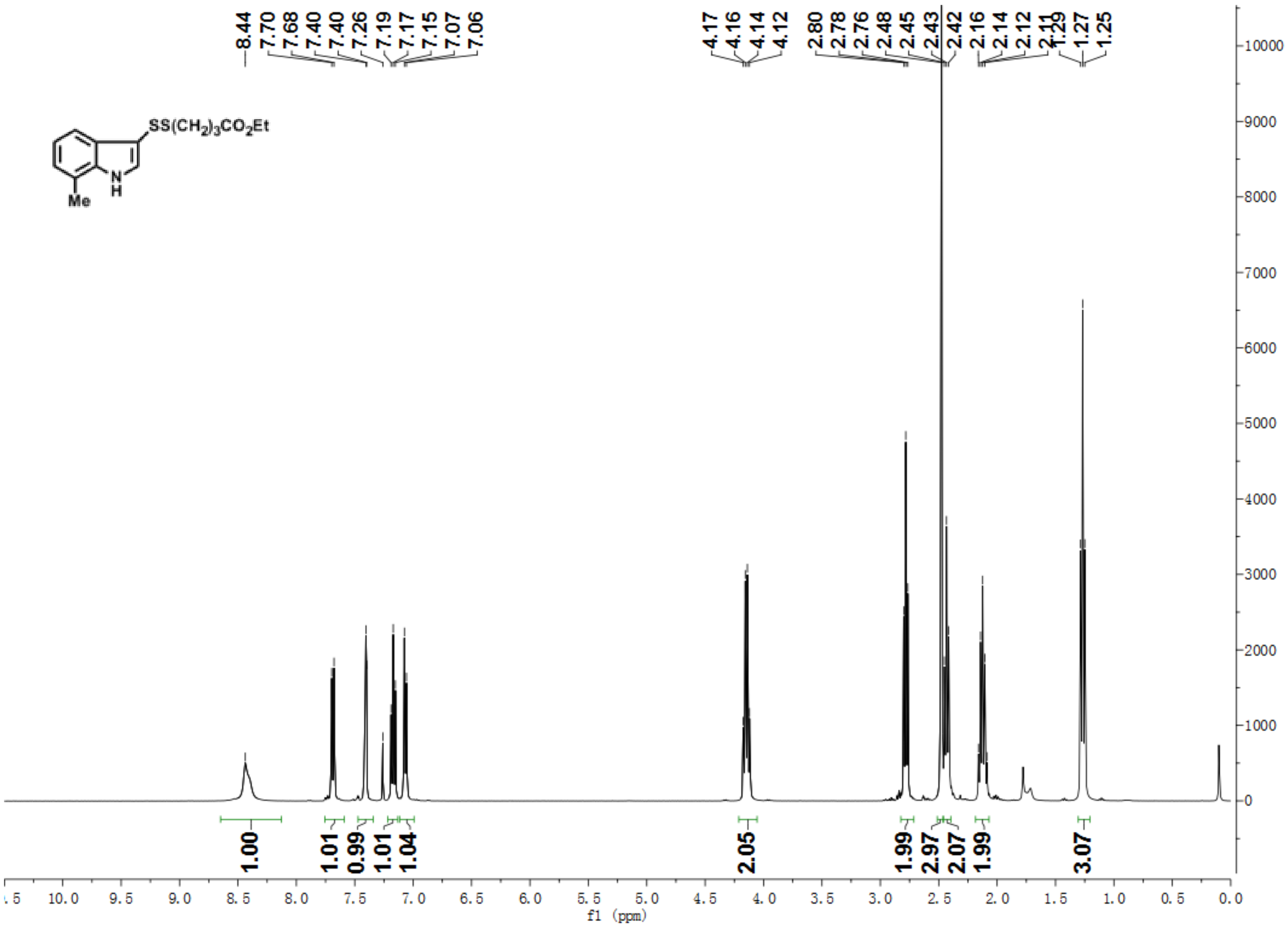

Supplementary Figure 81. ^{1}H NMR spectra for Compound **5c**.

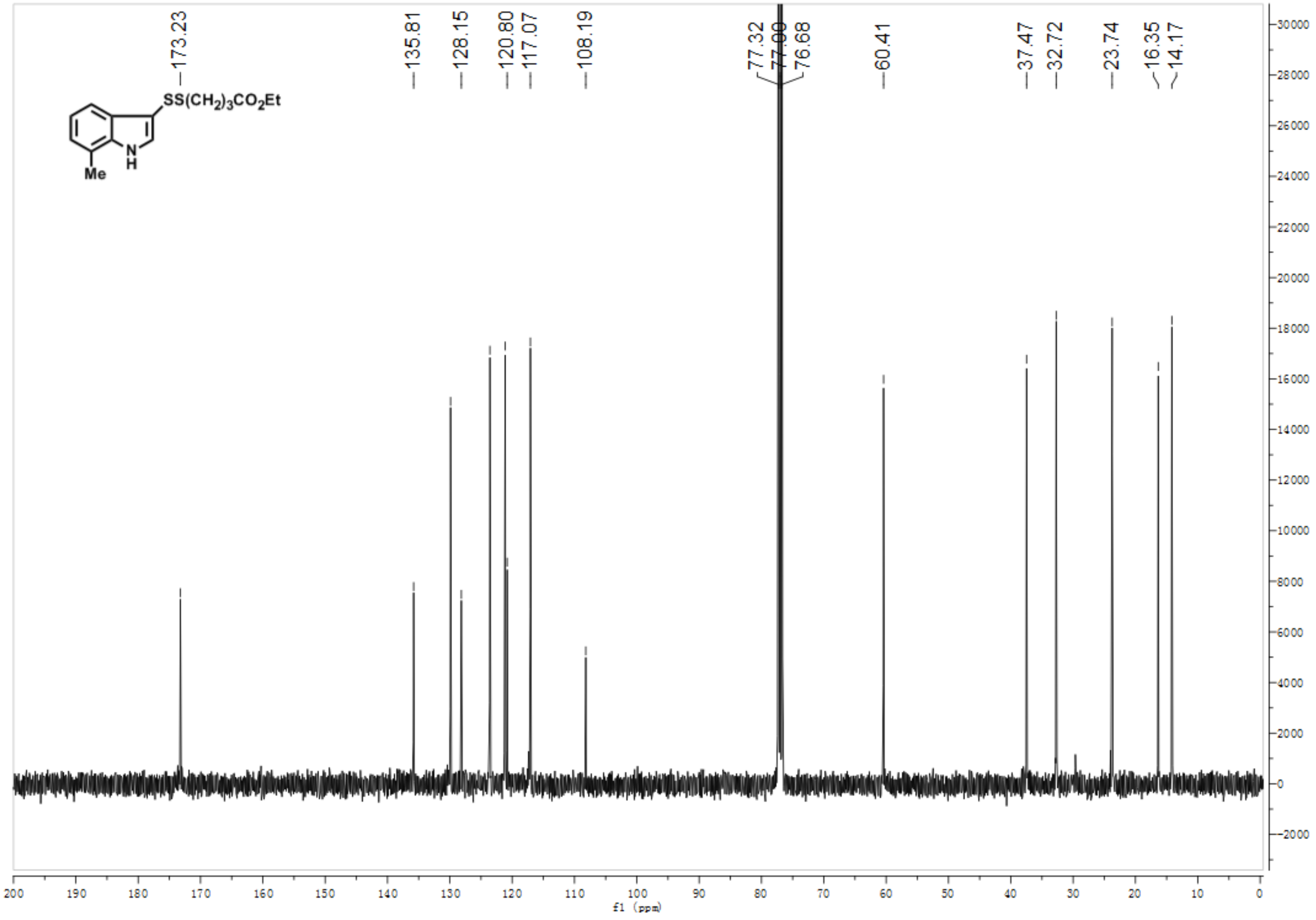

Supplementary Figure 82. ^{13}C NMR spectra for Compound **5c**.

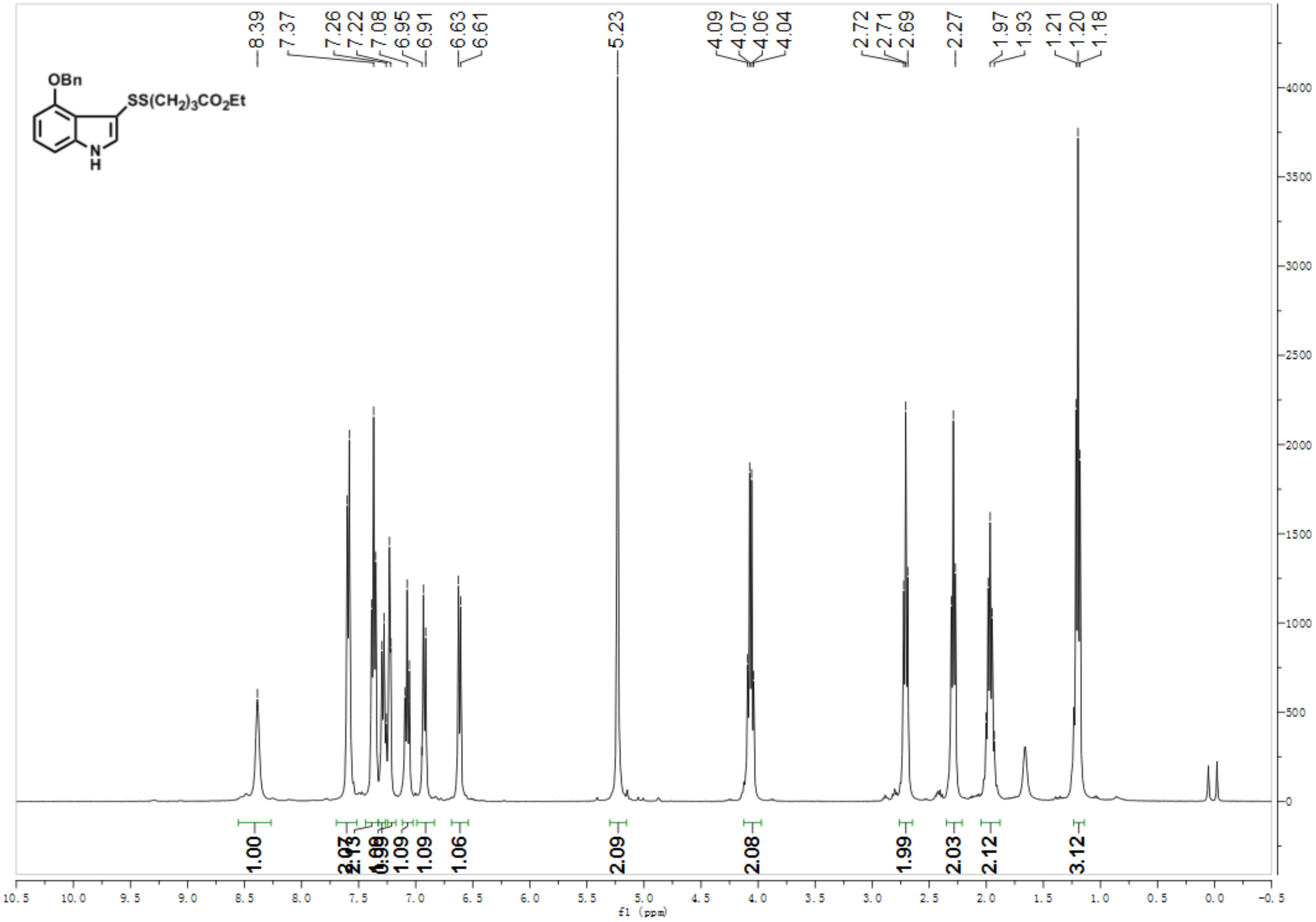

Supplementary Figure 83. ^{1}H NMR spectra for Compound **5d**.

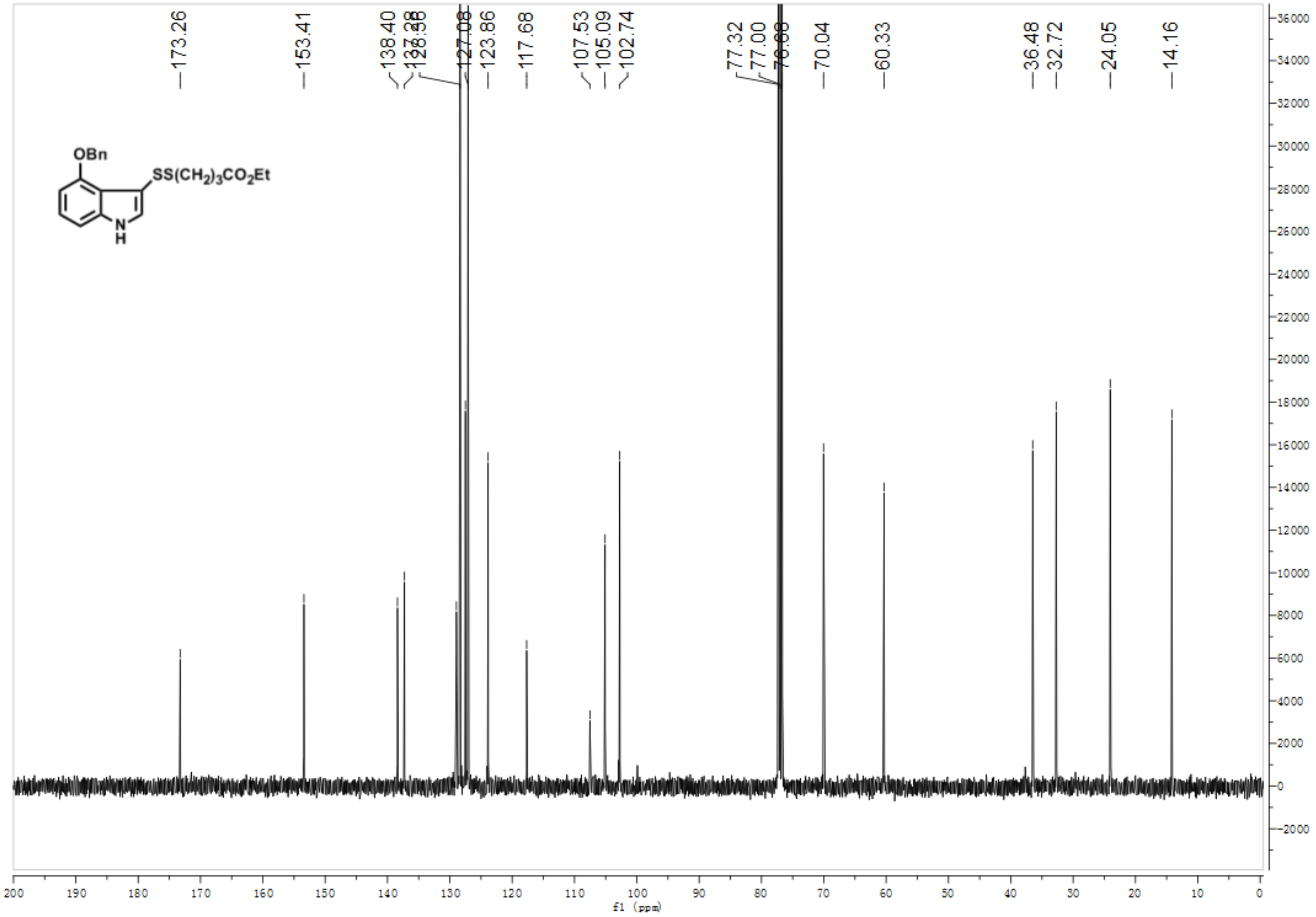

Supplementary Figure 84. ^{1}H NMR spectra for Compound **5d**.

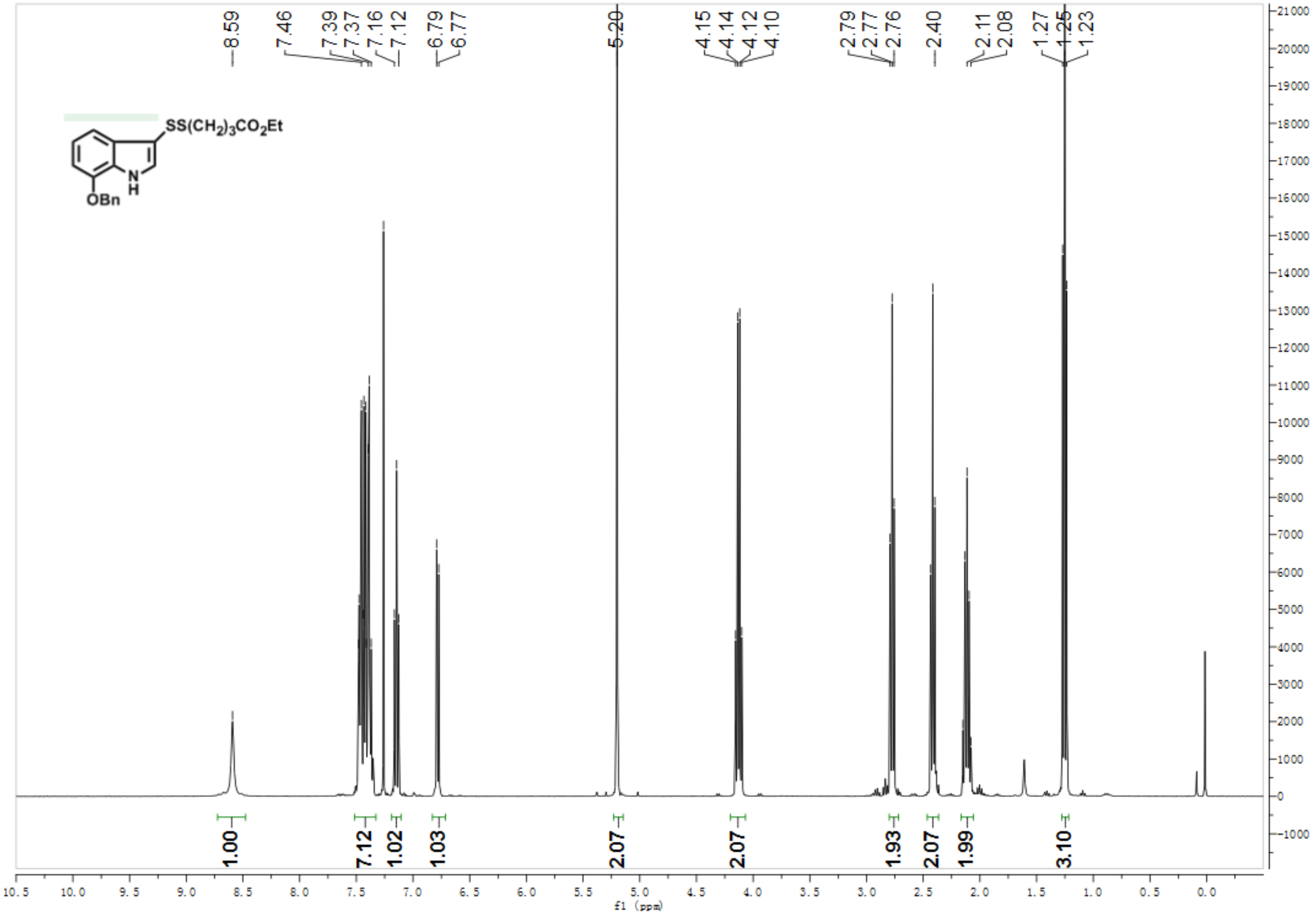

Supplementary Figure 85. ^{1}H NMR spectra for Compound **5e**.

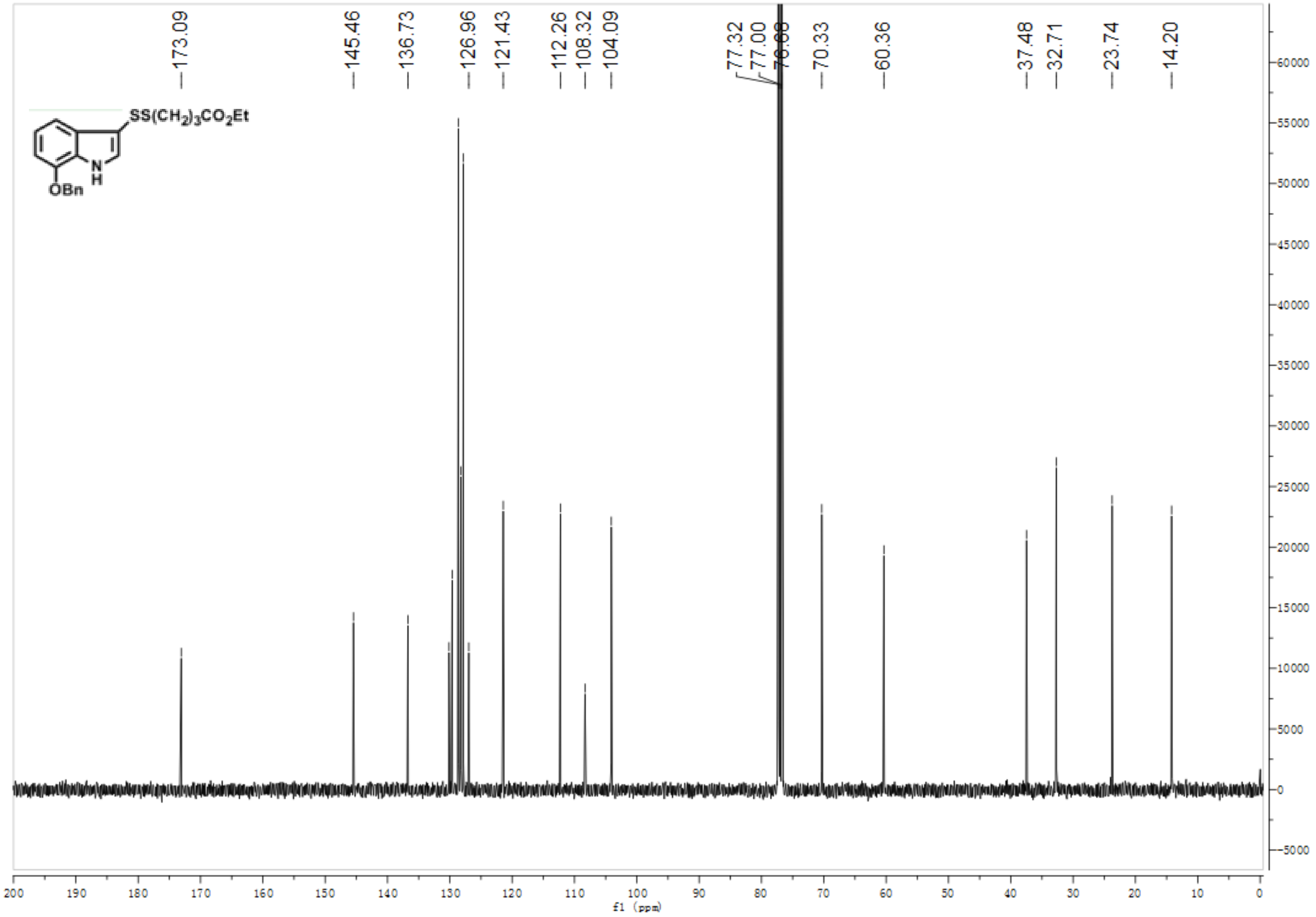

Supplementary Figure 86. ^{13}C NMR spectra for Compound **5e**.

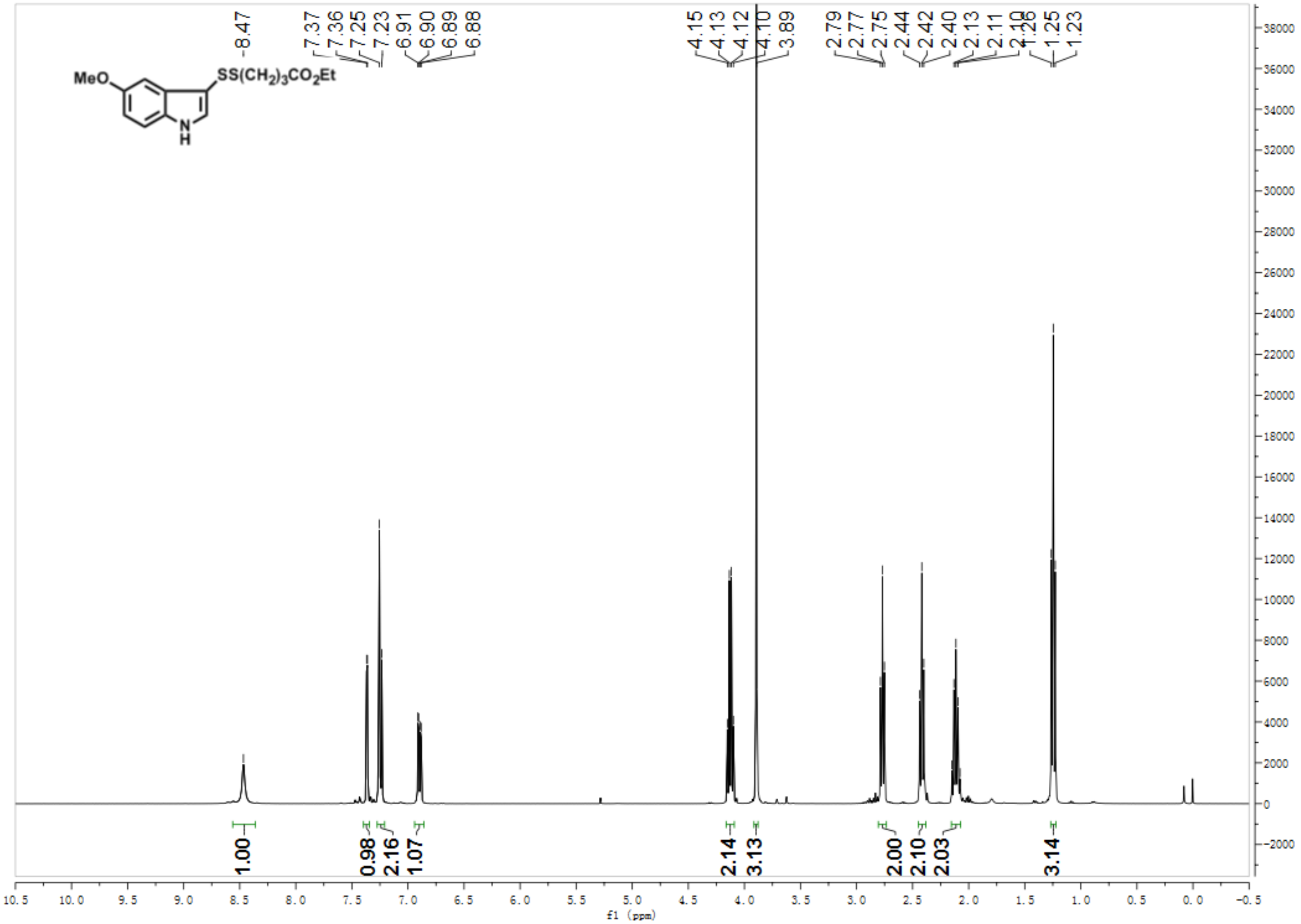

Supplementary Figure 87. ^{1}H NMR spectra for Compound **5f**.

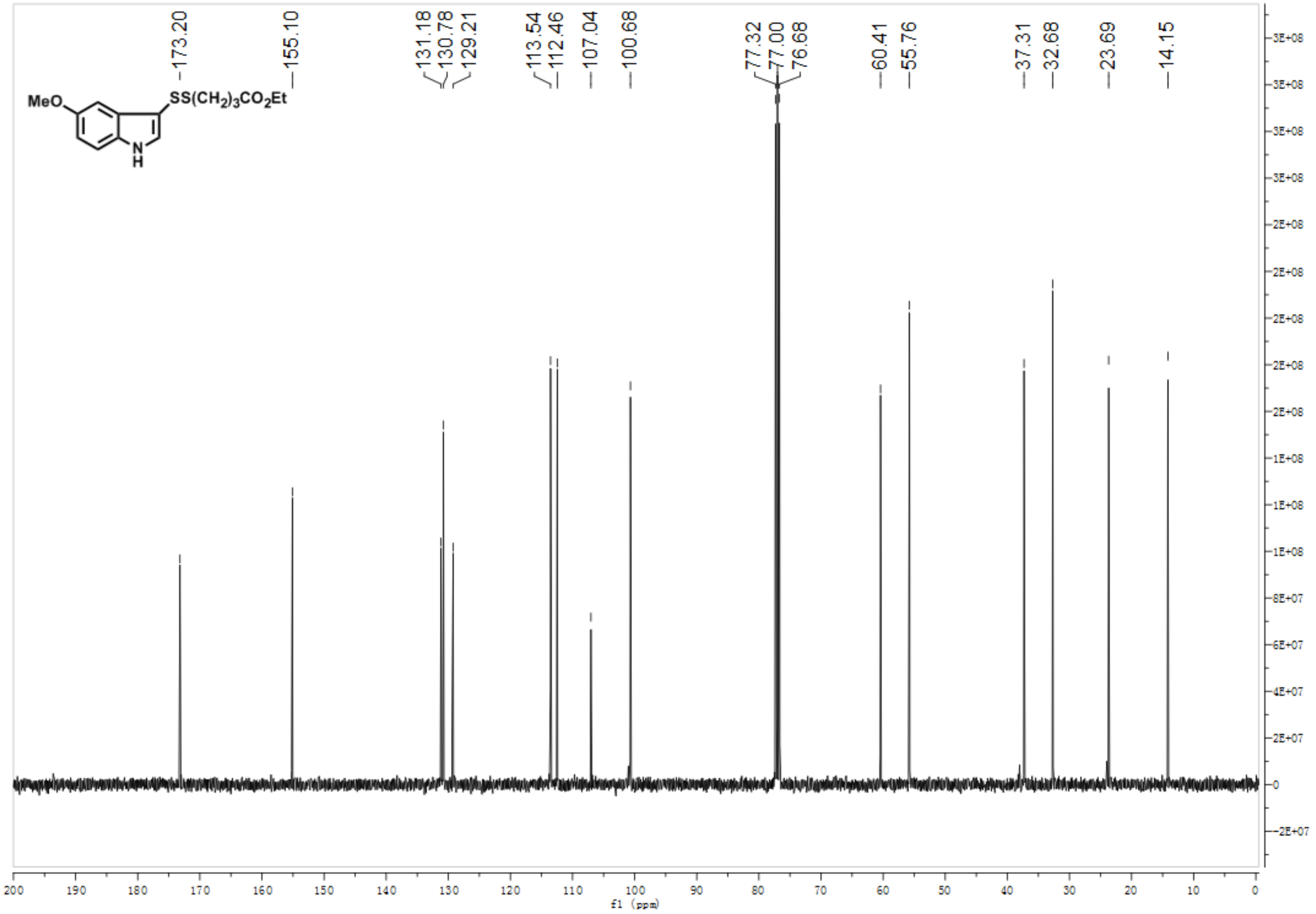

Supplementary Figure 88. ^{13}C NMR spectra for Compound **5f**.

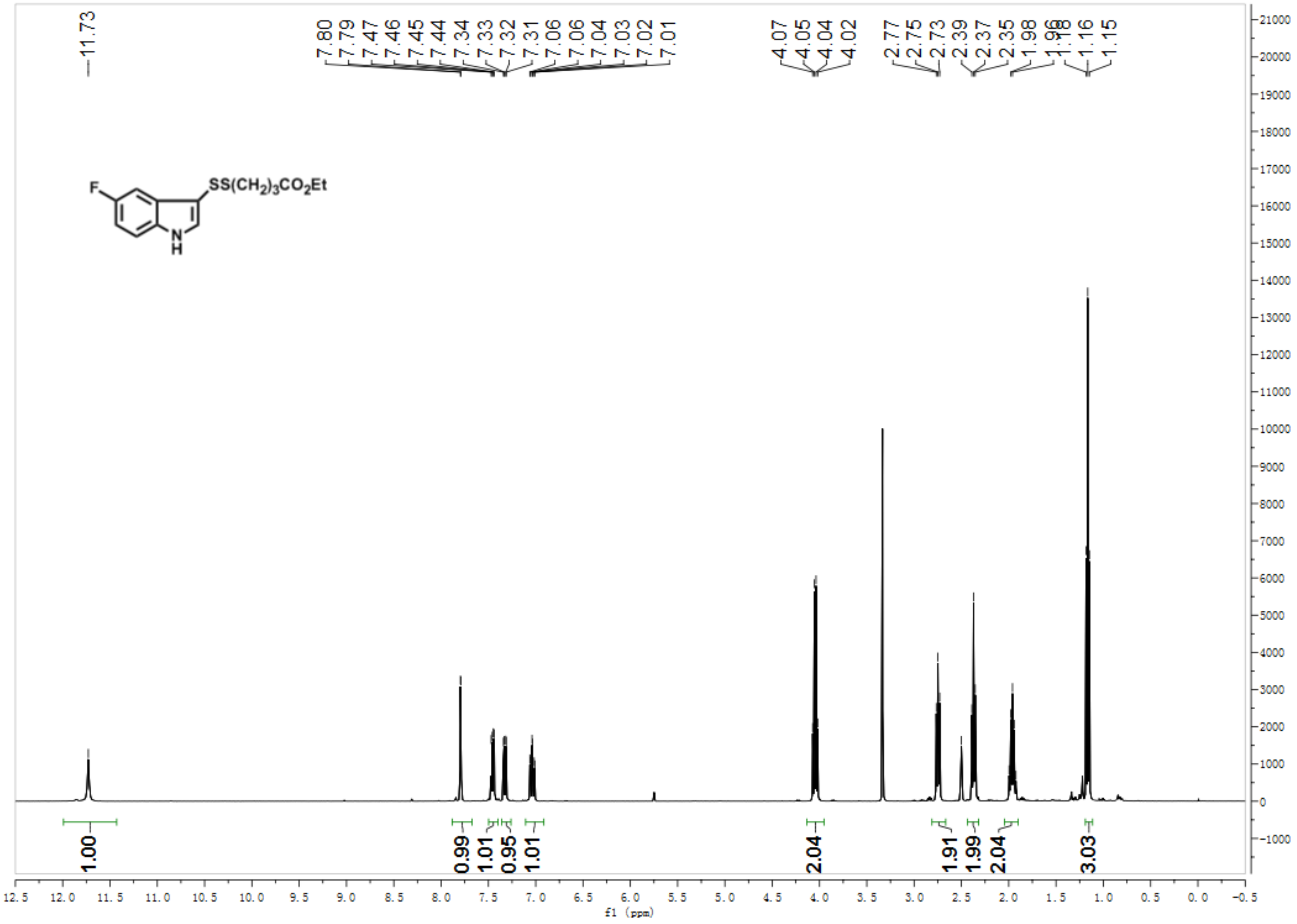

Supplementary Figure 89. ^{1}H NMR spectra for Compound **5g**.

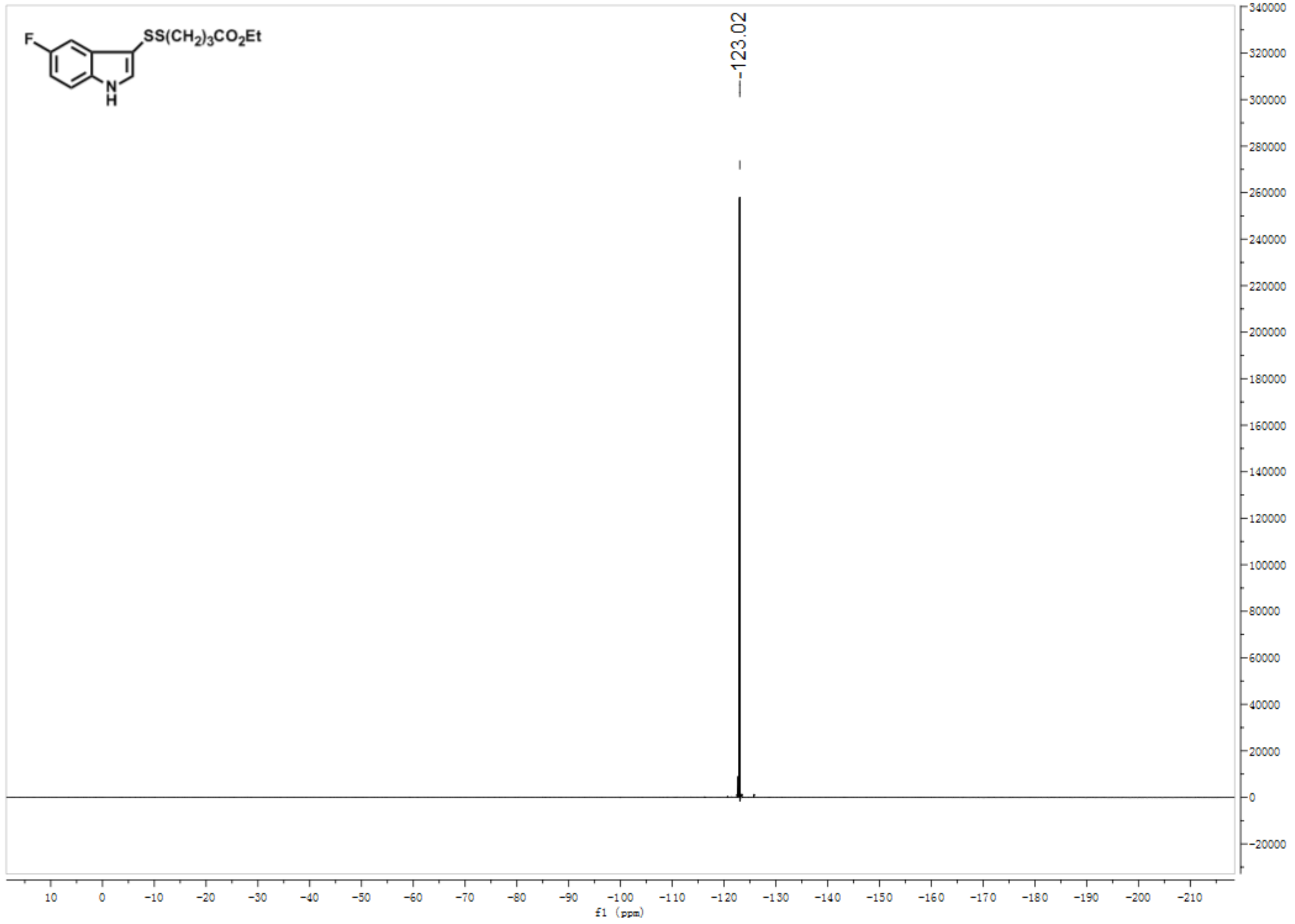

Supplementary Figure 90. ^{19}F NMR spectra for Compound **5g**.

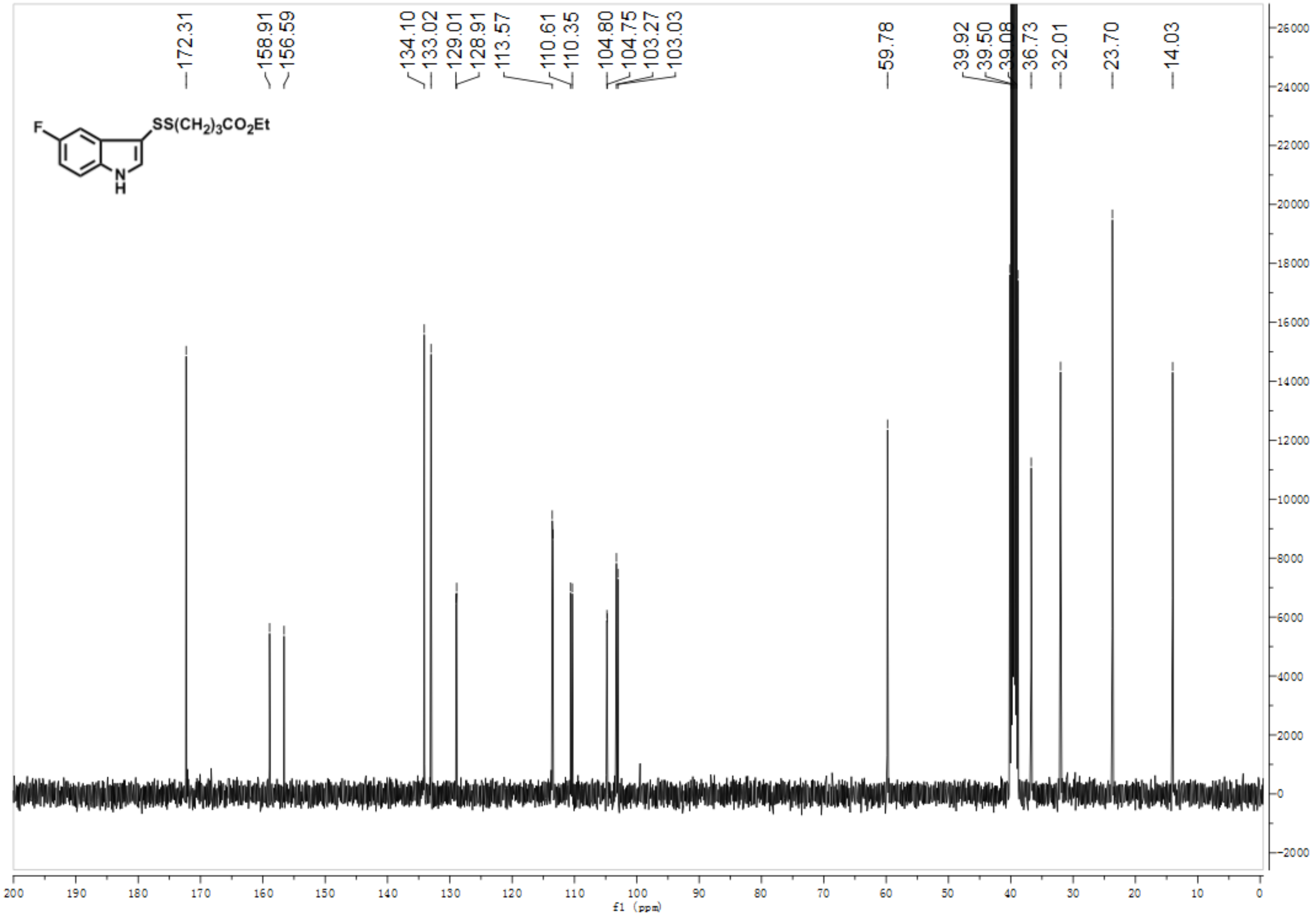

Supplementary Figure 91. ^{13}C NMR spectra for Compound **5g**.

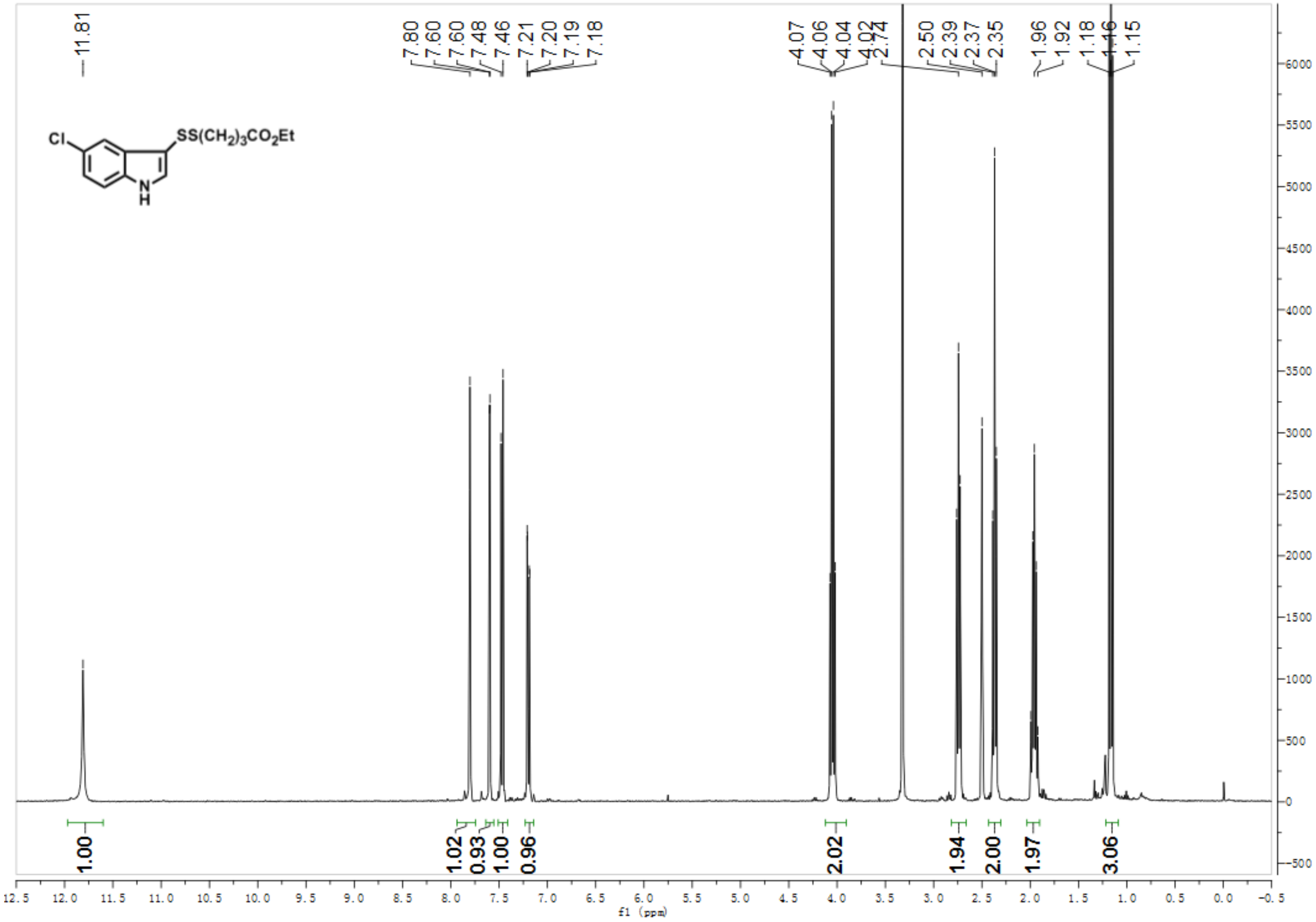

Supplementary Figure 92. ^{1}H NMR spectra for Compound **5h**.

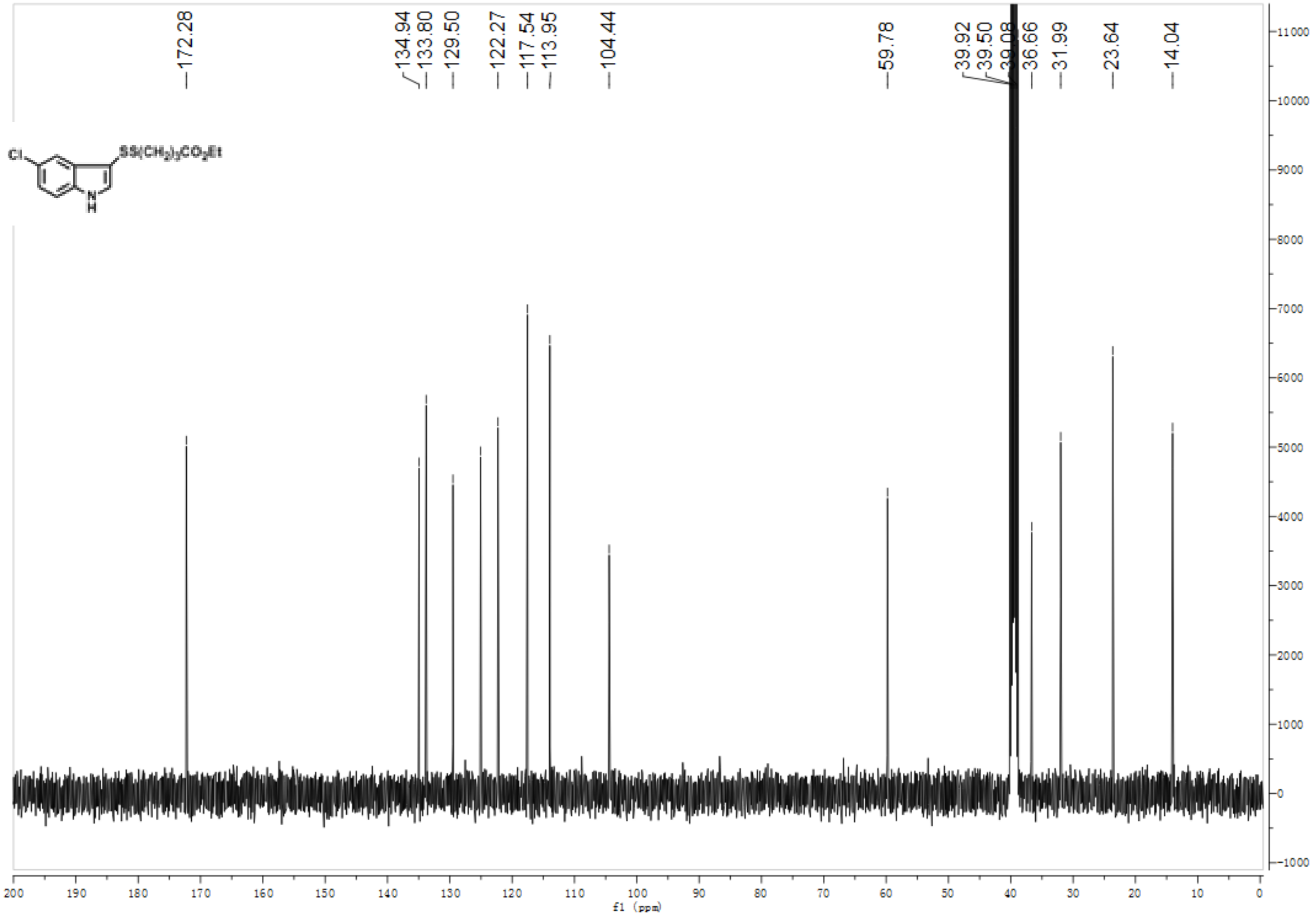

Supplementary Figure 93. ^{13}C NMR spectra for Compound **5h**.

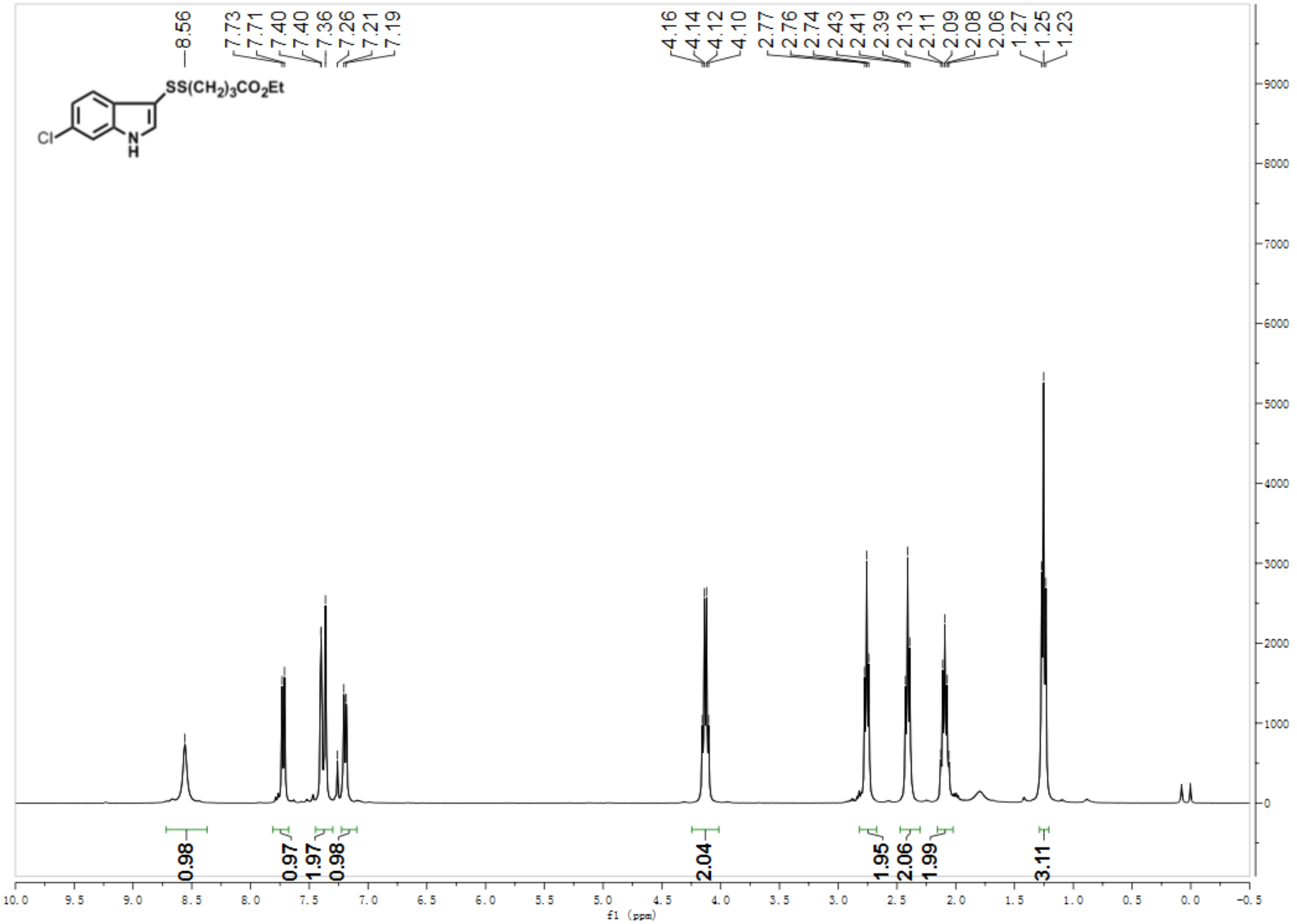

Supplementary Figure 94. ^{1}H NMR spectra for Compound **5i**.

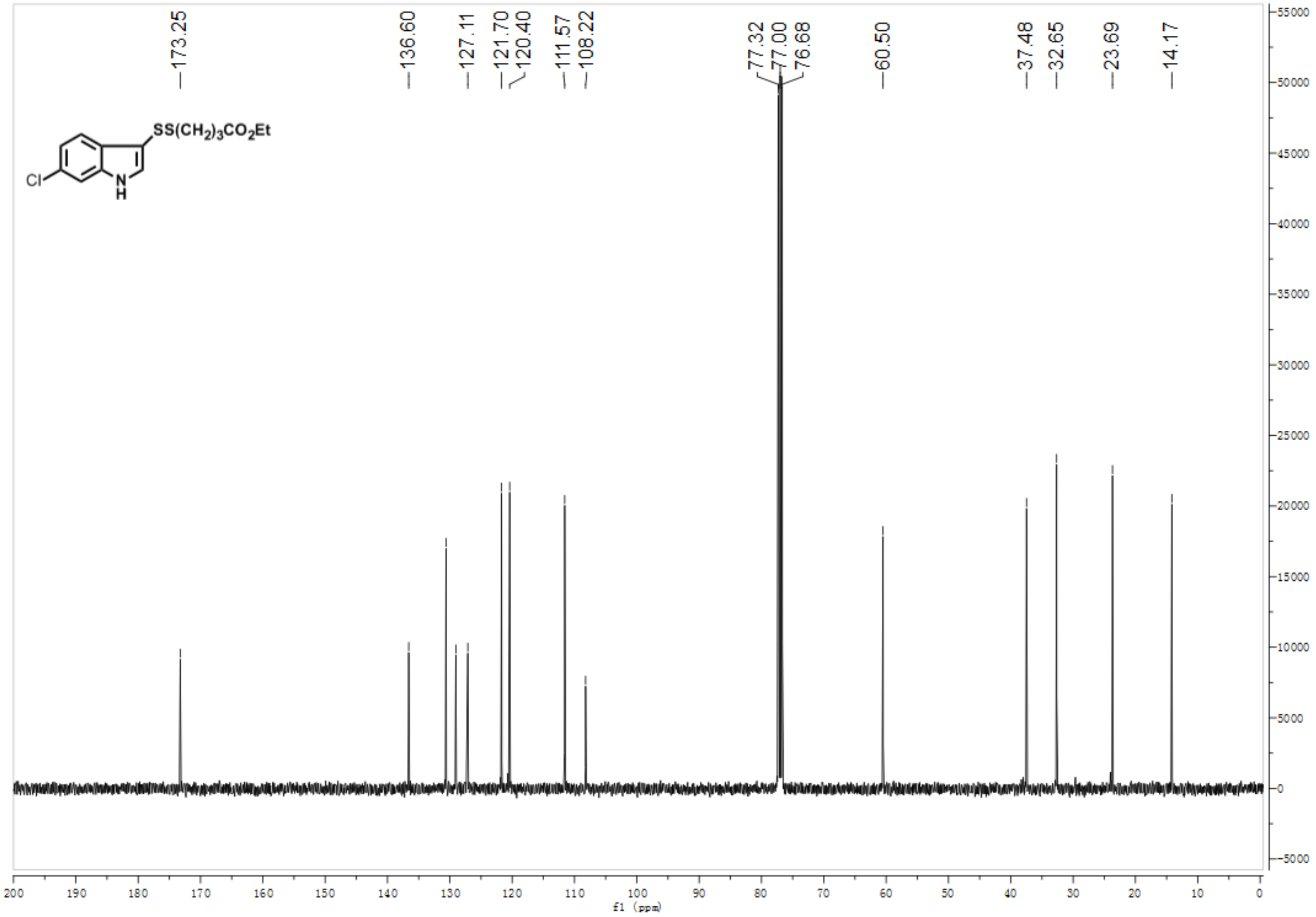

Supplementary Figure 95. ^{13}C NMR spectra for Compound **5i**.

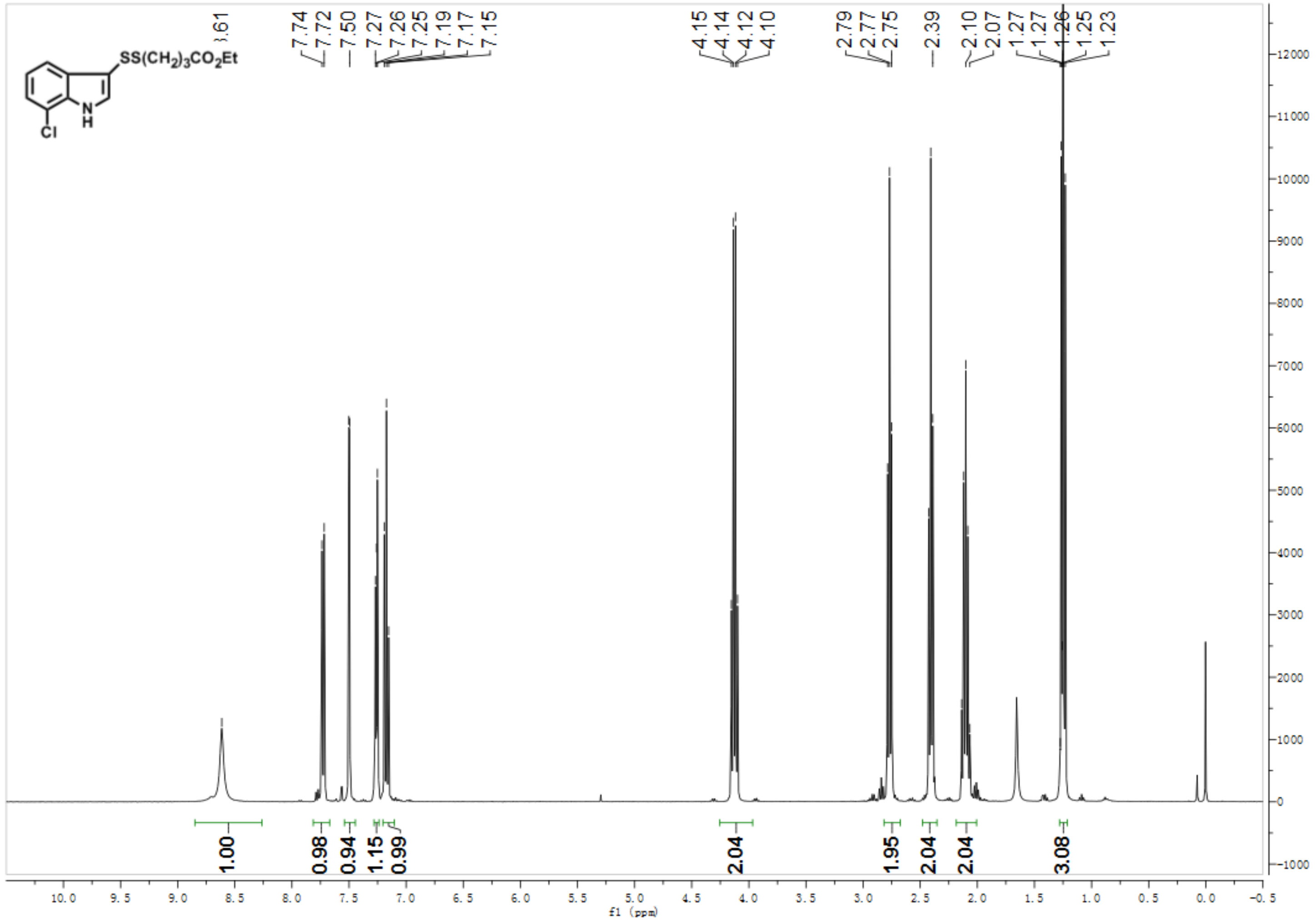

Supplementary Figure 96. ^{1}H NMR spectra for Compound **5j**.

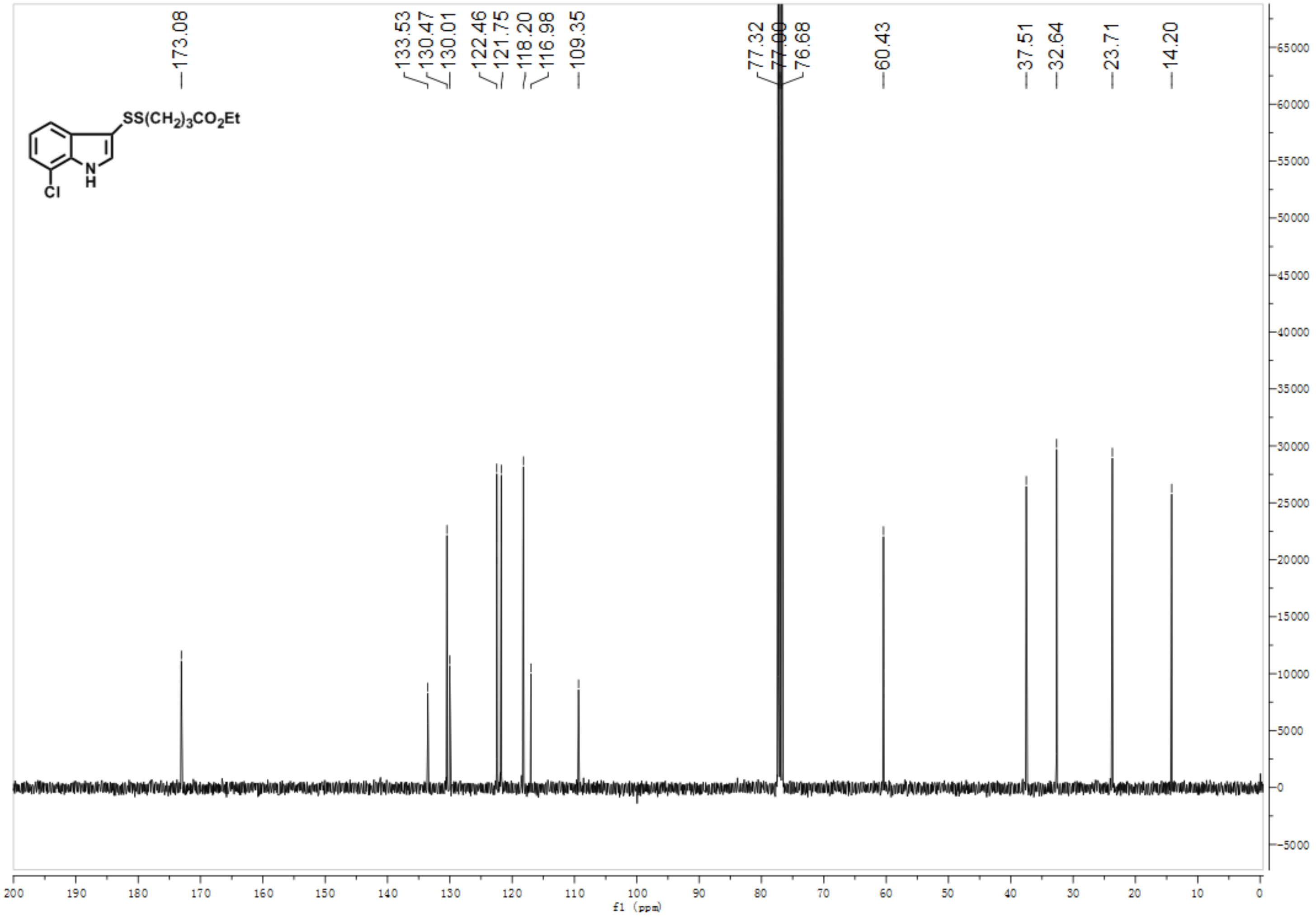

Supplementary Figure 97. ^{13}C NMR spectra for Compound **5j**.

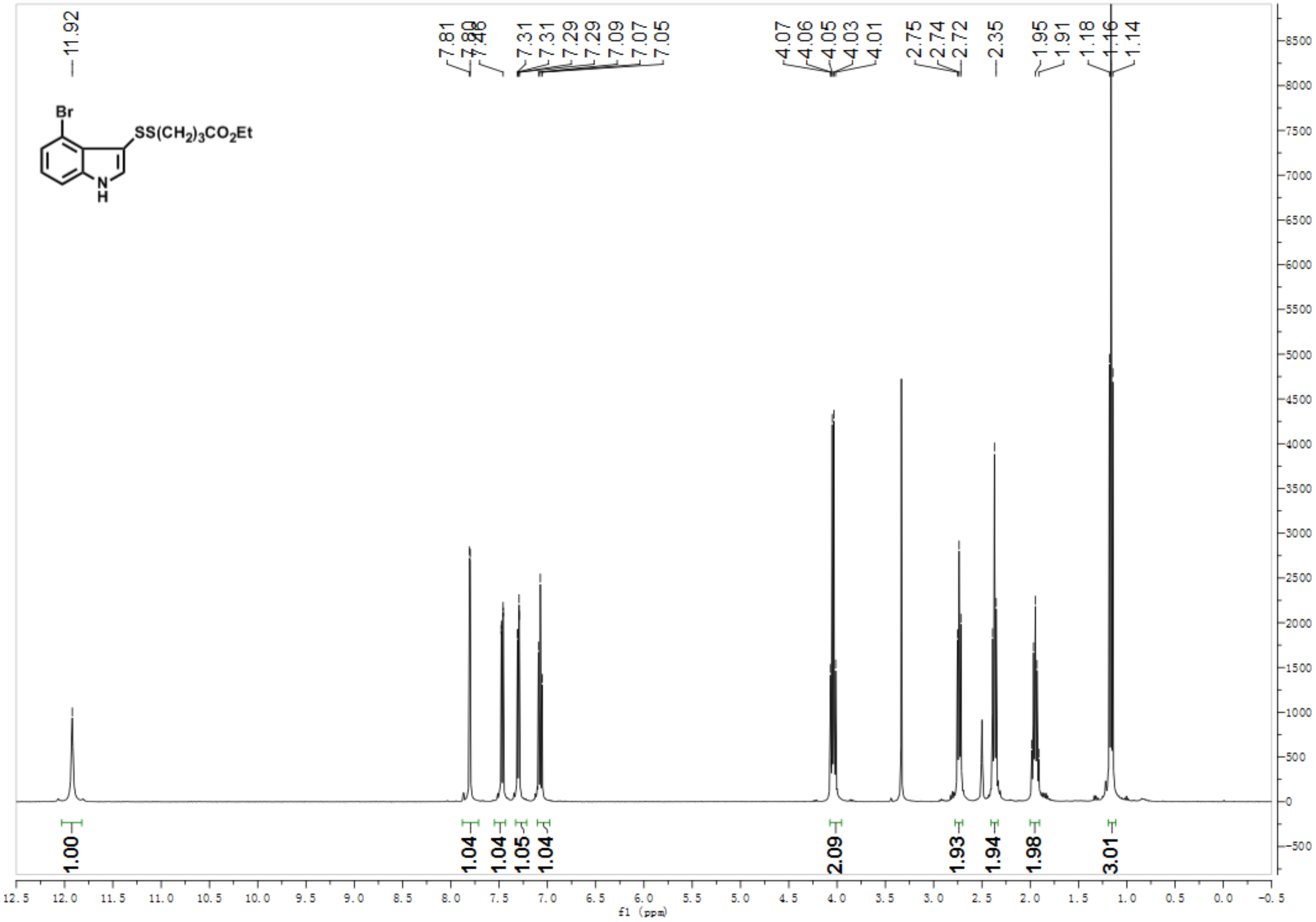

Supplementary Figure 98. ^{1}H NMR spectra for Compound **5k**.

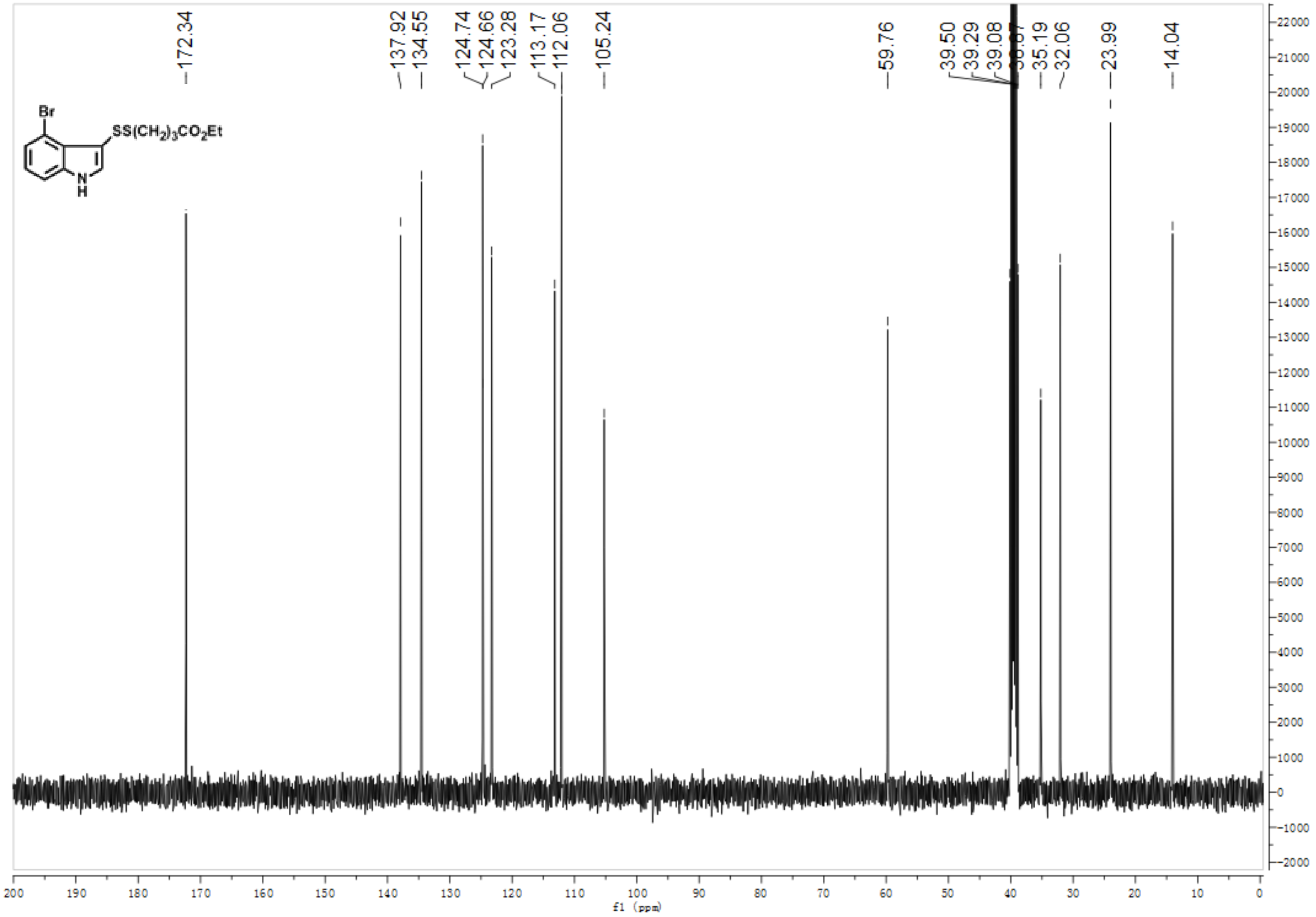

Supplementary Figure 99. ^{13}C NMR spectra for Compound **5k**.

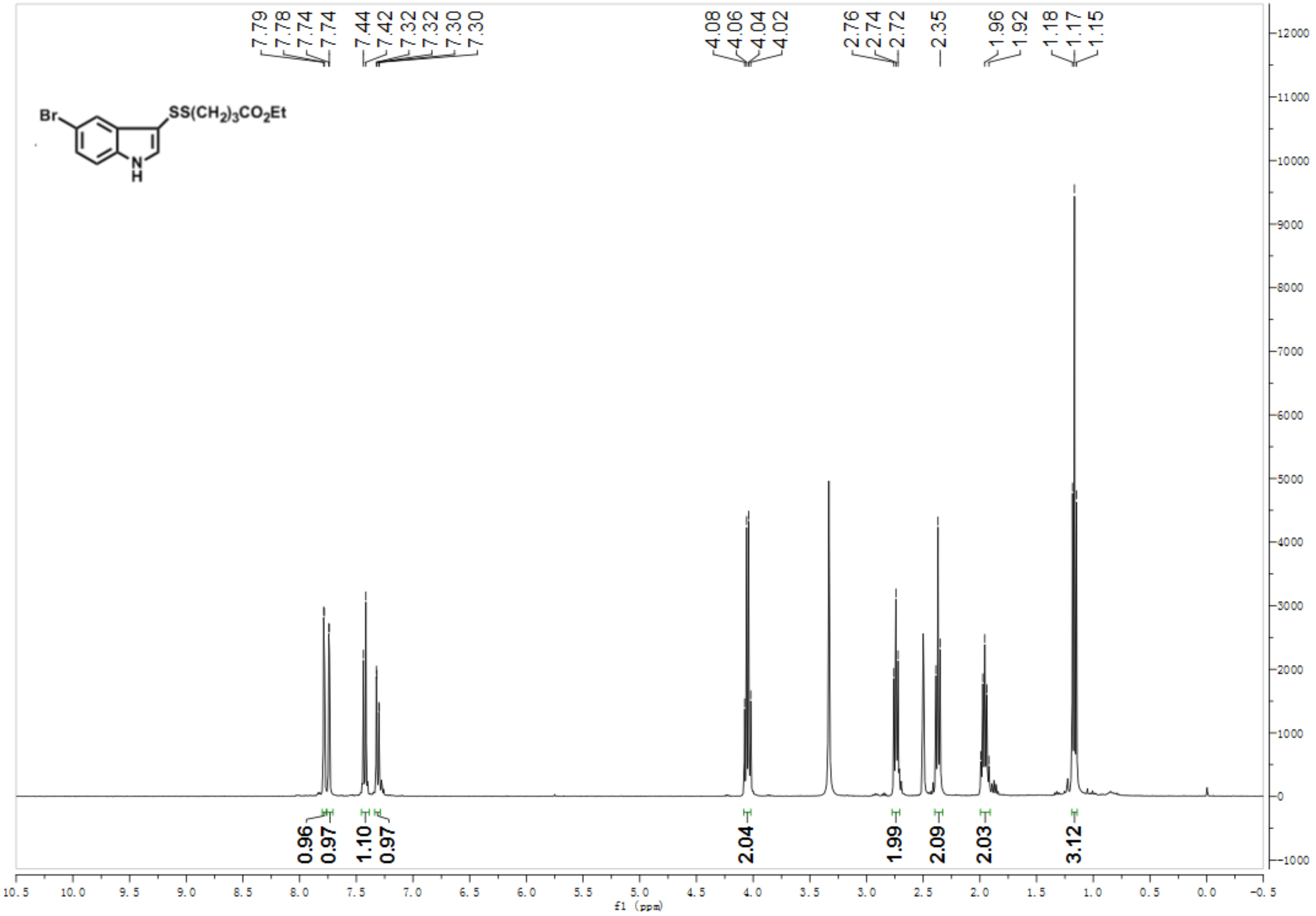

Supplementary Figure 100. ^{1}H NMR spectra for Compound **5l**.

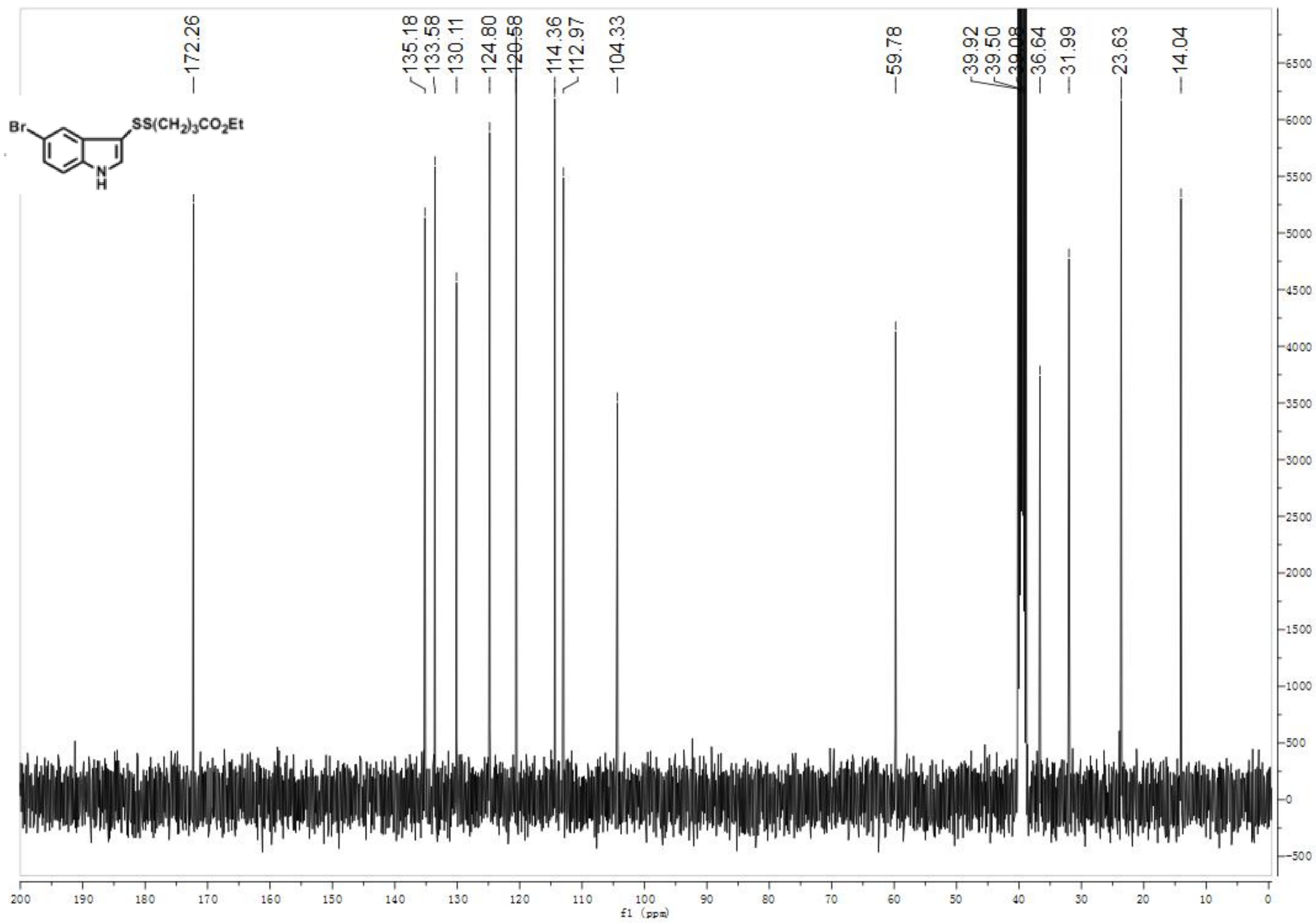

Supplementary Figure 101. ^{13}C NMR spectra for Compound **5l**.

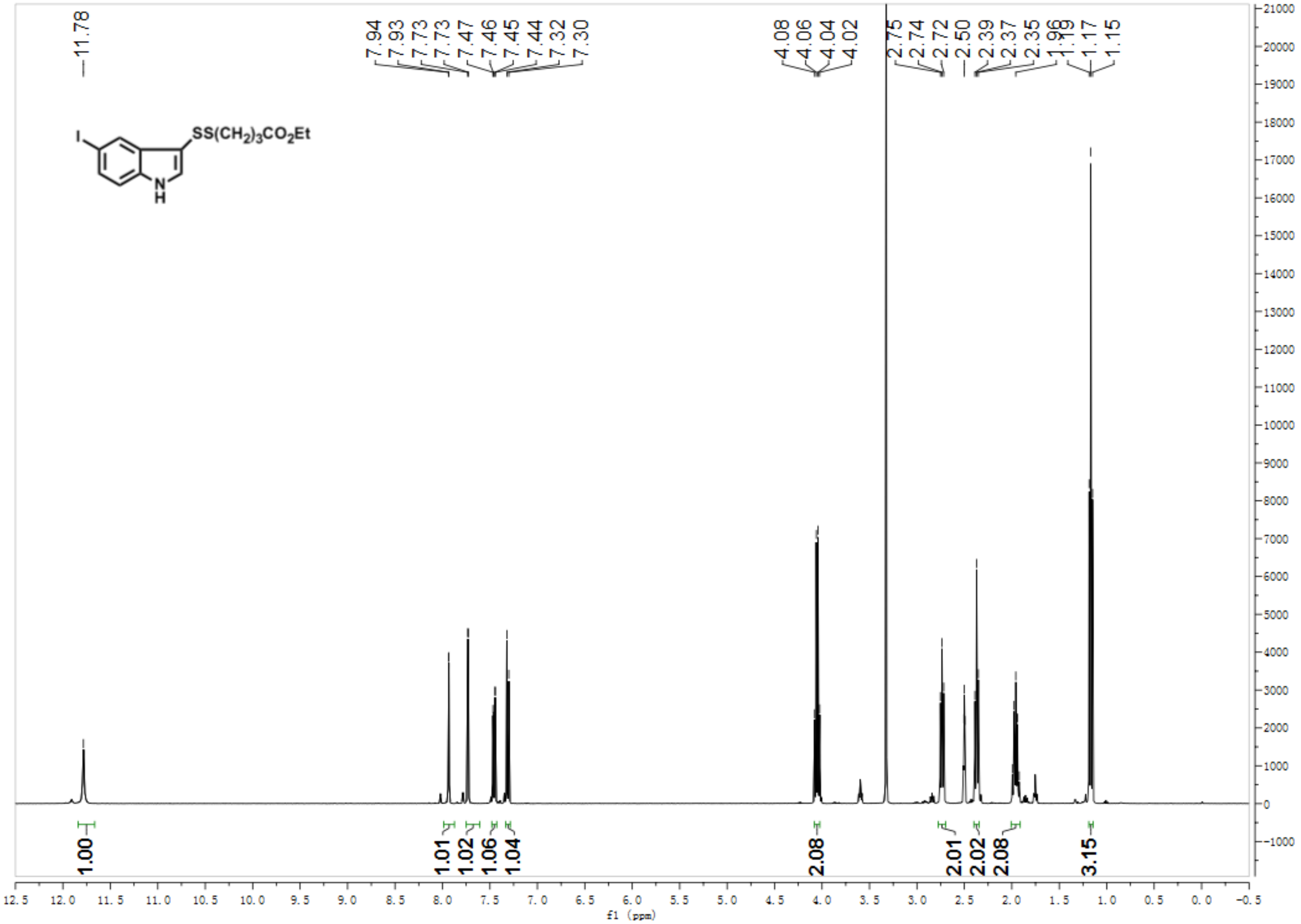

Supplementary Figure 102. ^{1}H NMR spectra for Compound **5m**.

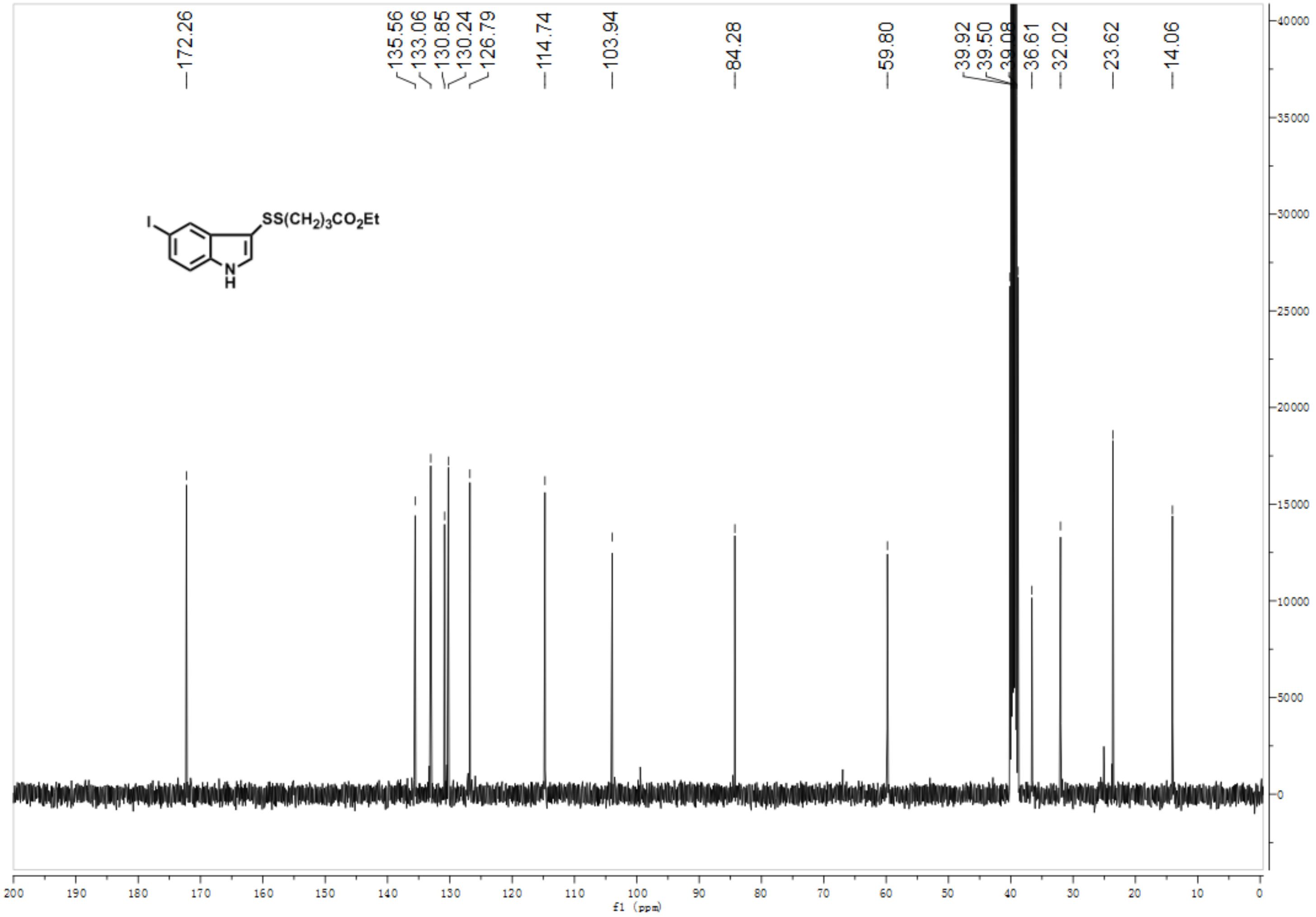

Supplementary Figure 103. ^{13}C NMR spectra for Compound **5m**.

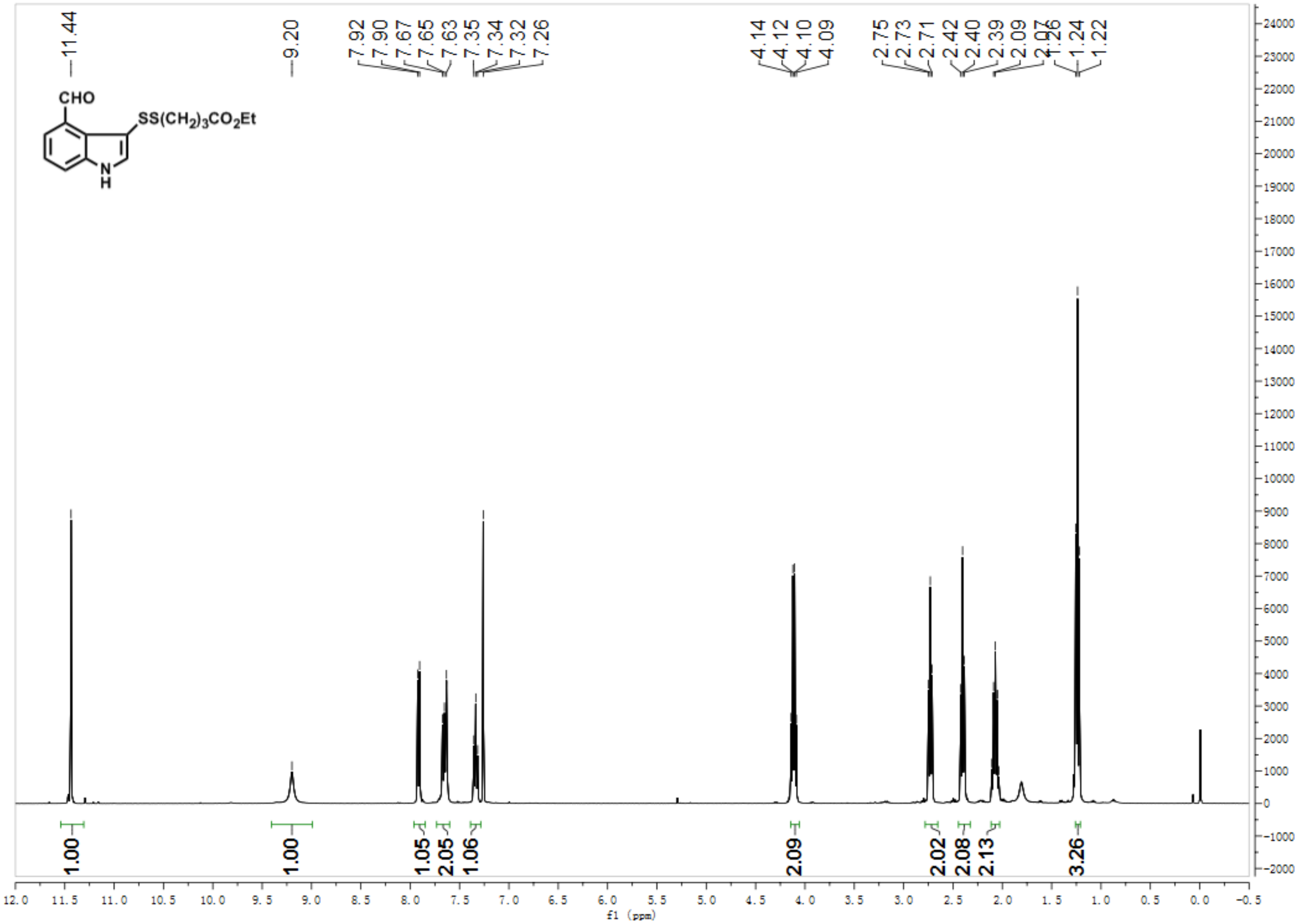

Supplementary Figure 104. ^{1}H NMR spectra for Compound **5n**.

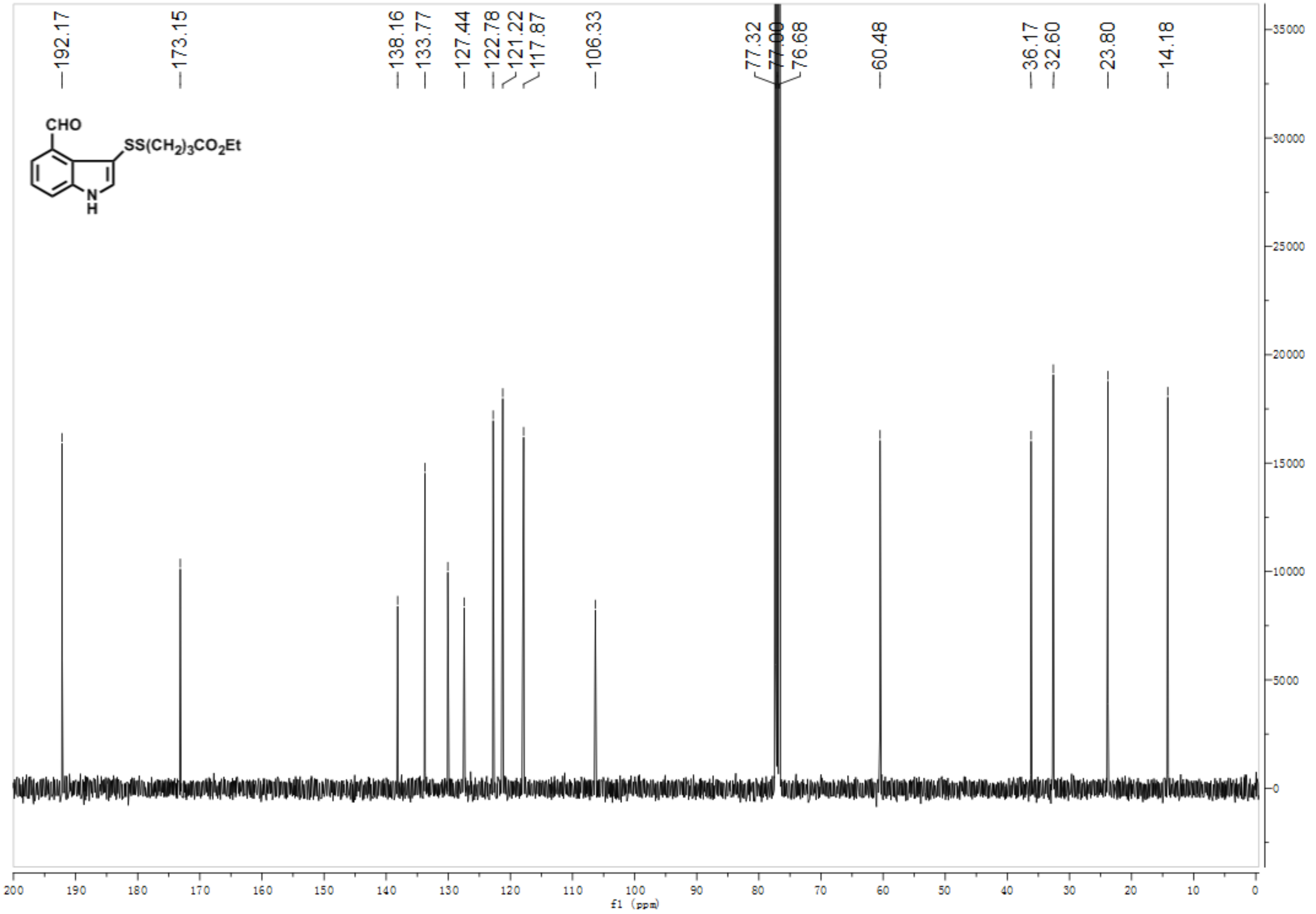

Supplementary Figure 105. ^{13}C NMR spectra for Compound **5n**.

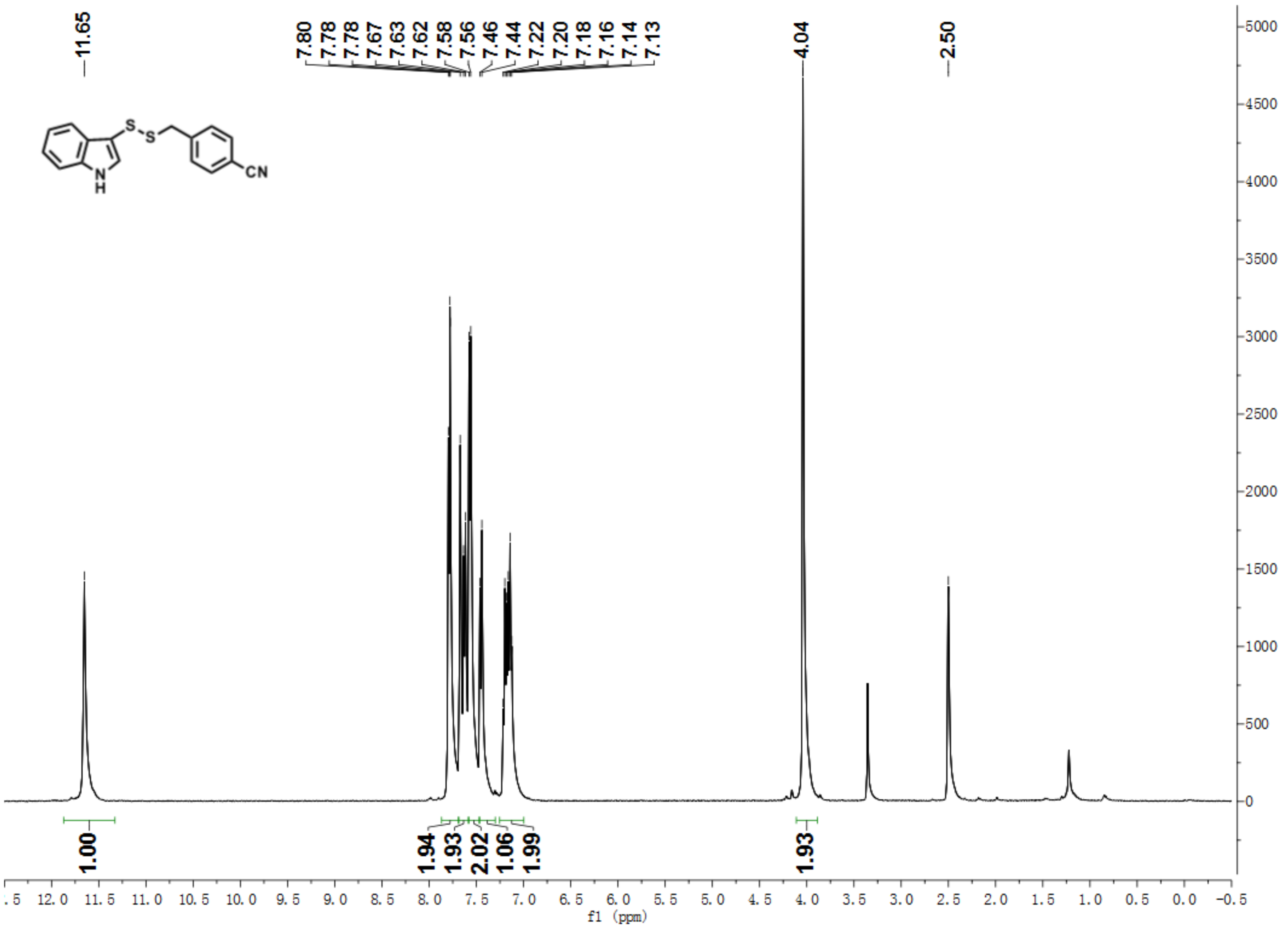

Supplementary Figure 106. ^{1}H NMR spectra for Compound **5o**.

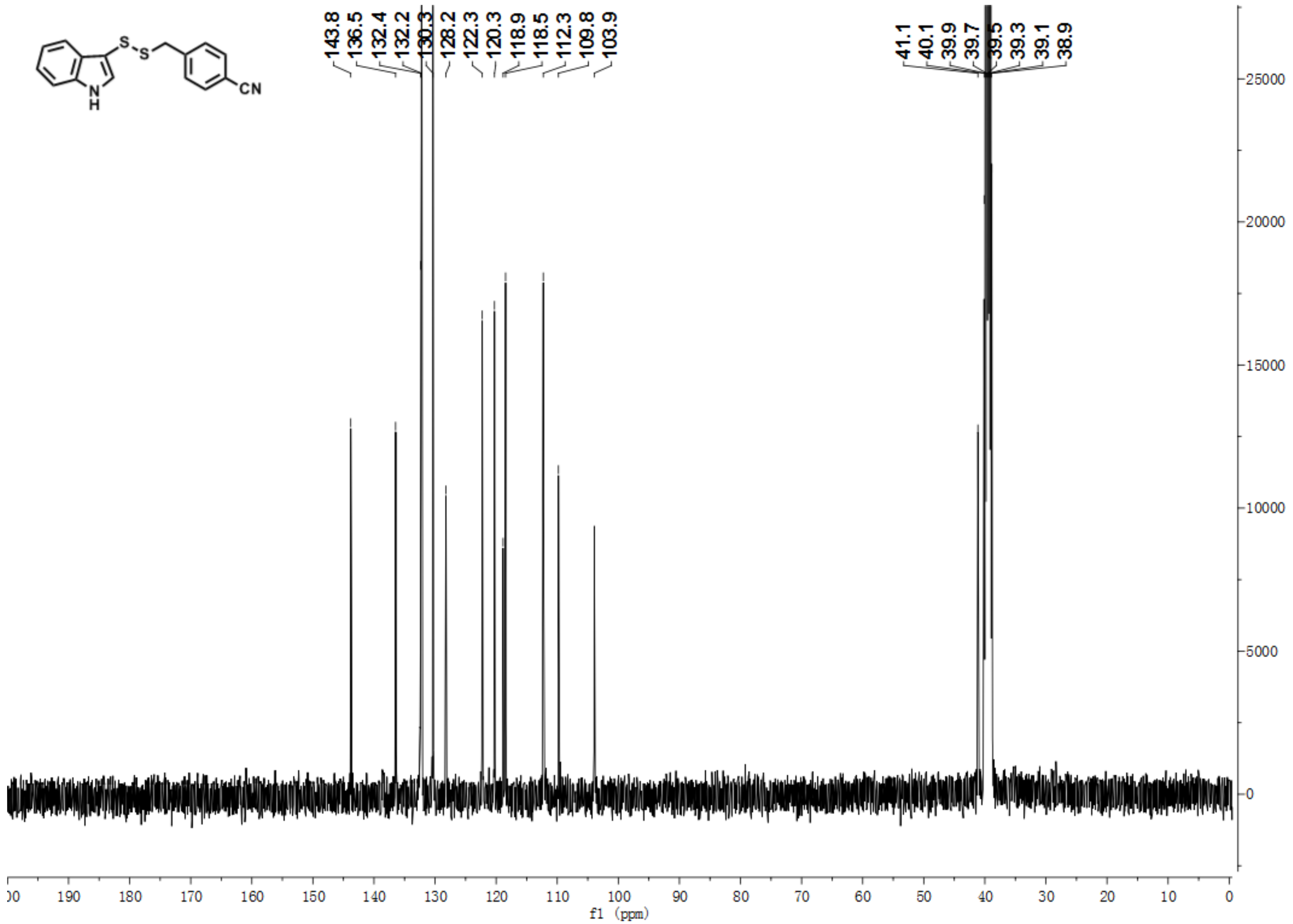

Supplementary Figure 107. ^{13}C NMR spectra for Compound **5o**.

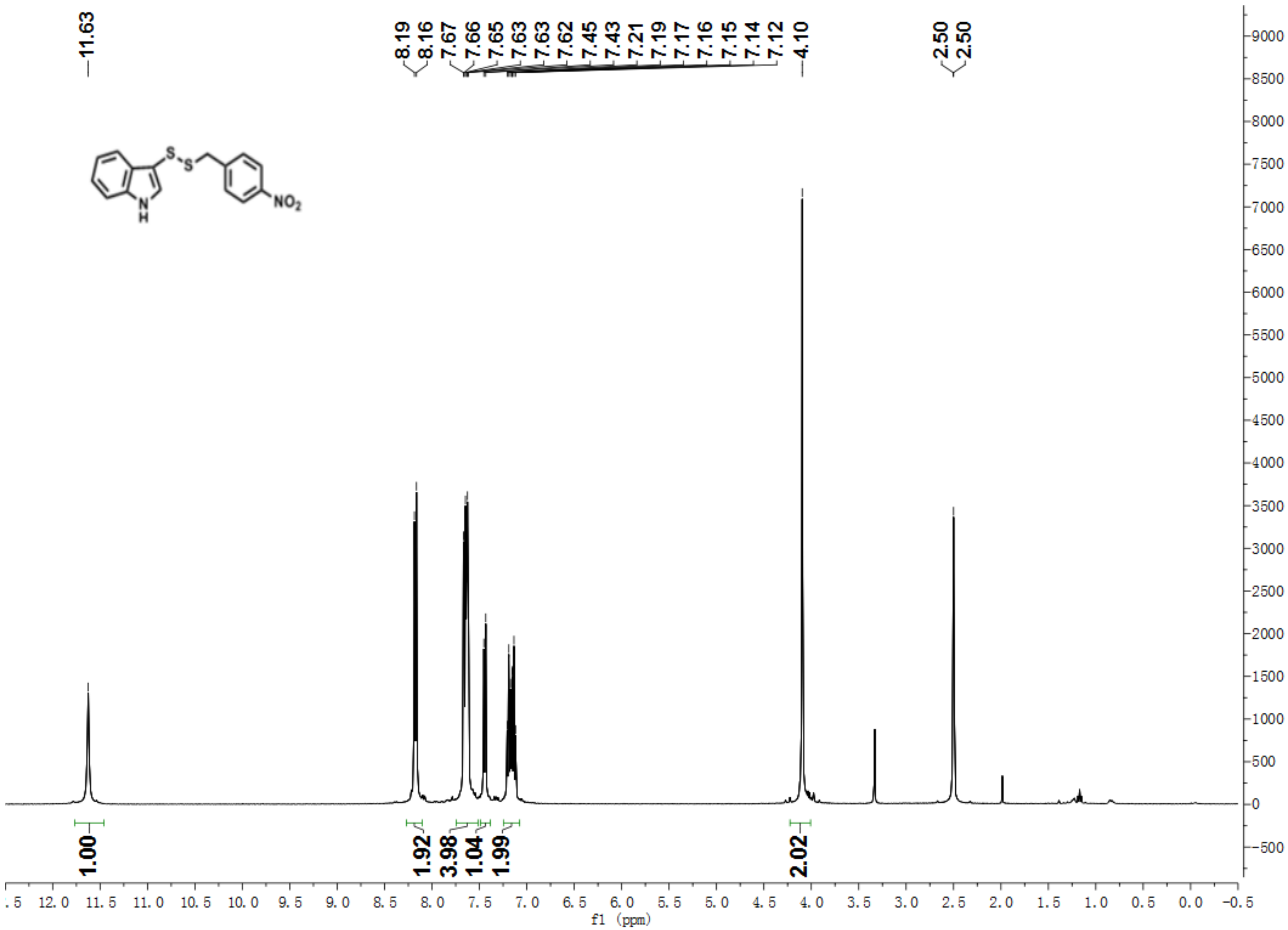

Supplementary Figure 108. ^{1}H NMR spectra for Compound **5p**.

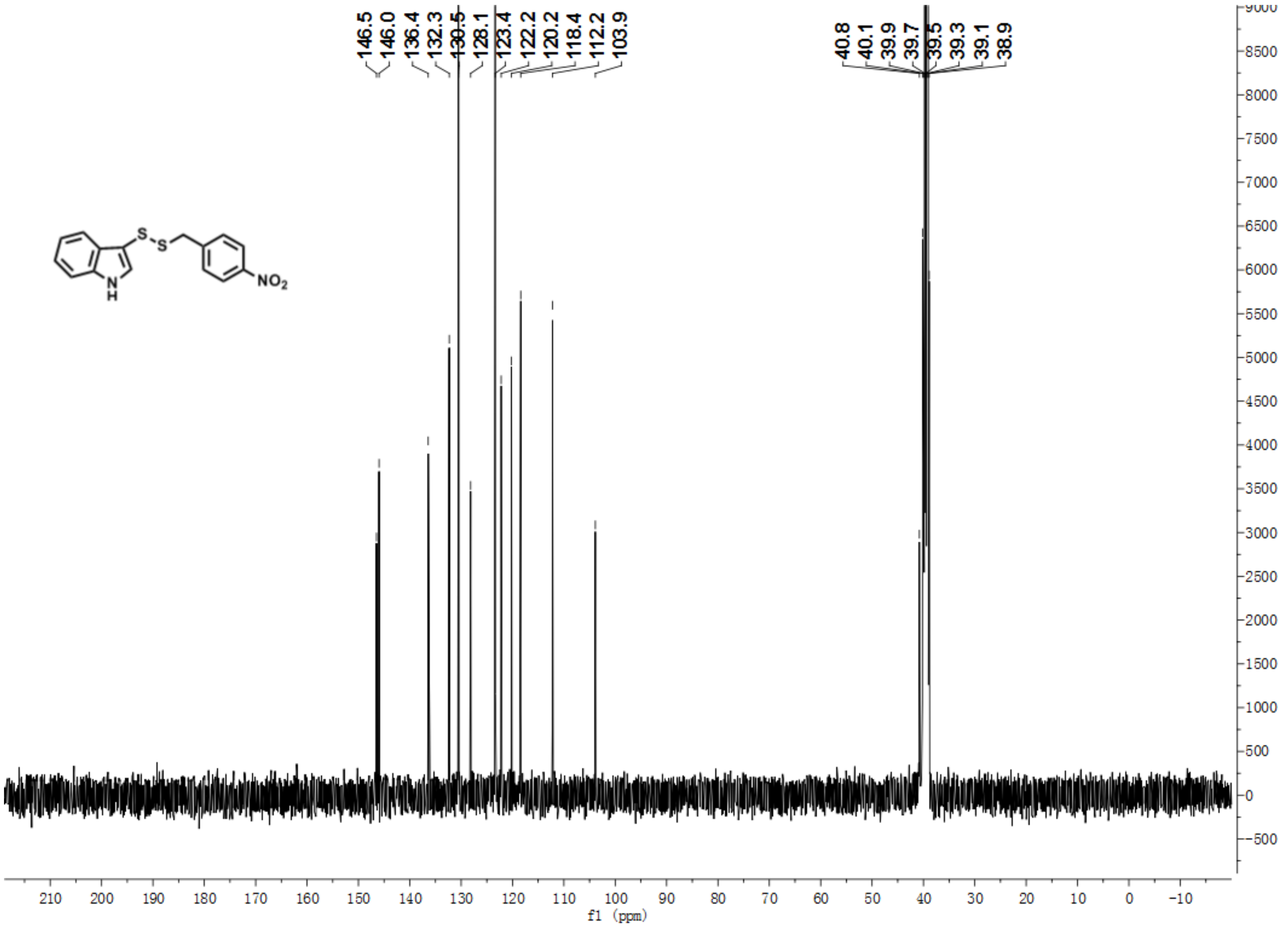

Supplementary Figure 109. ^{13}C NMR spectra for Compound **5p**.

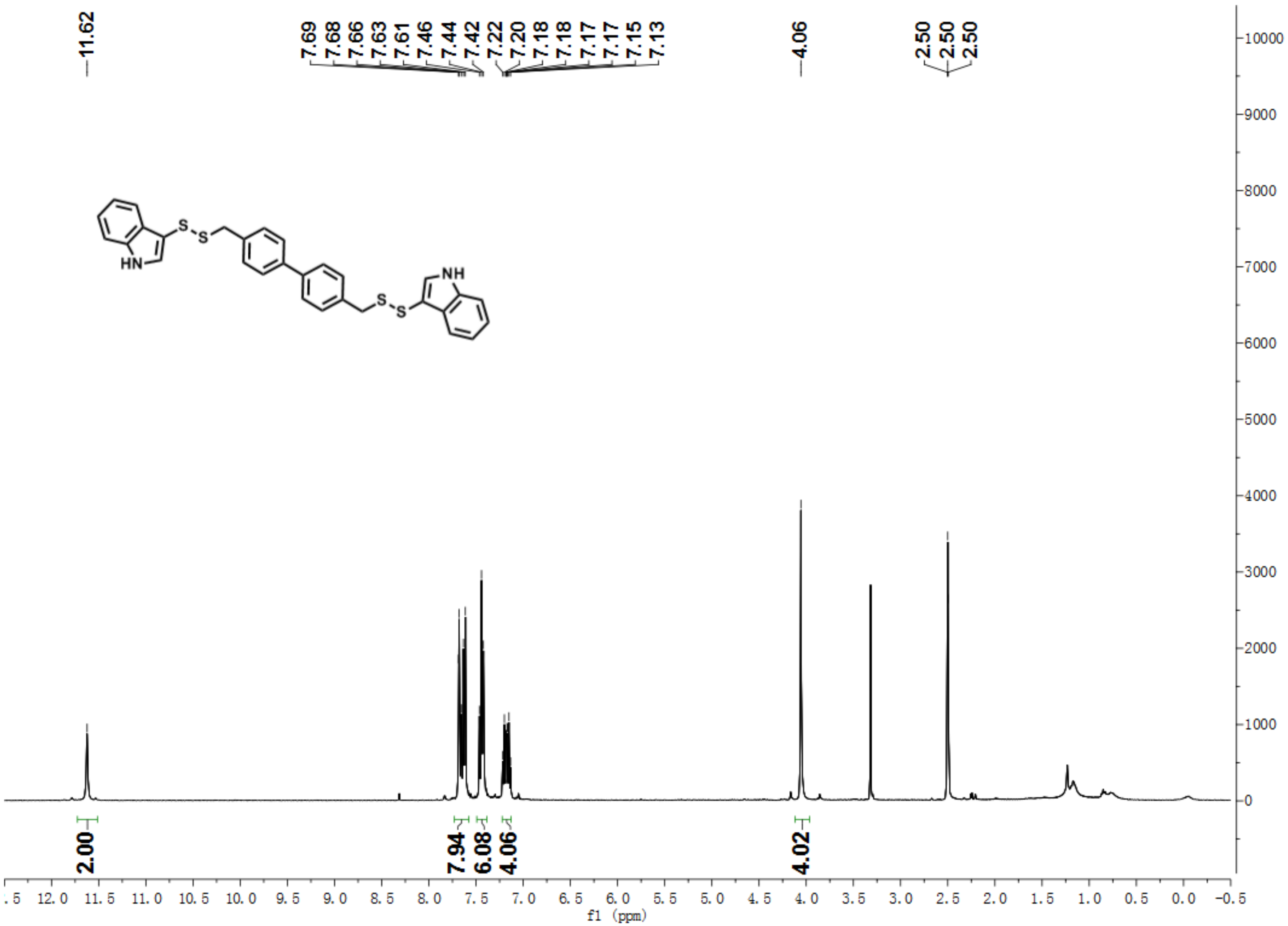

Supplementary Figure 110. ^{1}H NMR spectra for Compound **5q**.

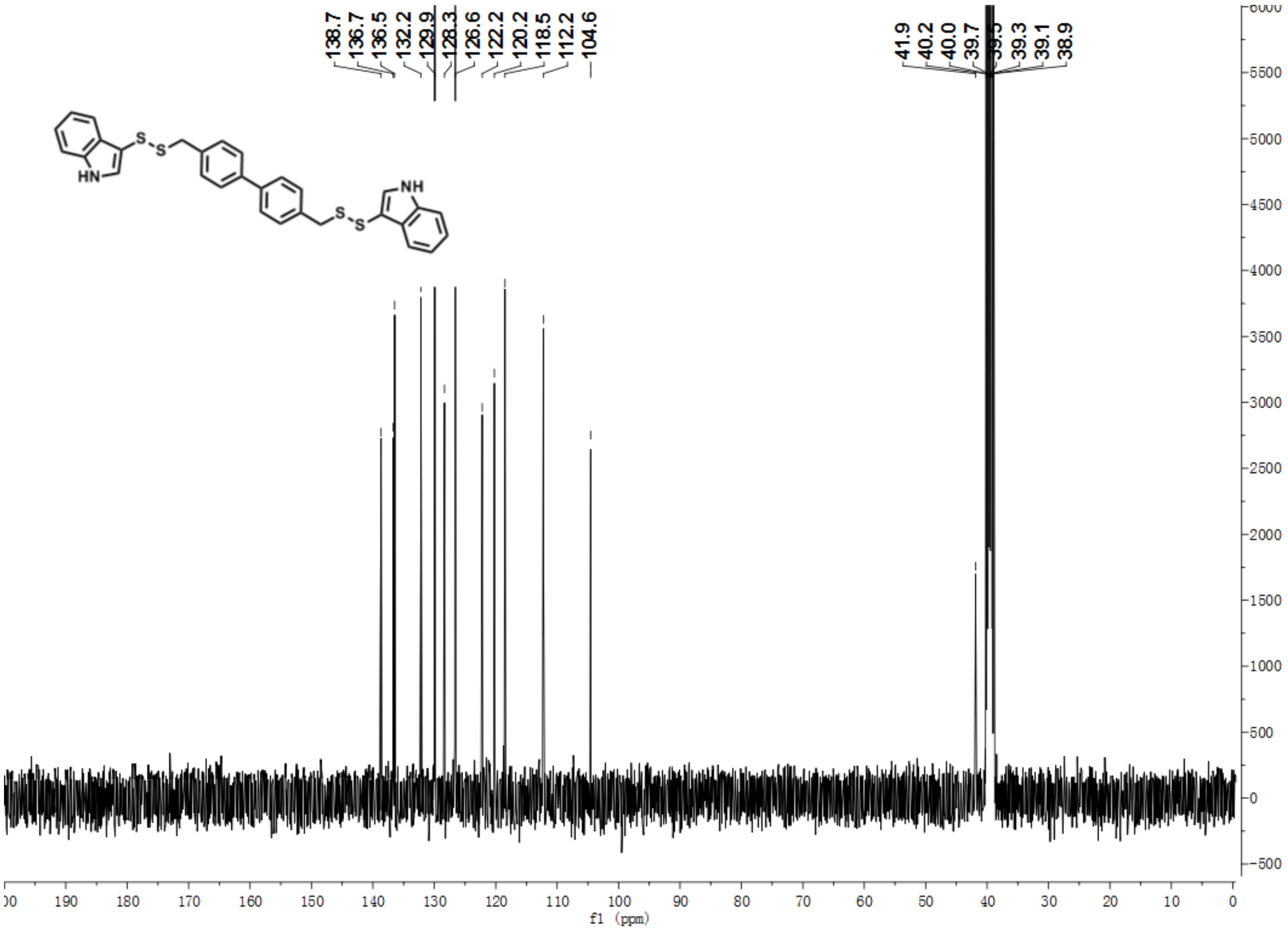

Supplementary Figure 111. ^{13}C NMR spectra for Compound **5q**.

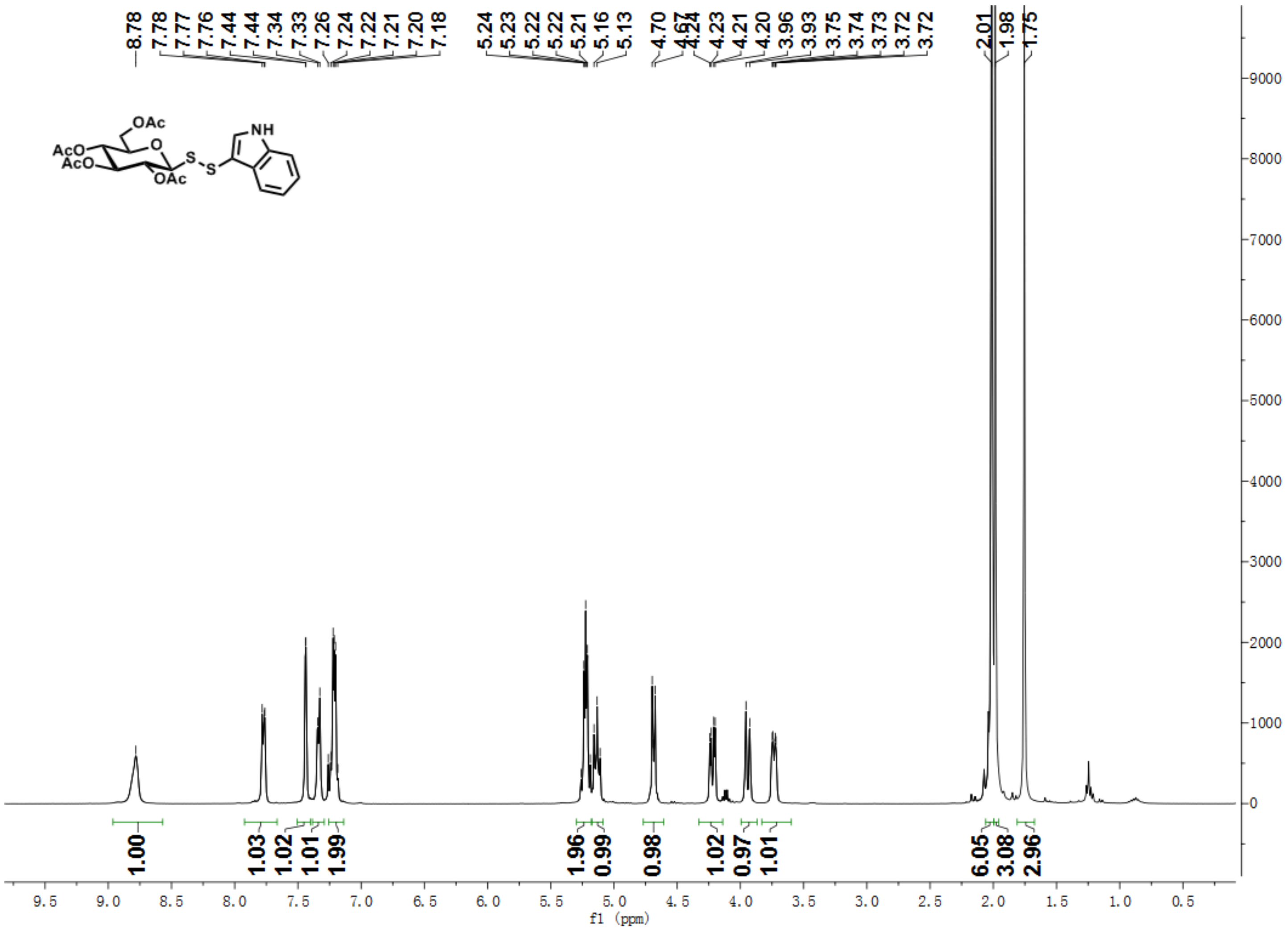

Supplementary Figure 112. ^{1}H NMR spectra for Compound **5r**.

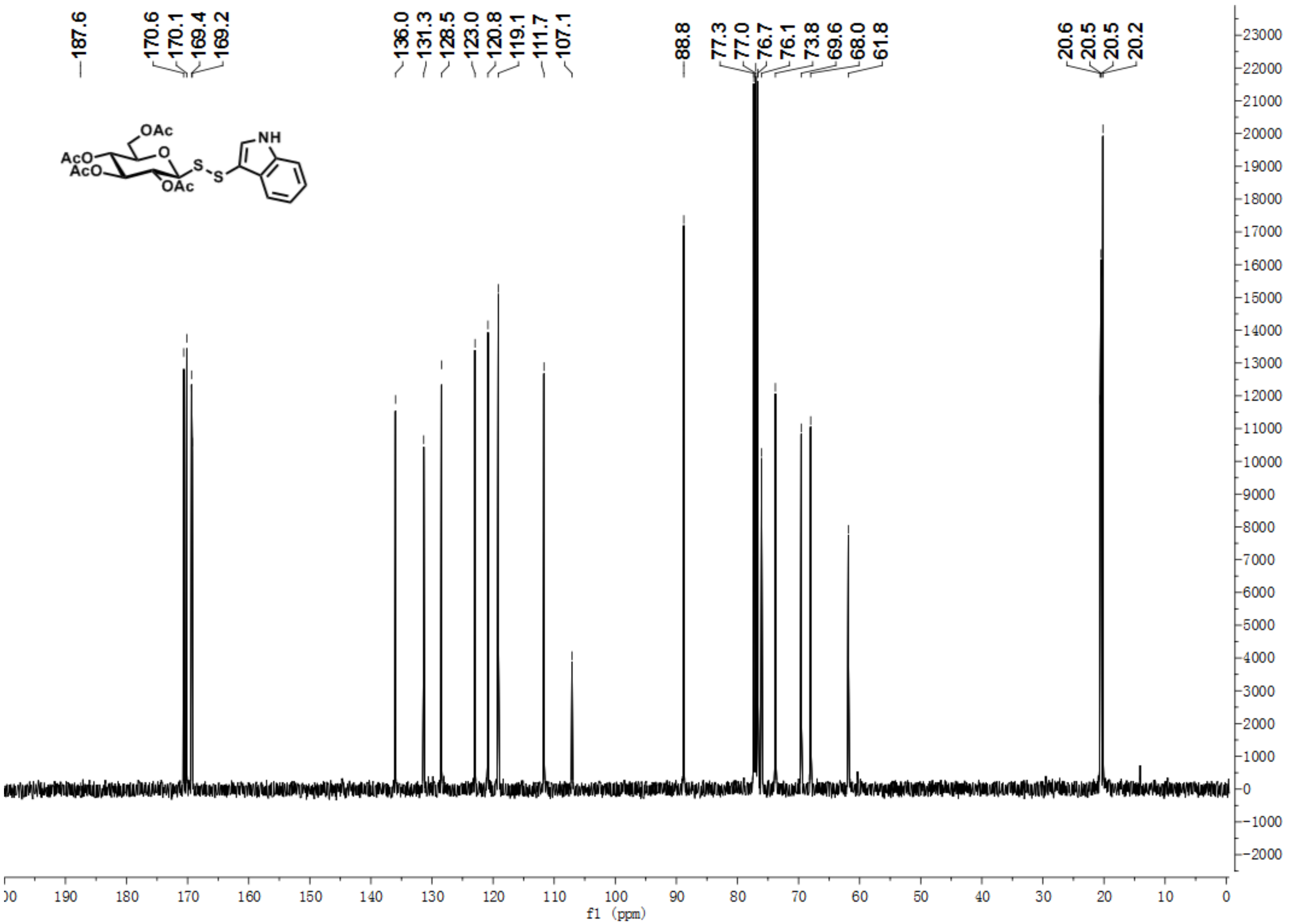

Supplementary Figure 113. ^{13}C NMR spectra for Compound **5r**.

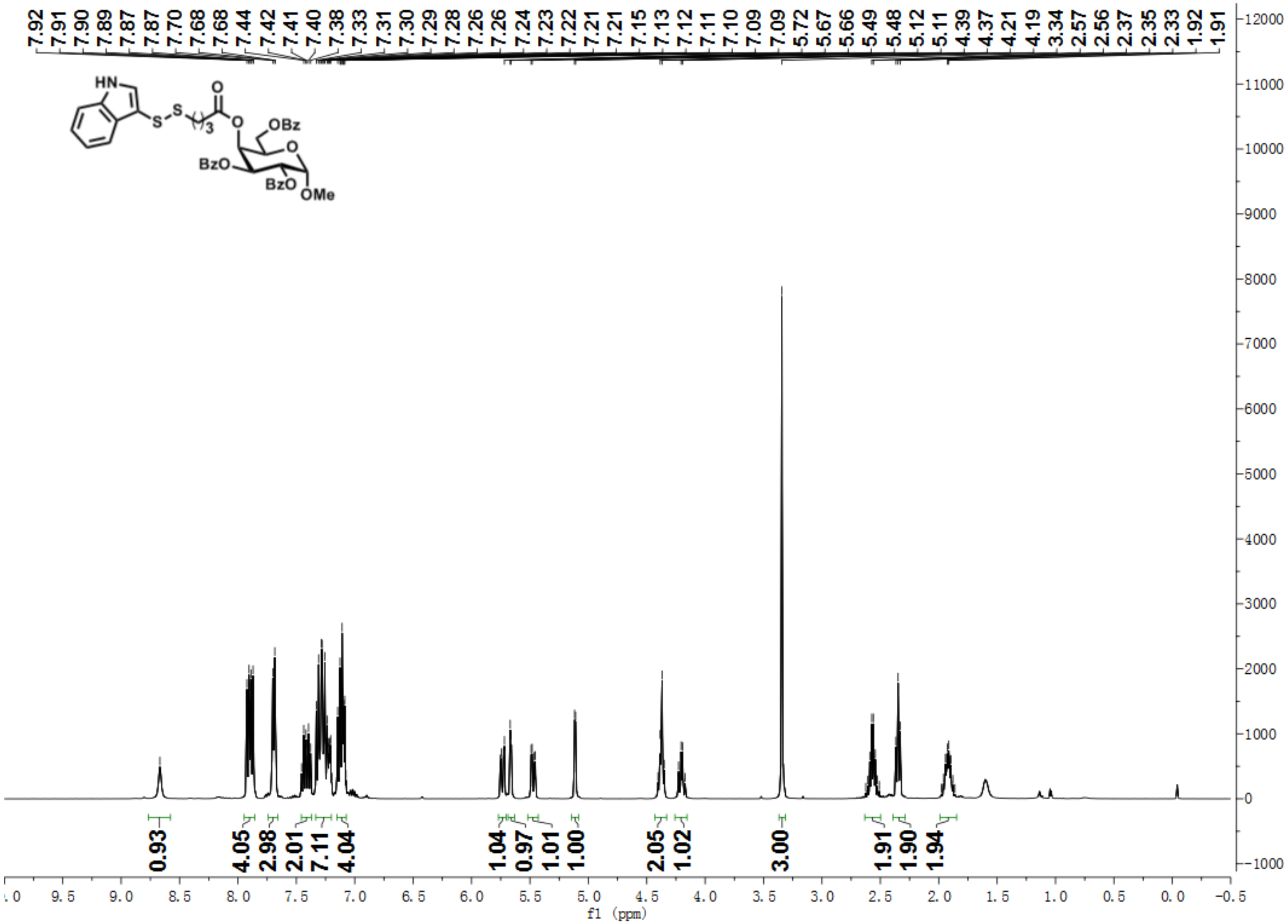

Supplementary Figure 114. ^{1}H NMR spectra for Compound **5s**.

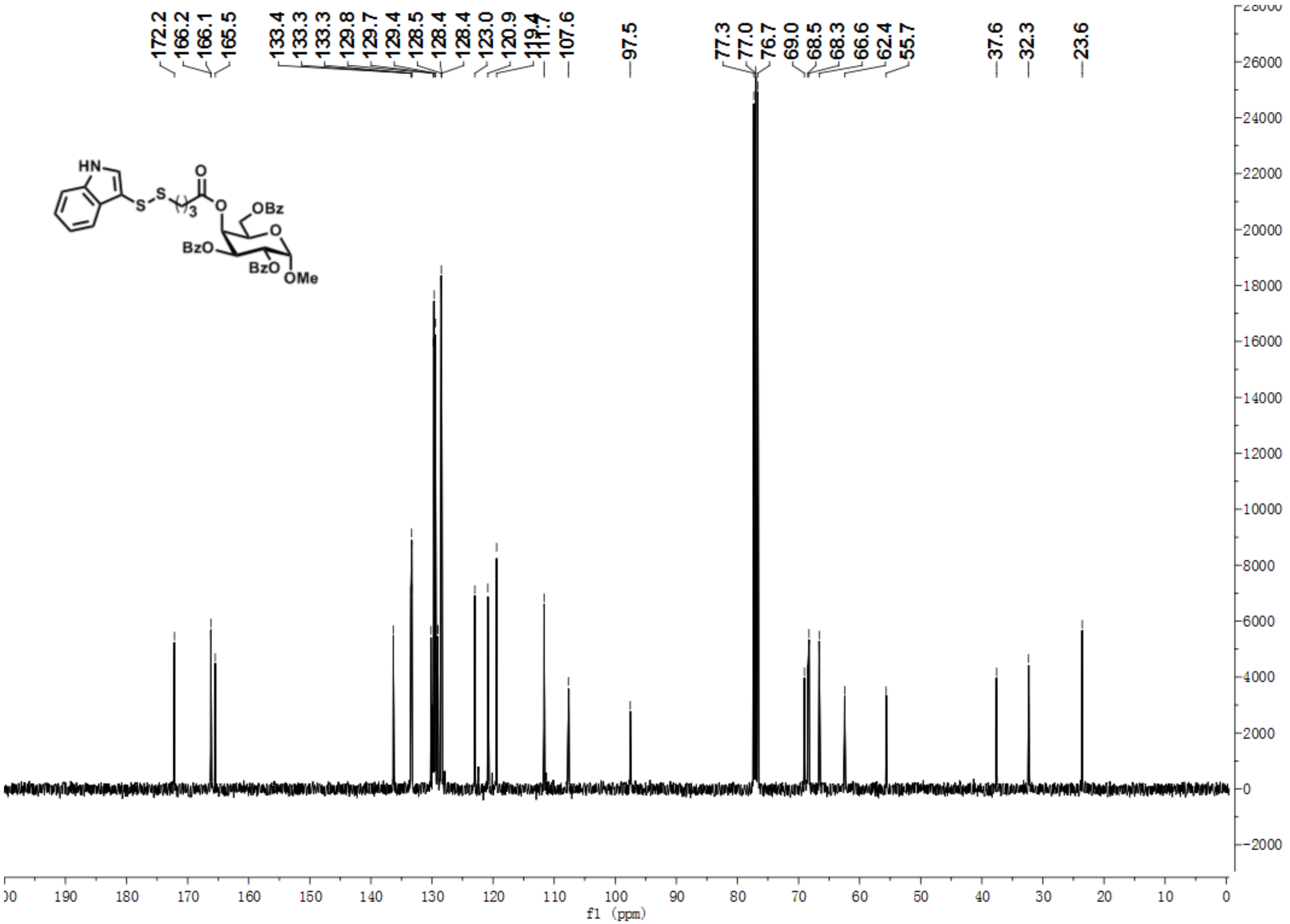

Supplementary Figure 115. ^{13}C NMR spectra for Compound **5s**.

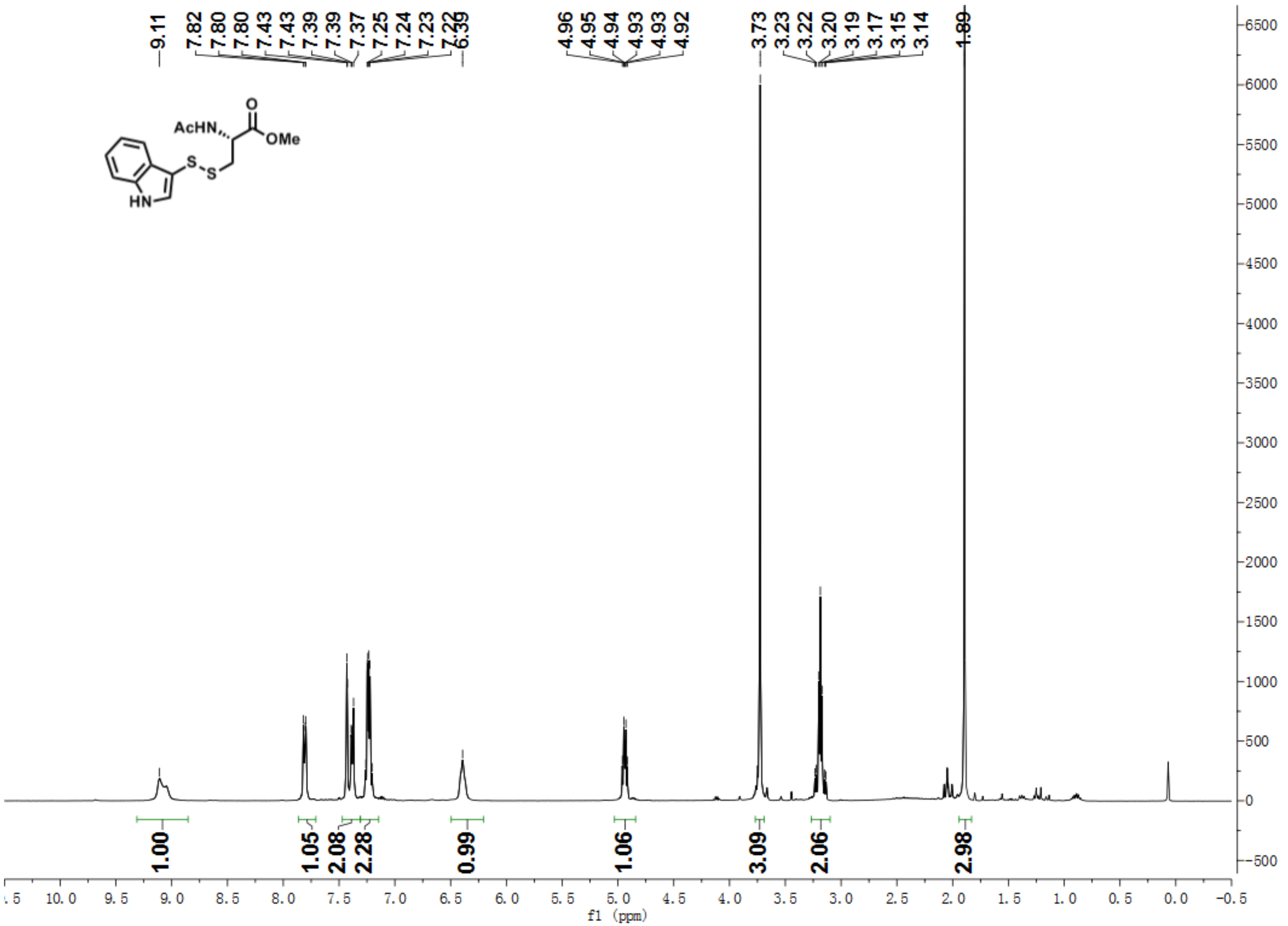

Supplementary Figure 116. ^{1}H NMR spectra for Compound **5t**.

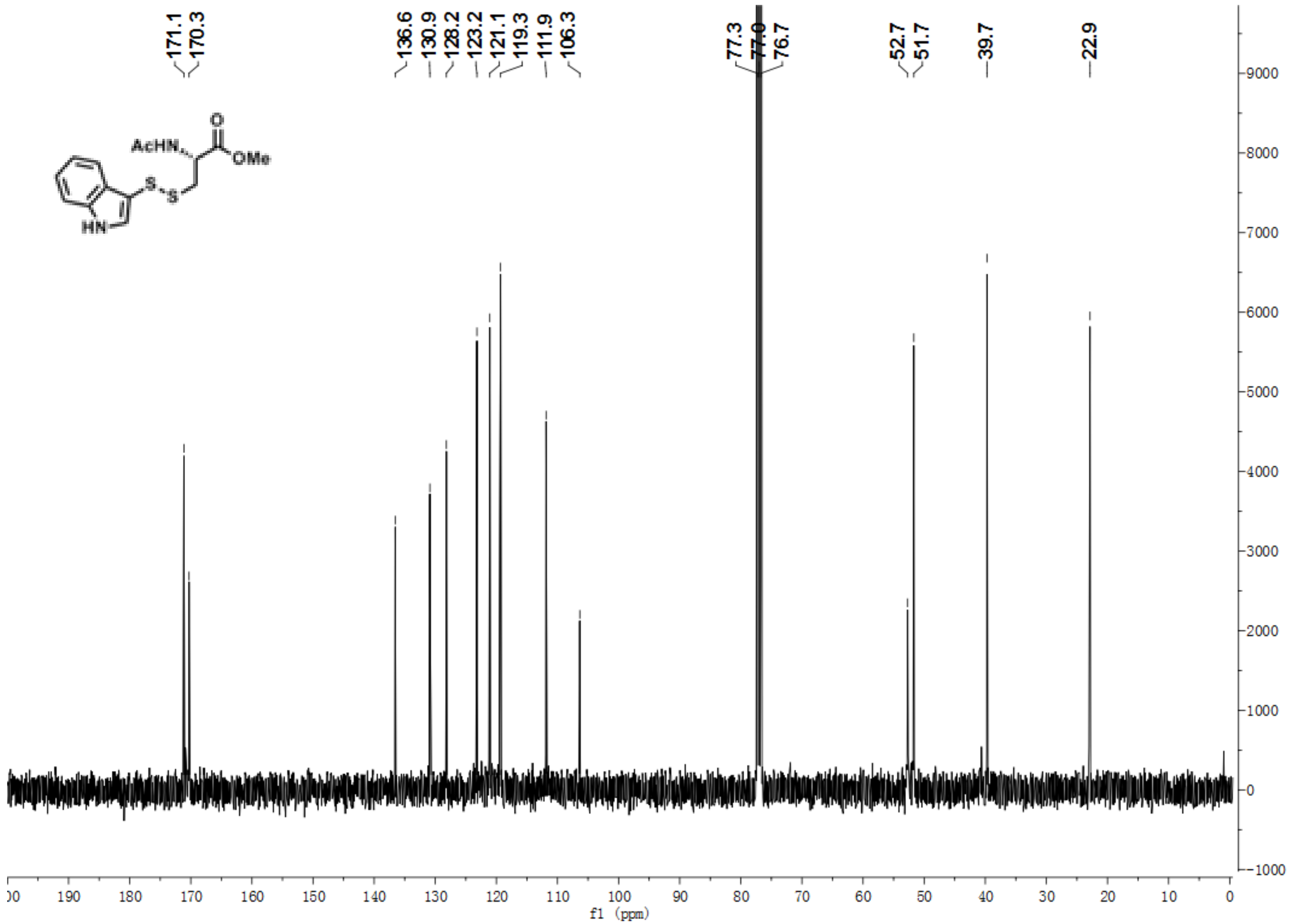

Supplementary Figure 117. ^{13}C NMR spectra for Compound **5t**.

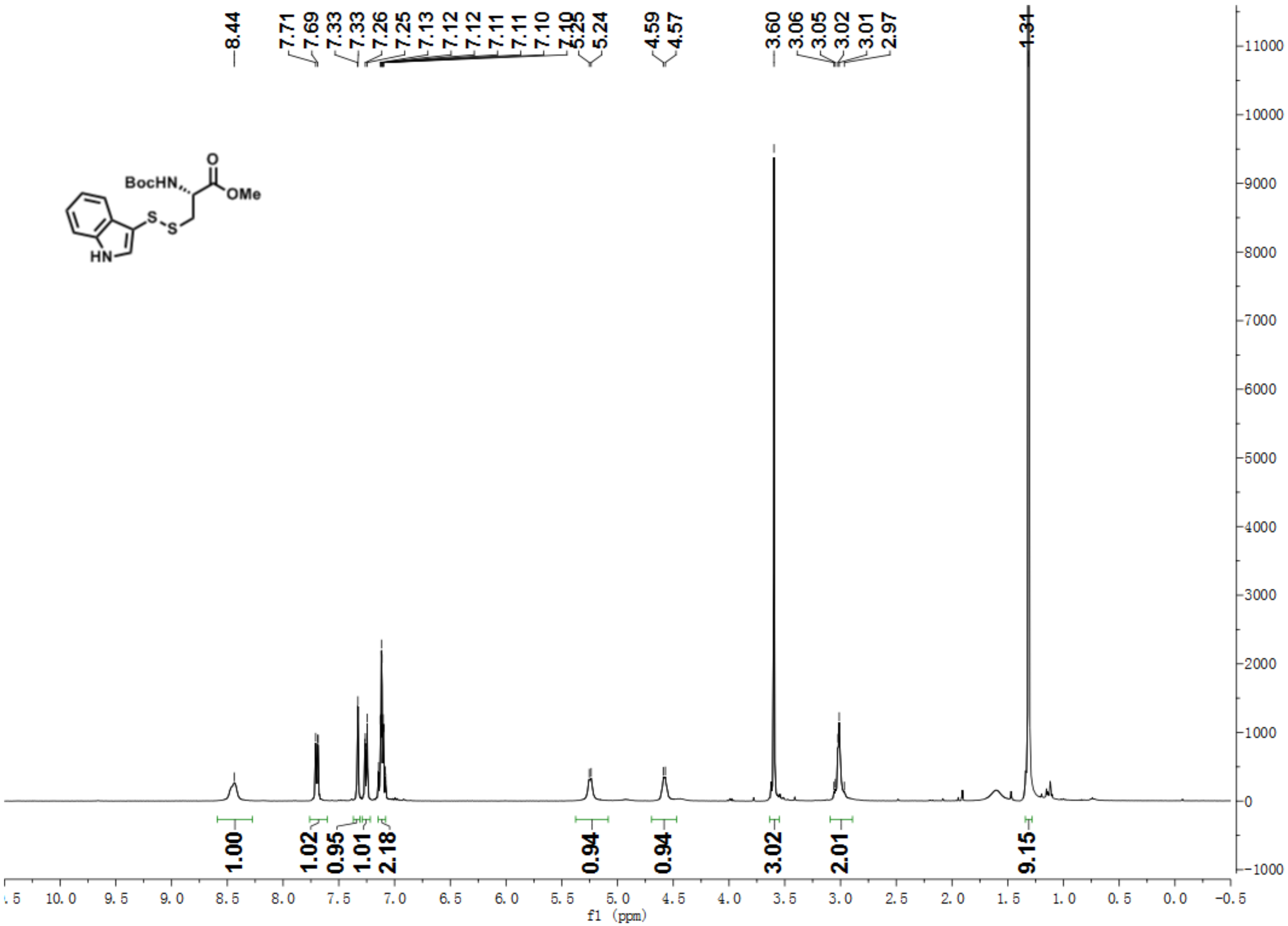

Supplementary Figure 118. ^{1}H NMR spectra for Compound **5u**.

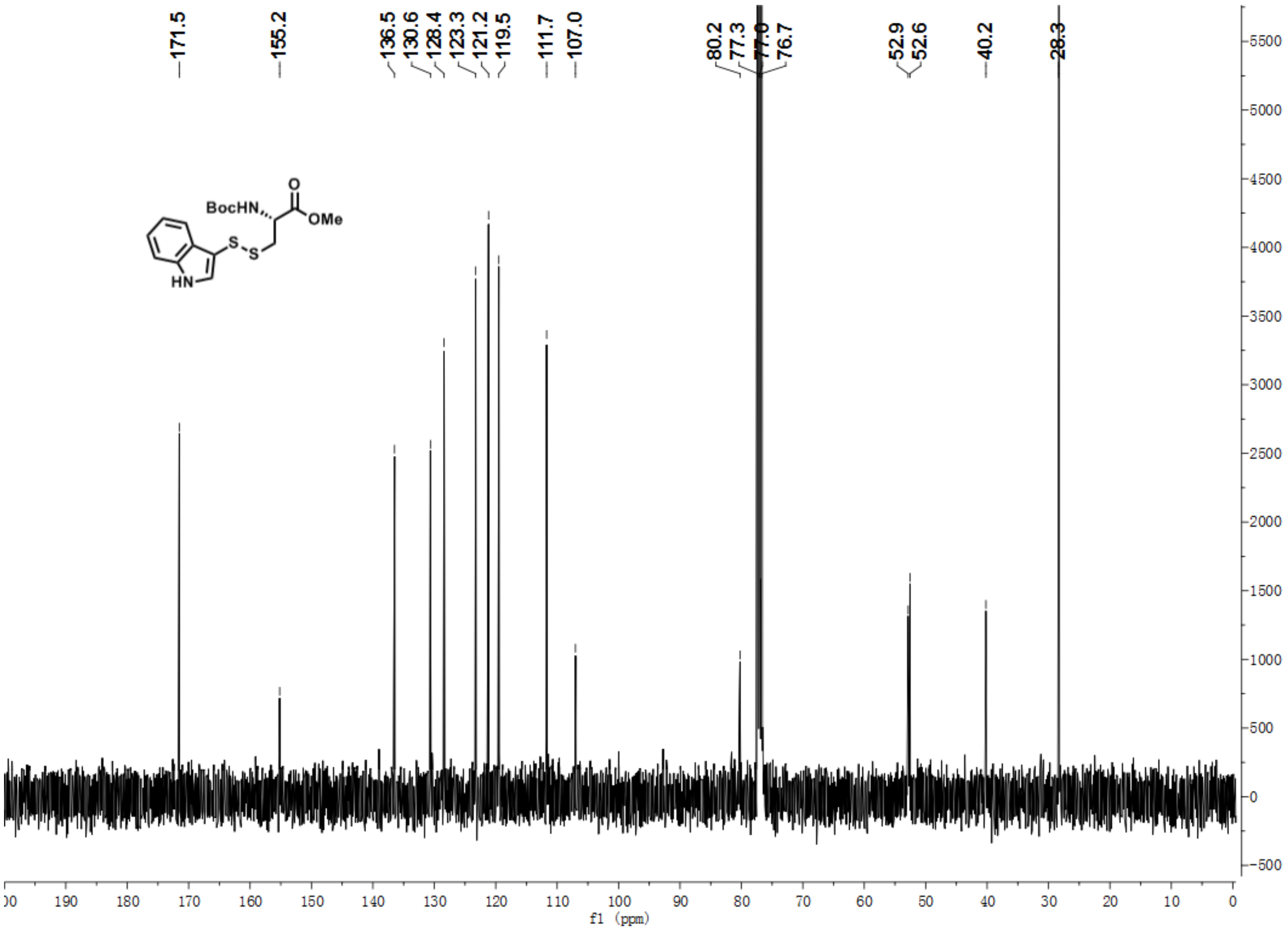

Supplementary Figure 119. ^{13}C NMR spectra for Compound **5u**.

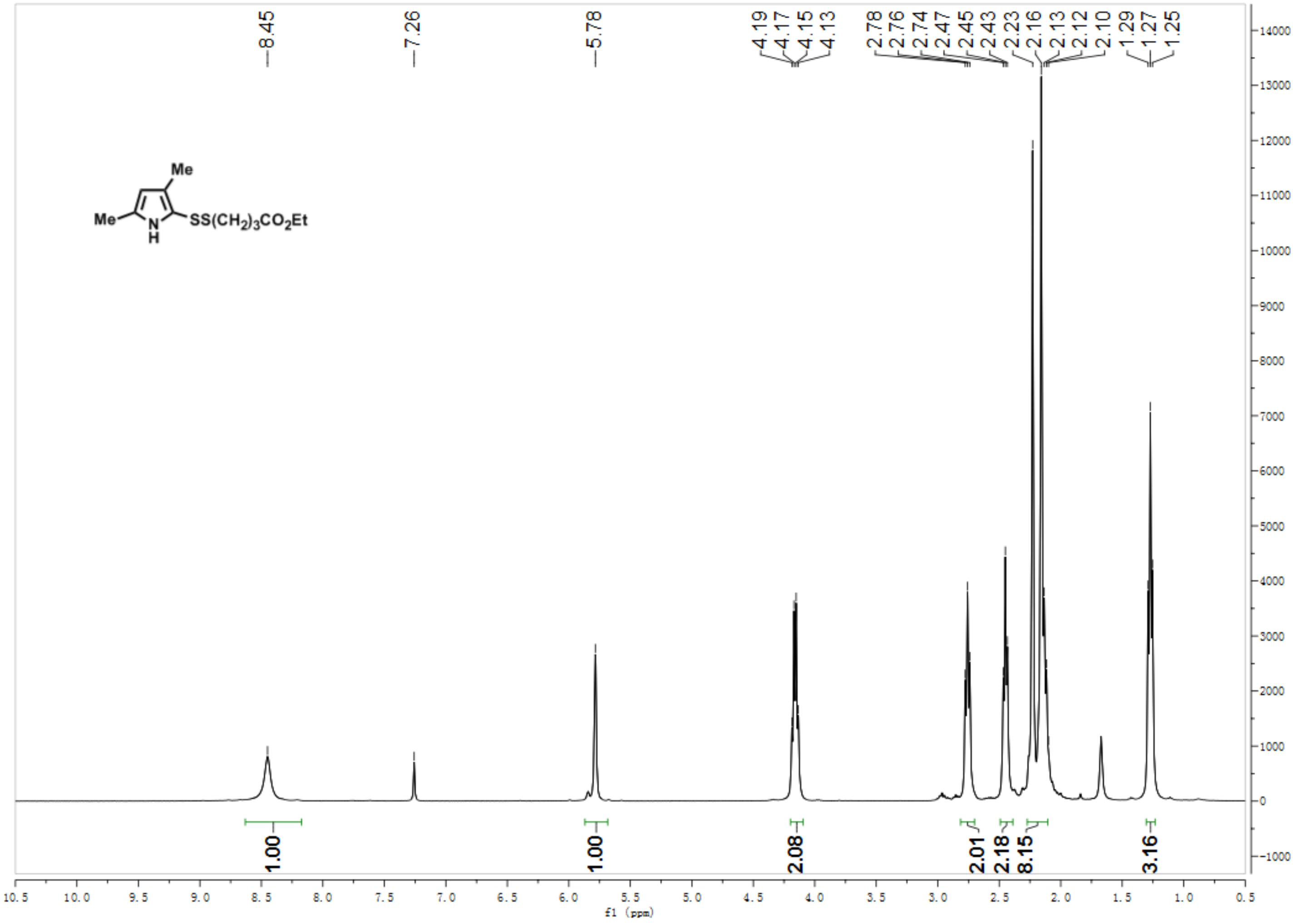

Supplementary Figure 120. ^{1}H NMR spectra for Compound **5v**.

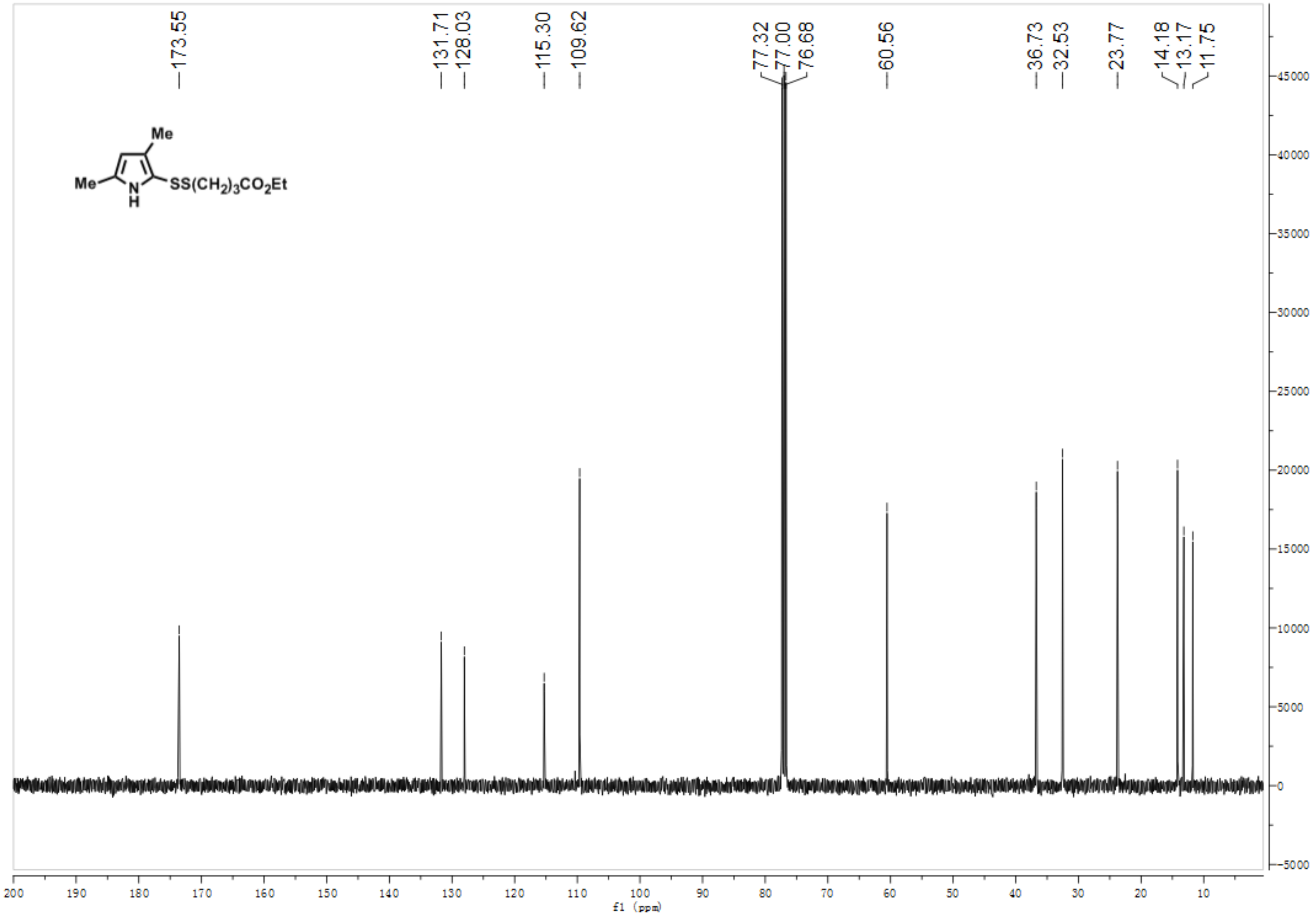

Supplementary Figure 121. ^{13}C NMR spectra for Compound **5v**.

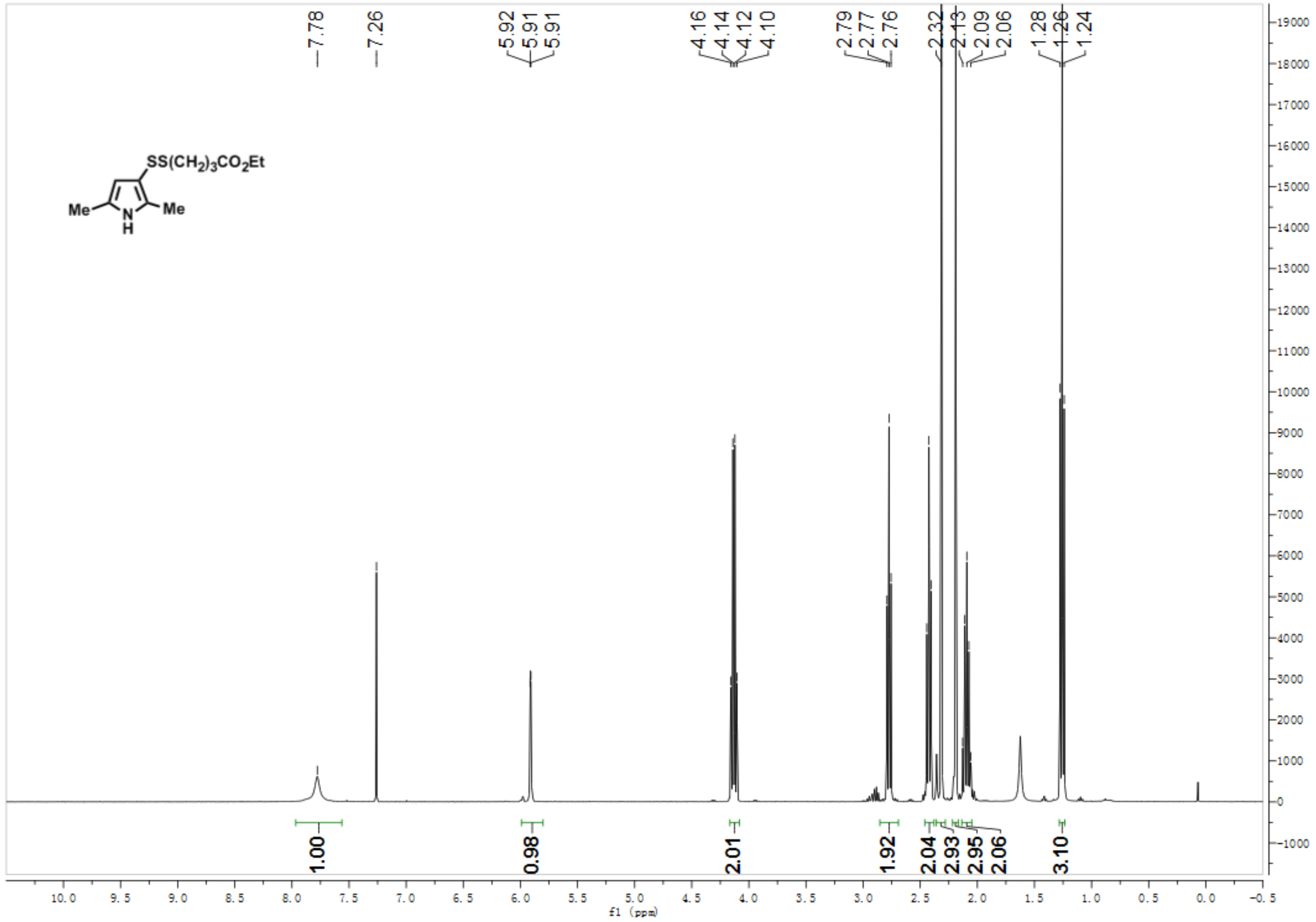

Supplementary Figure 122. ^{1}H NMR spectra for Compound **5w**.

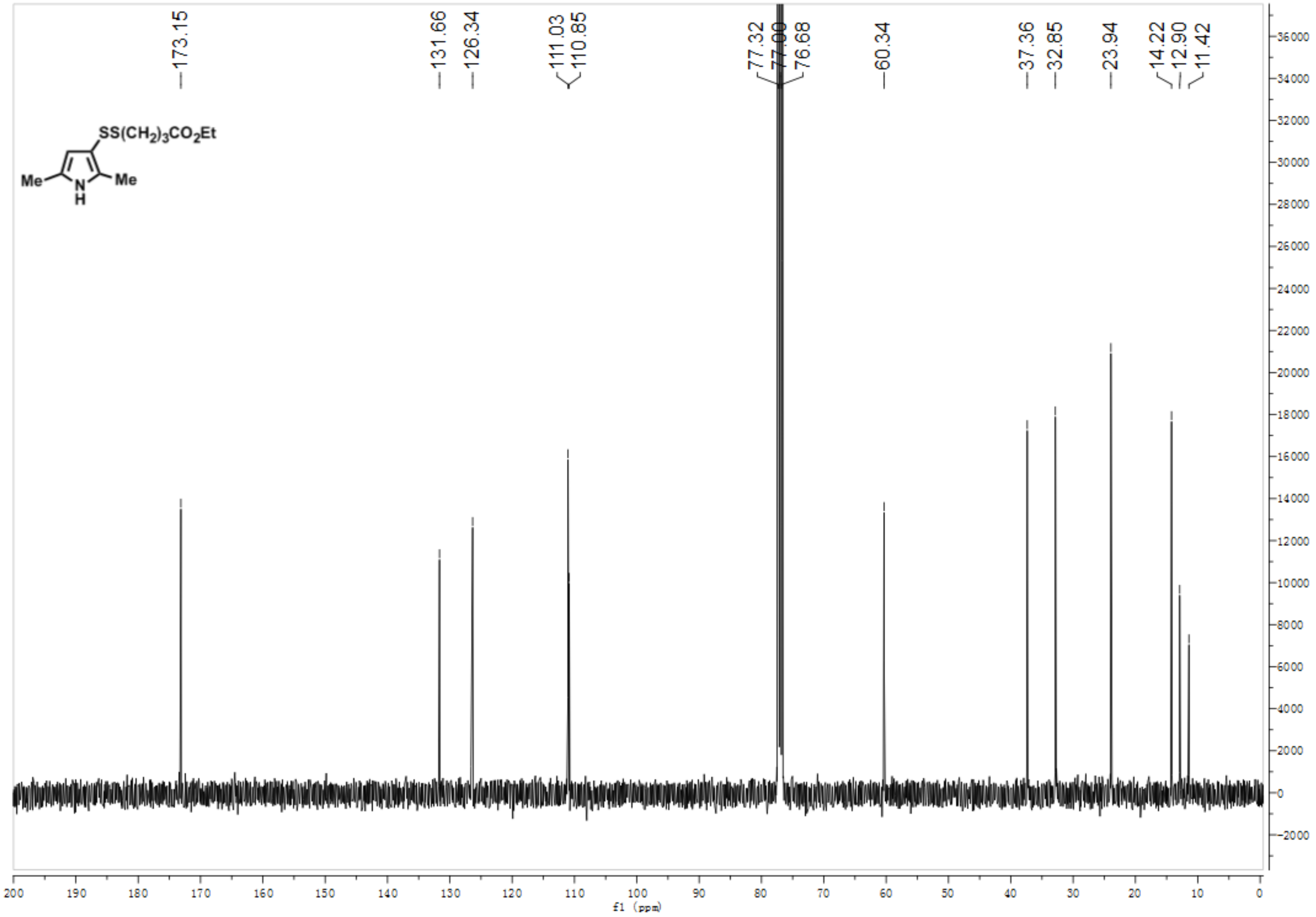

Supplementary Figure 123. ^{13}C NMR spectra for Compound **5w**.

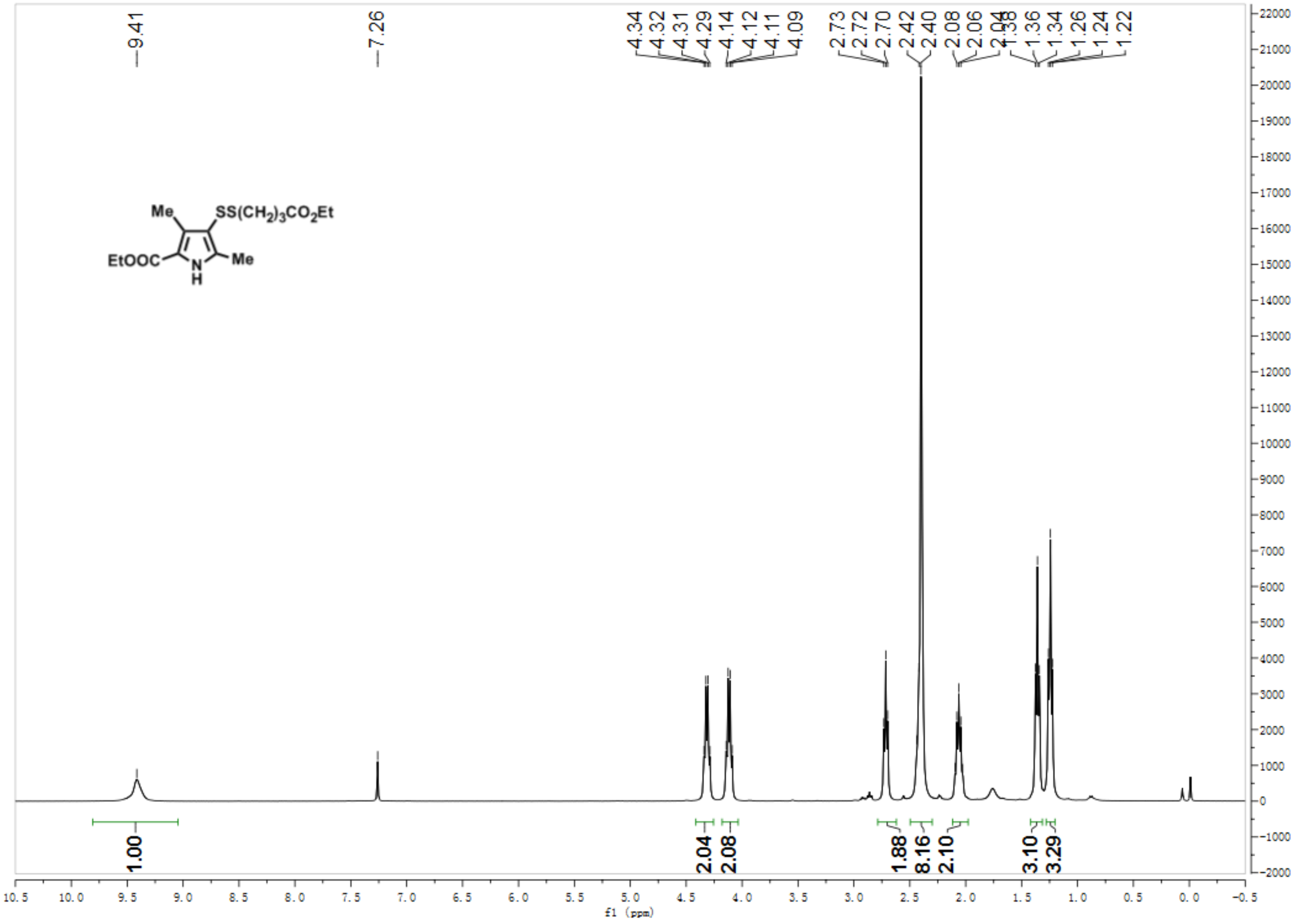

Supplementary Figure 124. ^{1}H NMR spectra for Compound **5x**.

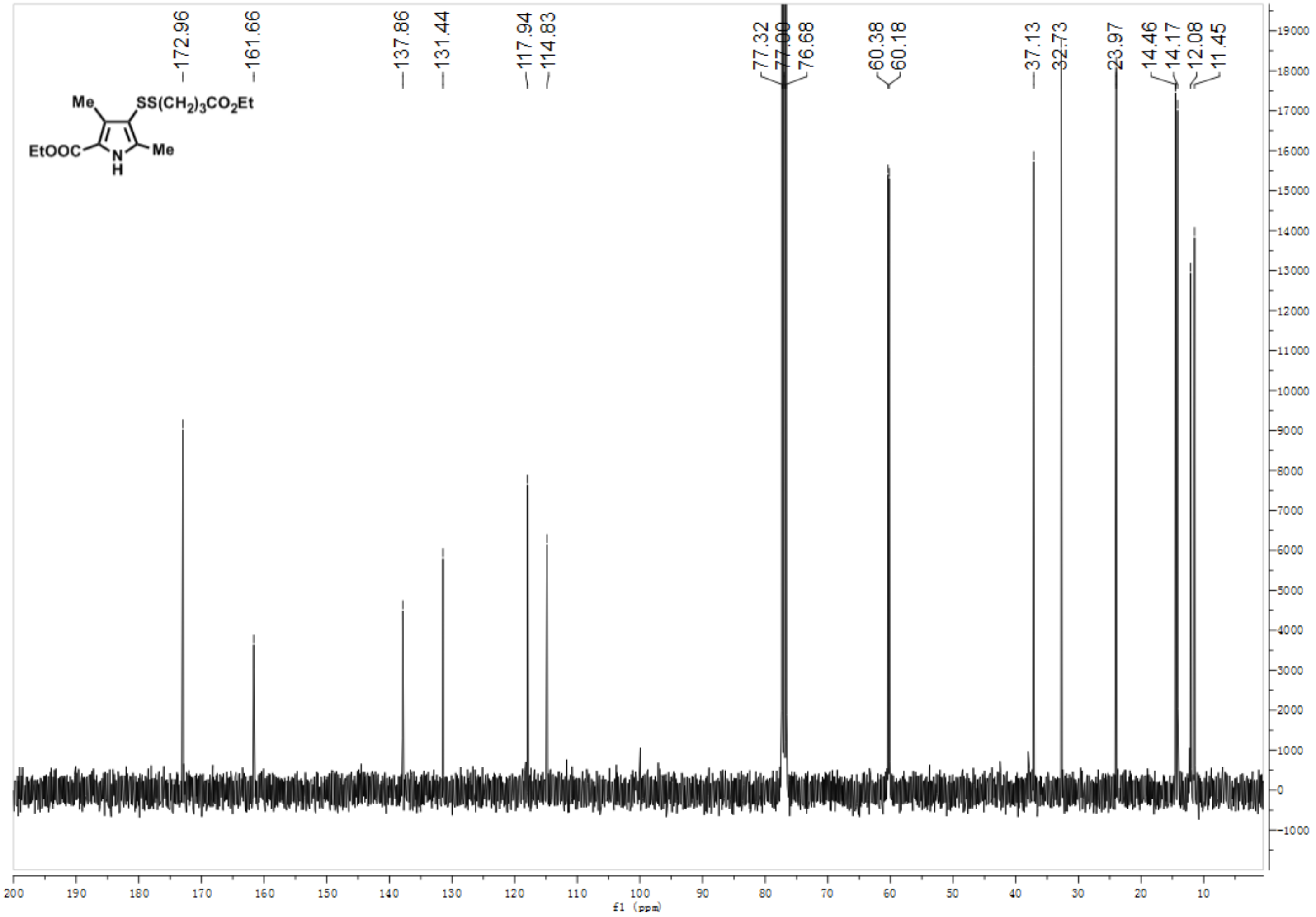

Supplementary Figure 125. ^{13}C NMR spectra for Compound **5x**.

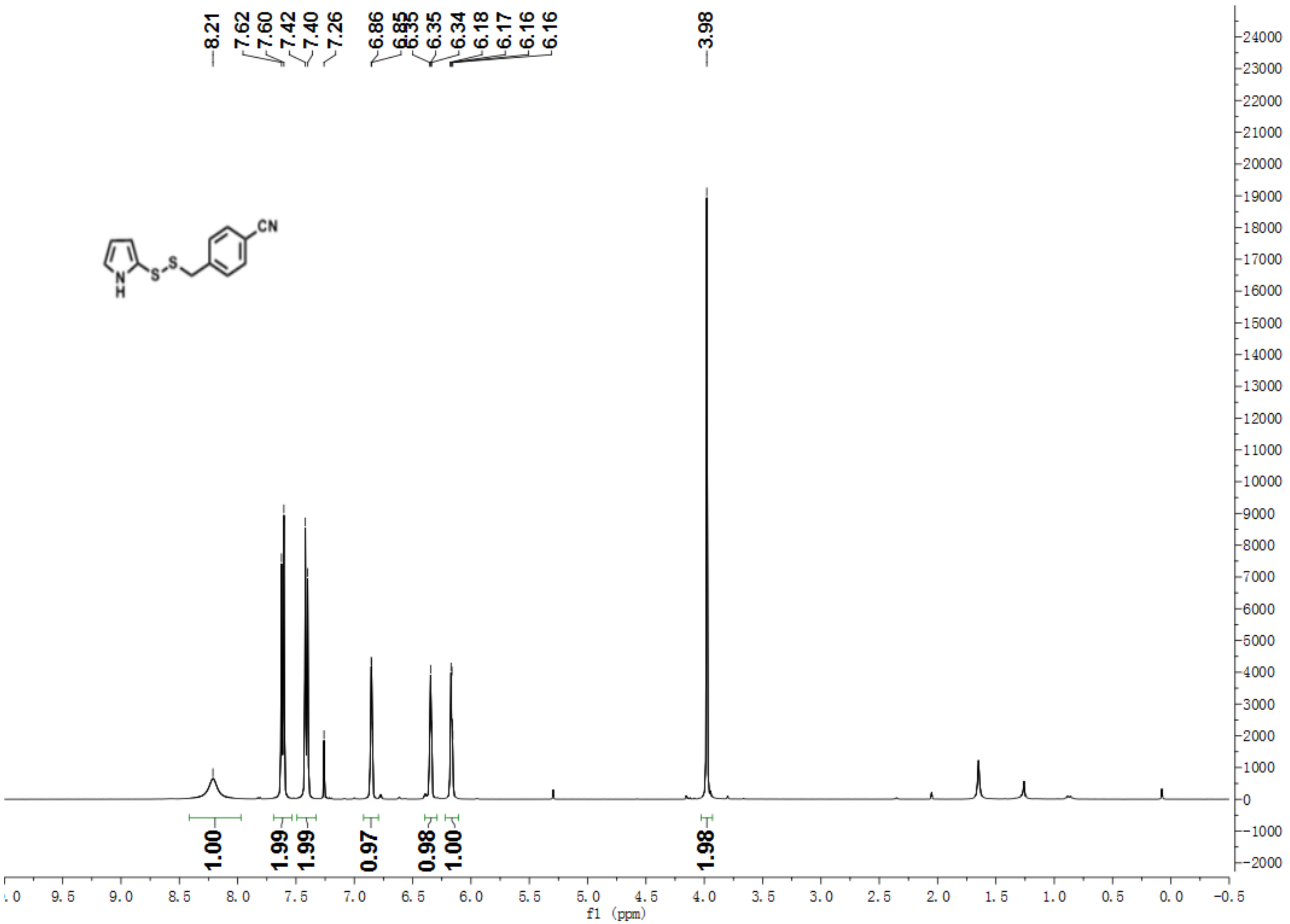

Supplementary Figure 126. ^{1}H NMR spectra for Compound **5y**.

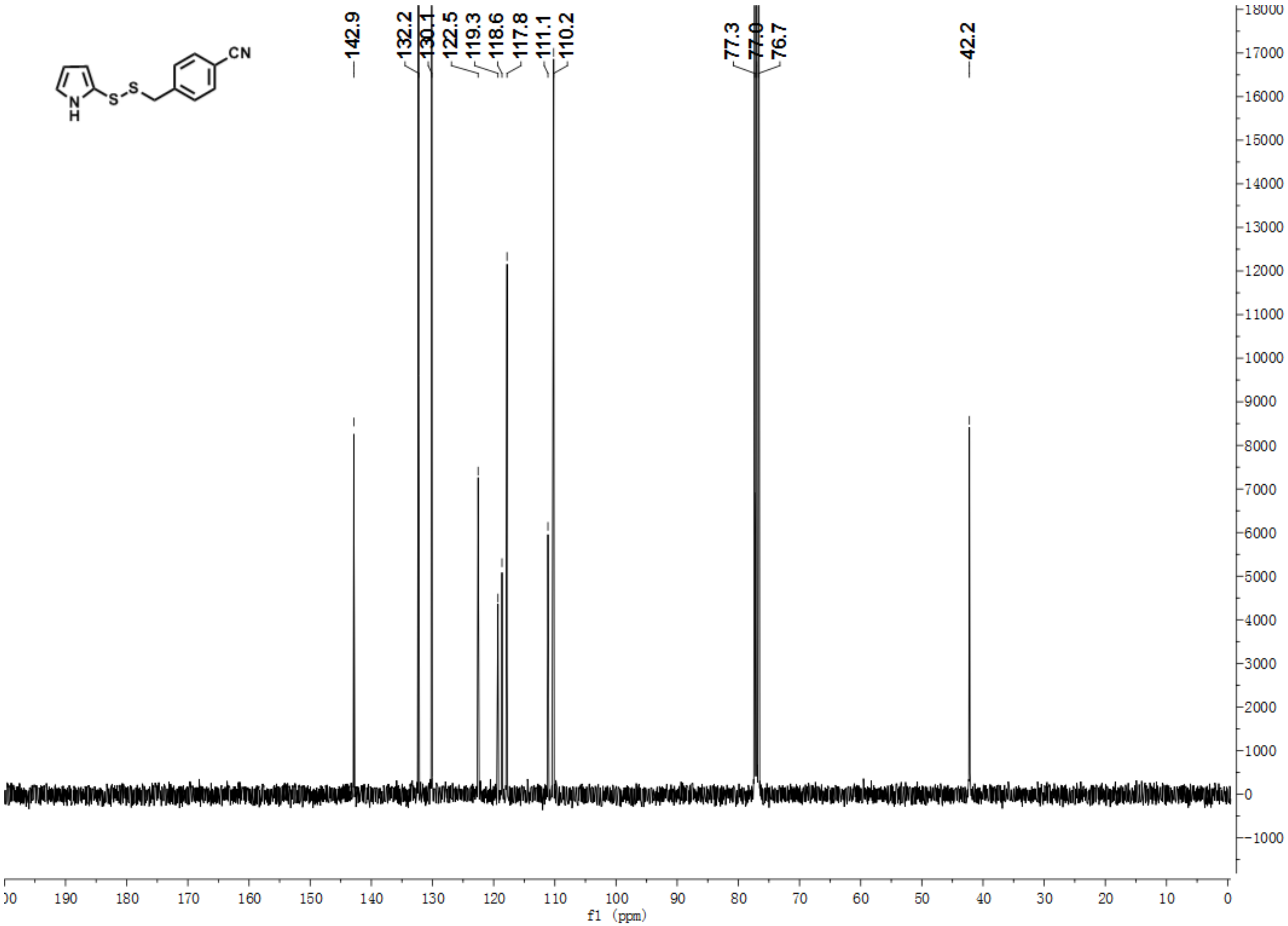

Supplementary Figure 127. ^{13}C NMR spectra for Compound **5y**.

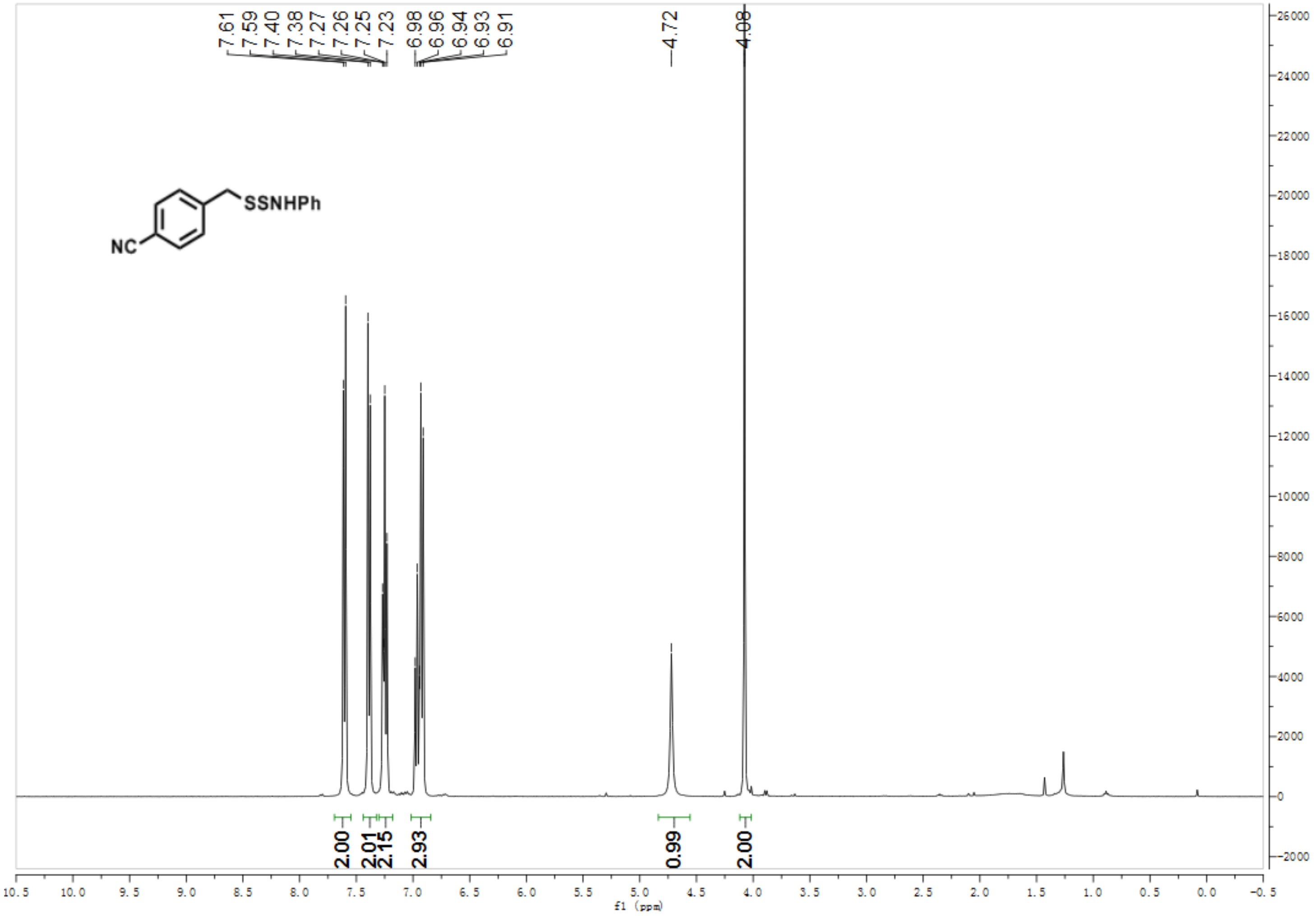

Supplementary Figure 128. ^{1}H NMR spectra for Compound **6a**.

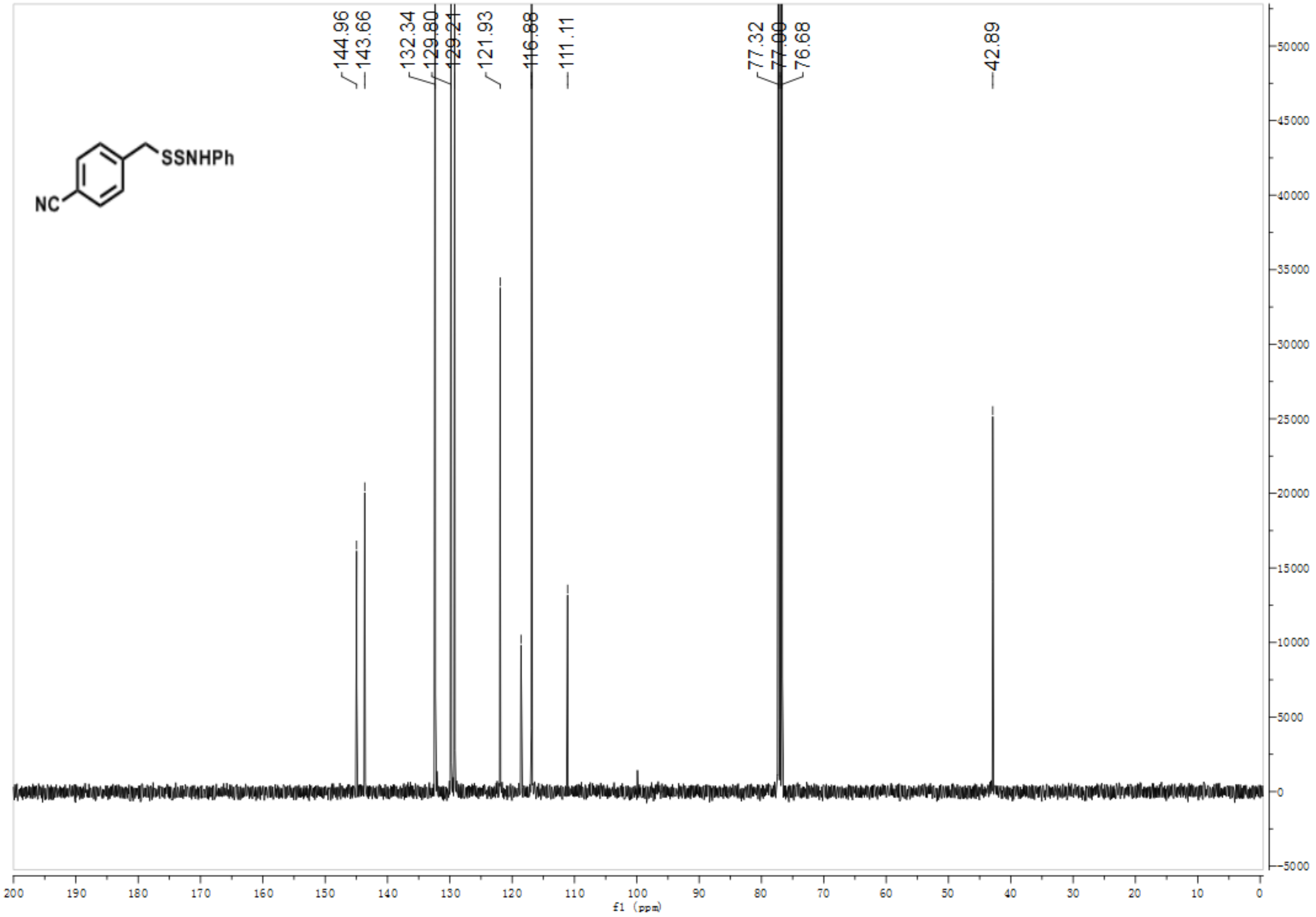

Supplementary Figure 129. ^{13}C NMR spectra for Compound **6a**.

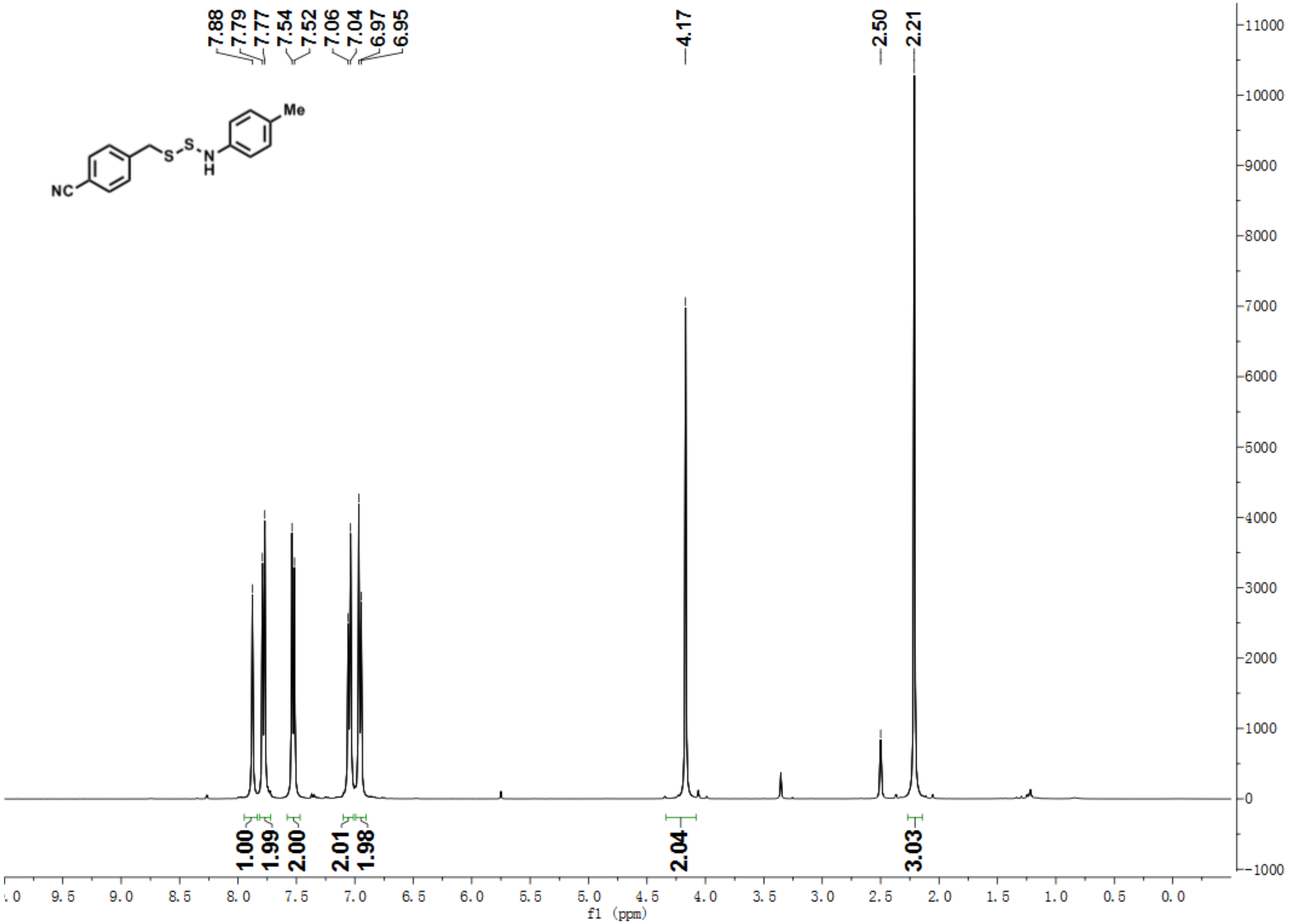

Supplementary Figure 130. ^{1}H NMR spectra for Compound **6b**.

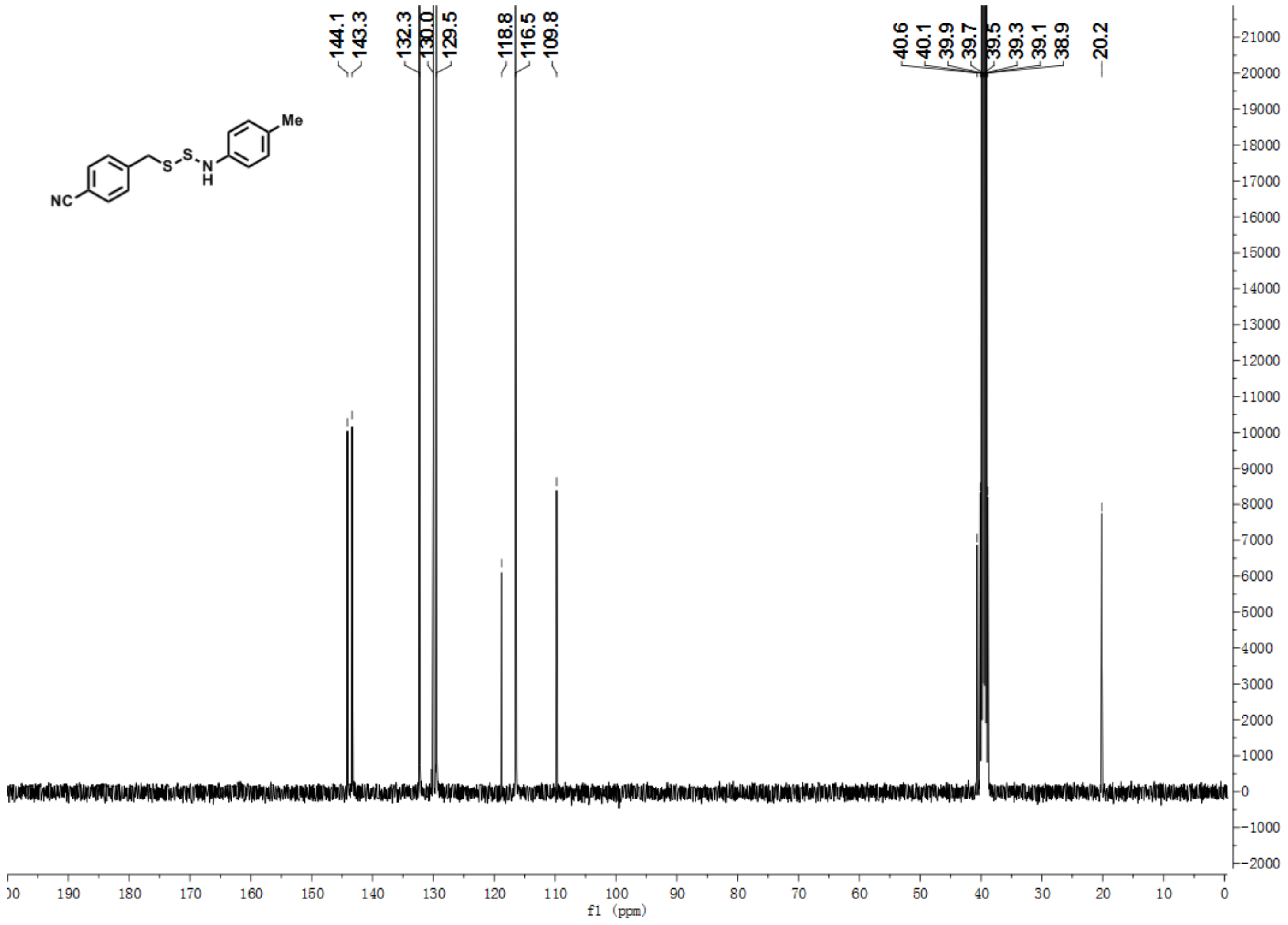

Supplementary Figure 131. ^{13}C NMR spectra for Compound **6b**.

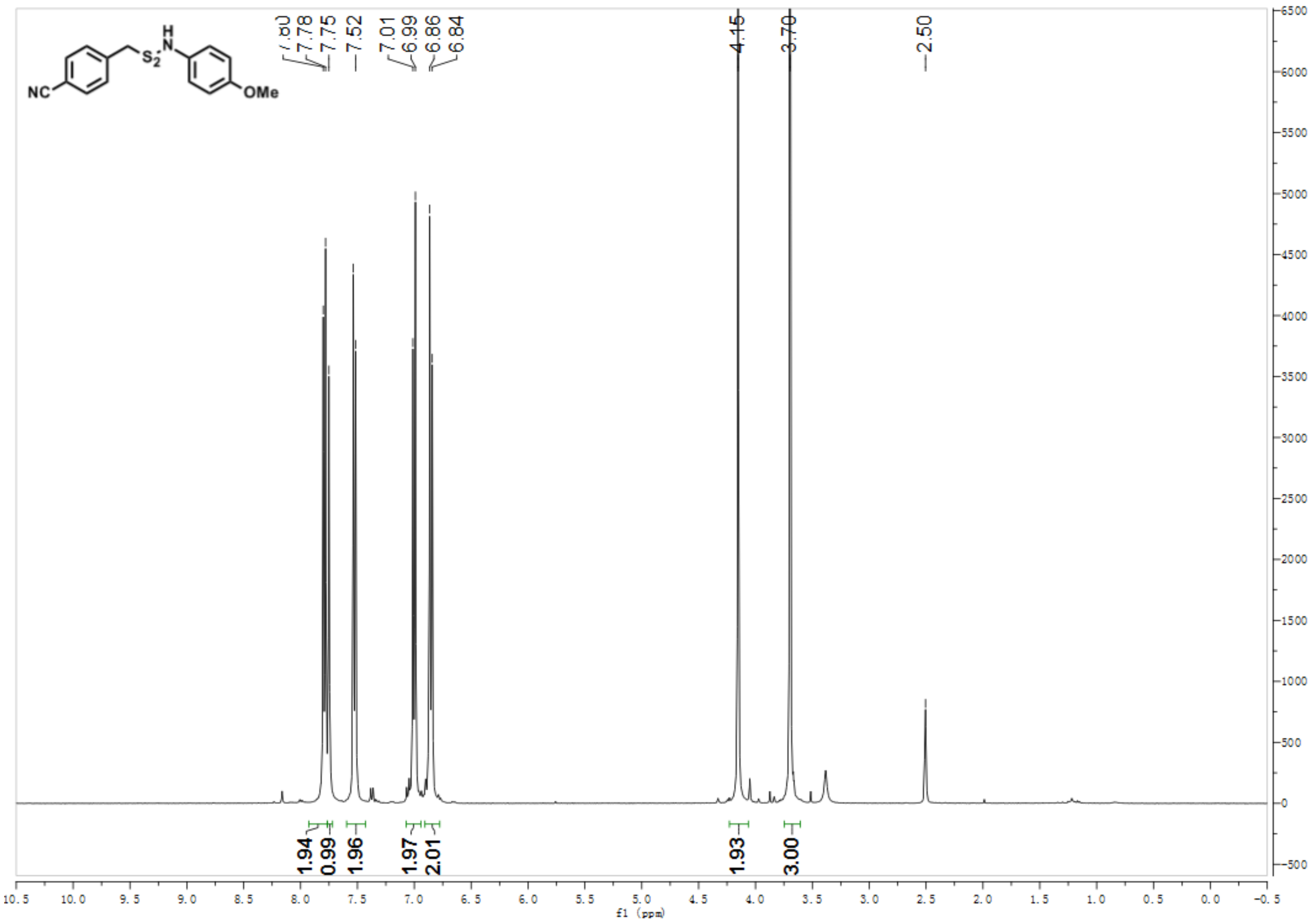

Supplementary Figure 132. ^{1}H NMR spectra for Compound **6c**.

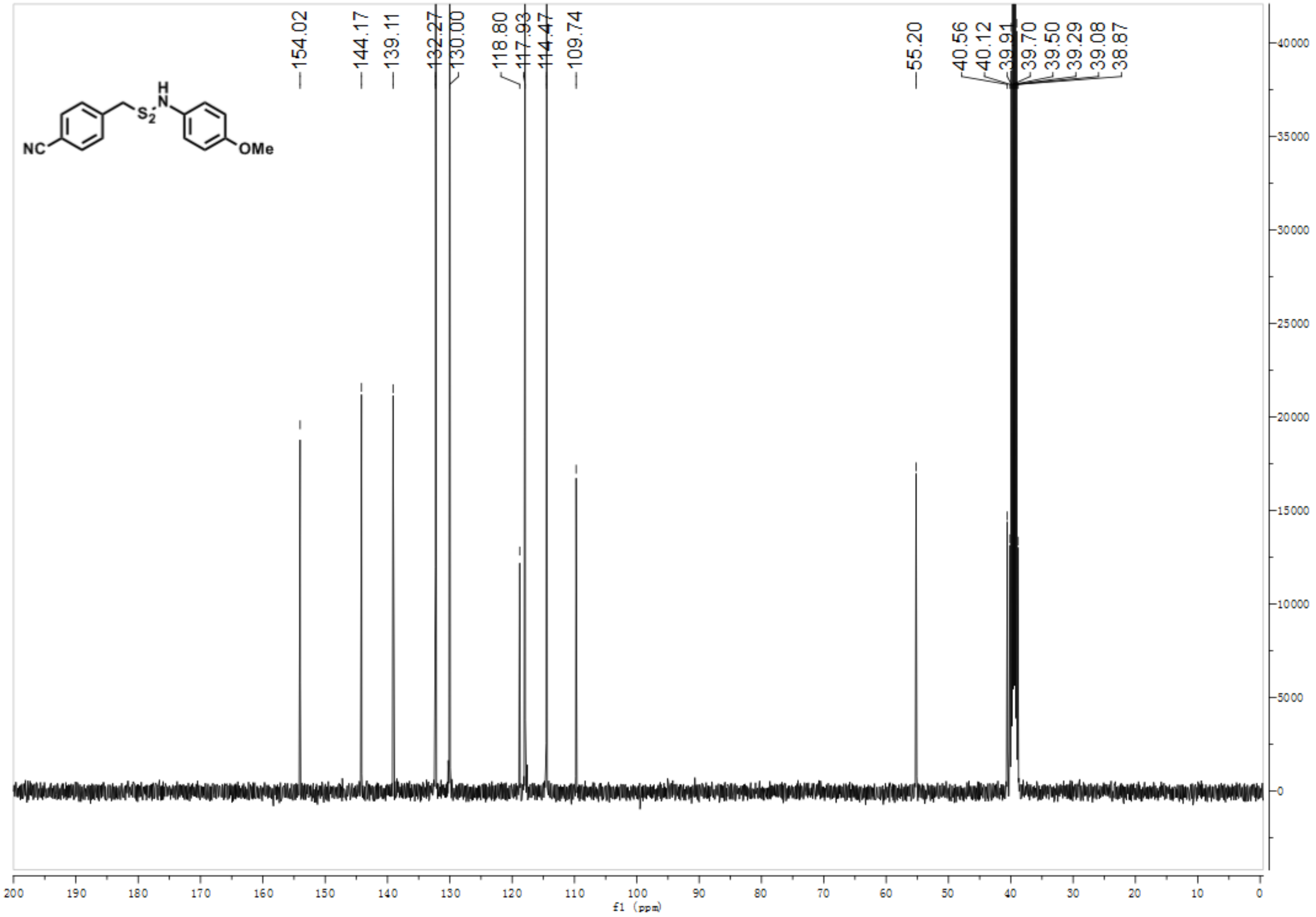

Supplementary Figure 133. ^{13}C NMR spectra for Compound **6c**.

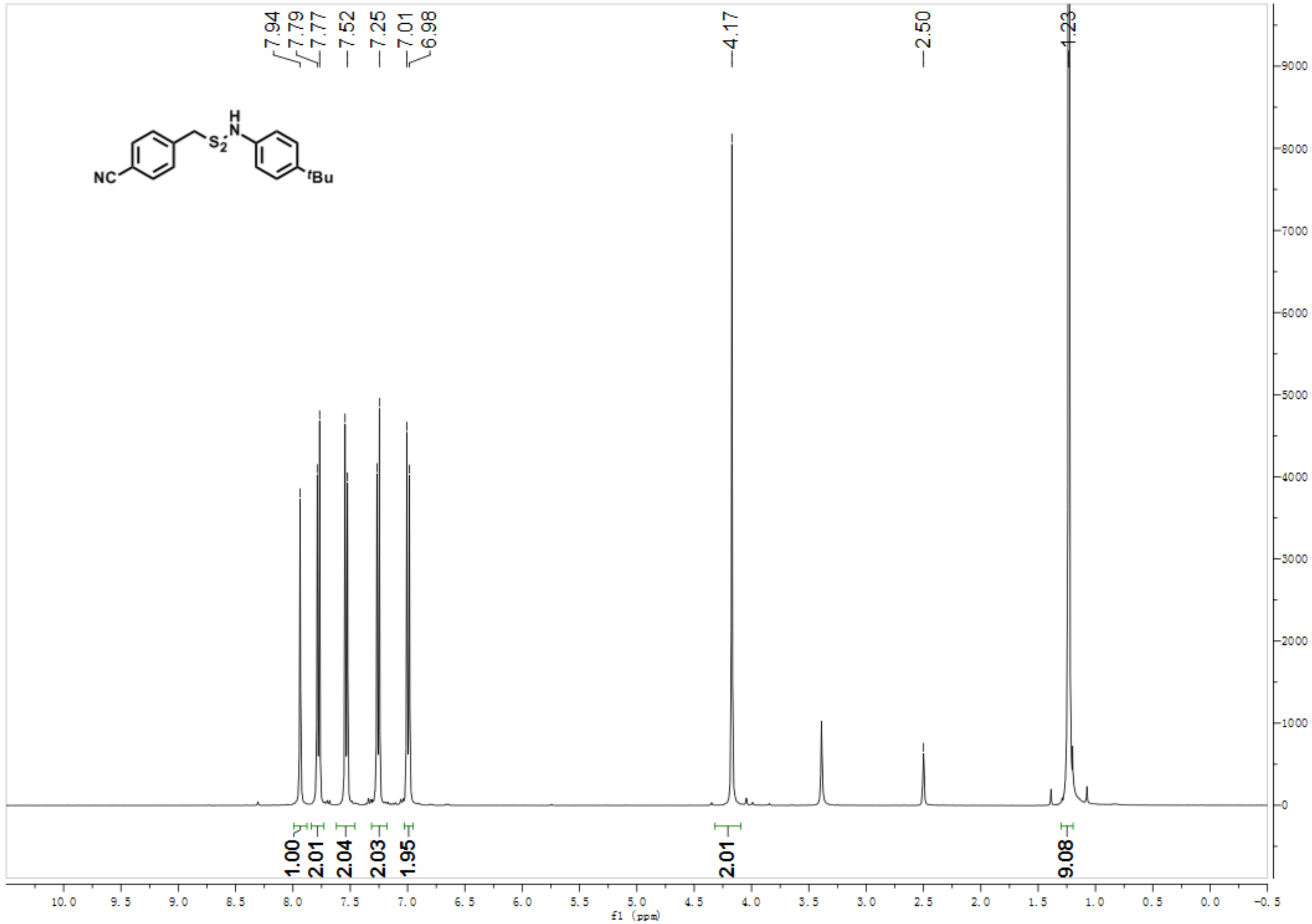

Supplementary Figure 134. ^{1}H NMR spectra for Compound **6d**.

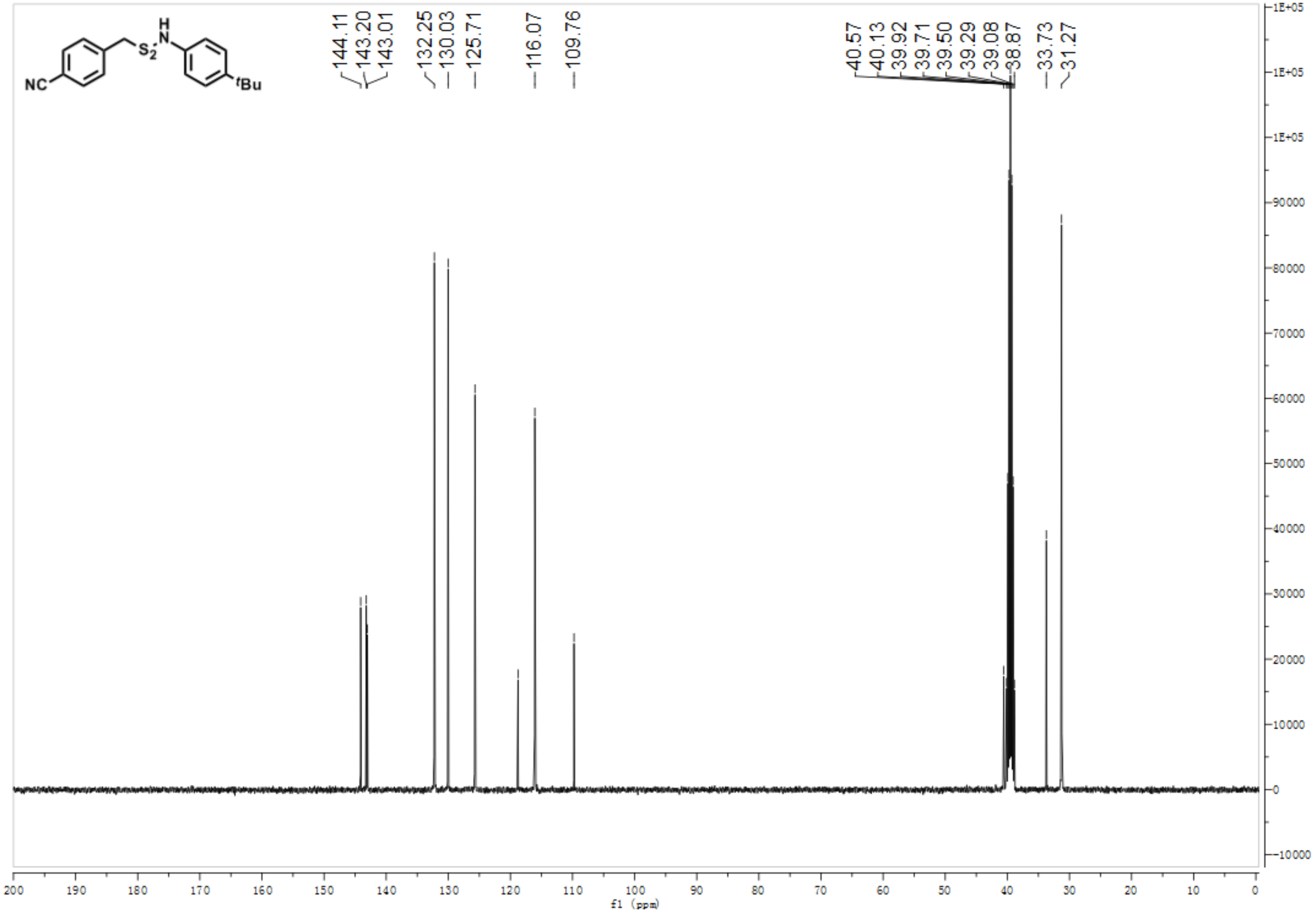

Supplementary Figure 135. ^{13}C NMR spectra for Compound **6d**.

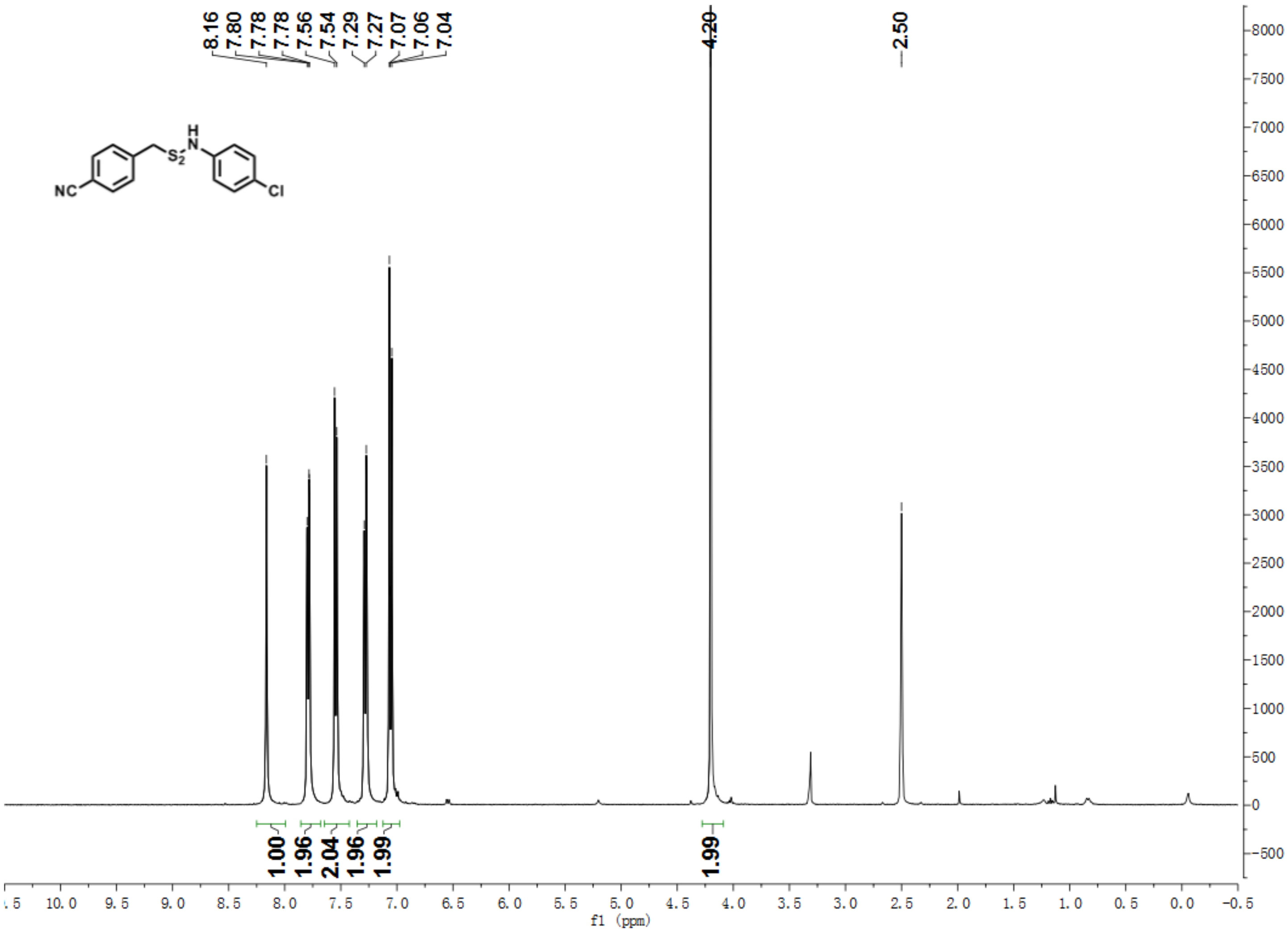

Supplementary Figure 136. ^{1}H NMR spectra for Compound **6e**.

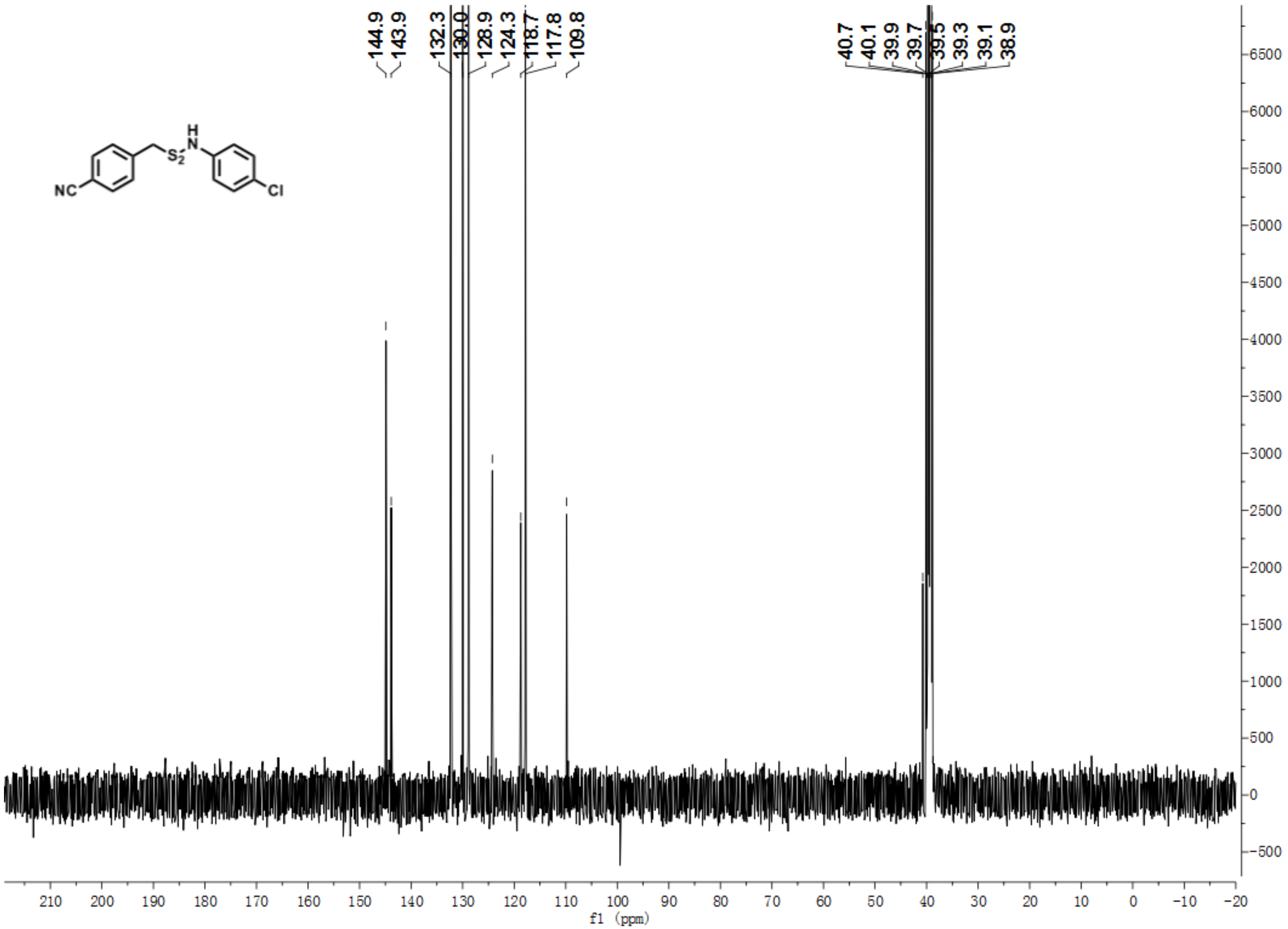

Supplementary Figure 137. ^{13}C NMR spectra for Compound **6e**.

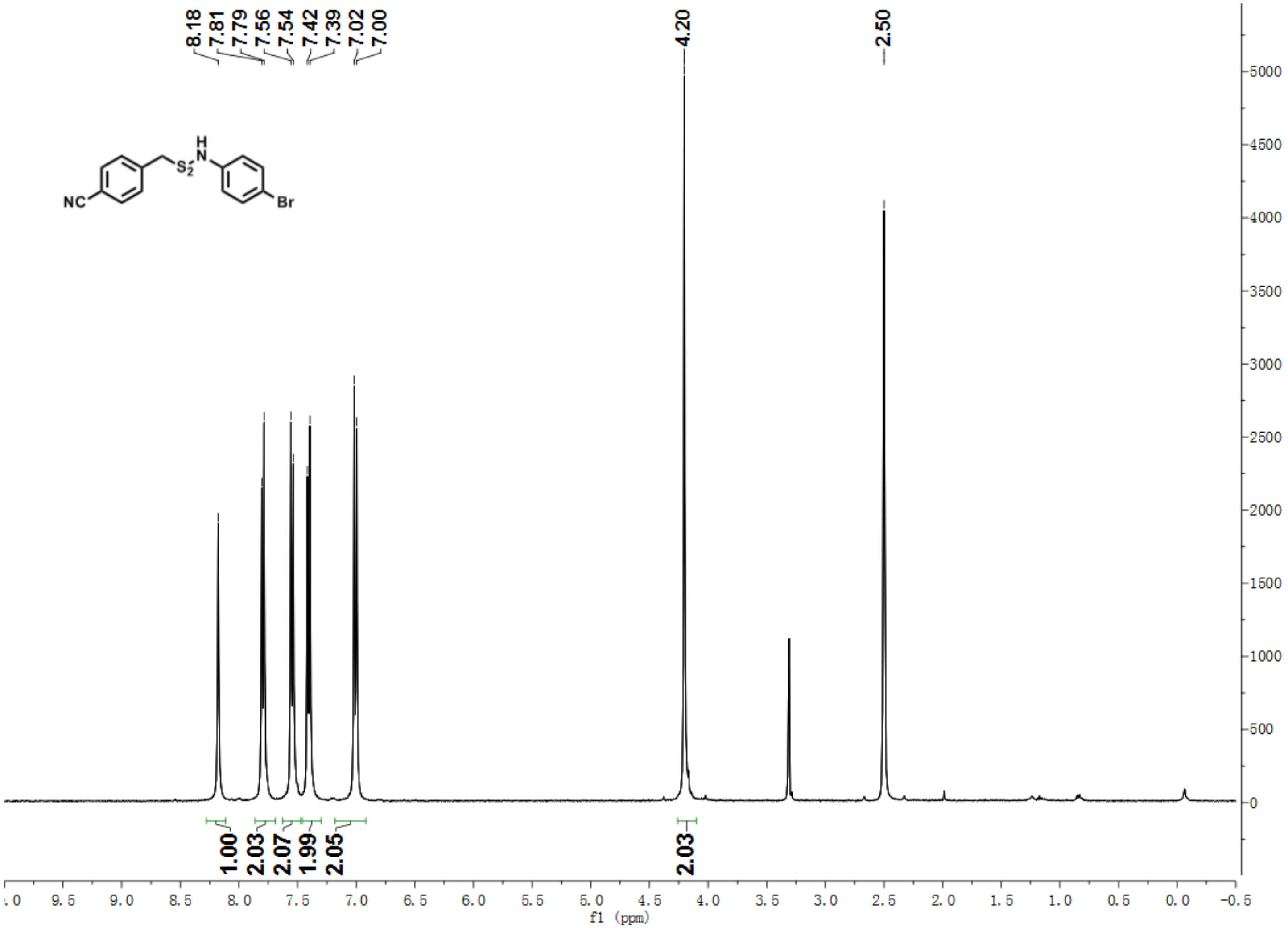

Supplementary Figure 138. ^{1}H NMR spectra for Compound **6f**.

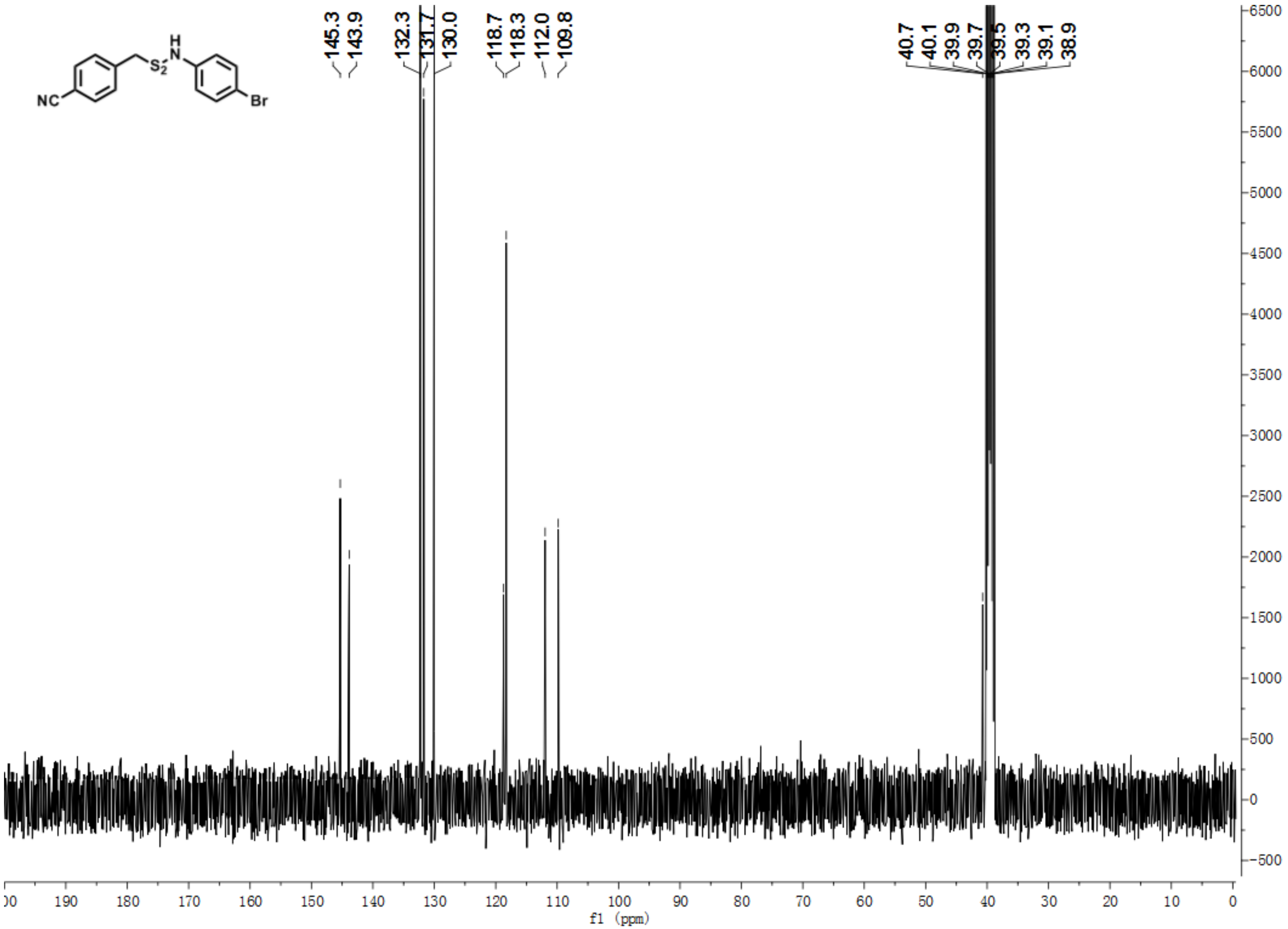

Supplementary Figure 139. ^{13}C NMR spectra for Compound **6f**.

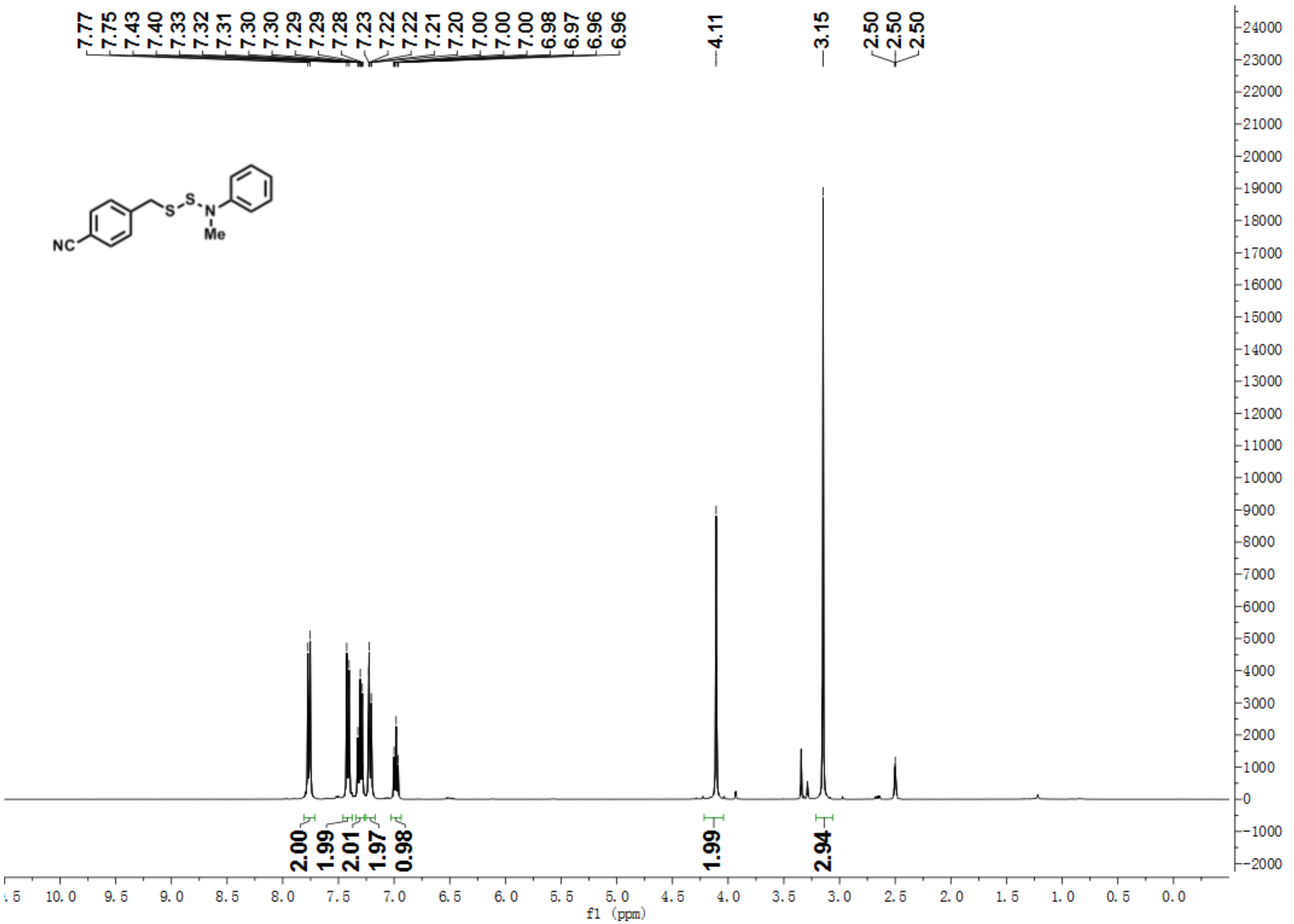

Supplementary Figure 140. ^{1}H NMR spectra for Compound **6g**.

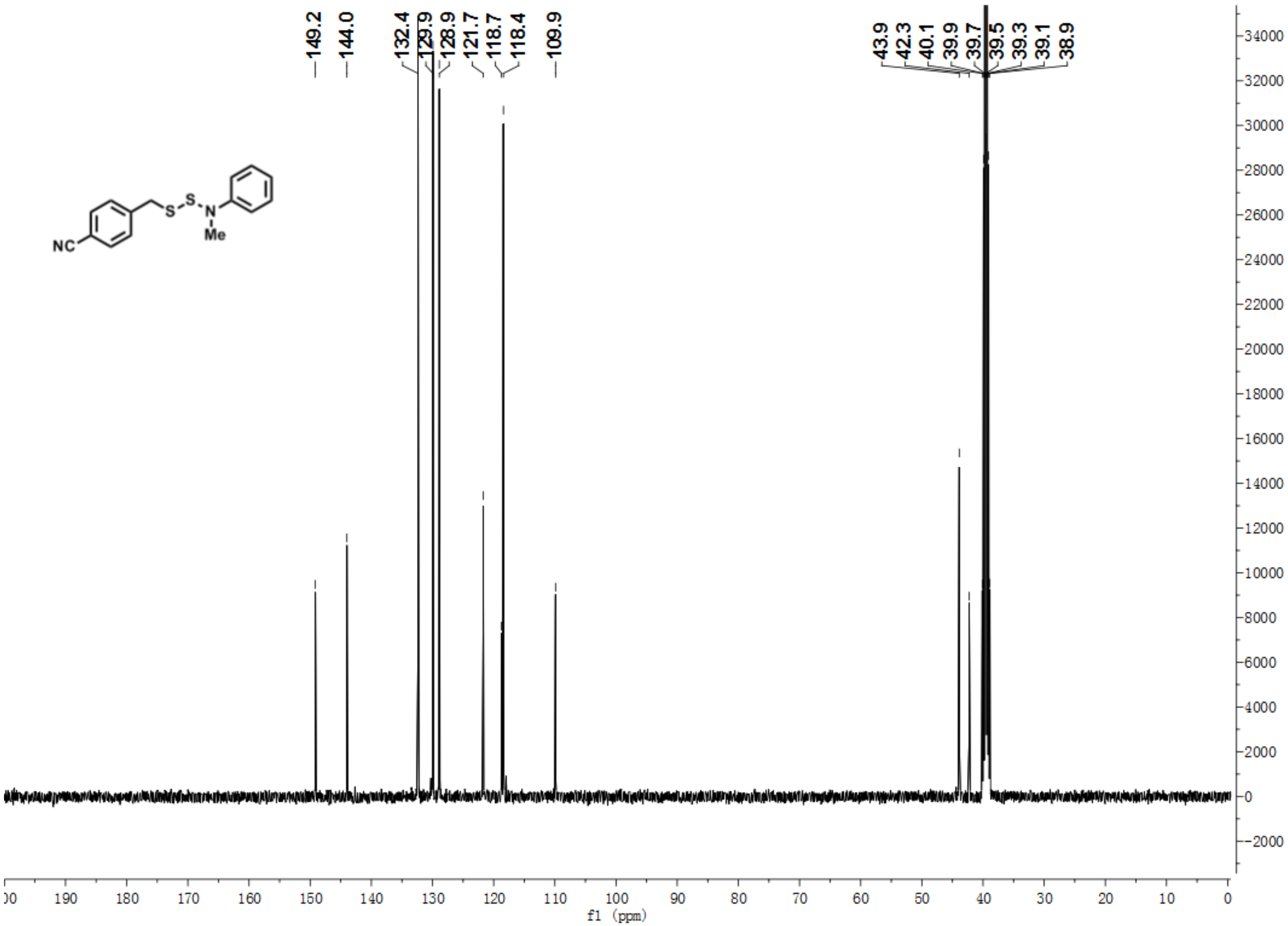

Supplementary Figure 141. ^{13}C NMR spectra for Compound **6g**.

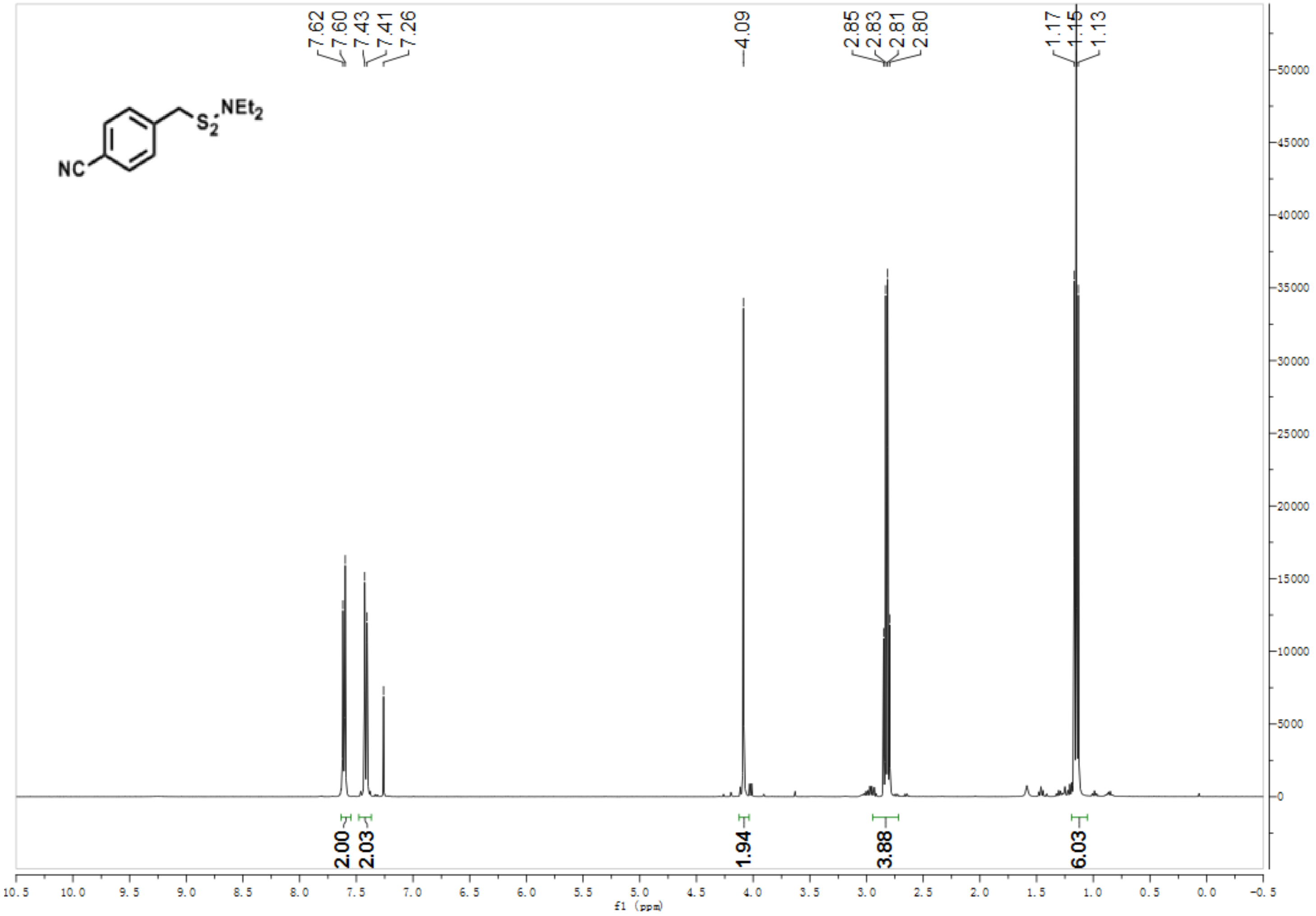

Supplementary Figure 142. ^{1}H NMR spectra for Compound **6h**.

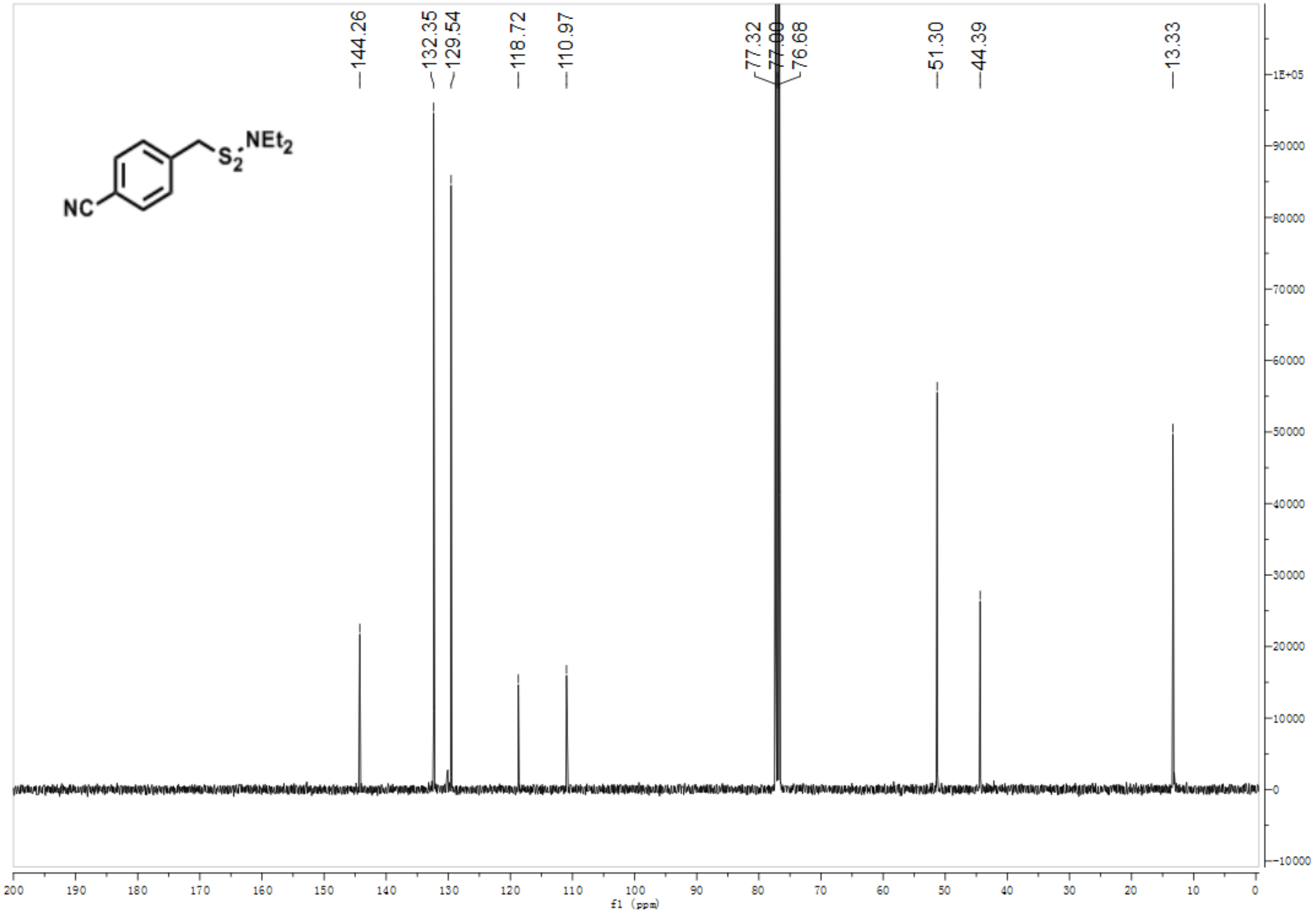

Supplementary Figure 143. ^{13}C NMR spectra for Compound **6h**.

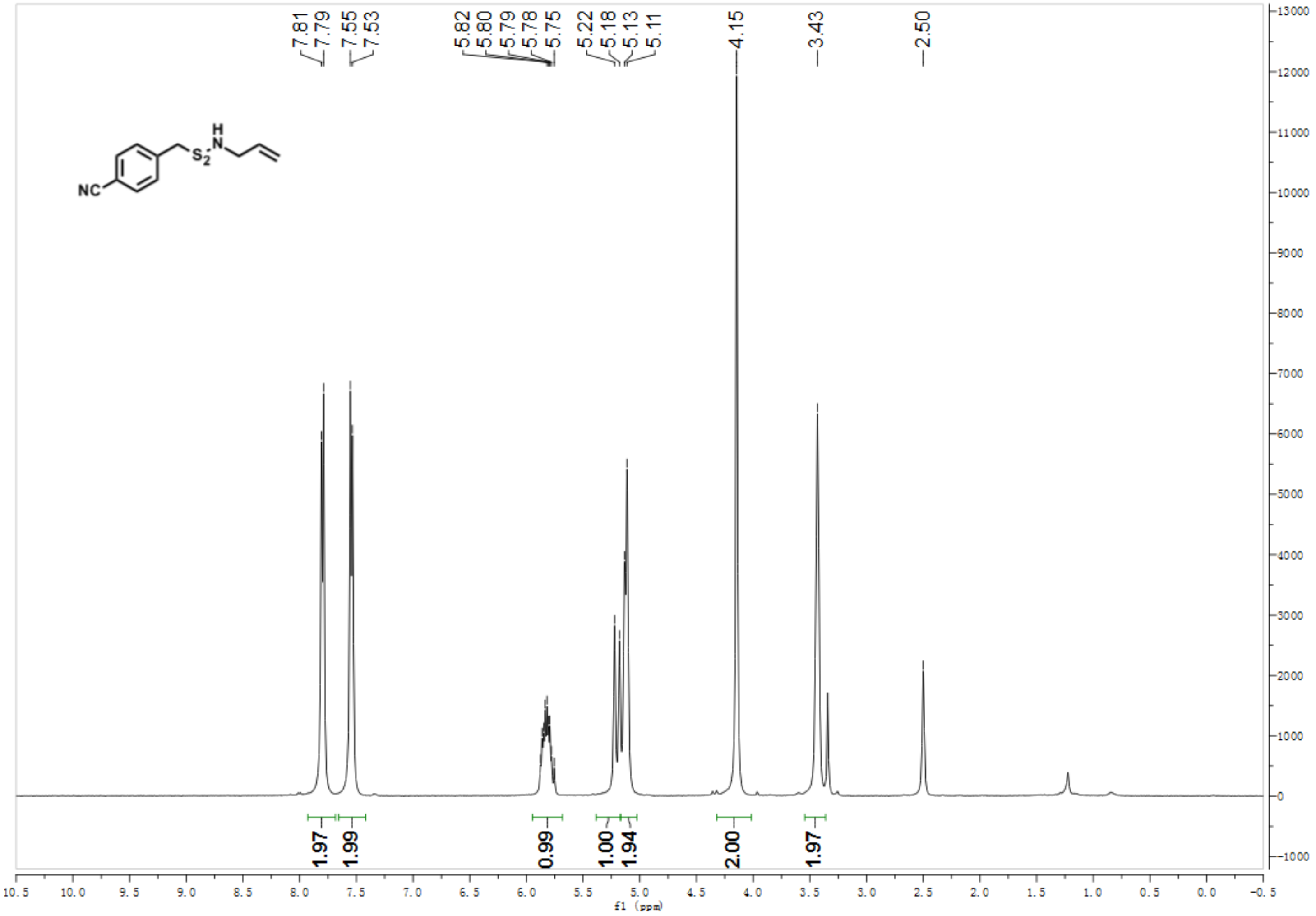

Supplementary Figure 144. ^{1}H NMR spectra for Compound **6i**.

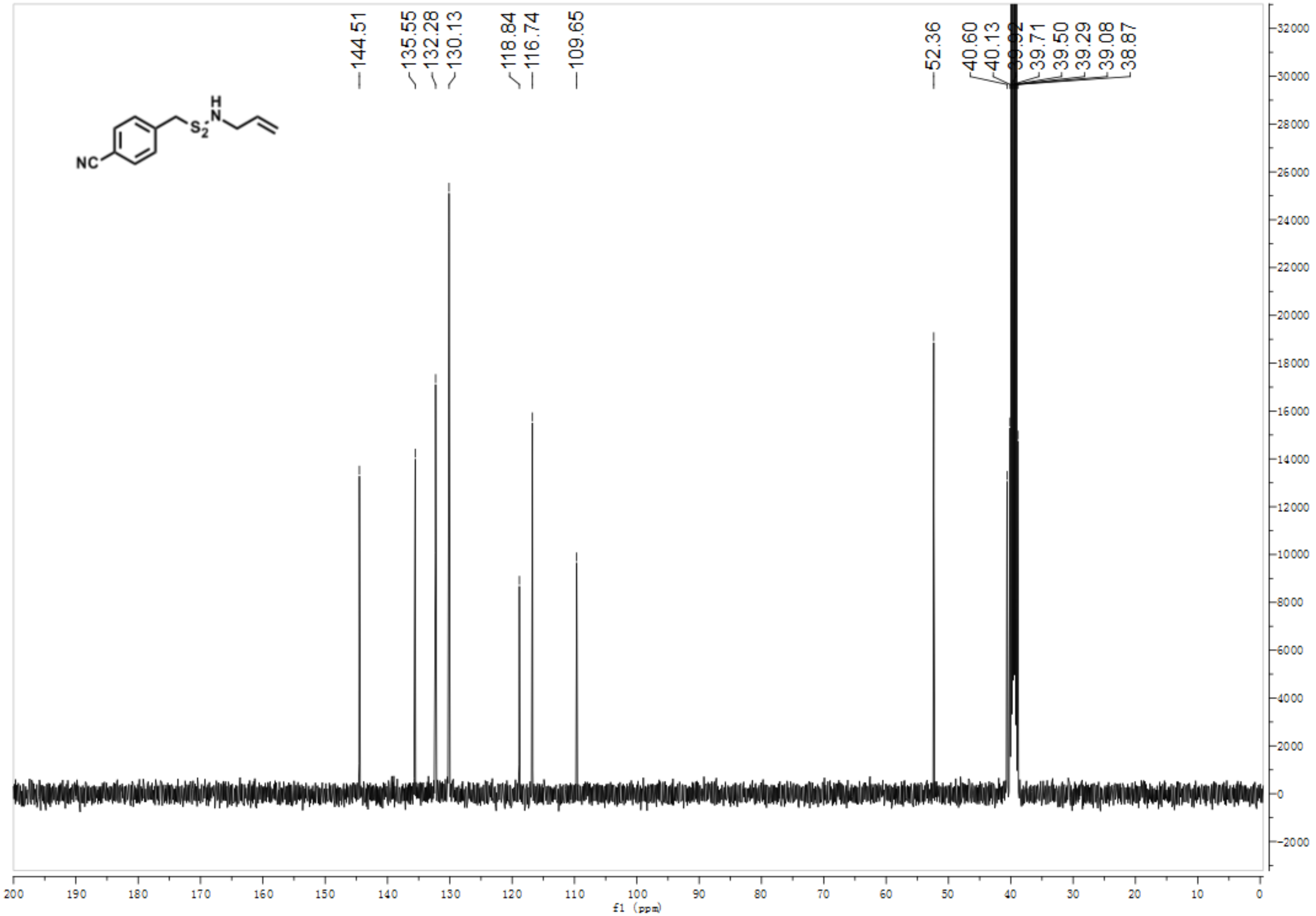

Supplementary Figure 145. ^{13}C NMR spectra for Compound **6i**.

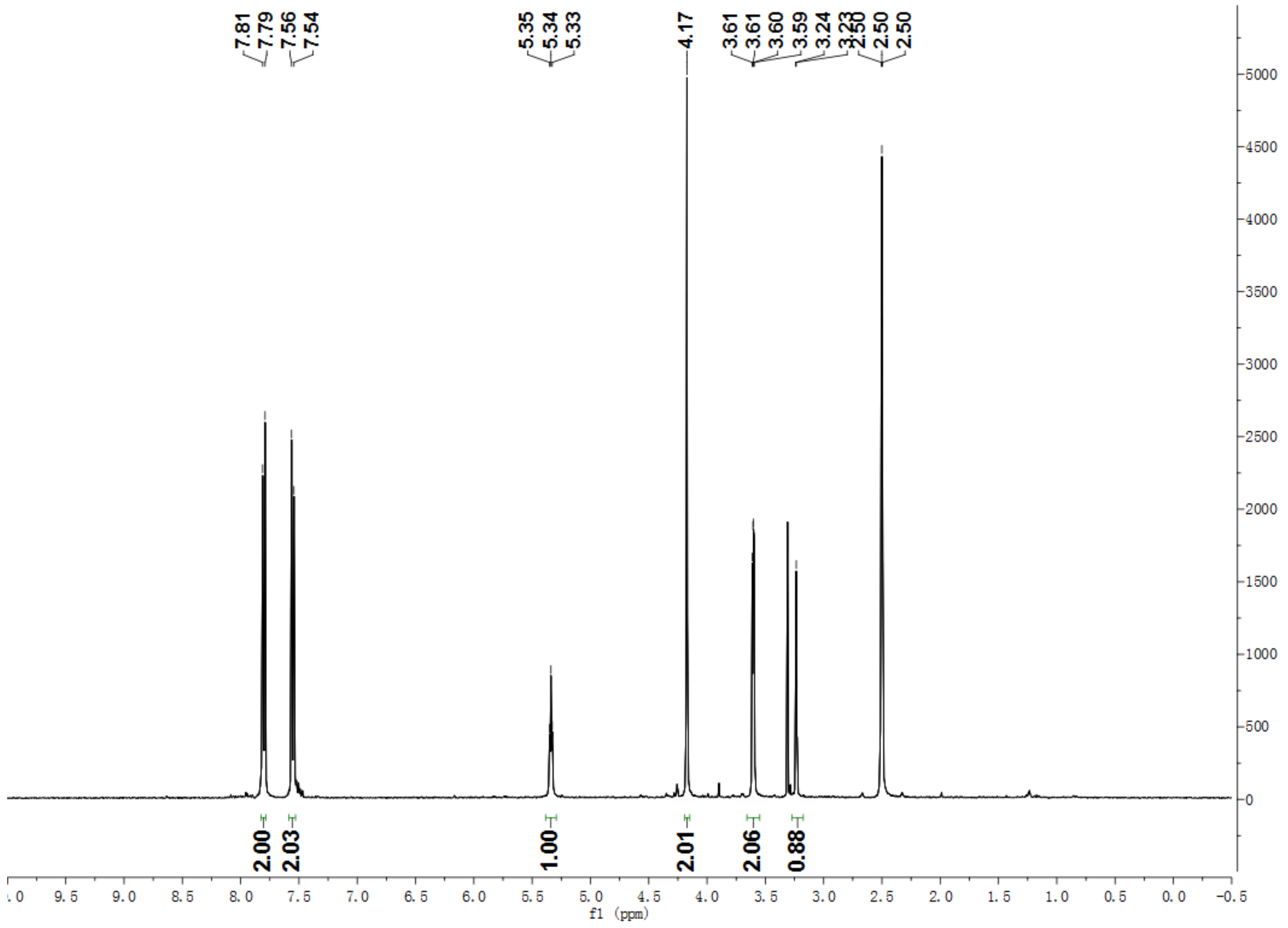

Supplementary Figure 146. ^{1}H NMR spectra for Compound **6j**.

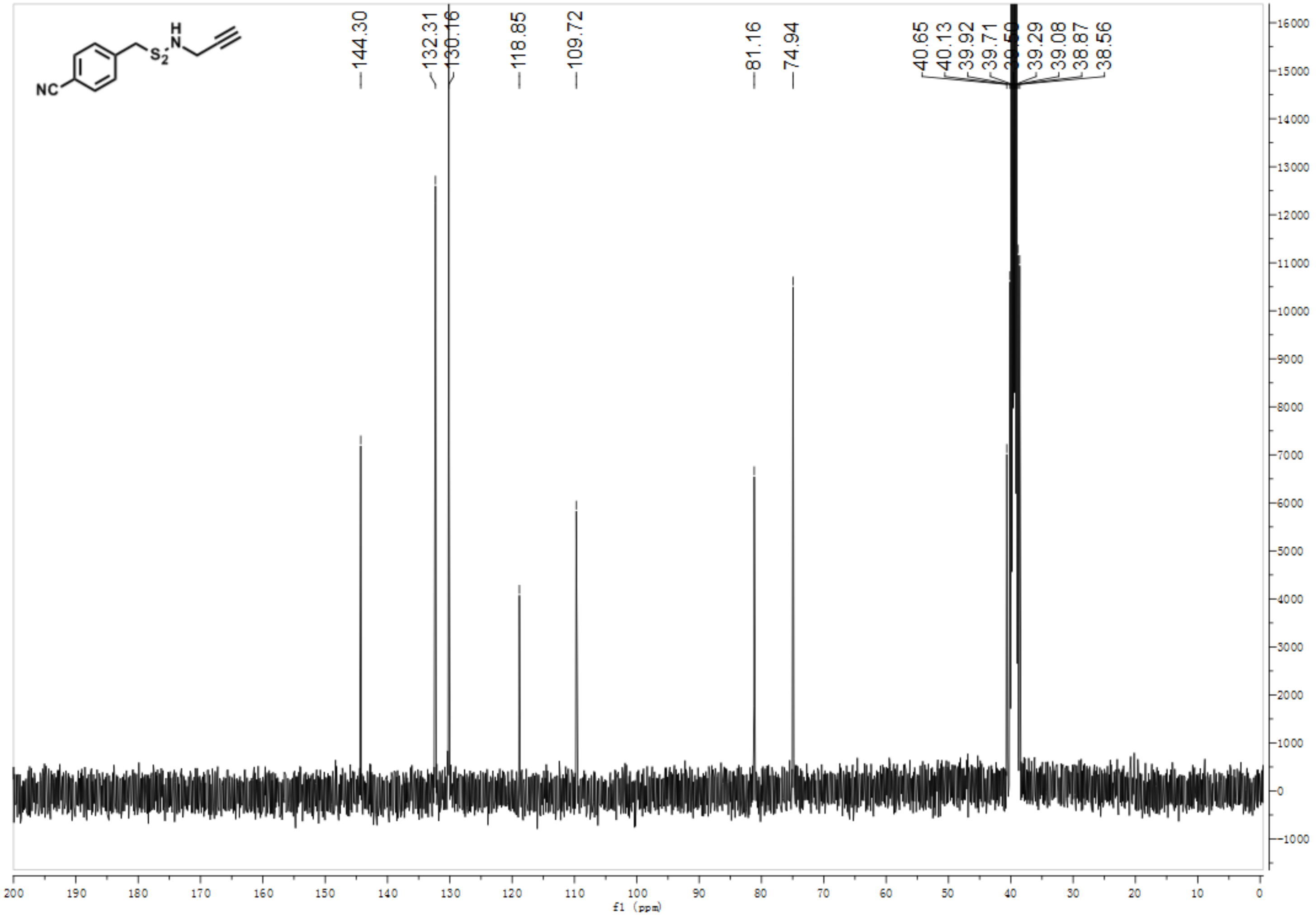

Supplementary Figure 147. ^{13}C NMR spectra for Compound **6j**.

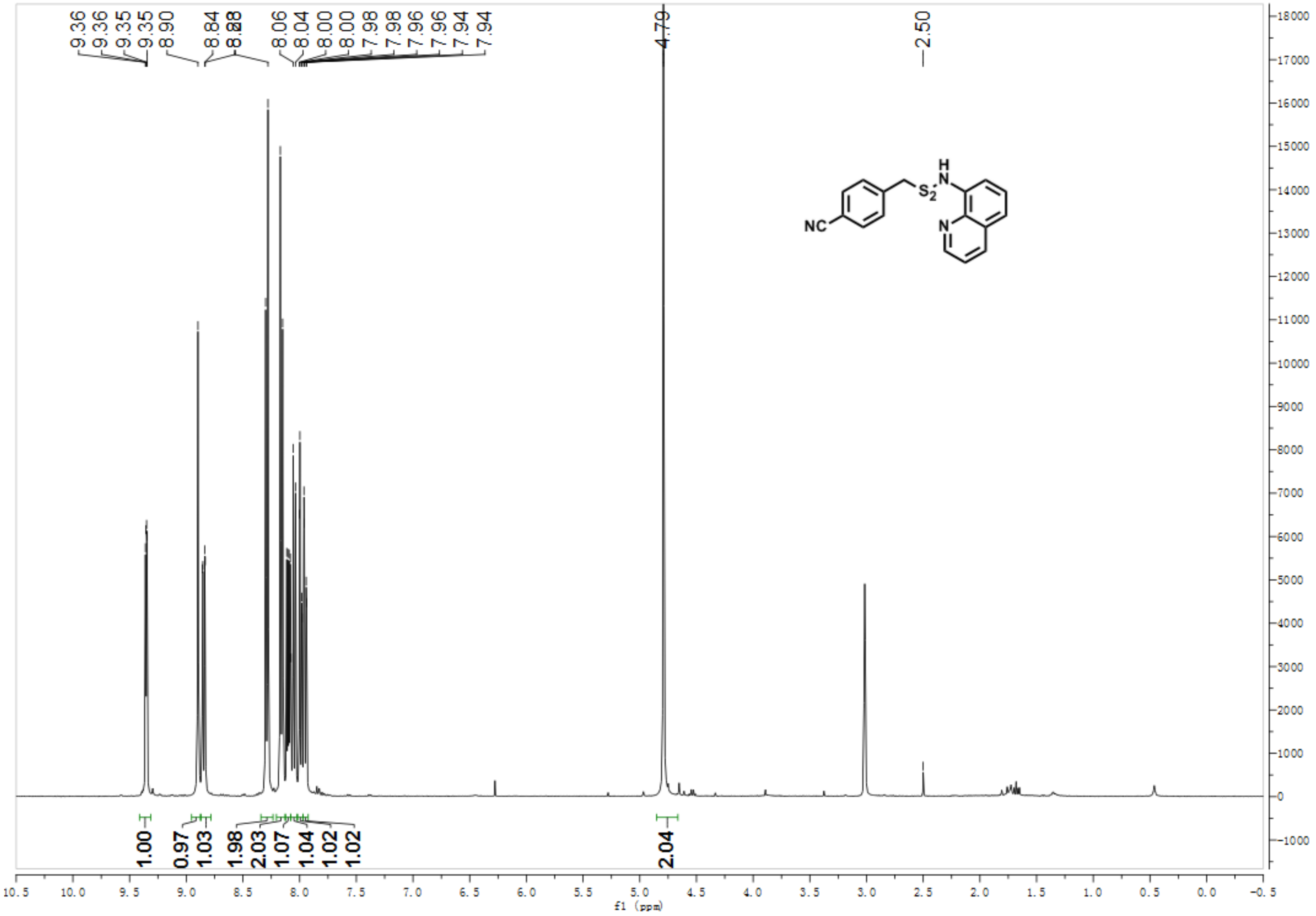

Supplementary Figure 148. ^{1}H NMR spectra for Compound **6k**.

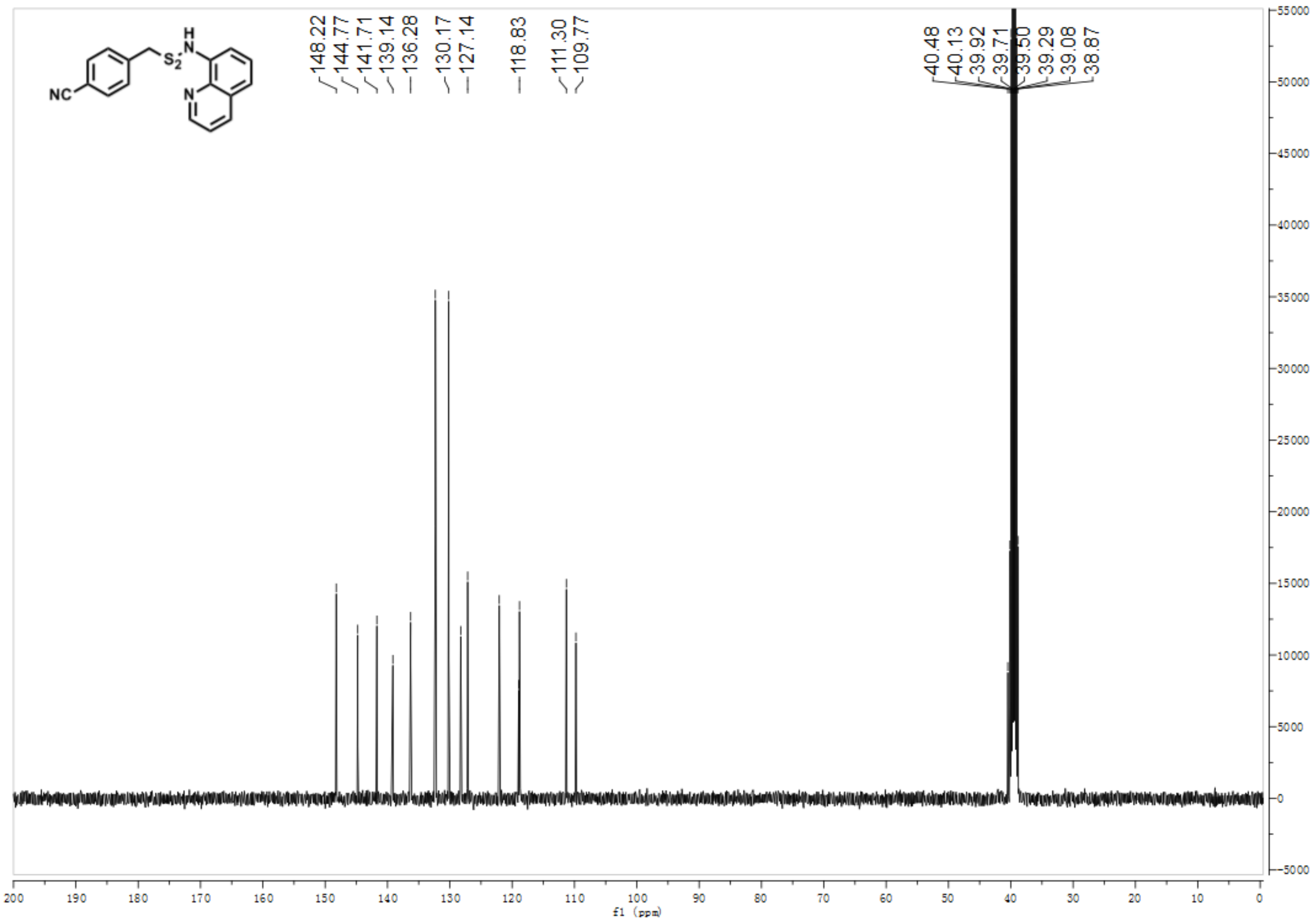

Supplementary Figure 149. ^{13}C NMR spectra for Compound **6k**.

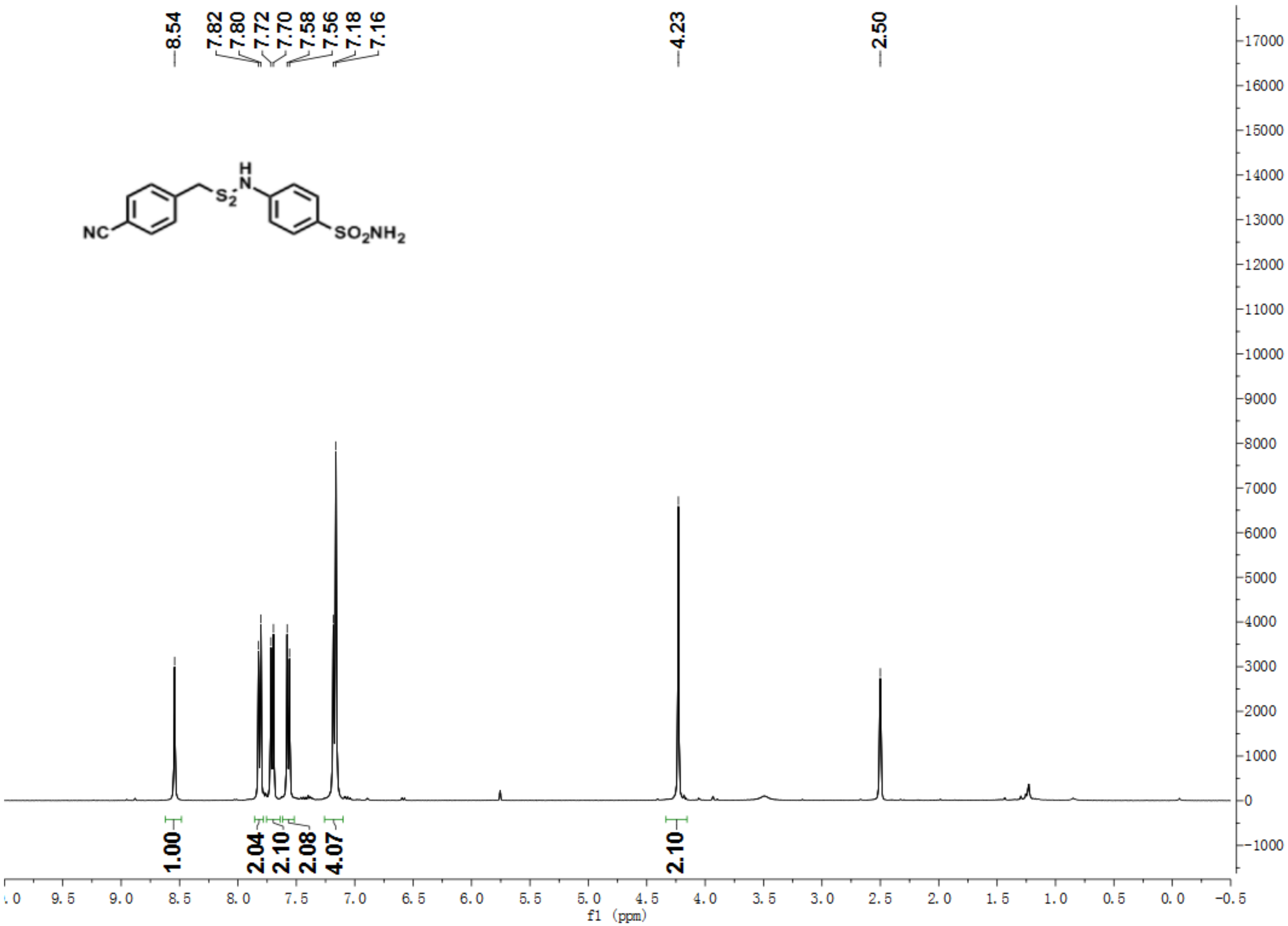

Supplementary Figure 150. ^{1}H NMR spectra for Compound **6l**.

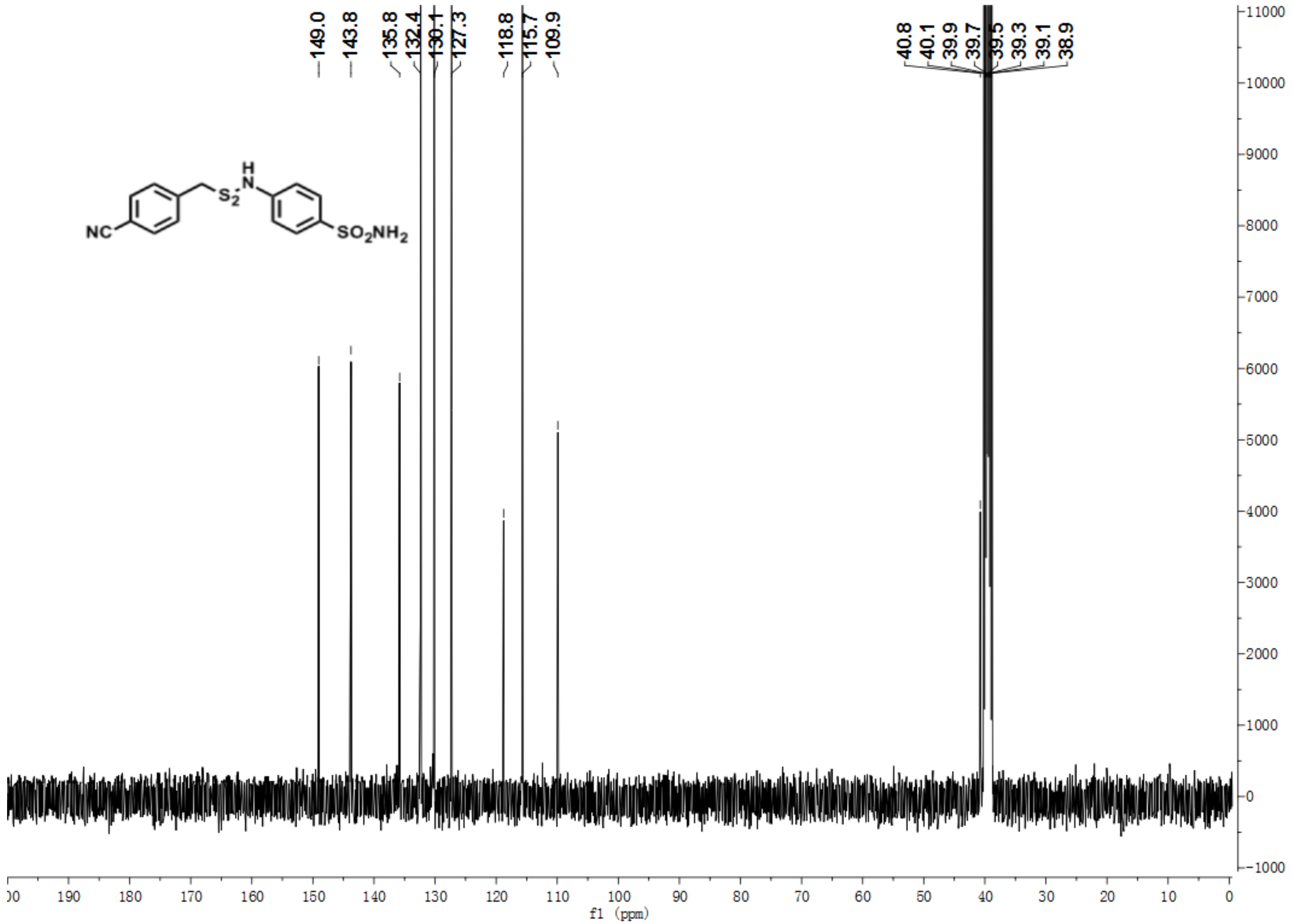

Supplementary Figure 151. ^{13}C NMR spectra for Compound **6l**.

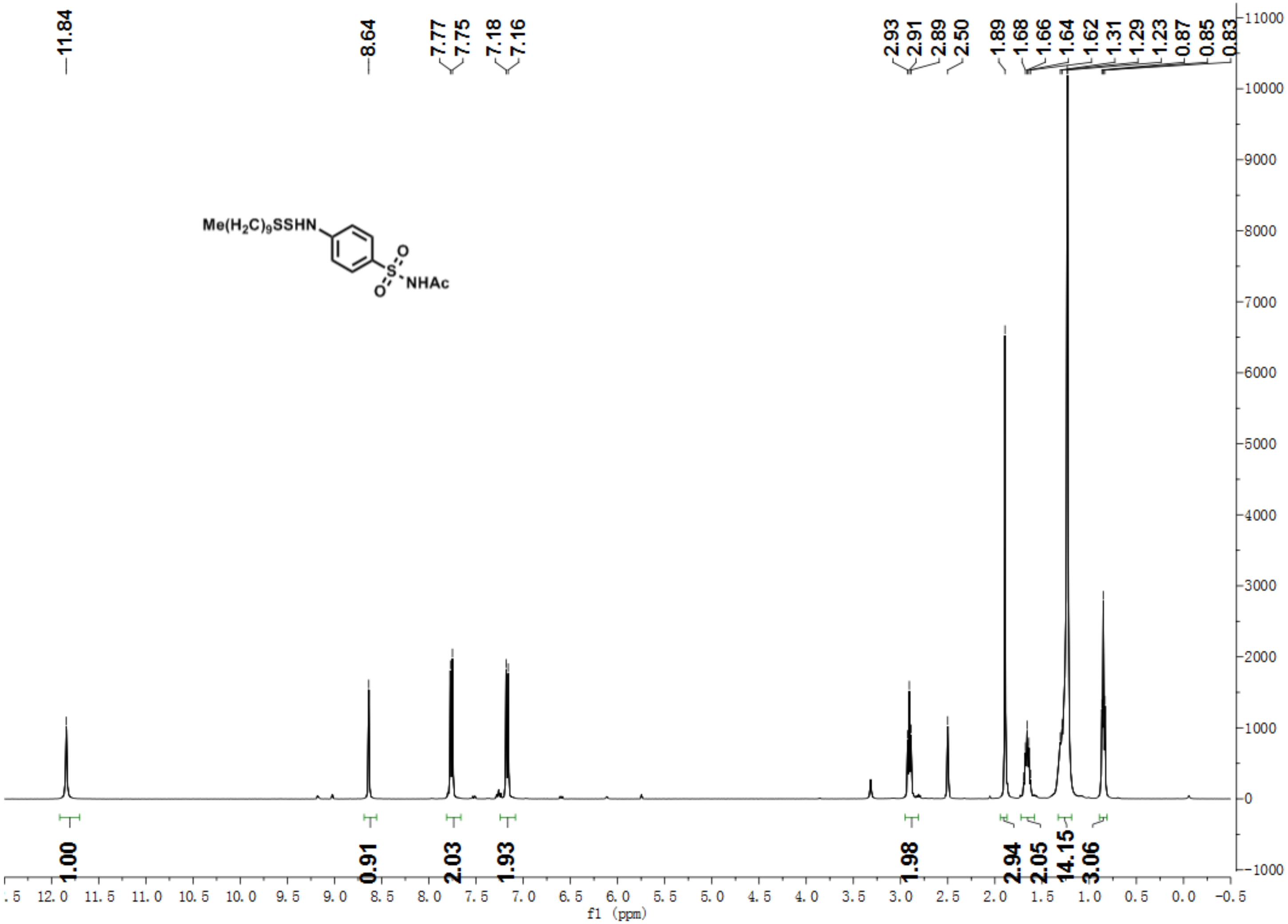

Supplementary Figure 152. ^{1}H NMR spectra for Compound **6m**.

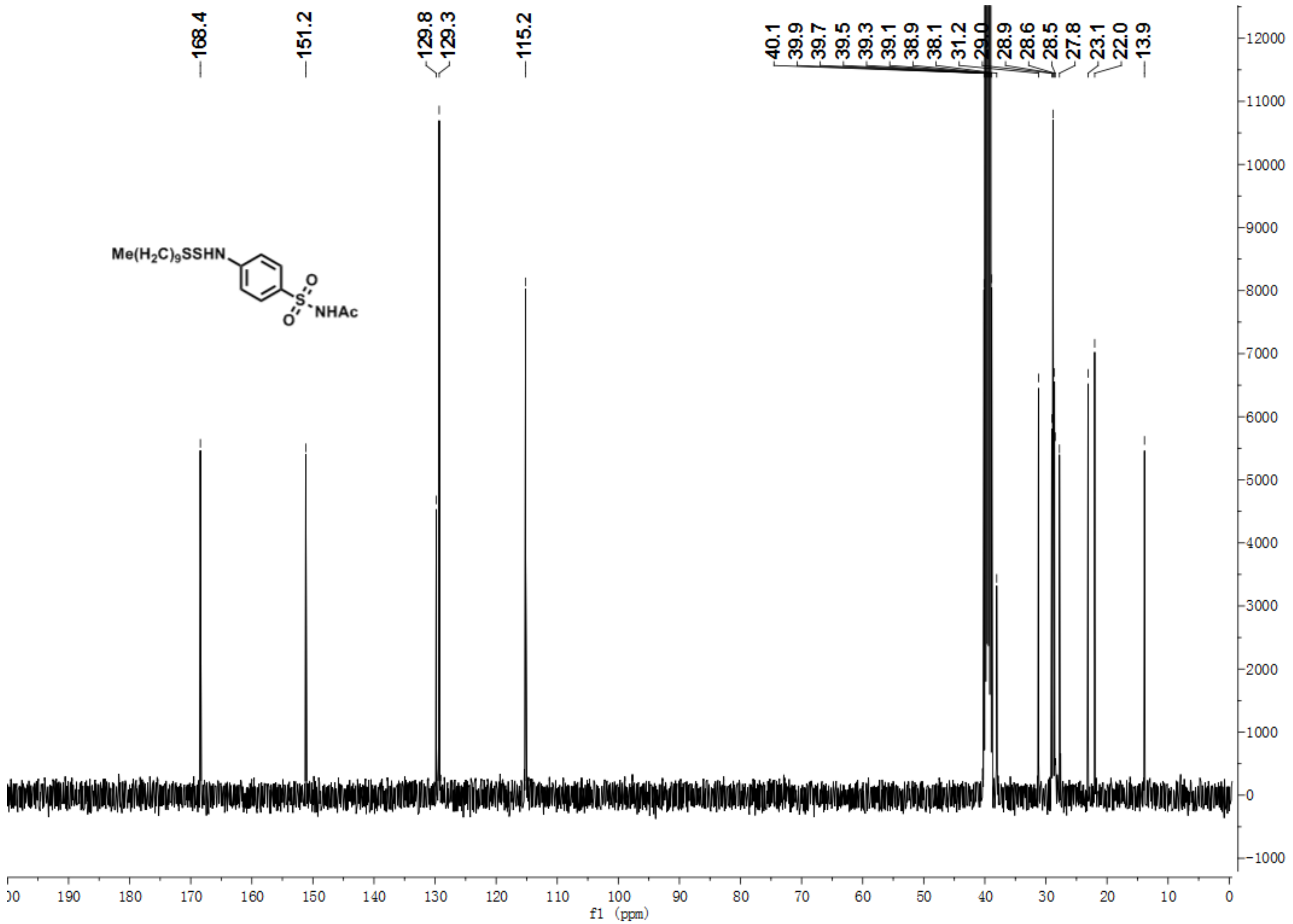

Supplementary Figure 153. ^{13}C NMR spectra for Compound **6m**.

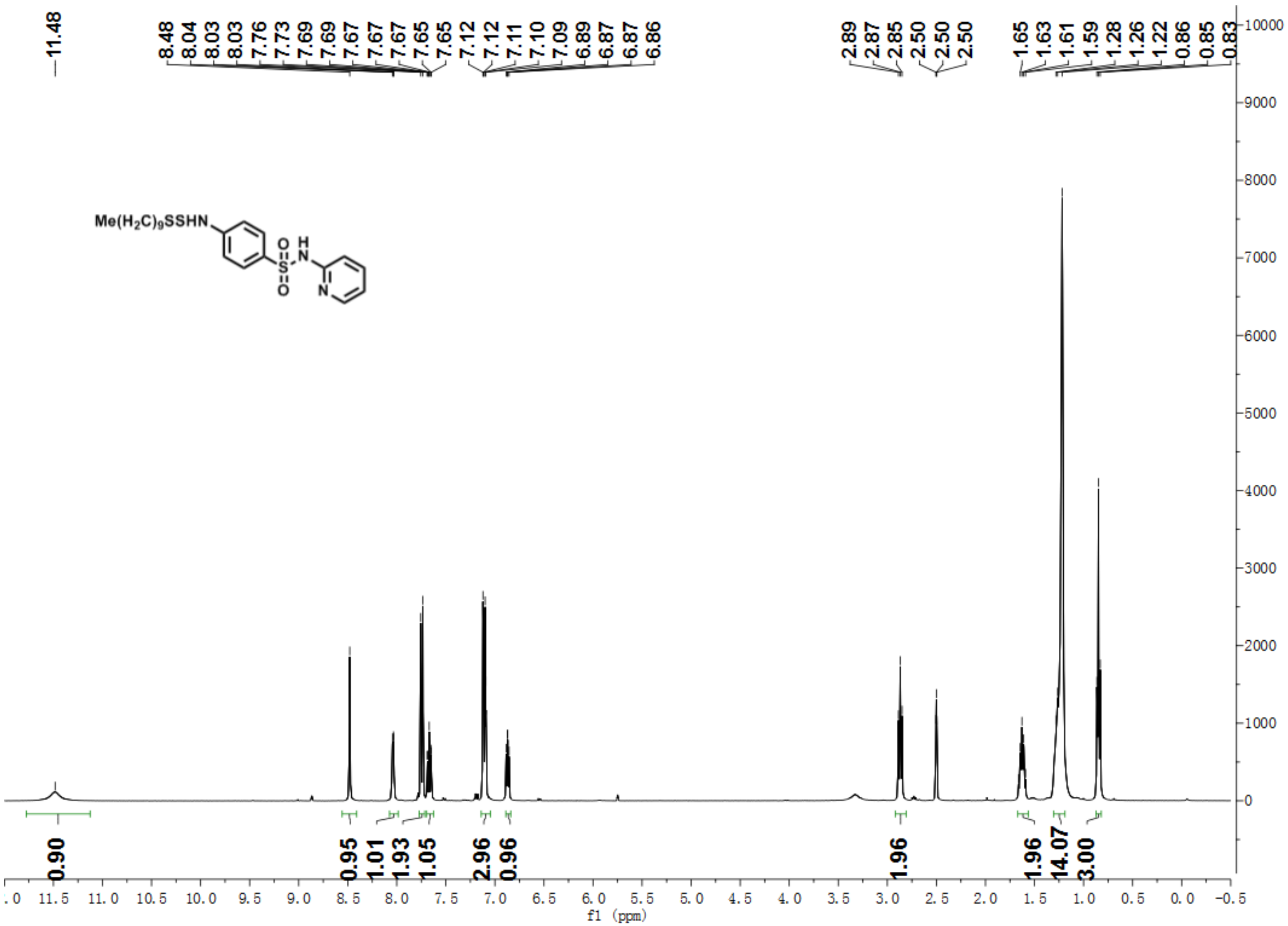

Supplementary Figure 154. ^{1}H NMR spectra for Compound **6n**.

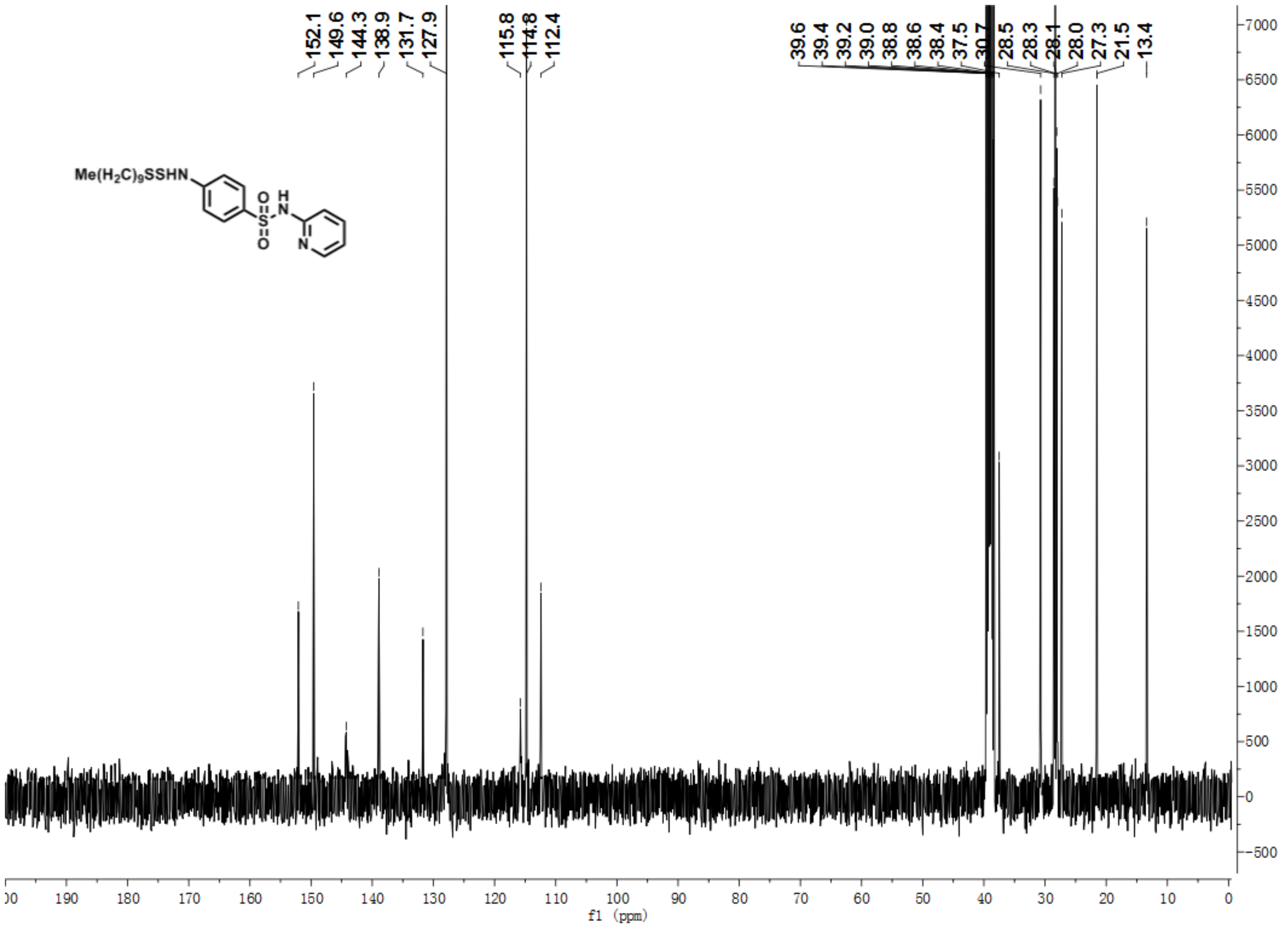

Supplementary Figure 155. ^{13}C NMR spectra for Compound **6n**.

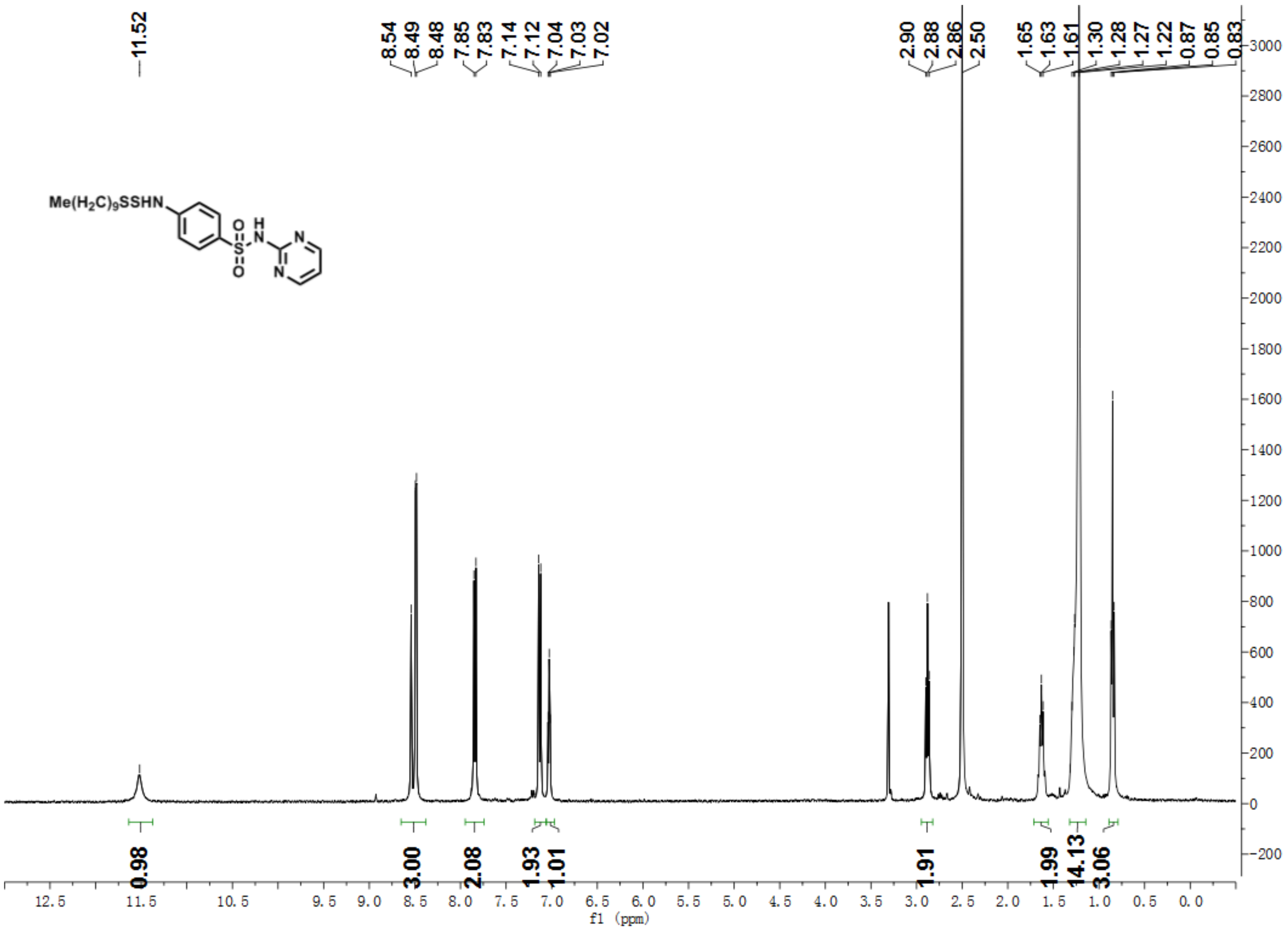

Supplementary Figure 156. ^{1}H NMR spectra for Compound **6o**.

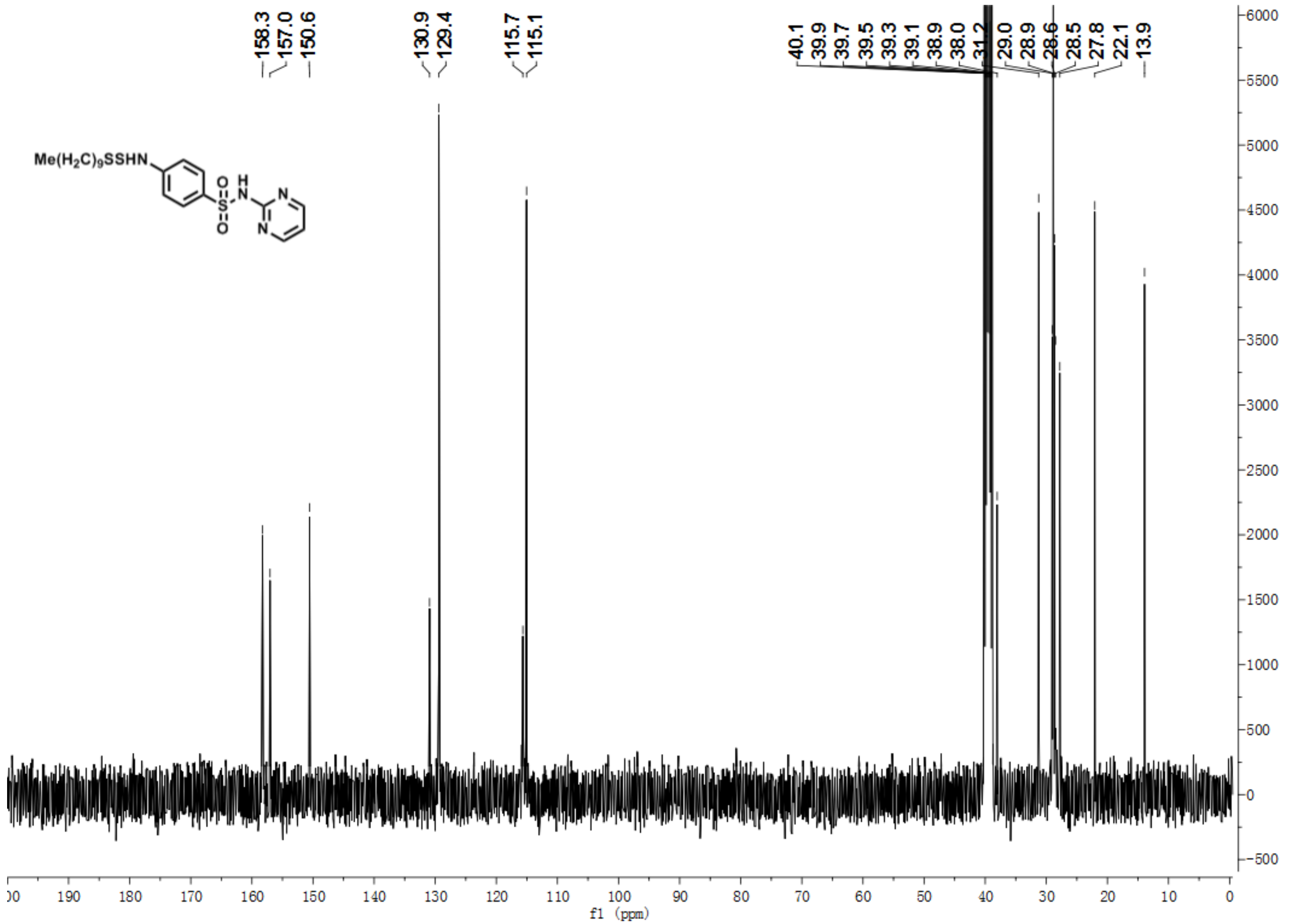

Supplementary Figure 157. ^{13}C NMR spectra for Compound **6o**.

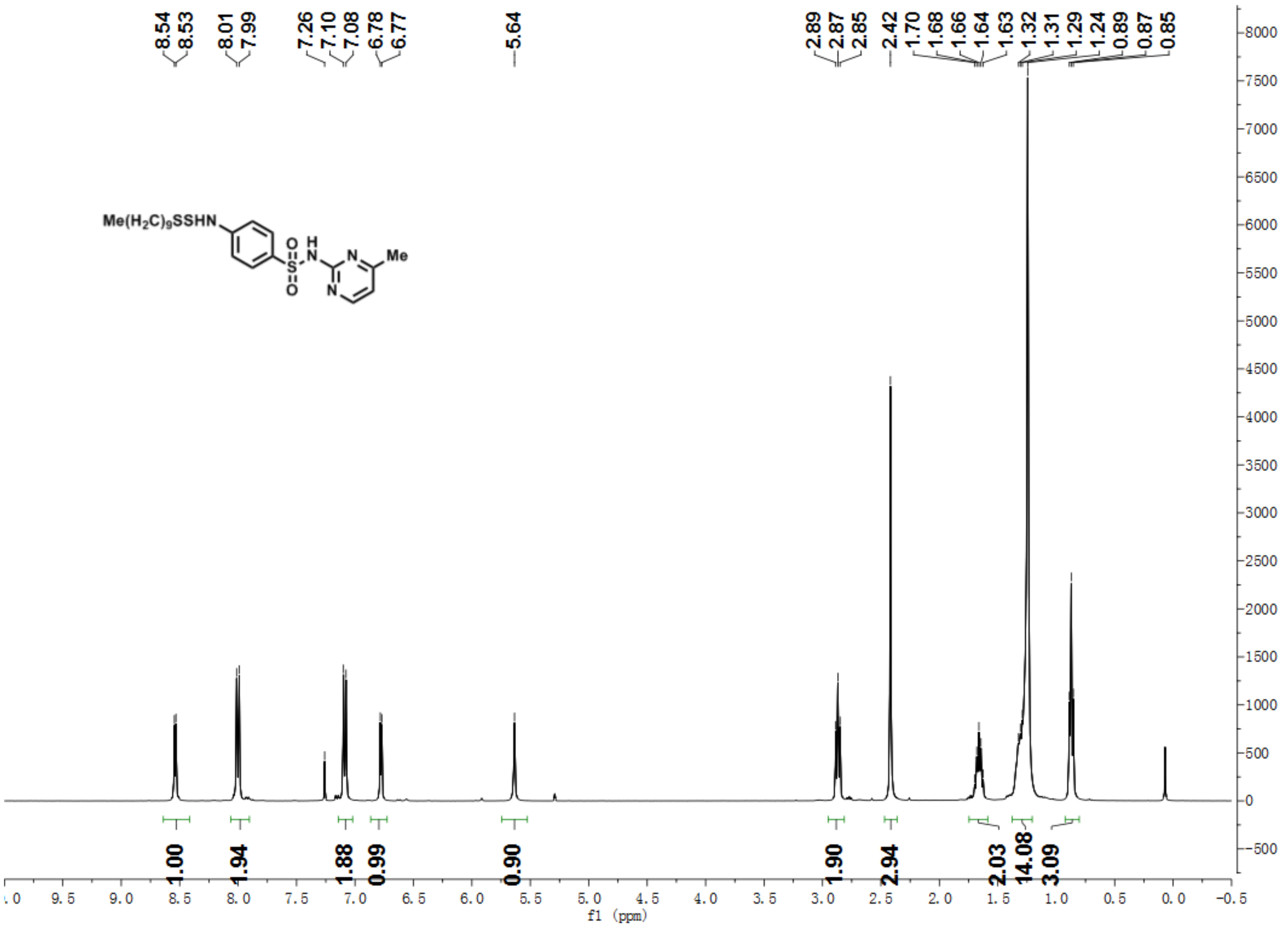

Supplementary Figure 158. ^{1}H NMR spectra for Compound **6p**.

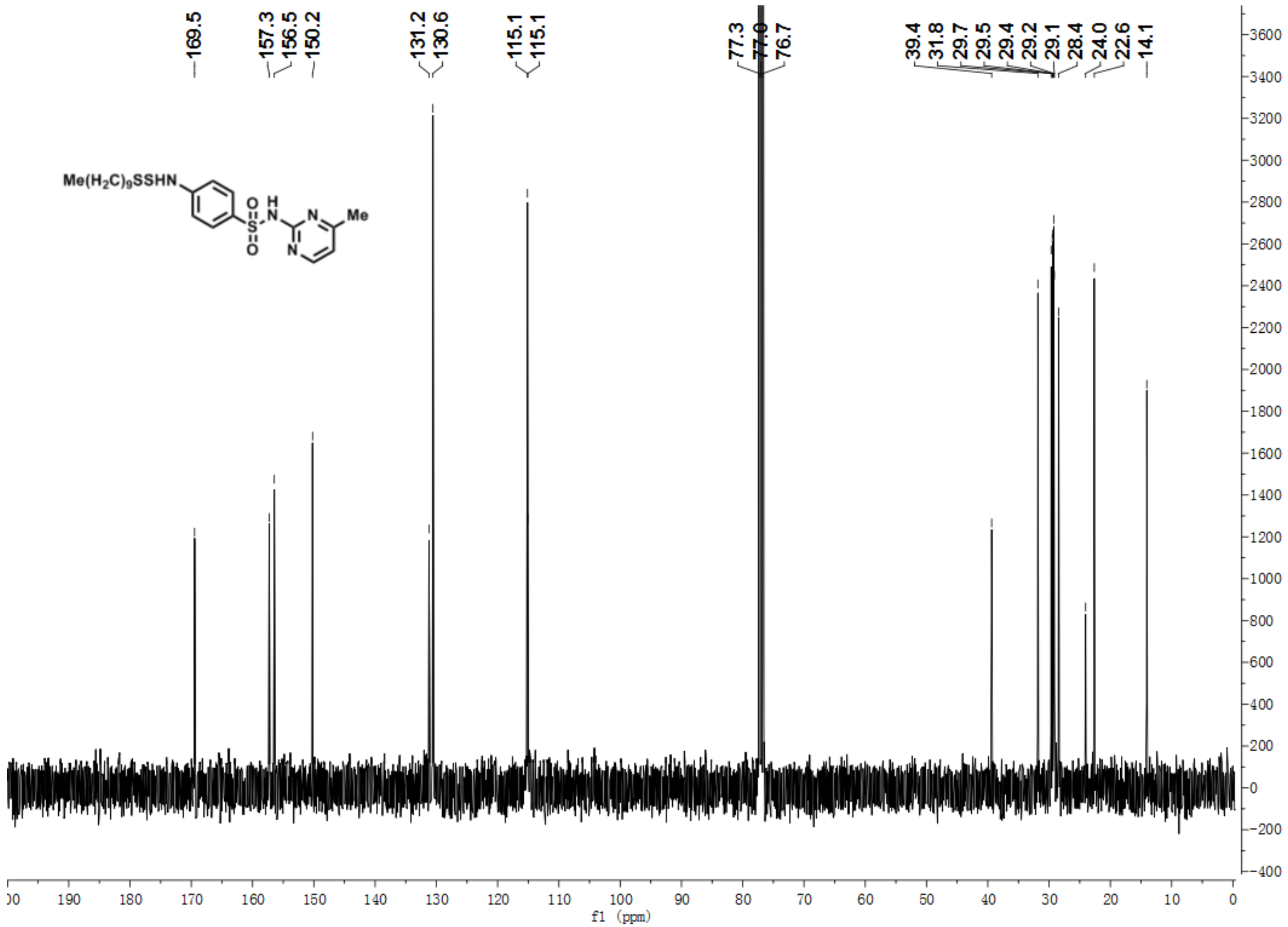

Supplementary Figure 159. ^{13}C NMR spectra for Compound **6p**.

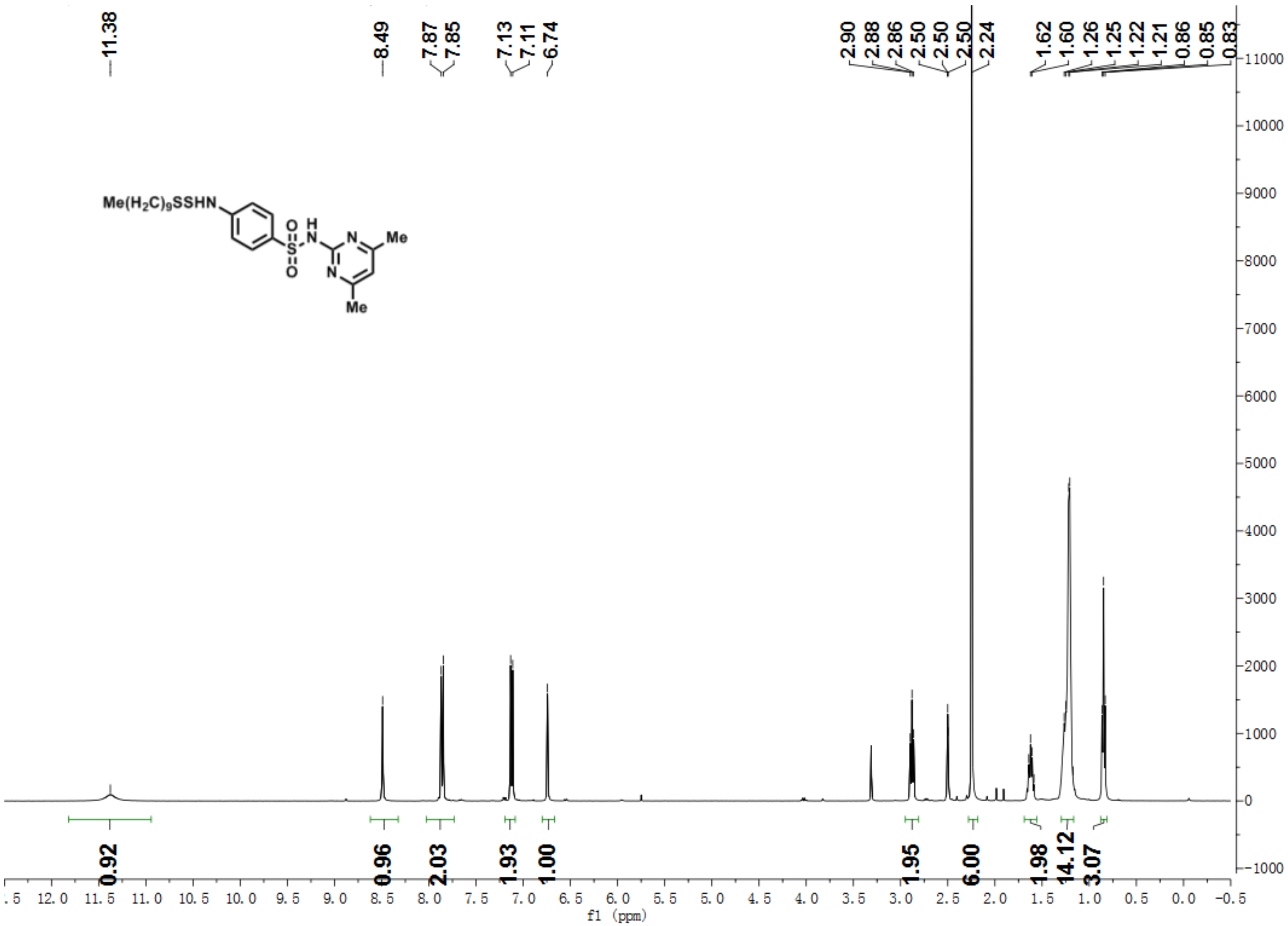

Supplementary Figure 160. ^{1}H NMR spectra for Compound **6q**.

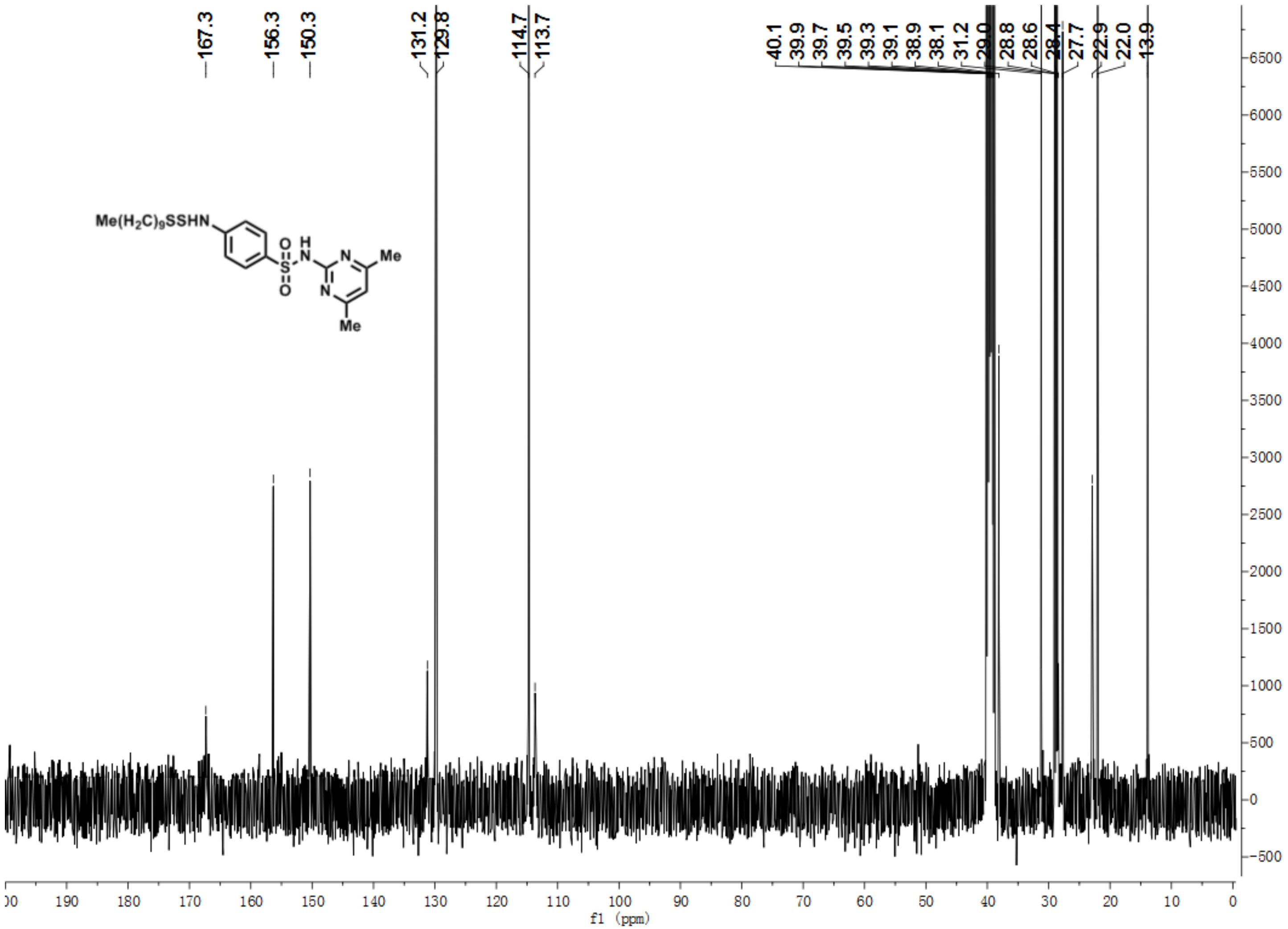

Supplementary Figure 161. ^{13}C NMR spectra for Compound **6q**.

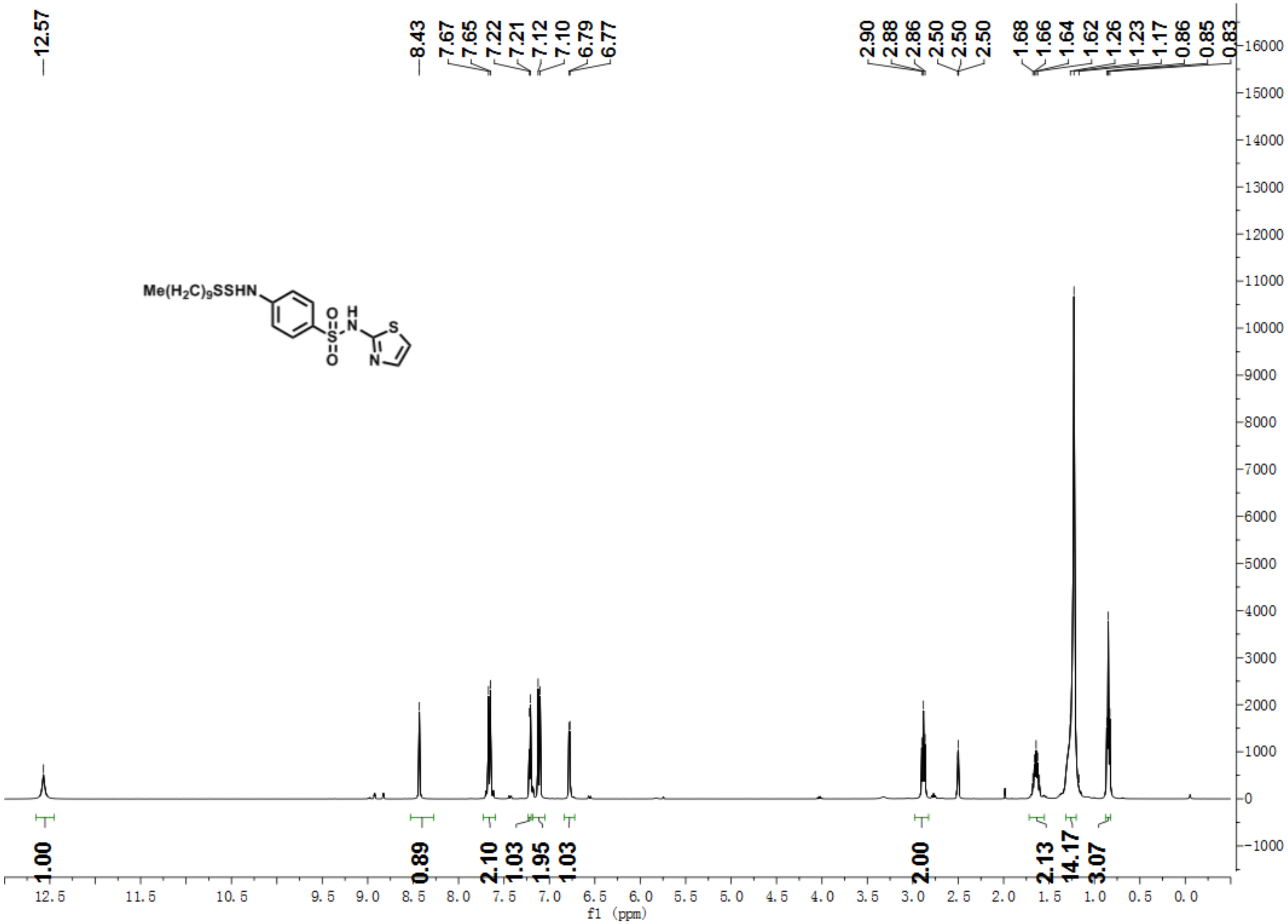

Supplementary Figure 162. ^{1}H NMR spectra for Compound **6r**.

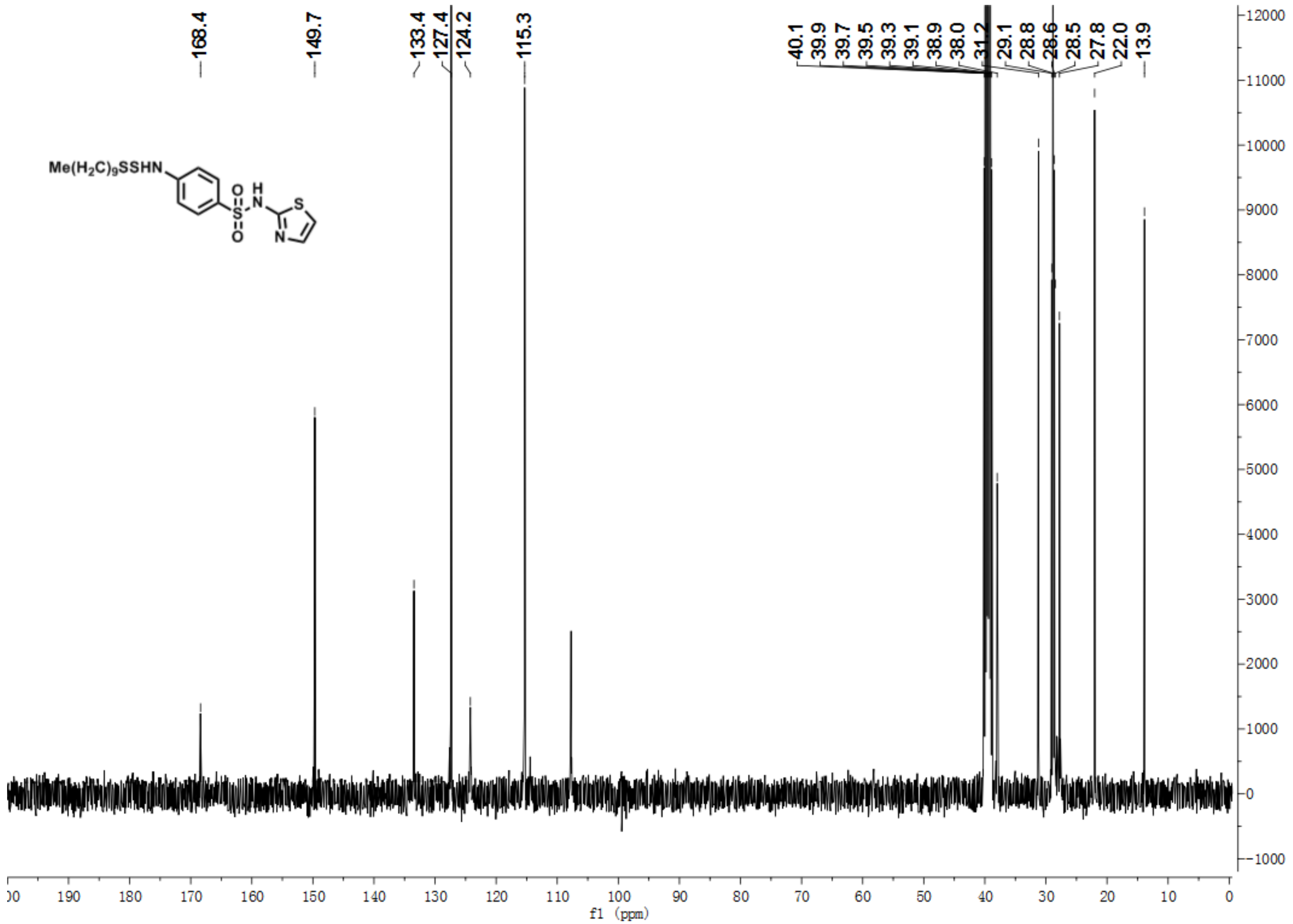

Supplementary Figure 163. ^{13}C NMR spectra for Compound **6r**.

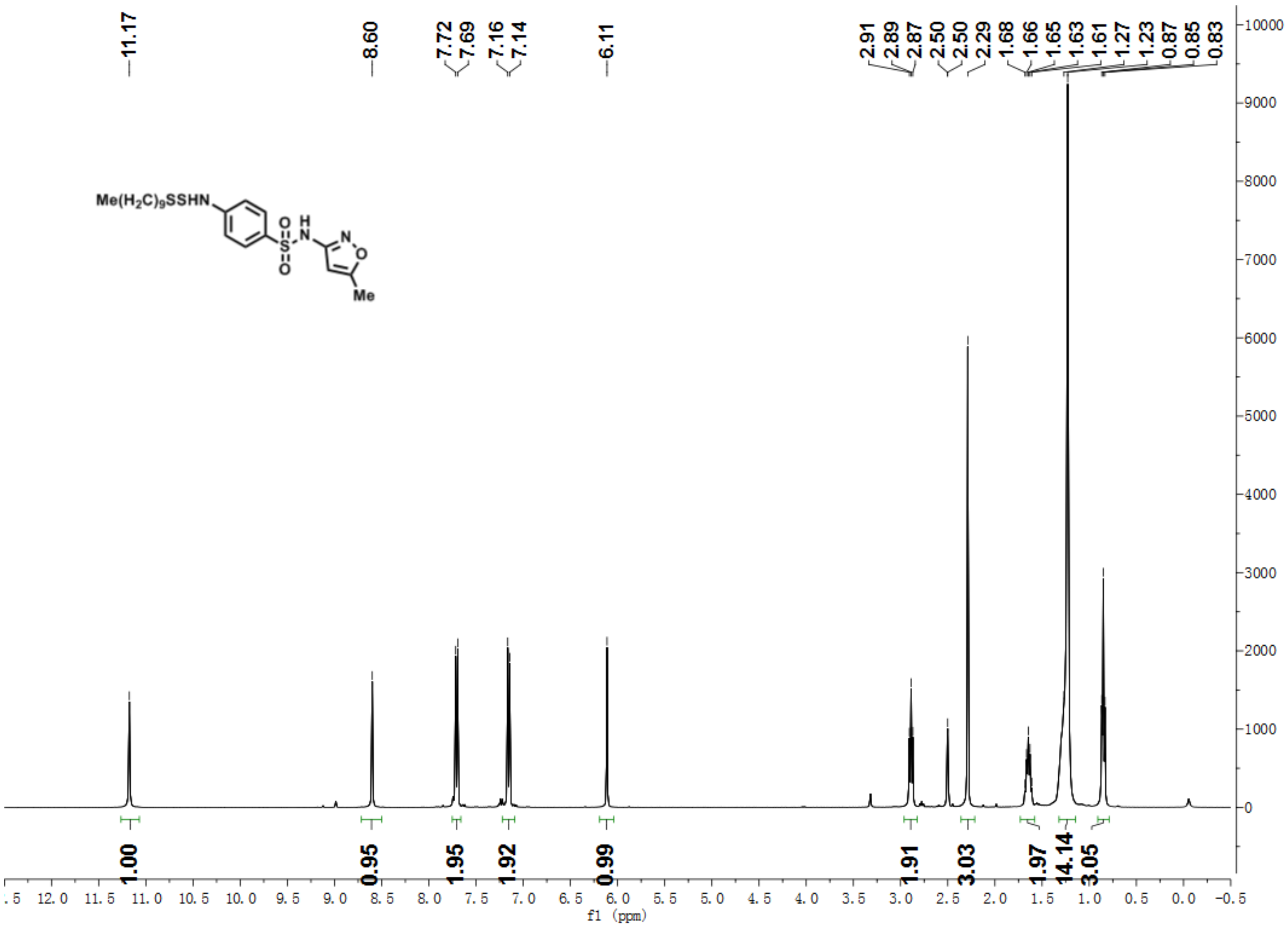

Supplementary Figure 164. ^{1}H NMR spectra for Compound **6s**.

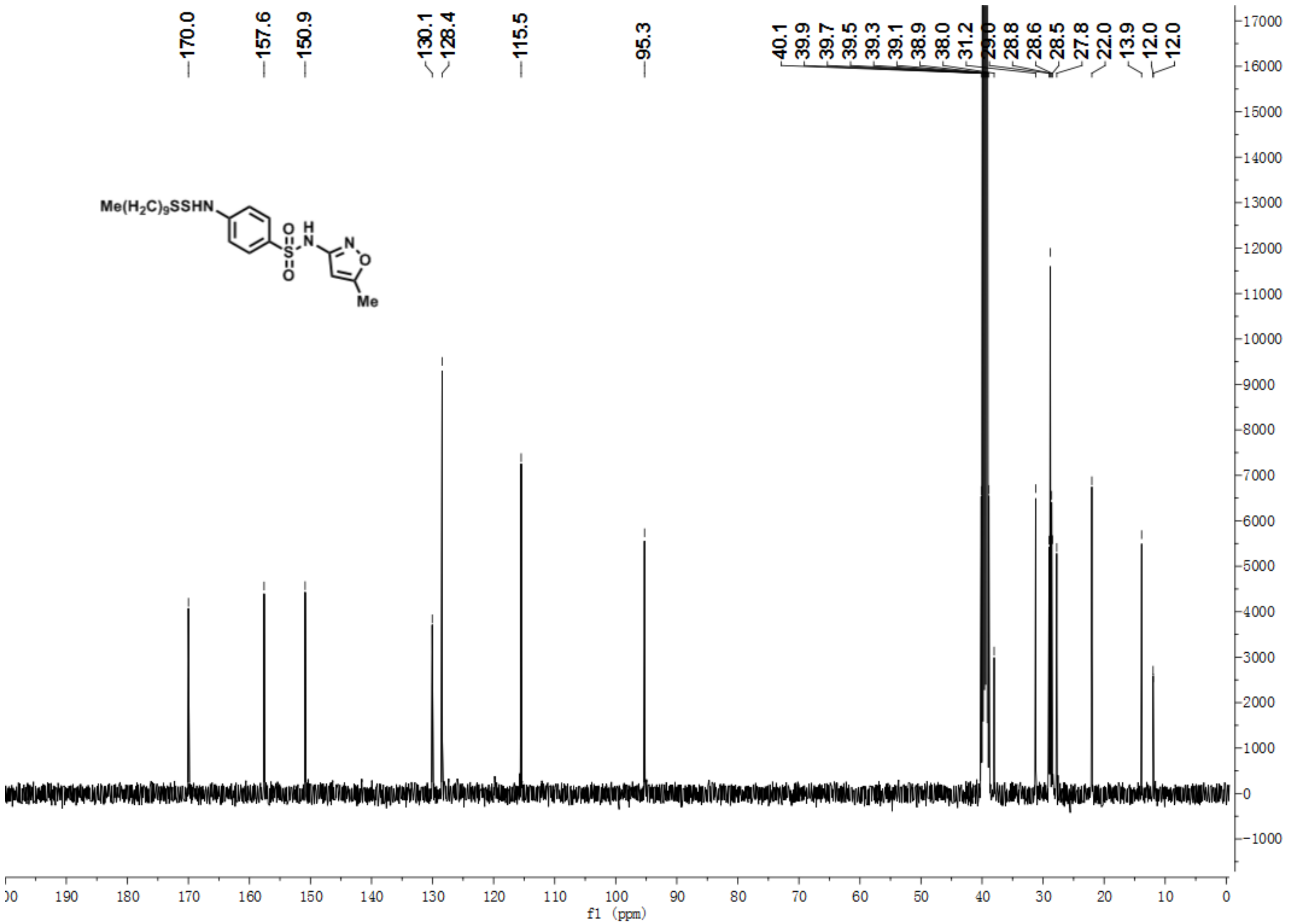

Supplementary Figure 165. ^{13}C NMR spectra for Compound **6s**.

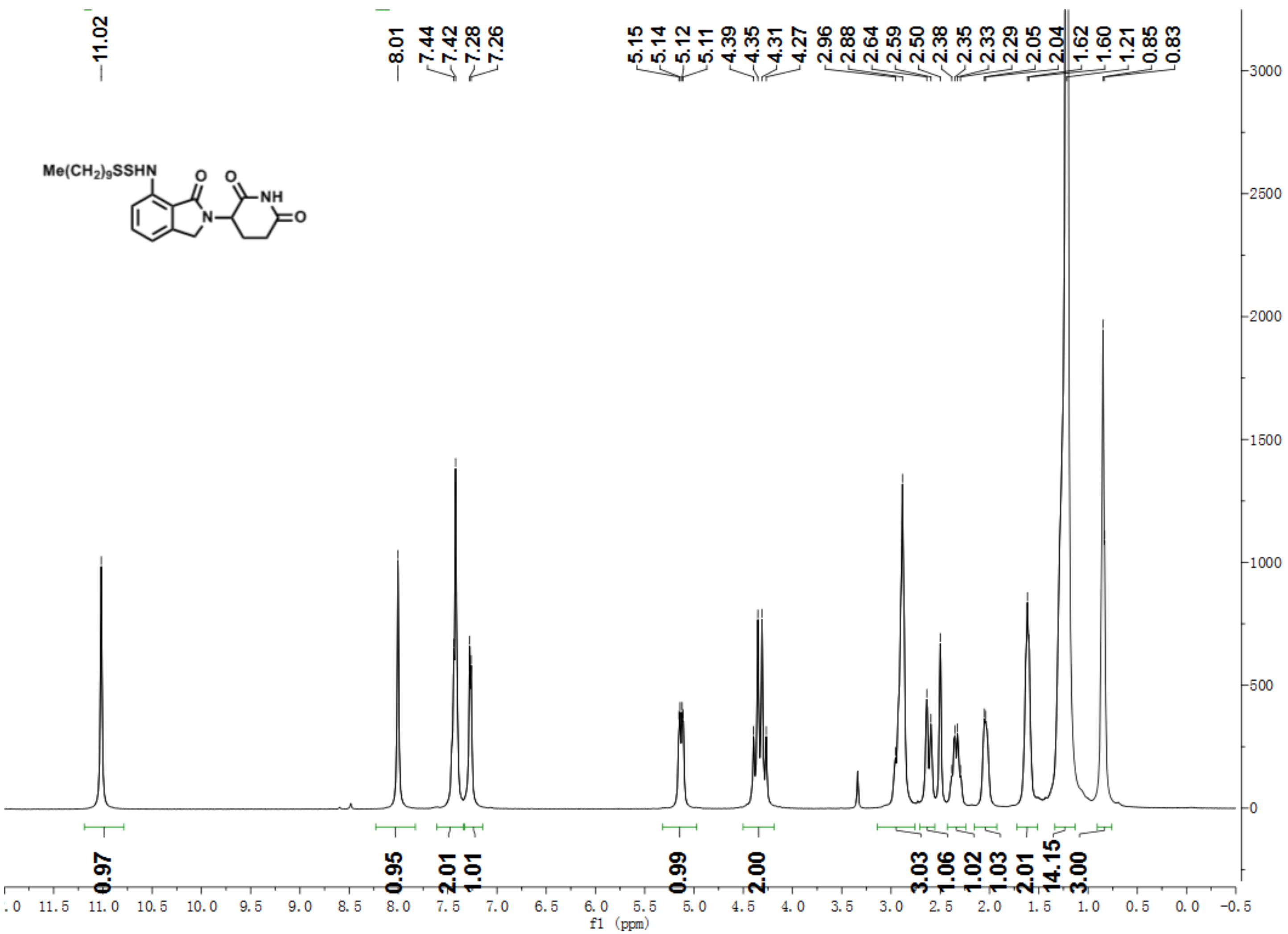

Supplementary Figure 166. ¹H NMR spectra for Compound **6t**.

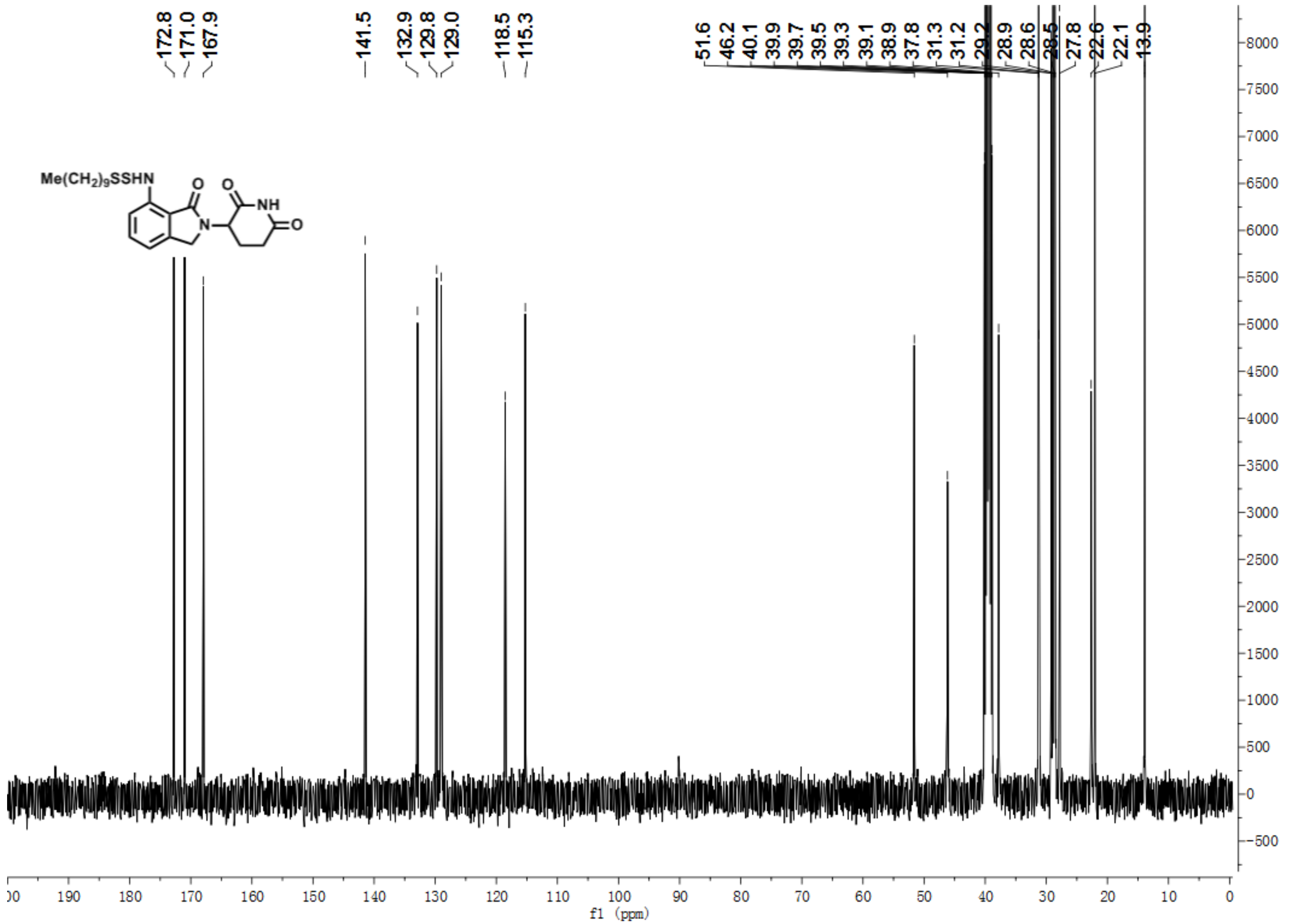

Supplementary Figure 167. ^{13}C NMR spectra for Compound **6t**.

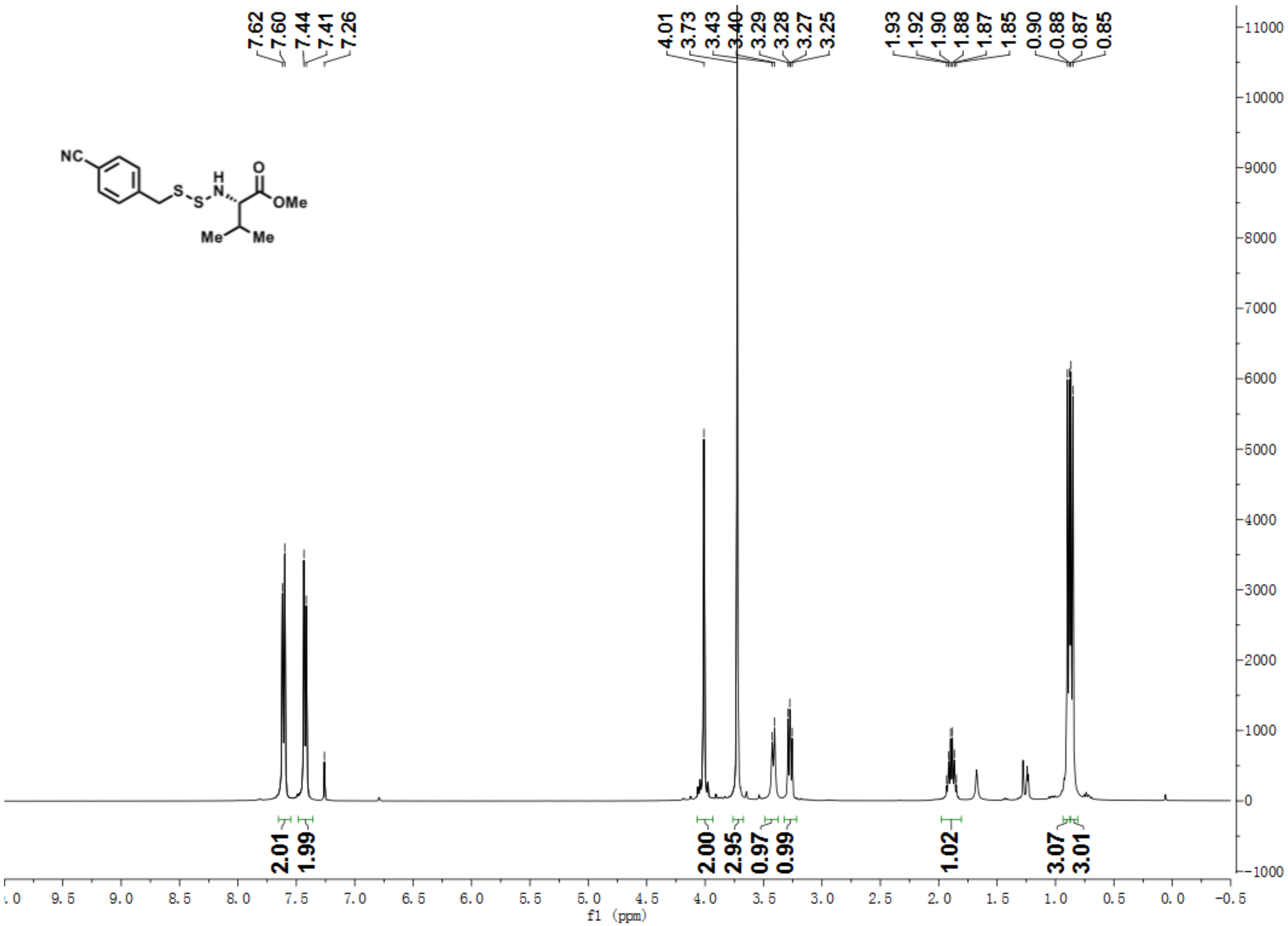

Supplementary Figure 168. ^{1}H NMR spectra for Compound **6u**.

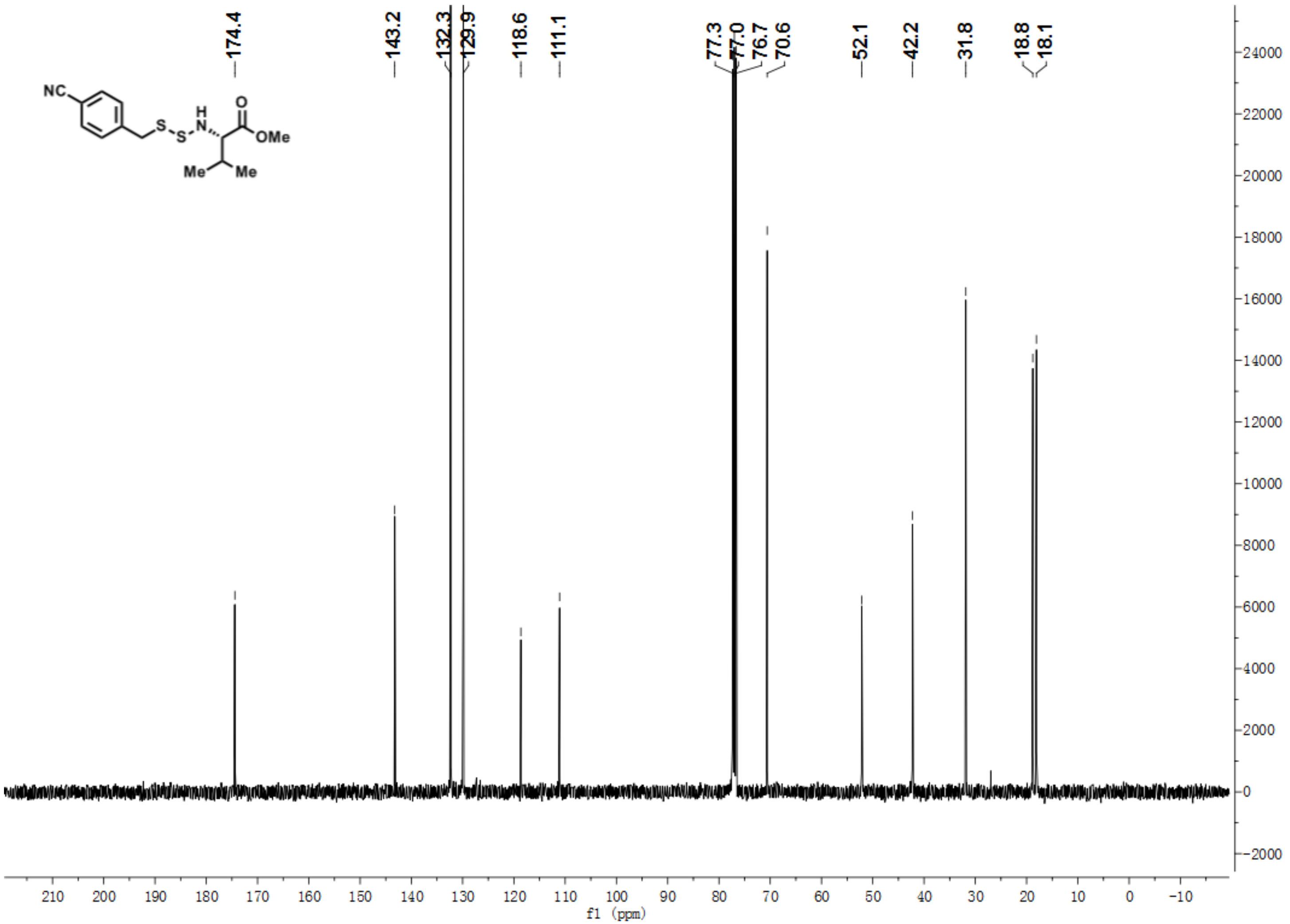

Supplementary Figure 169. ^{13}C NMR spectra for Compound **6u**.

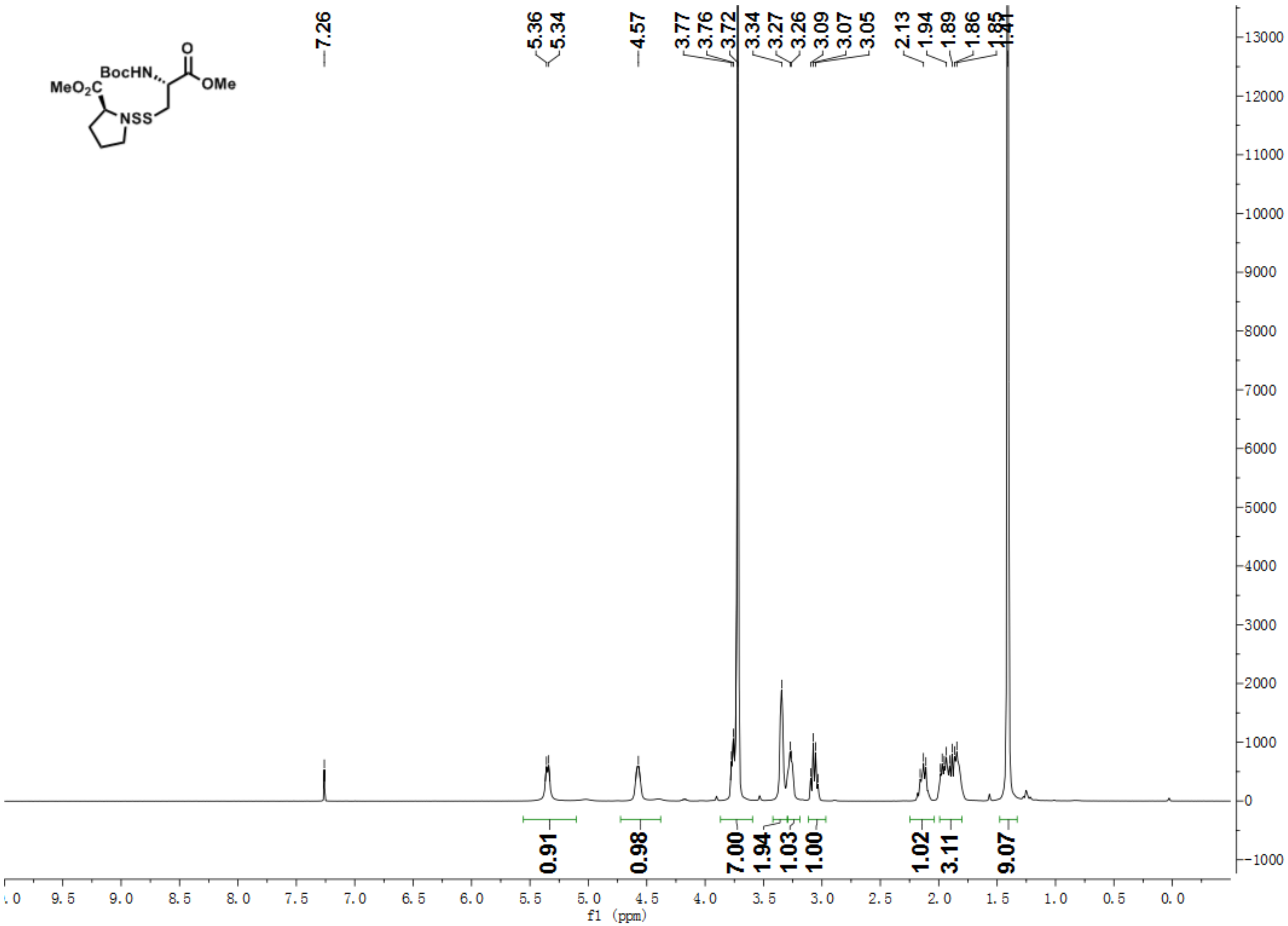

Supplementary Figure 170. ^{1}H NMR spectra for Compound **6v**.

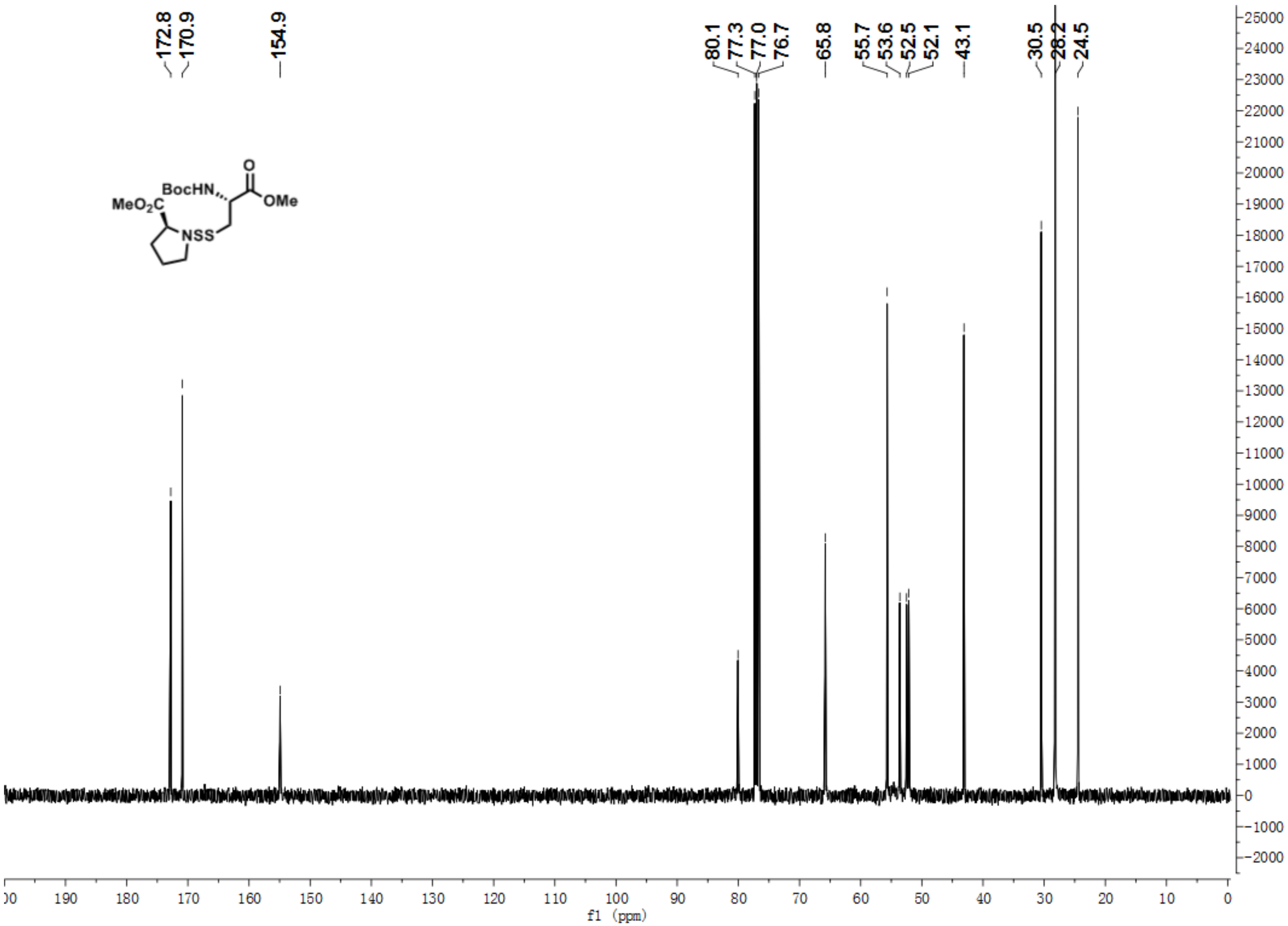

Supplementary Figure 171. ^{13}C NMR spectra for Compound **6v**.

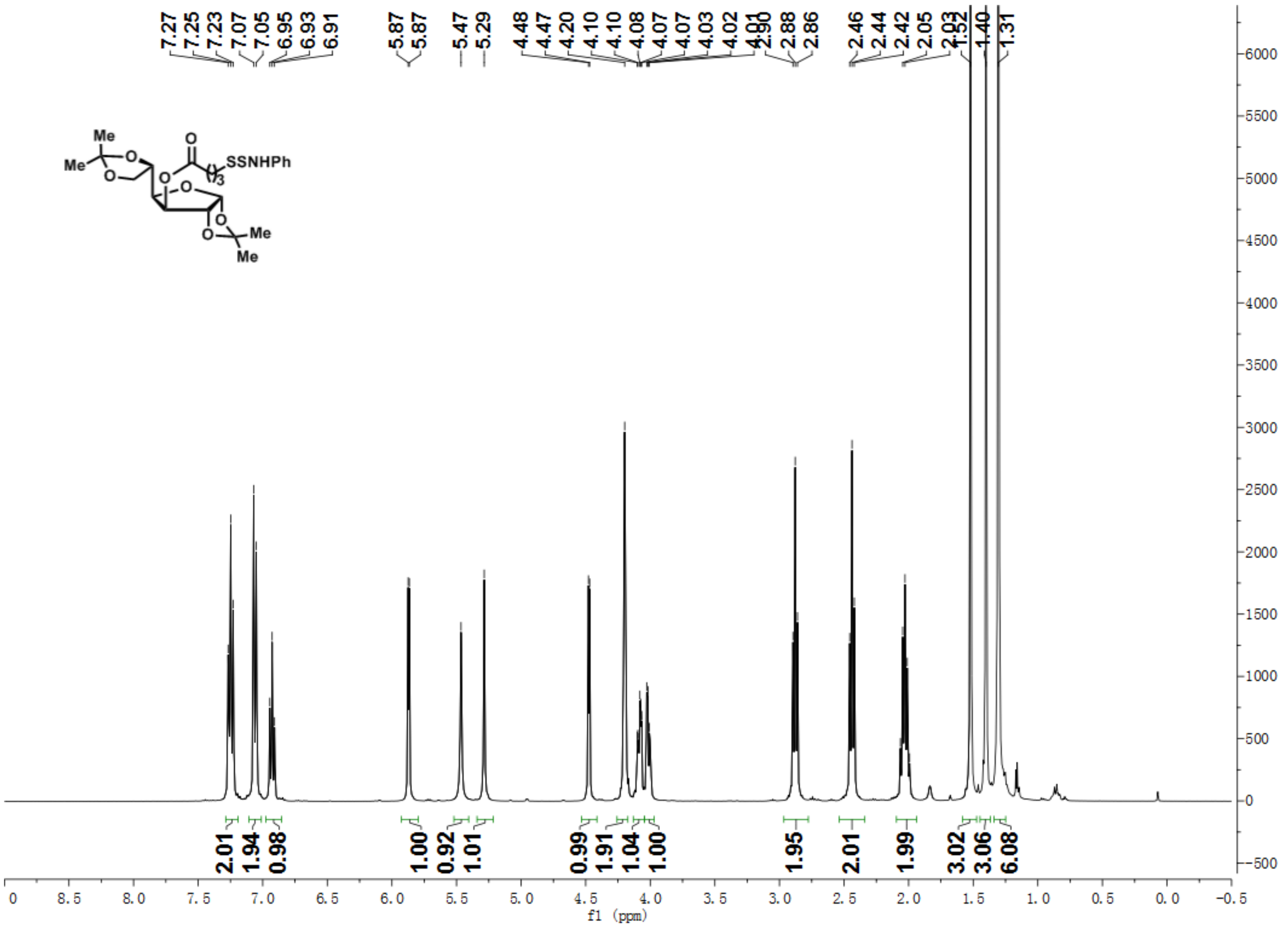

Supplementary Figure 172. ^{1}H NMR spectra for Compound **6w**.

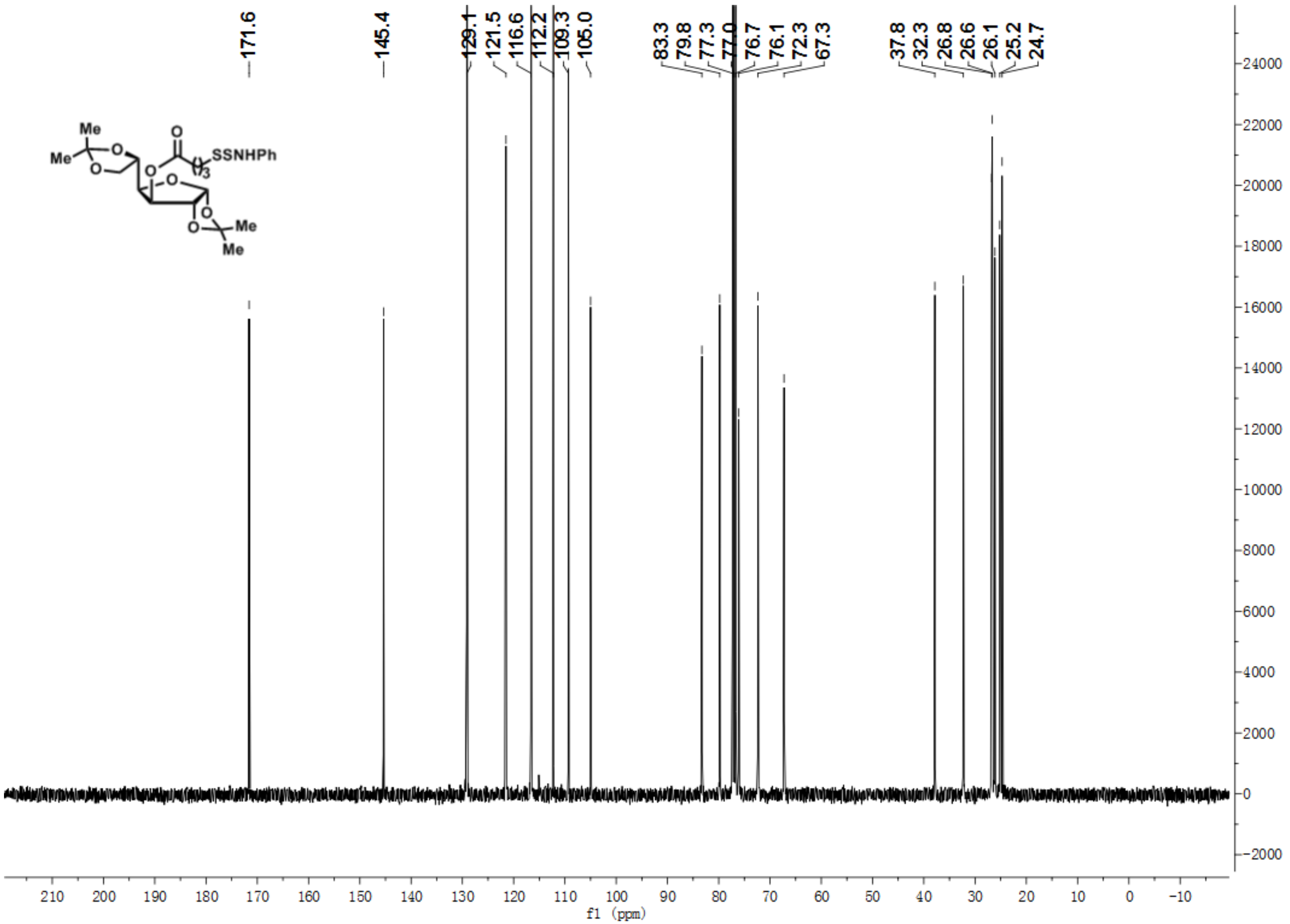

Supplementary Figure 173. ^{13}C NMR spectra for Compound **6w**.

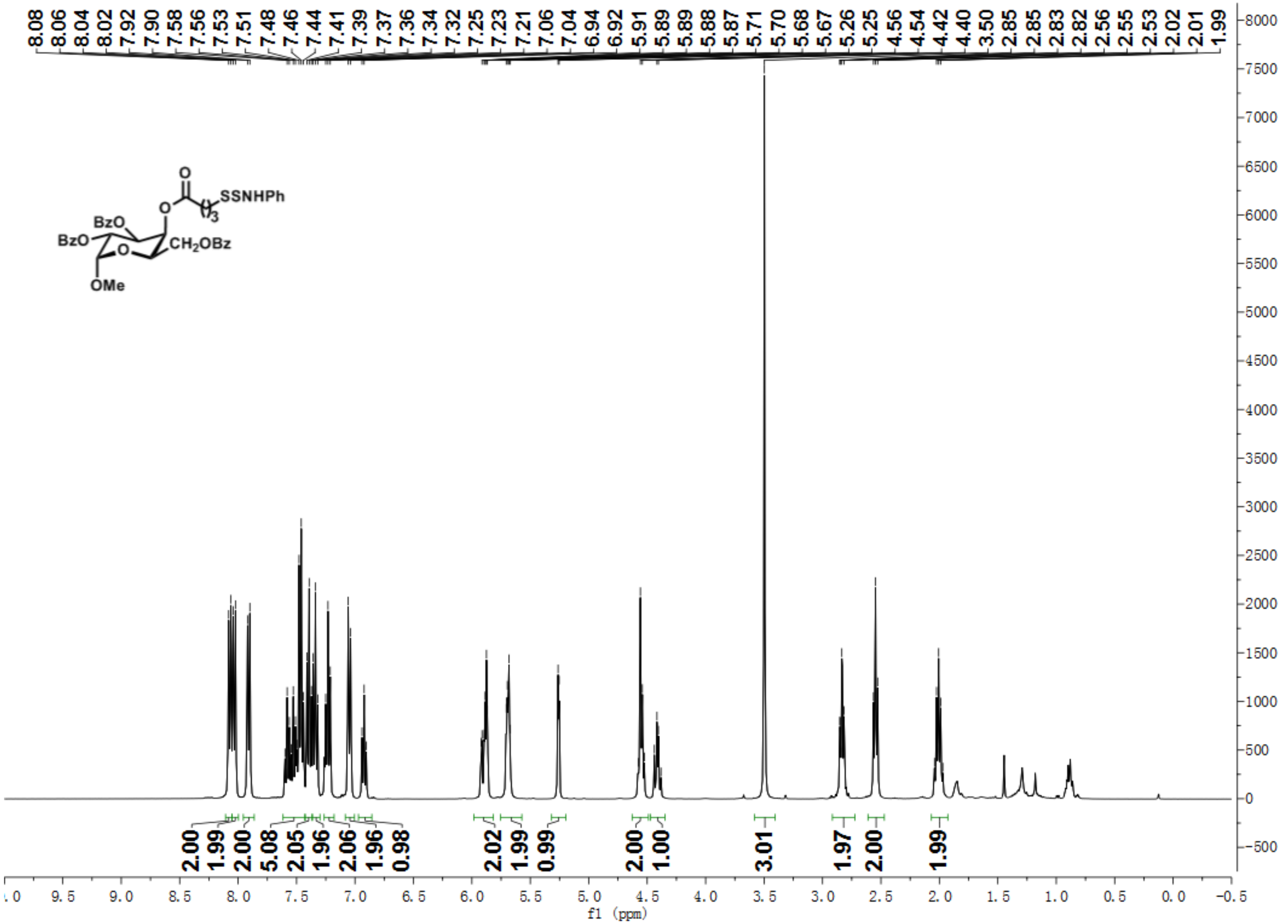

Supplementary Figure 174. ^{1}H NMR spectra for Compound **6x**.

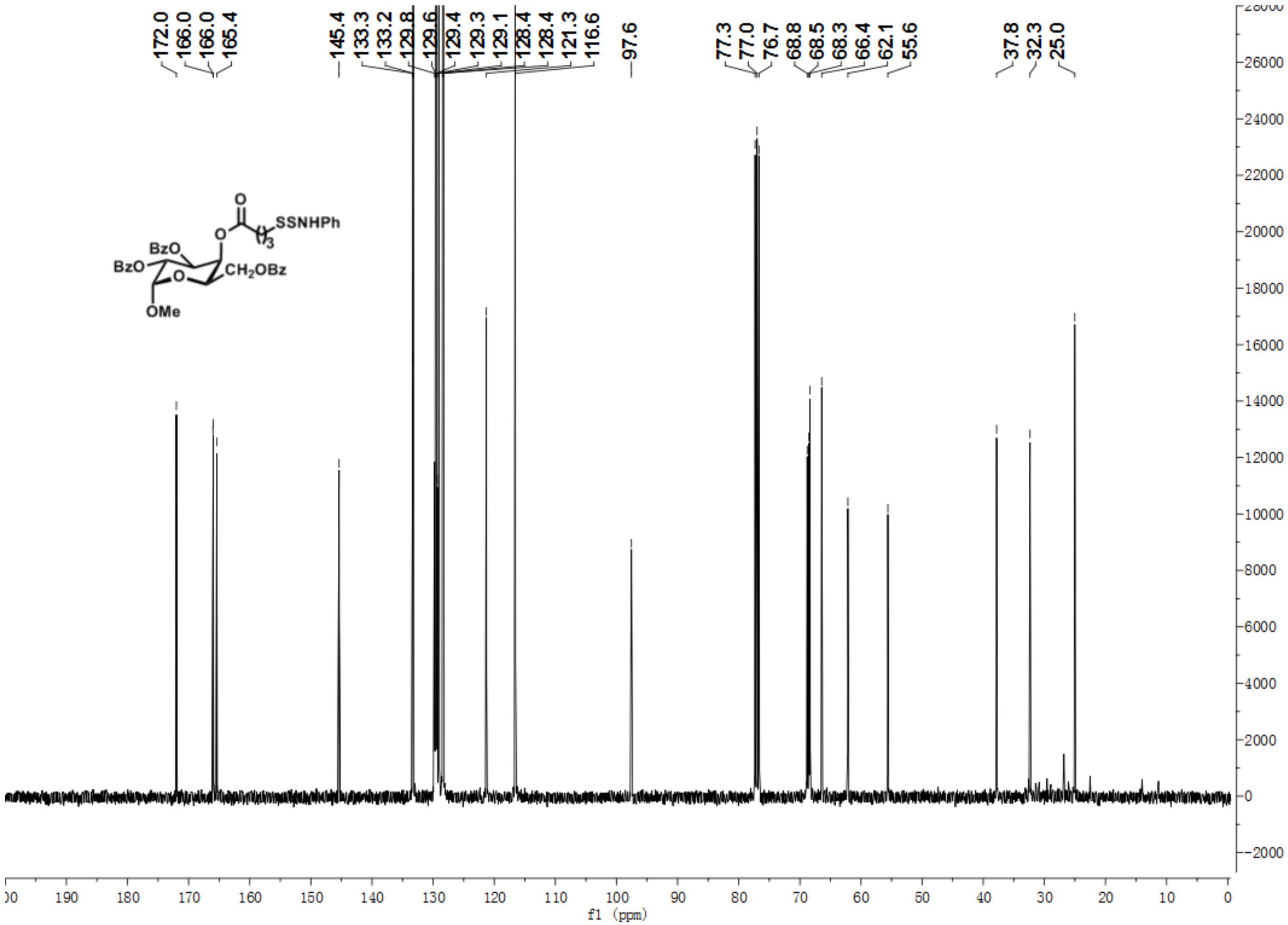

Supplementary Figure 175. ^{13}C NMR spectra for Compound **6x**.

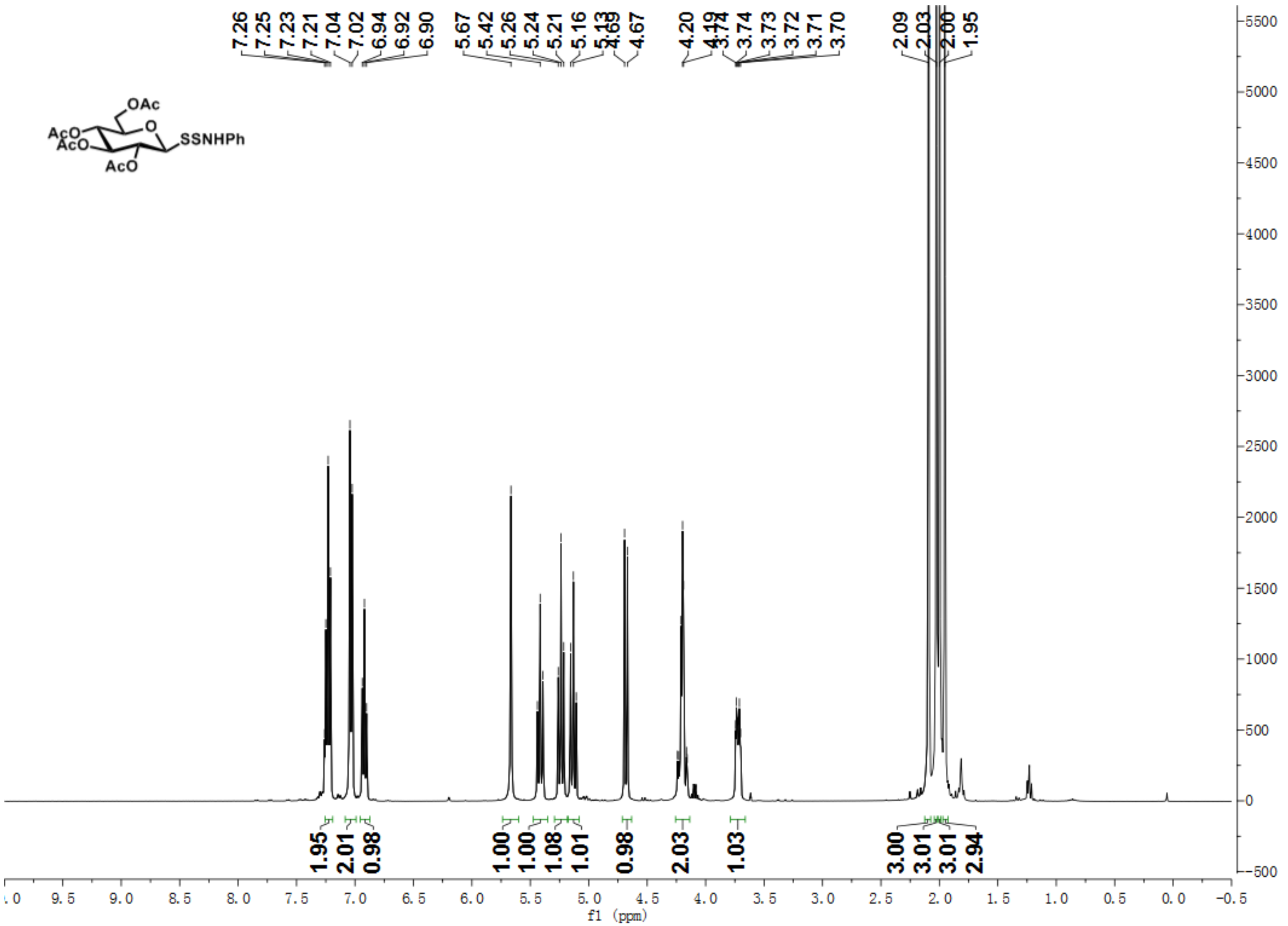

Supplementary Figure 176. ^{1}H NMR spectra for Compound **6y**.

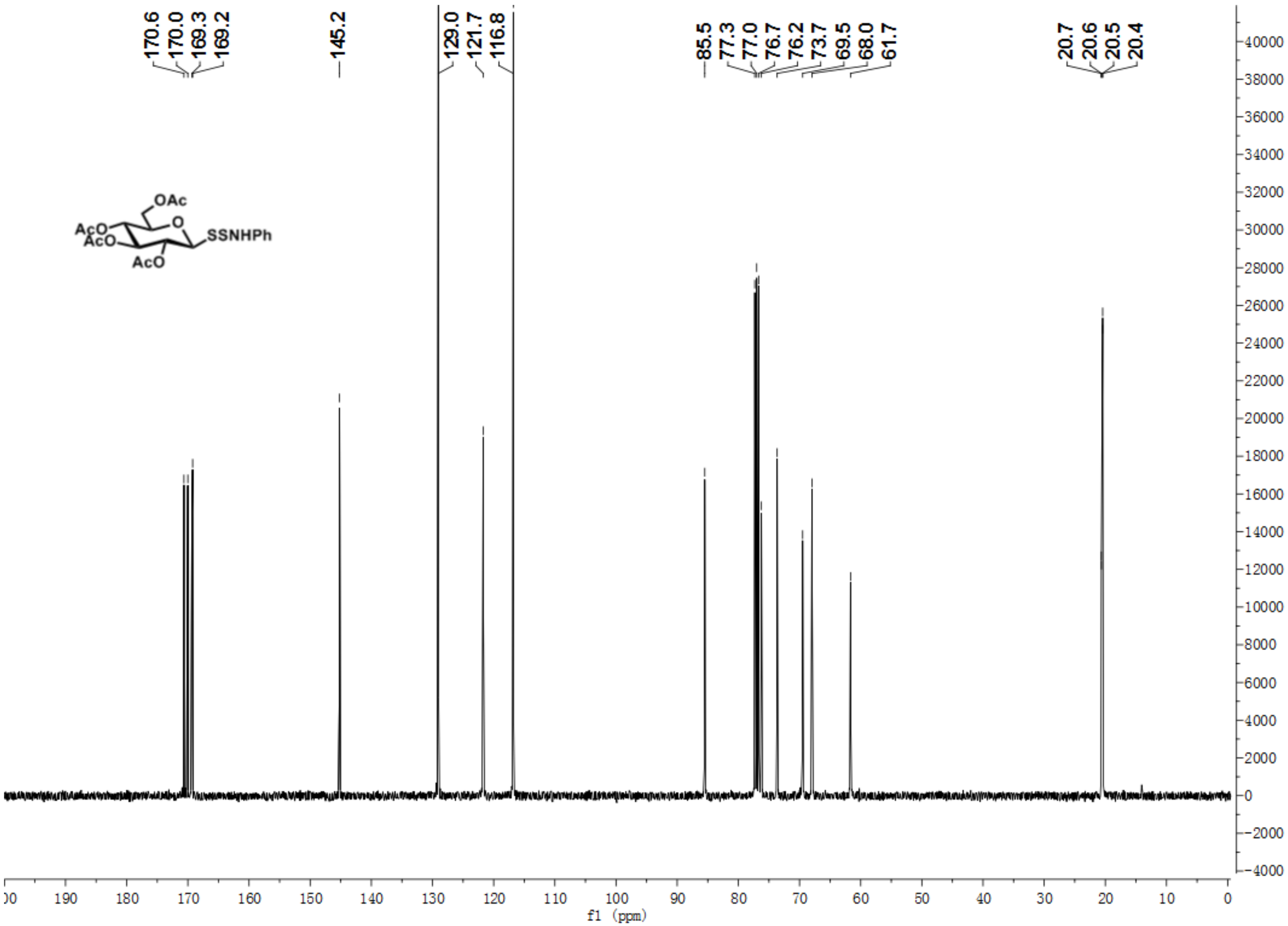

Supplementary Figure 177. ^{13}C NMR spectra for Compound **6y**.

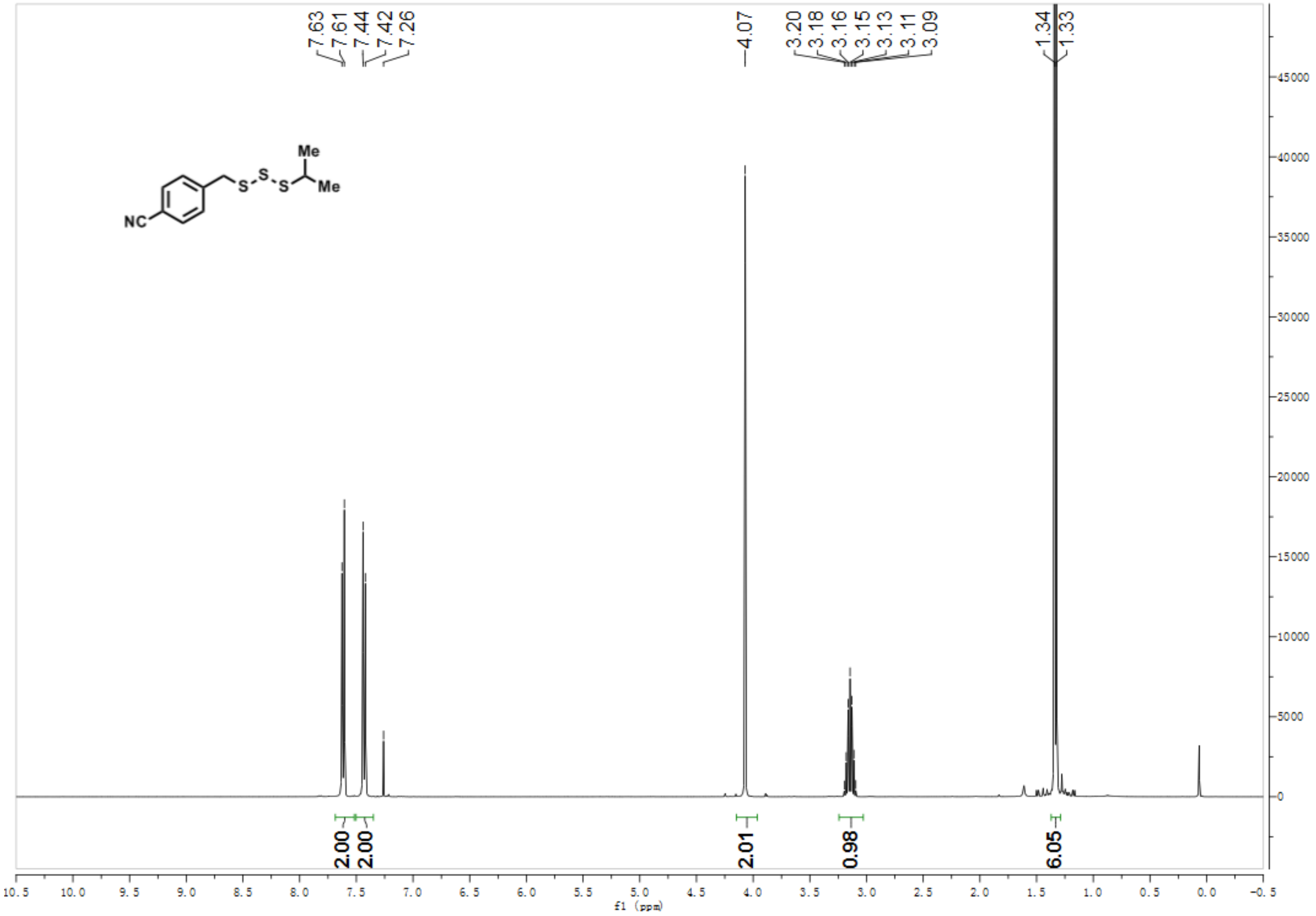

Supplementary Figure 178. ^{1}H NMR spectra for Compound **7a**.

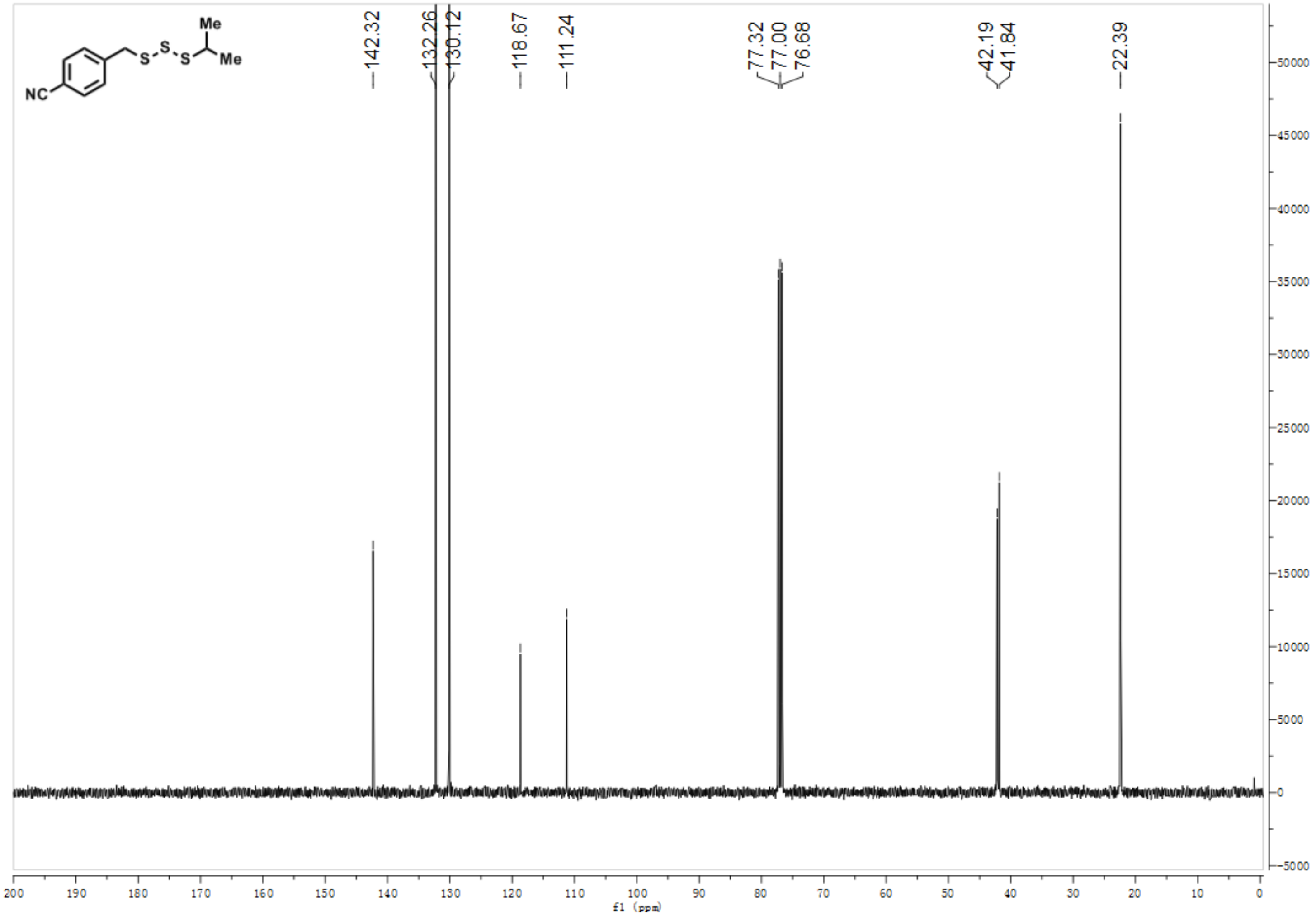

Supplementary Figure 179. ^{13}C NMR spectra for Compound **7a**.

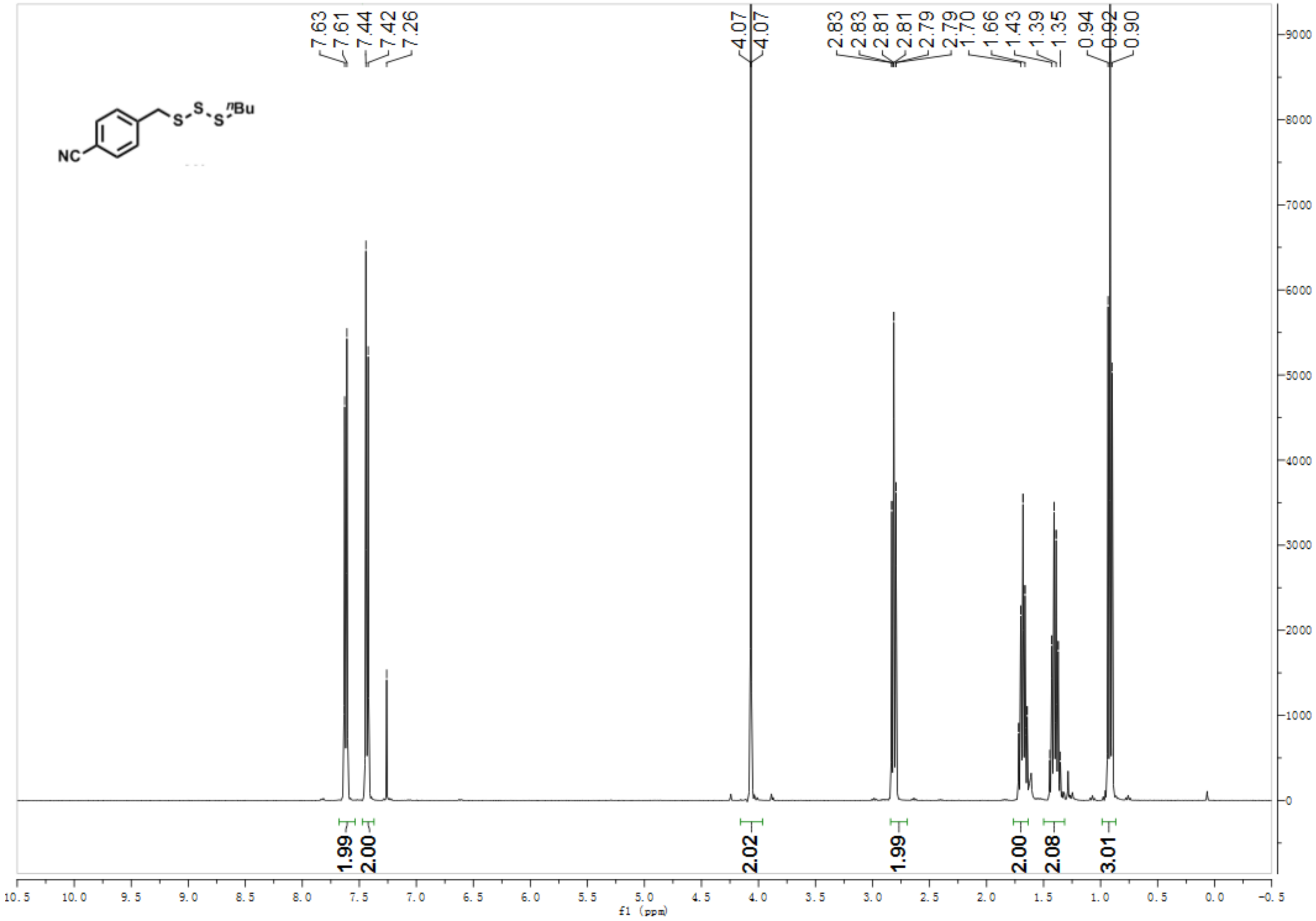

Supplementary Figure 180. ^{1}H NMR spectra for Compound **7b**.

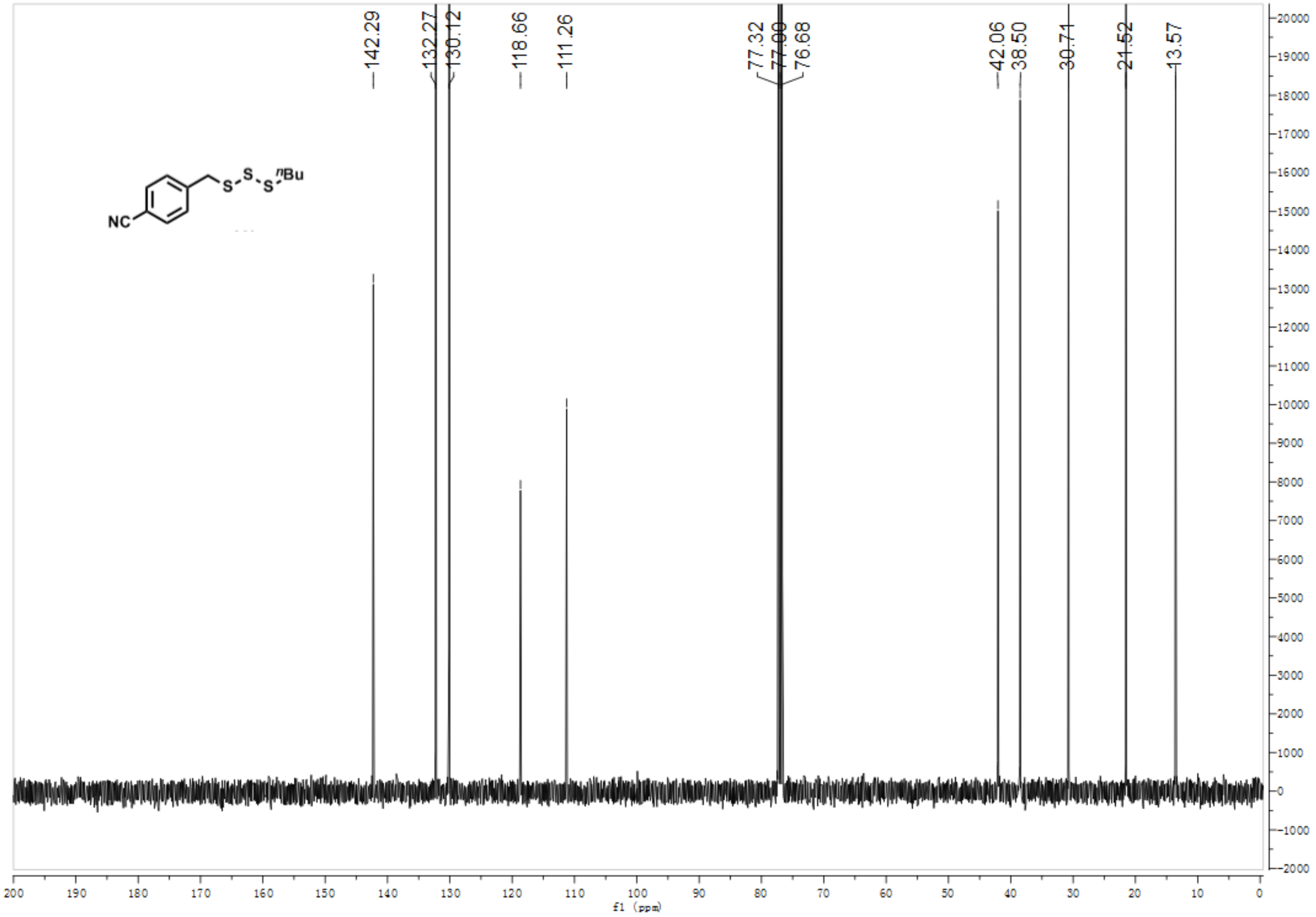

Supplementary Figure 181. ^{13}C NMR spectra for Compound **7b**.

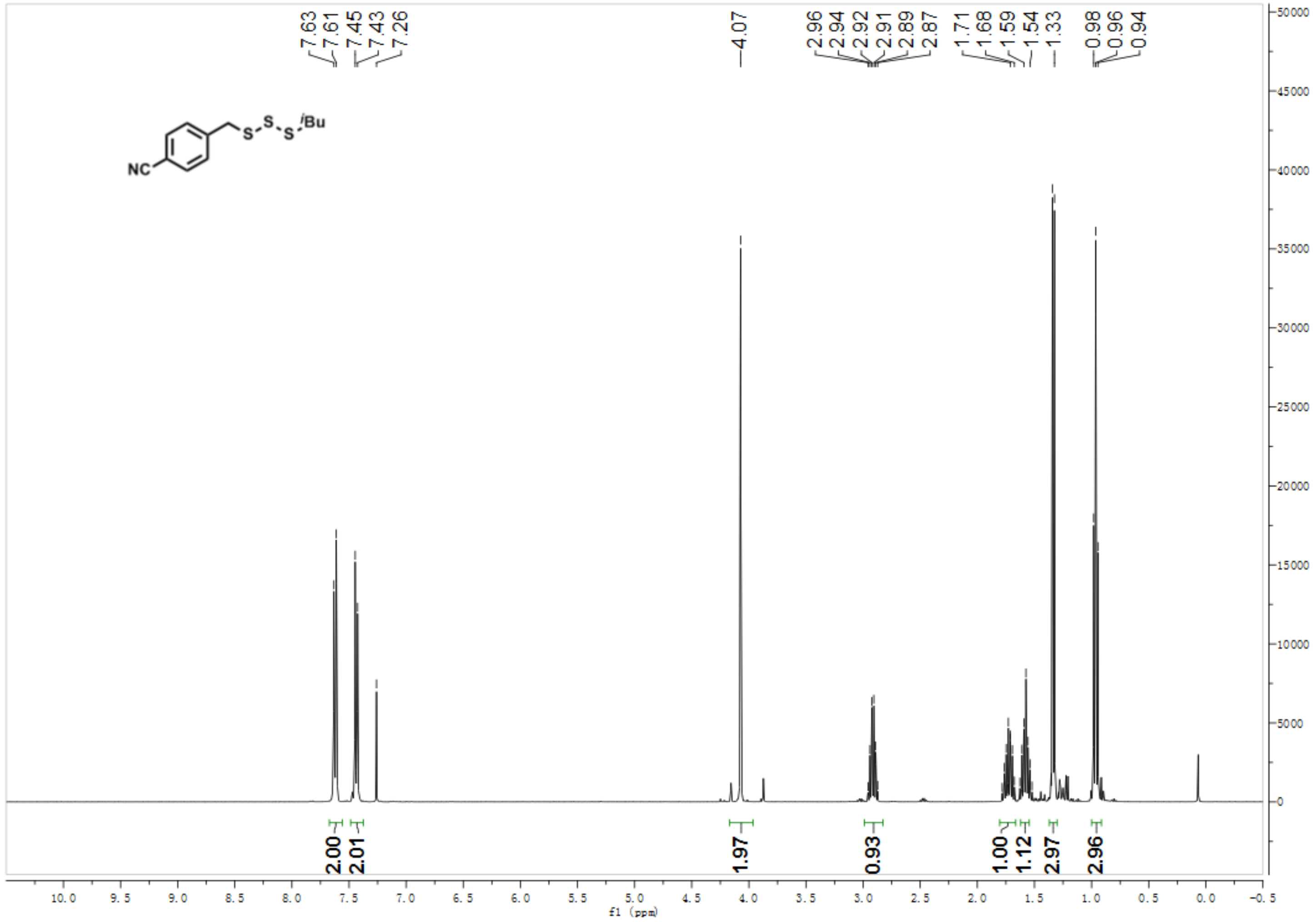

Supplementary Figure 182. ^{1}H NMR spectra for Compound **7c**.

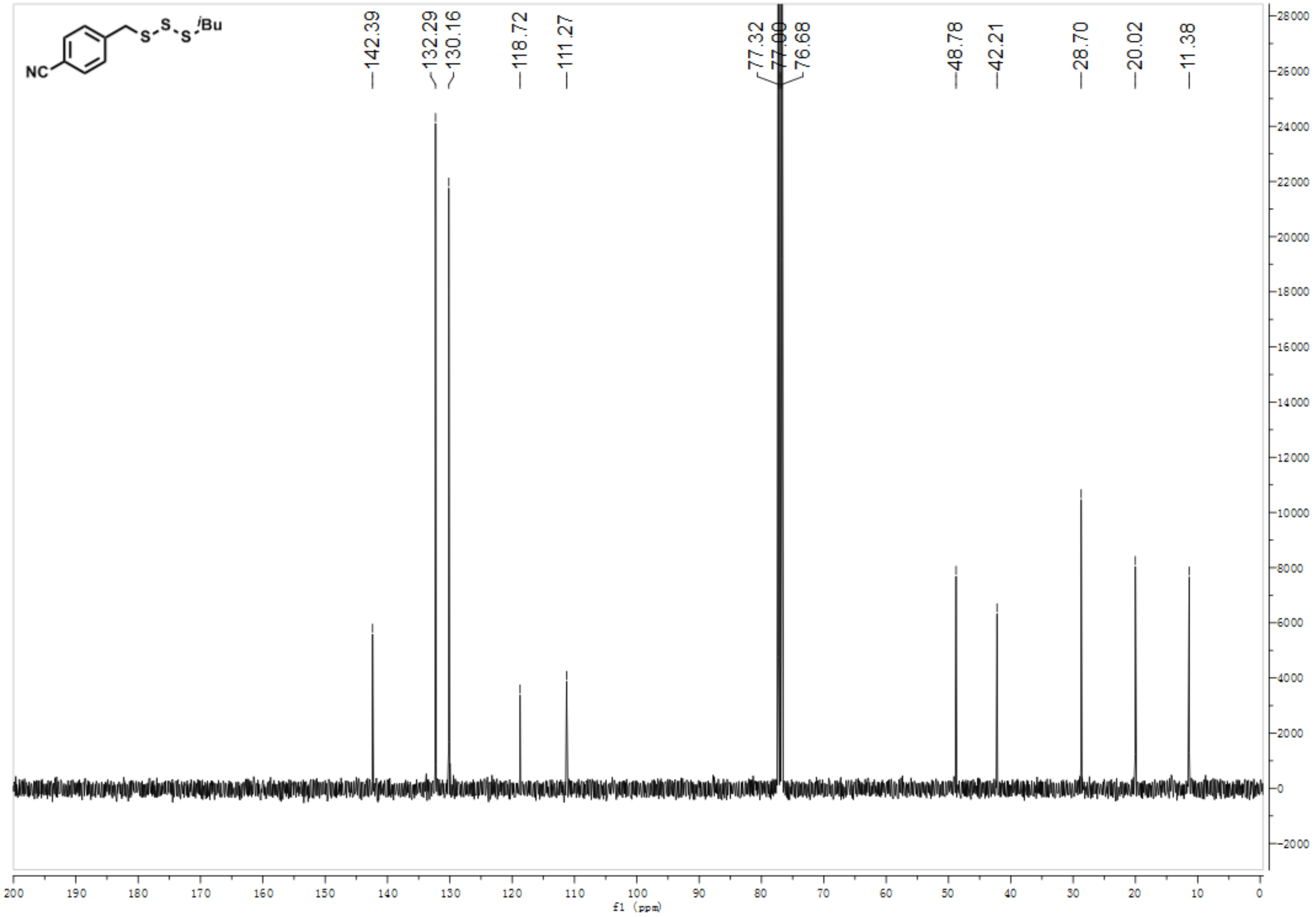

Supplementary Figure 183. ^{13}C NMR spectra for Compound **7c**.

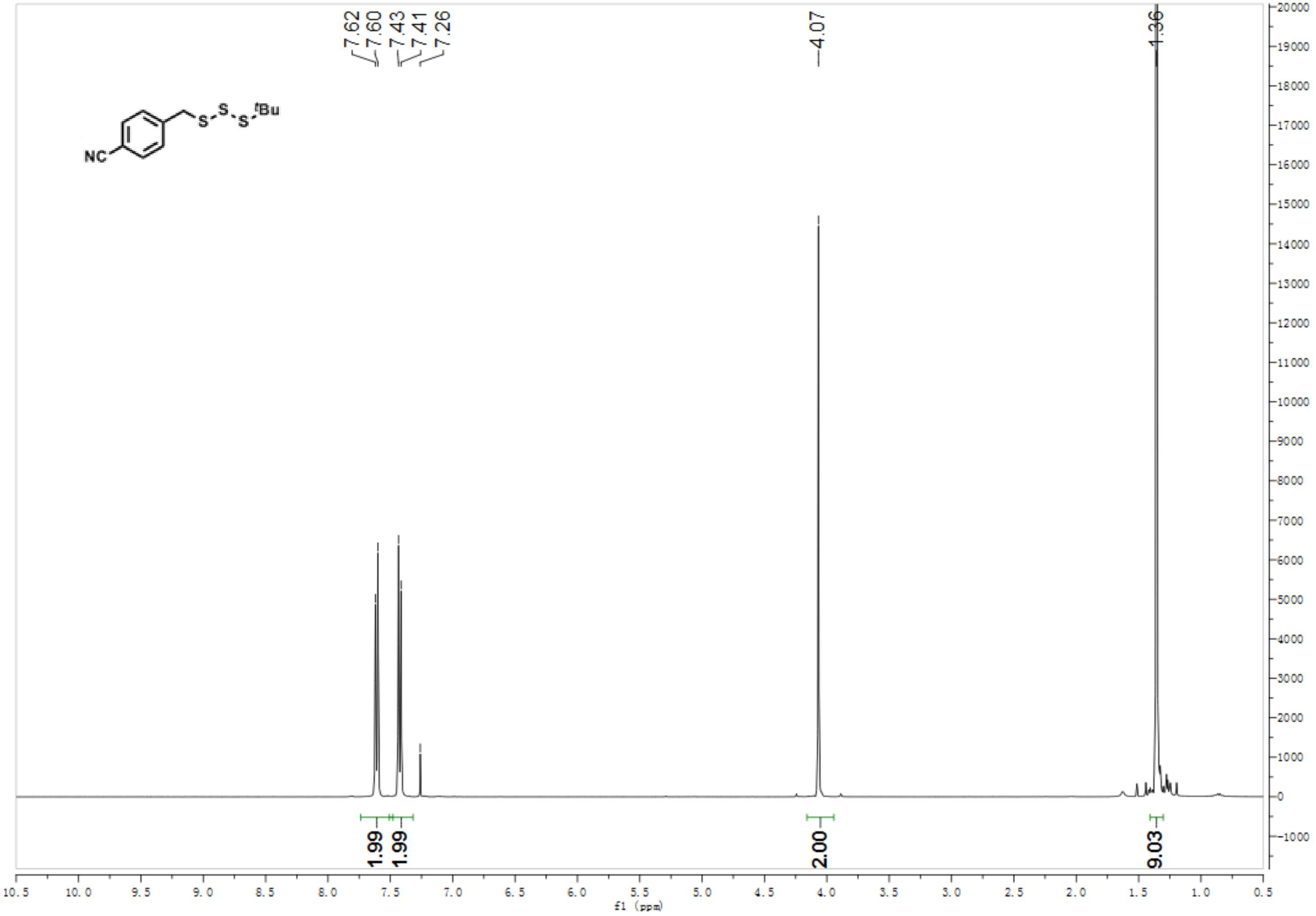

Supplementary Figure 184. ^{1}H NMR spectra for Compound **7d**.

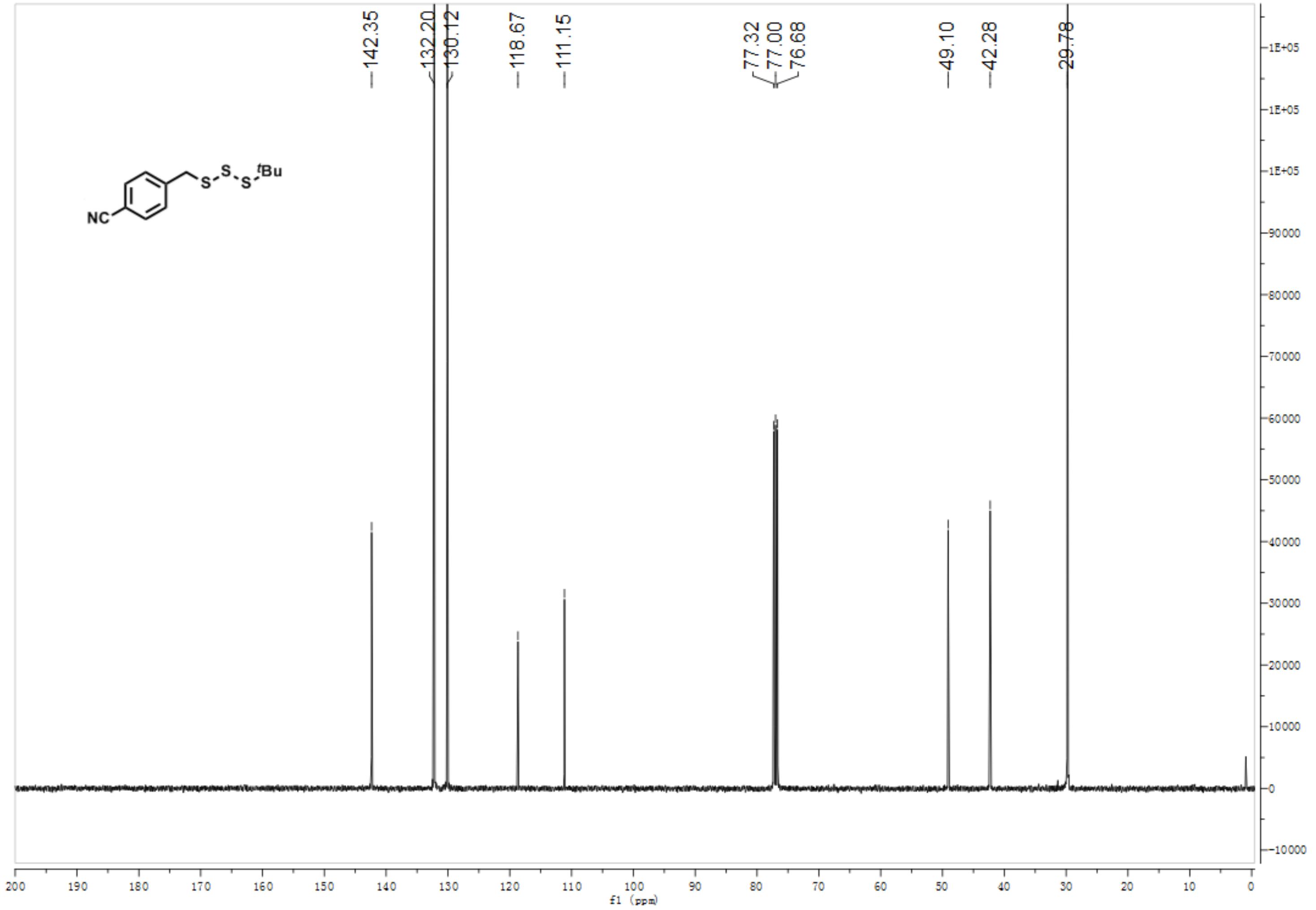

Supplementary Figure 185. ^{13}C NMR spectra for Compound **7d**.

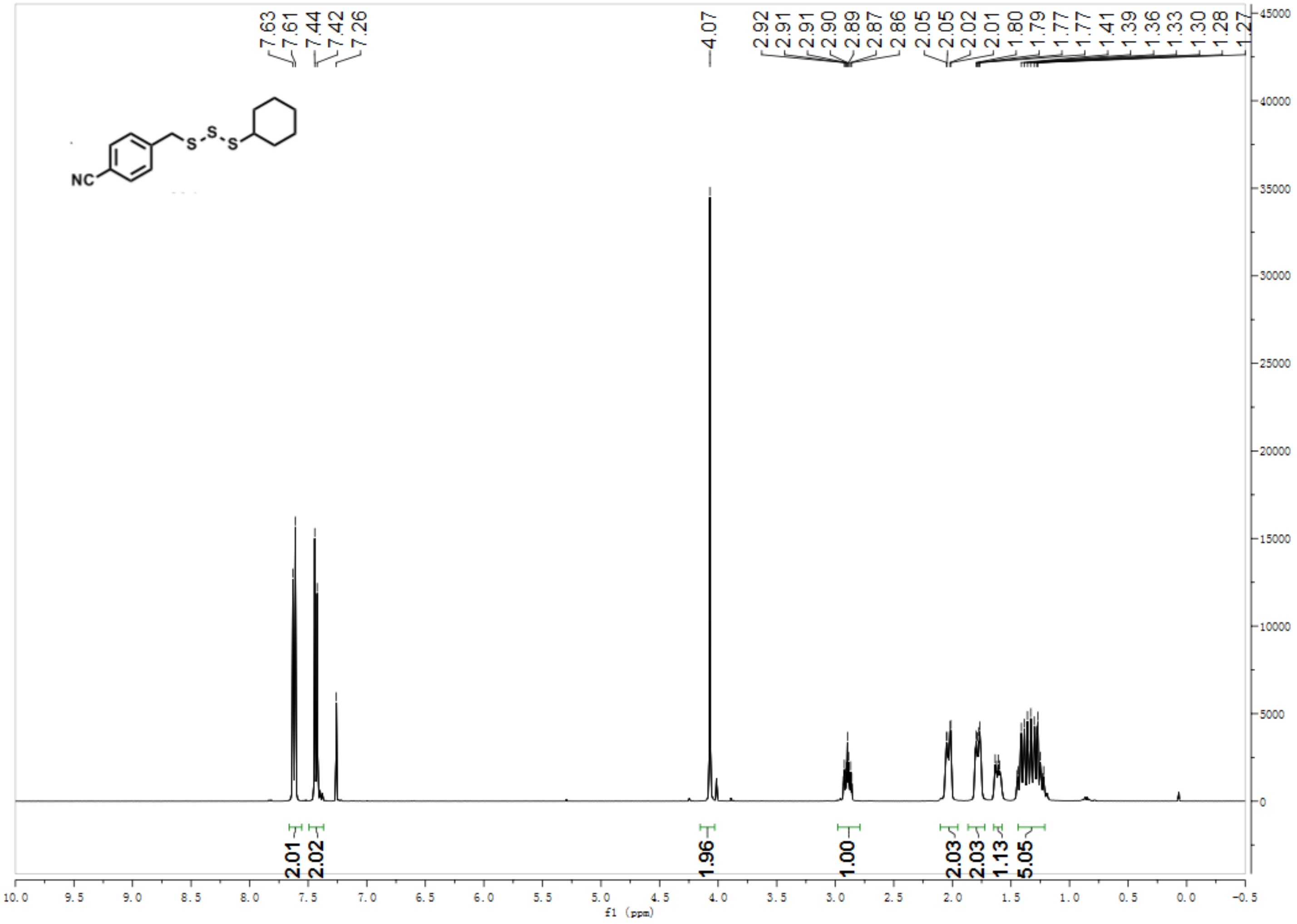

Supplementary Figure 186. ^{1}H NMR spectra for Compound **7e**.

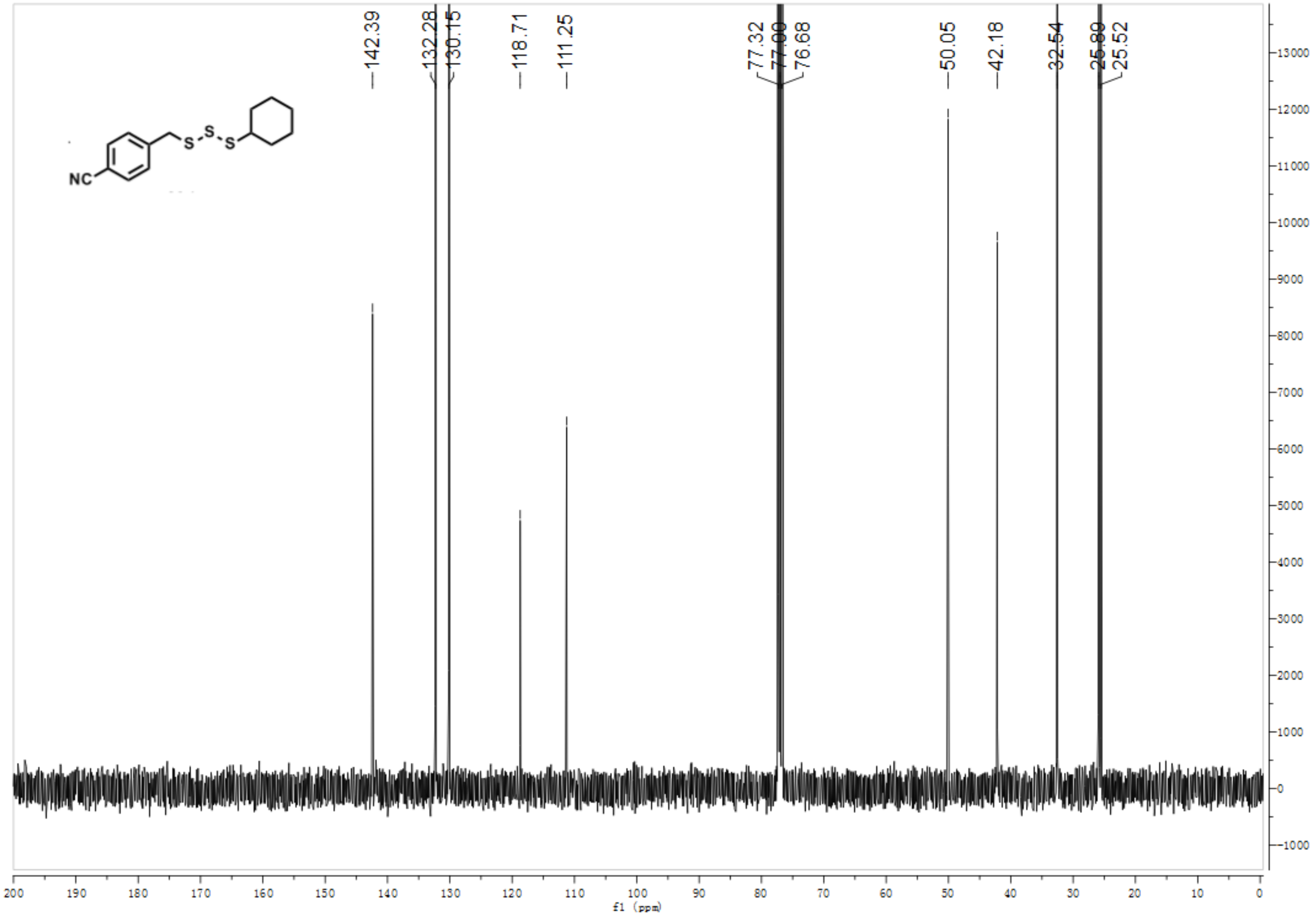

Supplementary Figure 187. ^{13}C NMR spectra for Compound **7e**.

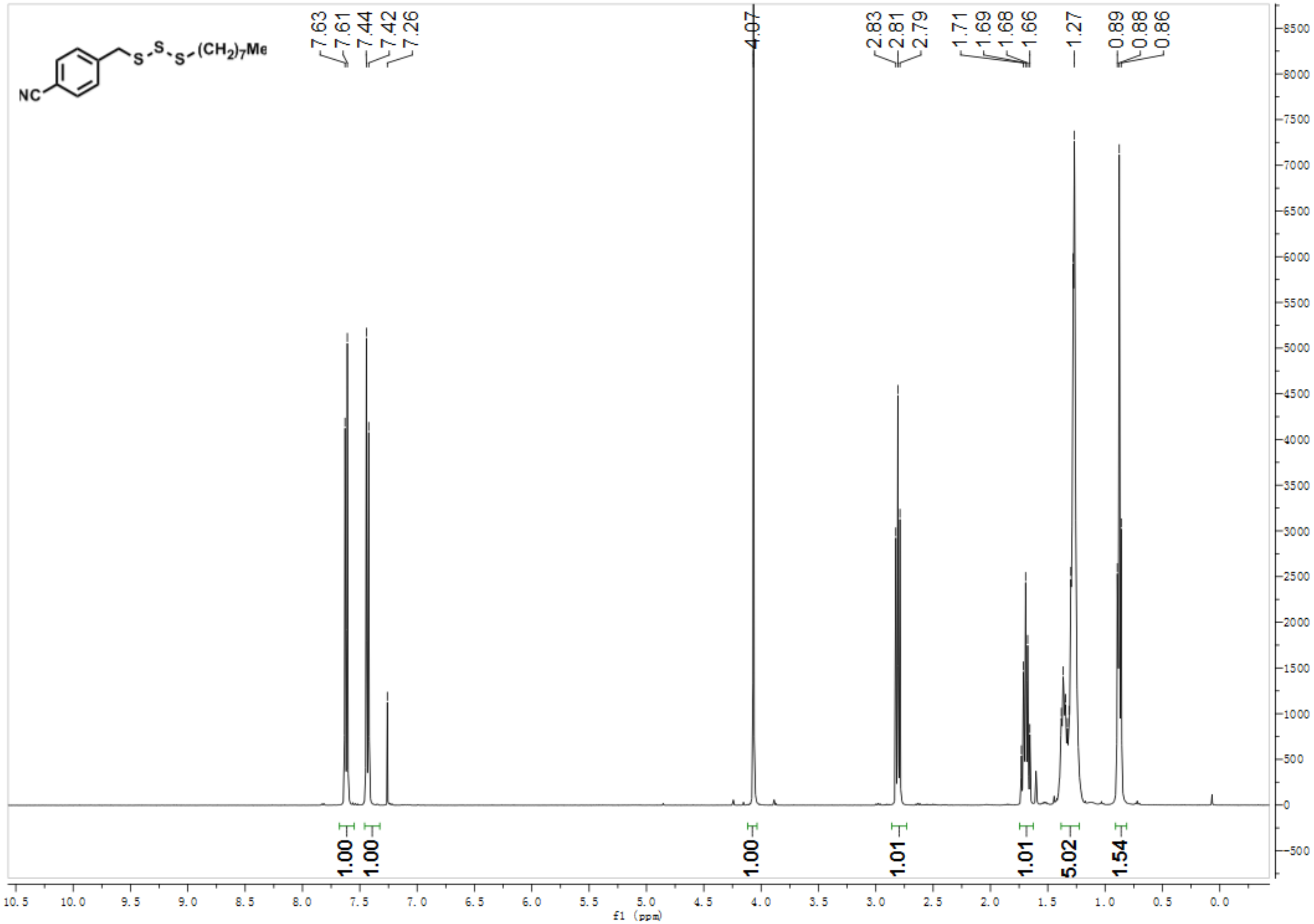

Supplementary Figure 188. ^{1}H NMR spectra for Compound **7f**.

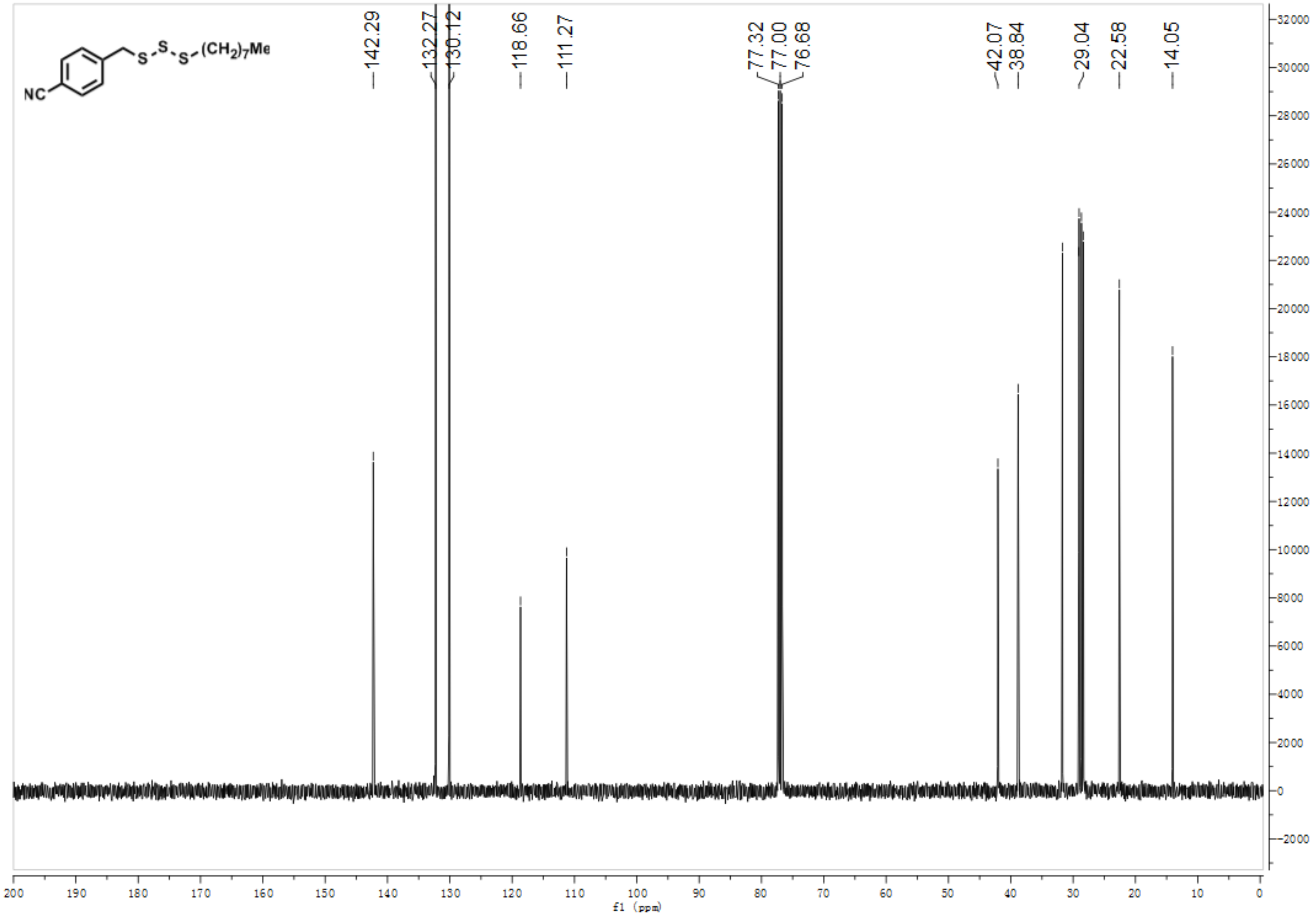

Supplementary Figure 189. ^{13}C NMR spectra for Compound **7f**.

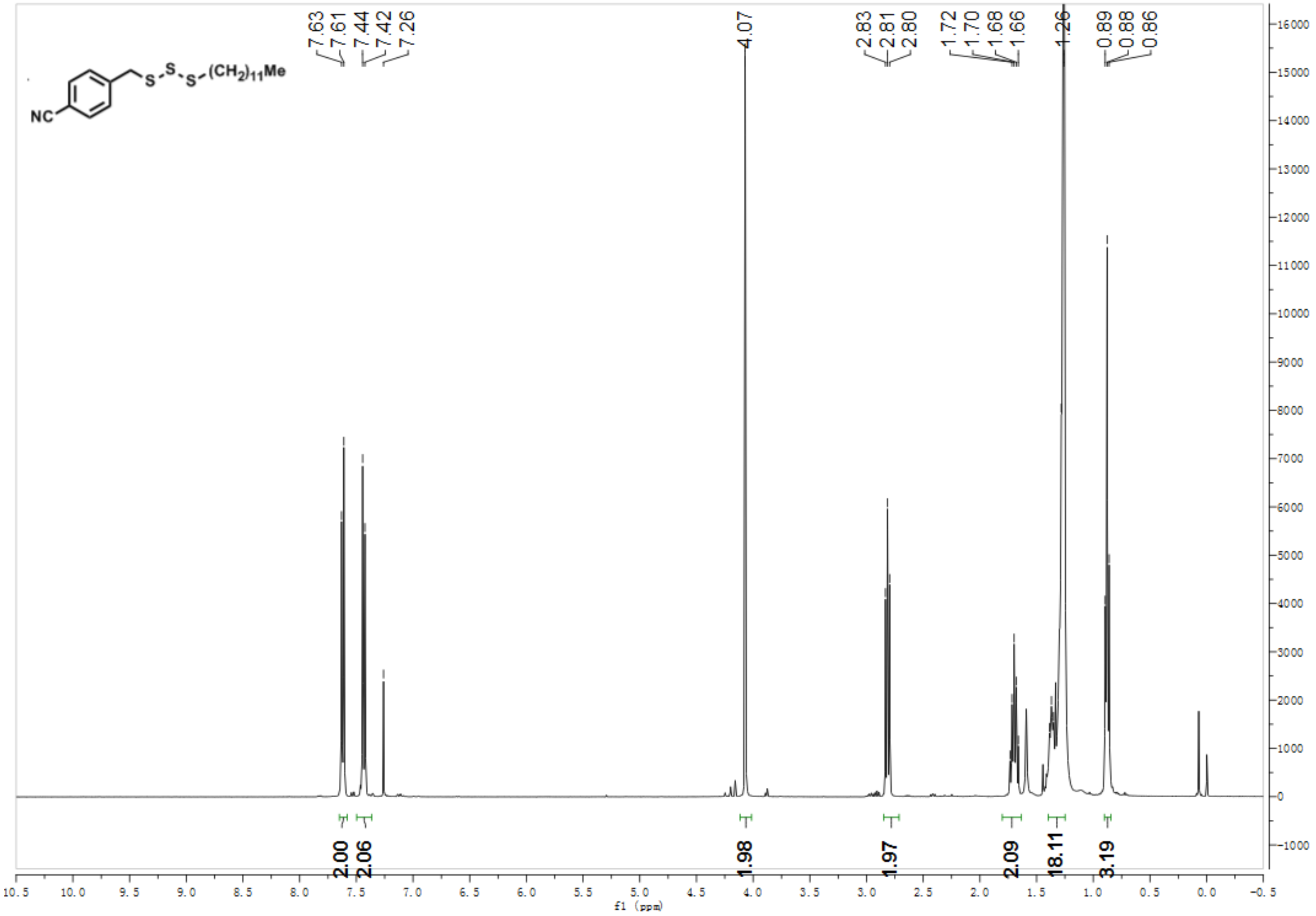

Supplementary Figure 190. ^{1}H NMR spectra for Compound **7g**.

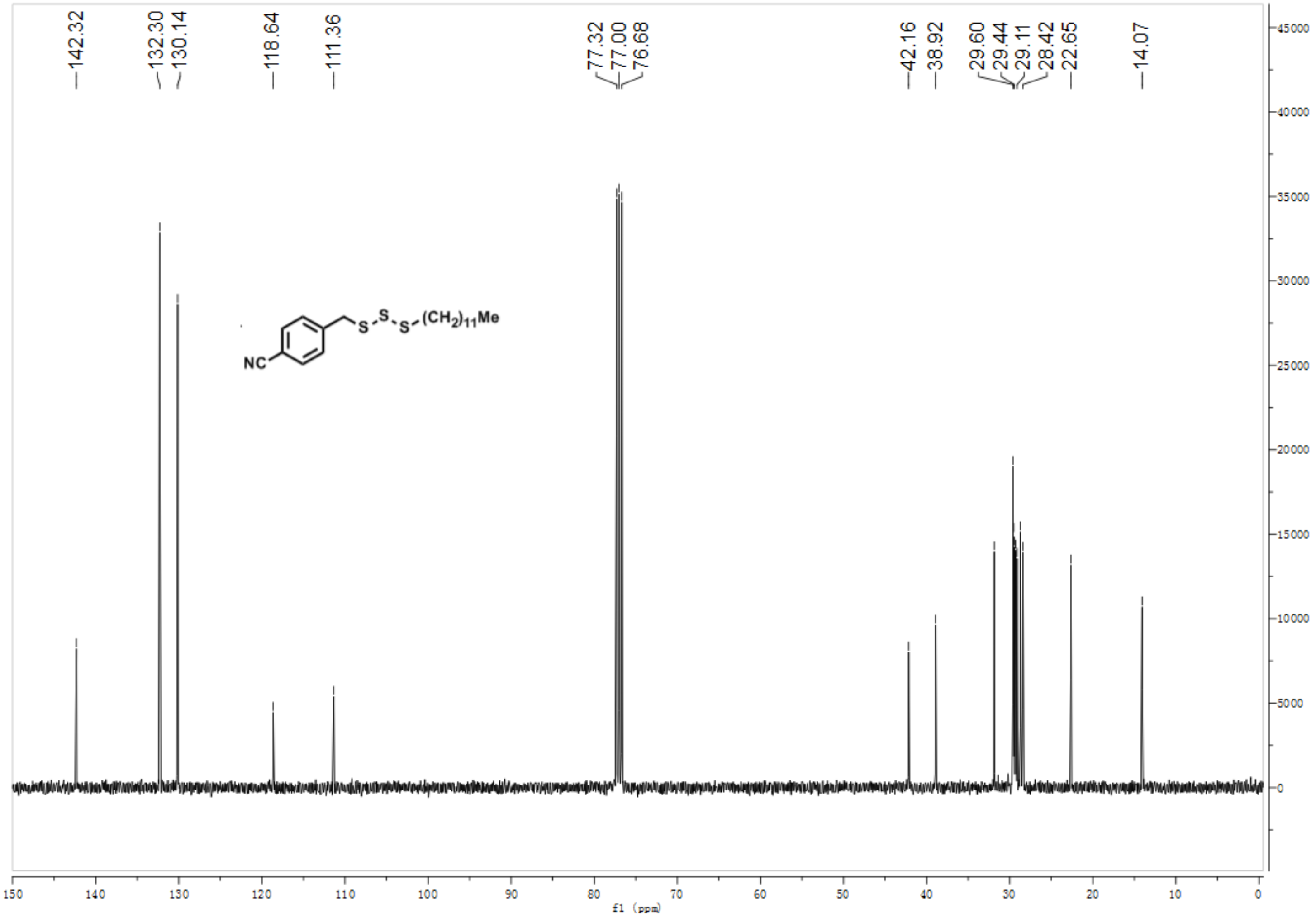

Supplementary Figure 191. ^{13}C NMR spectra for Compound **7g**.

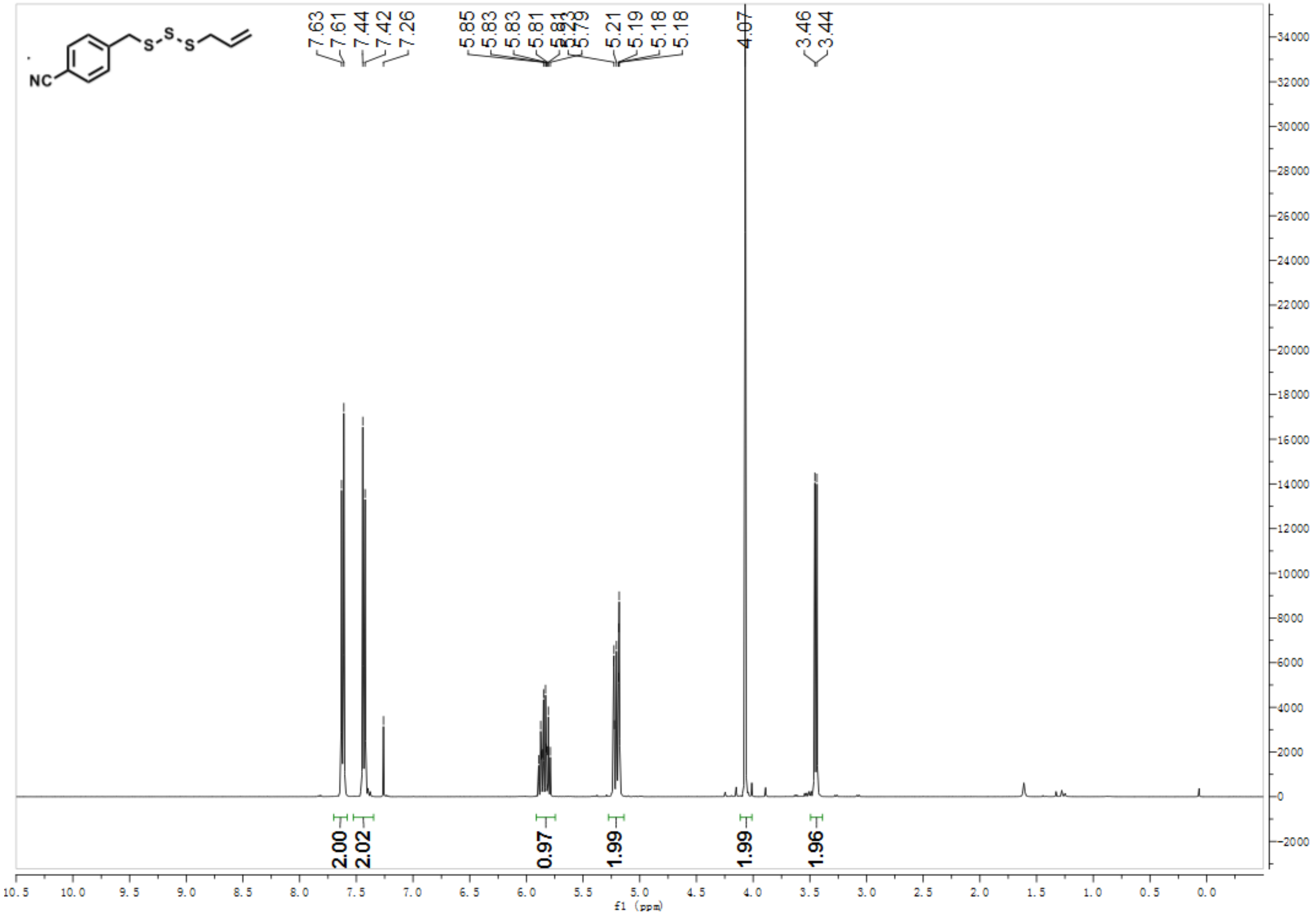

Supplementary Figure 192. ^{1}H NMR spectra for Compound **7h**.

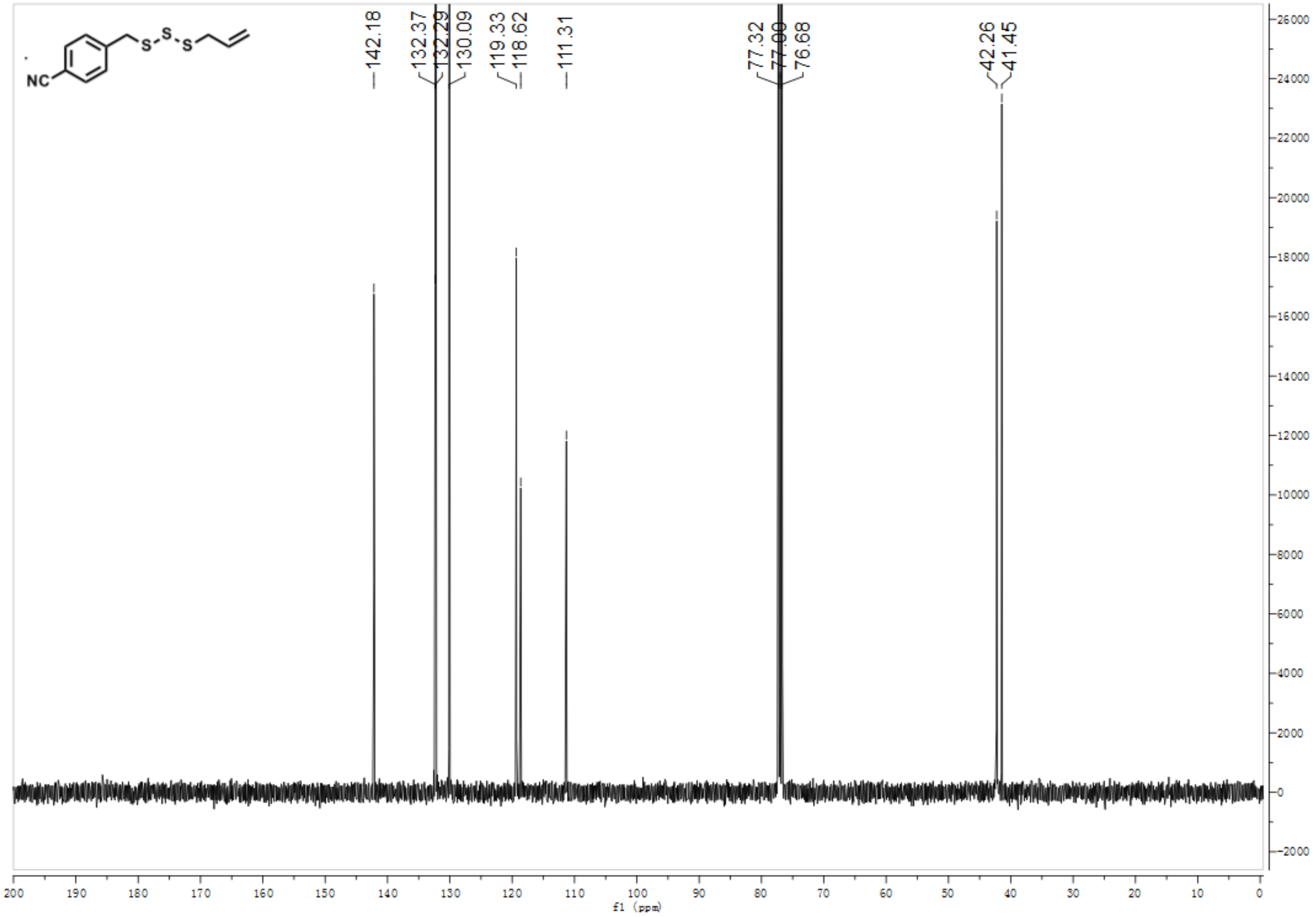

Supplementary Figure 193. ^{13}C NMR spectra for Compound **7h**.

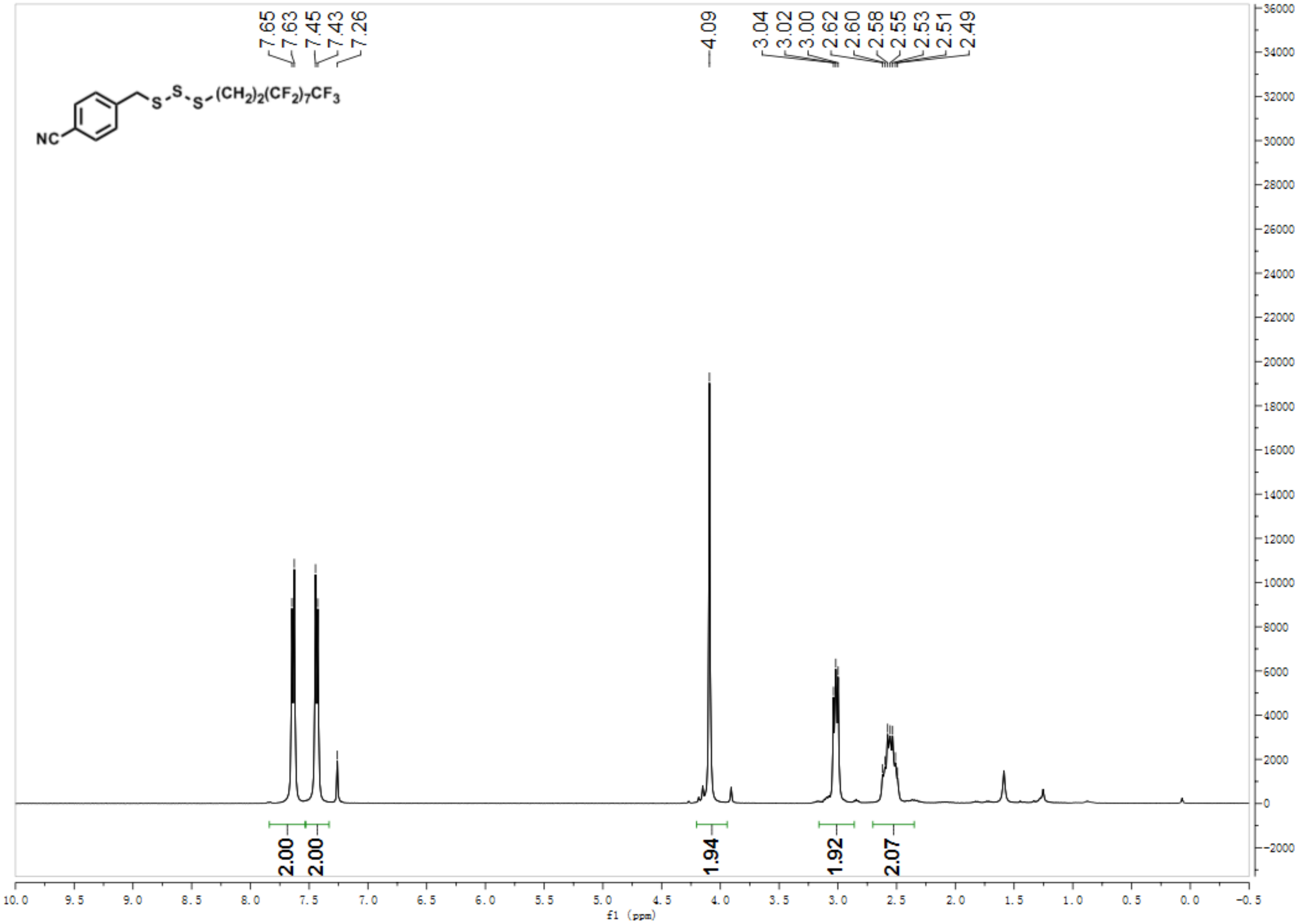

Supplementary Figure 194. 13H NMR spectra for Compound **7i**.

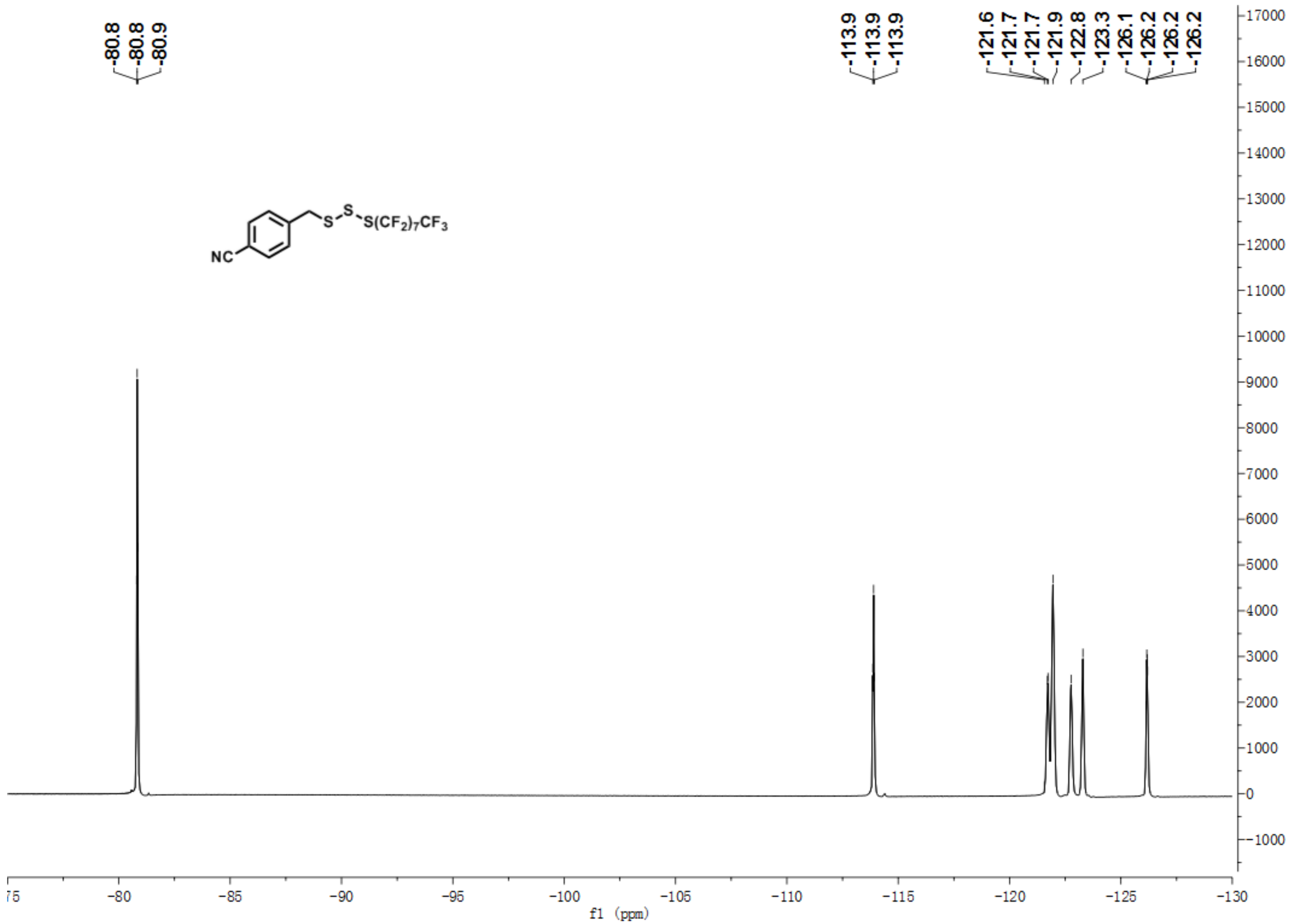

Supplementary Figure 195. ^{19}F NMR spectra for Compound **7i**.

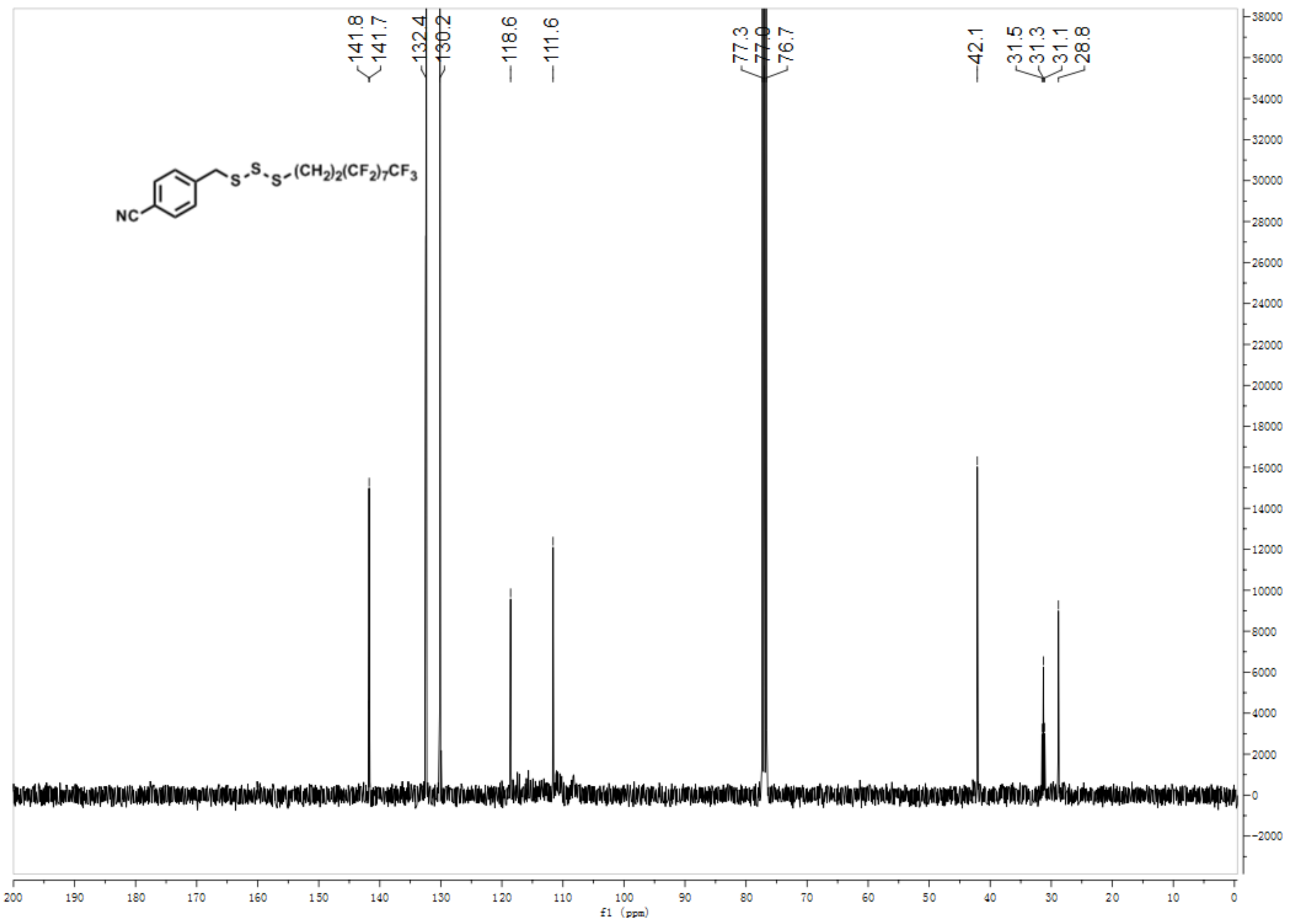

Supplementary Figure 196. ^{13}C NMR spectra for Compound **7i**.

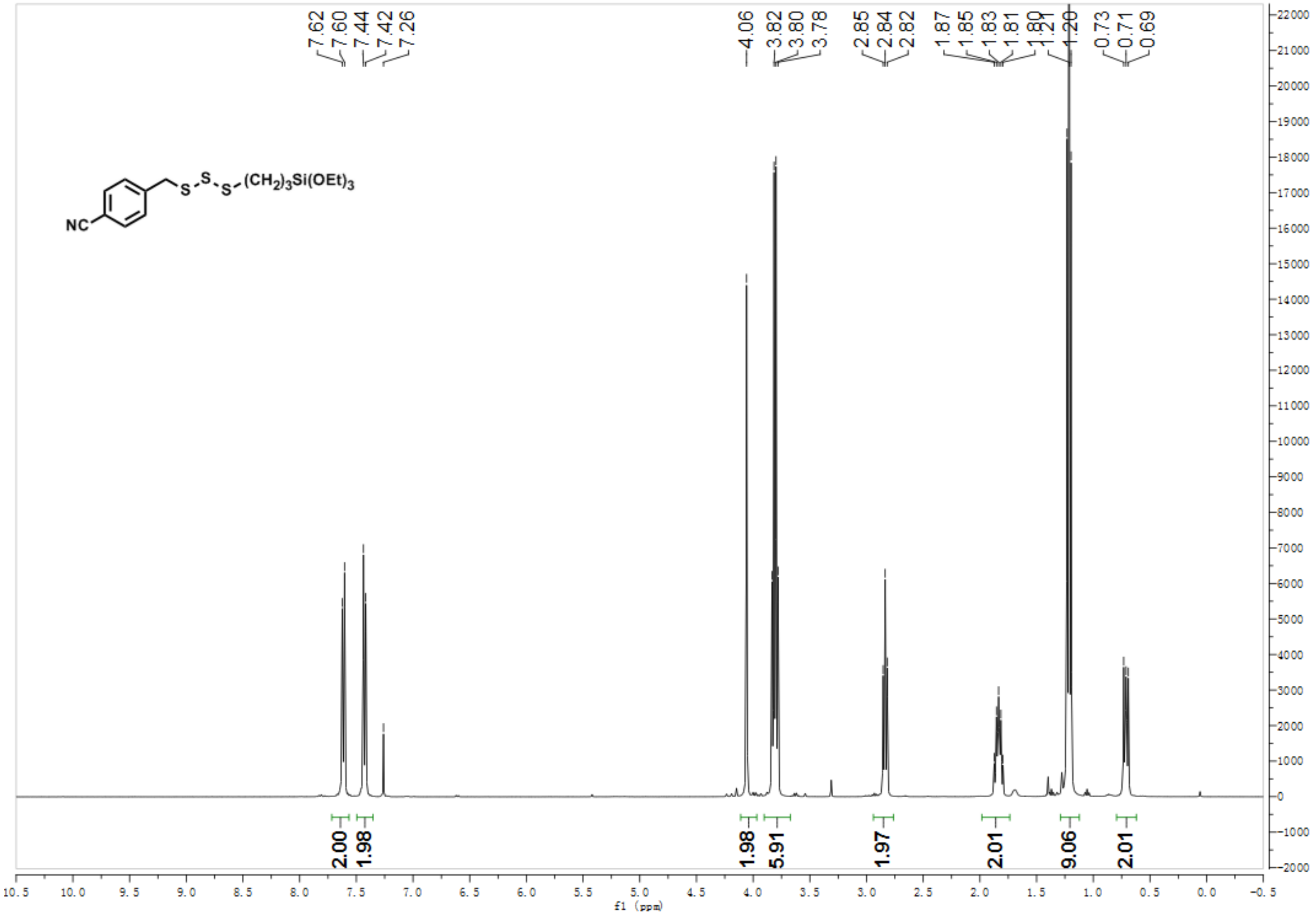

Supplementary Figure 197. ^{1}H NMR spectra for Compound **7j**.

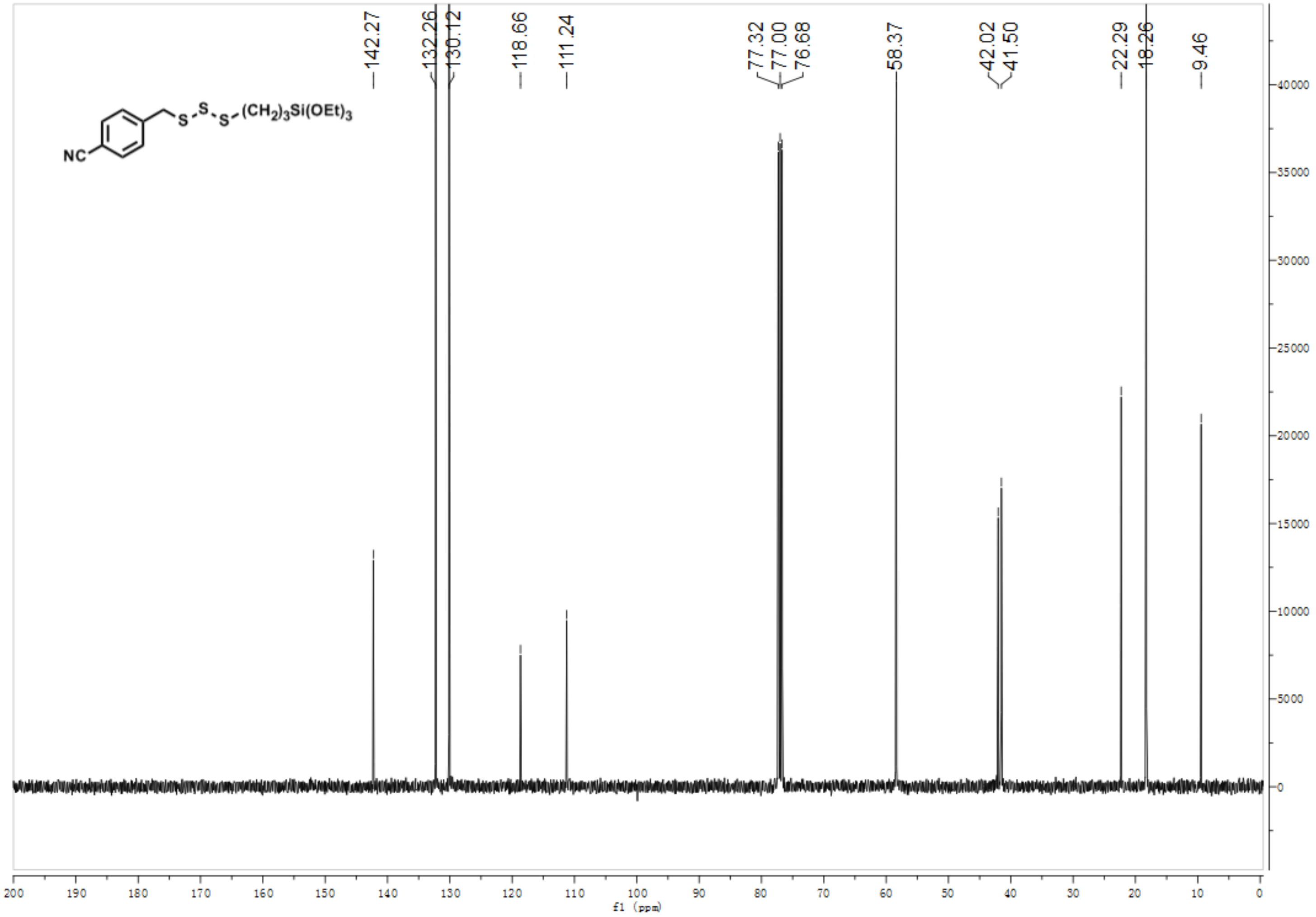

Supplementary Figure 198. ^{13}C NMR spectra for Compound **7j**.

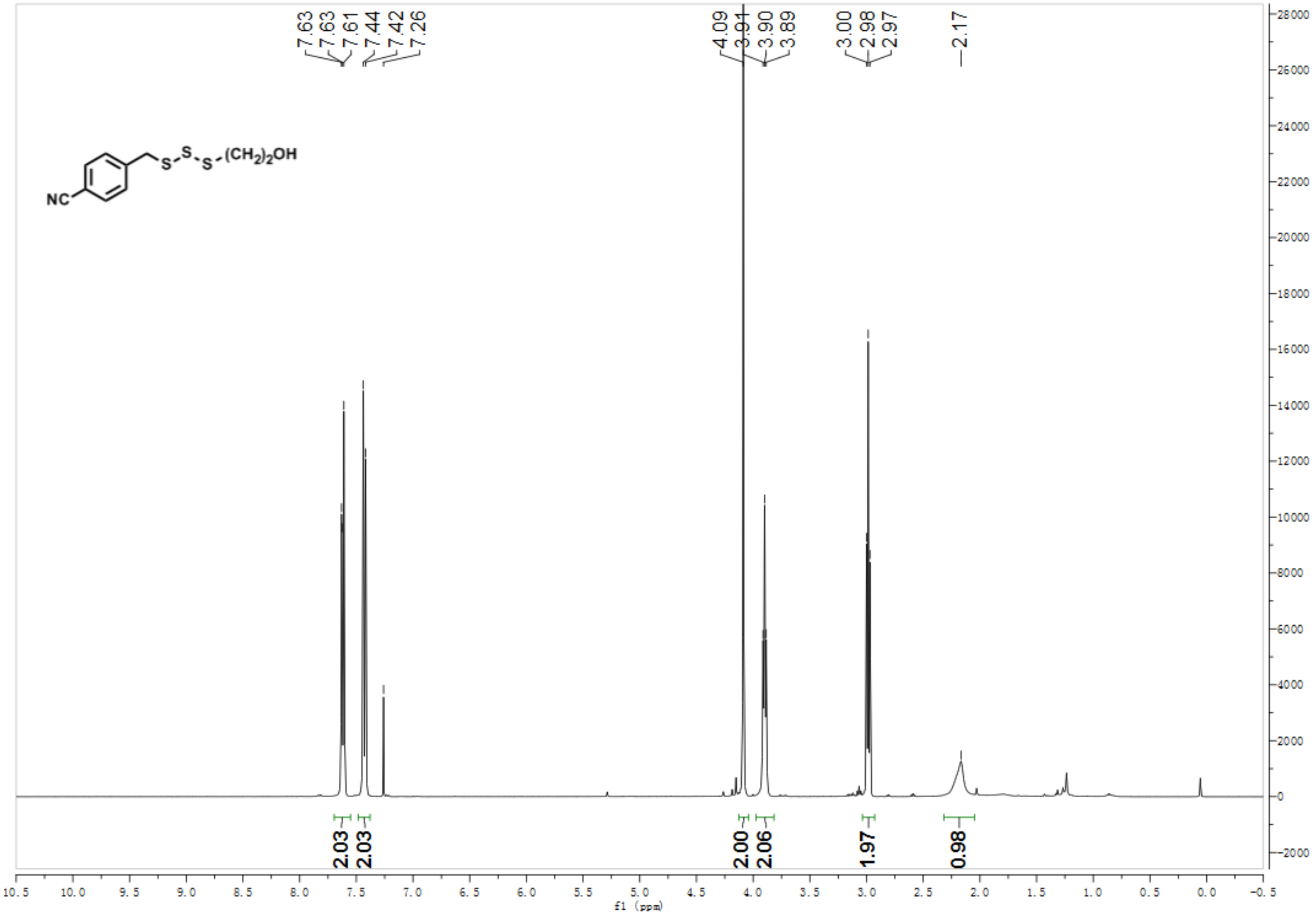

Supplementary Figure 199. ^{1}H NMR spectra for Compound **7k**.

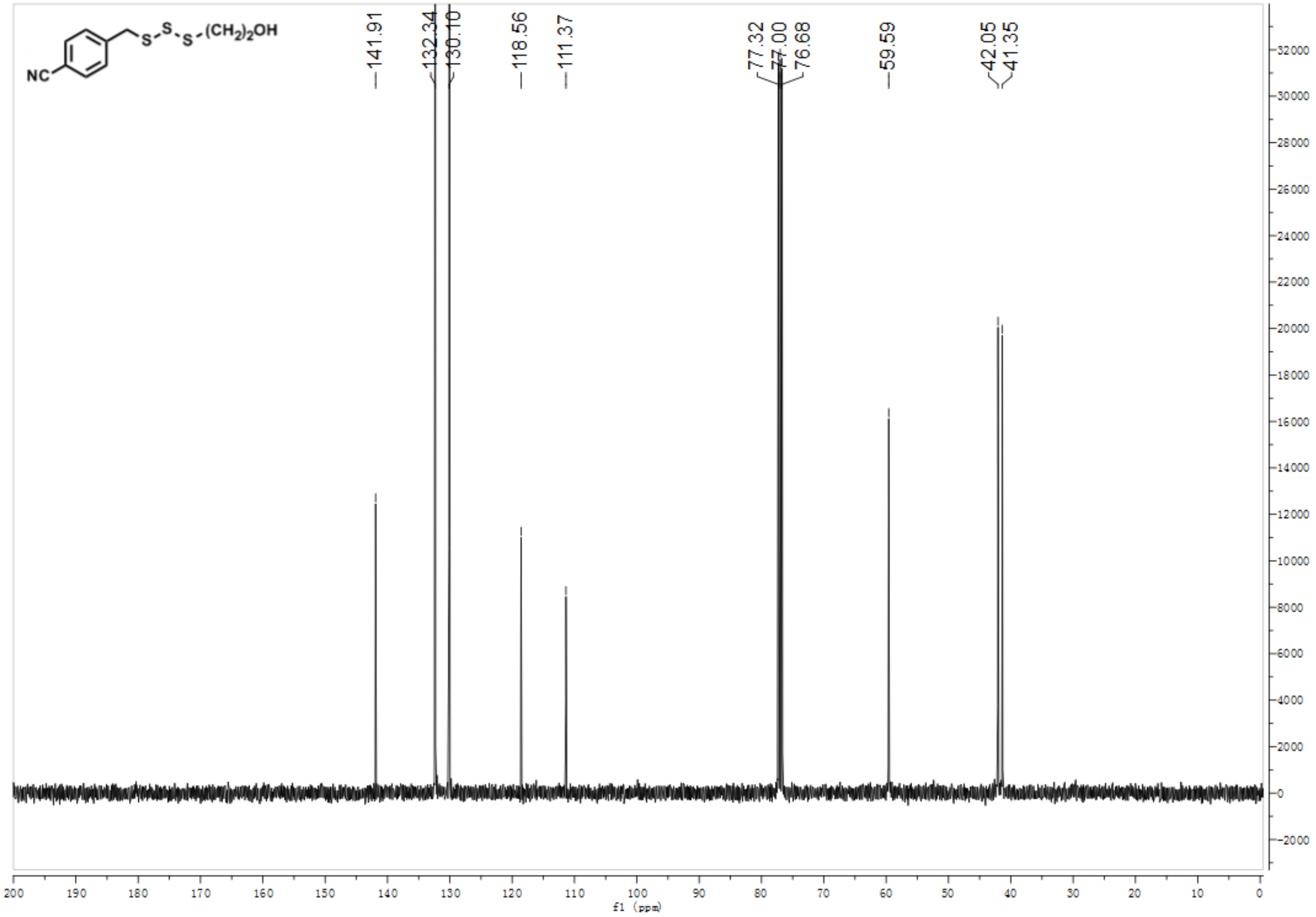

Supplementary Figure 200. ^{13}C NMR spectra for Compound **7k**.

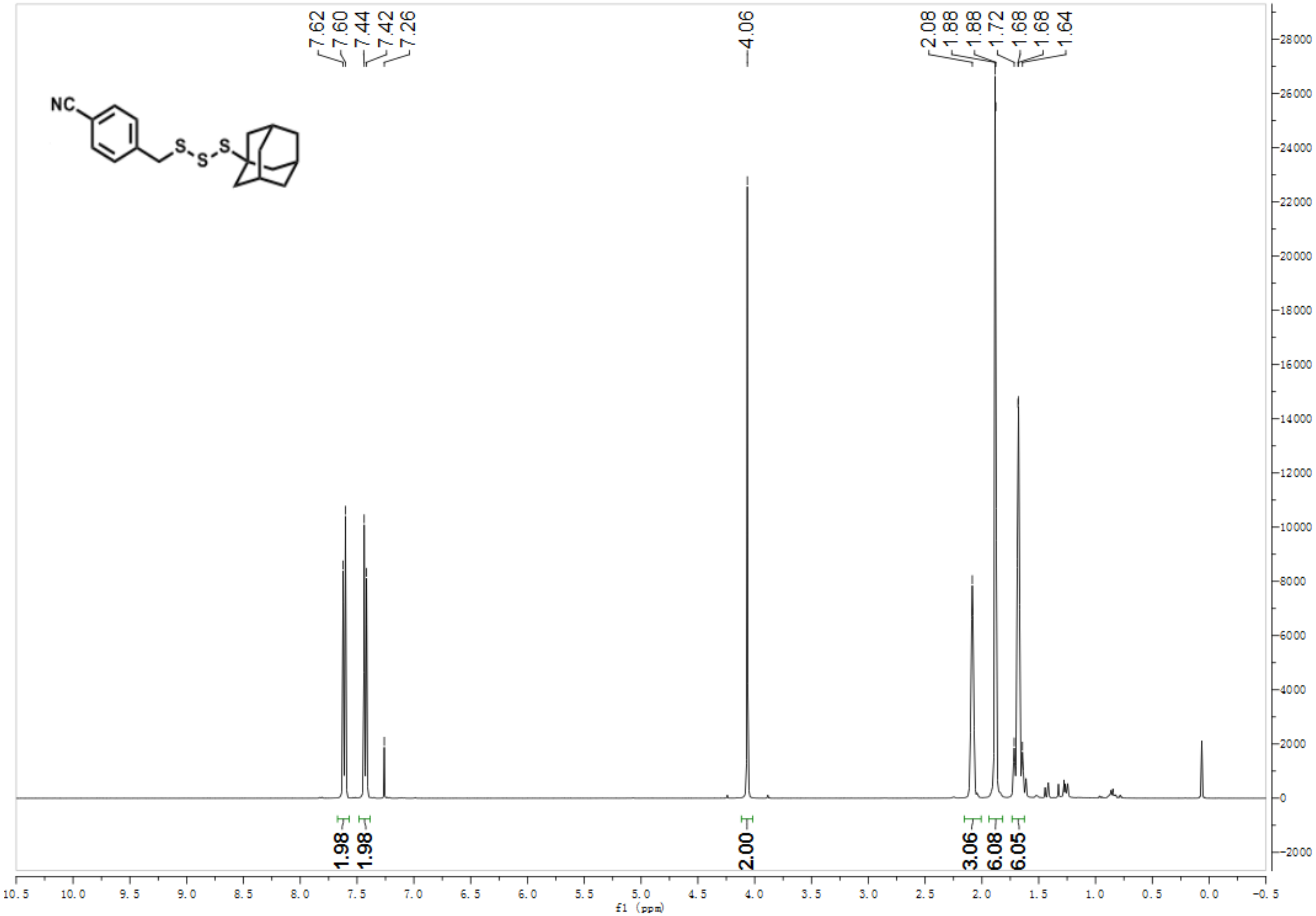

Supplementary Figure 201. ^{1}H NMR spectra for Compound **7l**.

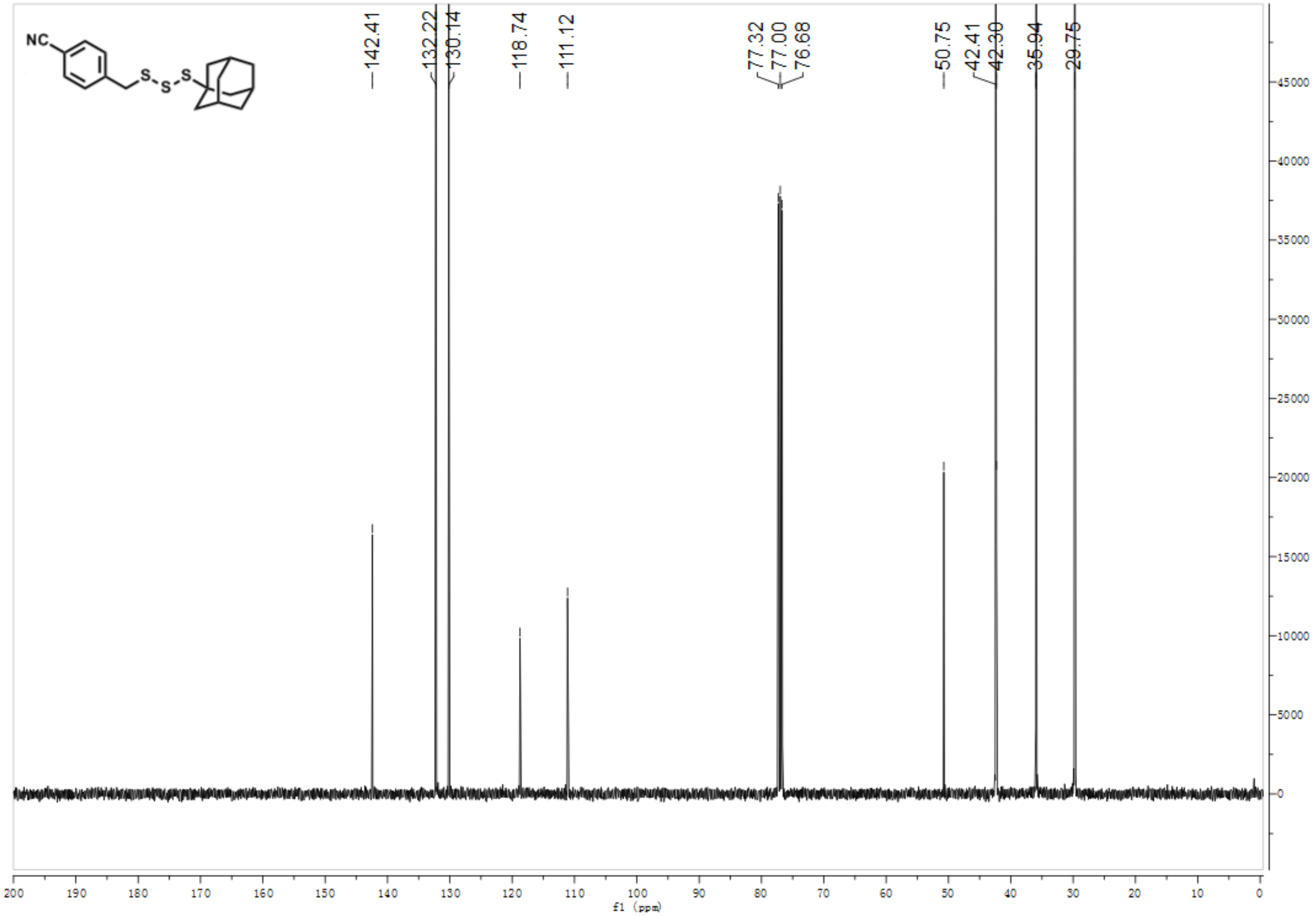

Supplementary Figure 202. ^{13}C NMR spectra for Compound **7l**.

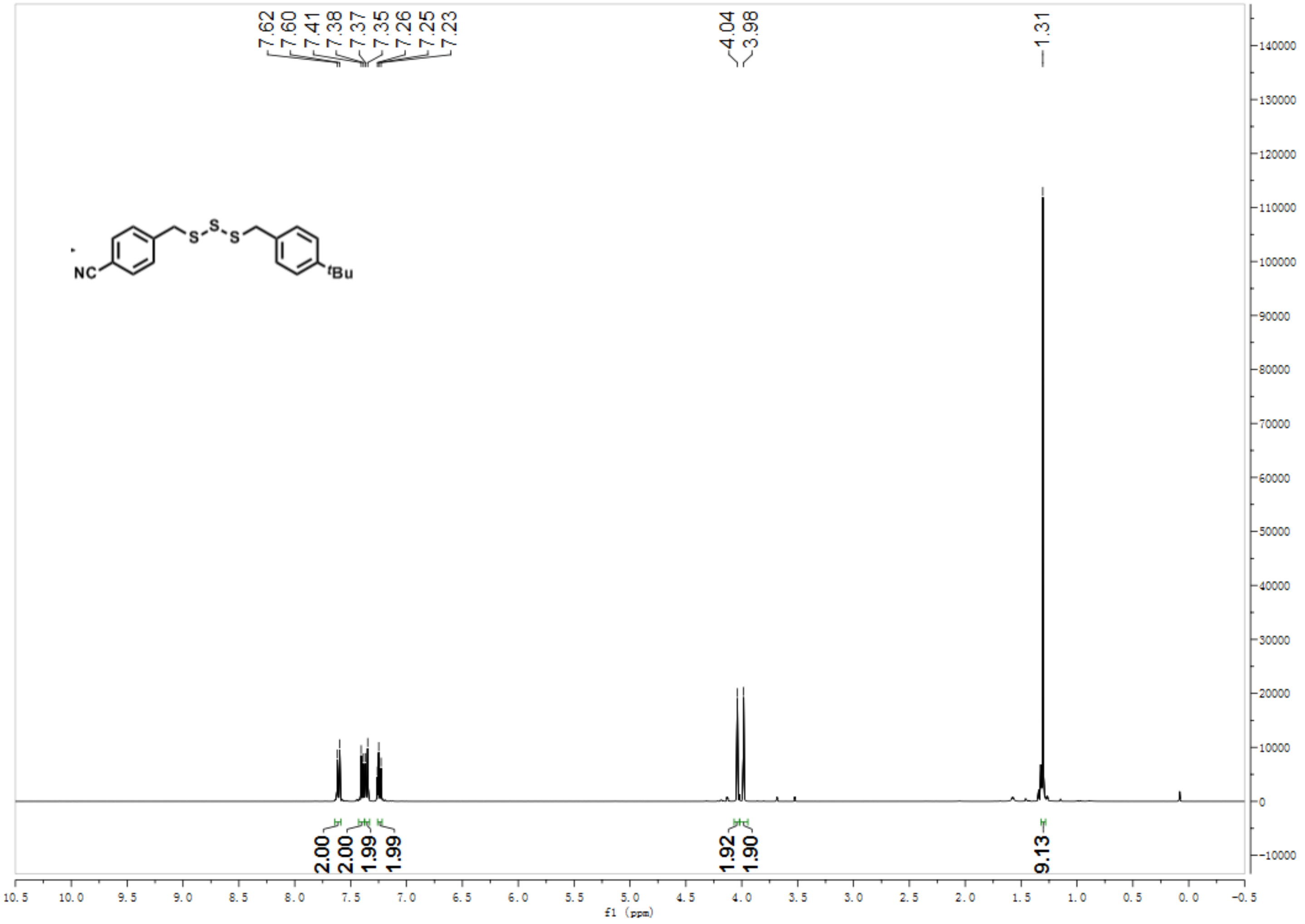

Supplementary Figure 203. ^{1}H NMR spectra for Compound **7m**.

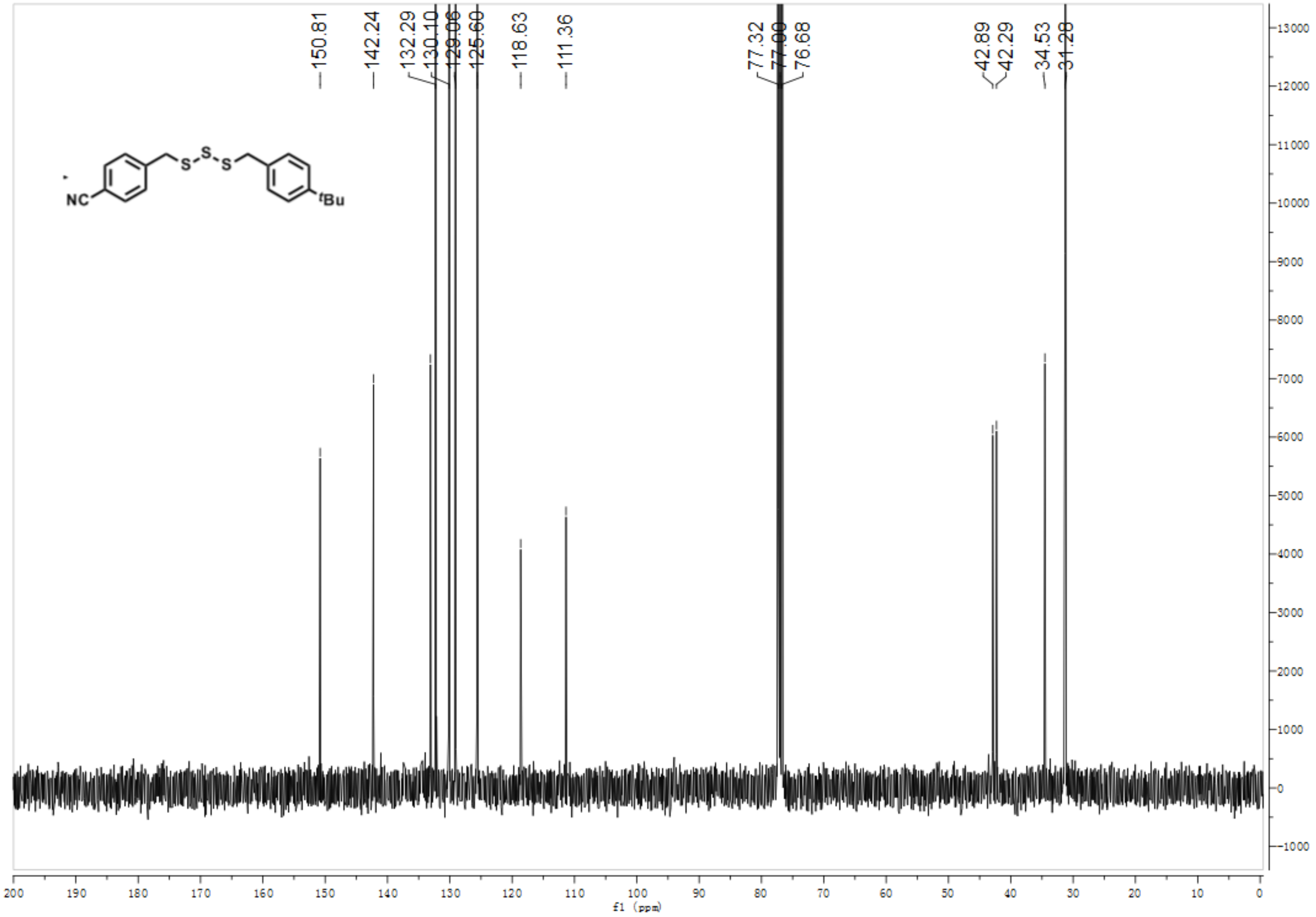

Supplementary Figure 204. ^{13}C NMR spectra for Compound **7m**.

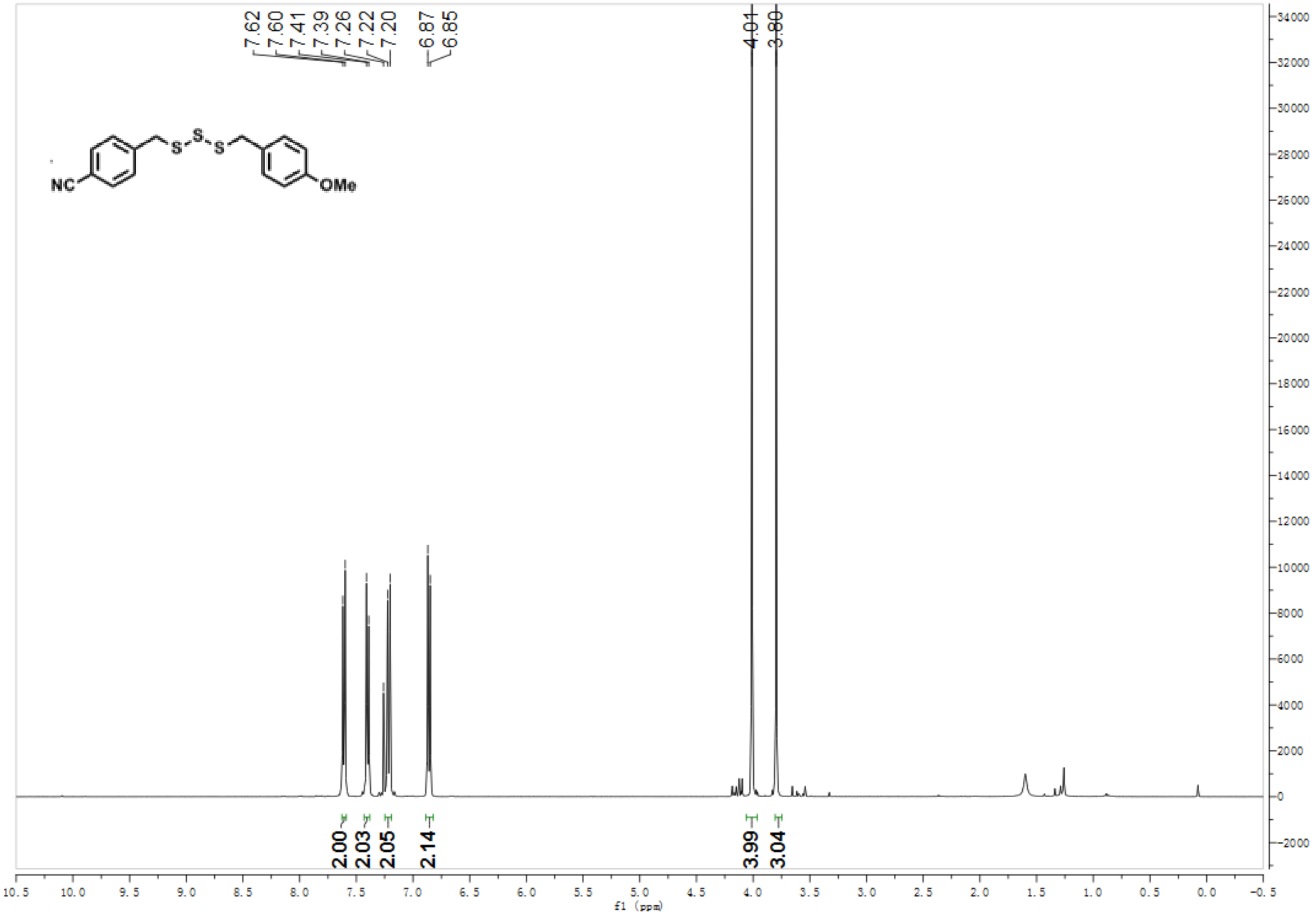

Supplementary Figure 205. ^{1}H NMR spectra for Compound **7n**.

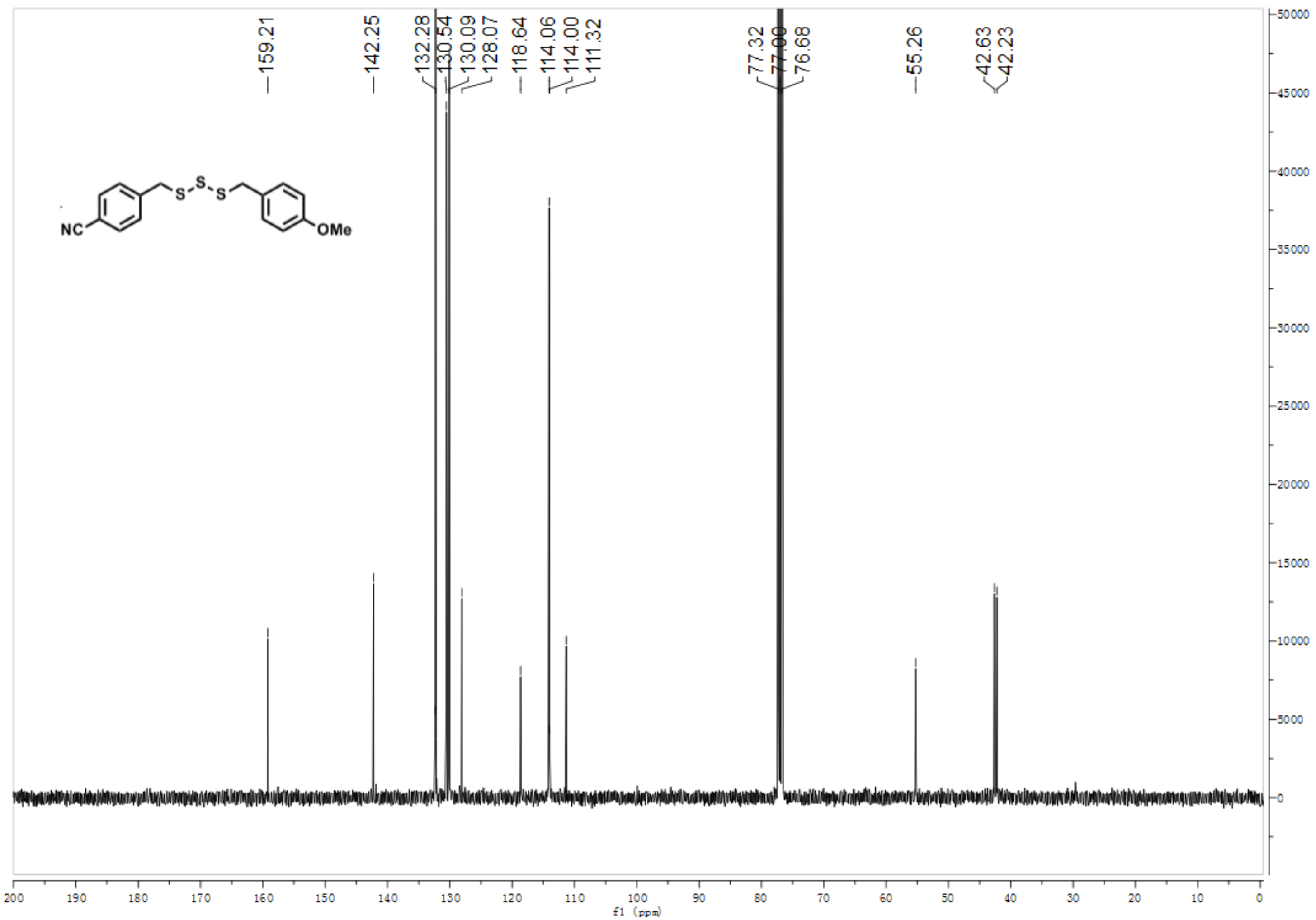

Supplementary Figure 206. ^{13}C NMR spectra for Compound **7n**.

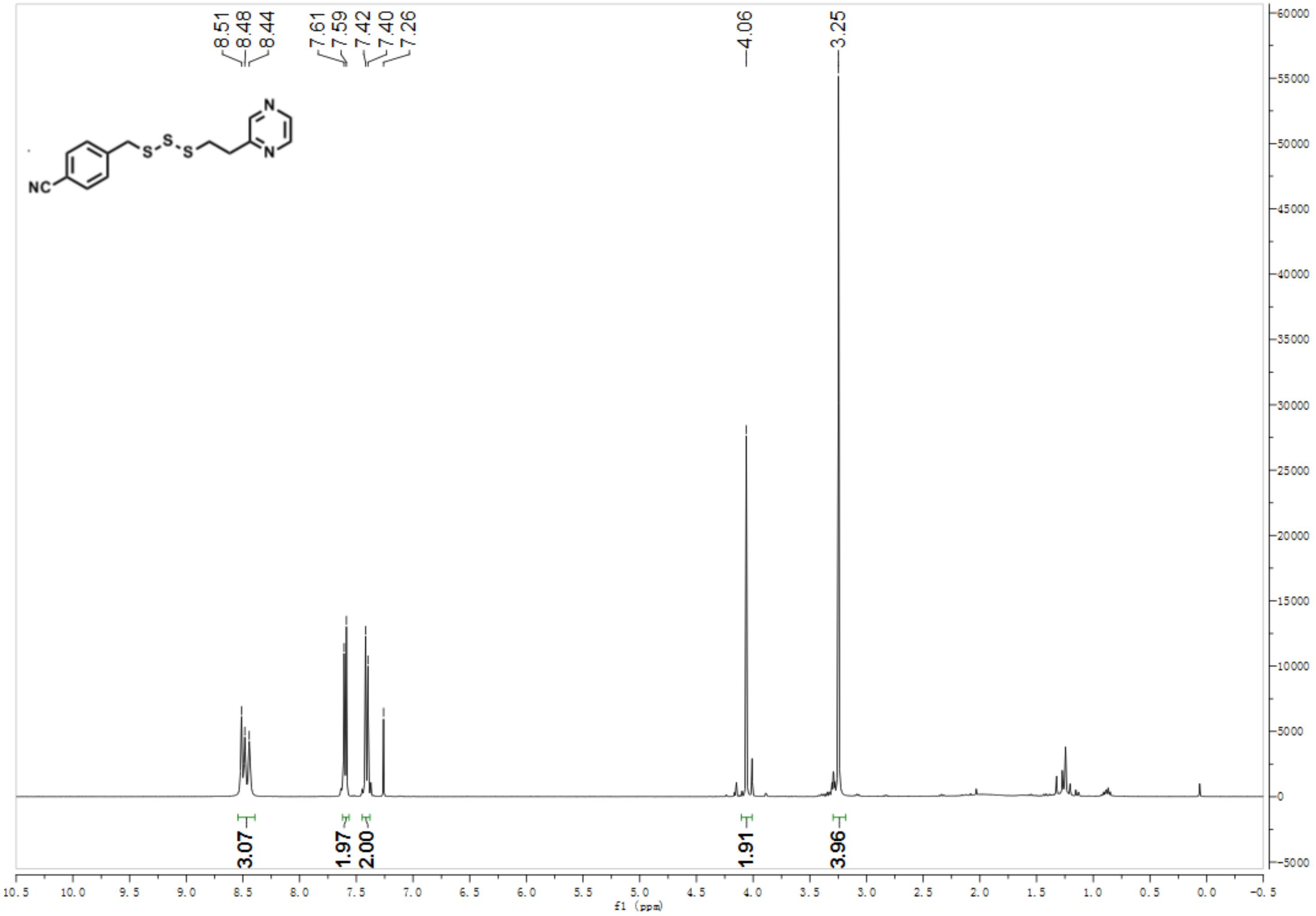

Supplementary Figure 207. ^{1}H NMR spectra for Compound **7o**.

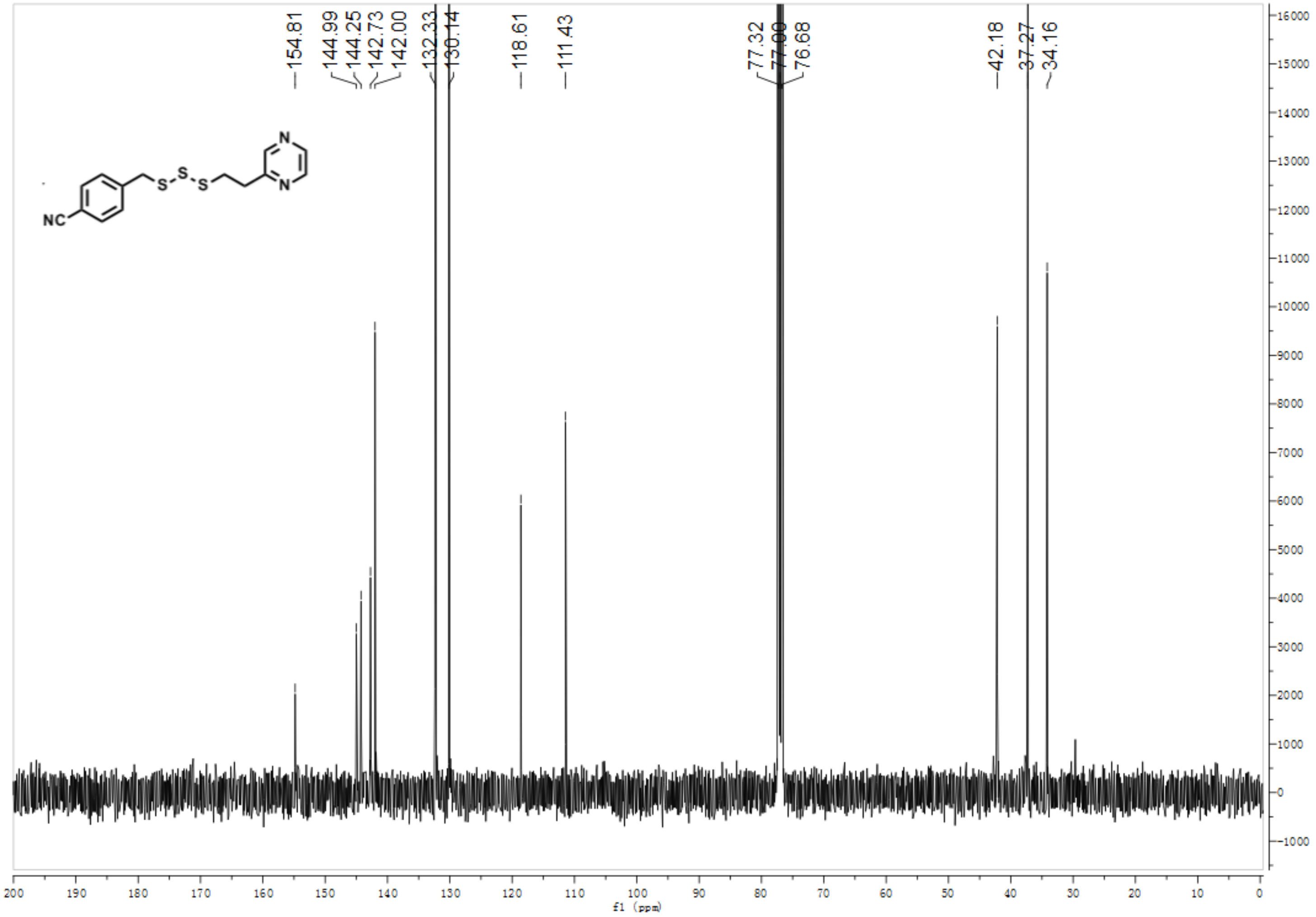

Supplementary Figure 208. ^{13}C NMR spectra for Compound **7o**.

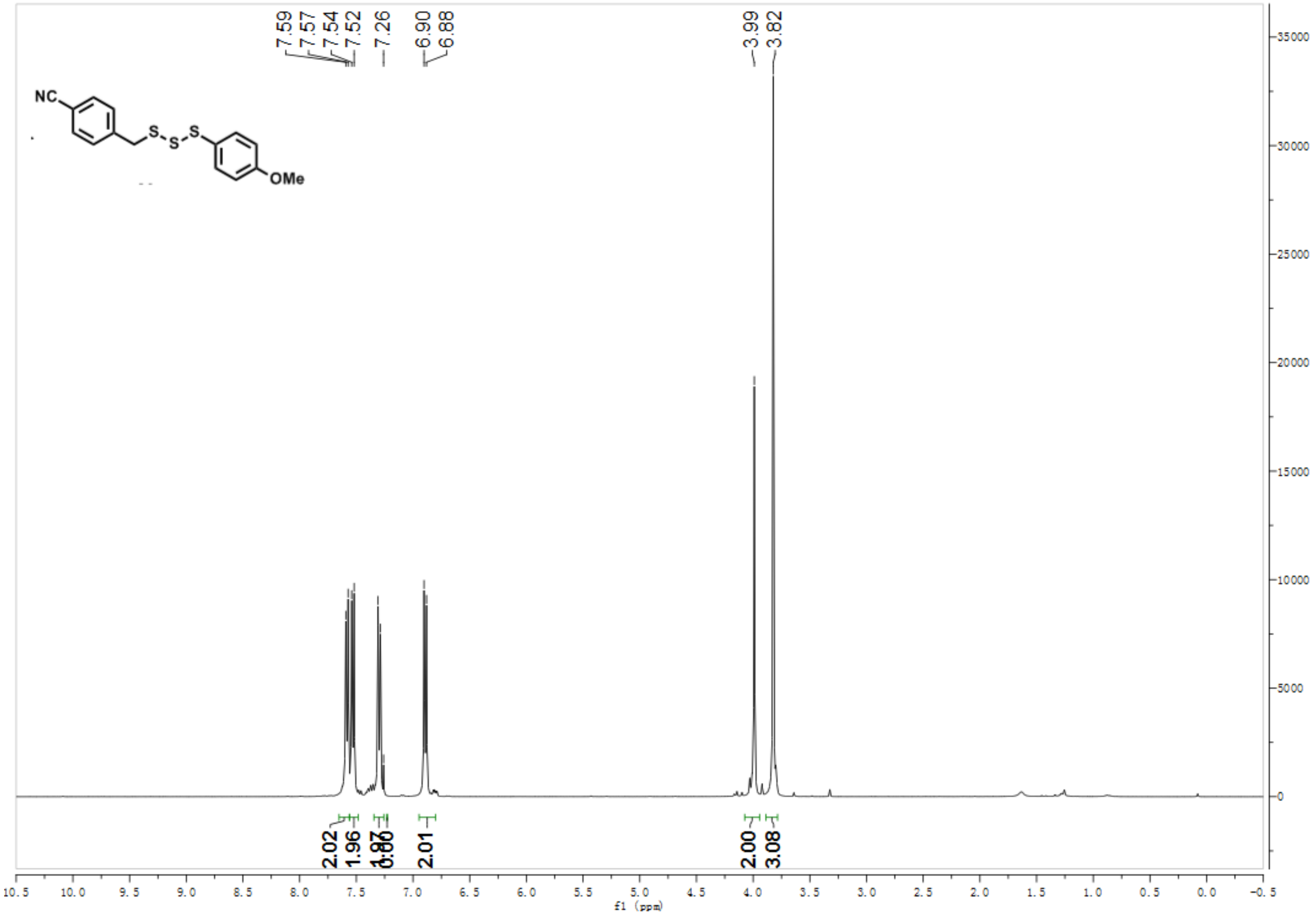

Supplementary Figure 209. ^{1}H NMR spectra for Compound **7p**.

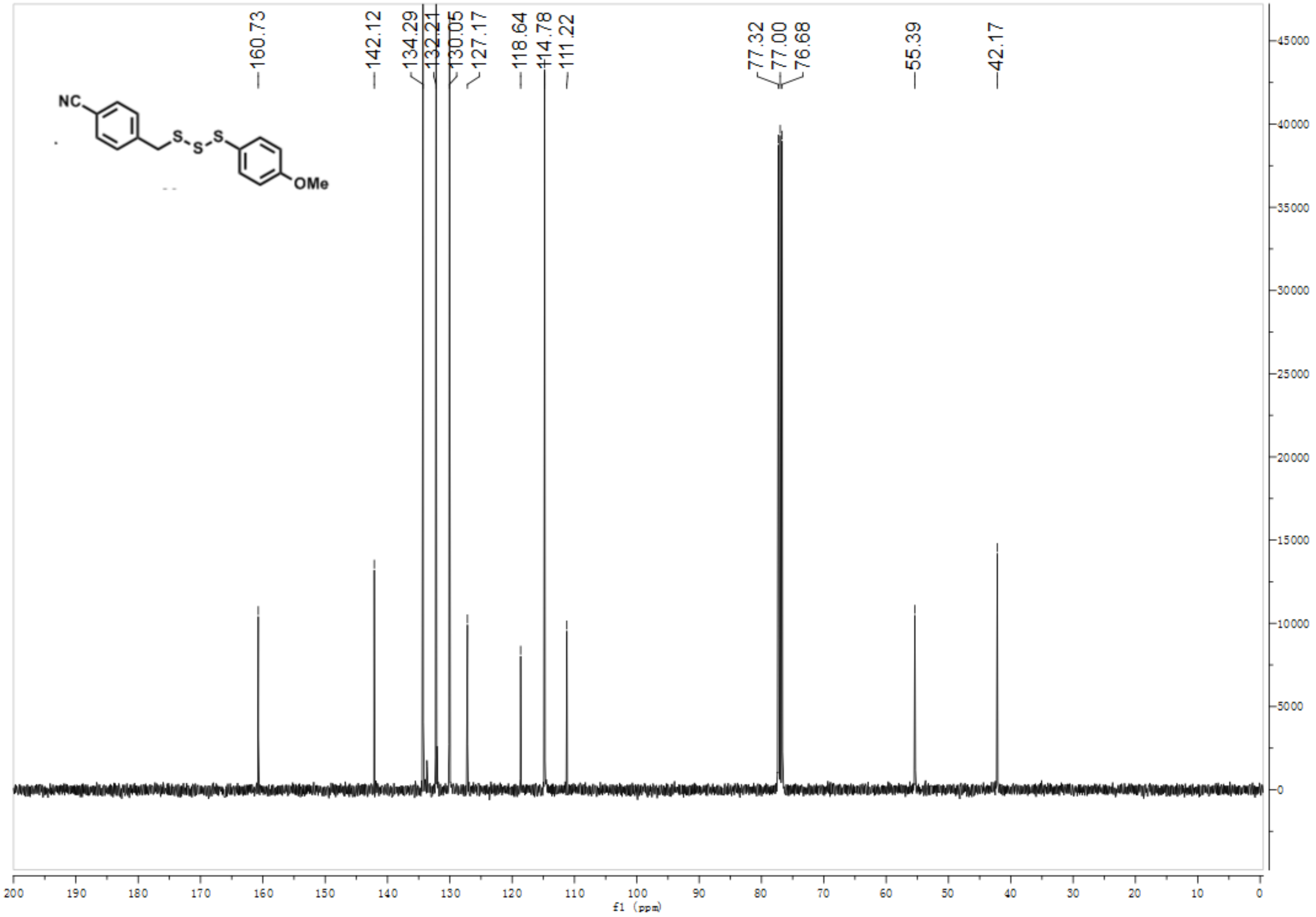

Supplementary Figure 210. ^{13}C NMR spectra for Compound **7p**.

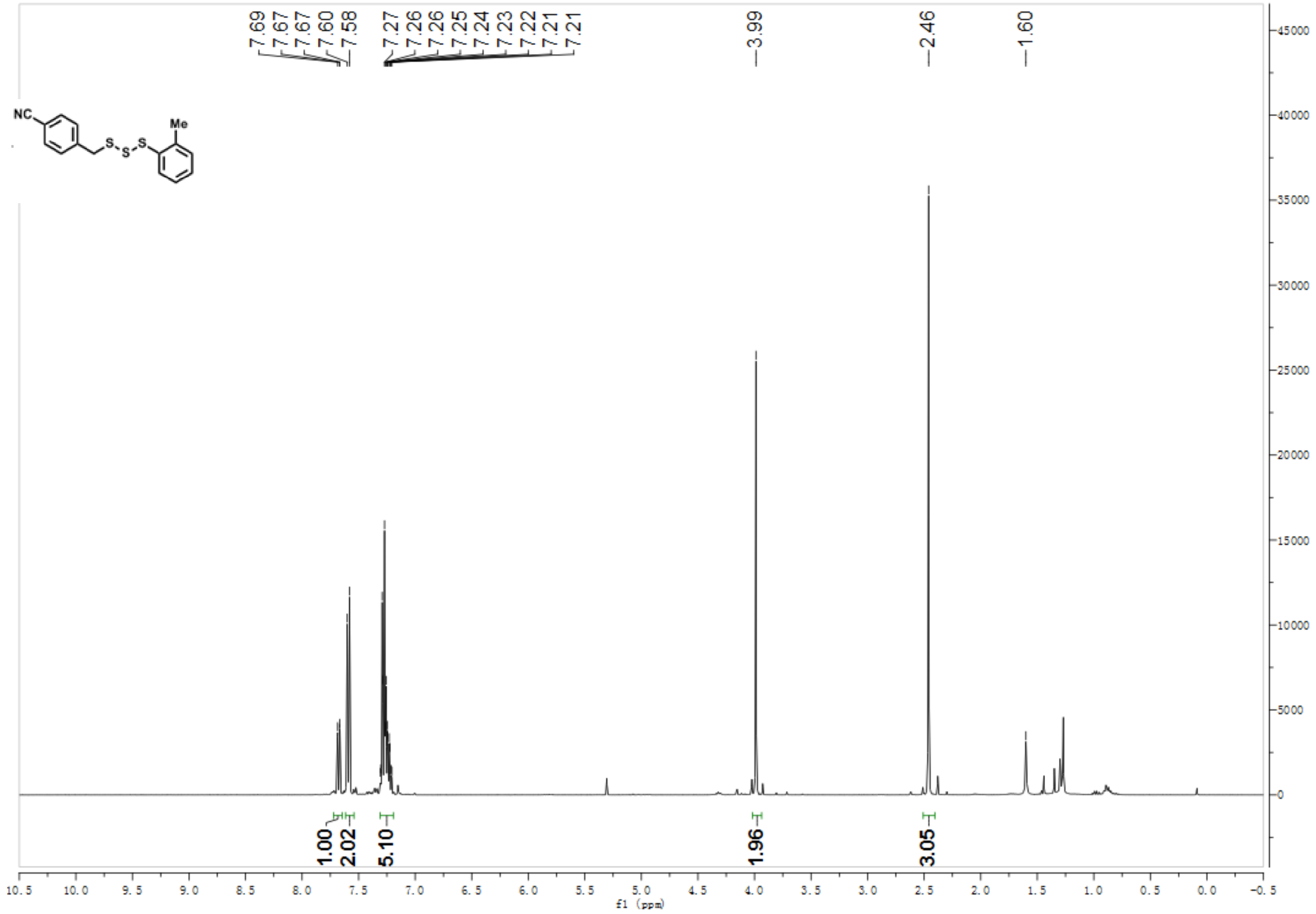

Supplementary Figure 211. ^{1}H NMR spectra for Compound **7q**.

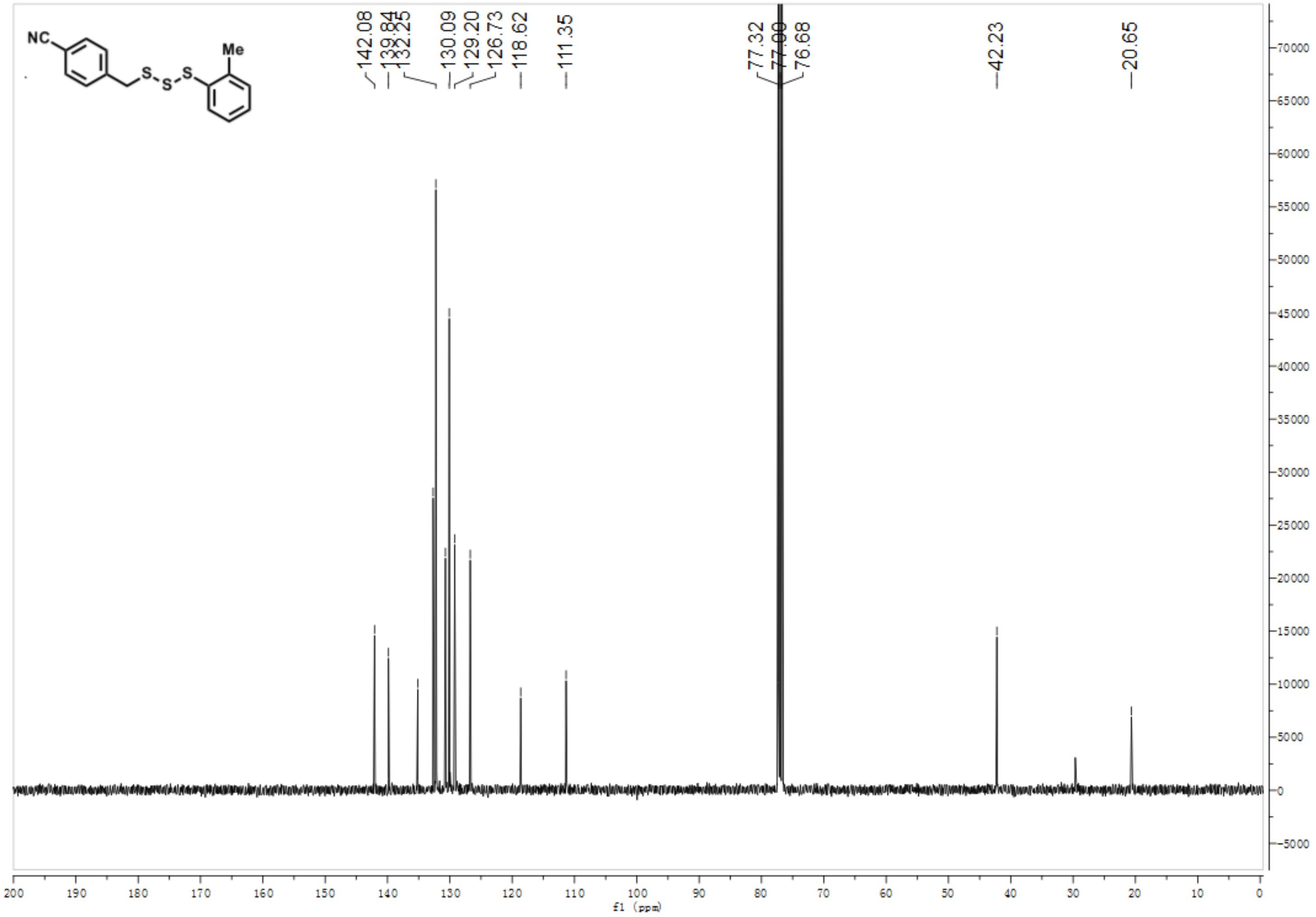

Supplementary Figure 212. ^{13}C NMR spectra for Compound **7q**.

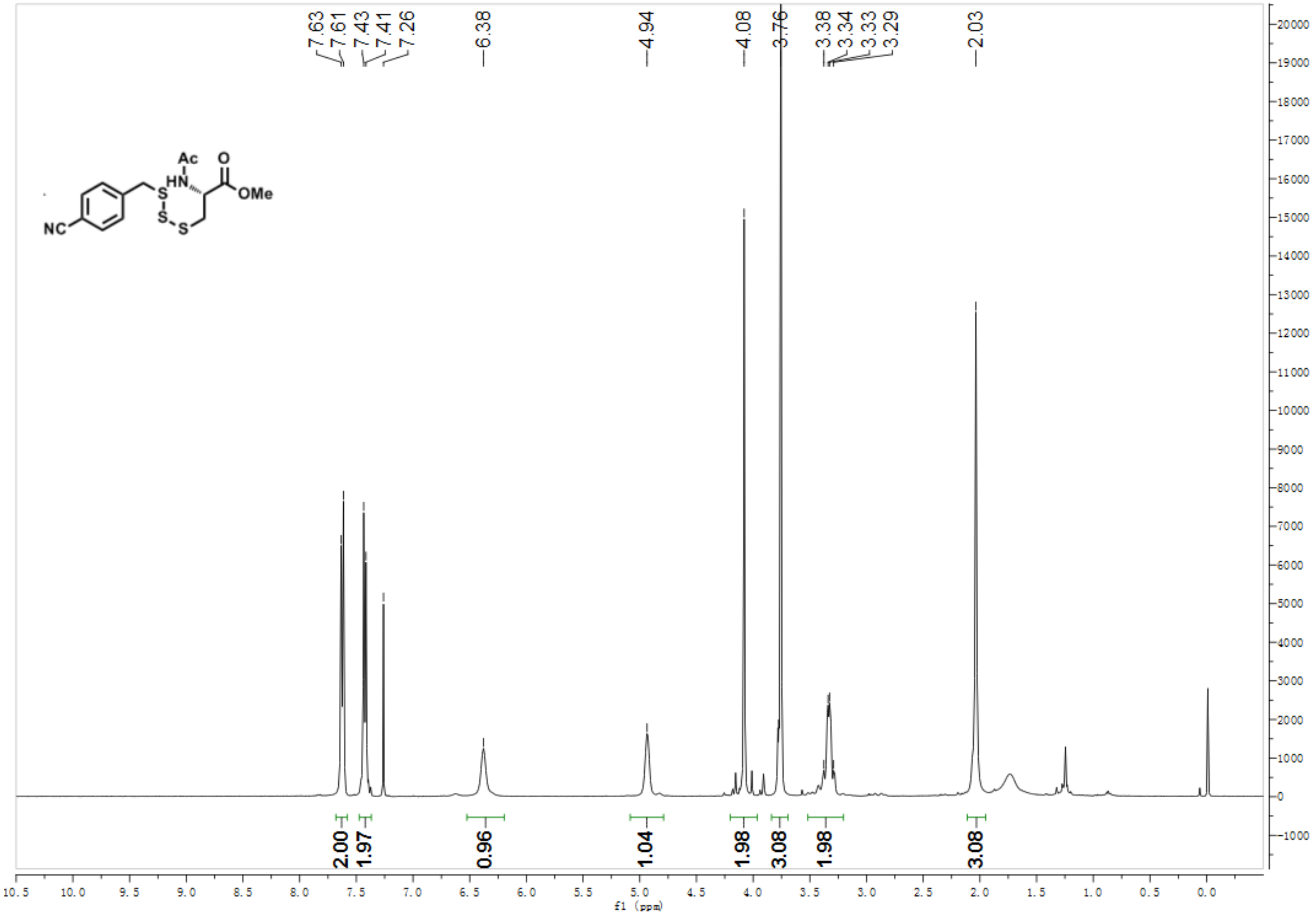

Supplementary Figure 213. ^{1}H NMR spectra for Compound **7r**.

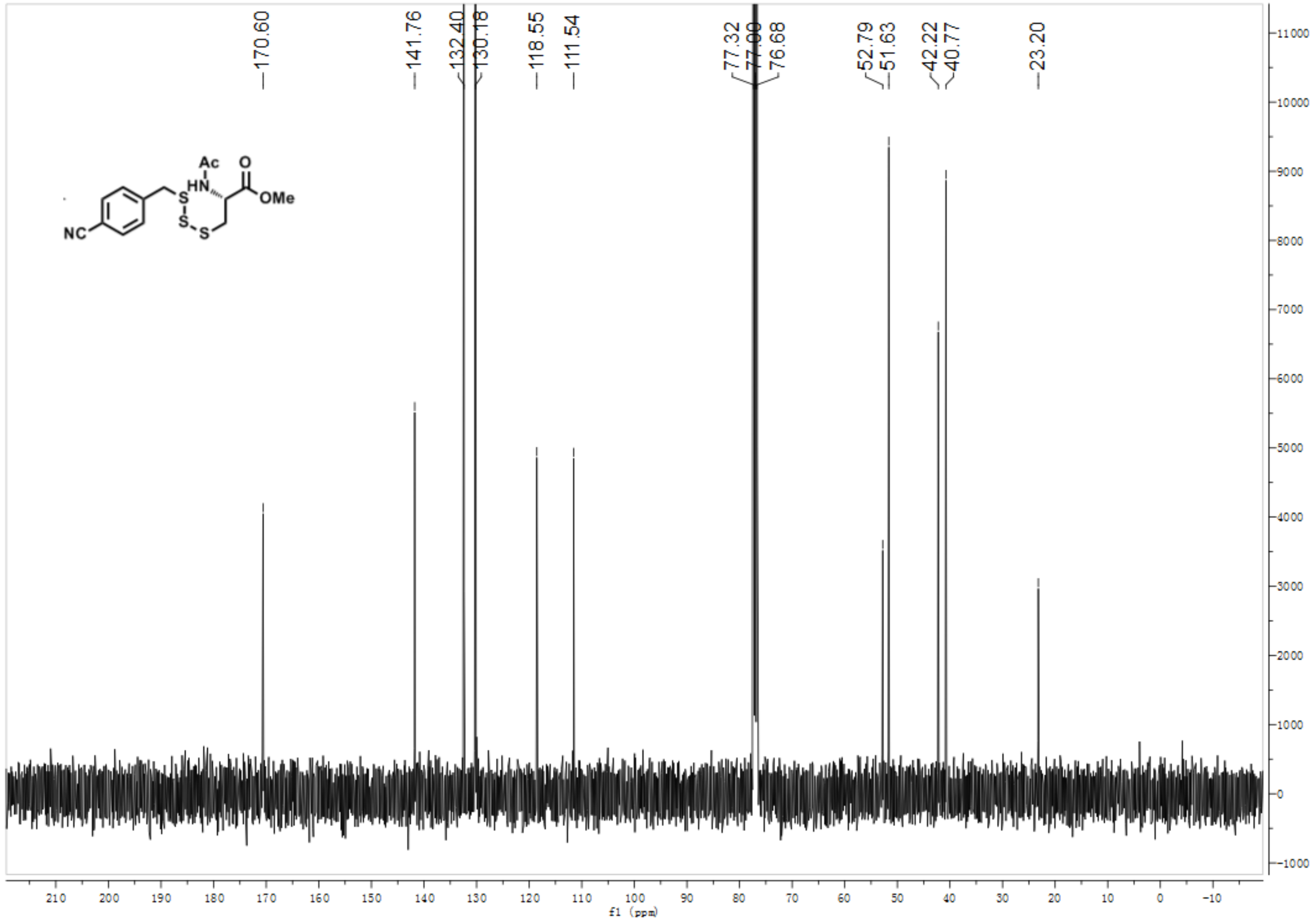

Supplementary Figure 214. ^{13}C NMR spectra for Compound **7r**.

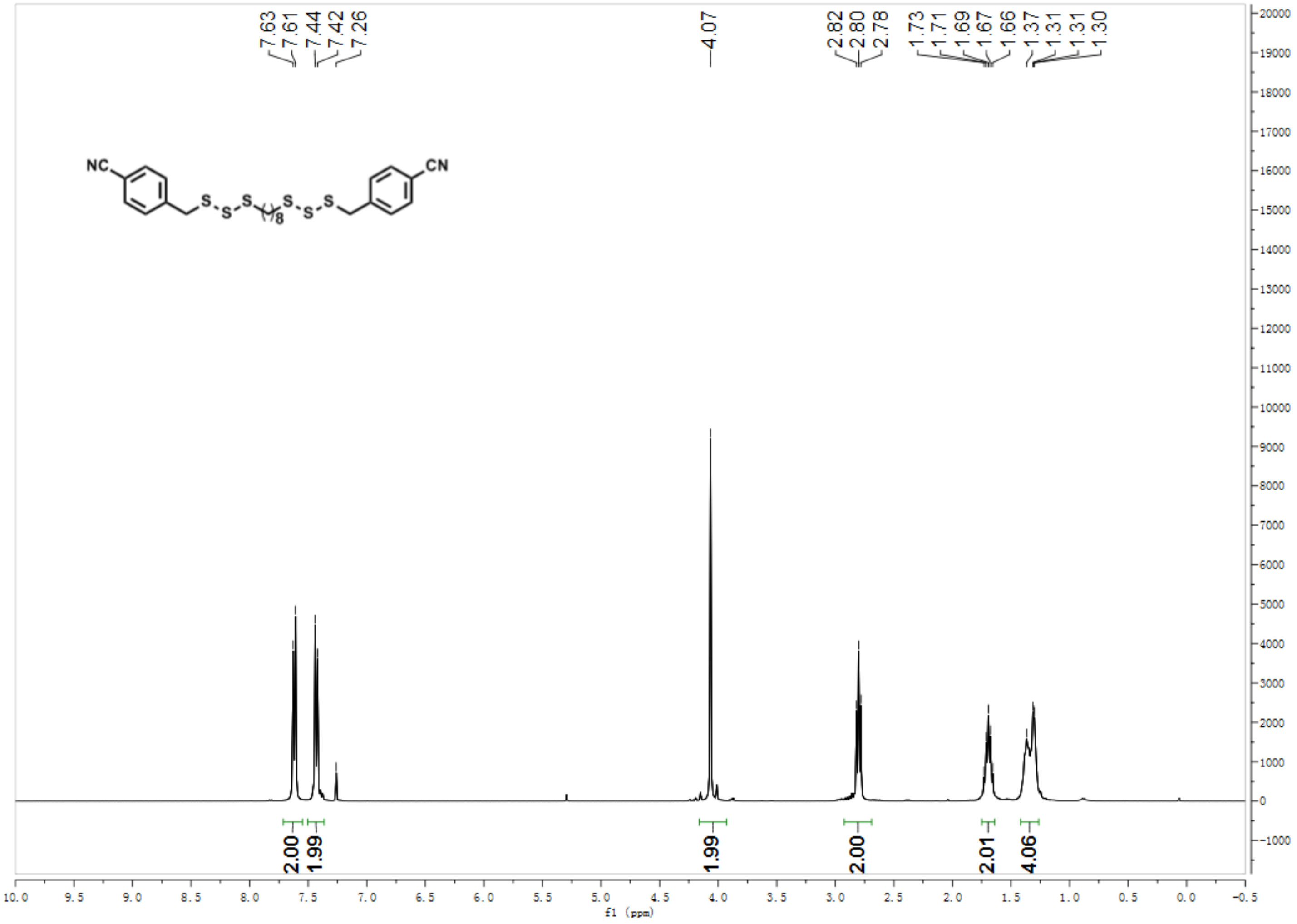

Supplementary Figure 215. ^{1}H NMR spectra for Compound **7s**.

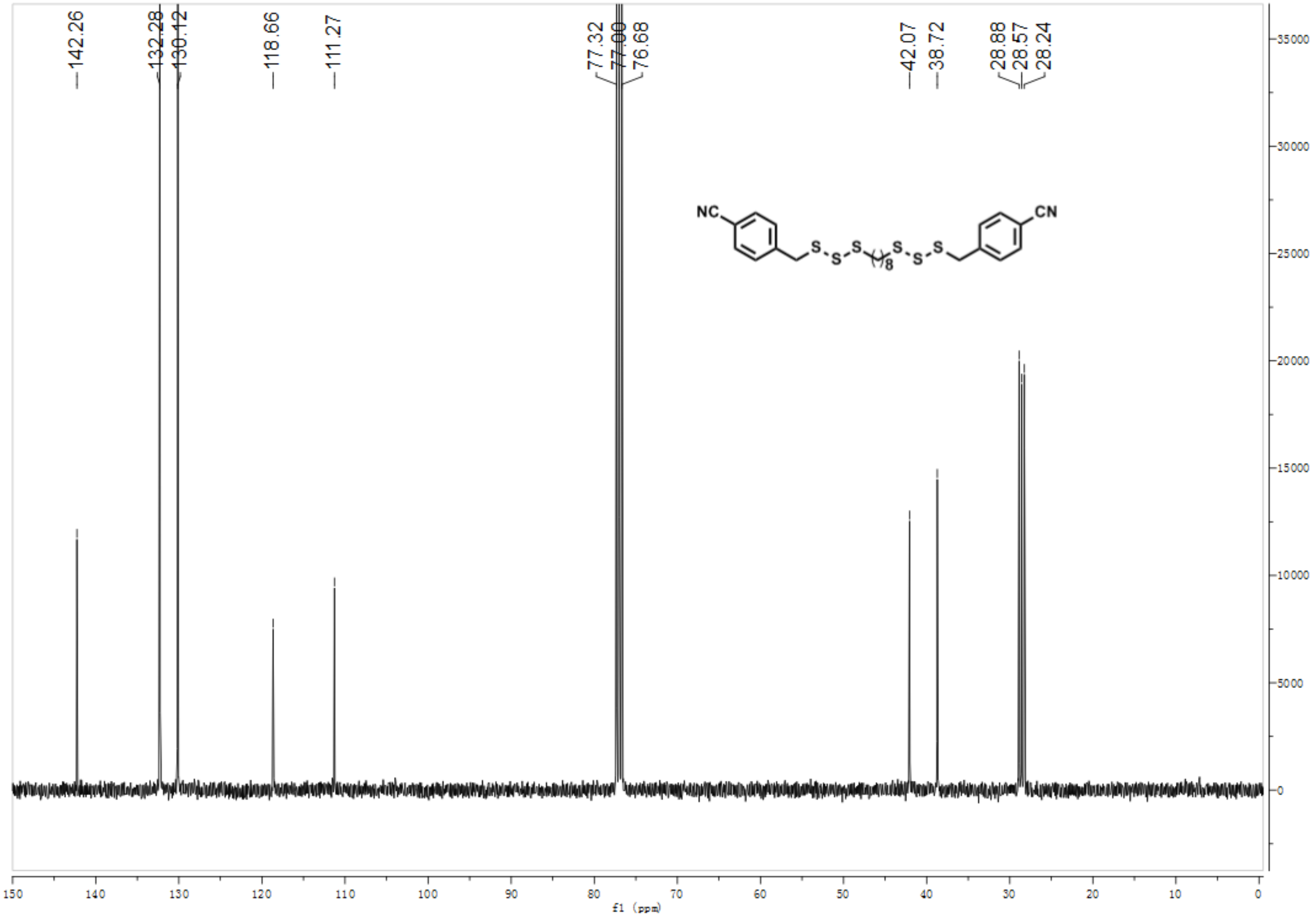

Supplementary Figure 216. ^{13}C NMR spectra for Compound **7s**.

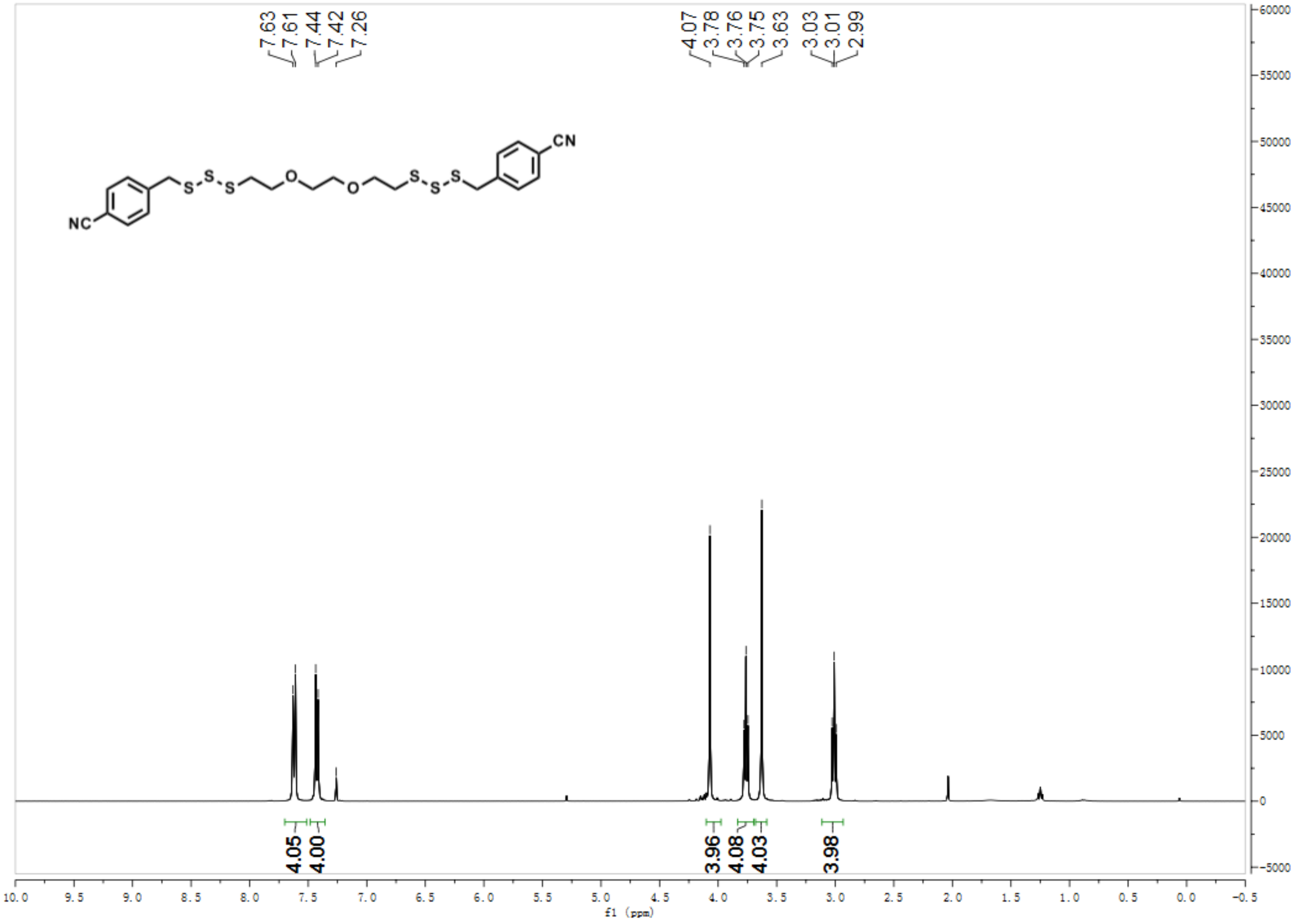

Supplementary Figure 217. ^{1}H NMR spectra for Compound **7t**.

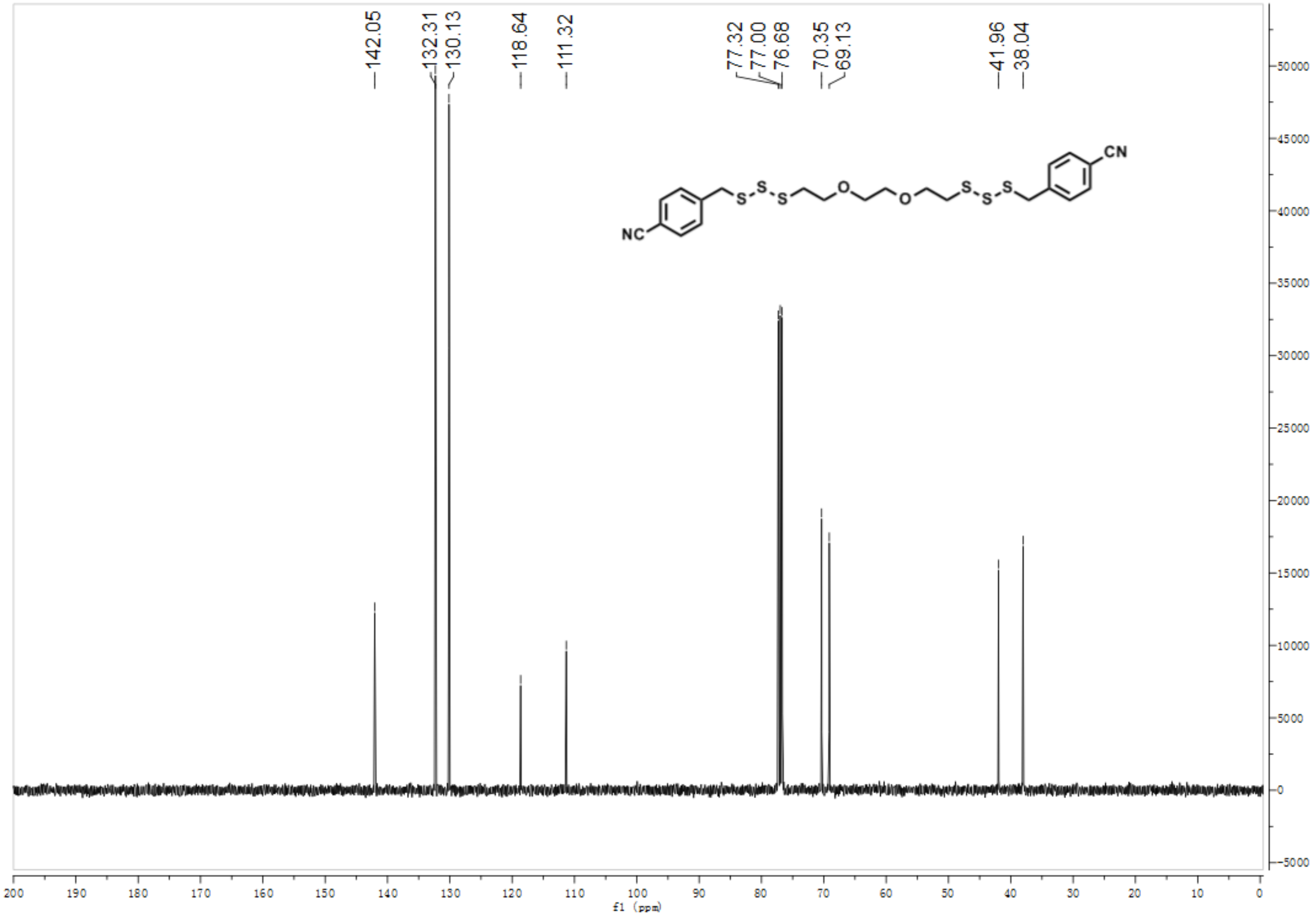

Supplementary Figure 218. ^{13}C NMR spectra for Compound **7t**.

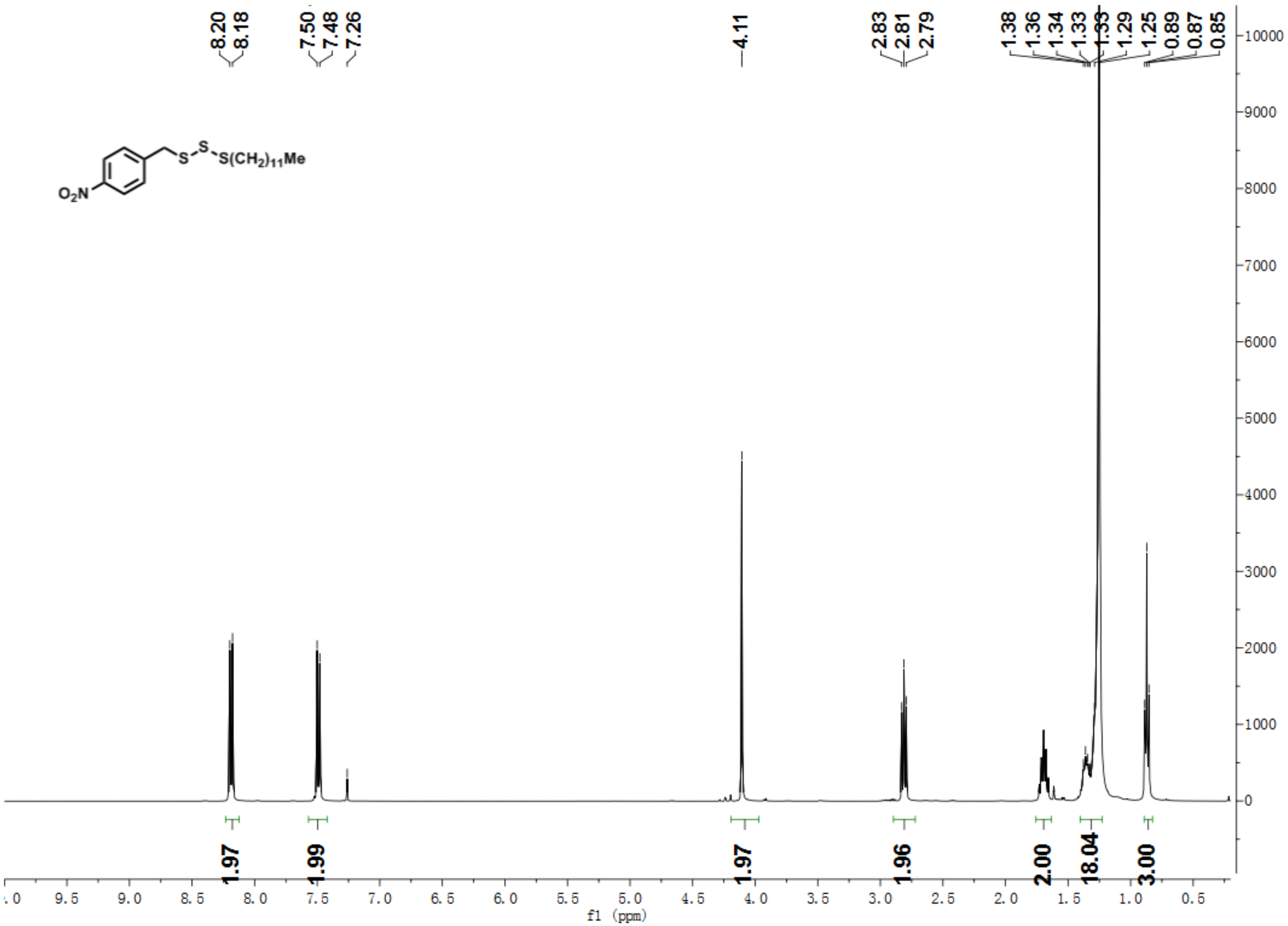

Supplementary Figure 219. ^{1}H NMR spectra for Compound **7u**.

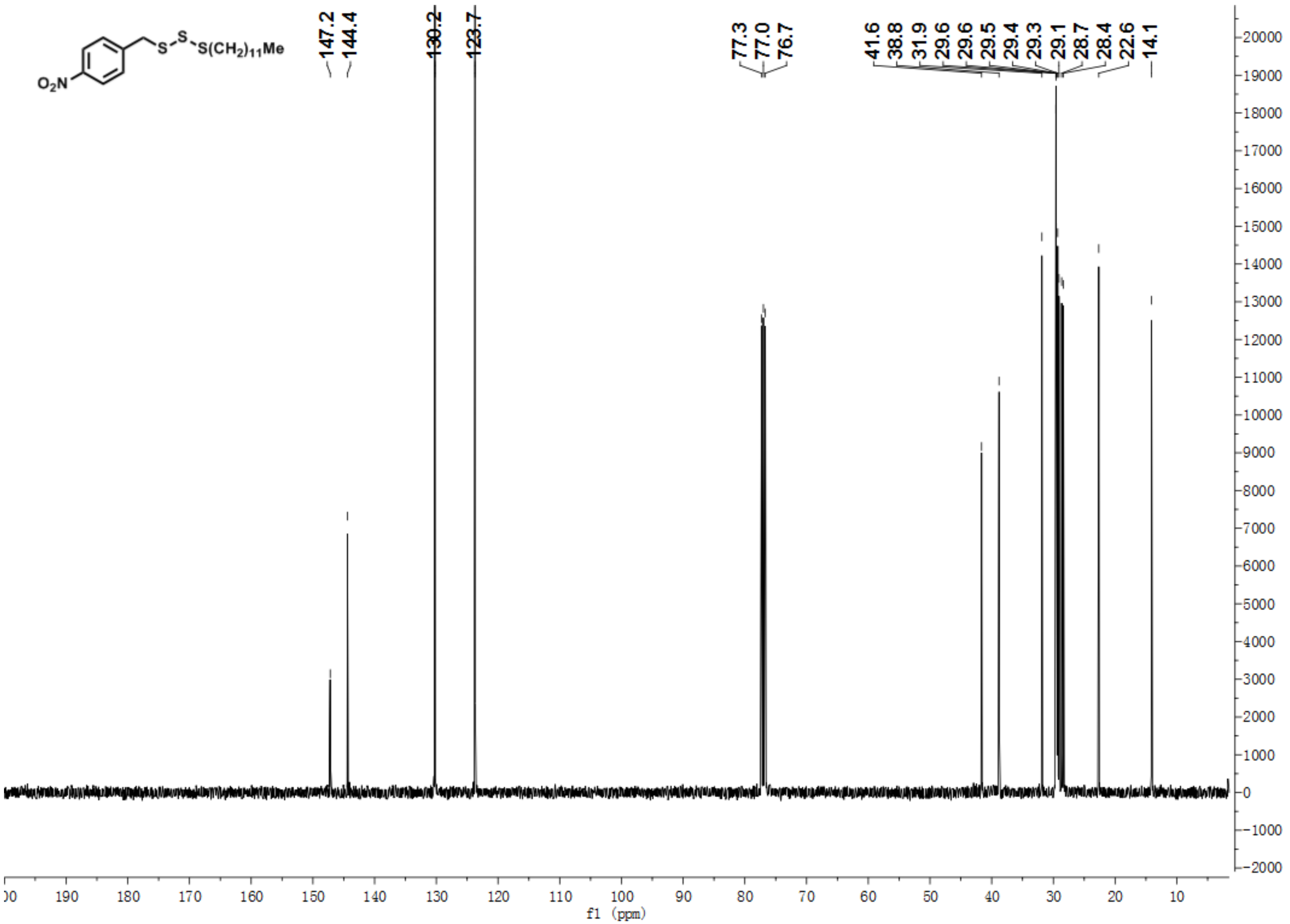

Supplementary Figure 220. ^{13}C NMR spectra for Compound **7u**.

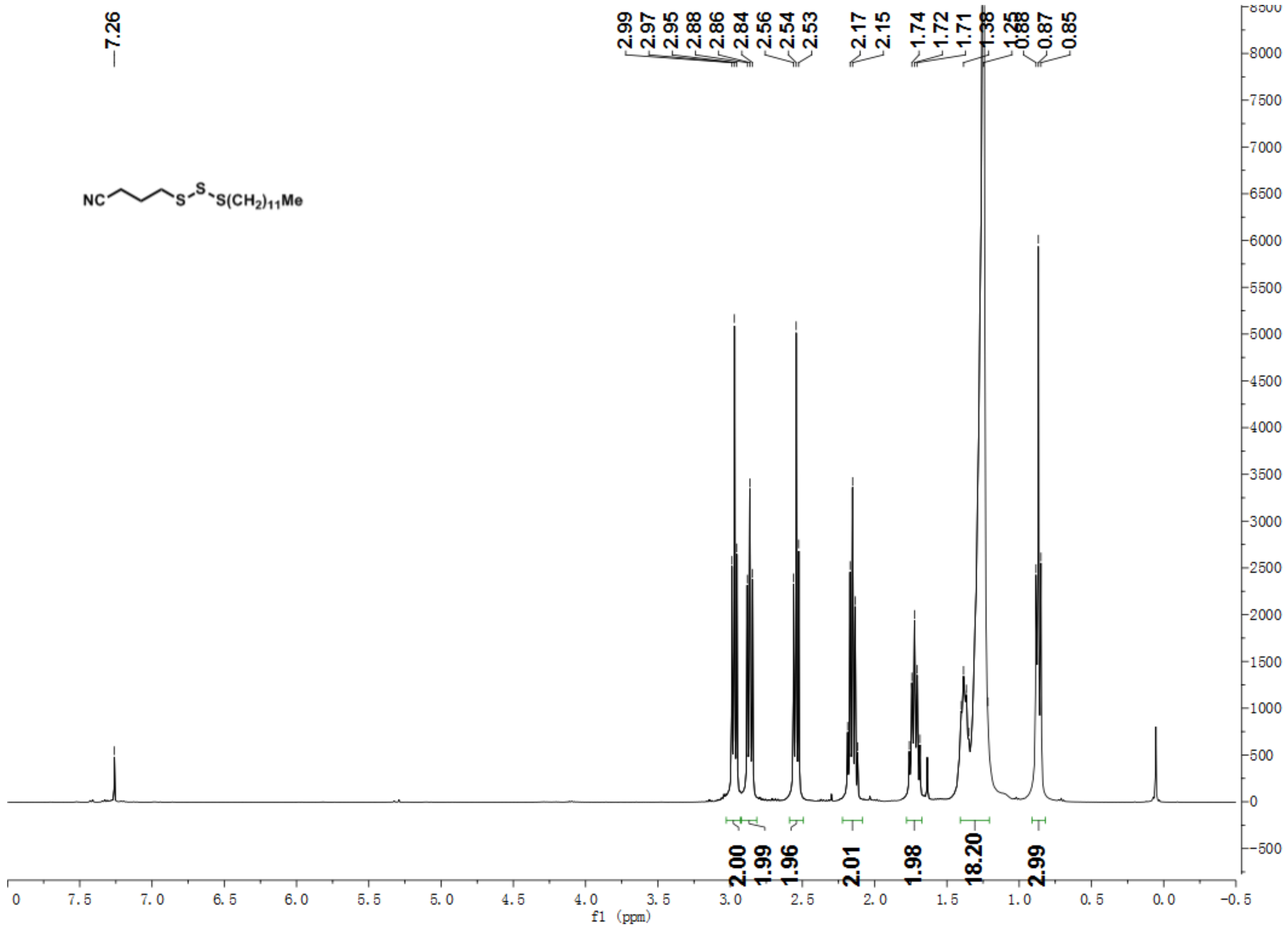

Supplementary Figure 221. ^{1}H NMR spectra for Compound **7v**.

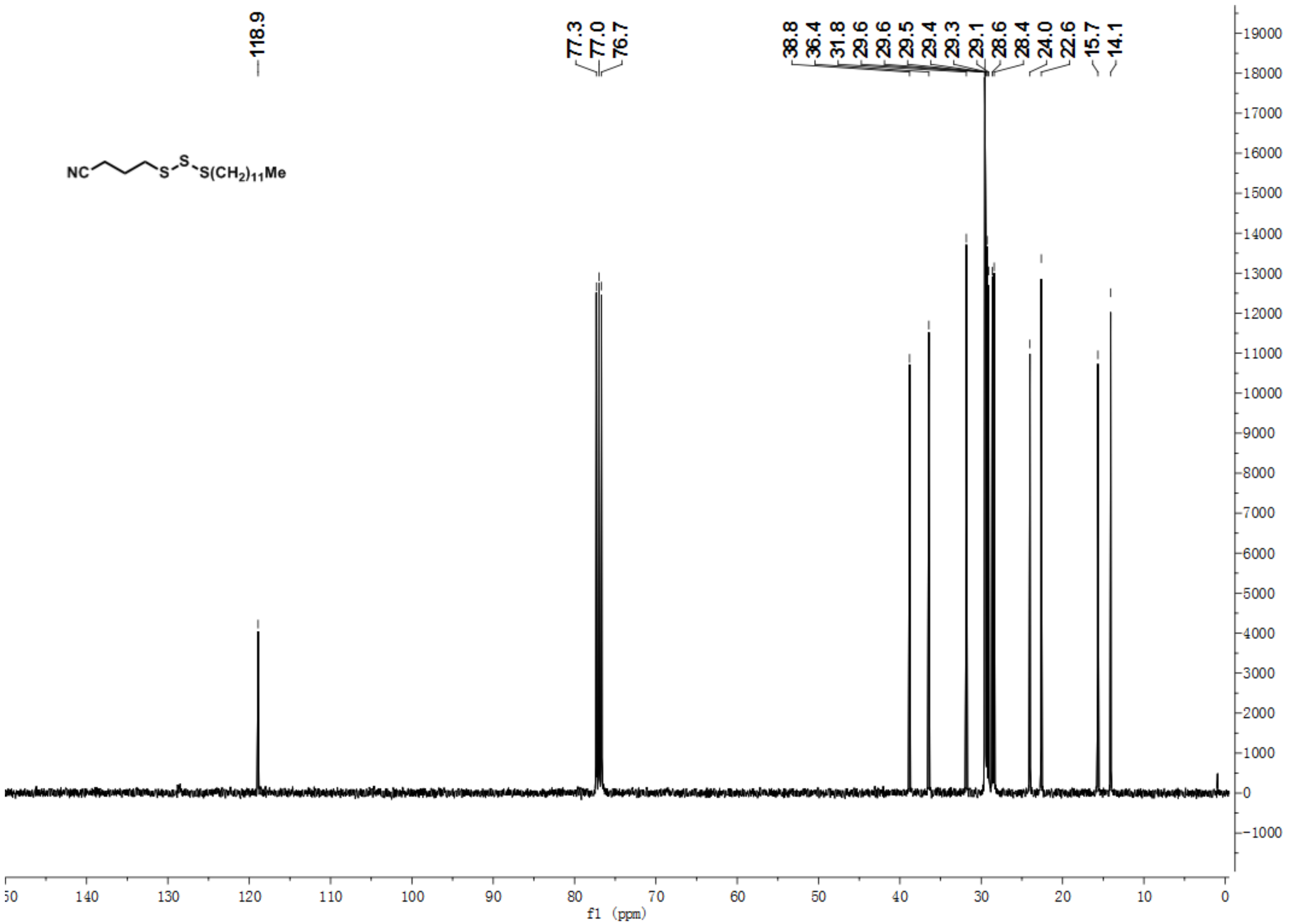

Supplementary Figure 222. ^{13}C NMR spectra for Compound **7v**.

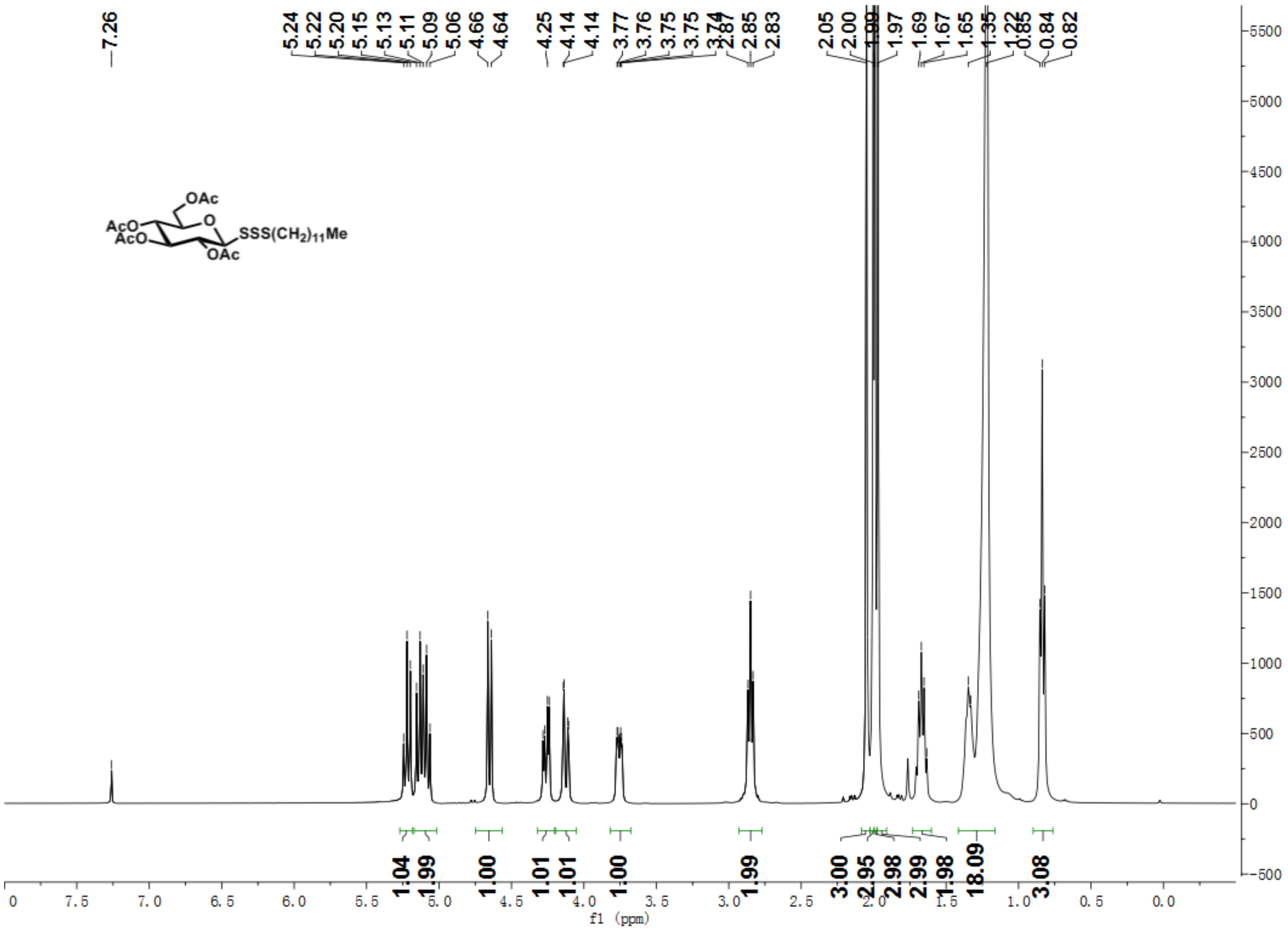

Supplementary Figure 223. ^{1}H NMR spectra for Compound **7w**.

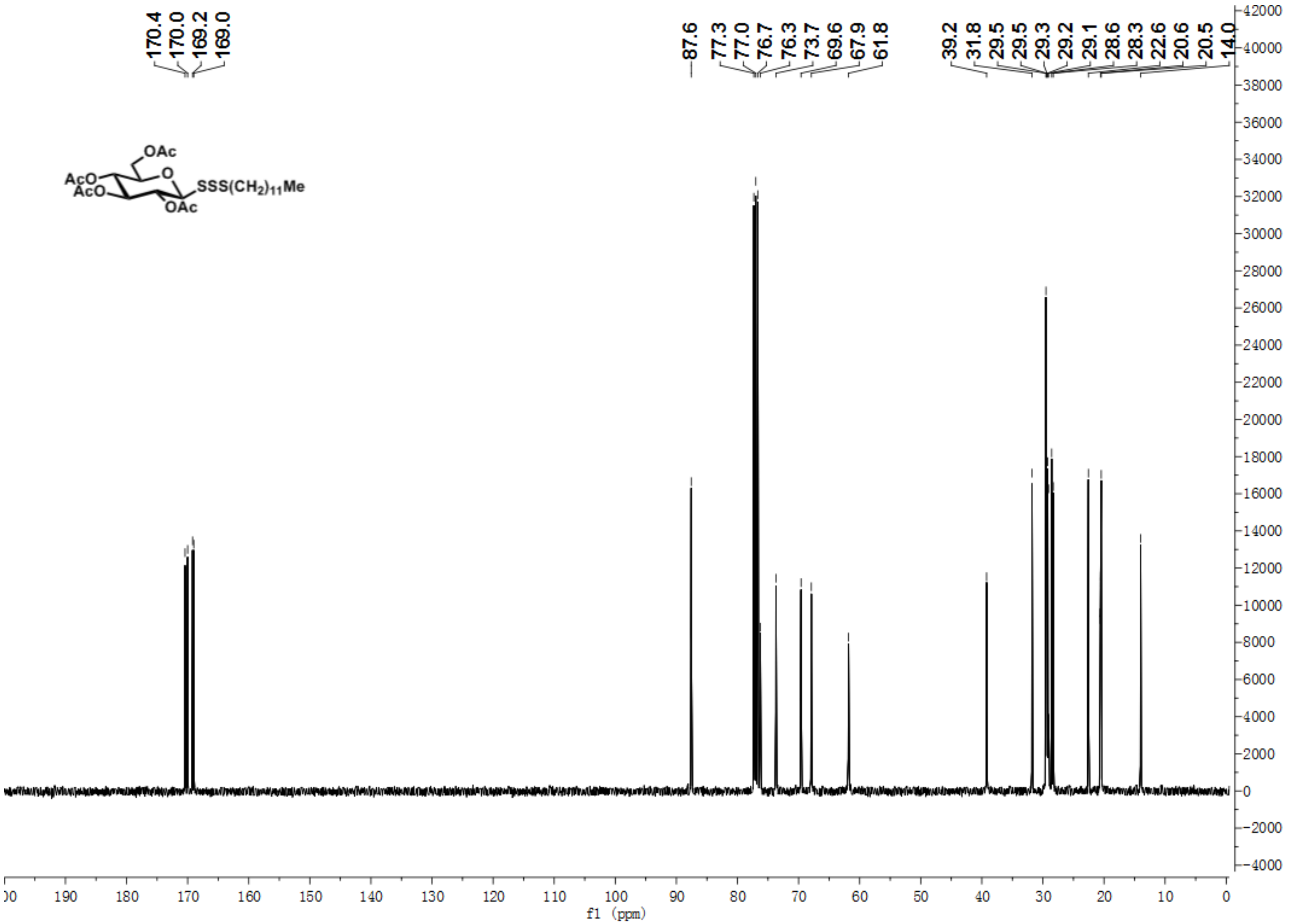

Supplementary Figure 224. ^{13}C NMR spectra for Compound **7w**.

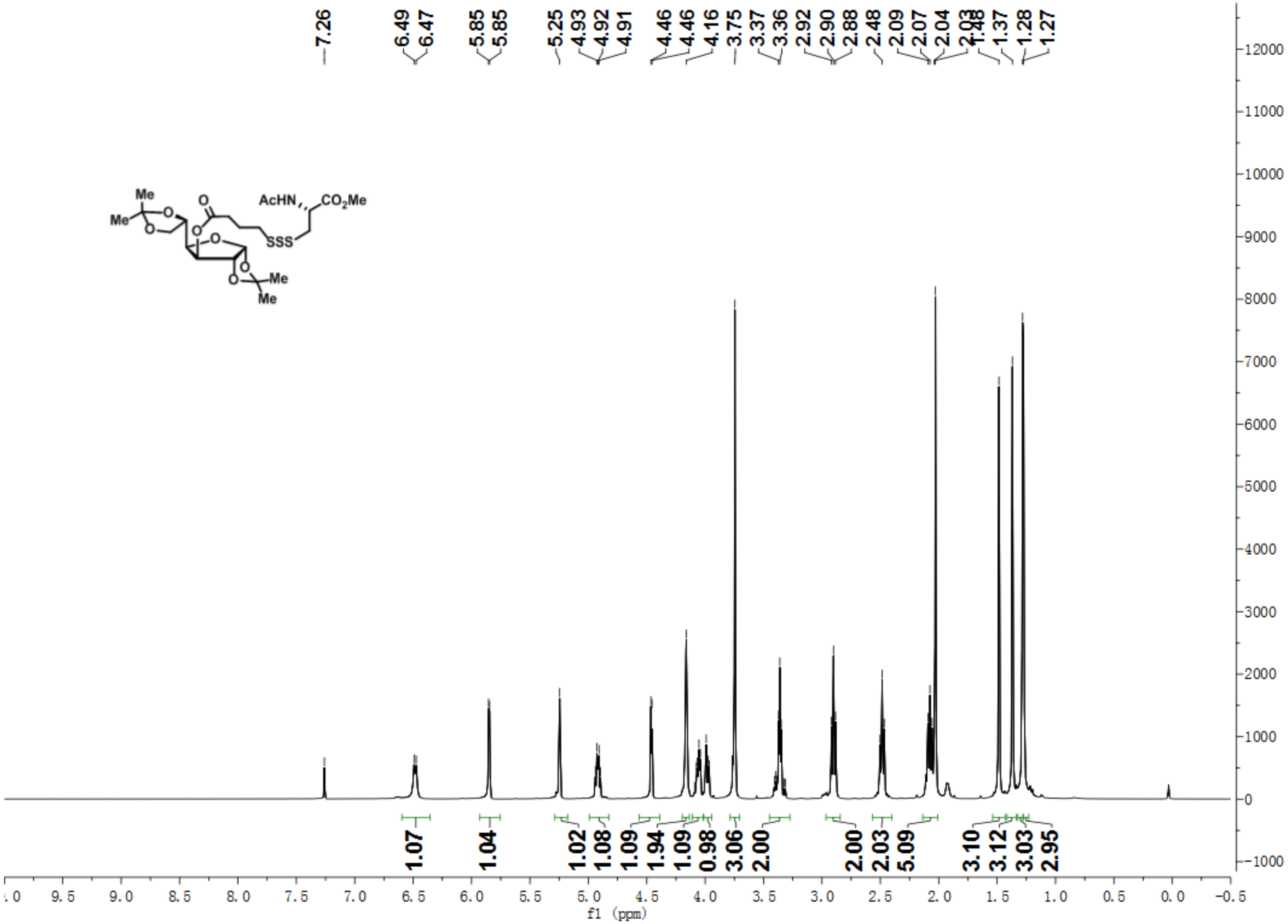

Supplementary Figure 225. ^{1}H NMR spectra for Compound **7x**.

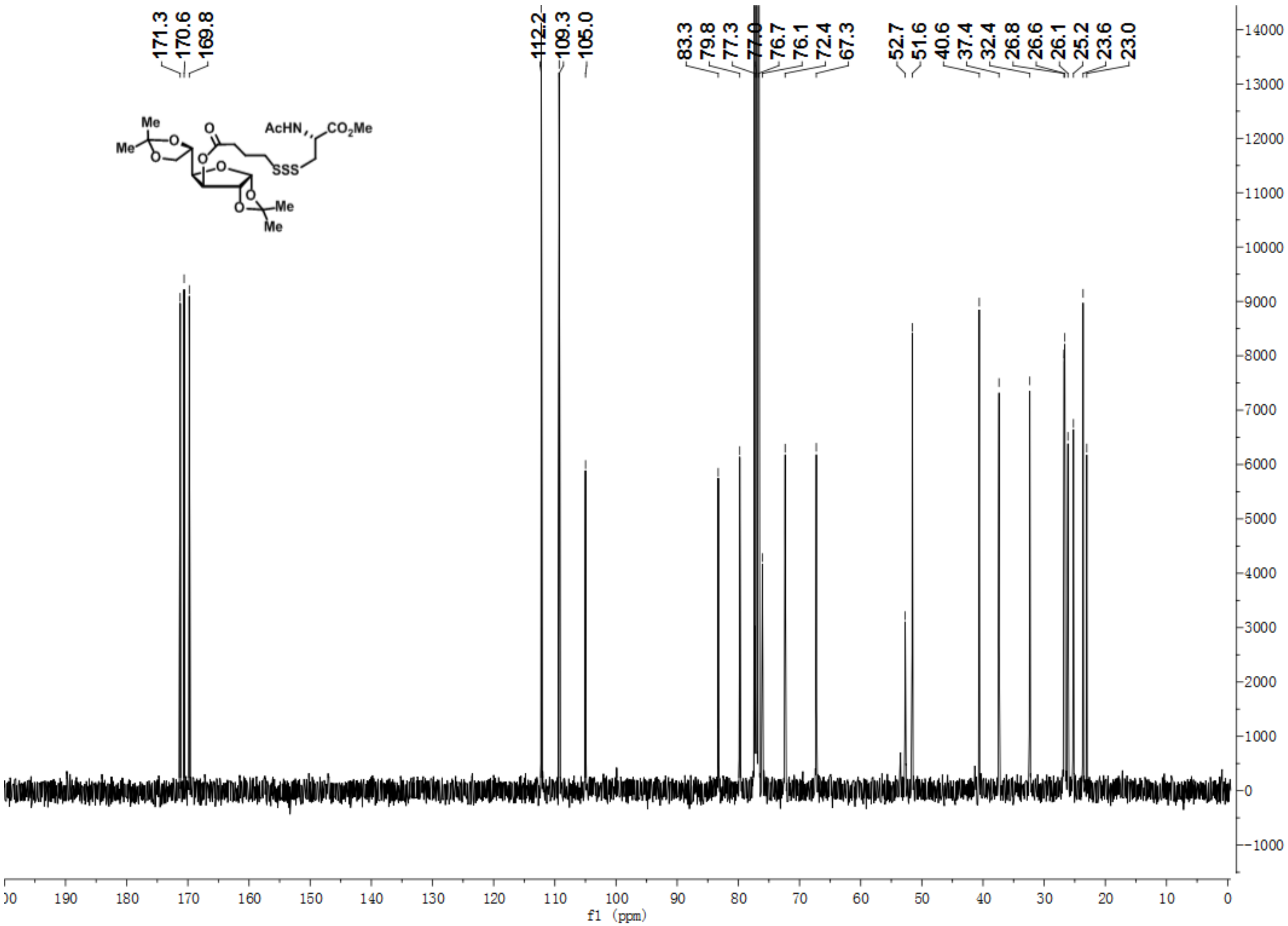

Supplementary Figure 226. ^{13}C NMR spectra for Compound **7x**.

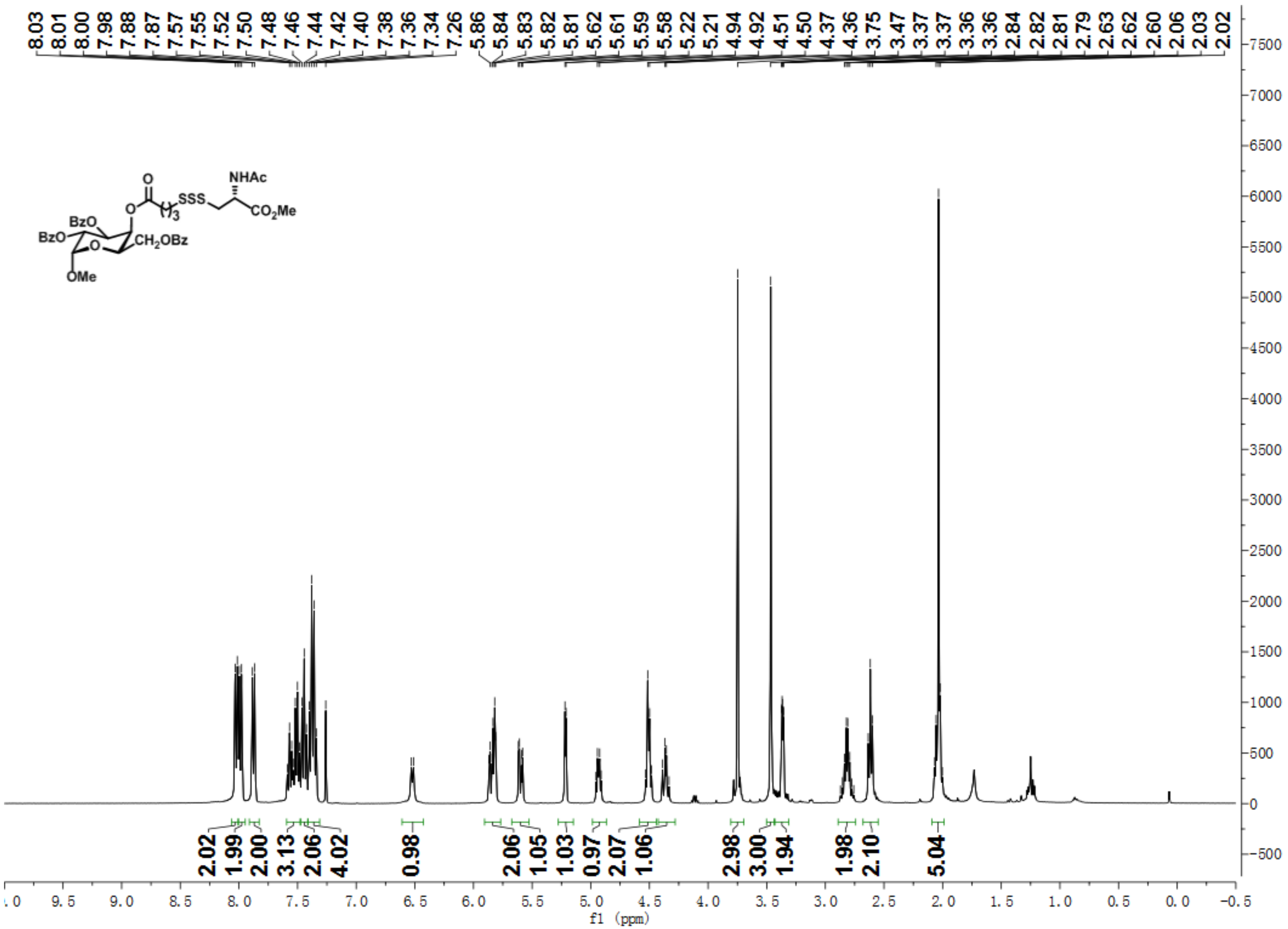

Supplementary Figure 227. ^{1}H NMR spectra for Compound **7y**.

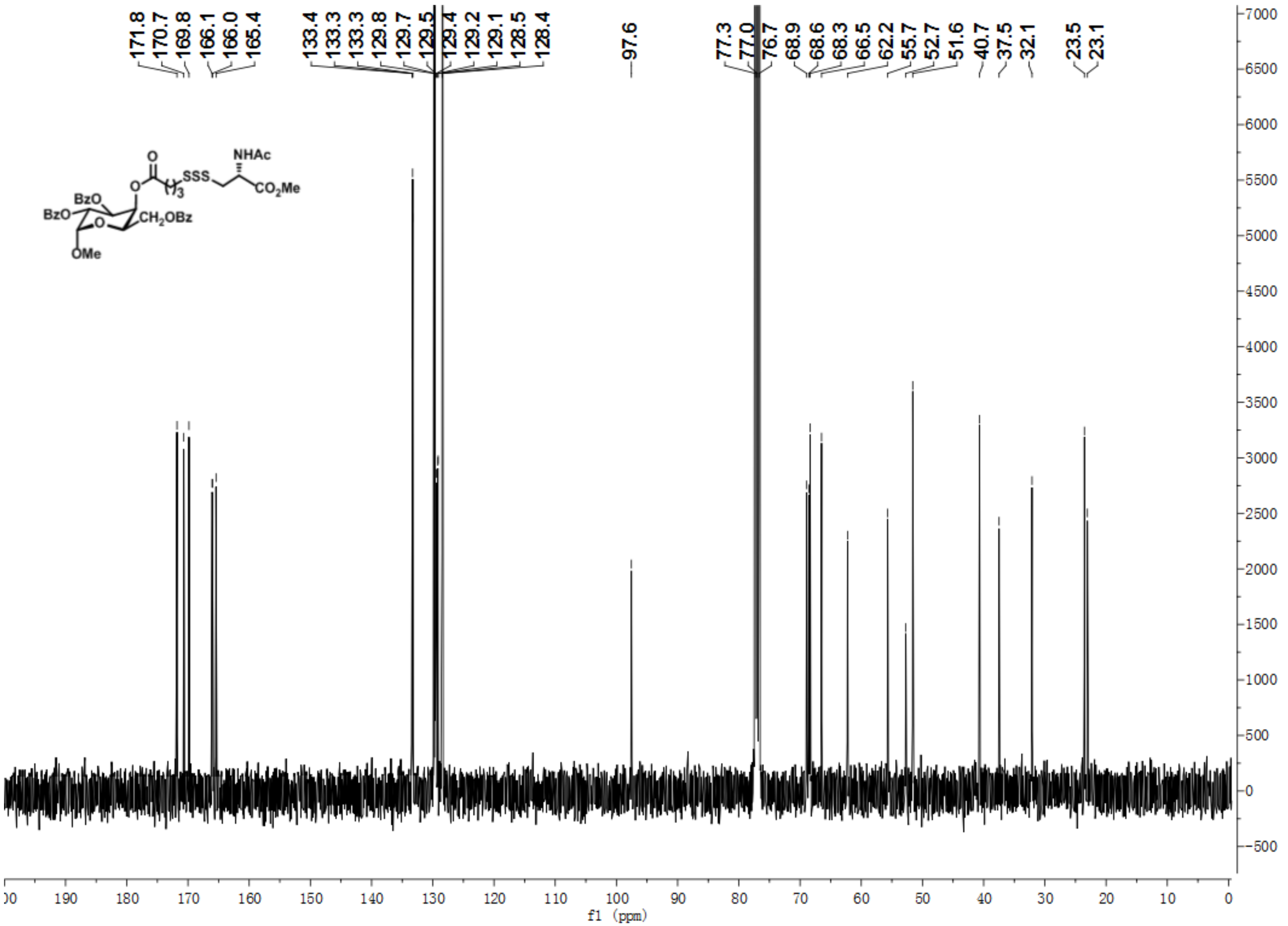

Supplementary Figure 228. ^{13}C NMR spectra for Compound **7y**.

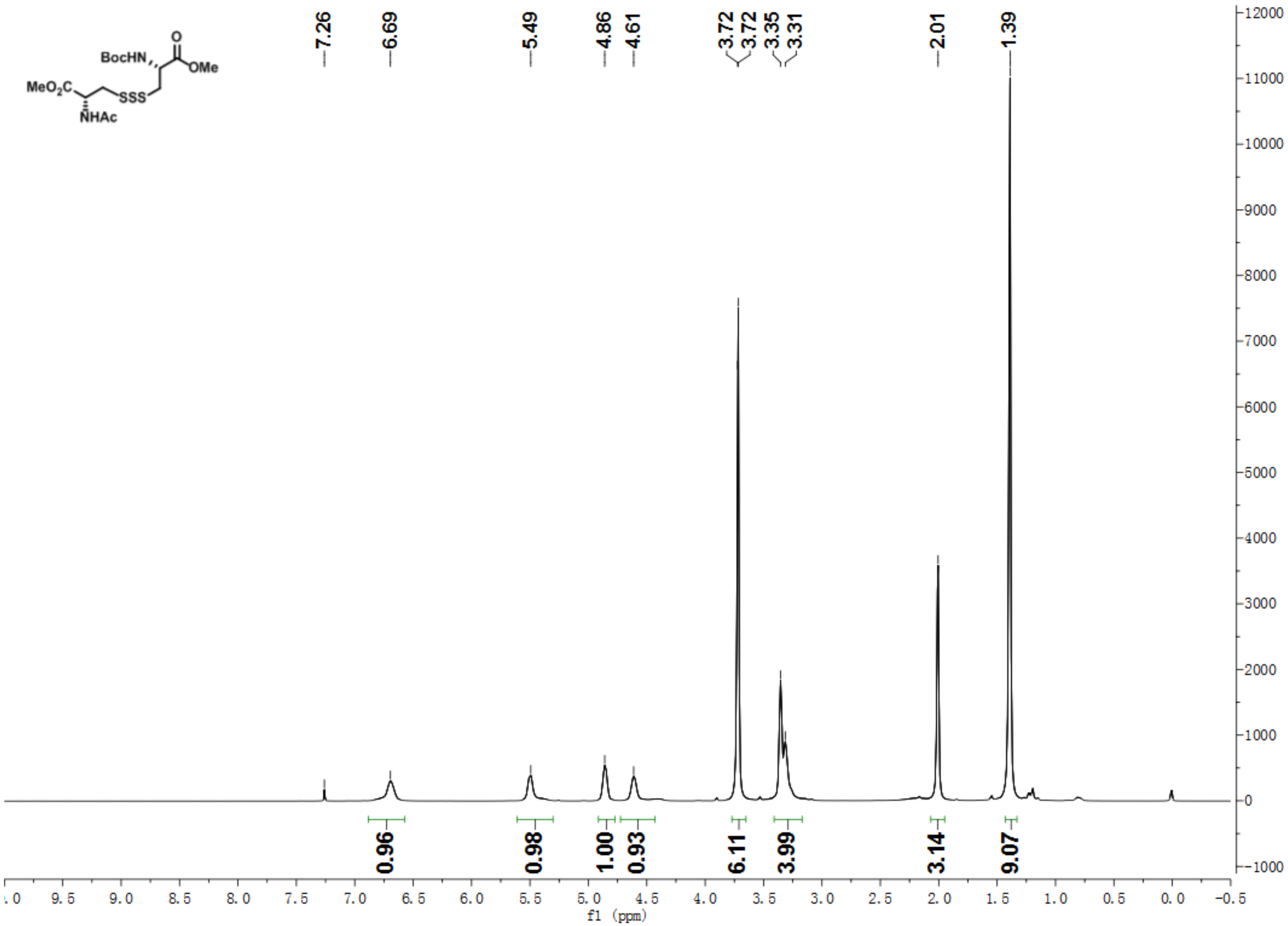

Supplementary Figure 229. ^{1}H NMR spectra for Compound **7z**.

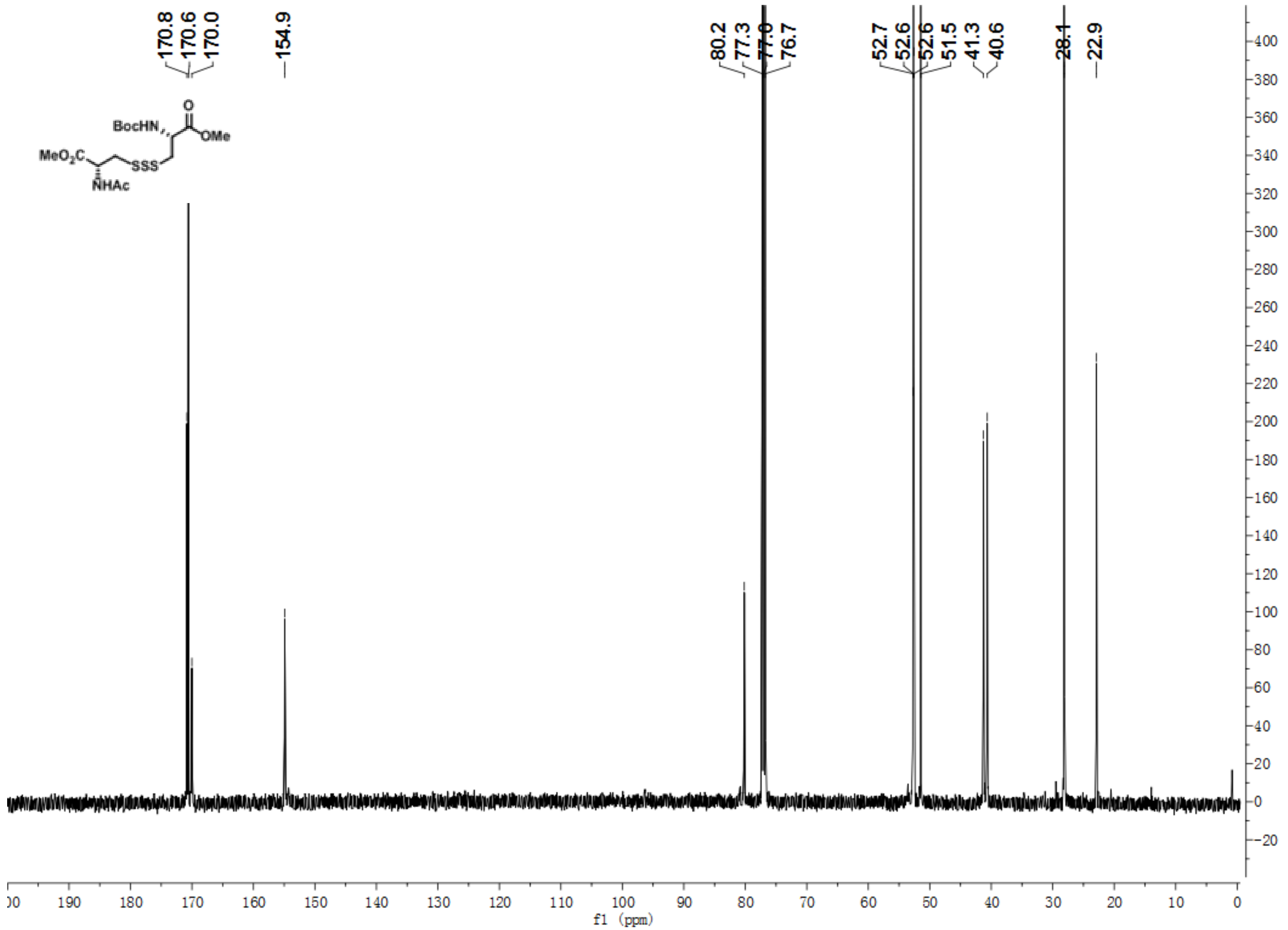

Supplementary Figure 230. ^{13}C NMR spectra for Compound **7z**.

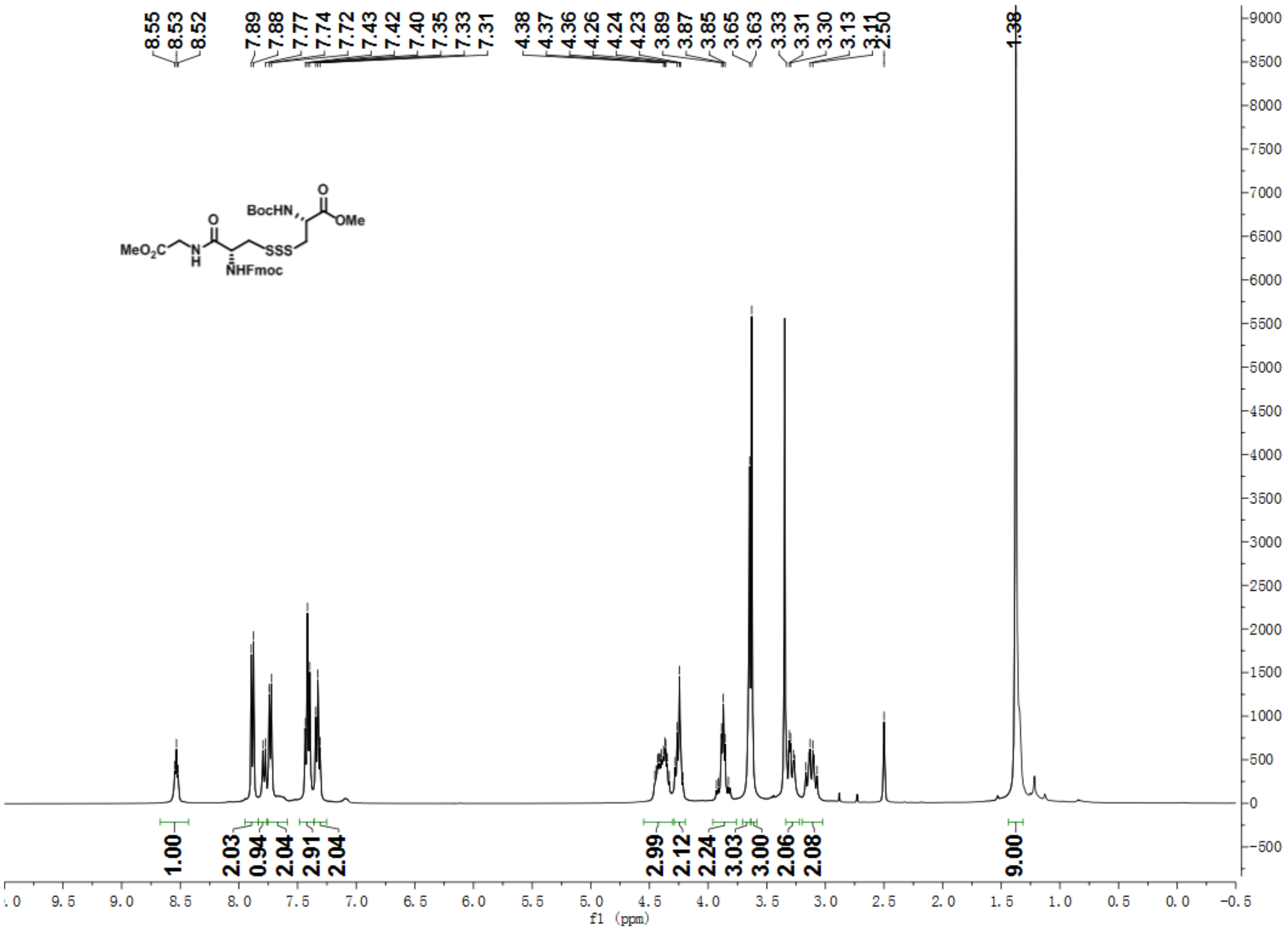

Supplementary Figure 231. ^{1}H NMR spectra for Compound **7aa**.

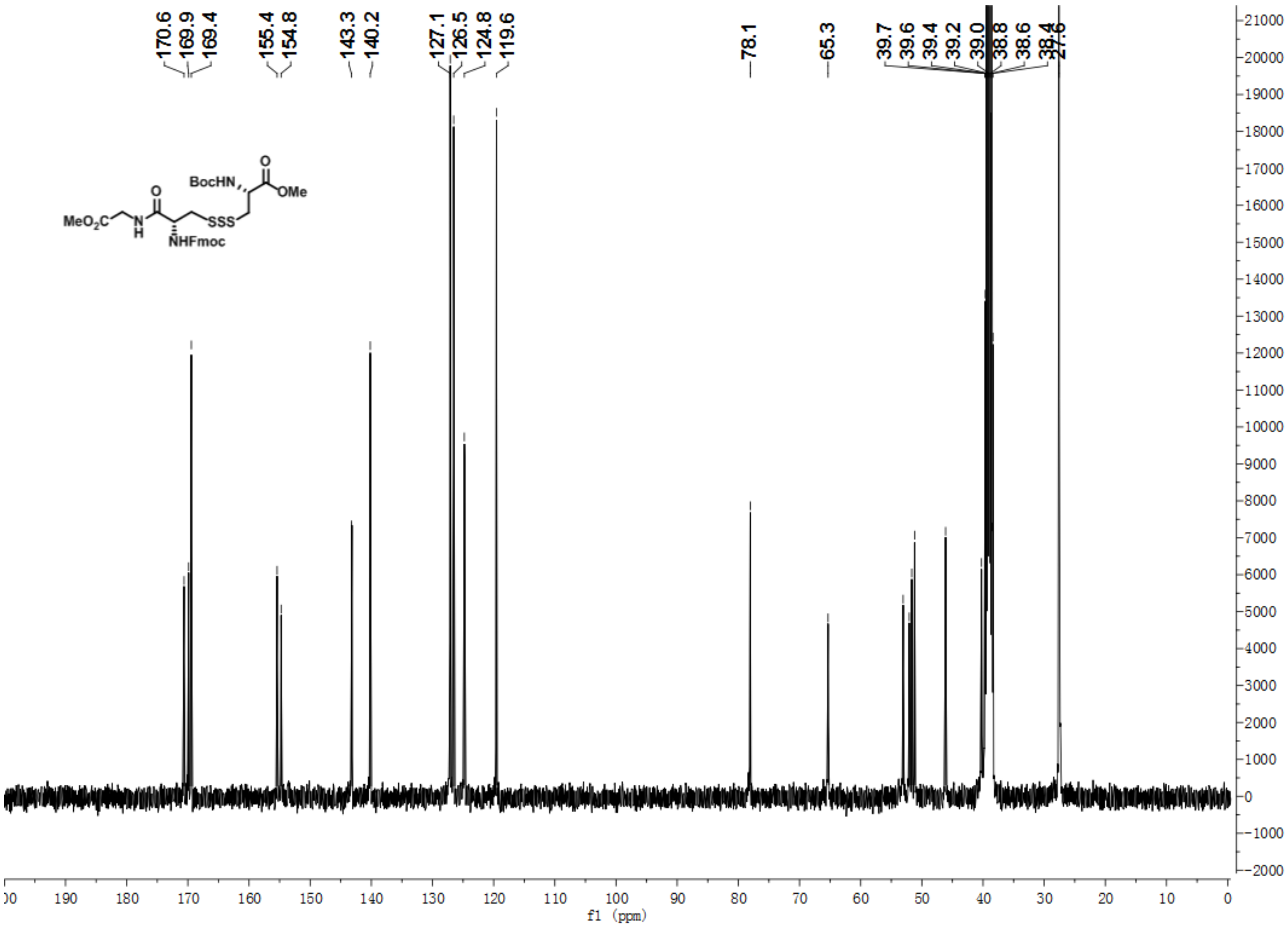

Supplementary Figure 232. ^{13}C NMR spectra for Compound **7aa**.

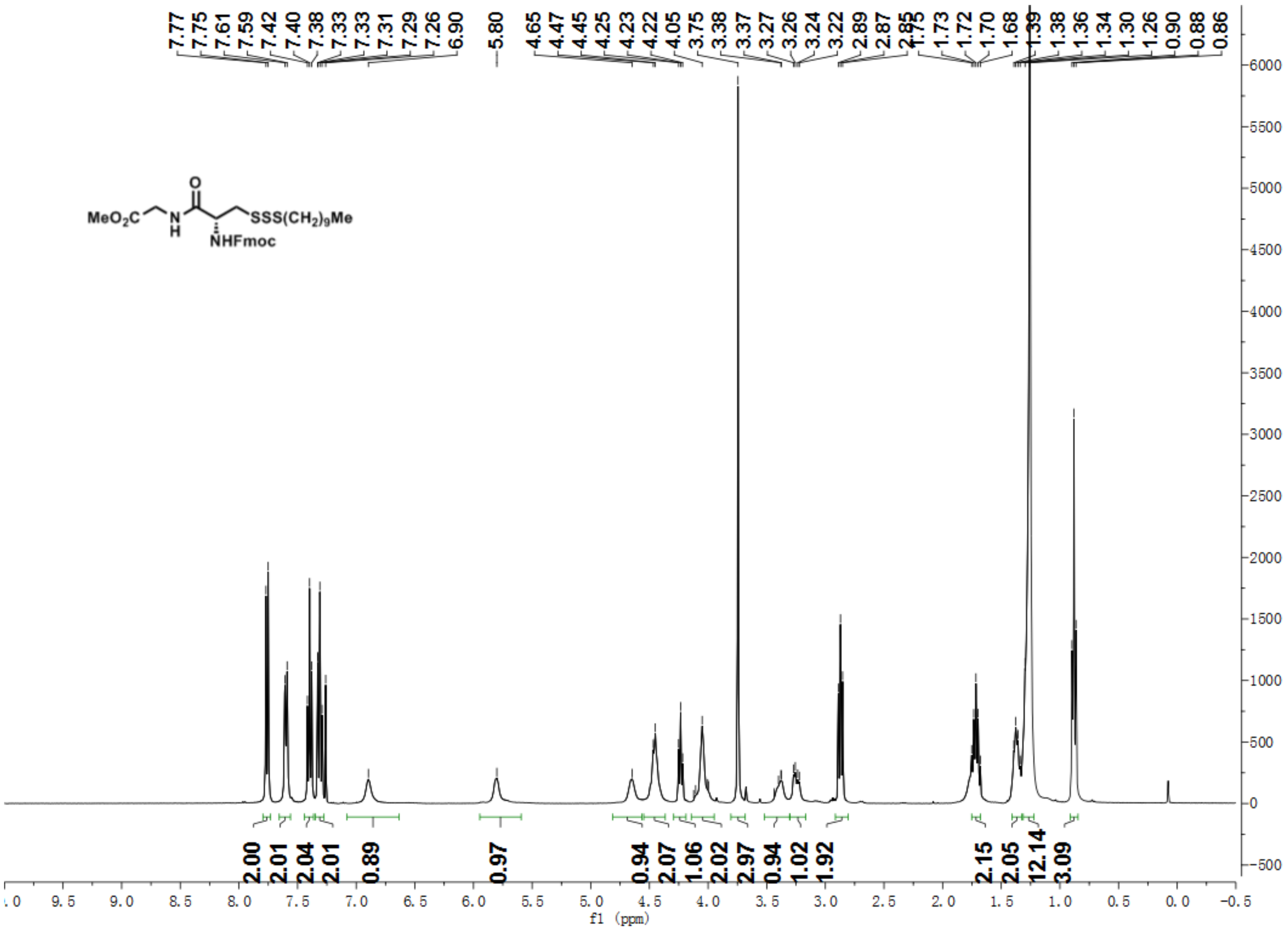

Supplementary Figure 233. ^{1}H NMR spectra for Compound **7ab**.

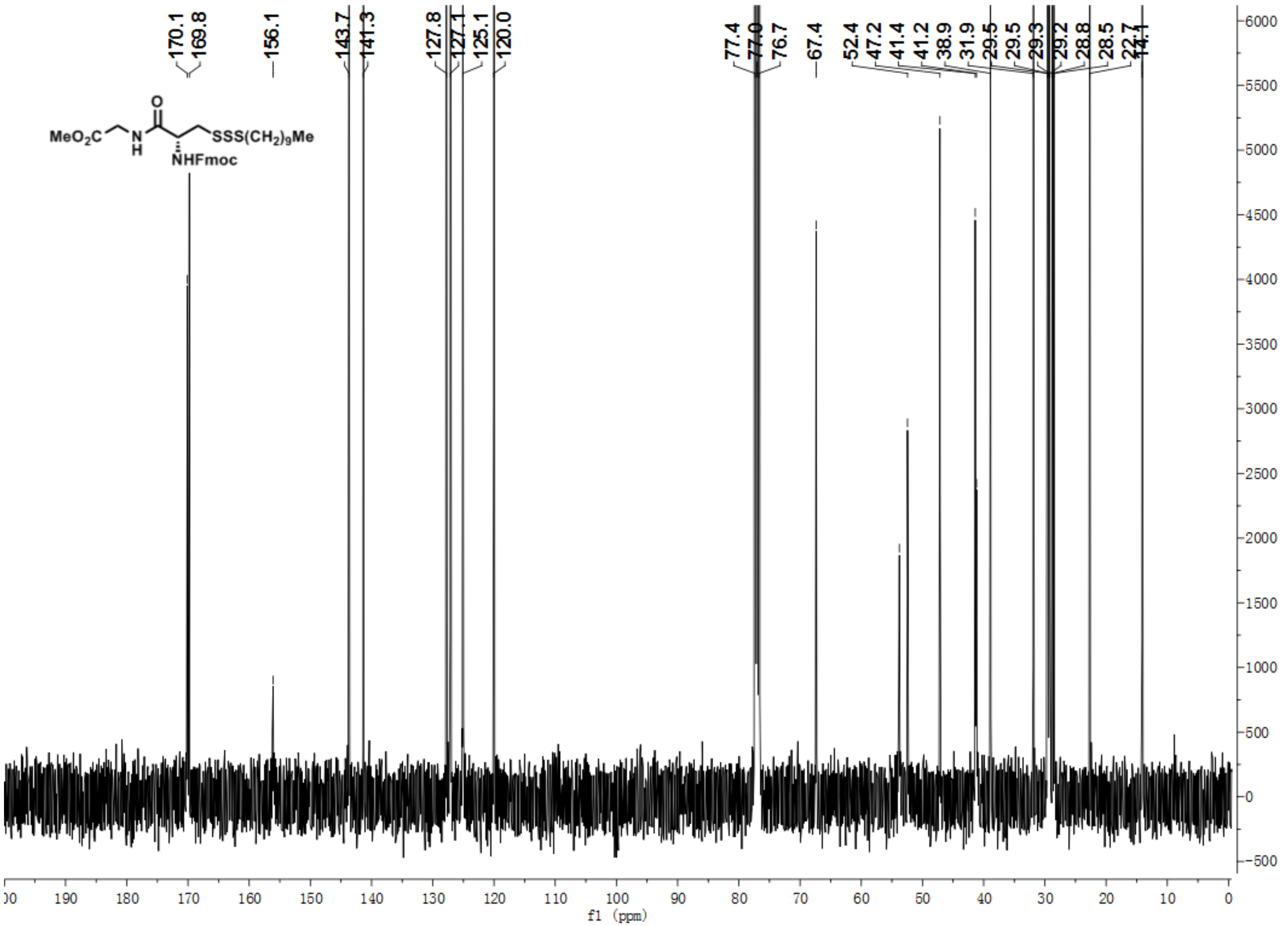

Supplementary Figure 234. ^{13}C NMR spectra for Compound **7ab**.

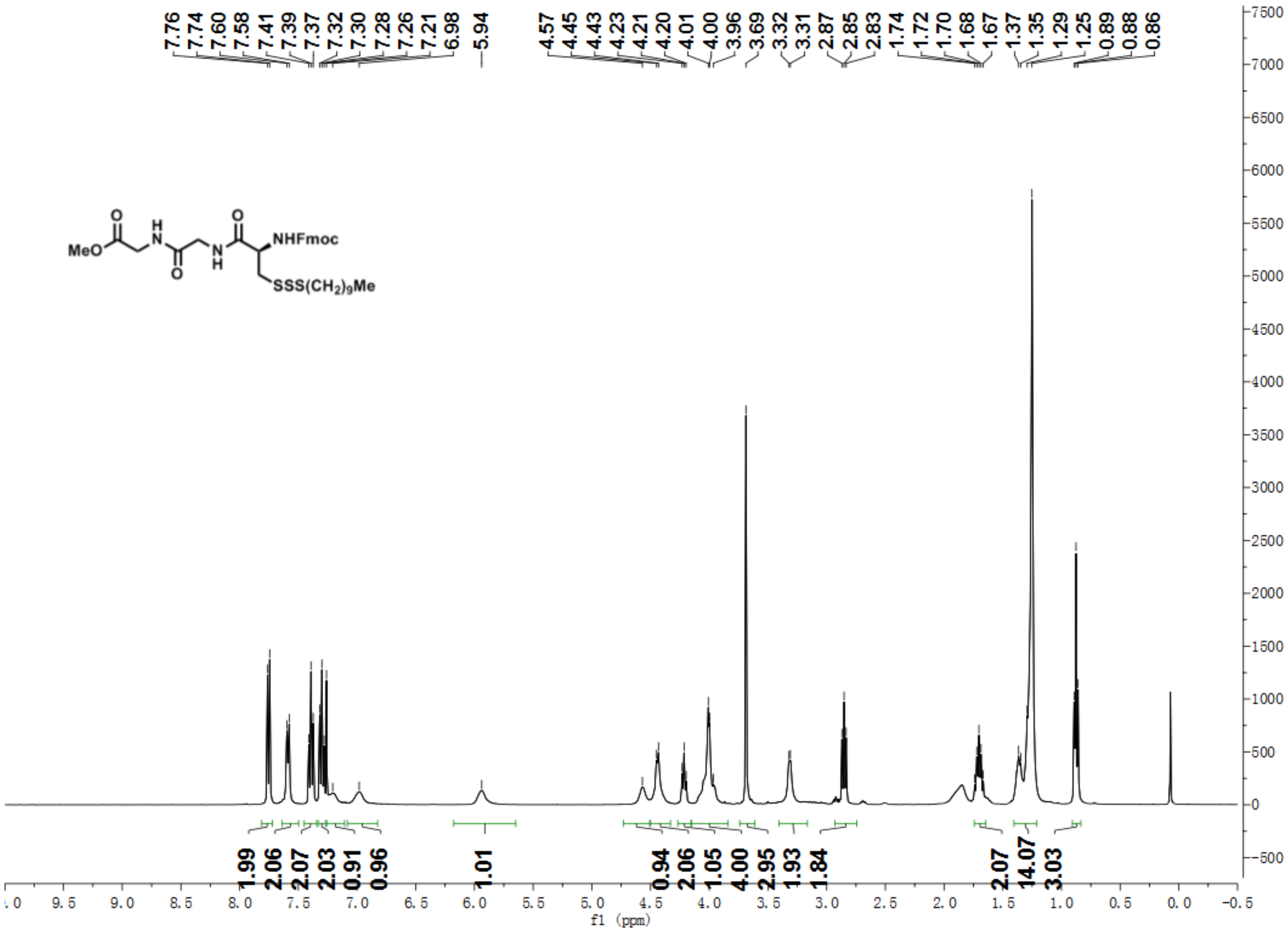

Supplementary Figure 235. ^{1}H NMR spectra for Compound **7ac**.

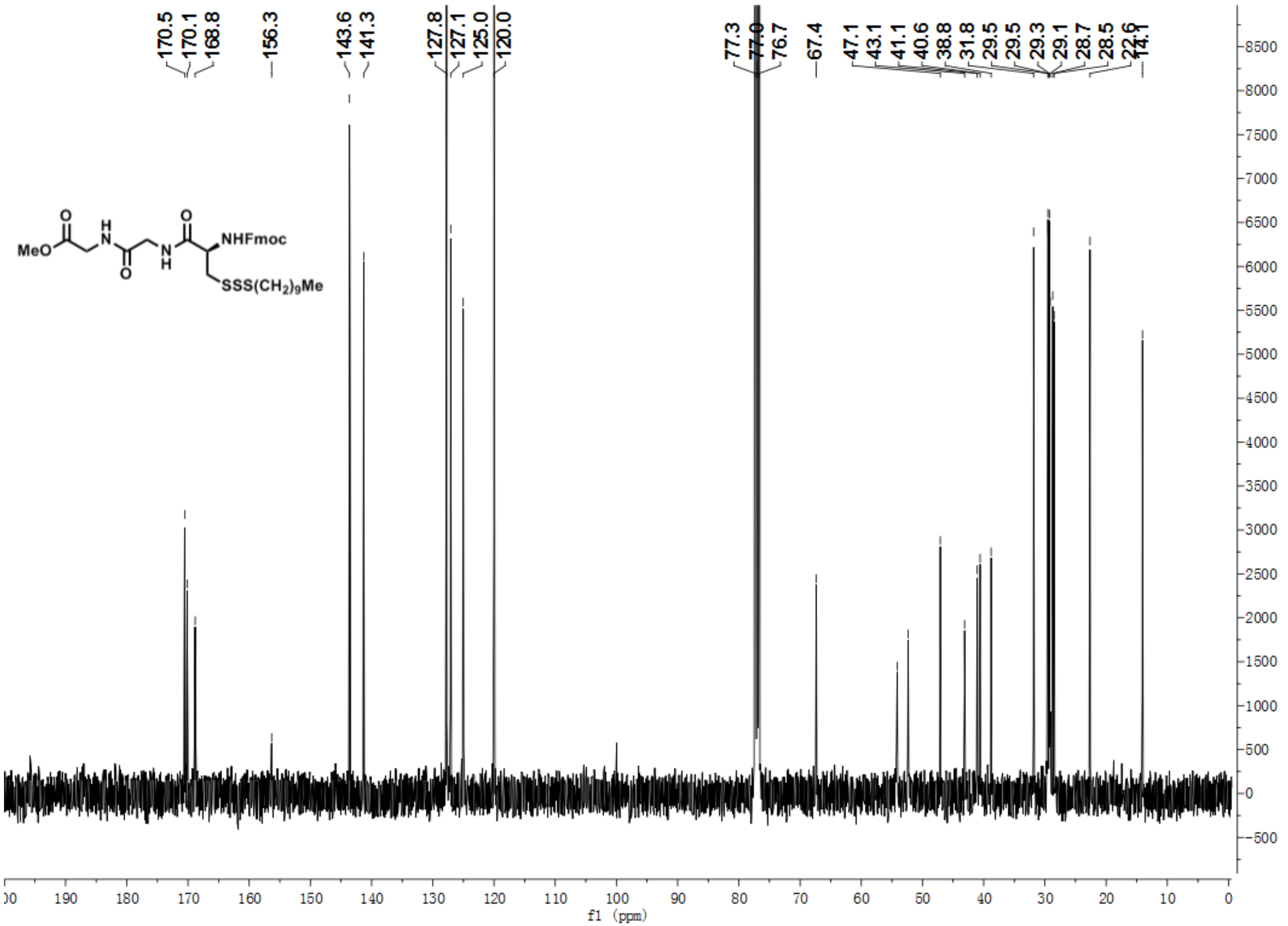

Supplementary Figure 236. ^{13}C NMR spectra for Compound **7ac**.

Supplementary References

1. Xiao, X., Feng, M. & Jiang, X. New design of disulfurating reagent: facile and straightforward pathway to unsymmetrical disulfanes via Cu-catalyzed oxidative cross coupling. *Angew. Chem. Int. Ed.*, **55**, 14121-14121 (2016).

2. Fiori, K. W. & Bois, J. D. Catalytic intermolecular amination of C-H bonds: Method development and mechanistic Insights. *J. Am. Chem. Soc.*, **129**, 526-568 (2007).

3. Stang, P. J., Boehshar, M., Wingert, H. & Kitamura, T. Acetylenic esters. Preparation and characterization of alkynyl carboxylates via polyvalent iodonium species. *J. Am. Chem. Soc.*, **110**, 3272-3278 (1988).
